# Supplementary material for: Research on quantitative evaluation of digital economy policy in China based on the PMC index model
Source: PLoS One. 2024 Feb 15;19(2):e0298312. doi: 10.1371/journal.pone.0298312 (PMC10868804; doi:10.1371/journal.pone.0298312)
Supplement: S1 Appendix — (DOCX) [file pone.0298312.s001.docx]

**数字经济相关政策**

（1）国务院关于数字经济发展情况的报告

2022年10月28日

全国人民代表大会常务委员会：

按照全国人大常委会监督工作计划安排，受国务院委托，现就数字经济发展情况报告如下，请审议。

党的二十大对加快建设数字中国作出重要部署。习近平总书记强调，要站在统筹中华民族伟大复兴战略全局和世界百年未有之大变局的高度，统筹国内国际两个大局、发展安全两件大事，充分发挥海量数据和丰富应用场景优势，促进数字技术和实体经济深度融合，赋能传统产业转型升级，催生新产业新业态新模式，不断做强做优做大我国数字经济。李克强总理指出，要加快数字化发展，打造数字经济新优势，协同推进数字产业化和产业数字化转型，加快数字社会建设步伐，提高数字政府建设水平，营造良好数字生态，建设数字中国。栗战书委员长对相关工作作出重要批示，韩正副总理等国务院领导同志提出明确要求。发展改革委会同有关方面认真贯彻落实党中央、国务院决策部署，牵头建立数字经济发展部际联席会议制度，扎实做好数字经济发展各项工作。

一、我国数字经济发展成效显著

党的十八大以来，我国深入实施网络强国战略、国家大数据战略，先后印发数字经济发展战略、“十四五”数字经济发展规划，有关部门认真落实各项部署，加快推进数字产业化和产业数字化，推动数字经济蓬勃发展。十年来，我国数字经济取得了举世瞩目的发展成就，总体规模连续多年位居世界第二，对经济社会发展的引领支撑作用日益凸显。

（一）数字基础设施实现跨越式发展。统筹谋划新型基础设施建设布局，加快推动高速泛在、天地一体、云网融合、智能敏捷、绿色低碳、安全可控的智能化综合性数字基础设施建设。一是信息通信网络建设规模全球领先。深入实施“宽带中国”战略，建成了全球最大的光纤和移动宽带网络，光缆线路长度从2012年的1479万公里增加到2021年的5481万公里，增长2.7倍。截至2022年7月，我国已许可的5G中低频段频谱资源共计770MHz，许可的中低频段频率资源总量位居世界前列，累计建成开通5G基站达196.8万个。网络基础设施全面向IPv6演进升级，IPv6活跃用户数达6.97亿。深入实施工业互联网创新发展战略，网络、平台、安全体系以及工业互联网标识解析体系基本建成。二是信息通信服务能力大幅提升。我国移动通信实现从“3G突破”到“4G同步”再到“5G引领”的跨越，6G领域的愿景需求研究、关键技术研发、国际交流合作加快。互联网普及率从2012年的42.1%提高到2021年的73%，上网人数达10.32亿人，移动电话用户总数达16.43亿户，其中5G移动电话用户达3.55亿户，约占全球的四分之三。面向中小企业连续4年推进宽带和专线降费，让利超过7000亿元。相比2012年，宽带网络平均下载速率提高近40倍，移动网络单位流量平均资费降幅超95%。三是算力基础设施达到世界领先水平。全国一体化大数据中心体系基本构建，“东数西算”工程加快实施。截至2022年6月，我国数据中心机架总规模超过590万标准机架，建成153家国家绿色数据中心，行业内先进绿色中心电能使用效率降至1.1左右，达到世界领先水平。建成一批国家新一代人工智能公共算力开放创新平台，以低成本算力服务支撑中小企业发展需求。

（二）数字产业创新能力加快提升。深入实施创新驱动发展战略，推进关键核心技术攻关，加快锻造长板、补齐短板，构建自主可控产业生态。一是关键核心技术取得突破。数字技术研发投入逐年上升，量子计算原型机、类脑计算芯片、碳基集成电路等基础前沿领域取得原创性突破，人工智能、区块链、物联网等新兴领域形成一批自主底层软硬件平台和开源社区，关键产品技术创新能力大幅提升，初步形成规模化应用效应。二是产业创新活力不断提升。产业创新能力取得突破性进展，2021年我国数字经济核心产业发明专利授权量达27.6万件，占同期全社会发明专利授权量的39.6%。关键数字技术中人工智能、物联网、量子信息领域发明专利授权量居世界首位。不断发挥金融支持数字经济发展作用，深化股票发行注册制改革，2021年至2022年6月，近150家数字经济相关企业在主板、科创板、创业板完成首发上市，募集资金近3000亿元。持续扩大数字经济产业中长期贷款投放，截至2022年6月末，计算机、通信和其他电子设备制造业中长期贷款余额1.48万亿元。三是数字产业快速成长。数字经济核心产业规模加快增长，全国软件业务收入从2012年2.5万亿元增长到2021年9.6万亿元，年均增速达16.1%。截至2021年，我国工业互联网核心产业规模超过1万亿元，大数据产业规模达1.3万亿元，并成为全球增速最快的云计算市场之一，2012年以来年均增速超过30%。

（三）产业数字化转型提档加速。深入推进企业“上云用数赋智”，加快推动工业互联网、数字商务、智慧农业发展，促进传统产业全方位、全链条转型升级。一是制造业数字化转型持续深化。信息化和工业化融合不断走深向实，企业数字技术应用水平显著提升。截至2022年6月底，我国工业企业关键工序数控化率、数字化研发设计工具普及率分别达55.7%、75.1%，比2012年分别提升31.1个和26.3个百分点。截至2022年7月底，“5G＋工业互联网”建设项目超过3100个，形成一系列新场景、新模式、新业态。全国具备行业、区域影响力的工业互联网平台超过150个，重点平台工业设备连接数超过7900万台套，服务工业企业超过160万家，助力制造业降本增效。智能制造工程深入实施，通过智能化改造，110家智能制造示范工厂的生产效率平均提升32%，资源综合利用率平均提升22%，产品研发周期平均缩短28%，运营成本平均下降19%，产品不良率平均下降24%。二是服务业数字化水平显著提高。全国网络零售市场规模连续9年居于世界首位，从2012年的1.31万亿元增长到2021年的13.1万亿元，年均增速达29.15%。近年来，我国电子商务交易额保持快速增长，由2012年的8万亿元增长至2021年的42.3万亿元，年均增长20.3%。电子商务、移动支付规模全球领先，网约车、网上外卖、数字文化、智慧旅游等市场规模不断扩大。三是农业数字化转型稳步推进。2021年，农作物耕种收综合机械化率超过72%，农机应用北斗终端超过60万台套，产品溯源、智能灌溉、智能温室、精准施肥等智慧农业新模式得到广泛推广，大幅提高了农业生产效率。

（四）公共服务数字化深入推进。加快推进数字政府建设，不断提升数字化公共服务水平。一是“互联网＋政务服务”取得显著成效。全国一体化政务服务平台基本建成，“一网通办”“异地可办”“跨省通办”广泛实践。全国96.68%的办税缴费事项实现“非接触式”办理，全面数字化电子发票试点稳步推进，电子发票服务平台用户数量突破千万级。联合国电子政务调查报告显示，我国电子政务在线服务指数排名从2012年全球第78位提高到目前的第9位，企业、群众办事更加便捷高效。二是数字惠民水平不断提升。全国中小学（含教学点）互联网接入率达100%，住房公积金小程序服务1.64亿缴存人，社会保障卡持卡人数达13.63亿人，电子社保卡领用人数达6.19亿人，全国已审批设置1700多家互联网医院。在抗击新冠肺炎疫情期间，线上教学、互联网诊疗、线上健身等线上服务和无接触配送有力保障了居民需求。三是数字城乡建设纵深推进。新型智慧城市建设取得积极进展，城市信息模型平台和运行管理服务平台建设稳步推进，全国国土空间规划数字化监管平台基本建成，数字孪生流域、水网、水利工程加快建设，智慧交通、应急、广电等建设成效显著。数字乡村建设加快推进，促进乡村宜居宜业、农民富裕富足。全国现有行政村全面实现“村村通宽带”，农村通信难问题得到历史性解决。乡村治理数字化助力强村善治，党务、村务、财务“三务”在线公开率超过70%。乡村信息服务体系逐步健全，累计建设运营益农信息设施46.7万个，提供各类服务9.8亿人次。“互联网＋”农产品出村进城带动农民增收，2021年全国农产品网络零售额达4221亿元。

（五）网络安全保障和数字经济治理水平持续提升。在全国人大的指导推动下，加快健全法律法规体系，强化网络安全机制、手段、能力建设，完善数字经济治理体系，提升网络风险防范能力，推动数字经济健康发展。一是法律和政策制度体系逐步健全。相继颁布实施《网络安全法》《电子商务法》《数据安全法》《个人信息保护法》，修改《反垄断法》，制定新就业形态劳动者权益保障政策。中央全面深化改革委员会第二十六次会议审议通过了《关于构建数据基础制度更好发挥数据要素作用的意见》，初步构建了数据基础制度体系的“四梁八柱”。二是网络安全防护能力持续增强。建立网络安全监测预警和信息通报工作机制，持续加强网络安全态势感知、监测预警和应急处置能力。完善关键信息基础设施安全保护、数据安全保护和网络安全审查等制度，健全国家网络安全标准体系，完善数据安全和个人信息保护认证体系，确保国家网络安全、数据和个人隐私安全。基本建成国家、省、企业三级联动的工业互联网安全技术监测服务体系。三是数字经济治理能力持续提升。建立数字经济部际联席会议等跨部门协调机制，强化部门间协同监管。提升税收征管、银行保险业监管、通关监管、国资监管、数字经济监测和知识产权保护、反垄断、反不正当竞争、网络交易监管等领域的信息化水平，推动“智慧监管”。有序推进金融科技创新监管工具试点、资本市场金融科技创新试点、网络市场监管与服务示范区等工作，探索新型监管机制。

（六）数字经济国际合作行稳致远。习近平总书记在第二届世界互联网大会上提出“构建网络空间命运共同体”理念，深入阐释全球互联网发展治理的“四项原则”“五点主张”，得到国际社会积极响应和广泛认同。习近平总书记在二十国集团（G20）罗马峰会上宣布中方将申请加入《数字经济伙伴关系协定》（DEPA），彰显中国开放姿态。我与各方以此为遵循，推进高质量共建“一带一路”，加强在网络基础设施、数字产业、网络安全等方面的合作，建设21世纪数字丝绸之路，与世界各国人民共享数字经济发展红利。一是积极提出“中国倡议”。提出全球发展倡议，将数字经济作为倡议重点领域。发起《携手构建网络空间命运共同体行动倡议》《“一带一路”数字经济国际合作倡议》《金砖国家数字经济伙伴关系框架》《金砖国家制造业数字化转型合作倡议》等，共同构建和平、安全、开放、合作、有序的网络空间。截至目前，已与16个国家签署“数字丝绸之路”合作谅解备忘录，与24个国家建立“丝路电商”双边合作机制，中国-中东欧国家、中国-中亚五国电子商务合作对话机制建设取得积极进展，中国-东盟信息港、中阿网上丝绸之路建设成效日益显著。二是推动共享“中国红利”。主办“一带一路”国际合作高峰论坛、世界互联网大会等国际会议，搭建全球数字经济交流合作平台。累计建设34条跨境陆缆和多条国际海缆，推动网络基础设施互联互通。中国电商平台助力全球中小企业开拓中国市场，2021年我国跨境电商进出口规模近2万亿元。在非洲20多个国家实施“万村通”项目，共享数字经济发展红利。加强人才交流，举办系列研修研讨，实施学历学位项目，积极分享产业创新升级、数字经济等领域实践经验。三是积极提供“中国方案”。深度参与数字经济国际治理，推进G20、亚太经合组织机制下数字经济合作，推动构建开放、公平、非歧视的数字营商环境，促进数字创新、数字技能与素养、数字化转型等务实合作，引导包容性规则制定。

成绩来之不易，经验弥足珍贵。十年来的成绩，根本在于以习近平同志为核心的党中央的坚强领导，有习近平新时代中国特色社会主义思想的科学指引，习近平总书记的系列重要指示批示，为推动数字经济发展指明了方向，提供了根本遵循；在于中国特色社会主义制度的优越性和先进性，为我国从工业经济向数字经济时代迈进提供了前提和基础；在于充分发挥集中力量办大事的制度优势，为提高数字技术基础研发能力，打好关键核心技术攻坚战提供了条件和可能；在于依托超大规模市场和完备产业体系优势，为数字技术快速大规模应用和迭代升级，新业态新模式蓬勃发展创造了机会和空间。十年来的探索，深化了我们对数字经济发展趋势和规律的认识，只有正确处理好国内和国际、发展和安全、政府和市场的关系，牢牢牵住数字关键核心技术自主创新这个“牛鼻子”，不断推进数字技术与实体经济深度融合，赋能传统产业转型升级，催生新产业新业态新模式，才能为数字经济发展注入源源不断的动力。十年来的实践，坚定了我们推动数字经济发展的信心，只要坚决贯彻落实党中央决策部署，勇于进取、攻坚克难、奋发作为，完全有能力有条件战胜任何艰难险阻、应对各种风险挑战，不断做强做优做大我国数字经济。

二、当前面临的总体形势

当今世界正经历百年未有之大变局，我国数字经济发展的内外部环境正在发生深刻变化，既有错综复杂国际环境带来的新矛盾新挑战，也有我国社会主要矛盾变化带来的新特征新要求。

放眼全球，新一轮科技革命和产业变革深入发展，互联网、大数据、云计算、人工智能、区块链等数字技术创新活跃，数据作为关键生产要素的价值日益凸显，深入渗透到经济社会各领域全过程，数字化转型深入推进，传统产业加速向智能化、绿色化、融合化方向转型升级，新产业、新业态、新模式蓬勃发展，推动生产方式、生活方式发生深刻变化，数字经济成为重组全球要素资源、重塑全球经济结构、改变全球竞争格局的关键力量。世界主要国家都在加紧布局数字经济发展，制定战略规划、加大研发投入，力图打造未来竞争新优势。

立足国内，党的二十大擘画了以中国式现代化全面推进中华民族伟大复兴的使命任务，明确了未来五年是全面建设社会主义现代化国家开局起步的关键时期，作出了加快构建新发展格局，着力推动高质量发展的重要部署，对推进数字技术创新、深化数字化转型、建设数字中国提出了更高要求。我国网民数量、数据资源、数字化应用场景全球领先，人民日益增长的美好生活需要还将催生更大规模、更加多元的内需市场，将为数字经济发展创造无限可能。还要看到，我国数字经济发展的外部环境也在发生深刻变化，个别国家为维护自身科技垄断和霸权地位，遏制打压我国数字技术和数字产业创新发展，我们必须把数字技术的命脉牢牢掌握在自己手中，在科技自立自强上取得更大进展，才能不断提高我国发展的竞争力和持续性，在日趋激烈的国际竞争中把握主动、赢得未来。

与此同时，我国数字经济还存在大而不强、快而不优等问题，突出表现在四个方面：一是关键领域创新能力不足。在操作系统、工业软件、高端芯片、基础材料等领域，技术研发和工艺制造水平落后于国际先进水平。二是传统产业数字化发展相对较慢。农业、工业等传统产业数字化还需深化，部分企业数字化转型存在“不愿”“不敢”“不会”的困境，中小企业数字化转型相对滞后。三是数字鸿沟亟待弥合。不同行业、不同区域、不同群体的数字化基础不同，发展差异明显，甚至有进一步扩大的趋势。四是数字经济治理体系还需完善。适应数字经济发展的规则制度体系有待健全，数据要素基础制度体系尚在建设，既能激发活力又能保障安全的平台经济治理体系需要完善，与相关法律法规配套的各类实施细则亟待出台，数字经济国际治理参与度需进一步提升。跨部门协同、多方参与的治理机制还需完善，治理能力仍需持续提高。

“十四五”时期是我国全面建成小康社会、实现第一个百年奋斗目标之后，乘势而上开启全面建设社会主义现代化国家新征程、向第二个百年奋斗目标进军的第一个五年。面向未来，我们要保持战略定力，充分发挥我国社会主义制度优势、新型举国体制优势、超大规模市场优势，强化目标导向和问题导向，牢牢抓住数字技术发展主动权，把握新一轮科技革命和产业变革发展先机，大力发展数字经济。

三、下一步工作安排

以习近平新时代中国特色社会主义思想为指导，深入贯彻党的二十大精神，按照党中央、国务院决策部署和全国人大常委会审议意见要求，统筹国内和国际、发展和安全，坚持科技自立自强，以数据为关键要素，以推动数字技术与实体经济深度融合为主线，以协同推进数字产业化和产业数字化，赋能传统产业转型升级为重点，以加强数字基础设施建设为基础，以完善数字经济治理体系为保障，不断做强做优做大我国数字经济。到2025年，数字经济迈向全面扩展期，数字化创新引领发展能力大幅提升，智能化水平明显增强，数字技术与实体经济深度融合取得显著成效，具有国际竞争力的数字产业集群初步形成，数字经济治理体系更加完善，我国数字经济竞争力和影响力稳步提升。展望2035年，数字经济迈向繁荣成熟期，力争形成统一公平、竞争有序、成熟完备的数字经济现代市场体系，数字经济发展基础、产业体系发展水平位居世界前列。

（一）集中力量推进关键核心技术攻关，牢牢掌握数字经济发展自主权。以国家战略需求为导向，瞄准全球数字技术基础前沿领域和关键核心技术重大问题，积聚力量进行原创性引领性数字技术攻关。加大集成电路、新型显示、关键软件、人工智能、大数据、云计算等重点领域核心技术创新力度。着力提升基础软硬件、核心电子元器件、关键基础材料和智能制造装备的供给水平，加快锻造长板、补齐短板。打造原创技术策源地，强化原创技术供给，建设新型创新主体，培育创新生态体系。推动数字技术成果转化，以数字技术与各领域融合应用为导向，优化创新成果快速转化机制，打造安全可靠、系统完备的产业发展生态。

（二）适度超前部署数字基础设施建设，筑牢数字经济发展根基。完善信息通信网络建设，深入实施新型基础设施建设专项，持续推动5G网络规模化部署和融合应用，积极培育垂直行业应用场景，建设面向重点区域和重点行业的人工智能基础设施，优化IPv6性能和服务能力。加快建设空间信息基础设施，系统推进北斗产业化重大工程，推进构建民商统筹、集约高效的卫星遥感系统，加强民用遥感卫星应用，建设高低协同的卫星通信系统，推动卫星互联网加快发展。统筹布局绿色智能的数据与算力基础设施，推进“东数西算”工程，建设完善全国一体化大数据中心体系，加快国家绿色数据中心建设。全面发展融合基础设施，加强工业互联网新型基础设施建设，推动车联网部署应用，加快交通、能源、民生、文化、环境等领域基础设施数字化改造。

（三）大力推动数字产业创新发展，打造具有国际竞争力的产业体系。聚焦人工智能、先进计算等重点领域，培育一批掌握关键核心技术、具有国际竞争力的生态主导型企业。加强面向多元化应用场景的技术融合和产品创新，打好关键核心技术攻坚战，提升产业链关键环节竞争力，保障产业链供应链稳定。不断培育壮大云计算、大数据、区块链、工业软件等数字产业，探索建设中国特色的开源生态。推动数字产业集群化发展，高质量建设中国软件名城、名园，提升软件产业集聚度，打造世界级数字经济产业集群。充分激发市场活力，支持互联网企业不断加强技术创新，提升核心竞争力，鼓励平台企业依托市场、技术、数据等优势，赋能实体经济，支持平台企业不断提升国际化发展水平。

（四）加快深化产业数字化转型，释放数字对经济发展的放大、叠加、倍增作用。加快装备数字化发展，组织专项工程，打造标杆企业，发挥数字协同平台等公共服务平台以及龙头骨干企业的赋能作用，带动中小企业数字化改造，提升“上云用数赋智”水平。推动新一代信息技术与制造业融合发展，实施制造业数字化转型发展行动、工业互联网创新发展战略、智能制造工程，深化“5G＋工业互联网”融合发展，推动建设5G全连接工厂，加快培育“专精特新”企业和制造业单项冠军企业，建立工业互联网安全分类分级管理制度，完善公共服务体系，丰富解决方案供给，加快提升工业大数据价值。组织实施数字化绿色化协同转型发展行动计划，推动数字产业绿色低碳发展，加快数字技术赋能行业绿色化转型。推进“三农”综合信息服务，创新发展智慧农业，提升农业数字化水平。大力发展数字商务，持续推进“数商兴农”，创新发展数字内容，培育新兴文化业态，加快商贸、物流、金融等服务业数字化，促进一二三产业融合发展。培育转型支撑服务生态，布局数字化转型促进中心，降低数字化转型门槛。

（五）持续提升数字公共服务水平，不断满足人民美好生活需要。推动政务信息化共建共用，持续提高“互联网＋政务服务”效能，强化政务数据共享和业务协同，提升政务服务标准化、规范化、便利化水平。深化“互联网＋社会服务”，推进教育教学、体育健身、医疗健康、文化服务等领域数字化，提高公共服务资源数字化供给和网络化服务水平。强化就业、社保、养老、托育、助残等重点民生领域社会服务供需对接，加快完善全国统一的社会保险公共服务平台，提升服务资源配置效率和共享水平。加大适老化智能终端供给，加快推动信息无障碍建设，运用数字技术为弱势群体生活、就业、学习等增加便利。统筹推进智慧城市和数字乡村融合发展，大力推进新型城市基础设施建设，加快智能设施和公共服务向乡村延伸覆盖，推进城乡要素双向自由流动，形成以城带乡、共建共享的数字城乡融合发展格局。

（六）不断完善数字经济治理体系，推动数字经济规范健康持续发展。依托数字经济部际联席会议等机制，强化部门协同，加强各项政策协调。加快出台数据要素基础制度及配套政策，推进公共数据、企业数据、个人数据分类分级确权授权使用，构建数据产权、流通交易、收益分配、安全治理制度规则，统筹推进全国数据要素市场体系。持续深化对数字经济发展规律的研究，统筹发展和安全，完善制度体系，规范投资审查流程和办法，细化反垄断执法标准，加强灵活就业和新业态形态劳动者权益保障，增强市场主体活力。支持和引导平台经济规范健康持续发展，完成平台经济专项整改，实施常态化监管，集中推出一批“绿灯”投资案例。完善数字经济统计监测，积极开展数字经济统计核算与分析，完善数字经济统计方法制度，及时反映数字经济发展态势。

（七）全面加强网络安全和数据安全保护，筑牢数字安全屏障。贯彻国家网络安全、数据安全等法律法规，落实网络安全等级保护、关键信息基础设施安全保护等制度要求，强化网络、数据等安全保障体系建设，健全网络应急事件预警通报和应急处置机制，强化网络安全技术措施同步规划、同步建设、同步使用要求，推动网络安全产业高质量发展，增强网络安全防护能力。建立健全数据安全治理体系，完善数据分类分级保护制度，规范数据全生命周期管理，加强数据跨境流动安全管理，推动数据安全产业发展，加强个人信息保护，提升数据安全保障水平，提升防诈反诈技防水平，完善长效治理机制。强化数字经济安全风险综合研判，防范各类风险叠加可能引发的经济风险、技术风险和社会稳定问题。

（八）积极参与数字经济国际合作，推动构建网络空间命运共同体。推进全球发展倡议，落实全球发展高层对话会数字经济领域成果，在联合国贸发会议、金砖国家、上合组织、东盟等多边和区域框架下开展数字经济交流合作。主动参与多边机制和国际组织数字经济议题谈判，积极推进加入《数字经济伙伴关系协定》（DEPA），在世界贸易组织、《区域全面经济伙伴关系协定》（RCEP）等框架下推动电子商务规则构建，开展双多边数字经济治理合作，构建良好国际环境。加快贸易数字化发展，大力发展跨境电商，继续加强跨境电商综试区建设，打造跨境电商产业链和生态圈。务实推进数字经济交流合作，推动“数字丝绸之路”走深走实，拓展“丝路电商”全球布局。鼓励数字经济企业“走出去”，提升国际化运营能力，高质量开展智慧城市、电子商务、移动支付等领域合作。

委员长、各位副委员长、秘书长、各位委员，我们将在以习近平同志为核心的党中央坚强领导下，增强“四个意识”，坚定“四个自信”，做到“两个维护”，切实把思想认识行动统一到习近平总书记重要讲话和指示批示精神上来，坚定不移贯彻落实党中央、国务院决策部署，按照本次会议审议提出的意见，积极采取有效举措，不断做强做优做大我国数字经济，为推动我国经济高质量发展提供有力支撑。

（2）国务院关于印发“十四五”数字经济发展规划的通知

（国发〔2021〕29号）

各省、自治区、直辖市人民政府，国务院各部委、各直属机构：

　　现将《“十四五”数字经济发展规划》印发给你们，请认真贯彻执行。

国务院

2021年12月12日

“十四五”数字经济发展规划

　　数字经济是继农业经济、工业经济之后的主要经济形态，是以数据资源为关键要素，以现代信息网络为主要载体，以信息通信技术融合应用、全要素数字化转型为重要推动力，促进公平与效率更加统一的新经济形态。数字经济发展速度之快、辐射范围之广、影响程度之深前所未有，正推动生产方式、生活方式和治理方式深刻变革，成为重组全球要素资源、重塑全球经济结构、改变全球竞争格局的关键力量。“十四五”时期，我国数字经济转向深化应用、规范发展、普惠共享的新阶段。为应对新形势新挑战，把握数字化发展新机遇，拓展经济发展新空间，推动我国数字经济健康发展，依据《中华人民共和国国民经济和社会发展第十四个五年规划和2035年远景目标纲要》，制定本规划。

　　一、发展现状和形势

　　（一）发展现状

　　“十三五”时期，我国深入实施数字经济发展战略，不断完善数字基础设施，加快培育新业态新模式，推进数字产业化和产业数字化取得积极成效。2020年，我国数字经济核心产业增加值占国内生产总值（GDP）比重达到7.8%，数字经济为经济社会持续健康发展提供了强大动力。

　　信息基础设施全球领先。建成全球规模最大的光纤和第四代移动通信（4G）网络，第五代移动通信（5G）网络建设和应用加速推进。宽带用户普及率明显提高，光纤用户占比超过94%，移动宽带用户普及率达到108%，互联网协议第六版（IPv6）活跃用户数达到4.6亿。

　　产业数字化转型稳步推进。农业数字化全面推进。服务业数字化水平显著提高。工业数字化转型加速，工业企业生产设备数字化水平持续提升，更多企业迈上“云端”。

　　新业态新模式竞相发展。数字技术与各行业加速融合，电子商务蓬勃发展，移动支付广泛普及，在线学习、远程会议、网络购物、视频直播等生产生活新方式加速推广，互联网平台日益壮大。

　　数字政府建设成效显著。一体化政务服务和监管效能大幅度提升，“一网通办”、“最多跑一次”、“一网统管”、“一网协同”等服务管理新模式广泛普及，数字营商环境持续优化，在线政务服务水平跃居全球领先行列。

　　数字经济国际合作不断深化。《二十国集团数字经济发展与合作倡议》等在全球赢得广泛共识，信息基础设施互联互通取得明显成效，“丝路电商”合作成果丰硕，我国数字经济领域平台企业加速出海，影响力和竞争力不断提升。

　　与此同时，我国数字经济发展也面临一些问题和挑战：关键领域创新能力不足，产业链供应链受制于人的局面尚未根本改变；不同行业、不同区域、不同群体间数字鸿沟未有效弥合，甚至有进一步扩大趋势；数据资源规模庞大，但价值潜力还没有充分释放；数字经济治理体系需进一步完善。

　　（二）面临形势。

　　当前，新一轮科技革命和产业变革深入发展，数字化转型已经成为大势所趋，受内外部多重因素影响，我国数字经济发展面临的形势正在发生深刻变化。

　　发展数字经济是把握新一轮科技革命和产业变革新机遇的战略选择。数字经济是数字时代国家综合实力的重要体现，是构建现代化经济体系的重要引擎。世界主要国家均高度重视发展数字经济，纷纷出台战略规划，采取各种举措打造竞争新优势，重塑数字时代的国际新格局。

　　数据要素是数字经济深化发展的核心引擎。数据对提高生产效率的乘数作用不断凸显，成为最具时代特征的生产要素。数据的爆发增长、海量集聚蕴藏了巨大的价值，为智能化发展带来了新的机遇。协同推进技术、模式、业态和制度创新，切实用好数据要素，将为经济社会数字化发展带来强劲动力。

　　数字化服务是满足人民美好生活需要的重要途径。数字化方式正有效打破时空阻隔，提高有限资源的普惠化水平，极大地方便群众生活，满足多样化个性化需要。数字经济发展正在让广大群众享受到看得见、摸得着的实惠。

　　规范健康可持续是数字经济高质量发展的迫切要求。我国数字经济规模快速扩张，但发展不平衡、不充分、不规范的问题较为突出，迫切需要转变传统发展方式，加快补齐短板弱项，提高我国数字经济治理水平，走出一条高质量发展道路。

　　二、总体要求

　　（一）指导思想

　　以习近平新时代中国特色社会主义思想为指导，全面贯彻党的十九大和十九届历次全会精神，立足新发展阶段，完整、准确、全面贯彻新发展理念，构建新发展格局，推动高质量发展，统筹发展和安全、统筹国内和国际，以数据为关键要素，以数字技术与实体经济深度融合为主线，加强数字基础设施建设，完善数字经济治理体系，协同推进数字产业化和产业数字化，赋能传统产业转型升级，培育新产业新业态新模式，不断做强做优做大我国数字经济，为构建数字中国提供有力支撑。

　　（二）基本原则

　　坚持创新引领、融合发展。坚持把创新作为引领发展的第一动力，突出科技自立自强的战略支撑作用，促进数字技术向经济社会和产业发展各领域广泛深入渗透，推进数字技术、应用场景和商业模式融合创新，形成以技术发展促进全要素生产率提升、以领域应用带动技术进步的发展格局。

　　坚持应用牵引、数据赋能。坚持以数字化发展为导向，充分发挥我国海量数据、广阔市场空间和丰富应用场景优势，充分释放数据要素价值，激活数据要素潜能，以数据流促进生产、分配、流通、消费各个环节高效贯通，推动数据技术产品、应用范式、商业模式和体制机制协同创新。

　　坚持公平竞争、安全有序。突出竞争政策基础地位，坚持促进发展和监管规范并重，健全完善协同监管规则制度，强化反垄断和防止资本无序扩张，推动平台经济规范健康持续发展，建立健全适应数字经济发展的市场监管、宏观调控、政策法规体系，牢牢守住安全底线。

　　坚持系统推进、协同高效。充分发挥市场在资源配置中的决定性作用，构建经济社会各主体多元参与、协同联动的数字经济发展新机制。结合我国产业结构和资源禀赋，发挥比较优势，系统谋划、务实推进，更好发挥政府在数字经济发展中的作用。

　　（三）发展目标。

　　到2025年，数字经济迈向全面扩展期，数字经济核心产业增加值占GDP比重达到10%，数字化创新引领发展能力大幅提升，智能化水平明显增强，数字技术与实体经济融合取得显著成效，数字经济治理体系更加完善，我国数字经济竞争力和影响力稳步提升。

　　--数据要素市场体系初步建立。数据资源体系基本建成，利用数据资源推动研发、生产、流通、服务、消费全价值链协同。数据要素市场化建设成效显现，数据确权、定价、交易有序开展，探索建立与数据要素价值和贡献相适应的收入分配机制，激发市场主体创新活力。

　　--产业数字化转型迈上新台阶。农业数字化转型快速推进，制造业数字化、网络化、智能化更加深入，生产性服务业融合发展加速普及，生活性服务业多元化拓展显著加快，产业数字化转型的支撑服务体系基本完备，在数字化转型过程中推进绿色发展。

　　--数字产业化水平显著提升。数字技术自主创新能力显著提升，数字化产品和服务供给质量大幅提高，产业核心竞争力明显增强，在部分领域形成全球领先优势。新产业新业态新模式持续涌现、广泛普及，对实体经济提质增效的带动作用显著增强。

　　--数字化公共服务更加普惠均等。数字基础设施广泛融入生产生活，对政务服务、公共服务、民生保障、社会治理的支撑作用进一步凸显。数字营商环境更加优化，电子政务服务水平进一步提升，网络化、数字化、智慧化的利企便民服务体系不断完善，数字鸿沟加速弥合。

　　--数字经济治理体系更加完善。协调统一的数字经济治理框架和规则体系基本建立，跨部门、跨地区的协同监管机制基本健全。政府数字化监管能力显著增强，行业和市场监管水平大幅提升。政府主导、多元参与、法治保障的数字经济治理格局基本形成，治理水平明显提升。与数字经济发展相适应的法律法规制度体系更加完善，数字经济安全体系进一步增强。

　　展望2035年，数字经济将迈向繁荣成熟期，力争形成统一公平、竞争有序、成熟完备的数字经济现代市场体系，数字经济发展基础、产业体系发展水平位居世界前列。

“十四五”数字经济发展主要指标


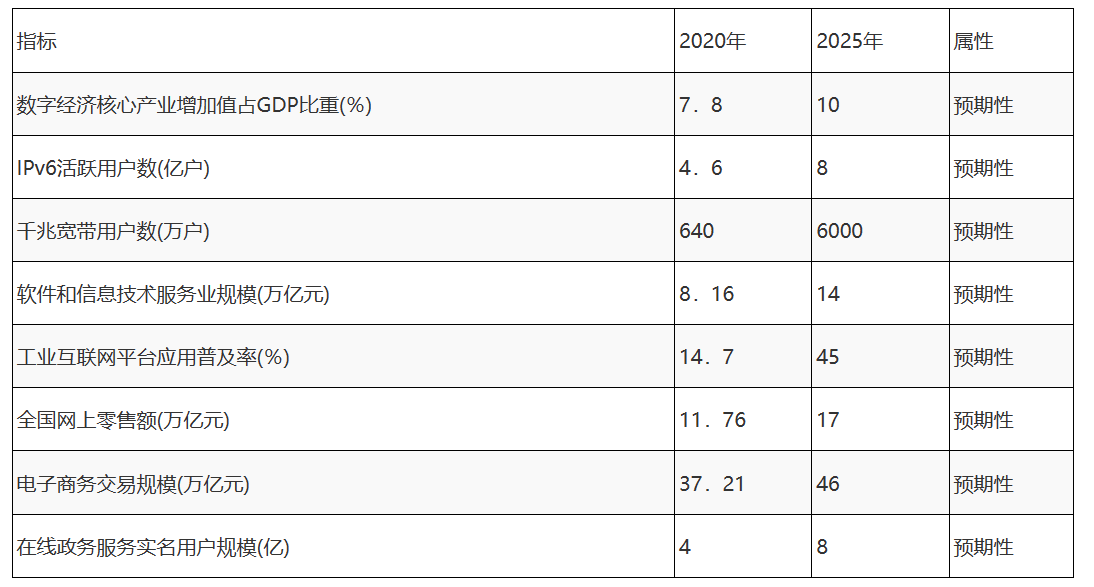


　　三、优化升级数字基础设施

　　（一）加快建设信息网络基础设施。建设高速泛在、天地一体、云网融合、智能敏捷、绿色低碳、安全可控的智能化综合性数字信息基础设施。有序推进骨干网扩容，协同推进千兆光纤网络和5G网络基础设施建设，推动5G商用部署和规模应用，前瞻布局第六代移动通信（6G）网络技术储备，加大6G技术研发支持力度，积极参与推动6G国际标准化工作。积极稳妥推进空间信息基础设施演进升级，加快布局卫星通信网络等，推动卫星互联网建设。提高物联网在工业制造、农业生产、公共服务、应急管理等领域的覆盖水平，增强固移融合、宽窄结合的物联接入能力。

专栏1信息网络基础设施优化升级工程

1．推进光纤网络扩容提速。加快千兆光纤网络部署，持续推进新一代超大容量、超长距离、智能调度的光传输网建设，实现城市地区和重点乡镇千兆光纤网络全面覆盖。

2．加快5G网络规模化部署。推动5G独立组网(SA)规模商用，以重大工程应用为牵引，支持在工业、电网、港口等典型领域实现5G网络深度覆盖，助推行业融合应用。

3．推进IPv6规模部署应用。深入开展网络基础设施IPv6改造，增强网络互联互通能力，优化网络和应用服务性能，提升基础设施业务承载能力和终端支持能力，深化对各类网站及应用的IPv6改造。

4．加速空间信息基础设施升级。提升卫星通信、卫星遥感、卫星导航定位系统的支撑能力，构建全球覆盖、高效运行的通信、遥感、导航空间基础设施体系。

　　（二）推进云网协同和算网融合发展。加快构建算力、算法、数据、应用资源协同的全国一体化大数据中心体系。在京津冀、长三角、粤港澳大湾区、成渝地区双城经济圈、贵州、内蒙古、甘肃、宁夏等地区布局全国一体化算力网络国家枢纽节点，建设数据中心集群，结合应用、产业等发展需求优化数据中心建设布局。加快实施“东数西算”工程，推进云网协同发展，提升数据中心跨网络、跨地域数据交互能力，加强面向特定场景的边缘计算能力，强化算力统筹和智能调度。按照绿色、低碳、集约、高效的原则，持续推进绿色数字中心建设，加快推进数据中心节能改造，持续提升数据中心可再生能源利用水平。推动智能计算中心有序发展，打造智能算力、通用算法和开发平台一体化的新型智能基础设施，面向政务服务、智慧城市、智能制造、自动驾驶、语言智能等重点新兴领域，提供体系化的人工智能服务。

　　（三）有序推进基础设施智能升级。稳步构建智能高效的融合基础设施，提升基础设施网络化、智能化、服务化、协同化水平。高效布局人工智能基础设施，提升支撑“智能＋”发展的行业赋能能力。推动农林牧渔业基础设施和生产装备智能化改造，推进机器视觉、机器学习等技术应用。建设可靠、灵活、安全的工业互联网基础设施，支撑制造资源的泛在连接、弹性供给和高效配置。加快推进能源、交通运输、水利、物流、环保等领域基础设施数字化改造。推动新型城市基础设施建设，提升市政公用设施和建筑智能化水平。构建先进普惠、智能协作的生活服务数字化融合设施。在基础设施智能升级过程中，充分满足老年人等群体的特殊需求，打造智慧共享、和睦共治的新型数字生活。

　　四、充分发挥数据要素作用

　　（一）强化高质量数据要素供给。支持市场主体依法合规开展数据采集，聚焦数据的标注、清洗、脱敏、脱密、聚合、分析等环节，提升数据资源处理能力，培育壮大数据服务产业。推动数据资源标准体系建设，提升数据管理水平和数据质量，探索面向业务应用的共享、交换、协作和开放。加快推动各领域通信协议兼容统一，打破技术和协议壁垒，努力实现互通互操作，形成完整贯通的数据链。推动数据分类分级管理，强化数据安全风险评估、监测预警和应急处置。深化政务数据跨层级、跨地域、跨部门有序共享。建立健全国家公共数据资源体系，统筹公共数据资源开发利用，推动基础公共数据安全有序开放，构建统一的国家公共数据开放平台和开发利用端口，提升公共数据开放水平，释放数据红利。

专栏2数据质量提升工程

1．提升基础数据资源质量。建立健全国家人口、法人、自然资源和空间地理等基础信息更新机制，持续完善国家基础数据资源库建设、管理和服务，确保基础信息数据及时、准确，可靠。

2．培育数据服务商。支持社会化数据服务机构发展，依法依规开展公共资源数据、互联　网数据、企业数据的采集、整理、聚合、分析等加工业务。

3．推动数据资源标准化工作。加快数据资源规划、数据治理、数据资产评估、数据服务、数据安全等国家标准研制，加大对数据管理、数据开放共享等重点国家标准的宣贯力度。

　　（二）加快数据要素市场化流通。加快构建数据要素市场规则，培育市场主体、完善治理体系，促进数据要素市场流通。鼓励市场主体探索数据资产定价机制，推动形成数据资产目录，逐步完善数据定价体系。规范数据交易管理，培育规范的数据交易平台和市场主体，建立健全数据资产评估、登记结算、交易撮合、争议仲裁等市场运营体系，提升数据交易效率。严厉打击数据黑市交易，营造安全有序的市场环境。

专栏3数据要素市场培育试点工程

1．开展数据确权及定价服务试验。探索建立数据资产登记制度和数据资产定价规则，试点开展数据权属认定，规范完善数据资产评估服务。

2．推动数字技术在数据流通中的应用。鼓励企业、研究机构等主体基于区块链等数字技术，探索数据授权使用、数据溯源等应用，提升数据交易流通效率。

3．培育发展数据交易平台。提升数据交易平台服务质量，发展包含数据资产评估、登记结算、交易撮合、争议仲裁等的运营体系，健全数据交易平台报价、询价、竞价和定价机制，探索协议转让、挂牌等多种形式的数据交易模式。

　　（三）创新数据要素开发利用机制。适应不同类型数据特点，以实际应用需求为导向，探索建立多样化的数据开发利用机制。鼓励市场力量挖掘商业数据价值，推动数据价值产品化、服务化，大力发展专业化、个性化数据服务，促进数据、技术、场景深度融合，满足各领域数据需求。鼓励重点行业创新数据开发利用模式，在确保数据安全、保障用户隐私的前提下，调动行业协会、科研院所、企业等多方参与数据价值开发。对具有经济和社会价值、允许加工利用的政务数据和公共数据，通过数据开放、特许开发、授权应用等方式，鼓励更多社会力量进行增值开发利用。结合新型智慧城市建设，加快城市数据融合及产业生态培育，提升城市数据运营和开发利用水平。

　　五、大力推进产业数字化转型

　　（一）加快企业数字化转型升级。引导企业强化数字化思维，提升员工数字技能和数据管理能力，全面系统推动企业研发设计、生产加工、经营管理、销售服务等业务数字化转型。支持有条件的大型企业打造一体化数字平台，全面整合企业内部信息系统，强化全流程数据贯通，加快全价值链业务协同，形成数据驱动的智能决策能力，提升企业整体运行效率和产业链上下游协同效率。实施中小企业数字化赋能专项行动，支持中小企业从数字化转型需求迫切的环节入手，加快推进线上营销、远程协作、数字化办公、智能生产线等应用，由点及面向全业务全流程数字化转型延伸拓展。鼓励和支持互联网平台、行业龙头企业等立足自身优势，开放数字化资源和能力，帮助传统企业和中小企业实现数字化转型。推行普惠性“上云用数赋智”服务，推动企业上云、上平台，降低技术和资金壁垒，加快企业数字化转型。

　　（二）全面深化重点产业数字化转型。立足不同产业特点和差异化需求，推动传统产业全方位、全链条数字化转型，提高全要素生产率。大力提升农业数字化水平，推进“三农”综合信息服务，创新发展智慧农业，提升农业生产、加工、销售、物流等各环节数字化水平。纵深推进工业数字化转型，加快推动研发设计、生产制造、经营管理、市场服务等全生命周期数字化转型，加快培育一批“专精特新”中小企业和制造业单项冠军企业。深入实施智能制造工程，大力推动装备数字化，开展智能制造试点示范专项行动，完善国家智能制造标准体系。培育推广个性化定制、网络化协同等新模式。大力发展数字商务，全面加快商贸、物流、金融等服务业数字化转型，优化管理体系和服务模式，提高服务业的品质与效益。促进数字技术在全过程工程咨询领域的深度应用，引领咨询服务和工程建设模式转型升级。加快推动智慧能源建设应用，促进能源生产、运输、消费等各环节智能化升级，推动能源行业低碳转型。加快推进国土空间基础信息平台建设应用。推动产业互联网融通应用，培育供应链金融、服务型制造等融通发展模式，以数字技术促进产业融合发展。

专栏4重点行业数字化转型提升工程

1．发展智慧农业和智慧水利。加快推动种植业、畜牧业、渔业等领域数字化转型，加强大数据、物联网、人工智能等技术深度应用，提升农业生产经营数字化水平。构建智慧水利体系，以流域为单元提升水情测报和智能调度能力。

2．开展工业数字化转型应用示范。实施智能制造试点示范行动，建设智能制造示范工厂，培育智能制造先行区。针对产业痛点、堵点，分行业制定数字化转型路线图，面向原材料、消费品、装备制造、电子信息等重点行业开展数字化转型应用示范和评估，加大标杆应用推广力度。

3．加快推动工业互联网创新发展。深入实施工业互联网创新发展战略，鼓励工业企业利用5G、时间敏感网络(TSN)等技术改造升级企业内外网，完善标识解析体系，打造若干具有国际竞争力的工业互联网平台，提升安全保障能力，推动各行业加快数字化转型。

4．提升商务领域数字化水平。打造大数据支撑、网络化共享、智能化协作的智慧供应链体系。健全电子商务公共服务体系，汇聚数字赋能服务资源，支持商务领域中小微企业数字化转型升级。提升贸易数字化水平。引导批发零售、住宿餐饮、租赁和商务服务等传统业态积极开展线上线下、全渠道、定制化、精准化营销创新。

5．大力发展智慧物流。加快对传统物流设施的数字化改造升级，促进现代物流业与农业、制造业等产业融合发展。加快建设跨行业、跨区域的物流信息服务平台，实现需求、库存和物流信息的实时共享，探索推进电子提单应用。建设智能仓储体系，提升物流仓储的自动化、智能化水平。

6．加快金融领域数字化转型。合理推动大数据、人工智能、区块链等技术在银行、证券、保险等领域的深化应用，发展智能支付、智慧网点、智能投顾、数字化融资等新模式，稳妥推进数字人民币研发，有序开展可控试点。

7．加快能源领域数字化转型。推动能源产、运、储、销、用各环节设施的数字化升级，实施煤矿、油气田、油气管网、电厂、电网、油气储备库、终端用能等领域设备设施、工艺　流程的数字化建设与改造。推进微电网等智慧能源技术试点示范应用。推动基于供需衔接、生产服务、监督管理等业务关系的数字平台建设，提升能源体系智能化水平，

　　（三）推动产业园区和产业集群数字化转型。引导产业园区加快数字基础设施建设，利用数字技术提升园区管理和服务能力。积极探索平台企业与产业园区联合运营模式，丰富技术、数据、平台、供应链等服务供给，提升线上线下相结合的资源共享水平，引导各类要素加快向园区集聚。围绕共性转型需求，推动共享制造平台在产业集群落地和规模化发展。探索发展跨越物理边界的“虚拟”产业园区和产业集群，加快产业资源虚拟化集聚、平台化运营和网络化协同，构建虚实结合的产业数字化新生态。依托京津冀、长三角、粤港澳大湾区、成渝地区双城经济圈等重点区域，统筹推进数字基础设施建设，探索建立各类产业集群跨区域、跨平台协同新机制，促进创新要素整合共享，构建创新协同、错位互补、供需联动的区域数字化发展生态，提升产业链供应链协同配套能力。

　　（四）培育转型支撑服务生态。建立市场化服务与公共服务双轮驱动，技术、资本、人才、数据等多要素支撑的数字化转型服务生态，解决企业“不会转”、“不能转”、“不敢转”的难题。面向重点行业和企业转型需求，培育推广一批数字化解决方案。聚焦转型咨询、标准制定、测试评估等方向，培育一批第三方专业化服务机构，提升数字化转型服务市场规模和活力。支持高校、龙头企业、行业协会等加强协同，建设综合测试验证环境，加强产业共性解决方案供给。建设数字化转型促进中心，衔接集聚各类资源条件，提供数字化转型公共服务，打造区域产业数字化创新综合体，带动传统产业数字化转型。

专栏5数字化转型支撑服务生态培育工程

1．培育发展数字化解决方案供应商。面向中小微企业特点和需求，培育若干专业型数字化解决方案供应商，引导开发轻量化、易维护、低成本、一站式解决方案。培育若干服务能力强，集成水平高、具有国际竞争力的综合型数字化解决方案供应商。

2．建设一批数字化转型促进中心。依托产业集群、园区、示范基地等建立公共数字化转型促进中心，开展数字化服务资源条件衔接集聚、优质解决方案展示推广、人才招聘及培养、测试试验、产业交流等公共服务。依托企业、产业联盟等建立开放型、专业化数字化转型促进中心，面向产业链上下游企业和行业内中小微企业提供供需撮合、转型咨询、定制化系统解决方案开发等市场化服务。制定完善数字化转型促进中心遴选、评估、考核等标准、程序和机制。

3．创新转型支撑服务供给机制。鼓励各地因地制宜，探索建设数字化转型产品、服务、解决方案供给资源池，搭建转型供需对接平台，开展数字化转型服务券等创新，支持企业加快数字化转型。深入实施数字化转型伙伴行动计划，加快建立高校、龙头企业、产业联盟、行业协会等市场主体资源共享、分工协作的良性机制。

　　六、加快推动数字产业化

　　（一）增强关键技术创新能力。瞄准传感器、量子信息、网络通信、集成电路、关键软件、大数据、人工智能、区块链、新材料等战略性前瞻性领域，发挥我国社会主义制度优势、新型举国体制优势、超大规模市场优势，提高数字技术基础研发能力。以数字技术与各领域融合应用为导向，推动行业企业、平台企业和数字技术服务企业跨界创新，优化创新成果快速转化机制，加快创新技术的工程化、产业化。鼓励发展新型研发机构、企业创新联合体等新型创新主体，打造多元化参与、网络化协同、市场化运作的创新生态体系。支持具有自主核心技术的开源社区、开源平台、开源项目发展，推动创新资源共建共享，促进创新模式开放化演进。

专栏6数字技术创新突破工程

1．补齐关键技术短板。优化和创新“揭榜挂帅”等组织方式，集中突破高端芯片、操作系统、工业软件、核心算法与框架等领域关键核心技术，加强通用处理器、云计算系统和软件关键技术一体化研发。

2．强化优势技术供给。支持建设各类产学研协同创新平台，打通贯穿基础研究、技术研发、中试熟化与产业化全过程的创新链，重点布局5G、物联网、云计算、大数据、人工智能、区块链等领域，突破智能制造、数字孪生、城市大脑、边缘计算、脑机融合等集成技术。

3．抢先布局前沿技术融合创新。推进前沿学科和交叉研究平台建设，重点布局下一代移动通信技术、量子信息、神经芯片、类脑智能、脱氧核糖核酸(DNA)存储、第三代半导体等新兴技术，推动信息、生物、材料、能源等领域技术融合和群体性突破。

　　（二）提升核心产业竞争力。着力提升基础软硬件、核心电子元器件、关键基础材料和生产装备的供给水平，强化关键产品自给保障能力。实施产业链强链补链行动，加强面向多元化应用场景的技术融合和产品创新，提升产业链关键环节竞争力，完善5G、集成电路、新能源汽车、人工智能、工业互联网等重点产业供应链体系。深化新一代信息技术集成创新和融合应用，加快平台化、定制化、轻量化服务模式创新，打造新兴数字产业新优势。协同推进信息技术软硬件产品产业化、规模化应用，加快集成适配和迭代优化，推动软件产业做大做强，提升关键软硬件技术创新和供给能力。

　　（三）加快培育新业态新模式。推动平台经济健康发展，引导支持平台企业加强数据、产品、内容等资源整合共享，扩大协同办公、互联网医疗等在线服务覆盖面。深化共享经济在生活服务领域的应用，拓展创新、生产、供应链等资源共享新空间。发展基于数字技术的智能经济，加快优化智能化产品和服务运营，培育智慧销售、无人配送、智能制造、反向定制等新增长点。完善多元价值传递和贡献分配体系，有序引导多样化社交、短视频、知识分享等新型就业创业平台发展。

专栏7数字经济新业态培育工程

1．持续壮大新兴在线服务。加快互联网医院发展，推广健康咨询、在线问诊、远程会诊等互联网医疗服务，规范推广基于智能康养设备的家庭健康监护、慢病管理、养老护理等新模式。推动远程协同办公产品和服务优化升级，推广电子合同、电子印章、电子签名、电子认证等应用。

2．深入发展共享经济。鼓励共享出行等商业模式创新，培育线上高端品牌，探索错时共享、有偿共享新机制。培育发展共享制造平台，推进研发设计、制造能力、供应链管理等资源共享，发展可计量可交易的新型制造服务。

3．鼓励发展智能经济。依托智慧街区、智慧商圈、智慧园区、智能工厂等建设，加强运营优化和商业模式创新，培育智能服务新增长点。稳步推进自动驾驶、无人配送、智能停车等应用，发展定制化、智慧化出行服务。

4．有序引导新个体经济。支持线上多样化社交、短视频平台有序发展，鼓励微创新、微产品等创新模式。鼓励个人利用电子商务、社交软件、知识分享、音视频网站、创客等新型平台就业创业，促进灵活就业、副业创新。

　　（四）营造繁荣有序的产业创新生态。发挥数字经济领军企业的引领带动作用，加强资源共享和数据开放，推动线上线下相结合的创新协同、产能共享、供应链互通。鼓励开源社区、开发者平台等新型协作平台发展，培育大中小企业和社会开发者开放协作的数字产业创新生态，带动创新型企业快速壮大。以园区、行业、区域为整体推进产业创新服务平台建设，强化技术研发、标准制修订、测试评估、应用培训、创业孵化等优势资源汇聚，提升产业创新服务支撑水平。

　　七、持续提升公共服务数字化水平

　　（一）提高“互联网＋政务服务”效能。全面提升全国一体化政务服务平台功能，加快推进政务服务标准化、规范化、便利化，持续提升政务服务数字化、智能化水平，实现利企便民高频服务事项“一网通办”。建立健全政务数据共享协调机制，加快数字身份统一认证和电子证照、电子签章、电子公文等互信互认，推进发票电子化改革，促进政务数据共享、流程优化和业务协同。推动政务服务线上线下整体联动、全流程在线、向基层深度拓展，提升服务便利化、共享化水平。开展政务数据与业务、服务深度融合创新，增强基于大数据的事项办理需求预测能力，打造主动式、多层次创新服务场景。聚焦公共卫生、社会安全、应急管理等领域，深化数字技术应用，实现重大突发公共事件的快速响应和联动处置。

　　（二）提升社会服务数字化普惠水平。加快推动文化教育、医疗健康、会展旅游、体育健身等领域公共服务资源数字化供给和网络化服务，促进优质资源共享复用。充分运用新型数字技术，强化就业、养老、儿童福利、托育、家政等民生领域供需对接，进一步优化资源配置。发展智慧广电网络，加快推进全国有线电视网络整合和升级改造。深入开展电信普遍服务试点，提升农村及偏远地区网络覆盖水平。加强面向革命老区、民族地区、边疆地区、脱贫地区的远程服务，拓展教育、医疗、社保、对口帮扶等服务内容，助力基本公共服务均等化。加强信息无障碍建设，提升面向特殊群体的数字化社会服务能力。促进社会服务和数字平台深度融合，探索多领域跨界合作，推动医养结合、文教结合、体医结合、文旅融合。

专栏8社会服务数字化提升工程

1．深入推进智慧教育。推进教育新型基础设施建设，构建高质量教育支撑体系。深入推进智慧教育示范区建设，进一步完善国家数字教育资源公共服务体系，提升在线教育支撑服务能力，推动“互联网+教育”持续健康发展，充分依托互联网、广播电视网络等渠道推进优质教育资源覆盖农村及偏远地区学校。

2．加快发展数字健康服务。加快完善电子健康档案、电子处方等数据库，推进医疗数据共建共享。推进医疗机构数字化、智能化转型，加快建设智慧医院，推广远程医疗。精准对接和满足群众多层次、多样化、个性化医疗健康服务需求，发展远程化、定制化、智能比数字健康新业态，提升“互联网+医疗健康”服务水平。

3．以数字化推动文化和旅游融合发展。加快优秀文化和旅游资源的数字化转化和并发，推动景区、博物馆等发展线上数字化体验产品，发展线上演播、云展览、沉浸式体验等新型文旅服务，培育一批具有广泛影响力的数字文化品牌。

4．加快推进智慧社区建设。充分依托已有资源，推动建设集约化、联网规范化、应用智能化、资源社会化，实现系统集成、数据共享和业务协同，更好提供政务、商超、家政、托育、养老、物业等社区服务资源，扩大感知智能技术应用，推动社区服务智能化，提升城乡社区服务效能。

5．提升社会保障服务数字化水平。完善社会保障大数据应用、开展跨地区、跨部门、跨层级数据共享应用，加快实现“跨省通办”。健全风险防控分类管理，加强业务运行监测，构建制度化、常态化数据核查机制。加快推进社保经办数字化转型，为参保单位和个人搭建数字全景图，支持个性服务和精准监管。

　　（三）推动数字城乡融合发展。统筹推动新型智慧城市和数字乡村建设，协同优化城乡公共服务。深化新型智慧城市建设，推动城市数据整合共享和业务协同，提升城市综合管理服务能力，完善城市信息模型平台和运行管理服务平台，因地制宜构建数字孪生城市。加快城市智能设施向乡村延伸覆盖，完善农村地区信息化服务供给，推进城乡要素双向自由流动，合理配置公共资源，形成以城带乡、共建共享的数字城乡融合发展格局。构建城乡常住人口动态统计发布机制，利用数字化手段助力提升城乡基本公共服务水平。

专栏9新型智慧城市和数字乡村建设工程

1．分级分类推进新型智慧城市建设。结合新型智慧城市评价结果和实践成效，遴选有条件的地区建设一批新型智慧城市示范工程，围绕惠民服务、精准治理、产业发展、生态宜居、应急管理等领域打造高水平新型智慧城市样板，着力突破数据融合堆、业务协同难、应急联动难等痛点问题。

2．强化新型智慧城市统筹规划和建设运营。加强新型智慧城市总体规划与顶层设计，创新智慧城市建设、应用、运营等模式，建立完善智慧城市的绩效管理、发展评价、标准规范体系，推进智慧城市规划、设计、建设、运营的一体化、协同化，建立智慧城市长效发展的运营机制。

3．提升信息惠农服务水平。构建乡村综合信息服务体系，丰富市场、科技、金融、就业培训等涉农信息服务内容，推进乡村教育信息化应用，推进农业生产、市场交易、信贷保险、农村生活等数字化应用。

4．推进乡村治理数字化。推动基本公共服务更好向乡村延伸，推进涉农服务事项线上线下一体化办理。推动农业农村大数据应用，强化市场预警、政策评估、监管执法、资源管理、舆情分析、应急管理等领域的决策支持服务。

　　（四）打造智慧共享的新型数字生活。加快既有住宅和社区设施数字化改造，鼓励新建小区同步规划建设智能系统，打造智能楼宇、智能停车场、智能充电桩、智能垃圾箱等公共设施。引导智能家居产品互联互通，促进家居产品与家居环境智能互动，丰富“一键控制”、“一声响应”的数字家庭生活应用。加强超高清电视普及应用，发展互动视频、沉浸式视频、云游戏等新业态。创新发展“云生活”服务，深化人工智能、虚拟现实、8K高清视频等技术的融合，拓展社交、购物、娱乐、展览等领域的应用，促进生活消费品质升级。鼓励建设智慧社区和智慧服务生活圈，推动公共服务资源整合，提升专业化、市场化服务水平。支持实体消费场所建设数字化消费新场景，推广智慧导览、智能导流、虚实交互体验、非接触式服务等应用，提升场景消费体验。培育一批新型消费示范城市和领先企业，打造数字产品服务展示交流和技能培训中心，培养全民数字消费意识和习惯。

　　八、健全完善数字经济治理体系

　　（一）强化协同治理和监管机制。规范数字经济发展，坚持发展和监管两手抓。探索建立与数字经济持续健康发展相适应的治理方式，制定更加灵活有效的政策措施，创新协同治理模式。明晰主管部门、监管机构职责，强化跨部门、跨层级、跨区域协同监管，明确监管范围和统一规则，加强分工合作与协调配合。深化“放管服”改革，优化营商环境，分类清理规范不适应数字经济发展需要的行政许可、资质资格等事项，进一步释放市场主体创新活力和内生动力。鼓励和督促企业诚信经营，强化以信用为基础的数字经济市场监管，建立完善信用档案，推进政企联动、行业联动的信用共享共治。加强征信建设，提升征信服务供给能力。加快建立全方位、多层次、立体化监管体系，实现事前事中事后全链条全领域监管，完善协同会商机制，有效打击数字经济领域违法犯罪行为。加强跨部门、跨区域分工协作，推动监管数据采集和共享利用，提升监管的开放、透明、法治水平。探索开展跨场景跨业务跨部门联合监管试点，创新基于新技术手段的监管模式，建立健全触发式监管机制。加强税收监管和税务稽查。

　　（二）增强政府数字化治理能力。加大政务信息化建设统筹力度，强化政府数字化治理和服务能力建设，有效发挥对规范市场、鼓励创新、保护消费者权益的支撑作用。建立完善基于大数据、人工智能、区块链等新技术的统计监测和决策分析体系，提升数字经济治理的精准性、协调性和有效性。推进完善风险应急响应处置流程和机制，强化重大问题研判和风险预警，提升系统性风险防范水平。探索建立适应平台经济特点的监管机制，推动线上线下监管有效衔接，强化对平台经营者及其行为的监管。

专栏10数字经济治理能力提升工程

1．加强数字经济统计监测。基于数字经济及其核心产业统计分类，界定数字经济统计范围，建立数字经济统计监测制度，组织实施数字经济统计监测。定期开展数字经济核心产业核算，准确反映数字经济核心产业发展规模、速度、结构等情况。探索开展产业数字化发展状况评估。

2．加强重大问题研判和风险预警。整合各相关部门和地方风险监测预警能力，健全完善风险发现、研判会商、协同处置等工作机制，发挥平台企业和专业研究机构等力量的作用，有效监测和防范大数据、人工智能等技术滥用可能引发的经济、社会和道德风险。

3．构建数字服务监管体系。加强对平台治理、人工智能伦理等问题的研究，及时跟踪研判数字技术创新应用发展趋势，推动完善数字中介服务、工业APP、云计算等数字技术和服务监管规则。探索大数据、人工智能、区块链等数字技术在监管领域的应用。强化产权和知识产权保护，严厉打击网络侵权和盗版行为，营造有利于创新的发展环境。

　　（三）完善多元共治新格局。建立完善政府、平台、企业、行业组织和社会公众多元参与、有效协同的数字经济治理新格局，形成治理合力，鼓励良性竞争，维护公平有效市场。加快健全市场准入制度、公平竞争审查机制，完善数字经济公平竞争监管制度，预防和制止滥用行政权力排除限制竞争。进一步明确平台企业主体责任和义务，推进行业服务标准建设和行业自律，保护平台从业人员和消费者合法权益。开展社会监督、媒体监督、公众监督，培育多元治理、协调发展新生态。鼓励建立争议在线解决机制和渠道，制定并公示争议解决规则。引导社会各界积极参与推动数字经济治理，加强和改进反垄断执法，畅通多元主体诉求表达、权益保障渠道，及时化解矛盾纠纷，维护公众利益和社会稳定。

专栏11多元协同治理能力提升工程

1．强化平台治理。科学界定平台责任与义务，引导平台经营者加强内部管理和安全保障，强化平台在数据安全和隐私保护、商品质量保障、食品安全保障、劳动保护等方面的责任，研究制定相关措施，有效防范潜在的技术、经济和社会风险。

2．引导行业自律。积极支持和引导行业协会等社会组织参与数字经济治理，鼓励出台行业标准规范，自律公约，并依法依规参与纠纷处理，规范行业企业经营行为。

3．保护市场主体权益。保护数字经济领域各类市场主体尤其是中小微企业和平台从业人员的合法权益、发展机会和创新活力，规范网络广告、价格标示、宣传促销等行为。

4．完善社会参与机制。拓宽消费者和群众参与渠道，完善社会举报监督机制，推动主管部门、平台经营者等及时回应社会关切，合理引导预期。

　　九、着力强化数字经济安全体系

　　（一）增强网络安全防护能力。强化落实网络安全技术措施同步规划、同步建设、同步使用的要求，确保重要系统和设施安全有序运行。加强网络安全基础设施建设，强化跨领域网络安全信息共享和工作协同，健全完善网络安全应急事件预警通报机制，提升网络安全态势感知、威胁发现、应急指挥、协同处置和攻击溯源能力。提升网络安全应急处置能力，加强电信、金融、能源、交通运输、水利等重要行业领域关键信息基础设施网络安全防护能力，支持开展常态化安全风险评估，加强网络安全等级保护和密码应用安全性评估。支持网络安全保护技术和产品研发应用，推广使用安全可靠的信息产品、服务和解决方案。强化针对新技术、新应用的安全研究管理，为新产业新业态新模式健康发展提供保障。加快发展网络安全产业体系，促进拟态防御、数据加密等网络安全技术应用。加强网络安全宣传教育和人才培养，支持发展社会化网络安全服务。

　　（二）提升数据安全保障水平。建立健全数据安全治理体系，研究完善行业数据安全管理政策。建立数据分类分级保护制度，研究推进数据安全标准体系建设，规范数据采集、传输、存储、处理、共享、销毁全生命周期管理，推动数据使用者落实数据安全保护责任。依法依规加强政务数据安全保护，做好政务数据开放和社会化利用的安全管理。依法依规做好网络安全审查、云计算服务安全评估等，有效防范国家安全风险。健全完善数据跨境流动安全管理相关制度规范。推动提升重要设施设备的安全可靠水平，增强重点行业数据安全保障能力。进一步强化个人信息保护，规范身份信息、隐私信息、生物特征信息的采集、传输和使用，加强对收集使用个人信息的安全监管能力。

　　（三）切实有效防范各类风险。强化数字经济安全风险综合研判，防范各类风险叠加可能引发的经济风险、技术风险和社会稳定问题。引导社会资本投向原创性、引领性创新领域，避免低水平重复、同质化竞争、盲目跟风炒作等，支持可持续发展的业态和模式创新。坚持金融活动全部纳入金融监管，加强动态监测，规范数字金融有序创新，严防衍生业务风险。推动关键产品多元化供给，着力提高产业链供应链韧性，增强产业体系抗冲击能力。引导企业在法律合规、数据管理、新技术应用等领域完善自律机制，防范数字技术应用风险。健全失业保险、社会救助制度，完善灵活就业的工伤保险制度。健全灵活就业人员参加社会保险制度和劳动者权益保障制度，推进灵活就业人员参加住房公积金制度试点。探索建立新业态企业劳动保障信用评价、守信激励和失信惩戒等制度。着力推动数字经济普惠共享发展，健全完善针对未成年人、老年人等各类特殊群体的网络保护机制。

　　十、有效拓展数字经济国际合作

　　（一）加快贸易数字化发展。以数字化驱动贸易主体转型和贸易方式变革，营造贸易数字化良好环境。完善数字贸易促进政策，加强制度供给和法律保障。加大服务业开放力度，探索放宽数字经济新业态准入，引进全球服务业跨国公司在华设立运营总部、研发设计中心、采购物流中心、结算中心，积极引进优质外资企业和创业团队，加强国际创新资源“引进来”。依托自由贸易试验区、数字服务出口基地和海南自由贸易港，针对跨境寄递物流、跨境支付和供应链管理等典型场景，构建安全便利的国际互联网数据专用通道和国际化数据信息专用通道。大力发展跨境电商，扎实推进跨境电商综合试验区建设，积极鼓励各业务环节探索创新，培育壮大一批跨境电商龙头企业、海外仓领军企业和优秀产业园区，打造跨境电商产业链和生态圈。

　　（二）推动“数字丝绸之路”深入发展。加强统筹谋划，高质量推动中国-东盟智慧城市合作、中国-中东欧数字经济合作。围绕多双边经贸合作协定，构建贸易投资开放新格局，拓展与东盟、欧盟的数字经济合作伙伴关系，与非盟和非洲国家研究开展数字经济领域合作。统筹开展境外数字基础设施合作，结合当地需求和条件，与共建“一带一路”国家开展跨境光缆建设合作，保障网络基础设施互联互通。构建基于区块链的可信服务网络和应用支撑平台，为广泛开展数字经济合作提供基础保障。推动数据存储、智能计算等新兴服务能力全球化发展。加大金融、物流、电子商务等领域的合作模式创新，支持我国数字经济企业“走出去”，积极参与国际合作。

　　（三）积极构建良好国际合作环境。倡导构建和平、安全、开放、合作、有序的网络空间命运共同体，积极维护网络空间主权，加强网络空间国际合作。加快研究制定符合我国国情的数字经济相关标准和治理规则。依托双边和多边合作机制，开展数字经济标准国际协调和数字经济治理合作。积极借鉴国际规则和经验，围绕数据跨境流动、市场准入、反垄断、数字人民币、数据隐私保护等重大问题探索建立治理规则。深化政府间数字经济政策交流对话，建立多边数字经济合作伙伴关系，主动参与国际组织数字经济议题谈判，拓展前沿领域合作。构建商事协调、法律顾问、知识产权等专业化中介服务机制和公共服务平台，防范各类涉外经贸法律风险，为出海企业保驾护航。

　　十一、保障措施

　　（一）加强统筹协调和组织实施。建立数字经济发展部际协调机制，加强形势研判，协调解决重大问题，务实推进规划的贯彻实施。各地方要立足本地区实际，健全工作推进协调机制，增强发展数字经济本领，推动数字经济更好服务和融入新发展格局。进一步加强对数字经济发展政策的解读与宣传，深化数字经济理论和实践研究，完善统计测度和评价体系。各部门要充分整合现有资源，加强跨部门协调沟通，有效调动各方面的积极性。

　　（二）加大资金支持力度。加大对数字经济薄弱环节的投入，突破制约数字经济发展的短板与瓶颈，建立推动数字经济发展的长效机制。拓展多元投融资渠道，鼓励企业开展技术创新。鼓励引导社会资本设立市场化运作的数字经济细分领域基金，支持符合条件的数字经济企业进入多层次资本市场进行融资，鼓励银行业金融机构创新产品和服务，加大对数字经济核心产业的支持力度。加强对各类资金的统筹引导，提升投资质量和效益。

　　（三）提升全民数字素养和技能。实施全民数字素养与技能提升计划，扩大优质数字资源供给，鼓励公共数字资源更大范围向社会开放。推进中小学信息技术课程建设，加强职业院校（含技工院校）数字技术技能类人才培养，深化数字经济领域新工科、新文科建设，支持企业与院校共建一批现代产业学院、联合实验室、实习基地等，发展订单制、现代学徒制等多元化人才培养模式。制定实施数字技能提升专项培训计划，提高老年人、残障人士等运用数字技术的能力，切实解决老年人、残障人士面临的困难。提高公民网络文明素养，强化数字社会道德规范。鼓励将数字经济领域人才纳入各类人才计划支持范围，积极探索高效灵活的人才引进、培养、评价及激励政策。

　　（四）实施试点示范。统筹推动数字经济试点示范，完善创新资源高效配置机制，构建引领性数字经济产业集聚高地。鼓励各地区、各部门积极探索适应数字经济发展趋势的改革举措，采取有效方式和管用措施，形成一批可复制推广的经验做法和制度性成果。支持各地区结合本地区实际情况，综合采取产业、财政、科研、人才等政策手段，不断完善与数字经济发展相适应的政策法规体系、公共服务体系、产业生态体系和技术创新体系。鼓励跨区域交流合作，适时总结推广各类示范区经验，加强标杆示范引领，形成以点带面的良好局面。

　　（五）强化监测评估。各地区、各部门要结合本地区、本行业实际，抓紧制定出台相关配套政策并推动落地。要加强对规划落实情况的跟踪监测和成效分析，抓好重大任务推进实施，及时总结工作进展。国家发展改革委、中央网信办、工业和信息化部要会同有关部门加强调查研究和督促指导，适时组织开展评估，推动各项任务落实到位，重大事项及时向国务院报告。

（3）商务部、中央网信办、工业和信息化部关于印发《数字经济对外投资合作工作指引》的通知

（商合函〔2021〕355号）

各省、自治区、直辖市、计划单列市及新疆生产建设兵团商务主管部门、网信办、工业和信息化主管部门，有关中央企业：

　　为全面贯彻党的十九大和十九届二中、三中、四中、五中全会精神，深入实施数字经济战略，推动数字经济对外投资合作高质量发展，更好服务构建新发展格局，我们制定了《数字经济对外投资合作工作指引》（以下简称《指引》），现予印发。请结合实际，抓好贯彻落实。

商务部

　　中央网信办

　　工业和信息化部

　　2021年7月20日

数字经济对外投资合作工作指引

　　为贯彻落实党中央、国务院决策部署，推动数字经济对外投资合作高质量发展，服务构建新发展格局，制定本工作指引。

　　一、重要意义

　　数字经济以数字化的知识和信息为关键生产要素、以现代信息网络为重要载体、以信息通信技术为效率提升重要动力，推动经济发展质量变革、效率变革、动力变革。随着新一轮科技革命和产业变革深入发展，数字经济成为未来全球经济增长的新引擎，各国纷纷将数字经济视为重大战略机遇，国际合作与竞争面临新形势。进入新发展阶段，加快推动数字经济对外投资合作，有利于实现数字产业化和产业数字化，推动传统经济与数字经济相融合，培育经济发展新动能；有利于实现科技自立自强，加快建设科技强国，赢得未来发展新空间；有利于企业参与全球产业链重塑，巩固和创造我国在数字经济领域的发展优势，推动实现更高水平的国内国际双循环。

　　二、总体要求

　　推动数字经济对外投资合作，要以习近平新时代中国特色社会主义思想为指导，深入贯彻落实党的十九大和十九届二中、三中、四中、五中全会精神，立足新发展阶段，贯彻新发展理念，深入实施数字经济战略，坚持企业主体、政府引导、市场运作的原则，坚持创新驱动发展，统筹发展与安全，充分利用两个市场两种资源，着力推动技术进步，着力培育新业态新模式，积极参与全球数字经济合作与竞争，更好服务构建新发展格局。

　　三、重点工作

　　（一）积极融入数字经济全球产业链。鼓励数字经济企业加快布局海外研发中心、产品设计中心，汇聚全球创新要素，加强与境外科技企业在大数据、5G、人工智能、区块链等数字技术领域开展合作，联合研发前沿技术。鼓励开展数字技术产业化国际合作，加快国外先进技术与国内产业化优势对接融合，带动数字产品和服务贸易。鼓励企业加强国际上下游产业链合作，提升国际化发展水平。

　　（二）加快推进数字基础设施建设。鼓励企业抓住海外数字基础设施市场机遇，投资建设陆海光缆、宽带网络、卫星通信等通信网络基础设施，大数据中心、云计算等算力基础设施，人工智能、5G网络等智慧基础设施，在全球范围内提供数字服务。挖掘传统基础设施升级改造市场潜力，积极参与东道国市政、交通、能源、电力、水利等传统基础设施数字化、网络化、智能化升级改造。

　　（三）推动传统产业数字化转型。鼓励制造企业主动参与全球制造业产业链数字化、智能化、自动化、服务化进程，加快应用工业互联网开展境内外网络化协同、个性化定制等先进制造，提升生产和管理效能。加强新业态新模式国际合作，紧扣疫情催生的各国消费需求，加快共享经济、电子支付、远程医疗、普惠金融、智能物流等合作。支持平台型企业走出去，带动中小企业拓展海外市场。

　　（四）优化数字经济走出去布局。加强与发达国家在人工智能、虚拟现实、区块链等领域合作，通过设立实验室、共建孵化平台、建立研发战略联盟等多种方式，积极融入全球先进数字技术发展体系。鼓励企业在条件成熟的“一带一路”共建国家开展技术创新合作和电子政务、远程医疗等应用场景合作。对于基础设施条件不足的发展中国家，加强移动终端合作，提高硬件普及率，推动软件开发与应用。积极参与东道国数字惠民、数字金融、数字治理等民生项目，结合当地复工复产需求开展云经济合作。鼓励龙头企业加强碳排放领域的国际交流合作，提高对碳减排和碳消除技术的投资，积极培育数字化绿色化融合发展新业态新模式。

　　（五）打造具有国际竞争力的数字经济企业。建设数字经济领域国际化领军企业，加大技术研发投入，健全自主创新机制，打造全球产品创新中心、技术服务中心和先进制造管理中心，提升企业全球资源整合能力。精心培育数字经济标杆企业，发挥国内外金融资本作用，聚焦核心科技和创新链短板，引进国际高端人才，加快发展进程。鼓励民营企业走出去，培养一批小而美的数字经济企业，开展本土化经营。

　　（六）建设数字化境外经贸合作区。鼓励数字经济企业参与科技研发型境外经贸合作区建设，打造境外科技创新平台。推动合作区智能设施共享，提升合作区数字化管理服务功能，加强数字技术的融合应用。探索境外经贸合作区与跨境电商综合试验区等园区联动发展，共同推进海外仓建设，推动跨境电商企业等走出去，形成协同效应。打造跨越物理边界的虚拟合作区和产业集群，通过网络平台将区内企业与当地上下游、产供销资源对接，构建跨国数字化产业链供应链。

　　（七）强化数字经济走出去的指导监管。完善数字经济走出去相关制度建设，遵循包容审慎原则，加强对数字经济对外投资合作新业态新模式的监管。完善对外投资备案报告制度，用好境外企业和对外投资联络服务平台，加强监测与分析，做好风险预警。提升对外投资合作数字化管理水平，加强部门间信息共享和协同监管。

　　（八）提高数字经济走出去公共服务水平。加强走出去公共服务平台建设，整合咨询、法律、会计、金融等中介服务资源，增强相关公共服务产品对数字经济走出去指导作用。发挥境外中资企业商协会作用，发布数字经济对外投资合作相关指南。鼓励相关行业商协会在市场调查、咨询评估、行业自律、政策法规等方面，加强对数字经济企业的指导和协调。

　　（九）积极参与国际数字规则标准制定。积极参与高水平双边或区域经贸协定谈判，视情纳入数字经济发展议题。努力在全球数字经济治理进程中发挥重要作用，深入参与联合国、世界贸易组织等数字经济议题谈判，通过二十国集团、金砖国家、亚太经合组织等合作机制推动建立数字经济领域经贸规则，提出中国主张。积极签署标准互认协定，加快与东道国数字标准对接融合，推动我国数字标准走出去。

　　（十）做好数字经济走出去风险防范。鼓励数字经济企业完善内部合规制度，严格落实我国法律法规有关数据出境安全管理的规定，遵守东道国法律法规及国际通行规则，妥善应对数字经济领域审查和监管措施。提高知识产权保护意识，健全数据安全管理制度，采取必要技术措施，保护数据安全和个人信息，支持企业通过法律手段维权。密切跟踪全球数字经济反垄断及加征数字税最新政策动向，做好应对准备。

　　（十一）营造数字经济国际合作良好环境。完善数字经济领域多双边交流合作机制，发挥投资合作工作组作用，加强与有关国家数字经济发展战略对接和政策沟通。鼓励数字经济企业、研究机构、行业协会加强与国际同行交流合作。鼓励数字经济企业积极参与东道国复工复产和民生项目，履行社会责任，培养当地数字经济人才，树立良好口碑。扩大正面宣传，营造数字经济企业良好国际形象，做好舆情应对工作。

　　四、适用范围

　　本工作指引适用于企业在数字经济领域开展对外投资合作，由企业自觉遵守，同时适用于地方商务、网信办、工业和信息化主管部门对我国企业开展对外投资合作的指导、管理和服务。相关单位可在工作中参考。

　　五、组织实施

地方商务、网信办、工业和信息化主管部门应充分认识到数字经济对外投资合作的重要意义，按照工作指引，结合实际加强制度建设，压实工作责任，务求工作实效。重要情况和问题及时向商务部、中央网信办、工业和信息化部报告。

（4）国家发展改革委、教育部、科技部等关于发展数字经济稳定并扩大就业的指导意见

国家发展改革委、教育部、科技部、工业和信息化部、公安部、财政部、人力资源社会保障部、自然资源部、农业农村部、商务部、人民银行、税务总局、市场监管总局、国家统计局、银保监会、证监会、知识产权局、全国总工会、全国工商联关于发展数字经济稳定并扩大就业的指导意见

（发改就业〔2018〕1363号）

各省、自治区、直辖市发展改革委、教育厅（委）、科技厅（委）、工业和信息化（中小企业）主管部门、公安厅（局）、财政厅（局）、人力资源社会保障厅（局）、自然资源主管部门、农业（农牧、农村经济）厅（局、委）、商务主管部门、税务局、工商行政管理局（市场监督管理部门）、统计局、知识产权局、总工会、工商联，中国人民银行上海总部、各分行、营业管理部、省会（首府）城市中心支行，银监局、证监局、保监局、国家统计局各调查总队：

　　随着新一轮科技革命和产业变革孕育兴起，互联网、大数据、云计算、人工智能等数字技术日新月异，以数据资源为重要生产要素、以全要素数字化转型为重要推动力的数字经济蓬勃发展，数字经济领域就业加速增长，新就业形态不断涌现。但同时，数字人才供给缺口大、适应劳动者流动性和就业方式多样化的就业服务及用工管理制度有待完善等问题仍较突出。当前和今后一段时期，要深入贯彻落实党中央、国务院的决策部署，抢抓发展机遇，大力发展数字经济稳定并扩大就业，促进经济转型升级和就业提质扩面互促共进。为此，提出如下意见。

　　一、总体要求

　　（一）指导思想。

　　全面贯彻落实党的十九大精神，以习近平新时代中国特色社会主义思想为指导，紧紧围绕统筹推进“五位一体”总体布局和协调推进“四个全面”战略布局，牢固树立和贯彻落实创新、协调、绿色、开放、共享的发展理念，坚持就业优先战略和积极就业政策，以大力发展数字经济促进就业为主线，以同步推进产业结构和劳动者技能数字化转型为重点，加快形成适应数字经济发展的就业政策体系，大力提升数字化、网络化、智能化就业创业服务能力，不断拓展就业创业新空间，着力实现更高质量和更充分就业，为保障和改善民生、全面建成小康社会、建设社会主义现代化强国提供强大支撑。

　　（二）基本原则。

　　--坚持市场主导、政府引导。既要健全机制，加快消除制度性、体制性障碍，充分发挥市场决定性作用，又要加强政策支持，强化公共服务，更好地发挥政府作用，努力营造发展数字经济促进就业的良好环境。

　　--坚持就业优先、协调发展。要坚持就业优先战略和积极就业政策，把促进充分就业作为经济社会发展优先目标、放在更加突出位置，前瞻性地加强数字人才培养培训，优化人力资本服务，引导更多劳动者有序向数字经济领域转岗就业，在数字经济发展壮大中实现更高质量和更充分就业。

　　--坚持盘活存量、创造增量。坚持以供给侧结构性改革为主线，既要着眼于数字经济发展趋势，加快传统经济数字化转型步伐，盘活存量就业岗位，又要整合资源、优化环境，大力发展互联网、物联网、大数据、云计算、人工智能等新兴产业，不断催生数字化生产新业态新模式，提高新成长劳动力数字技能水平，创造更多新兴就业机会。

　　--坚持包容创新、共建共享。既要加快完善包容创新的政策体系，营造适度宽松的发展环境，又要制定差异化动态化监管政策，创新就业创业服务方式，加快形成适应和引领发展数字经济促进就业的政策环境，使广大劳动者共建共享数字经济发展成果。

　　（三）主要目标。

　　到2025年，伴随数字经济不断壮大，国民数字素养达到发达国家平均水平，数字人才规模稳步扩大，数字经济领域成为吸纳就业的重要渠道。适应数字经济领域就业要求的法律制度框架基本完善，数字化公共就业创业服务能力大幅提升，人力资源市场配置效率明显提高，就业规模不断扩大，就业质量持续改善。

　　二、加快培育数字经济新兴就业机会

　　（四）推动数字产业发展壮大，拓展就业新空间。抓住数字经济发展机遇，深入推进创新驱动发展战略，加快数字基础设施建设，着力发展壮大互联网、物联网、大数据、云计算、人工智能等信息技术产业，做大做强平台企业，在带动经济转型提质过程中创造更多更高质量的新兴就业创业增长点。鼓励数据资源高效利用、开放共享，进一步扩大和升级信息消费，促进电子商务、共享经济等新业态蓬勃发展，培育更多新就业形态，吸纳更多就业。（工业和信息化部、发展改革委牵头，科技部、商务部按职责分工负责）

　　（五）促进传统产业数字化转型，带动更多劳动者转岗提质就业。推动互联网、大数据、人工智能和实体经济深度融合，培育新增长点、形成新动能。深入推进数字技术与制造业融通发展，建立健全工业互联网基础设施体系，大力发展核心工业软件，推动传统制造业加快数字化转型，在提升国际竞争力、拓展产业链条中带动更多劳动力转岗就业。加速传统服务业数字化、网络化转型，提升精准服务、高效服务、智能服务能力，带动更多数字经济领域就业创业。充分应用物联网、大数据等新一代信息技术，促进农业生产、经营、管理、服务数字化，大力发展智慧农业，推进农业全产业链延伸和升级，促进农村一二三产业融合发展，加快乡村振兴步伐，切实提升新农民新主体数字技能。（工业和信息化部、发展改革委、科技部、人力资源社会保障部、农业农村部、商务部按职责分工负责）

　　（六）激发数字经济创新创业活力，厚植就业增长沃土。加大融资政策支持力度，切实落实支持新产业新业态发展、促进大众创业万众创新用地意见，支持互联网龙头企业、各类开发区建设开放平台，建设一批数字产业承接能力强的返乡创业示范基地，营造富有活力的数字经济创新创业环境。进一步深化新三板改革，稳步扩大创新创业公司债试点规模，支持私募股权和创业投资基金投资数字经济领域，增强资本市场支持数字经济创新创业能力。积极引进掌握先进数字技术知识的外国高层次人才，培育推动数字经济创新发展的国际化专家团队。（发展改革委、自然资源部、人力资源社会保障部、工业和信息化部、科技部、商务部、财政部、教育部、人民银行、证监会按职责分工负责）

　　三、持续提升劳动者数字技能

　　（七）强化数字人才教育。深化教育改革，建立健全高等院校、中等职业学校学科专业动态调整机制，加快推进面向数字经济的新工科建设，积极发展数字领域新兴专业，促进计算机科学、数据分析与其他专业学科间的交叉融合，扩大互联网、物联网、大数据、云计算、人工智能等数字人才培养规模。进一步扩大和落实高校专业设置自主权，鼓励高校根据经济社会发展需要和自身办学能力，加大数字领域相关专业人才培养。加强数字人才教育师资力量培养培训，推动实现基础教育、职业教育、高等教育普遍开展数字知识和技能教育，逐步建立健全多层次、多类型数字人才培养体系。加大职业教育数字化资源共建共享力度，加快建设适应数字经济发展的职业教育相关专业教学标准体系，进一步优化中等职业学校信息化相关专业设置。（教育部牵头，发展改革委、人力资源社会保障部按职责分工负责）

　　（八）加强数字技能培训。大规模开展职业技能培训，创新培训方式，探索职业培训包模式。实施国家职业资格目录，做好有关人才资格认证工作。面向新成长劳动力、失业人员等群体，加大大数据分析、软件编程、工业软件、数据安全等数字技能培训规模。引导企业用好用活教育培训经费，加强数字技能在职培训。进一步整合资源，突出重点，打造一批功能突出、资源共享的区域性数字技能公共实训基地。创新公共实训基地运营管理模式，全面提升数字技能实训能力。（人力资源社会保障部牵头，发展改革委按职责分工负责）

　　（九）建设终身学习数字化平台体系。大力发展覆盖职业生涯全过程的数字化终身教育，开发一批大规模在线开放课程平台，推动教育培训机构和部分企业共建在线模块化网络课程，强化课程认证，方便劳动者随时随地利用碎片化时间学习。完善网络平台教学管理系统，开展自适应学习实践项目，构建能动学习的良好环境。（教育部、人力资源社会保障部按职责分工负责）

　　（十）创新人才培养培训方式。加强教育与培训信息化基础设施和数字教育资源建设，提升教育、培训机构网络运行能力，促进教育、培训数据资源共享。开发全网络学习培训方案，实现从课程设计、课程开发、教学过程到教学评估全流程网络化。大力发展“互联网＋”教学和技能培训，积极采用移动技术、互联网、虚拟现实与增强现实、人机互动等数字化教学培训手段，推广微课程、线上线下混合式教学、在线直播等新型教学培训模式。（教育部、人力资源社会保障部按职责分工负责）

　　（十一）吸引社会力量参与数字人才培养培训。深化产教融合、校企合作，探索校企联合培养新模式，推进普通本科高校、职业院校（含技工院校）与科研机构、行业企业协同育人，及时将数字领域先进成果和实用技术转化为教学内容。支持行业企业特别是大型企业举办或参与举办职业院校，支持数字经济大型骨干企业与科研院所共建人才培养基地。加大政府购买服务力度，充分发挥企业、行业协会、培训机构的积极作用，建立多方协同的职业培训规范管理制度和协调发展机制，提升数字人才培养培训能力。（教育部、发展改革委、人力资源社会保障部按职责分工负责）

　　四、大力推进就业创业服务数字化转型

　　（十二）加快推动公共就业创业服务数字化转型。深入实施“互联网＋”公共就业创业服务，加强全国公共就业信息服务平台建设，强化移动端应用，打造集政策解读、业务办理咨询于一体的智能服务体系，充分利用大数据技术，提升精准服务能力，提供全方位公共就业服务。（人力资源社会保障部牵头负责）

　　（十三）鼓励发展数字化人力资源市场服务机构。持续推进和深化商事制度改革，放宽市场准入条件，大力发展“互联网＋”人力资源服务业和基于数字技术的人力资源服务新机构、新业态，加快线下业务向线上转移，线上业务向精准匹配、智能服务转型。引导和鼓励人力资源服务企业加强数字化管理服务系统研发，提升数字化服务水平。（人力资源社会保障部牵头，市场监管总局、科技部按职责分工负责）

　　（十四）做大做强数字经济创新创业服务孵化平台。支持建设一批数字经济创新创业孵化机构。完善以众创空间、孵化器为核心，创业企业、科研机构、金融机构、中介服务机构、资本市场和其他创业资源有机结合的创新创业服务网络。积极推进供应链创新与应用，支持构建以企业为主导，产学研用合作的供应链创新网络，建设跨界交叉领域的创新服务平台。鼓励行业龙头企业、国家级开发区围绕做大做强主业、延伸产业链条，开放企业技术链、供应链、物流链、渠道链，整合培训、金融等相关服务，打造集孵化器和加速器于一体的创客空间。鼓励高校、科研机构发挥技术优势，建设数字经济创新创业服务平台，盘活优质技术资源，服务数字经济创业企业发展。（科技部、发展改革委、人力资源社会保障部、人民银行、工业和信息化部、商务部、教育部按职责分工负责）

　　五、不断完善政策法律体系

　　（十五）不断完善新就业形态劳动用工。按照审慎包容监管、增强劳动力市场灵活性的要求，推动完善劳动法律法规，及时完善新就业形态下的劳动用工政策，切实维护劳动者合法权益。（人力资源社会保障部牵头，全国工商联、全国总工会按职责分工负责）

　　（十六）继续完善适应新就业形态的社会保险参保缴费政策和管理服务机制。全面实施全民参保计划，推动将依托互联网平台实现灵活就业人员纳入社会保障覆盖范围。积极发挥失业保险保生活、防失业、促就业功能作用，拓宽失业保险覆盖范围。适应数字经济新就业形态发展要求，创新社会保险经办服务管理模式，推进“网上社保”，建立全国统一的社会保险公共服务平台。（人力资源社会保障部牵头负责）

　　（十七）加快健全激励机制。支持引导薪酬分配政策向数字技能等高层次人才倾斜，向关键岗位急需紧缺人才倾斜，探索实行项目工资、协议工资和年薪制等灵活多样的薪酬分配方式。加快新就业形态薪酬制度改革，不断完善兼职、一人多岗等灵活就业人员按次提成、计件取酬等工资制度。研究完善适应数字经济特点的税收征管制度。发挥企业主体作用，完善数字人才在人才落户、招聘录用、岗位聘任、职务职级晋升、职称评定、学习进修、休假体检等方面的政策，破除妨碍劳动力、人才社会性流动的体制机制弊端，全面做好数字人才激励工作。（发展改革委、人力资源社会保障部、税务总局、公安部按职责分工负责）

　　六、着力健全保障措施

　　（十八）加强示范引领。支持有条件的地方积极开展发展数字经济促进就业改革示范和探索创新，形成可复制可推广的好经验好做法。（发展改革委牵头，教育部、科技部、工业和信息化部、人力资源社会保障部、商务部按职责分工负责）

　　（十九）注重市场驱动。支持市场资源设立发展数字经济促进就业产业基金，着力培育数字经济市场主体，建立健全数字经济企业融资信息平台，完善融资风险分担机制，在依法合规基础上，开发更多适合数字经济企业的融资工具，更好地满足数字经济企业投融资需求。（发展改革委、科技部、工业和信息化部、人民银行、银保监会按职责分工会负责）

　　（二十）优化发展环境。全面实施市场准入负面清单制度，及时优化完善数字经济管理事项，形成公平透明稳定可预期的制度安排，激发发展数字经济促进就业市场活力。建立健全信息保护、数据交易和共享等方面的相关制度，规范交易行为。发挥行业领军企业示范引领作用，完善企业守信联合激励机制和失信联合惩戒机制，增加企业失信成本，促进数字经济领域就业健康稳定发展。（发展改革委、商务部、工业和信息化部、市场监管总局、知识产权局、人民银行按职责分工负责）

　　（二十一）强化风险应对。统筹发展和安全，提高就业形势感知、科学决策和风险预警能力，稳妥做好风险防控应对。建立健全部门、科研机构、互联网人力资源服务企业联动分析机制，充分利用云计算、大数据等数字技术，推动数字化、信息化监测统计平台建设。探索构建适应数字经济融合业务的国民经济行业分类标准，加强数字经济新产业、新业态、新商业模式和新就业形态统计监测。（发展改革委、人力资源社会保障部、统计局、工业和信息化部按职责分工负责）

　　各地区、各有关部门要强化落实责任，按照本意见要求，结合自身实际，明确目标任务和责任分工，确保各项工作任务落到实处。

国家发展改革委

教　　育　　部

科　　技　　部

工业和信息化部

公　　安　　部

财　　政　　部

人力资源社会保障部

自然资源部

农业农村部

商　　务　　部

人　民　银　行

税　务　总　局

市场监管总局

国家统计局

银　保　监　会

证　　监　　会

知识产权局

全国总工会

全国工商联

2018年9月18日

（5）山西省数字经济促进条例

2022年12月9日山西省第十三届人民代表大会常务委员会第三十八次会议通过

第一章　总 则

　　第一条　为了促进数字经济发展，加快数字经济与实体经济深度融合，建设数字经济强省，全方位推动高质量发展，根据有关法律、行政法规，结合本省实际，制定本条例。

　　第二条　本省行政区域内促进数字经济发展的相关活动，适用本条例。

　　本条例所称数字经济，是指以数据资源为关键要素，以现代信息网络为主要载体，以信息通信技术融合应用、全要素数字化转型为重要推动力，促进公平与效率更加统一的新经济形态。

　　第三条　省人民政府应当加强对全省数字经济发展的领导，统筹部署、组织推进全省数字经济发展工作。

　　县级以上人民政府应当把数字经济发展作为全方位推动高质量发展的重大战略，将数字经济发展纳入国民经济和社会发展规划，建立健全数字经济发展工作协调机制，协调解决数字经济发展中的重大问题。

　　第四条　省人民政府数字经济主管部门负责数字经济发展工作的组织、协调和推进，承担拟定促进数字经济发展战略、规划和重大政策等工作。

　　省人民政府发展改革部门负责数字基础设施建设布局，推进实施数字化发展重大工程和项目等工作。

　　省人民政府工业和信息化部门负责数字产业化发展、工业数字化转型等工作。

　　省人民政府科技部门负责数字经济关键核心技术攻关、创新平台建设和科技成果转化等工作。

　　省人民政府政务信息管理部门负责政务数据统筹管理，组织协调政务数据归集、共享、开放、应用，推进数字政府建设等工作。

　　省通信管理部门负责推进信息通信网络布局、基础设施建设和应用以及相关监督管理等工作。

　　省人民政府其他有关部门应当按照职责分工，做好促进数字经济发展相关工作。

　　设区的市、县（市、区）人民政府数字经济主管部门负责本地区数字经济发展具体工作，其他有关部门应当按照职责分工，做好促进数字经济发展的相关工作。

　　第五条　省人民政府数字经济主管部门应当会同有关部门编制全省数字经济发展规划，报省人民政府批准后组织实施。

　　设区的市、县（市、区）人民政府数字经济主管部门会同有关部门根据全省数字经济发展规划的要求和实际需要，编制本地区数字经济发展规划，报本级人民政府批准后组织实施。

　　数字经济发展规划确定的数字基础设施建设和布局应当纳入国土空间规划，编制市政、交通、通信、电力、公共安全等相关基础设施专项规划应当与数字经济发展专项规划相衔接。

　　第六条　省人民政府应当推动数字经济标准体系建设。

　　鼓励和支持行业协会、产业联盟、企业等牵头或者参与制定数字经济标准，建设数字经济示范或者试点项目。

　　第七条　省人民政府及其有关部门应当加强与有关地区在数字经济领域的交流合作，构建数字经济开放体系，推动数字经济区域协同发展。

　　第八条　县级以上人民政府及有关部门应当加强数字经济领域相关法律、法规，以及技术、知识的教育、培训和宣传普及，提升全民数字素养和数字技能，夯实数字经济发展社会基础。

　　广播、电视、报刊、互联网等媒体应当开展常态化数字经济宣传，刊登、播放公益广告，普及数字经济知识。

　　第九条　鼓励和支持各类市场主体参与数字基础设施投资建设、数字产业化发展、产业数字化转型、数字化治理和服务以及数据资源开发利用等。

　　第十条　县级以上人民政府对在数字经济发展工作中作出突出贡献的单位和个人，按照国家和省有关规定给予表彰和奖励。

第二章　数字基础设施

　　第十一条　县级以上人民政府应当加强数字基础设施建设，布局创新基础设施，推动传统基础设施数字化升级。

　　第十二条　省通信管理部门应当加快通信网络基础设施建设，推进城乡信息通信网络服务能力一体化，提升网络性能和服务能力。

　　第十三条　县级以上人民政府应当组织协调各类社会公共资源向新一代通信网络基站开放共享，强化新一代通信网络基站建设要素资源供给保障。

　　第十四条　省人民政府及发展改革、工业和信息化、通信管理等有关部门应当加强数据中心建设，有序推进算力基础设施规模化、集约化、绿色化发展。

　　第十五条　省人民政府应当完善工业互联网标识解析体系，支持工业互联网标识解析节点建设，推动标识解析与区块链、大数据等技术融合创新，推进标识解析体系与工业互联网应用模式深度融合。

第三章　数字产业化

　　第十六条　省人民政府应当根据数字经济技术、产业发展趋势，结合数字产业发展水平，统筹规划全省数字产业空间布局、功能定位和发展方向，提高数字产业整体竞争力。

　　第十七条　县级以上人民政府应当按照全省数字产业发展要求，结合本地实际，制定政策措施，加快发展电子信息制造业、软件和信息技术服务业、信息通信业、广播电视和卫星传输服务业、互联网服务业，重点推动大数据、信创、通用计算设备、光电信息、半导体、新型化学电池、人工智能及智能装备、网络安全、电磁防护等新一代信息技术产业发展，培育区块链、量子科技、虚拟现实等产业。

　　第十八条　县级以上人民政府应当围绕数字基础设施、数字基础服务、数据融合应用、数据流通交易等大数据产业链条关键环节，培育、引进行业领军企业，壮大大数据产业市场主体，培育大数据产业基地。

　　第十九条　县级以上人民政府应当推动信创产业基地建设，加强信创技术与行业的深度融合应用，推动供给侧与需求侧协同发展，加速信创产业资源高效汇聚；加大核心技术攻关、产业链上下游对接配套、重大项目跟踪服务，提升信创产业现代化水平。

　　第二十条　县级以上人民政府应当围绕智能终端、能源电子、半导体等重点领域，培育、引进行业头部企业，大力发展通信终端、光伏、新型化学电池、新型半导体、计算机、电子专用设备等主导产品，提升关键核心技术，推动形成特色优势电子信息产业集群。

　　第二十一条　县级以上人民政府应当培育互联网平台经济等数字经济新业态和新模式，加大政策引导、支持和保障力度，创新监管理念和方式，建立和健全适应平台经济发展特点的新型监管机制。

　　互联网平台经营者应当建立和健全平台管理规则和制度，依法依约履行商品和服务质量保障、网络安全保障、数据安全保障、消费者权益保护、个人信息保护等方面的义务。

　　第二十二条　县级以上人民政府应当加强数字经济相关产业项目谋划、储备、引进、建设、投产全环节、全链条管理，优化招商引资各项服务，延伸产业链条，吸引配套产业。

　　第二十三条　县级以上人民政府应当引导和支持数字经济核心产业龙头企业、高新技术企业，以及科技型中小企业和专精特新中小企业发展。

　　第二十四条　县级以上人民政府应当加强数字技术在园区的融合应用，支持园区内企业数字化转型；加快数字经济园区建设，推动数字产业向开发区集聚。

　　园区管理机构应当培育或者引进数字产业服务第三方机构、工业互联网平台等企业，向园区内外企业提供数字领域专业化服务。

　　第二十五条　鼓励和支持数字经济领域实验室、技术创新中心、新型研发机构等科技创新平台建设，创建数字经济领域科技企业孵化器、大学科技园和众创空间等线上线下创业平台，推动数字经济科技成果转移转化。

　　第二十六条　鼓励和支持企业联合高等院校、科研院所等，开展信创、大数据、先进计算、量子科技等领域的技术研发和成果应用，提高数字经济核心竞争力。

第四章　产业数字化

　　第二十七条　县级以上人民政府应当通过规划引导、试点示范、政策支持、服务指导等方式，推动新一代信息技术广泛应用，实现工业、农业、服务业等产业数字化转型。

　　第二十八条　县级以上人民政府应当推进工业数字化，加快推动研发设计、生产制造、经营管理、市场服务等全生命周期数字化转型，围绕煤炭、焦化、煤化工、钢铁、装备制造等传统优势产业，推广数字技术融合应用，提升工业企业数字化水平。

　　第二十九条　县级以上人民政府应当加强工业互联网建设，推动重点工业企业对内部网络进行改造升级，提升生产各环节网络化水平；面向装备制造、原材料、新材料、化工等重点行业，推进工业互联网平台建设和应用。

　　第三十条　县级以上人民政府应当加快煤炭产业与数字技术一体化融合发展，创新发展光伏、风电、储能、氢能等新能源领域的数字技术应用，推进智慧煤矿、智能电网、能源互联网建设，以数字化转型驱动能源领域综合改革。

　　第三十一条　县级以上人民政府应当加强智慧矿山建设，开发智慧矿山产品，推进智慧矿山装备制造。

　　第三十二条　县级以上人民政府应当推进制造业数字化转型，加强智能制造支撑服务体系建设，推动制造业重点领域实现智能化制造，形成钢铁、有色、化工、装备制造、消费品等智能化产业集群。

　　第三十三条　县级以上人民政府应当推进农业数字化建设，健全农业信息监测预警、发布机制，推动数字技术在农业各环节的应用，发展智慧农业。

　　县级以上人民政府应当推进重要农产品和小杂粮等特色农产品全产业链大数据建设，建立农产品和投入品电子追溯监管体系。

　　第三十四条　县级以上人民政府应当加快数字商务发展，推动传统商业数字化转型，引导和支持城乡电子商务发展，推进跨境电子商务发展和数字化商贸平台建设。

　　第三十五条　鼓励和支持建设智能交通基础设施，开展智能网联汽车和智慧交通应用示范，推动智慧交通发展。

　　第三十六条　鼓励和支持智慧物流建设，推广仓储数字管理、车辆货物自动匹配、园区智能调度、无人智慧配送等数字化应用。

　　第三十七条　鼓励和支持文化旅游数实融合，建设智慧景区，推动数字化文化生产和消费以及文化遗产资源的数字化转化，发展数字文化产业。

　　第三十八条　鼓励和支持研发设计、检验检测、商务咨询、人力资源等生产性服务业数字化转型，以及数字技术与餐饮、住宿、家政、体育、健康、培训、娱乐等生活性服务业的深度融合。

第五章　治理数字化

　　第三十九条　县级以上人民政府应当推动数字技术在政府治理中的创新应用，推进政府治理数字化，构建协同高效的政府数字化履职能力体系。

　　第四十条　省人民政府及政务信息管理部门应当统筹推进全省一体化在线政务服务平台等建设，推动“一网通办”政务服务、“一网协同”政府运行、“一网统管”省域治理。

　　鼓励和支持推进“多卡合一”、“多码合一”基本公共服务数字化应用，建立多元参与、功能完备的数字化生活网络。

　　第四十一条　省人民政府发展改革、工业和信息化、住房和城乡建设、自然资源以及网信等部门应当统筹指导智慧城市建设，促进数字技术在城市治理中的应用。

　　县级以上人民政府应当支持建设城市大脑、数字孪生模型等应用赋能平台，提升城市综合管理治理水平。

　　第四十二条　县级以上人民政府应当开展智慧社区建设，推动政务服务、公共服务、数字商务向社区延伸，构建文体活动、家政服务、居家养老、儿童和残疾人关爱等数字化创新应用场景。

　　第四十三条　县级以上人民政府应当加强数字乡村建设，构建农业农村数字资源体系，完善农村综合信息服务平台，促进乡村振兴。

　　第四十四条　县级以上人民政府应当推进智慧教育建设，实施教育数字化转型，开展智慧校园建设，优化升级新型教育信息网络。

　　第四十五条　县级以上人民政府应当加强智慧医疗健康体系建设，建设完善全民健康信息平台和传染病监测预警与应急指挥信息平台，推进互联网医院和云药房建设。

　　第四十六条　县级以上人民政府应当推进信息无障碍建设，为老年人、残疾人等运用智能技术困难的特殊群体，提供便利适用的智能化产品和服务措施。

第六章　数据资源的利用与保护

　　第四十七条　数据资源开发利用应当遵守法律、法规，尊重社会公德和伦理，遵守商业道德和职业道德，诚实守信，履行数据安全保护义务，承担社会责任，不得危害国家安全、公共利益，不得损害个人、组织的合法权益。

　　第四十八条　国家机关应当遵循公正、公平、便民的原则，按照规定及时、准确地公开政务数据。依法不予公开的除外。

　　鼓励个人和组织依法开放自有数据。

　　第四十九条　县级以上人民政府应当全面落实政务数据开放共享责任，建立政务数据开放范围动态调整机制，创新政务数据开发利用模式。

　　省人民政府政务信息管理部门应当统筹推进全省政务数据共享利用工作，建立健全政务数据治理机制，建设完善政务数据资源目录体系和共享交换体系，加强政务数据共享工作的监督检查。

　　第五十条　鼓励和支持发展数据治理、数据代理、数据加工、数据标注、数据交易等新兴数据服务，推动数据有序流通交易。

　　第五十一条　省人民政府应当培育发展数据交易平台，构建数据资产市场化流通体系，推动建设山西省大数据交易中心，推进数据交易主体在依法设立的大数据交易平台进行交易。

　　第五十二条　省人民政府应当建立健全数据安全治理体系，推动建立数据分类分级保护制度和数据目录管理制度。

　　县级以上人民政府及有关部门应当依法按照数据分类分级保护制度，对本地区、本部门以及相关行业、领域的数据开展分类分级管理，确定本地区、本部门以及相关行业、领域的重要数据具体目录，对列入目录的数据进行重点保护。

第七章　保障措施

　　第五十三条　省人民政府应当将数字经济发展列入年度绩效考核评价内容，优化考核指标，完善统计体系。

　　第五十四条　省人民政府应当设立数字经济全面发展专项财政资金，重点支持数字基础设施、关键核心技术攻关、科技创新平台建设、典型示范应用、重大项目建设、产业化发展、企业培育和人才培养引进等。

　　设区的市、县（市、区）人民政府应当根据实际情况，安排专项财政资金支持数字经济发展。

　　鼓励和引导社会资本参与数字经济发展。

　　第五十五条　鼓励和引导金融机构在贷款、政策性融资担保以及其他金融服务等方面对数字经济发展给予支持。

　　第五十六条　鼓励和引导保险机构开发适应数字经济发展特点的新型保险产品，为符合政策的数字经济企业和项目贷款提供保证保险和信用保险。

　　第五十七条　鼓励和支持数字经济创新型企业通过股权投资、股票债券发行等方式融资，提高直接融资比例，改善融资结构。支持符合条件的数字经济企业上市融资。

　　第五十八条　鼓励和支持高等院校、职业学校等开设数字经济相关课程，企业事业单位、社会组织设立数字经济人才培训基地，深化校企合作。

　　第五十九条　县级以上人民政府应当制定扶持政策，加强数字经济领域专家人才引进工作，为其在职称评定、住房、落户、医疗保健以及配偶就业、子女入学等方面提供支持。

　　第六十条　县级以上人民政府应当完善政策措施，在土地供给、电力供应、能耗指标、设施保护等方面保障数字经济发展。

　　第六十一条　省人民政府发展改革部门应当会同工业和信息化、财政、科技等部门将计算机、物联网、智能装备、信息技术、网络安全等数字技术产品和服务列入全省创新产品和服务推荐清单。

　　第六十二条　鼓励企业、高等院校、科研机构、行业协会组成数字经济共享服务联合体，整合产学研平台资源，为数字经济发展提供研发、合作、推广、培训等服务。

　　支持行业协会、商会依照法律、法规和章程，加强行业自律，及时反映行业诉求，为数字经济领域市场主体提供信息咨询、宣传培训、市场拓展、权益保护、纠纷处理等方面的服务。

　　第六十三条　支持举办数字经济领域展览、赛事、论坛等活动，搭建数字经济展示、交易、交流、合作平台，宣传数字经济相关企业、产品、服务，推动建立供需对接渠道。

第八章　附则

第六十四条　本条例自2023年1月1日起施行。

(6)北京市数字经济促进条例

2022年11月25日北京市第十五届人民代表大会常务委员会第四十五次会议通过

目　　　录

第一章　总　　则

第二章　数字基础设施

第三章　数据资源

第四章　数字产业化

第五章　产业数字化

第六章　智慧城市建设

第七章　数字经济安全

第八章　保障措施

第九章　附　　则

第一章　总　　则

　　第一条　为了加强数字基础设施建设，培育数据要素市场，推进数字产业化和产业数字化，完善数字经济治理，促进数字经济发展，建设全球数字经济标杆城市，根据有关法律、行政法规，结合本市实际情况，制定本条例。

　　第二条　本市行政区域内数字经济促进相关活动适用本条例。

　　本条例所称数字经济，是指以数据资源为关键要素，以现代信息网络为主要载体，以信息通信技术融合应用、全要素数字化转型为重要推动力，促进公平与效率更加统一的新经济形态。

　　第三条　促进数字经济发展是本市的重要战略。促进数字经济发展应当遵循创新驱动、融合发展、普惠共享、安全有序、协同共治的原则。

　　第四条　市、区人民政府应当加强对数字经济促进工作的领导，建立健全推进协调机制，将数字经济发展纳入国民经济和社会发展规划和计划，研究制定促进措施并组织实施，解决数字经济促进工作中的重大问题。

　　第五条　市经济和信息化部门负责具体组织协调指导全市数字经济促进工作，拟订相关促进规划，推动落实相关促进措施，推进实施重大工程项目；区经济和信息化部门负责本行政区域数字经济促进工作。

　　发展改革、教育、科技、公安、民政、财政、人力资源和社会保障、城市管理、农业农村、商务、文化和旅游、卫生健康、市场监管、广播电视、体育、统计、金融监管、政务服务、知识产权、网信、人才工作等部门按照职责分工，做好各自领域的数字经济促进工作。

　　第六条　市经济和信息化部门会同市场监管等有关部门推进数字经济地方标准体系建设，建立健全关键技术、数据治理和安全合规、公共数据管理等领域的地方标准；指导和支持采用先进的数字经济标准。

　　鼓励行业协会、产业联盟和龙头企业参与制定数字经济国际标准、国家标准、行业标准和地方标准，自主制定数字经济团体标准和企业标准。

　　第七条　市统计部门会同经济和信息化部门完善数字经济统计测度和评价体系，开展数字经济评价，定期向社会公布主要统计结果、监测结果和综合评价指数。

　　第八条　本市为在京单位数字化发展做好服务，鼓励其利用自身优势参与本市数字经济建设；推进京津冀区域数字经济融合发展，在技术创新、基础设施建设、数据流动、推广应用、产业发展等方面深化合作。

第二章　数字基础设施

　　第九条　市、区人民政府及其有关部门应当按照统筹规划、合理布局、集约高效、绿色低碳的原则，加快建设信息网络基础设施、算力基础设施、新技术基础设施等数字基础设施，推进传统基础设施的数字化改造，推动新型城市基础设施建设，并将数字基础设施建设纳入国民经济和社会发展规划和计划、国土空间规划。相关部门做好能源、土地、市政、交通等方面的保障工作。

　　第十条　信息网络基础设施建设应当重点支持新一代高速固定宽带和移动通信网络、卫星互联网、量子通信等，形成高速泛在、天地一体、云网融合、安全可控的网络服务体系。

　　新建、改建、扩建住宅区和商业楼宇，信息网络基础设施应当与主体工程同时设计、同时施工、同时验收并投入使用。信息网络基础设施运营企业享有公平进入市场的权利，不得实施垄断和不正当竞争行为；用户有权自主选择电信业务经营企业。

　　信息网络基础设施管道建设应当统一规划，合理利用城市道路、轨道交通等空间资源，减少和降低对城市道路交通的影响，为信息网络基础设施运营企业提供公平普惠的网络接入服务。

　　第十一条　感知物联网建设应当支持部署低成本、低功耗、高精度、安全可靠的智能化传感器，提高工业制造、农业生产、公共服务、应急管理等领域的物联网覆盖水平。

　　支持建设车路协同基础设施，推进道路基础设施、交通标志标识的数字化改造和建设，提高路侧单元与道路交通管控设施的融合接入能力。

　　第十二条　算力基础设施建设应当按照绿色低碳、集约高效的原则，建设城市智能计算集群，协同周边城市共同建设全国一体化算力网络京津冀国家枢纽节点，强化算力统筹、智能调度和多样化供给，提升面向特定场景的边缘计算能力，促进数据、算力、算法和开发平台一体化的生态融合发展。

　　支持对新建数据中心实施总量控制、梯度布局、区域协同，对存量数据中心实施优化调整、技改升级。

　　第十三条　新技术基础设施建设应当统筹推进人工智能、区块链、大数据、隐私计算、城市空间操作系统等。支持建设通用算法、底层技术、软硬件开源等共性平台。

　　对主要使用财政资金形成的新技术基础设施，项目运营单位应当在保障安全规范的前提下，向社会提供开放共享服务。

　　第十四条　除法律、行政法规另有规定外，数字基础设施建设可以采取政府投资、政企合作、特许经营等多种方式；符合条件的各类市场主体和社会资本，有权平等参与投资、建设和运营。

第三章　数据资源

　　第十五条　本市加强数据资源安全保护和开发利用，促进公共数据开放共享，加快数据要素市场培育，推动数据要素有序流动，提高数据要素配置效率，探索建立数据要素收益分配机制。

　　第十六条　公共数据资源实行统一的目录管理。市经济和信息化部门应当会同有关部门制定公共数据目录编制规范，有关公共机构依照规范及有关管理规定，编制本行业、本部门公共数据目录，并按照要求向市级大数据平台汇聚数据。公共机构应当确保汇聚数据的合法、准确、完整、及时，并探索建立新型数据目录管理方式。

　　本条例所称公共机构，包括本市各级国家机关、经依法授权具有管理公共事务职能的组织。本条例所称公共数据，是指公共机构在履行职责和提供公共服务过程中处理的各类数据。

　　第十七条　市人民政府建立全市公共数据共享机制，推动公共数据和相关业务系统互联互通。

　　市大数据中心具体负责公共数据的汇聚、清洗、共享、开放、应用和评估，通过集中采购、数据交换、接口调用等方式，推进非公共数据的汇聚，建设维护市级大数据平台、公共数据开放平台以及自然人、法人、信用、空间地理、电子证照、电子印章等基础数据库，提升跨部门、跨区域和跨层级的数据支撑能力。

　　区人民政府可以按照全市统一规划，建设本区域大数据中心，将公共数据资源纳入统一管理。

　　第十八条　市经济和信息化部门、区人民政府等有关公共机构应当按照需求导向、分类分级、安全可控、高效便捷的原则，制定并公布年度公共数据开放清单或者计划，采取无条件开放、有条件开放等方式向社会开放公共数据。单位和个人可以通过公共数据开放平台获取公共数据。

　　鼓励单位和个人依法开放非公共数据，促进数据融合创新。

　　第十九条　本市设立金融、医疗、交通、空间等领域的公共数据专区，推动公共数据有条件开放和社会化应用。市人民政府可以开展公共数据专区授权运营。

　　市人民政府及其有关部门可以探索设立公共数据特定区域，建立适应数字经济特征的新型监管方式。

　　市经济和信息化部门推动建设公共数据开放创新基地以及大数据相关的实验室、研究中心、技术中心等，对符合条件的单位和个人提供可信环境和特定数据，促进数据融合创新应用。

　　第二十条　除法律、行政法规另有规定或者当事人另有约定外，单位和个人对其合法正当收集的数据，可以依法存储、持有、使用、加工、传输、提供、公开、删除等，所形成的数据产品和数据服务的相关权益受法律保护。

　　除法律、行政法规另有规定外，在确保安全的前提下，单位和个人可以对城市基础设施、建筑物、构筑物、物品等进行数字化仿真，并对所形成的数字化产品持有相关权益，但需经相关权利人和有关部门同意的，应当经其同意。

　　第二十一条　支持市场主体探索数据资产定价机制，推动形成数据资产目录，激发企业在数字经济领域投资动力；推进建立数据资产登记和评估机制，支持开展数据入股、数据信贷、数据信托和数据资产证券化等数字经济业态创新；培育数据交易撮合、评估评价、托管运营、合规审计、争议仲裁、法律服务等数据服务市场。

　　第二十二条　支持在依法设立的数据交易机构开展数据交易活动。数据交易机构应当制定数据交易规则，对数据提供方的数据来源、交易双方的身份进行合规性审查，并留存审查和交易记录，建立交易异常行为风险预警机制，确保数据交易公平有序、安全可控、全程可追溯。

　　本市公共机构依托数据交易机构开展数据服务和数据产品交易活动。

　　鼓励市场主体通过数据交易机构入场交易。

第四章　数字产业化

　　第二十三条　市、区人民政府及其有关部门应当支持数字产业基础研究和关键核心技术攻关，引导企业、高校、科研院所、新型研发机构、开源社区等，围绕前沿领域，提升基础软硬件、核心元器件、关键基础材料和生产装备的供给水平，重点培育高端芯片、新型显示、基础软件、工业软件、人工智能、区块链、大数据、云计算等数字经济核心产业。支持企业发展数字产业，培育多层次的企业梯队。

　　第二十四条　支持建设开源社区、开源平台和开源项目等，鼓励软件、硬件的开放创新发展，推动创新资源共建共享。

　　第二十五条　支持网络安全、数据安全、算法安全技术和软硬件产品的研发应用，鼓励安全咨询设计、安全评估、数据资产保护、存储加密、隐私计算、检测认证、监测预警、应急处置等数据安全服务业发展；支持相关专业机构依法提供服务；鼓励公共机构等单位提高数据安全投入水平。

　　第二十六条　支持平台企业规范健康发展，鼓励利用互联网优势，加大创新研发投入，加强平台企业间、平台企业与中小企业间的合作共享，优化平台发展生态，促进数字技术与实体经济融合发展，赋能经济社会转型升级。

　　发展改革、市场监管、网信、经济和信息化等部门应当优化平台经济发展环境，促进平台企业开放生态系统，通过项目合作等方式推动政企数据交互共享。

　　第二十七条　鼓励数字经济业态创新，支持远程办公等在线服务和产品的优化升级；有序引导新个体经济，鼓励个人利用电子商务、社交软件、知识分享、音视频网站、创客等新型平台就业创业。

　　支持开展自动驾驶全场景运营试验示范，培育推广智能网联汽车、智能公交、无人配送机器人、智能停车、智能车辆维护等新业态。

　　支持互联网医院发展，鼓励提供在线问诊、远程会诊、机器人手术、智慧药房等新型医疗服务，规范推广利用智能康养设备的新型健康服务，创新对人工智能新型医疗方式和医疗器械的监管方式。

　　支持数据支撑的研发和知识生产产业发展，积极探索基于大数据和人工智能应用的跨学科知识创新和知识生产新模式，以数据驱动产、学、研、用融合。

　　第二十八条　支持建设数字经济产业园区和创新基地，推动重点领域数字产业发展，推动数字产业向园区聚集，培育数字产业集群。

　　第二十九条　商务部门应当会同有关部门推动数字贸易高质量发展，探索放宽数字经济新业态准入、建设数字口岸、国际信息产业和数字贸易港；支持发展跨境贸易、跨境物流和跨境支付，促进数字证书和电子签名国际互认，构建国际互联网数据专用通道、国际化数据信息专用通道和基于区块链等先进技术的应用支撑平台，推动数字贸易交付、结算便利化。

第五章　产业数字化

　　第三十条　支持农业、制造业、建筑、能源、金融、医疗、教育、流通等产业领域互联网发展，推进产业数字化转型升级，支持产业互联网平台整合产业资源，提供远程协作、在线设计、线上营销、供应链金融等创新服务，建立健全安全保障体系和产业生态。

　　第三十一条　经济和信息化部门应当会同国有资产监管机构鼓励国有企业整合内部信息系统，在研发设计、生产加工、经营管理、销售服务等方面形成数据驱动的决策能力，提升企业运行和产业链协同效率，树立全面数字化转型的行业标杆。

　　经济和信息化部门应当推动中小企业数字化转型，培育发展第三方专业服务机构，鼓励互联网平台、龙头企业开放数据资源、提升平台能力，支持中小微企业和创业者创新创业，推动建立市场化服务与公共服务双轮驱动的数字化转型服务生态。

　　第三十二条　经济和信息化部门应当会同通信管理部门健全工业互联网标识解析体系和新型工业网络部署，支持工业企业实施数字化改造，加快建设智能工厂、智能车间，培育推广智能化生产、网络化协同、个性化定制等新模式。

　　第三十三条　地方金融监管部门应当推动数字金融体系建设，支持金融机构加快数字化转型，以数据融合应用推动普惠金融发展，促进数字技术在支付清算、登记托管、征信评级、跨境结算等环节的深度应用，丰富数字人民币的应用试点场景和产业生态。鼓励单位和个人使用数字人民币。

　　第三十四条　商务部门应当会同有关部门推动超市等传统商业数字化升级，推动传统品牌、老字号数字化推广，促进生活性服务业数字化转型。

　　第三十五条　农业农村部门应当会同有关部门推动农业农村基础设施数字化改造和信息网络基础设施建设，推进物联网、遥感监测、区块链、人工智能等技术的深度应用，提升农产品生产、加工、销售、物流，以及乡村公共服务、乡村治理的数字化水平，促进数字乡村和智慧农业创新发展。

　　第三十六条　教育、文化和旅游、体育、广播电视等部门应当支持和规范在线教育、在线旅游、网络出版、融媒体、数字动漫等数字消费新模式；发展数字化文化消费新场景；加强未成年人网络保护；鼓励开发智慧博物馆、智慧体育场馆、智慧科技馆，提升数字生活品质。

第六章　智慧城市建设

　　第三十七条　市、区人民政府及其有关部门围绕优政、惠民、兴业、安全的智慧城市目标，聚焦交通体系、生态环保、空间治理、执法司法、人文环境、商务服务、终身教育、医疗健康等智慧城市应用领域，推进城市码、空间图、基础工具库、算力设施、感知体系、通信网络、政务云、大数据平台以及智慧终端等智慧城市基础建设。

　　市人民政府建立健全智慧城市建设统筹调度机制，统筹规划和推进社会治理数字化转型，建立智慧城市规划体系，通过统一的基础设施、智慧终端和共性业务支撑平台，实现城市各系统间信息资源共享和业务协同，提升城市管理和服务的智慧化水平。

　　第三十八条　市经济和信息化部门应当会同有关部门编制全市智慧城市发展规划、市级控制性规划，报市人民政府批准后组织实施。区人民政府、市人民政府有关部门应当按照全市智慧城市发展规划、市级控制性规划，编制区域控制性规划、专项规划并组织实施。

　　第三十九条　政务服务部门应当会同有关部门全方位、系统性、高标准推进数字政务“一网通办”领域相关工作，加快推进政务服务标准化、规范化、便利化，推进线上服务统一入口和全程数字化，促进电子证照、电子印章、电子档案等广泛应用和互信互认。

　　市发展改革部门应当会同有关部门开展营商环境的监测分析、综合管理、“互联网＋”评价，建设整体联动的营商环境体系。

　　第四十条　城市管理部门应当会同有关部门推进城市运行“一网统管”领域相关工作，建设城市运行管理平台，依托物联网、区块链等技术，开展城市运行生命体征监测，在市政管理、城市交通、生态环境、公共卫生、社会安全、应急管理等领域深化数字技术应用，实现重大突发事件的快速响应和应急联动。

　　市场监管部门应当会同有关部门推进一体化综合监管工作，充分利用公共数据和各领域监管系统，推行非现场执法、信用监管、风险预警等新型监管模式，提升监管水平。

　　第四十一条　经济和信息化部门应当会同有关部门推进各级决策“一网慧治”相关工作，建设智慧决策应用统一平台，支撑各级智能决策管理信息系统，统筹引导市、区、乡镇、街道和社区、村开展数据智慧化应用。

　　区人民政府和有关部门依托智慧决策应用统一平台推进各级决策，深化数据赋能基层治理。

　　第四十二条　公共机构应当通过多种形式的场景开放，引导各类市场主体参与智慧城市建设，并为新技术、新产品、新服务提供测试验证、应用试点和产业孵化的条件。市科技部门应当会同有关部门定期发布应用场景开放清单。

　　鼓励事业单位、国有企业开放应用场景，采用市场化方式，提升自身数字化治理能力和应用水平。

　　第四十三条　政府投资新建、改建、扩建、运行维护的信息化项目，应当符合智慧城市发展规划，通过同级经济和信息化部门的技术评审，并实行项目规划、建设、验收、投入使用、运行维护、升级、绩效评价等流程管理。不符合流程管理要求的，不予立项或者安排资金，具体办法由市经济和信息化部门会同有关部门制定，报市人民政府批准后实施。

　　为公共机构提供信息化项目开发建设服务的单位，应当依法依约移交软件源代码、数据和相关控制措施，保证项目质量并履行不少于两年保修期义务，不得擅自留存、使用、泄露或者向他人提供公共数据。

第七章　数字经济安全

　　第四十四条　市、区人民政府及其有关部门和有关组织应当强化数字经济安全风险综合研判，推动关键产品多元化供给，提高产业链供应链韧性；引导社会资本投向原创性、引领性创新领域，支持可持续发展的业态和模式创新；规范数字金融有序创新，严防衍生业务风险。

　　第四十五条　本市依法保护与数据有关的权益。任何单位和个人从事数据处理活动，应当遵守法律法规、公序良俗和科技伦理，不得危害国家安全、公共利益以及他人的合法权益。

　　任何单位和个人不得非法处理他人个人信息。

　　第四十六条　市、区人民政府及其有关部门应当建立健全数据安全工作协调机制，采取数据分类分级、安全风险评估和安全保障措施，强化监测预警和应急处置，切实维护国家主权、安全和发展利益，提升本市数据安全保护水平，保护个人信息权益。各行业主管部门、各区人民政府对本行业、本地区数据安全负指导监督责任。单位主要负责人为本单位数据安全第一责任人。

　　第四十七条　市网信部门会同公安等部门对关键信息基础设施实行重点保护，建立关键信息基础设施网络安全保障体系，构建跨领域、跨部门、政企合作的安全风险联防联控机制，采取措施监测、防御、处置网络安全风险和威胁，保护关键信息基础设施免受攻击、侵入、干扰和破坏，依法惩治危害关键信息基础设施安全的违法犯罪活动。

　　第四十八条　开展数据处理活动，应当建立数据治理和合规运营制度，履行数据安全保护义务，严格落实个人信息合法使用、数据安全使用承诺和重要数据出境安全管理等相关制度，结合应用场景对匿名化、去标识化技术进行安全评估，并采取必要技术措施加强个人信息安全保护，防止非法滥用。鼓励各单位设立首席数据官。

　　开展数据处理活动，应当加强风险监测，发现数据安全缺陷、漏洞等风险时，应当立即采取补救措施；发生数据安全事件时，应当立即采取处置措施，按照规定及时告知用户并向有关主管部门报告。

　　第四十九条　平台企业应当建立健全平台管理制度规则；不得利用数据、算法、流量、市场、资本优势，排除或者限制其他平台和应用独立运行，不得损害中小企业合法权益，不得对消费者实施不公平的差别待遇和选择限制。

　　发展改革、市场监管、网信等部门应当建立健全平台经济治理规则和监管方式，依法查处垄断和不正当竞争行为，保障平台从业人员、中小企业和消费者合法权益。

第八章　保障措施

　　第五十条　本市建立完善政府、企业、行业组织和社会公众多方参与、有效协同的数字经济治理新格局，以及协调统一的数字经济治理框架和规则体系，推动健全跨部门、跨地区的协同监管机制。

　　数字经济相关协会、商会、联盟等应当加强行业自律，建立健全行业服务标准和便捷、高效、友好的争议解决机制、渠道。

　　鼓励平台企业建立争议在线解决机制和渠道，制定并公示争议解决规则。

　　第五十一条　网信、教育、人力资源和社会保障、人才工作等部门应当组织实施全民数字素养与技能提升计划。畅通国内外数字经济人才引进绿色通道，并在住房、子女教育、医疗服务、职称评定等方面提供支持。

　　鼓励高校、职业院校、中小学校开设多层次、多方向、多形式的数字经济课程教学和培训。

　　支持企业与院校通过联合办学，共建产教融合基地、实验室、实训基地等形式，拓展多元化人才培养模式，培养各类专业化和复合型数字技术、技能和管理人才。

　　第五十二条　财政、发展改革、科技、经济和信息化等部门应当统筹运用财政资金和各类产业基金，加大对数字经济关键核心技术研发、重大创新载体平台建设、应用示范和产业化发展等方面的资金支持力度，引导和支持天使投资、风险投资等社会力量加大资金投入，鼓励金融机构开展数字经济领域的产品和服务创新。

　　政府采购的采购人经依法批准，可以通过非公开招标方式，采购达到公开招标限额标准的首台（套）装备、首批次产品、首版次软件，支持数字技术产品和服务的应用推广。

　　第五十三条　知识产权等部门应当执行数据知识产权保护规则，开展数据知识产权保护工作，建立知识产权专利导航制度，支持在数字经济行业领域组建产业知识产权联盟；加强企业海外知识产权布局指导，建立健全海外预警和纠纷应对机制，建立快速审查、快速维权体系，依法打击侵权行为。

　　第五十四条　政务服务、卫生健康、民政、经济和信息化等部门应当采取措施，鼓励为老年人、残疾人等提供便利适用的智能化产品和服务，推进数字无障碍建设。对使用数字公共服务确有困难的人群，应当提供可替代的服务和产品。

　　第五十五条　市、区人民政府及其有关部门应当加强数字经济领域相关法律法规、政策和知识的宣传普及，办好政府网站国内版、国际版，深化数字经济理论和实践研究，营造促进数字经济的良好氛围。

　　第五十六条　鼓励拓展数字经济领域国际合作，支持参与制定国际规则、标准和协议，搭建国际会展、论坛、商贸、赛事、培训等合作平台，在数据跨境流动、数字服务市场开放、数字产品安全认证等领域实现互惠互利、合作共赢。

　　第五十七条　鼓励政府及其有关部门结合实际情况，在法治框架内积极探索数字经济促进措施；对探索中出现失误或者偏差，符合规定条件的，可以予以免除或者从轻、减轻责任。

第九章　附　　则

第五十八条　本条例自2023年1月1日起施行。

(7)江苏省数字经济促进条例

2022年5月31日江苏省第十三届人民代表大会常务委员会第三十次会议通过

目 录

　　第一章　总则

　　第二章　数字技术创新

　　第三章　数字基础设施建设

　　第四章　数字产业化

　　第五章　产业数字化

　　第六章　治理和服务数字化

　　第七章　数据利用和保护

　　第八章　保障和监督

　　第九章　附则

第一章　总则

　　第一条　为了推动数字经济与实体经济深度融合，推进数据要素依法有序流动，保障数据安全，建设数字经济强省，促进经济高质量发展，根据有关法律、行政法规，结合本省实际，制定本条例。

　　第二条　本省行政区域内促进数字经济发展，以及为数字经济提供支撑保障等相关活动，适用本条例。

　　本条例所称数字经济，是指以数据资源为关键要素，以现代信息网络为主要载体，以信息通信技术融合应用、全要素数字化转型为重要推动力，促进公平与效率更加统一的新经济形态。

　　第三条　数字经济发展应当遵循创新引领、融合发展，应用牵引、数据赋能，公平竞争、安全有序，系统推进、协同高效的原则。

　　第四条　省人民政府应当加强对数字经济发展的领导，统筹部署、组织推进全省数字经济发展。

　　县级以上地方人民政府应当将数字经济发展纳入国民经济和社会发展规划，并根据需要制定本地区数字经济发展规划，支持开展数字技术创新和应用，培育和发展新产业新业态新模式，加快建设与数字经济发展相适应的产业生态体系、公共服务体系和现代治理体系，营造优良的数字经济发展环境。

　　县级以上地方人民政府应当建立数字经济推进协调机制，完善数字经济发展政策，协调解决数字经济发展中的重大问题。省发展改革部门和设区的市、县级人民政府确定的数字经济主管部门承担协调机制日常工作。

　　第五条　省发展改革部门负责拟定促进全省数字经济发展战略、规划和重大政策，推进实施数字化发展重大工程和项目。

　　省工业和信息化部门负责制定并实施数字经济发展相关专项规划和政策措施，推进数字产业化发展、工业数字化转型、数字技术应用和信息基础设施建设。

　　省科技部门负责指导、协调数字经济创新平台建设，推动数字技术基础研究、关键核心技术攻关和科技成果转化。

　　省网信部门负责协调推动公共服务和社会治理信息化，统筹协调网络安全、网络数据安全、个人信息保护和相关监督管理工作。

　　省政务服务管理部门负责公共数据管理，组织协调公共数据归集、共享、开放。

　　省通信部门负责协调推进信息通信基础设施建设和应用。

　　省其他有关部门在各自职责范围内做好促进数字经济发展相关工作。

　　设区的市、县级人民政府确定的数字经济主管部门负责推进本地区数字经济发展具体工作。

　　第六条　省人民政府以及有关部门应当在参与“一带一路”建设等对外合作中加强数字经济领域对外交流合作，构建数字经济开放体系。鼓励和支持中国（江苏）自由贸易试验区探索数据跨境安全有序流动。

　　省人民政府以及有关部门应当按照长三角区域一体化发展、长江经济带发展等国家战略要求，加强跨省域合作，推动重大数字基础设施共建共享、数据标准统一、数据资源共享开放、智能制造协同发展以及区域一体化协同治理应用。

　　县级以上地方人民政府应当加强省内外数字经济跨区域合作，创新体制机制，加强政策协调，共同促进数字经济发展。

　　第七条　鼓励和支持各类市场主体参与数字基础设施投资建设、数字产业化发展、产业数字化转型、治理和服务数字化以及数据开发利用。

第二章　数字技术创新

　　第八条　省人民政府以及有关部门应当推动数字技术创新，加强数字技术基础研究、应用研究和技术成果转化，完善产业技术创新体系和共性基础技术供给体系。

　　省人民政府以及有关部门应当建立数字经济关键核心技术攻关新型体制机制，支持企业、高等学校、科研机构聚焦传感器、量子信息、网络通信、集成电路、人工智能、区块链等重点领域，提高数字技术基础研发能力，突破高端芯片、工业软件、核心算法等关键核心技术。

　　第九条　省人民政府以及有关部门应当围绕云计算、大数据、物联网、新一代移动通信、人工智能、区块链等领域，推动建设国家和省级实验室、产业创新中心、制造业创新中心、技术创新中心等创新平台。

　　第十条　省人民政府以及有关部门应当统筹协调数字经济产业链整体发展，推进产业链核心企业带动上下游企业协同创新，提升产业链创新水平。

　　第十一条　县级以上地方人民政府以及有关部门应当推动数字技术融合创新，强化企业创新主体地位，发挥企业在数字技术创新中的重要作用，支持各类市场主体平等获取数字技术创新资源。

　　引导企业与高等学校、科研机构开展数字经济产学研合作，共建技术创新联盟、科技创新基地、博士后科研工作站等创新平台，推动获取重大原创科技成果和自主知识产权。

　　第十二条　县级以上地方人民政府以及市场监督管理、知识产权、版权等部门应当加强数字经济领域知识产权保护，推动知识产权转化运用，建立快速维权体系，依法打击知识产权侵权行为。

　　第十三条　科技等部门应当支持数字经济产业领域科技创新，可以通过专项资金支持科技成果转化，采用发放科技创新券等方式购买检验检测、研发设计、中间试验、科技评估、技术查新、知识产权、技术培训等服务。

　　第十四条　支持数字技术创新产品和服务的应用推广，将符合条件的数字技术产品和服务认定为首台（套）装备、首批次新材料、首版次软件，列入创新产品目录。

　　省人民政府或者其授权的单位可以根据需要，将数字技术产品和服务列入全省集中采购目录。确因数字技术产品和服务应用推广需要，政府采购达到公开招标限额标准的首台（套）装备、首批次新材料、首版次软件的，经依法批准，可以通过非公开招标方式进行采购。

第三章　数字基础设施建设

　　第十五条　县级以上地方人民政府应当支持完善通信网络、算力等信息基础设施，建设物联网、车联网等融合基础设施，布局创新基础设施，推动传统基础设施数字化升级，构建数字基础设施体系。

　　第十六条　省工业和信息化、通信部门应当会同省发展改革部门根据省数字经济发展规划，编制省数字基础设施发展规划，报省人民政府批准后实施。

　　设区的市应当根据省数字基础设施发展规划，编制、实施本地区数字基础设施发展规划和数字基础设施建设专项规划。有条件的县（市）可以编制、实施本地区数字基础设施发展规划和数字基础设施建设专项规划。

　　编制、实施数字基础设施发展规划和数字基础设施建设专项规划应当遵循适度超前、合理布局、共建共享、互联互通的原则，重点推进高速泛在、天地一体、云网融合、智能敏捷、绿色低碳、安全可控的数字基础设施建设。

　　第十七条　县级以上地方人民政府编制国土空间规划时应当统筹考虑数字基础设施的空间布局安排。交通、电力、市政、公共安全等相关基础设施规划应当结合数字经济发展需要，与数字基础设施相关规划相互协调和衔接。

　　第十八条　县级以上地方人民政府以及有关部门应当加快通信网络基础设施建设，支持新一代移动通信网络和高速固定宽带网络部署，推进城乡信息通信网络服务能力一体化，提升网络性能和服务能力。

　　新建、扩建建设工程，建设单位应当根据规划，按照国家和省有关标准预留基站站址，配套建设通信网络基础设施，与主体工程同步设计、同步施工、同步验收。推动老旧小区改造配套建设通信网络基础设施。

　　第十九条　公共机构以及公共场所、公共设施的所有者、管理者或者使用者应当支持通信网络基础设施建设，按照国家和省有关规定开放建筑物、绿地、杆塔等资源，推进智慧杆塔建设和一杆多用。

　　推动通信网络基础设施与铁路、城市轨道、道路、桥梁、隧道、电力、地下综合管廊、机场、港口、枢纽场站等基础设施以及相关配套设施共商共建共享共维。

　　第二十条　省自然资源部门应当统筹本省卫星导航定位基准服务系统和配套基础设施建设，提供卫星导航定位基准信息公共服务。

　　鼓励符合法定条件的组织参与卫星互联网基础设施建设，构建通信、导航、遥感空间基础设施体系。

　　第二十一条　省人民政府以及有关部门应当推进算力基础设施建设，统筹全省数据中心合理布局，推动智能计算中心、边缘数据中心等新型数据中心建设，支持互联网、工业、金融、政务等领域数据中心规模化发展，提升计算能力，强化算力统筹和智能调度。

　　省、设区的市人民政府以及有关部门应当推进数据中心向集约高效、绿色低碳方向发展，推动已建数据中心节能改造。支持数据中心集群配套可再生能源电站，鼓励数据中心参与可再生能源市场交易；支持数据中心采用大用户直供、建设分布式光伏等方式提升可再生能源电力消费。

　　第二十二条　鼓励有条件的地区建设泛在互联、智能感知的物联网，推进基础设施、城乡治理、物流仓储、生产制造、生活服务、生态环境保护、应急管理等领域感知系统的建设应用、互联互通和数据共享。

　　第二十三条　县级以上地方人民政府以及交通运输等部门应当推动发展智能交通，加速交通基础设施网、运输服务网、能源网与信息网络融合发展。

　　县级以上地方人民政府以及工业和信息化等部门应当加快国家级和省级车联网先导区建设，扩大车联网覆盖范围，提高路侧单元与道路基础设施、智能管控设施的融合接入能力，推进道路基础设施、交通标志标识的数字化改造和建设。

　　第二十四条　省人民政府以及有关部门应当在数字经济重点方向布局未来网络试验设施等创新基础设施。

　　支持发展开源社区、开源代码托管平台，建设人工智能技术应用平台、自主安全可控的区块链底层平台和重点领域大数据训练平台等。

　　第二十五条　县级以上地方人民政府应当统筹推进能源、城乡建设、物流、教育、健康、文化、旅游、体育、自然资源、水利、生态环境保护、应急管理等领域的传统基础设施数字化、智能化改造，建立健全跨行业基础设施协同推进机制。

　　第二十六条　数字基础设施依法受到保护。任何组织、个人不得侵占或者擅自迁移、拆除数字基础设施，不得实施非法侵入、干扰、破坏数字基础设施的活动，不得危害数字基础设施安全。

　　确因公共利益或者其他法定事由需要迁移、拆除数字基础设施的，应当依法给予补偿。

第四章　数字产业化

　　第二十七条　省人民政府应当根据全球数字经济的技术、产业发展趋势，结合本省数字产业发展水平和各地区禀赋差异，统筹规划全省数字产业发展，围绕数字经济核心产业，通过推进产业链强链补链、保障供应链安全、培育产业集群等方式，构建优势产业链，促进产业协同和供应保障，提高数字产业整体竞争力。

　　第二十八条　县级以上地方人民政府以及有关部门应当按照全省数字产业发展要求，结合本地区实际，通过规划引导、政策支持等方式，在集成电路、物联网、工业机器人、新型显示、智能终端、光电缆、通信设备、核心电子元器件以及设备制造等特色优势领域，加快重大项目推进、产业链上下游对接配套、骨干龙头企业培育，打造具有国际竞争力的产业高地。

　　第二十九条　省人民政府以及工业和信息化、发展改革等部门应当统筹规划软件产业发展，支持基础软件、工业软件核心技术自主创新和开源软件发展，提升自主可控关键软件和创新应用软件供给能力。

　　县级以上地方人民政府以及有关部门应当根据全省统一布局，结合本地区产业基础和特点，推动软件产业集群建设，培育软件名城和软件名园，构建安全可控、开放协同的现代软件产业体系。

　　第三十条　县级以上地方人民政府以及有关部门应当结合本地区实际，培育云计算、大数据、区块链、人工智能等新兴数字产业，促进跨界融合和集成创新；前瞻布局类脑智能、量子信息、基因技术、宽禁带半导体、下一代移动通信等未来产业。

　　第三十一条　鼓励企业平台化发展，在工业互联网、网络销售服务、物流专业服务、信息资讯服务、检验检测服务等重点领域，支持和培育平台经济重点企业。

　　县级以上地方人民政府以及有关部门应当引导和支持平台企业加强数据、产品、内容等资源整合共享，探索适宜本地区的平台经济发展场景和模式，推进平台经济领域互联互通；按照国家规定明确平台企业定位和监管规则，促进平台经济健康发展。

　　第三十二条　县级以上地方人民政府以及有关部门应当推进文化产业线上线下融合，推动文化遗产资源的数字化转化，发展网络视听、数字影视、数字广告、互动新媒体等数字文化产业，引导沉浸式体验、动漫游戏、数字演艺等健康发展。

　　第三十三条　县级以上地方人民政府以及发展改革、科技、工业和信息化、商务、市场监督管理等部门应当采取措施，引导和支持数字经济核心产业龙头企业、高新技术企业，以及科技型中小企业和专精特新中小企业发展，培育多层次、递进式的数字产业企业梯队，形成大中小企业相互协同、优势互补的发展格局。

　　第三十四条　县级以上地方人民政府以及有关部门应当结合本地区实际，推进数字经济相关产业园区优化发展，培育建设特色数字产业创新、数字技术应用创新等载体。

　　第三十五条　引导互联网企业、行业龙头企业、基础电信企业开放数据资源和平台计算能力等，支持企业、高等学校、科研机构以及其他组织、个人创建数字经济领域科技企业孵化器、大学科技园和众创空间等线上线下创新创业平台。

　　鼓励第三方专业化服务机构为数字产业相关企业引进落地、融资增资、股改上市、平台化转型、并购和合作等提供服务。

第五章　产业数字化

　　第三十六条　县级以上地方人民政府应当促进数字经济与实体经济深度融合，利用数字技术促进产业改造升级，催生新产业新业态新模式，推动制造业、服务业、农业等产业数字化。

　　第三十七条　县级以上地方人民政府以及有关部门应当推动企业实施制造装备、生产线、车间、工厂等的智能化改造，提升企业研发设计、生产制造、经营管理、销售服务等环节的智能化水平，推广网络化协同、个性化定制、柔性化生产、共享制造等服务型制造新模式，实现工业生产模式变革。

　　第三十八条　县级以上地方人民政府以及有关部门应当支持数字技术在先进制造业集群的深度应用，鼓励产业链龙头企业打造供应链数字化协作平台，实现产业链上下游的供需数据对接和协同生产。支持工业骨干企业、工业数字化转型设备供应商和服务商等组建数字化转型联盟，分行业研发推广数字化解决方案，促进集群企业协同转型。

　　第三十九条　县级以上地方人民政府以及有关部门应当加大对工业互联网发展的支持力度，推进企业级、区域级、行业级等工业互联网平台建设和应用，培育国家级跨行业跨领域工业互联网平台，提升工业信息安全保障能力；支持企业基础设施、业务、设备和数据上云、上平台，推动运用高适配、快部署、易运维的工业互联网解决方案，降低中小企业的工业互联网使用成本，普及应用工业互联网。

　　第四十条　县级以上地方人民政府以及工业和信息化等部门应当推动纺织、冶金、化工、建材、轻工、机械、医药、电子等传统优势制造业的数字化转型，鼓励工业生产方式和组织模式创新，提高全要素生产率。

　　第四十一条　支持装备制造企业研制高端数控机床、工业机器人等数字化装备，加强新型传感器、智能测量仪表、工业控制系统、网络通信模块等智能核心装置的集成应用，提升智能装备供给能力。

　　支持软件企业、智能装备制造企业围绕工业企业数字化转型需求，开展工业基础软件、工业控制软件、数据管理软件、系统解决方案的联合攻关，加强工业软件支撑能力建设。

　　第四十二条　县级以上地方人民政府以及有关部门应当推进研发设计、现代物流、检验检测、商务咨询、人力资源服务等生产性服务业数字化，提升生产性服务业智能化、网络化、专业化水平。

　　县级以上地方人民政府以及有关部门应当推动数字技术与健康、养老、旅游、体育、文化、居民出行、住宿餐饮、教育培训等生活性服务业深度融合，发展体验式消费、个性需求定制服务等新业态，丰富数字服务产品供给。

　　第四十三条　县级以上地方人民政府以及有关部门应当在风险可控前提下推动发展数字金融，优化移动支付应用，按照国家有关规定推行数字人民币应用，推进数字金融与产业链、供应链融合。

　　第四十四条　县级以上地方人民政府以及有关部门应当制定相关政策，完善发展机制、监管模式，引导和支持电子商务发展，促进跨境电商综合试验区建设，支持数字化商贸平台建设，加快数字贸易发展，推广新零售，发展社交电商、直播电商等新业态新模式。

　　第四十五条　县级以上地方人民政府以及农业农村等部门应当推进农业数字化，加快种植业、畜牧业、渔业、种业、农产品加工业等领域数字技术应用，推广应用智能农机装备，加强农业农村大数据建设，强化益农信息服务，加大农村仓储、物流、冷链设施建设支持力度，提升农业生产、加工、销售、物流等各环节数字化水平。

　　支持新型农业经营主体、加工流通企业与电商企业对接融合，发展直采直供、冷链配送、社区拼购等农产品销售服务新业态新模式。

　　第四十六条　县级以上地方人民政府以及有关部门应当建立公共服务与市场化服务相结合，技术、资本、人才、数据等多要素支撑的数字化转型服务生态。

　　鼓励和支持企业、高等学校、科研机构、学会、协会、商会等围绕产业数字化转型，提供诊断咨询、应用培训、测试评估等服务。

　　支持建立公共型数字化转型促进中心，鼓励行业龙头企业建立开放型数字化转型促进中心，重点面向中小企业提供数字化转型诊断和低成本、轻量化、模块化的数字化解决方案。

第六章　治理和服务数字化

　　第四十七条　县级以上地方人民政府应当推动数字技术在政府治理中的创新应用，推进政府治理数字化，发挥数字化在政府履行经济调节、市场监管、社会管理、公共服务、生态环境保护、突发事件应对等方面职能的支撑作用，构建协同高效的政府数字化履职能力体系，提高事前预防、事中监管和事后处置能力。

　　第四十八条　县级以上地方人民政府以及有关部门应当推动非现场监管、移动电子执法和风险预警模型等现代化管理应用场景建设；全面推动政务服务一件事、社会治理一类事、政务运行一体事三大领域清单改革，加快实现一网通办，做好不见面审批服务。

　　县级以上地方人民政府以及有关部门应当推广电子签名、电子印章、电子证照、电子档案的应用。除法律、行政法规另有规定外，电子证照以及加盖电子印章的电子材料，可以作为办理政务服务事项的依据。

　　第四十九条　县级以上地方人民政府应当加强智慧城市建设，促进数字技术在城市治理中的应用，通过数据资源整合共享和开放，实现城市运行态势监测、公共资源配置、宏观决策、统一指挥调度和事件分拨处置的数字化，提升城市治理水平。

　　县级以上地方人民政府应当开展智慧社区建设，以数字技术强化社区服务和管理功能综合集成，推动政务服务、公共服务、数字商务向社区延伸，提升精细化、网格化管理能力，构建居家养老、儿童关爱、文体活动、家政服务、社区电商等数字化创新应用场景。

　　第五十条　县级以上地方人民政府应当推进数字乡村建设，加快基本公共服务向乡镇、村居延伸，实行涉农服务事项线上线下一体化办理，推动数字技术与农业农村基础设施融合发展，促进数字技术在乡村产业发展、农村集体资产管理等领域的综合应用，提升乡村治理数字化水平。

　　第五十一条　县级以上地方人民政府应当加强数字技术在园区的融合应用，提升园区公共服务、物业管理、产业集聚、人才服务、创新协同等方面的智慧化服务水平，支撑园区内企业数字化转型和数字产业集聚发展。

　　第五十二条　县级以上地方人民政府以及教育部门应当按照有关规定加强智慧校园建设，完善教育资源和信息管理公共服务平台，打造个性化、终身化的教育信息化公共服务体系，推进线上线下教育常态化融合发展新模式。

　　第五十三条　县级以上地方人民政府以及卫生健康、医疗保障等部门应当加强智慧医疗健康体系建设，推进全省统一医疗保障信息平台落地应用，统筹全民健康信息平台、医院信息平台建设和信息互联互通，强化医疗健康大数据开发应用，发展互联网医疗，促进大健康产业发展。

　　第五十四条　省人民政府以及人力资源社会保障部门应当加强智慧人社体系建设，依托全省人社一体化信息平台推进就业创业、社会保障、人才人事、劳动关系等领域数字化转型，与有关部门开展协同合作，以社会保障卡为载体，在居民服务领域逐步实现多卡合一、一卡通用。

　　第五十五条　县级以上地方人民政府以及民政、工业和信息化等部门应当加强智慧养老体系建设，推进移动终端、可穿戴设备、服务机器人等智能设备在居家、社区、机构等养老场景集成应用，推广智慧养老服务平台，提供简便快捷的养老政务服务、公共服务、公益服务和链接市场服务。

　　第五十六条　县级以上地方人民政府以及有关部门应当按照优化传统服务与创新数字服务并行的原则，针对老年人等运用智能技术困难群体，制定完善相关措施，保障和改善其基本服务需求和服务体验。

第七章　数据利用和保护

　　第五十七条　县级以上地方人民政府以及有关部门应当遵循促进流通、合理使用、依法规范、保障安全的原则，发挥数据的基础资源作用和创新引擎作用，加强数据资源全生命周期管理，促进数据资源开发利用和健康发展。

　　第五十八条　县级以上地方人民政府以及有关部门应当统筹推进国家机关、法律法规授权的具有管理公共事务职能的组织、公共企业事业单位为履行法定职责、提供公共服务收集、产生的公共数据资源汇集整合、共享开放和开发利用。

　　公共数据应当以共享为原则、不共享为例外。公共数据应当通过公共数据平台进行共享开放。

　　县级以上地方人民政府以及有关部门应当加大公共数据资源供给，统筹运用公共数据平台，释放公共数据价值，规范公共数据管理。具体办法由省人民政府制定。

　　第五十九条　县级以上地方人民政府以及有关部门应当统筹建立公共数据开放范围动态调整机制，创新公共数据资源开发利用模式和运营机制，满足组织、个人的合理需求。

　　县级以上地方人民政府以及有关部门应当推进公共数据创新应用，运用公共数据发展和完善数据要素市场，支持和推动公共数据资源开发利用，提升公共数据资源价值。

　　鼓励和支持组织、个人依法开发利用公共数据资源，提供数据产品和数据服务。

　　第六十条　县级以上地方人民政府以及有关部门应当通过产业政策引导、社会资本引入、应用模式创新、强化合作交流等方式，引导企业等组织、个人有序开放自有数据资源。

　　第六十一条　县级以上地方人民政府应当推动数据要素市场化建设，发展数据运营机构、数据经纪人，推进数据交易，规范数据交易行为，促进数据高效流通。有条件的地区可以依法设立数据交易场所，鼓励和引导数据供需双方在数据交易场所进行交易。

　　第六十二条　县级以上地方人民政府应当健全网络安全保障体系和数据安全治理体系，依法实施网络安全等级保护制度和数据安全审查制度，建立健全数据安全风险评估、报告、信息共享、监测预警、应急处置机制，推动建立政府监管、平台自治、行业自律、公众参与的多元共治体系。

　　第六十三条　各地区、各部门应当按照数据分类分级保护制度，对本地区、本部门以及相关行业、领域的数据开展分类分级管理，确定本地区、本部门以及相关行业、领域的重要数据具体目录，对列入目录的数据进行重点保护。

　　第六十四条　组织、个人与数据有关的权益依法受到保护。

　　数据的收集、存储、使用、加工、传输、提供、公开等处理活动，应当遵守法律、法规，尊重社会公德和伦理，遵守商业道德和职业道德，诚实守信，履行数据安全保护义务，承担社会责任。

　　开展数据处理活动，不得危害国家安全、公共利益，不得损害组织、个人的合法权益。

　　第六十五条　数据的处理者应当依法建立健全全流程数据安全管理制度，组织开展数据安全教育培训，采取相应的技术措施和其他必要措施，保障数据安全。

　　处理个人信息应当具有明确、合理的目的，遵循合法、正当、必要和诚信原则，依法制定、公开个人信息处理规则，明示处理的目的、方式和范围，保障所处理的个人信息的安全。禁止通过误导、欺诈、胁迫等方式处理个人信息。

　　县级以上地方人民政府有关部门依法履行个人信息保护和监督管理职责。

　　第六十六条　工业和信息化等部门应当组织企业开展数据管理国家标准实施工作，推动企业规范数据管理，提升数据质量。引导行业龙头企业参与制定行业数据国家标准并应用推广，提升行业数据标准化水平。

　　第六十七条　各地区、各部门应当推行首席数据官制度，由本地区、本部门相关负责人担任首席数据官。首席数据官应当协同管理本地区、本部门数据与业务工作，推动数据共享开放，建立与数字经济相关企业联系机制，提升本地区、本部门数据治理能力。

　　鼓励企业建立首席数据官制度，由企业相关负责人担任首席数据官，推动企业构建数据驱动的生产方式和管理模式。

　　第六十八条　支持社会化数据服务机构发展，依法依规开展公共数据、互联网数据、企业数据的采集、整理、聚合、分析等加工业务，提升数据资源处理能力，培育壮大数据服务产业。

第八章　保障和监督

　　第六十九条　省人民政府以及有关部门应当统筹使用省级专项资金，用于数字经济关键核心技术攻关、重大创新平台、公共技术平台和产业载体建设、应用示范和产业化发展等。

　　有条件的设区的市、县在本级财政预算中安排资金予以支持。

　　第七十条　省人民政府设立数字经济产业投资基金，用于数字经济领域重大项目建设和关键产业发展；鼓励有条件的地方设立数字经济股权基金，吸引社会资本支持数字经济发展。

　　县级以上地方人民政府应当完善投融资服务体系，拓宽数字经济市场主体融资渠道，发挥政策性基金作用，重点支持数字经济领域重大项目建设和高成长、初创型企业发展。

　　第七十一条　县级以上地方人民政府以及有关部门应当落实国家和省对高新技术企业研发、信息技术产品制造、软件开发、信息服务以及科技企业孵化器、大学科技园和众创空间等的优惠政策，并为相关组织、个人提供畅通的办理渠道。

　　第七十二条　鼓励和引导金融机构、地方金融组织对符合国家和省数字经济产业政策的项目、企业、平台和创新人才，在贷款、政策性融资担保以及其他金融服务等方面给予支持。鼓励银行业金融机构创新产品和服务，加大对数字经济核心产业的支持力度。

　　鼓励和支持数字经济创新型企业通过股权投资、股票债券发行等方式融资，提高直接融资比例，改善融资结构。

　　第七十三条　省人民政府以及有关部门应当推进数字经济标准体系建设，制定和实施关键核心技术、通用算法、数据治理和安全合规等领域的地方标准。

　　鼓励企业、高等学校、科研机构、行业协会等参与制定数字经济相关国际规则、国际标准、国家标准、行业标准和地方标准。支持依法制定数字经济相关企业标准、团体标准。

　　第七十四条　本省应当加强数字人才建设，将数字经济领域引进的高层次、高技能以及紧缺人才纳入人才支持政策范围，为其在职称评定、住房、落户、医疗以及配偶就业、子女入学等方面提供支持。探索建立适应数字经济新业态发展需要的人才评价机制。

　　鼓励企业事业单位、社会组织等培养创新型、应用型、技能型、融合型人才。推进数字经济相关学科建设，支持高等学校、中等职业学校、技工院校开设数字经济专业、课程，与企业合作办学，共建现代产业学院、联合实验室、实习基地等，培养数字经济相关人才。

　　第七十五条　县级以上地方人民政府以及人力资源社会保障等部门应当加强数字经济领域劳动用工服务指导，鼓励依托数字经济创造更多灵活就业机会，完善平台经济、共享经济等新业态从业人员在工作时间、报酬支付、保险保障等方面政策规定，保障数字经济新业态从业人员的合法权益。

　　第七十六条　县级以上地方人民政府应当在土地供应、电力接入、能耗指标分配、频谱资源配置等方面完善政策措施，强化创新服务，为促进数字经济发展提供保障。

　　第七十七条　县级以上地方人民政府有关部门应当加强数字经济相关技术知识、法律知识的宣传、教育、培训，提升全民数字素养和数字技能。

　　教育、人力资源社会保障等部门应当指导和督促学校以及其他教育机构将数字经济知识纳入教育教学内容。

　　公务员主管部门应当将数字经济知识纳入公务员教育培训内容。

　　广播、电视、报刊、互联网等新闻媒体应当开展数字经济公益性宣传。鼓励企业事业单位、社会组织等加强从业人员数字经济知识培训，提升应用、管理和服务水平。

　　第七十八条　县级以上地方人民政府以及有关部门应当支持举办数字经济领域的国内国际展览、赛事、论坛等活动，搭建数字经济展示、交易、交流、合作平台，推动建立供需对接渠道，加强数字经济相关企业、产品、服务宣传，提高企业市场开拓能力。

　　县级以上地方人民政府以及有关部门应当支持数字经济领域企业参加境内外展览展销等活动。

　　第七十九条　县级以上地方人民政府以及有关部门应当建立健全与数字经济发展相适应的监管体系，创新基于数字技术手段的数字经济监管模式，提高数字经济监管和治理水平，优化数字经济营商环境，完善数字经济治理体系。

　　县级以上地方人民政府以及有关部门应当建立数字经济统计监测机制，开展数字经济统计、分析，依法向社会公布。

　　第八十条　市场监督管理部门应当依法查处滥用市场支配地位、达成并实施垄断协议以及从事不正当竞争等违法行为，保障数字经济市场主体的合法权益，营造公平竞争市场环境。

　　第八十一条　县级以上地方人民政府以及有关部门应当依法组织对使用财政资金的数字经济项目进行审计监督，保障财政资金的使用效益。

　　第八十二条　县级以上地方人民政府以及有关部门应当创新监管理念和方式，建立数字经济创新创业容错机制，对数字经济领域的新技术新产业新业态新模式实行包容审慎监管，对在数字经济促进工作中出现失误错误的有关部门及其工作人员，符合国家和省规定条件的可以不作负面评价。

　　第八十三条　省、设区的市人民政府定期对本级数字经济发展情况进行评估，对下一级人民政府数字经济发展情况开展监督检查。数字经济发展情况评估可以委托第三方机构开展。评估情况向社会公布。

　　第八十四条　违反网络安全、数据安全、个人信息保护等法律、法规规定的，依法给予行政处罚；给他人造成损害的，依法承担民事责任；构成犯罪的，依法追究刑事责任。

　　县级以上地方人民政府、有关部门及其工作人员在数字经济促进工作中不依法履行职责的，依法给予处分；构成犯罪的，依法追究刑事责任。

第九章　附则

第八十五条　本条例自2022年8月1日起施行。

(8)河北省数字经济促进条例

2022年5月27日河北省第十三届人民代表大会常务委员会第三十次会议通过

目　　录

　　第一章　总则

　　第二章　数字基础设施建设

　　第三章　数据资源开发利用

　　第四章　数字产业化

　　第五章　产业数字化

　　第六章　数字化治理

　　第七章　京津冀数字经济协同发展

　　第八章　保障和监督

　　第九章　附则

第一章　总　则

　　第一条　为了促进数字经济健康发展，培育经济增长新动能，加快建立现代化经济体系，推动经济社会高质量发展，根据有关法律、行政法规，结合本省实际，制定本条例。

　　第二条　本省行政区域内促进数字经济发展相关活动，适用本条例。

　　本条例所称数字经济，是指以数据资源为关键要素，以现代信息网络为主要载体，以信息通信技术融合应用、全要素数字化转型为重要推动力，促进公平与效率更加统一的新经济形态。

　　第三条　发展数字经济应当遵循创新引领、融合发展，应用牵引、数据赋能，公平竞争、安全有序，系统推进、协同高效的原则。

　　第四条　县级以上人民政府应当把发展数字经济作为推动经济社会高质量发展的重大战略，加强对数字经济发展工作的领导，建立健全数字经济发展工作协调机制，研究解决数字经济发展工作中的重大问题，推动数字产业化、产业数字化、数字化治理等数字经济各方面高质量发展。

　　第五条　省人民政府发展改革部门负责统筹推进、协调、督促全省数字经济发展工作，工业和信息化主管部门负责统筹推进工业领域数字化、网络化、智能化等工作，科学技术主管部门负责推动数字经济发展科技创新工作。省网信部门负责统筹、协调网络安全、数据安全和信息化重大专项任务，协同推进数字经济发展。省通信管理部门负责推进全省信息通信网络布局和建设。

　　设区的市、县级人民政府发展改革部门或者本级人民政府确定的数字经济主管部门，负责统筹推进、协调、督促本行政区域内数字经济发展工作。

　　县级以上人民政府其他有关部门按照各自职责，做好促进数字经济发展相关工作。

　　第六条　县级以上人民政府应当将数字经济发展纳入国民经济和社会发展规划。

　　省人民政府发展改革部门应当编制全省数字经济发展专项规划，报省人民政府批准后发布实施。

　　设区的市、县级人民政府发展改革部门或者本级人民政府确定的数字经济主管部门应当根据全省数字经济发展专项规划的要求和实际需要，编制本行政区域数字经济发展专项规划或者实施方案，报本级人民政府批准后发布实施。

　　第七条　县级以上人民政府统计主管部门应当按照国家数字经济分类标准，研究建立数字经济统计监测机制，加强数字经济发展情况和变化态势的运行监测分析，利用数字经济统计结果开展研究，提供统计资料和统计咨询意见，为本地区数字经济发展决策提供服务，并依法向社会公布数字经济发展情况。

　　第八条　县级以上人民政府及其有关部门应当推动数字经济开放合作，积极参与“一带一路”建设，加强同有关国家和地区在数字经济领域的交流合作和商务贸易。

　　省人民政府及其有关部门应当按照京津冀协同发展战略要求，加强与北京市、天津市人民政府及其有关部门在数字经济领域的交流合作，推动数字经济协同发展。设区的市、县级人民政府应当按照上级人民政府统一部署，根据当地比较优势，推动京津冀数字经济协同发展。

　　鼓励社会力量参与数字经济发展，加强国内外交流合作。

第二章　数字基础设施建设

　　第九条　县级以上人民政府及其有关部门、县级以上网信部门和省通信管理部门应当按照技术先进、适度超前、安全可靠、覆盖城乡、服务便捷的原则，建立和完善数字基础设施体系，重点统筹通信网络基础设施、新技术基础设施、算力基础设施等建设，推进传统基础设施的数字化改造。

　　数字基础设施应当实行集约化建设和管理，提高基础设施利用率，防止重复建设。

　　第十条　数字经济发展专项规划确定的数字基础设施建设和布局应当纳入国土空间规划，编制市政、交通、电力、公共安全等相关基础设施专项规划应当考虑数字基础设施建设的需要。

　　县级以上人民政府应当推动数字基础设施与铁路、城市轨道、道路、桥梁、隧道、电力、地下综合管廊、机场、港口、邮政快递、枢纽站场、智慧杆塔等基础设施以及相关配套设施共商共建共享共维。

　　第十一条　省通信管理部门应当加强通信网络布局和建设，推进新一代固定和移动通信网络建设，加强通信网络骨干网、城域网和接入网建设，提高网络容量、通信质量和传输速率。

　　新建、改建、扩建工程，根据相关规划需要配套建设数字基础设施的，工程建设、设计等相关单位应当按照有关建设设计标准和规范，预留通信网络等数字设施所需的空间、电力等资源，并与主体工程同步设计、同步施工、同步验收。

　　第十二条　省人民政府发展改革部门、工业和信息化主管部门、省网信部门和省通信管理部门应当统筹推进新型数据中心、云计算中心、超算中心、智能计算中心等建设及传统数据中心升级改造，支持企业建设公共算力服务平台，构建布局合理、存算均衡、绿色低碳、安全高效的算力基础设施。

　　设区的市、县级人民政府应当按照上级人民政府统一部署，推进算力基础设施建设。

　　第十三条　县级以上人民政府及其有关部门、省通信管理部门应当推进物联网建设，加强城乡基础设施、城乡治理、物流仓储、生产制造、生活服务等领域感知系统的建设和应用，推动感知系统共建共用和数据共享。

　　第十四条　省通信管理部门、省人民政府工业和信息化主管部门应当推动工业互联网基础设施建设，完善工业互联网标识解析体系，构建工业互联网平台体系，建立健全工业互联网安全保障体系。

　　鼓励企业积极开展企业内外网改造和网络配套能力建设，支撑企业数字化、网络化、智能化发展。

　　第十五条　省通信管理部门、县级以上网信部门、县级以上人民政府农业农村主管部门等应当加快农村地区数字基础设施建设，强化传统基础设施数字化改造和数字基础设施优化升级，推进县域内城乡数字设施一体化，提高乡村光纤网络、移动网络建设水平和覆盖水平，加快数字乡村建设。

第三章　数据资源开发利用

　　第十六条　数据资源开发利用应当遵循依法规范、促进流通、合理使用、保障安全的原则，加强数据资源全生命周期管理，提高数据要素质量，培育发展数据要素市场，激发数据要素潜能。

　　第十七条　国家机关、法律法规授权的具有管理公共事务职能的组织以及供水、供电、供暖、供气、民航、铁路、通信、邮政、公共交通等提供公共服务的组织（以下统称公共管理服务机构），在依法履行公共管理和服务职责过程中收集和产生的各类数据（以下统称公共数据），由省、设区的市、县（市、区）确定的公共数据主管部门实行统筹管理。

　　省公共数据主管部门应当会同公共管理服务机构制定统一的公共数据分类规则、分类标准和分类管理要求，对公共数据采集、汇聚、共享、开放、开发、交易、安全、销毁等全生命周期采取差异化管理措施。

　　设区的市、县级公共数据主管部门应当按照公共数据统筹管理要求，开展本行政区域内公共数据管理工作。

　　第十八条　本省公共数据实行目录管理。公共管理服务机构履行职责需要收集公共数据的，应当坚持一数一源、标准统一和谁收集、谁更新、谁负责的原则，对可以通过共享方式获得的数据，不得重复收集、多头收集。

　　公共管理服务机构应当加强数据质量管控，健全数据纠错机制，确保数据准确性、完整性和时效性。

　　企业、行业组织、科研机构等应当依法依规开展行业和市场数据收集。

　　第十九条　任何组织和个人收集数据，应当采取合法、正当的方式，不得窃取或者以其他非法方式获取数据。

　　法律、行政法规对收集、使用数据的目的、范围有规定的，应当在法律、行政法规规定的目的和范围内收集、使用数据。

　　第二十条　省、设区的市、县（市、区）应当建立健全公共数据共享协调推进机制，统筹协调本行政区域公共数据共享工作。

　　公共管理服务机构之间共享公共数据，应当坚持以共享为原则、不共享为例外，分为无条件共享、有条件共享和不予共享三种类型。

　　可以提供给所有公共管理服务机构共享使用的公共数据属于无条件共享类，只能提供给部分公共管理服务机构共享使用或者仅部分内容能够提供给公共管理服务机构共享使用的公共数据属于有条件共享类。凡列入不予共享类公共数据的，应当有法律、法规或者国家相关规定作为依据。

　　第二十一条　公共数据共享应当通过公共数据共享平台实现。公共管理服务机构应当将本单位无条件共享类和有条件共享类的公共数据汇聚到公共数据共享平台。

　　省公共数据共享平台汇聚的公共数据应当按照各地需求，根据属地原则及时回流至设区的市公共数据共享平台。已回流至设区的市公共数据共享平台的数据，设区的市公共数据共享平台不得重复收集。

　　公共管理服务机构通过公共数据共享平台获取公共数据应当明确具体应用场景，获取的公共数据仅用于本部门履行职责需要，不得提供给第三方，也不得用于其他目的。除法律、行政法规另有规定外，在公共数据共享平台获取的电子证照和加盖电子印章的电子材料可以作为办理政务服务事项的依据。

　　鼓励企业和其他组织向公共管理服务机构提供自有数据，推动政企公共数据共享机制建设，逐步扩大公共数据共享范围。

　　第二十二条　省、设区的市公共数据主管部门应当按照需求导向、依法有序、分类分级、安全可控的原则推进公共数据开放。公共管理服务机构向社会开放公共数据应当通过统一的公共数据开放平台实现。鼓励优先开放对民生服务、社会治理和产业发展具有重要价值的数据。

　　第二十三条　县级以上人民政府及其有关部门应当通过产业政策引导、社会资本引入、应用模式创新、强化合作交流等方式，引导企业和其他组织依法开放自有数据资源，促进各类数据深度融合。

　　鼓励企业和其他组织通过公共数据开放平台，对外提供各类数据服务。

　　第二十四条　县级以上人民政府及其有关部门应当培育数据要素市场主体，鼓励研发数据技术、推进数据应用，依法开展数据清洗、分析、挖掘和加工，形成数据产品和服务。

　　第二十五条　组织、个人依法获取并合法处理数据形成的数据产品和服务，所产生的财产权益受法律保护，可以依法交易。法律另有规定的除外。

　　县级以上人民政府及其有关部门应当按照国家数据管理有关规定，推动数据要素资源依法有序自由流动，支持开展数据资产管理、数据交易、结算交付等业务。

　　第二十六条　县级以上网信等部门应当按照国家统一部署，协调落实数据安全和相关监管职责，建立健全数据安全风险评估、报告、信息共享、监测预警机制，加强本地区数据安全风险信息的获取、分析、研判、预警工作，保障数字经济安全发展。

　　网络运营者应当依法建立健全内部数据安全管理制度，加强重要领域数据资源、重要网络、信息系统和硬件设备安全保障，采取相应技术措施和其他必要措施，保障数据安全。

　　第二十七条　国家机关、法律法规授权的具有管理公共事务职能的组织为履行法定职责需要收集、使用数据的，应当在其履行法定职责的范围内依照法律、行政法规规定的条件和程序进行；对在履行职责中知悉的个人隐私、个人信息、商业秘密、保密商务信息等数据应当依法予以保密，不得泄露或者非法向他人提供。

　　第二十八条　任何组织和个人收集、存储、使用、加工、传输、提供、公开数据资源，应当遵守有关法律、法规的规定，尊重社会公德和伦理，遵守商业道德和职业道德，诚实守信，履行数据安全保护义务，承担社会责任，不得危害国家安全、公共利益，不得损害个人、组织的合法权益。

　　任何组织和个人应当加强个人信息保护，依法依规开展涉及个人信息的数据处理活动。

第四章　数字产业化

　　第二十九条　省人民政府应当根据国内外数字经济的技术、产业发展趋势，结合本省数字产业发展水平和各地区经济禀赋差异，统筹规划全省数字产业空间布局、功能定位和发展方向，提高数字产业整体竞争力。

　　第三十条　县级以上人民政府及其工业和信息化主管部门、发展改革部门，县级以上网信部门、省通信管理部门等应当按照全省数字产业发展要求，结合本地实际，制定政策措施，加快发展电子信息制造业、软件和信息服务业、电信广播电视和卫星传输服务业、互联网服务业，重点推动现代通信、新型显示、半导体材料与器件、汽车电子及产品、软件产品及服务、人工智能及智能装备、网络安全等新一代信息技术产业发展，培育区块链、量子信息、虚拟现实等产业。

　　第三十一条　县级以上人民政府及其发展改革、工业和信息化、商务等部门应当围绕当地数字经济发展需求，加强数字经济相关产业项目谋划、储备、引进、建设、投产全环节、全链条管理，优化招商引资各项服务，培育和引进技术水平高、带动能力强、聚集效应好的数字经济龙头企业，延伸产业链条，吸引配套产业，完善数字产业体系。

　　第三十二条　县级以上人民政府及其科学技术等部门应当引导支持企业联合高等院校、科研院所开展人工智能、物联网、云计算、大数据、网络安全等领域关键核心技术研发攻关，提高数字经济核心竞争力。

　　第三十三条　县级以上人民政府及其发展改革、工业和信息化等部门应当制定鼓励数字产业发展的政策措施，引进国内外数字产业领域优势企业，培育数字产业领域骨干企业、高成长型企业和创新型中小企业等多层次、递进式企业梯队，支持特色企业专注行业细分市场，形成产业链上下游联动、大中小企业融通发展的数字产业生态。

　　第三十四条　县级以上人民政府及其商务、科学技术、工业和信息化、发展改革等部门应当采取措施推动数字产业向园区集聚，培育数字产业集群。

　　各类园区应当培育或者引进数字产业服务第三方机构、工业互联网平台等企业，面向园区内外企业提供数字化转型供需撮合、咨询、培训、解决方案、展览展示、知识产权保护、投资融资等服务，推动设计、制造、检验检测等设备平台化汇聚与共享。

　　第三十五条　县级以上人民政府及其发展改革、工业和信息化、市场监督管理、商务等部门应当培育发展新业态新模式。推动平台经济健康发展，支持平台经营者利用技术、市场、数据等优势，提供普惠化、便捷化和个性化服务。发展共享出行、共享租住、共享物品等共享经济，拓展创新、生产、供应链等资源共享空间。发展基于数字技术的智能经济，推动智慧销售、无人配送以及众创众包众扶众筹等新业态新模式发展。

　　第三十六条　县级以上人民政府及其科学技术、发展改革、工业和信息化等部门应当推动数字经济领域重点实验室、工程研究中心、技术创新中心、制造业创新中心等创新平台和新型研发机构建设，建设产学研用一体化平台，完善公共技术服务平台和大型科技基础设施，建立健全创新平台和基础设施共享机制，提高科技创新能力。

　　县级以上人民政府及其有关部门、各类园区应当支持企业、高等院校、科研院所及其他单位创建数字经济领域科技企业孵化器、大学科技园和众创空间等线上线下创新创业平台。

第五章　产业数字化

　　第三十七条　县级以上人民政府及其有关部门应当通过规划引导、试点示范、政策支持、服务指导等方式，推动企业广泛应用新一代信息技术，实现工业、农业、服务业等产业数字化转型，提高产业发展质量和效益。

　　第三十八条　省人民政府工业和信息化主管部门应当制定重点行业数字化转型路线图，推动钢铁、装备制造、石油化工、食品、医药等传统优势行业加快数字化转型。支持重点行业、龙头企业在数字化转型过程中形成数字设计研发、智能生产制造、数字运维等领域的自主数字产品和数字服务，推动传统产业优势转化为数字经济发展优势。

　　第三十九条　县级以上人民政府工业和信息化主管部门应当鼓励和支持产业集群龙头企业加快数字化转型，带动产业集群中小企业普及数字化应用，促进集群企业协同发展。

　　第四十条　县级以上人民政府工业和信息化主管部门应当通过试点示范、宣传指导、政策支持等方式，推进工业互联网平台建设，改造提升传统产业，发展先进制造业，提高企业生产和管理效能。

　　推动大型工业企业开展工业互联网集成应用创新，带动供应链企业数字化转型。推动中小型工业企业运用低成本、快部署、易运维的工业互联网解决方案，普及应用工业互联网。

　　第四十一条　县级以上人民政府及其工业和信息化、生态环境等部门应当推动实施“互联网＋”绿色制造。加快工业互联网、大数据、云计算、数字孪生、区块链等新一代信息技术在绿色制造领域的应用，提高绿色制造效率和效益。

　　省人民政府发展改革、生态环境等部门应当推动建设生态环境保护信息化平台，构建资源能源和污染物公共数据库，提升资源能源管理与环保治理精准化水平。引导工业企业应用新一代信息技术开展资源能源和污染物全过程动态监测、精准控制和优化管理，推动碳减排，助力实现碳达峰、碳中和。

　　第四十二条　县级以上人民政府能源主管部门应当加强数字技术在能源产业链各环节的深入应用，加快传统能源和新型能源生产的数字化改造，推动实现能源生产的实时监测、精准调度、故障判断和预测性维护，提升能源生产效能。

　　推动电力基础设施数字化，建设完善能源大数据中心，推进能源数据汇聚，加强综合能源网络建设，实现分布式能源和分布式智能微电网协调互补，推进新型绿色能源生产和消费。

　　第四十三条　县级以上人民政府农业农村主管部门应当推动发展智慧农业。依托智慧农业云平台，促进农业物联网应用，推进农业单品种生产、加工、流通、销售、消费的全产业链大数据建设，推广数字田园、智慧养殖、数字植物工厂、数字渔业、数字种业等高端农业，推动遥感监测、地理信息等信息通信技术在农田建设、农机作业、农产品质量安全追溯等方面的应用，实现农产品生产动态监测、物联网管控、生产数据自动汇总及分析，提高农业生产精细化、智慧化水平。

　　第四十四条　县级以上人民政府交通运输主管部门应当推动发展智慧交通。完善交通综合运行协调与应急指挥平台，构建综合交通大数据中心体系，建设涵盖重点领域、重点区域、移动装备的交通运行监测系统，加强数据资源的整合共享、综合开发和智能应用。推进智能网联汽车和智慧交通应用示范，建设智慧港口、智慧民航，提高智能运输和智能出行能力。

　　第四十五条　县级以上人民政府及其发展改革、交通运输、商务、邮政管理等部门应当加强智慧物流体系建设。推进物流枢纽智能化升级，推广仓储数字管理、车辆货物自动匹配、园区智能调度等应用，推动物流园区间信息共享和业务协作，提升智能仓储、智能货运服务能力。

　　推进各类数据跨运输方式、跨部门、跨区域的互联互通，建设高效多式联运体系，提高贸易流通数字化水平。

　　第四十六条　县级以上人民政府文化和旅游等部门应当推动数字技术在文化和旅游业的应用。推进文化数字化基础设施和服务平台建设，支持数字文化创意试验区建设，推动网络视听、数字影视、数字动漫、网络游戏、数字广告、数字出版、互动新媒体等数字文化创意产业发展，推进历史文化遗产和文物的数字化镜像建设，促进传统工艺与人工智能、大数据等深度融合。完善全省文化旅游大数据中心建设，整合全域旅游、乡村旅游、数字博物馆、数字图书馆、数字文物资源等数据资源，开展智慧景区试点示范，提高旅游业数字化、智慧化管理和服务水平。

　　第四十七条　县级以上人民政府卫生健康主管部门应当推动发展智慧医疗。加强公共卫生信息监测预警体系建设，完善省市县三级全民健康信息平台，发展互联网医院、远程医疗、线上便民服务等“互联网＋”健康医疗新模式。推动新一代信息技术和智能硬件产品在医疗和公共卫生事件防控等领域的应用，提高医疗、公共卫生服务能力和技术水平。

　　县级以上人民政府民政、工业和信息化、卫生健康等部门应当推广智慧健康养老，支持建设健康养老创新中心、养老服务信息平台和健康养老综合服务平台，促进新一代信息技术和智能硬件产品在养老服务领域深度应用。

　　第四十八条　县级以上人民政府教育行政部门应当推动发展智慧教育。推进教育新型基础设施建设，加快数字校园建设，完善河北“教育云”功能，推进各类优质教育资源跨层级、跨地区在线共享，培育互动教学、个性定制、智慧课堂等新模式，全面提高教育数字化水平。

　　第四十九条　县级以上人民政府体育主管部门应当推动发展智慧体育。推进体育场馆和设施数字化改造，完善训练赛事和市民健身运动的数字化服务体系，支持发展线上体育赛事、体育健身培训、体育装备销售、体育直播、电子竞技等业态，推动数字技术与冰雪运动和冰雪产业融合发展。

　　第五十条　省人民政府商务、工业和信息化等部门应当引导钢铁、建材、食品、医药、煤炭、石化、汽车等大型工业企业采购销售平台向行业电子商务平台转型，鼓励工业企业与平台对接，推进网上交易、支付结算、供应链金融、大数据分析等综合服务延伸，提高供应链产业链协同水平。

　　县级以上人民政府商务、农业农村、邮政管理等部门应当支持城乡电子商务发展，完善城乡电子商务和邮政快递设施，建立综合服务平台，推进跨境电子商务发展，培育社交电子商务、直播电子商务等新业态新模式。

　　第五十一条　县级以上人民政府及其地方金融监督管理部门、所在地国家金融管理部门派出机构应当推动发展数字金融。加快金融与数字技术融合创新发展，实现新一代信息技术在支付结算、信贷融资、保险业务、征信服务等金融领域融合应用，推动金融业数字化发展。

第六章　数字化治理

　　第五十二条　县级以上人民政府及其有关部门应当加强数字政府建设，推动数字技术在经济调节、市场监管、社会治理、公共服务、生态环境保护、政府自身运行等领域的应用，逐步实现政府履职全业务、全流程数字化，提高政府科学决策、高效监管、精准治理水平。

　　第五十三条　县级以上人民政府及其有关部门应当依托一体化政务服务平台开展政务服务工作。新建政务服务信息系统应当依托一体化政务服务平台进行建设，不得单独建设，已建业务系统应当按照有关规定进行整合。

　　推动数字技术与政务服务深度融合，推进政务服务通过一体化政务服务平台实现全流程网上办理、掌上办理，提高行政效能和便民服务水平。

　　第五十四条　省政务服务主管部门应当推动建设全省监管数据中心，建设完善一体化在线监管平台，为行政执法部门执法提供数据共享服务，逐步实现依托一体化在线监管平台开展行政执法。行政执法部门应当按照规定，及时向全省一体化在线监管平台汇聚监管数据。

　　省政务服务主管部门应当加强全省一体化政务服务平台、一体化信用平台、一体化在线监管平台对接，推动政务服务数据、信用数据、执法监管数据跨部门、跨层级共享应用。

　　行政执法部门应当推动数字技术在执法领域的应用，推进非现场监管、信用监管、风险预警等监管模式，提高监管效能和水平。

　　第五十五条　县级以上人民政府及其有关部门应当加强数字技术在突发自然灾害、事故灾难、公共卫生事件和社会安全事件中的应用，推动应急救援信息共享共用，提高应急监测预警、应急救援处置能力。

　　鼓励组织、个人对开放的公共数据依法进行开发利用，为应对突发事件提供支持。

　　第五十六条　省人民政府发展改革部门、省网信部门应当统筹指导新型智慧城市建设，构建智慧城市评价激励体系，推进城市综合管理涉及的各类数据互联互通。

　　设区的市、县级人民政府应当逐步推进智慧城市建设，提升城市道路桥梁、智慧管廊、智慧杆塔等基础设施运行数据的感知和采集能力，整合城市交通、平安建设、医疗健康、公共卫生、生态环境保护、文化旅游等领域的数据资源，推动建设城市大脑，实现城市运行态势监测、公共资源配置、宏观决策、统一指挥调度和事件分拨处置数字化，提高城市治理水平。

　　第五十七条　县级以上人民政府及其有关部门应当推动城乡基层社会治理数字化，推动公共数据向基层共享应用，运用数字技术统筹规划、协调推进基层公共安全、公共管理、公共服务等治理，提高基层治理的精准化、智能化水平。

　　第五十八条　县级以上人民政府及其有关部门应当按照优化传统服务与创新数字服务并行的原则，制定和完善老年人、残疾人等运用数字技术困难群体在出行、就医、消费、文娱、办事等方面的服务保障措施，鼓励企业和科研机构开发高水平适老化智能产品和防网络诈骗功能，开展信息无障碍改造，保留必要的线下办事渠道，保障和改善运用数字技术困难群体的服务需求和服务体验。

第七章　京津冀数字经济协同发展

　　第五十九条　省人民政府及其有关部门应当推动与北京市、天津市协同建设移动基站、传输光缆、算力等信息基础设施，协同建设工业互联网、智慧能源、智慧民生等融合基础设施，协同建设重大科技、科教、产业技术创新平台等创新基础设施，推进新型基础设施建设标准协同、布局协同、应用协同。

　　第六十条　省人民政府及其有关部门应当推进与北京市、天津市执行统一的数据技术规范，实现公共数据信息系统兼容。

　　省人民政府及其政务服务、市场监督管理等部门应当推动与北京市、天津市数据共享交换，支撑京津冀政务服务协同、监管协同和有关场景应用建设。推进京津冀区域信用合作工作机制，在信用制度规范、信用信息共享、信用服务市场、奖惩机制等方面开展创新示范。

　　第六十一条　省人民政府及其发展改革、工业和信息化、科学技术部门和省网信部门等应当根据本省在京津冀区域功能定位，统筹对接京津数字经济相关产业发展、承接产业转移成果，协同谋划数字经济产业发展布局，打造数字经济产业链集群、产业链生态，提升区域内数字经济产业链整体竞争力。

　　省人民政府及其发展改革、商务、交通运输、邮政管理、市场监督管理等部门应当推动完善京津冀物流对接合作长效机制，构建与北京市、天津市在要素、网络、标准等方面一体化的现代物流体系，推动多式联运电子化统一单证京津冀区域共认，推进机场、铁路、公路、货运场站等生产性物流设施京津冀共建共享共用，提升京津冀海关通关一体化效率，助力京津冀一体化产业链供应链建设。

　　第六十二条　省人民政府及其科学技术主管部门应当推动京津冀协同创新共同体建设，承接京津科技创新资源，推动建设河北京津冀国家技术创新中心，建设环京津科技园区、协同创新重要节点区、国家科技成果转移转化示范区，打造京津科技成果转移转化承载高地。

　　第六十三条　省人民政府及其有关部门应当统筹推进与北京市、天津市合作，推动京津优质医疗、公共卫生、健康、教育、文化、体育等资源通过数字手段、数字渠道在本省广泛应用，提升公共服务水平。

　　第六十四条　省人民政府及其生态环境主管部门应当推进与北京市、天津市生态环境联防联治实现一体化、网络化、智能化，深化大数据在京津冀生态环境监测、监管等领域的应用，推进生态环境治理与北京市、天津市标准统一、监管协同。

　　第六十五条　河北雄安新区管理委员会应当按照国家数字经济创新发展试验区建设要求，在智慧城市建设、数字要素流通、体制机制创新等方面先行先试，打造数字经济创新发展领军城市。

　　省人民政府及其有关部门应当及时总结推广雄安新区制度创新成果，为本省其他区域对接雄安新区产业及要素溢出提供必要条件，推动雄安新区辐射带动本省数字经济高质量发展。

第八章　保障和监督

　　第六十六条　县级以上网信部门，县级以上人民政府及其教育、工业和信息化、人力资源和社会保障等部门应当构建覆盖全民、城乡融合、公平一致、可持续、有韧性的数字素养与技能发展培育体系，推动数字教育资源、数字技能培训、数字产品和信息服务高质量发展和开放共享，加强数字技术、知识以及有关法律、法规的宣传，鼓励新闻媒体开展公益性宣传，推动建成全民终身数字学习体系，提高全民数字素养与技能。

　　第六十七条　县级以上人民政府应当建立多元化资金投入和保障机制，统筹运用相关财政性资金，发挥有关专项基金作用，鼓励和引导社会资本参与数字经济发展，重点支持大数据、物联网、信息制造、重大创新平台等建设。

　　第六十八条　县级以上人民政府应当支持符合条件的数字经济企业通过上市融资，拓展融资渠道。

　　县级以上人民政府及其地方金融监督管理部门、所在地国家金融管理部门派出机构应当培育、引进天使投资、风险投资等投资机构，为公募基金、私募基金对接数字经济相关产业发展提供条件，鼓励银行等金融机构加大创新力度，开发信息科技融资、知识产权质押融资、供应链金融等符合数字经济相关产业投融资特点的产品和服务，发挥政府性融资担保作用。推动建设金融机构与企业间常态长效信息对接平台。支持保险机构为符合政策的数字经济企业和项目贷款提供保证保险和信用保险，鼓励保险机构开发适应数字经济发展特点的新型保险产品。

　　第六十九条　省人民政府及其科学技术主管部门应当推动数字经济技术创新，加强数字经济技术研究，加快技术成果转化，建立完善产业技术创新体系和共性基础技术供给体系。构建国家重大科技项目承接机制，推动获取重大原创科技成果和自主知识产权。

　　县级以上人民政府及其科学技术、教育等部门应当加强产业链协同创新统筹协调，制定政策措施，引导高等院校、科研院所、科技企业协同攻关，推进数字经济产学研用合作，支持共建企业技术创新联盟、科技创新基地、国际科技合作基地、院士工作站、博士后科研工作站、博士工作站等平台，加强数字经济科研力量优化配置和资源共享。

　　省、设区的市人民政府科学技术主管部门可以向符合条件的科技型中小企业和创新创业团队发放科技创新券，支持开展技术开发、技术转让、试验测试、创新方法培训等服务，推动数字经济科技创新。

　　第七十条　县级以上人民政府及其有关部门应当依托重大人才工程，引进数字经济领域高水平专家人才和创新团队，并在住房、落户、科研经费、医疗保险及配偶就业、子女入学等方面给予支持。支持以挂职兼职、技术咨询、项目合作等方式柔性引进重点人才。

　　省人民政府及其教育、人力资源和社会保障部门应当支持高等院校加强数字经济新兴领域学科专业和专业课程建设。支持高等院校、职业学校、科研院所与数字经济企业深化产教融合、校企合作，共建数字经济专业学院和实训基地，培养计算机科学、软件工程、人工智能、数据科学、电子工程等数字经济紧缺人才。

　　县级以上人民政府及其人力资源和社会保障等部门应当加强劳动用工服务指导，清理对灵活就业的不合理限制，鼓励依托数字经济创造更多灵活就业机会，完善平台经济、共享经济等新业态从业人员在工作时间、报酬支付、保险保障等方面的规定。

　　第七十一条　县级以上人民政府及其有关部门应当完善政策措施，在土地供给、电力供应、能耗指标、设施保护、频谱资源、政府采购等方面保障数字经济发展。

　　第七十二条　县级以上人民政府及其政务服务、工业和信息化、科学技术、税务、人力资源和社会保障等部门应当推广应用数字化手段，开展大数据分析应用，针对不同市场主体，智能推送、高效落实各类惠企政策。

　　县级以上人民政府司法行政部门应当推进智慧法务体系建设，探索新型法律服务供给模式，推动云计算、大数据、人工智能等新一代信息技术与法律服务工作融合，提升法律服务智能化、均等化水平。

　　第七十三条　省、设区的市人民政府标准化行政主管部门应当会同发展改革、工业和信息化、网信等部门组织制定数字经济相关地方标准，完善数字经济标准体系。

　　县级以上人民政府应当组织和支持行业协会、产业联盟、企业事业单位参与制定、修订数字经济国际标准、国家标准、行业标准、地方标准。鼓励社会团体、企业制定满足市场和创新需要或者高于推荐性标准相关技术要求的团体标准、企业标准。

　　第七十四条　县级以上人民政府及其发展改革、工业和信息化、商务、科学技术、人力资源和社会保障等部门应当鼓励数字经济产业服务第三方机构，为数字经济产业相关企业提供创业培育和辅导、知识产权保护、投资融资、技术支持、产权交易等服务。

　　县级以上人民政府及其有关部门应当鼓励数字经济相关产业领域的社会组织加强行业服务，依法开展信息交流、企业合作、产业研究、人才培训、咨询评估等活动。

　　第七十五条　县级以上人民政府及其有关部门应当支持举办数字经济领域国际国内展会、论坛、研讨会等活动。运用中国国际数字经济博览会、中国·廊坊国际经济贸易洽谈会等平台，推进数字经济展示、交易、交流、合作。鼓励数字经济领域企业参加国内外展览展销等活动。

　　第七十六条　县级以上人民政府及其有关部门应当加强数字经济知识产权保护和服务，建立健全知识产权快速维权体系，依法查处侵犯知识产权行为。

　　第七十七条　县级以上人民政府及其有关部门应当依法开展对互联网平台经营者的监管，促进平台经济健康发展。

　　互联网平台经营者应当依法依约履行产品和服务质量保障、消费者权益保护、生态环境保护、知识产权保护、网络安全、数据安全与个人信息保护、劳动者权益保护等方面的义务，建立健全平台规则和用户账号信用管理、投诉举报等制度，提高平台自治水平。鼓励互联网平台经营者建立争议在线解决机制，制定和公示争议解决规则。

　　第七十八条　县级以上人民政府应当定期对本行政区域数字经济发展情况进行评估，并对下一级人民政府数字经济发展情况开展督导检查。

　　县级以上人民政府可以委托第三方机构开展本行政区域数字经济发展情况评估，并向社会公布。

　　第七十九条　国家机关、法律法规授权的具有管理公共事务职能的组织及其工作人员有下列行为之一的，由本级人民政府或者上级主管部门责令改正；情节严重的，由有关机关对负有责任的主管人员和其他直接责任人员依法给予处分；构成犯罪的，依法追究刑事责任：

　　（一）违反本条例规定重复收集、多头收集公共数据的；

　　（二）违反本条例规定，将通过公共数据共享平台获取的公共数据提供给第三方，或者用于其他目的的；

　　（三）未按照本条例规定履行公共数据共享和开放职责的；

　　（四）未按照本条例规定将本单位无条件共享类和有条件共享类的公共数据汇聚到公共数据共享平台，或者未按照本条例规定及时回流公共数据的；

　　（五）未按照本条例规定依托一体化政务服务平台新建政务服务信息系统，或者已建业务系统未按照有关规定进行整合的；

　　（六）未按照本条例规定推动政务服务数据、信用数据、执法监管数据跨部门、跨层级共享应用的；

　　（七）篡改、伪造或者指使篡改、伪造数字经济主要统计指标的；

　　（八）其他玩忽职守、滥用职权、徇私舞弊的行为。

　　第八十条　违反本条例规定的行为，法律、行政法规已有法律责任规定的，从其规定。

第九章　附　则

第八十一条　本条例自2022年7月1日起施行。

(9)广东省数字经济促进条例

2021年7月30日广东省第十三届人民代表大会常务委员会第三十三次会议通过

第一章　总　则

　　第一条　为了促进数字经济发展，推进数字产业化和产业数字化，推动数字技术与实体经济深度融合，打造具有国际竞争力的数字产业集群，全面建设数字经济强省，根据有关法律、行政法规，结合本省实际，制定本条例。

　　第二条　本条例适用于本省行政区域内促进数字经济发展，以及为数字经济提供支撑保障等相关活动。

　　本条例所称数字经济，是指以数据资源为关键生产要素，以现代信息网络作为重要载体，以信息通信技术的有效使用作为效率提升和经济结构优化的重要推动力的一系列经济活动。

　　第三条　数字经济发展应当遵循创新引领、数据驱动、融合赋能、包容审慎、安全发展的原则。

　　第四条　数字经济发展以数字产业化和产业数字化为核心。数字产业化主要促进数字产品制造业、数字产品服务业、数字技术应用业、数字要素驱动业的发展；产业数字化主要促进工业数字化、农业数字化、服务业数字化等数字化效率提升业的发展。

　　第五条　县级以上人民政府应当将数字经济发展纳入国民经济和社会发展规划，并根据需要制定本级数字经济发展规划。

　　省人民政府应当加强对全省数字经济发展的统筹部署，营造数字经济发展良好环境。地级以上市、县级人民政府应当及时掌握数字经济发展动态，协调解决重大问题，按照上级人民政府统筹部署组织实施。

　　第六条　省人民政府发展改革主管部门负责拟制促进数字化发展战略、规划和重大政策，推进数字化发展重大工程和项目实施；工业和信息化主管部门负责促进数字经济发展工作，拟制促进数字经济发展的战略、规划和政策措施并组织实施；统计主管部门负责建立数字经济统计监测机制，开展数字经济统计调查和监测分析，依法向社会公布。

　　地级以上市、县级人民政府工业和信息化主管部门或者本级人民政府确定的主管部门，负责推进数字经济发展具体工作。

　　县级以上人民政府其他有关部门按照职责分工，做好数字经济发展工作。

　　第七条　省人民政府及有关部门应当加强与“一带一路”沿线国家和地区在数字基础设施、数字商贸、数字金融、智慧物流等领域的交流合作，扩大数字经济领域开放。加强粤港澳大湾区数字经济规则衔接、机制对接，推进网络互联互通、数字基础设施共建共享、数字产业协同发展。

　　县级以上人民政府及有关部门应当按照本省关于珠三角核心区、沿海经济带、北部生态发展区的区域发展格局，加强数字经济区域优势互补、差异化协调发展。

　　鼓励社会力量参与数字经济发展，加强国内外交流合作。

　　第八条　引导企业等市场主体在促进数字经济发展政策支持下，进行数字化转型。支持和鼓励各类市场主体参与数字经济领域投资建设。

　　支持行业协会、科研机构、高等学校以及其他组织为促进数字经济发展提供创业孵化、投资融资、技术支持、法律服务、产权交易等服务。

第二章　数字产业化

　　第九条　县级以上人民政府应当促进计算机通信和其他电子设备制造业、电信广播电视和卫星传输服务、互联网和相关服务、软件和信息技术服务业等发展，培育人工智能、大数据、区块链、云计算、网络安全等新兴数字产业，谋划布局未来产业。

　　第十条　省人民政府及发展改革、科技、工业和信息化等有关部门应当统筹规划集成电路产业发展，提升基金、平台、高等学校、园区支撑水平，从制造、设计、封测、材料、装备、零部件、工具、应用等方面构建产业支柱，支持优质项目投资建设，打造集成电路产业创新发展高地。

　　第十一条　省人民政府及科技、工业和信息化等有关部门应当统筹规划软件产业发展，培育具有自主知识产权的软件产业，推进软件产品迭代、平台搭建、产业化应用、适配测试和开源开放，拓展用户市场，构建安全可控、共建共享的软件产业生态。

　　第十二条　省人民政府及工业和信息化、通信管理等有关部门应当统筹规划新一代移动通信产业发展和应用创新，加强材料、制造工艺等领域前沿布局，构建集材料、芯片、基站、设备、终端、应用于一体的新一代移动通信产业链。

　　第十三条　县级以上人民政府及发展改革、商务、市场监督管理等有关部门应当培育互联网平台企业，支持利用互联网平台推进资源集成共享和优化配置。依法依规明确平台企业定位和监管规则，促进平台经济和共享经济规范有序创新健康发展。

　　互联网平台经营者应当建立健全平台管理规则和制度，依法依约履行产品和服务质量保障、网络安全保障、数据安全保障、消费者权益保护、个人信息保护等方面的义务。

　　第十四条　县级以上人民政府及发展改革、科技、工业和信息化、商务、市场监督管理等有关部门应当引导支持数字经济领域的龙头企业、高新技术企业，以及科技型中小企业和专业化、精细化、特色化、新颖化中小企业发展。

　　县级以上人民政府及地方金融监督管理等有关部门应当培育数字经济领域企业上市资源，支持有条件的企业依法到证券交易机构上市。

　　第十五条　县级以上人民政府及发展改革、科技、工业和信息化等有关部门应当结合本地实际，引导支持数字产业基地和园区建设，重点培育下列数字产业集群：

　　（一）新一代电子信息；

　　（二）软件与信息服务；

　　（三）超高清视频显示；

　　（四）半导体与集成电路；

　　（五）智能机器人；

　　（六）区块链与量子信息；

　　（七）数字创意；

　　（八）其他重要数字产业集群。

　　第十六条　引导互联网企业、行业龙头企业、基础电信企业开放数据资源和平台计算能力等，支持企业、科研机构、高等学校等创建数字经济领域众创空间、科技企业孵化器、科技企业加速器、大学科技园等创新创业载体，构建协同共生的数字经济产业创新生态。

第三章　工业数字化

　　第十七条　县级以上人民政府应当推进工业实施全方位、全角度、全链条的改造，提升全要素生产率，加快工业生产模式和企业形态变革，促进工业数字化、网络化、智能化转型。

　　第十八条　县级以上人民政府及工业和信息化、通信管理等有关部门应当推动跨行业、跨领域以及特色型、专业型工业互联网平台建设，支持企业改造提升工业互联网内外网络，建立完善工业互联网标识解析体系，健全工业互联网安全保障体系。

　　第十九条　省人民政府及科技、工业和信息化、通信管理等有关部门应当通过推动工业互联网平台、网络、标识解析、安全等关键技术突破，增强工业芯片、工业软件、工业操作系统等供给能力，实现工业制造技术和工艺数字化、软件化。

　　第二十条　县级以上人民政府及工业和信息化等有关部门应当推动工业数字化产业生态建设，培育工业数字化转型服务商，以提供数字化平台、系统解决方案以及数字产品和服务。

　　第二十一条　县级以上人民政府及工业和信息化等有关部门应当推动发展智能制造，加强工业互联网创新应用，支持工业企业实施数字化改造，推进工业设备和业务系统上云上平台，建设智能工厂、智能车间，培育推广智能化生产、网络化协同、个性化定制、服务化延伸、数字化管理等新业态新模式。

　　第二十二条　县级以上人民政府及工业和信息化、国有资产监督管理等有关部门应当推动大型工业企业开展集成应用创新，推进关键业务环节数字化，带动供应链企业数字化转型。推动中小型工业企业运用低成本、快部署、易运维的工业互联网解决方案，普及应用工业互联网。

　　第二十三条　县级以上人民政府及工业和信息化等有关部门应当结合本地实际，推进产业集群数字化改造，推动产业集群利用工业互联网进行全要素、全产业链、全价值链的连接，通过信息、技术、产能、订单共享，实现跨地域、跨行业资源的精准配置与高效对接。

　　支持产业集群骨干企业、工业数字化转型服务商等组建产业联合体，开发推广行业通用的技术集成解决方案，促进集群企业协同发展。

第四章　农业数字化

　　第二十四条　县级以上人民政府应当加快种植业、种业、林业、畜牧业、渔业、农产品加工业等数字化转型，推动发展智慧农业，促进乡村振兴。

　　第二十五条　县级以上人民政府及农业农村等有关部门应当推动遥感监测、地理信息等信息通信技术在农田建设、农机作业、农产品质量安全追溯等的应用，支持建设智慧农业云平台和农业大数据平台，探索智慧农业技术集成应用解决方案，提升农业生产精细化、智能化水平。

　　第二十六条　县级以上人民政府及农业农村、商务等有关部门应当支持新型农业规模经营主体、加工流通企业与电子商务企业融合，推动农产品加工、包装、冷链、仓储、配送等物流设施数字化建设，培育电子商务农产品品牌，促进农业农村电子商务发展。

　　第二十七条　县级以上人民政府及农业农村、通信管理等有关部门应当提升乡村信息网络水平，推动乡村信息服务供给和基础设施数字化转型。

　　第二十八条　县级以上人民政府及农业农村、文化和旅游等有关部门应当推动互联网与特色农业融合发展，培育推广创意农业、认养农业、观光农业以及游憩休闲、健康养生、创意民宿等数字乡村新业态新模式。

第五章　服务业数字化

　　第二十九条　县级以上人民政府应当重点推动智能交通、智慧物流、数字金融、数字商贸、智慧教育、智慧医疗、智慧文旅等数字应用场景建设，创新服务内容和模式，提升服务质量和效率。

　　第三十条　县级以上人民政府交通运输主管部门应当推动发展智能交通，加速交通基础设施网、运输服务网、能源网与信息网络融合发展，构建泛在先进的交通信息基础设施。构建综合交通大数据中心体系。培育推广智能网联汽车、自动驾驶船舶、自动化码头，以及定制公交、智能公交、智能停车等新业态新模式。

　　第三十一条　县级以上人民政府及发展改革、交通运输、邮政管理等有关部门应当推动发展智慧物流，推进货物、运输工具、场站等物流要素数字化，支持物流园区、大型仓储设施、货运车辆等普及应用数字化技术和智能终端设备，提升物流智能化水平。

　　第三十二条　县级以上人民政府地方金融监督管理部门应当推动发展数字金融，优化移动支付应用，推进数字金融与产业链、供应链融合。

　　按照国家规定探索数字人民币的应用和国际合作。

　　第三十三条　县级以上人民政府及商务等有关部门应当推动发展数字商贸，引导支持服务贸易和数字贸易的集聚区、平台及其促进体系发展。促进跨境电子商务综合试验区、数字服务出口基地建设，培育推广云服务、数字内容、数字服务、跨境电子商务等新业态新模式，支持数字化商贸平台建设，发展社交电子商务、直播电子商务等，完善发展机制、监管模式，建设与国际接轨的高水平服务贸易和数字贸易开放体系，提升数字商贸水平。

　　第三十四条　县级以上人民政府教育主管部门应当推动发展智慧教育，推进教育数据和数字教学资源互通共享，支持建设智慧校园、智慧课堂、互联网教育资源服务大平台，培育推广并规范管理互动教学、个性定制等在线教育新业态新模式。

　　第三十五条　县级以上人民政府卫生健康主管部门应当推动发展智慧医疗，推进人工智能、大数据、区块链和云计算在医学影像辅助诊断、临床辅助决策、智能化医学设备、公共卫生事件防控等领域的应用，加快开展网上预约、咨询、挂号、分诊、问诊、结算以及药品配送、检查检验报告推送等网络医疗服务，建设互联网医院，拓展医疗卫生机构服务空间和内容。

　　县级以上人民政府民政、卫生健康主管部门应当推动发展智慧健康养老产业，推动个人、家庭、社区、机构与健康养老资源有效对接和优化配置，促进健康养老服务智慧化升级，以满足个人和家庭多层次、多样化健康养老服务需求。

　　第三十六条　县级以上人民政府及网信、文化和旅游、广电、版权等有关部门应当推动发展互联网文体娱乐业等，支持建设公共文化云平台和智慧图书馆、博物馆等数字文化场馆，培育推广游戏、动漫、电竞、网络直播、融媒体等新业态新模式，发展网络视听、数字出版、数字娱乐、线上演播等产业，鼓励拓展优秀传统文化产品和影视剧、游戏等数字文化产品的海外市场。

　　县级以上人民政府及文化和旅游等有关部门应当推动发展智慧旅游，加强线上旅游宣传，推广在线预约预订服务，创新道路信息、气象预警等旅游公共服务模式，引导旅游景区开发数字化体验产品并普及景区电子地图、线路推荐、语音导览等智慧化服务。

第六章　数据资源开发利用保护

　　第三十七条　鼓励对数据资源实行全生命周期管理，挖掘数据资源要素潜力，发挥数据的关键资源作用和创新引擎作用，提升数据要素质量，培育数据要素市场，促进数据资源开发利用保护。

　　第三十八条　国家机关以及法律、法规授权的具有管理公共事务职能的组织在依法履行职责、提供服务过程中产生或者获取的公共数据，应当按照国家和省的有关规定进行分类分级，实行目录制管理。

　　县级以上人民政府政务服务数据管理部门统筹推进公共数据资源共享开放和开发利用，规范公共数据产品服务。国家机关以及法律、法规授权的具有管理公共事务职能的组织应当建立公共数据开放范围的动态调整机制，创新公共数据资源开发利用模式和运营机制，满足市场主体合理需求。

　　第三十九条　县级以上人民政府及政务服务数据管理等有关部门应当促进各类数据深度融合，鼓励依法依规利用数据资源开展科学研究、数据加工等活动，引导各类主体通过省统一的开放平台开放数据资源。支持构建工业、农业、服务业等领域数据资源开发利用场景。

　　第四十条　自然人、法人和非法人组织对依法获取的数据资源开发利用的成果，所产生的财产权益受法律保护，并可以依法交易。法律另有规定或者当事人另有约定的除外。

　　探索数据交易模式，培育数据要素市场，规范数据交易行为，促进数据高效流通。有条件的地区可以依法设立数据交易场所，鼓励和引导数据供需方在数据交易场所进行交易。

　　第四十一条　数据的收集、存储、使用、加工、传输、提供、公开等处理活动，应当遵守法律、法规，履行数据安全保护义务，尊重社会公德和伦理，遵守商业道德和职业道德，诚实守信，承担社会责任。

　　开展数据处理活动，不得危害国家安全、公共利益，不得损害个人、组织的合法权益。

　　个人信息受法律保护。个人信息的收集、存储、使用、加工、传输、提供、公开等处理活动，应当遵循合法、正当、必要原则，不得过度处理，并符合法律、法规规定的条件。

　　第四十二条　县级以上人民政府及网信、发展改革、工业和信息化、农业农村、商务、市场监督管理、政务服务数据管理等有关部门应当推广数据管理相关国家标准和行业标准，规范数据管理，提升数据质量。

　　探索推动产业数据的收集、存储、使用、加工、传输和共享，加强产业数据分类分级管理，支持企业提升数据汇聚、分析、应用能力，以及构建数据驱动的生产方式和企业管理模式。

第七章　数字技术创新

　　第四十三条　省人民政府及有关部门应当围绕数据的产生、传输、存储、计算与应用环节，推动数字技术创新，加强数字技术基础研究、应用基础研究和技术成果转化，完善产业技术创新体系和共性基础技术供给体系。

　　第四十四条　省人民政府及科技等有关部门应当围绕数字经济实施省重点领域研发计划重大专项，构建国家重大科技项目承接机制，推动获取重大原创科技成果和自主知识产权。

　　第四十五条　省人民政府及科技等有关部门应当探索建立数字经济关键核心技术攻关新型体制机制，重点在集成电路、基础软件、工业软件等基础领域，新一代移动通信、人工智能、区块链、数字孪生、量子科技、类脑计算等前沿技术领域，加快推进基础理论、基础算法、装备材料等关键核心技术攻关和突破。

　　第四十六条　省人民政府应当统筹规划、科学布局，推进数字经济领域省实验室建设，打造数字技术大型综合研究基地和原始创新策源地。

　　第四十七条　省人民政府及发展改革、科技、工业和信息化、市场监督管理等有关部门应当推动数字经济领域的科技创新平台、公共技术服务平台和重大科技基础设施建设，构建以企业为主体、市场为导向的技术创新体系。

　　第四十八条　县级以上人民政府及教育、科技等有关部门应当推进数字经济产学研合作，支持科研机构、高等学校等与企业共建技术创新联盟、科技创新基地、博士工作站、博士后科研工作站等创新平台，加强科研力量优化配置和资源共享，促进关键共性技术研发、系统集成和工程化应用。

　　支持数字技术开源平台、开源社区和开放技术网络建设，鼓励企业开放软件源代码、硬件设计和应用服务。

　　第四十九条　县级以上人民政府市场监督管理部门，以及其他行政主管部门应当加强数字经济标准化工作，依法对数字经济标准的实施进行监督。

　　支持社会团体、企业及其他组织开展数字经济国际国内标准交流合作，参与制定数字经济国际规则、国际国内标准，自主制定数字经济团体标准和企业标准。

　　第五十条　县级以上人民政府及教育、科技、工业和信息化、财政等有关部门应当支持科研机构、高等学校和企业完善数字技术转移机制；探索实施政府采购首台（套）装备、首批次产品、首版次软件等政策，支持创新产品和服务的应用推广；鼓励将财政资金支持形成的科技成果许可给中小企业使用，提升成果转化与产业化水平。

第八章　数字基础设施建设

　　第五十一条　县级以上人民政府应当完善数字基础设施体系，重点统筹通信网络基础设施、新技术基础设施、存储和计算基础设施等建设，推进传统基础设施的数字化改造，布局卫星互联网等未来网络设施。

　　第五十二条　数字基础设施的建设和布局应当纳入国土空间规划，市政、交通、电力、公共安全等相关基础设施规划应当结合数字经济发展需要，与数字基础设施相关规划相互协调和衔接。

　　第五十三条　县级以上人民政府及通信管理等有关部门应当支持新一代固定宽带网络和移动通信网络建设，推进核心网、承载网、接入网及基站、管线等信息通信网络建设。

　　工程建设、设计等相关单位应当按照有关建设设计标准和规范，预留信息通信网络设施所需的空间、电力等资源，并与主体工程同时设计、同时施工、同时验收。

　　推动通信设施与铁路、城市轨道、道路、桥梁、隧道、电力、地下综合管廊、机场、港口、枢纽站场、智慧杆塔等基础设施以及相关配套设施共商共建共享共维。

　　第五十四条　县级以上人民政府及有关部门应当推进物联网建设，积极部署低成本、低功耗、高精度、高可靠的智能化传感器，推进基础设施、城市治理、物流仓储、生产制造、生活服务、应急管理、生态保护等领域感知系统的建设应用、互联互通和数据共享。

　　县级以上人民政府及有关部门可以根据实际情况推进车联网建设，扩大车联网覆盖范围，提高路侧单元与道路基础设施、智能管控设施的融合接入能力，推进道路基础设施、交通标志标识的数字化改造和建设。

　　第五十五条　省人民政府及发展改革、科技、工业和信息化等有关部门应当统筹推进人工智能、区块链、云计算等新技术基础设施建设，支持建设底层技术平台、算法平台、开源平台等基础平台，建立领先的通用技术能力支撑体系。

　　第五十六条　省人民政府及发展改革、科技、工业和信息化、通信管理等有关部门应当统筹推进数据中心、智能计算中心、超级计算中心、边缘计算节点等存储和计算基础设施建设，支持优化升级改造，提升计算能力，构建高效协同的数据处理体系。

　　第五十七条　县级以上人民政府应当结合本地实际，推动能源、交通、城市、物流、医疗、教育、文化、自然资源、农业农村、水利、生态环境、应急等领域的传统基础设施数字化、智能化改造。

　　第五十八条　省人民政府自然资源主管部门应当统筹建设本省卫星导航定位基准服务系统和配套基础设施，提供卫星导航定位基准信息公共服务。

　　鼓励符合法定条件的组织参与卫星互联网基础设施建设，构建通信、导航、遥感空间基础设施体系。

第九章　保障措施

　　第五十九条　县级以上人民政府应当坚持数字经济、数字政府、数字社会一体建设，营造良好数字生态。在政务服务、财政、税收、金融、人才、知识产权，以及土地供应、电力接引、设施保护、政府采购等方面完善政策措施，为促进数字经济发展提供保障。

　　第六十条　省人民政府及政务服务数据管理部门应当推进数字政府改革建设，完善管运分离、政企合作的管理体制，创新建设运营模式，优化一网通办政务服务，推动一网统管省域治理，强化一网协同政府运行，提高政府数字化服务数字经济发展效能。

　　省人民政府及政务服务数据管理部门应当统筹规划全省政务网络基础设施建设，打造全省统一的政务基础网络、政务云平台和政务大数据中心，推进一体化网上政务服务平台以及移动政务平台的建设和应用。

　　第六十一条　省人民政府及有关部门统筹使用省级专项资金，有条件的地级以上市、县级人民政府在本级财政预算中安排资金，重点用于数字经济关键核心技术攻关、重大创新平台、公共技术平台和产业载体建设、应用示范和产业化发展、企业培育等领域。

　　县级以上人民政府应当依法落实数字经济的税收优惠政策。完善投融资服务体系，拓宽数字经济市场主体融资渠道。发挥省级政策性基金作用，重点支持数字经济领域重大项目建设和高成长、初创型数字经济企业发展。

　　第六十二条　县级以上人民政府及教育、人力资源社会保障等有关部门应当鼓励企事业单位、社会组织等培养创新型、应用型、技能型、融合型人才，支持高等学校、中等职业学校与企业开展合作办学，培养数字经济专业人才。

　　县级以上人民政府及人力资源社会保障主管部门应当将数字经济领域引进的高层次、高技能以及紧缺人才纳入政府人才支持政策范围，按照规定享受入户、住房、子女教育等优惠待遇。探索建立适应数字经济新业态发展需要的人才评价机制。

　　第六十三条　县级以上人民政府及市场监督管理、版权等有关部门应当加强数字经济领域知识产权保护，培育知识产权交易市场，探索建立知识产权保护规则和快速维权体系，依法打击知识产权侵权行为。

　　第六十四条　县级以上人民政府及市场监督管理部门应当依法查处滥用市场支配地位、实施垄断协议以及从事不正当竞争等违法行为，保障各类市场主体的合法权益，营造公平竞争市场环境。

　　县级以上人民政府及人力资源社会保障等有关部门应当加强劳动用工服务指导，清理对灵活就业的不合理限制，鼓励依托数字经济创造更多灵活就业机会，完善平台经济、共享经济等新业态从业人员在工作时间、报酬支付、保险保障等方面政策规定。

　　第六十五条　县级以上人民政府及网信、应急管理、政务服务数据管理、通信管理等有关部门，企业、平台等处理数据的主体应当落实数字经济发展过程中的安全保障责任，健全安全管理制度，加强重要领域数据资源、重要网络、信息系统和硬件设备安全保障，健全关键信息基础设施保障体系，建立安全风险评估、监测预警和应急处置机制，采取必要安全措施，保护数据、网络、设施等方面的安全。

　　第六十六条　县级以上人民政府应当建立数字经济创新创业容错免责机制，对新技术、新产业、新业态、新模式等实行包容审慎监管。

　　第六十七条　县级以上人民政府及有关部门应当加强数字经济宣传、教育、培训，加强数字技能教育和培训，普及提升全社会数字素养。

　　支持举办数字经济领域的国际国内会展、赛事等活动，搭建数字经济展示交易、交流合作平台，畅通供需对接渠道，提高市场开拓能力。

　　第六十八条　县级以上人民政府及有关部门应当推进信息无障碍建设，坚持创新智能化服务与改进传统服务并行。鼓励针对老年人、残疾人等运用智能技术困难的群体的出行、就医、消费、文娱、办事等，提供适用的智能化产品和服务，帮助其共享数字生活。

　　第六十九条　县级以上人民政府有关部门应当按照职责分工，制定执行本条例的工作计划，并定期向本级人民政府报告执行情况。

　　第七十条　县级以上人民政府应当定期对本级数字经济发展情况进行评估，并对下一级人民政府数字经济发展情况开展监督检查。

　　数字经济发展情况评估可以委托第三方机构开展，并向社会公布。

　　第七十一条　各级人民政府及有关部门在数字经济促进工作中不依法履行职责的，依照法律、法规追究责任，对直接负责的主管人员和其他直接责任人员依法给予处分。

　　违反有关网络安全、数据安全、个人信息保护等法律、法规的，由有关主管部门依法予以处罚；构成犯罪的，依法追究刑事责任。

第十章　附　则

第七十二条　本条例自2021年9月1日起施行。

(10)浙江省数字经济促进条例

2020年12月24日浙江省第十三届人民代表大会常务委员会第二十六次会议通过

目　录

　　第一章　总则

　　第二章　数字基础设施

　　第三章　数据资源

　　第四章　数字产业化

　　第五章　产业数字化

　　第六章　治理数字化

　　第七章　激励和保障措施

　　第八章　法律责任

　　第九章　附则

第一章　总则

　　第一条　为了促进数字经济发展，加快建设现代化经济体系，提升核心竞争力，推动高质量发展，推进省域治理现代化，根据有关法律、行政法规，结合本省实际，制定本条例。

　　第二条　本省行政区域内促进数字经济发展，以及相关的数字政府、数字社会建设，适用本条例。

　　本条例所称数字经济，是指以数据资源为关键生产要素，以现代信息网络为主要载体，以信息通信技术融合应用、全要素数字化转型为重要推动力，促进效率提升和经济结构优化的新经济形态。

　　第三条　发展数字经济是本省经济社会发展的重要战略，应当遵循优先发展、应用先导、数据驱动、创新引领、人才支撑、包容审慎以及保障数据安全、保护个人信息的原则。

　　第四条　县级以上人民政府应当加强对数字经济发展工作的领导，将数字经济发展纳入国民经济和社会发展规划，建立健全数字经济发展工作协调机制，统筹政策制定，督促检查政策落实，协调数字经济发展中的重大问题，并将数字经济发展相关指标纳入高质量发展绩效评价体系。

　　县级以上人民政府应当采取措施，鼓励和支持开展数字技术研发和推广应用，培育和发展数字经济新产业、新业态和新模式，加快建设与数字经济发展相适应的技术创新体系、产业生态体系、公共服务体系和现代治理体系，营造有利于数字经济发展的最优环境。

　　第五条　省经济和信息化主管部门负责推进、协调、督促全省数字经济发展工作。

　　设区的市、县（市、区）人民政府经济和信息化主管部门或者设区的市、县（市、区）人民政府确定的其他部门（以下统称数字经济主管部门），负责推进、协调、督促本行政区域内的数字经济发展工作。

　　县级以上人民政府数据发展主管部门或者设区的市、县（市、区）人民政府确定的其他部门（以下统称公共数据主管部门），负责组织、指导、协调公共数据管理和推进政府数字化转型工作。

　　县级以上人民政府其他有关部门按照各自职责做好促进数字经济发展工作。

　　第六条　省经济和信息化主管部门会同省有关部门编制省数字经济发展规划，报省人民政府批准后组织实施。

　　设区的市、县（市、区）数字经济主管部门会同同级有关部门根据省数字经济发展规划的要求和实际需要，编制本地区数字经济发展规划，报本级人民政府批准后组织实施。

　　第七条　省标准化主管部门应当会同省经济和信息化等有关部门推进本省数字经济标准体系建设，建立和完善基础通用标准、关键技术标准、融合应用标准和安全评估标准等各类数字经济标准，指导和支持有关单位采用先进的数字经济标准。

　　县级以上人民政府应当组织和支持行业协会、产业联盟、龙头企业等参与制定数字经济国际标准、国家标准、行业标准和地方标准。鼓励行业协会、产业联盟、龙头企业等自主制定数字经济团体标准或者企业标准。

　　县级以上人民政府标准化主管部门、有关行政主管部门应当对标准的实施情况开展监督检查。

　　第八条　省经济和信息化主管部门应当会同统计等有关部门建立健全数字经济统计监测指标体系和发展综合评价体系，制定和完善数字经济核心产业统计分类目录，依法实施日常统计和运行监测，开展数字经济年度发展评价，定期向社会公布主要统计指标、监测结果和发展综合评价指数。

　　第九条　县级以上人民政府应当加强数字经济领域国际交流合作，参与“一带一路”建设，增强数字经济的资源集聚和发展辐射能力。

　　县级以上人民政府应当按照长三角区域一体化发展等国家战略要求，加强数字经济发展跨省域合作，推动重大数字基础设施共建共享、公共数据标准统一、公共数据资源共享开放、智能制造协同发展，以及区域一体化协同治理和治理数字化应用。

　　县级以上人民政府应当加强省内数字经济跨区域合作，创新体制机制，加强政策协调，共同促进数字经济发展。

第二章　数字基础设施

　　第十条　本条例所称数字基础设施，是指以信息技术为支撑、以信息网络为基础，为经济、社会发展及居民生活提供感知、传输、存储、计算及融合应用等基础性信息服务的公共设施体系，主要包括信息网络基础设施、算力基础设施、新技术基础设施、融合基础设施、信息安全基础设施等。

　　第十一条　省经济和信息化主管部门应当会同有关部门，根据省数字经济发展规划的要求编制全省数字基础设施发展规划，按照省人民政府规定的权限报经批准后组织实施。

　　设区的市、县（市）人民政府数字经济主管部门应当会同有关部门，根据全省数字基础设施发展规划编制相关数字基础设施建设专项规划，报本级人民政府批准后组织实施。数字基础设施建设专项规划应当符合国土空间总体规划，并与市政、交通、电力、公共安全等相关基础设施专项规划相互协调和衔接。

　　编制、实施数字基础设施发展规划和建设专项规划应当遵循技术先进、适度超前、安全可靠、共建共享、避免重复、覆盖城乡、服务便捷的原则，重点推进新一代移动通信网、大数据中心、工业互联网、物联网、车联网、人工智能、区块链、卫星通信等新型数字基础设施建设，加快市政、交通、能源、电力、水利等传统基础设施的数字化改造。

　　县级以上人民政府应当探索建立跨行业基础设施“多规合一”体制机制，推动数字基础设施共建共享。

　　第十二条　县级以上人民政府及其有关部门、省通信管理机构，应当推进新一代移动通信网建设、光纤网络优化布局和互联网络演进升级，加强骨干网、城域网和接入网建设，提高网络容量、通信质量和传输速率。

　　县级以上人民政府及其有关部门、省通信管理机构，应当按照乡村振兴战略等要求，加强山区、海岛等地区网络基础设施建设，提升乡村光纤网络、移动网络建设水平和覆盖质量，实现农村电信普遍服务。

　　省发展改革、经济和信息化、自然资源等有关部门，应当按照陆海统筹、空天一体要求推动信息网络基础设施建设。

　　第十三条　县级以上人民政府及其有关部门、省通信管理机构，应当按照空间集聚、规模发展、技术先进、节能降耗的要求，加强高等级绿色数据中心建设和传统数据中心整合改造，推动云计算、边缘计算等多元计算协同发展，构建高效协同的数据处理体系。

　　第十四条　县级以上人民政府及其有关部门应当推动物联网技术发展，推进城乡基础设施、城乡治理、物流仓储、生产制造、生活服务等领域建设和应用感知系统，实现感知系统互联互通和数据共享。

　　第十五条　国土空间总体规划应当体现数字基础设施建设的相关要求，国土空间详细规划应当对数字基础设施建设专项规划确定的设施位置、空间布局等内容作出安排。

　　基础电信业务经营者应当按照数字基础设施建设专项规划的要求，科学合理做好基站、室内分布系统、多功能智能杆塔、汇聚机房布局。

　　第十六条　新建、扩建建设工程，根据国土空间详细规划需要配套建设数字基础设施的，国有建设用地使用权出让前，自然资源主管部门应当将配套建设数字基础设施的要求纳入规划条件。

　　新建、扩建建设工程，根据国土空间详细规划需要配套建设数字基础设施的，建设单位应当按照国家和省有关标准预留基站站址，配套建设机房、管道、电力线路、电器装置、防雷、接地等通信基础设施，并与主体工程同步设计、同步施工、同步验收。老旧小区改造应当配套建设前述通信基础设施。

　　建设工程移动通信基础设施建设标准，由省住房城乡建设主管部门会同省通信管理机构及有关单位制定。

　　第十七条　公共机构以及公共场所、公共设施的所有者、管理者或者使用者应当支持通信设施建设，按照国家和省有关规定开放建筑物、绿地、杆塔等资源，推进智慧杆塔建设和一杆多用。禁止收取进场费、分摊费等不合理费用。

第三章　数据资源

　　第十八条　数据资源管理应当遵循依法规范、促进流通、合理使用、保障安全的原则，加强数据资源全生命周期管理，提升数据要素质量，培育发展数据要素市场，促进大数据开发利用和产业发展，推进治理工作数字化。

　　本条例所称数据资源，是指以电子化形式记录和保存的具备原始性、可机器读取、可供社会化再利用的数据集合，包括公共数据和非公共数据。

　　本条例所称公共数据，是指国家机关、法律法规规章授权的具有管理公共事务职能的组织（以下统称公共管理和服务机构）在依法履行职责和提供公共服务过程中获取的数据资源，以及法律、法规规定纳入公共数据管理的其他数据资源。

　　第十九条　任何单位和个人收集、存储、使用、加工、传输、提供、公开数据资源，应当遵循合法、正当、必要的原则，遵守网络安全、数据安全、电子商务、个人信息保护等有关法律、法规以及国家标准的强制性要求，不得损害国家利益、社会公共利益或者他人合法权益。

　　第二十条　公共数据应当按照规定在公共管理和服务机构之间实现共享或者协同应用。通过共享获得的公共数据，应当用于履行本机构职责，不得用于其他目的。

　　公共管理和服务机构应当按照需求导向、分类分级、统一标准、安全可控、便捷高效的原则向社会开放公共数据。鼓励使用公共数据从事科学技术研究、咨询服务、产品开发、数据加工等活动。

　　公共数据采集单位对所采集数据的真实性、准确性、完整性负责。公共数据主管部门发现公共数据不准确、不完整或者不同采集单位提供的数据不一致的，可以要求采集单位限期核实、更正。采集单位应当在要求的期限内核实、更正。

　　公共数据共享和开放的具体办法，按照国家和省有关规定执行。

　　第二十一条　县级以上人民政府及其有关部门应当通过产业政策引导、社会资本引入、应用模式创新、强化合作交流等方式，引导企业、社会组织等单位和个人开放自有数据资源。

　　鼓励企业、社会组织等单位和个人通过省、设区的市公共数据平台，对外提供各类数据服务或者数据产品。

　　第二十二条　县级以上人民政府及其有关部门应当坚持保障安全与发展数字经济并重的原则，建立健全网络安全、数据安全保障体系，完善协调机制以及安全预警、安全处置机制。

　　县级以上人民政府有关部门应当加强对个人信息数据收集、存储、使用、加工、传输、提供、公开等活动的监督管理，依法查处个人信息数据泄露、窃取、篡改、非法使用等危害个人信息数据安全的违法活动。

　　网络运营者等有关单位和个人，应当依法建立健全数据安全管理制度，采取相应技术措施和其他必要措施，保障数据安全。

第四章　数字产业化

　　第二十三条　本条例所称数字产业化，是指现代信息技术通过市场化应用，形成电子信息制造业、软件和信息技术服务业、电信广播卫星传输服务业和互联网服务业等数字产业。

　　第二十四条　省人民政府应当根据全球数字经济的技术、产业发展趋势，结合本省数字产业发展水平和各地区经济禀赋差异，统筹规划全省数字产业发展，通过提升产业链、保障供应链安全、培育产业集群等方式，促进产业协同创新和供应保障，提高数字产业整体竞争力。

　　第二十五条　县级以上人民政府及其有关部门应当按照全省数字产业发展要求，结合本地区实际，通过规划引导、政策支持、市场主体培育等方式，重点推动集成电路、高端软件、数字安防、网络通信、智能计算、新型显示、新型元器件及材料、网络安全等产业发展，促进云计算、大数据、物联网、人工智能等技术与各产业深度融合，培育区块链、量子信息、柔性电子、虚拟现实等产业发展。

　　第二十六条　省人民政府及其有关部门应当推动国家和省实验室、重点实验室、技术创新中心、制造业创新中心、企业技术中心等科技创新平台和大型科技基础设施建设，支持科研机构、高等院校、企业参与建设有关平台和设施。

　　利用财政性资金或者国有资本购置、建设大型科学仪器设施的，应当在保障安全规范的前提下，为科研机构、高等院校、企业等开展创新活动提供共享服务。省科技主管部门应当建立大型科学仪器开放共享平台，为仪器设施共享提供信息发布、使用预约等服务。

　　鼓励、支持企业加强信息技术和产品研发，加大资金投入，加强人才引进和储备，培育研发机构，提升研发能力。

　　县级以上人民政府及其科技等部门应当培育和发展数字产业技术交易市场，促进技术转让、创新成果转化和产业化。

　　市场监督管理部门、司法机关等应当完善知识产权领域的区域和部门协作机制，建立健全知识产权快速维权体系，提供境内外知识产权维权援助。

　　第二十七条　县级以上人民政府及其有关部门应当采取措施，培育多层次、递进式的数字产业企业梯队，形成大中小微企业协同共生的数字经济产业生态。

　　鼓励和支持企业、科研机构、高等院校及其他单位或者个人创建数字经济领域科技企业孵化器、大学科技园和众创空间等线上线下创新创业平台。

　　鼓励提供数字产业化服务的第三方机构，为数字产业相关企业引进落地、融资增资、股改上市、平台化转型、跨境并购和合作等提供服务，推动数字产业发展。

第五章　产业数字化

　　第二十八条　本条例所称产业数字化，是指利用现代信息技术对工业、农业、服务业等产业进行全方位、全角度、全链条改造，提高全要素生产率，实现工业、农业、服务业等产业的数字化、网络化、智能化。

　　第二十九条　县级以上人民政府经济和信息化主管部门应当推动企业实施制造装备、生产线、车间、工厂的智能化改造和产品智能化升级，推进网络化协同、个性化定制、柔性化生产、共享制造等智能制造和服务型制造。

　　县级以上人民政府应当通过服务指导、试点示范、政策支持等方式，加大对工业互联网发展的支持力度，推进行业级、产业链级、区域级、企业级等工业互联网平台建设及应用，推动工业技术软件化，促进大型企业开展研发设计、生产加工、经营管理、销售服务等集成创新，降低中小企业使用工业互联网成本，推动中小企业普及应用工业互联网。

　　鼓励和支持企业主动上云、深度用云，提升生产和管理效能。

　　第三十条　县级以上人民政府及其有关部门应当推进旅游、健康、家庭、养老、教育等生活性服务业数字化，推动数字技术和生活性服务业深度融合，丰富服务产品供给，促进生活消费方式升级。

　　县级以上人民政府及其有关部门应当通过培育服务业数字化转型试点等方式，推进研发设计、现代物流、检验检测服务、法律服务、商务咨询、人力资源服务等生产性服务业数字化，提升生产性服务业智能化、网络化、专业化水平。

　　县级以上人民政府及其有关部门应当通过建设数字文化创意产业试验区等方式，推进网络视听、数字影视、数字动漫、网络游戏、数字广告、互动新媒体等数字文化创意产业发展。

　　第三十一条　县级以上人民政府及其有关部门应当通过示范带动、技术指导、政策支持等方式，推广农业物联网应用，加快农业生产、农产品加工、农产品流通领域大数据基础和应用平台建设，加大农村仓储、物流、冷链设施建设支持力度，提升农业农村数字化、网络化、智能化改造和应用水平。

　　第三十二条　县级以上人民政府及其有关部门应当推进移动支付在全省域范围内的普及应用，有关国家机关、企事业单位、社会组织履行公共管理和公共服务职能时，应当推广应用移动支付，并鼓励市场主体应用移动支付。

　　省地方金融监督管理部门应当会同人民银行、银行保险监管、证券监管等有关机构制定相关政策，引导和支持现代信息技术在支付结算、信贷融资、保险业务、征信服务等金融领域融合应用，推动金融业数字化发展。

　　第三十三条　县级以上人民政府及其有关部门应当制定相关政策，引导和支持电子商务发展，促进跨境电商综合试验区建设，提升跨境电商普及应用水平，推广新零售，发展电子商务新业态新模式，推进数字生活新服务。

　　第三十四条　县级以上人民政府及其有关部门应当通过政策支持、市场主体培育等方式，促进互联网平台经济发展，推动建设产业互联网平台，完善工业、农业、服务业等互联网平台经济支撑体系，促进产业优化升级。

　　鼓励和支持工业信息工程企业、科研机构、高等院校及其他主体提供产业数字化转型第三方服务，加强对产业数字化转型的技术支撑保障，推动产业数字化转型。

　　鼓励互联网平台、提供产业数字化转型服务机构与中小微企业建立对接机制，针对不同行业的中小微企业需求场景提供数字化解决方案。

　　第三十五条　县级以上人民政府及其有关部门应当完善开发区（高新区）、小微企业园、农业产业园等各类园区的数字基础设施，提升园区数字化管理服务功能，加强现代信息技术在园区的融合应用，支撑园区内企业数字化转型和数字产业集聚发展。

第六章　治理数字化

　　第三十六条　本条例所称治理数字化，是指在政治、经济、文化、社会、生态文明等领域，运用现代信息技术，实现治理机制、方式和手段的数字化、网络化、智能化，推进治理体系和治理能力现代化。

　　县级以上人民政府应当推动建立政府监管、平台自治、行业自律、公众参与的多元共治体系，通过治理数字化促进数字经济发展。

　　第三十七条　县级以上人民政府及其有关部门应当深化“最多跑一次”改革，按照整体智治的要求，推动数字技术与政府履职全面深度融合，推进政务服务、政府办公全流程网上办理、掌上办理，实现数据共享和业务协同，推进政府数字化转型。

　　前款规定以外的公共管理和服务机构，应当按照高水平推进省域治理现代化的要求，加强数字化建设和应用，提升治理效能。

　　第三十八条　省人民政府及其有关部门应当完善并规范使用全省统一的行政处罚办案系统，推进简易处罚的掌上办理，完善行政执法监管系统功能，推动行政执法与刑事司法衔接。

　　行政机关按照行政执法证据的要求进行采集、固定并经审核确认的电子监测记录，可以作为行政执法的证据。

　　鼓励有关部门依托物联网、区块链等技术，在教育、医疗、交通、邮政、生态环境保护、药品监管、工程建设、公共安全等重点领域推行监管智能化应用。

　　第三十九条　省、设区的市人民政府应当统筹规划和推进公共数据平台建设，实现基础设施、数据资源和公共应用支撑体系共建共享。

　　县级以上人民政府应当推进经济调节、市场监管、社会治理、生态环境保护、政府自身运行等领域的数字化应用体系建设，逐步实现政府履职全业务、全流程数字化，提高政府科学决策、高效监管、精准治理水平。

　　第四十条　县级以上人民政府及其有关部门应当按照省有关规定加强智慧城市建设，依托省、设区的市公共数据平台，推动“城市大脑”应用推广，促进现代信息技术在城市交通、平安建设、医疗健康、生态环境保护、文化旅游等领域的综合应用，通过数据资源整合共享，实现城市运行态势监测、公共资源配置、宏观决策、统一指挥调度和事件分拨处置数字化，提升城市治理水平。

　　县级以上人民政府应当加强乡村数字基础设施建设，促进现代信息技术在乡村产业发展、公共服务、农村集体资产管理等领域的综合应用，提升乡村治理水平。

　　第四十一条　县级以上人民政府及其教育主管部门应当加强教育领域数字基础设施和数字校园建设，加快数字技术与教育管理、教育教学的深度融合，采取措施支持符合条件的各类主体规范发展在线教育，培育优质数字教育资源。

　　县级以上人民政府及其卫生健康、医疗保障等有关部门应当加强智慧医疗健康体系建设，推广电子凭证、电子病历、电子处方、电子票据的应用，实行医疗检验、检查信息共享，拓展医疗保障数字化平台便民应用。

　　省人民政府及其民政部门应当加强智慧养老体系建设，建立全省统一的智慧养老服务平台。民政部门应当通过智慧养老服务平台，为各类用户提供简便快捷的养老政务服务、公共服务和链接市场服务。

　　第四十二条　县级以上人民政府及其有关部门应当按照省有关规定统筹规划和推进社会治理数字化转型，强化综合治理工作平台、市场监管平台、综合执法平台、便民服务平台等基层治理平台建设和运营管理，提高社会治理社会化、智能化和专业化水平。

　　县级以上人民政府及其有关部门应当按照省有关规定，开展未来社区示范建设，以数字技术提升精细化、网络化管理能力，构建未来邻里、教育、健康、创业等数字化创新应用场景。

　　第四十三条　县级以上人民政府及其有关部门应当创新监管理念和方式，对数字经济领域的新技术、新产业、新业态和新模式实行包容审慎监管。

　　省公安机关、市场监督管理等部门应当会同有关部门建立数字经济领域跨区域异地执法协调机制，实现违法线索互联、监管标准互通。

　　市场监督管理部门应当按照国家和省有关规定和要求，加强数字市场竞争监管，发挥行业协会、产业联盟和其他组织作用，维护公平竞争秩序。从事数字经济活动的单位和个人不得有滥用市场支配地位、实施垄断协议等行为以及从事不正当竞争活动。

　　第四十四条　互联网平台经营者应当依法依约履行产品和服务质量保障、消费者权益保护、生态环境保护、知识产权保护、网络安全与个人信息保护、劳动者权益保护等方面的义务，建立健全平台规则和用户账号信用管理、投诉举报等制度。鼓励互联网平台经营者建立争议在线解决机制，制定并公示争议解决规则。

　　省人民政府及其有关部门应当组织建设网络交易监测平台。有关部门应当通过网络交易监测平台，对互联网平台经营者以及网络交易违法行为实施在线监测，实现网络交易的风险预警、协同监管、电子存证。

　　第四十五条　鼓励和支持行业协会、产业联盟和其他组织在数字经济发展中发挥技术指导和服务作用。县级以上人民政府及其有关部门可以通过向行业协会等组织购买服务等方式，开展技术推广、职业技能培训和咨询服务。

　　数字经济行业协会、产业联盟和其他组织应当依法依规开展活动，加强自律管理，开展纠纷处理和信用评价，反映合理诉求，依法维护企业合法权益。

　　鼓励和支持企业、第三方机构和社会公众参与数字经济治理。

　　第四十六条　县级以上人民政府及其有关部门应当按照优化传统服务与创新数字服务并行的原则，制定和完善老年人等运用智能技术困难群体在出行、就医、消费、文娱、办事等方面的服务保障措施，保障和改善运用智能技术困难群体的基本服务需求和服务体验。

第七章　激励和保障措施

　　第四十七条　省人民政府设立数字经济产业投资基金，用于数字经济领域重大项目建设。

　　县级以上人民政府应当完善产业投资基金投资融资机制，引导社会资本投资数字经济领域重大项目，拓宽数字经济企业融资渠道。

　　第四十八条　省人民政府及其有关部门应当将数字经济重大科技攻关项目的自主创新研究、应用示范和产业化发展列入国家或者省科技发展规划、高新技术产业发展规划，并安排财政性资金予以支持。

　　县级以上人民政府及其有关部门应当根据省有关规定并结合实际，安排财政性资金支持数字产业化发展、产业数字化转型以及数字经济企业培育等，引导和支持社会资本参与数字经济发展。

　　第四十九条　省人民政府及其有关部门应当加强产业链协同创新统筹协调，引导和支持科研机构、高等院校、企业加强协同攻关，共同开展数字经济基础前沿研究和关键共性技术研究。

　　县级以上人民政府科技主管部门可以通过向企业和创业者发放科技创新券的方式，支持数字经济产业科技创新和科技成果转化。科技创新券可以用于购买科技成果、检验检测、研究开发设计、中间试验、科技评估、技术查新、知识产权服务、技术培训等服务。科技创新券在全省范围内使用。推动科技创新券在长三角地区通用通兑。

　　第五十条　省人民政府或者其授权的单位可以根据需要，将云计算、大数据、人工智能等数字技术产品和服务列入全省集中采购目录。

　　政府采购的采购人经依法批准，可以通过非公开招标方式，采购达到公开招标限额标准的首台（套）装备、首批次产品、首版次软件，支持数字技术产品和服务的应用推广。

　　第五十一条　县级以上人民政府及其有关部门应当落实国家和省对高新技术企业研发、信息技术产品制造、软件开发、信息服务以及科技企业孵化器、大学科技园和众创空间等线上线下创新创业平台的税费优惠，并为相关单位和个人办理税费优惠提供便利。

　　第五十二条　本省实行有利于数字经济发展的金融政策，对符合国家和省数字经济产业政策的项目、企业、园区、平台和创新人才，金融机构、地方金融组织应当在贷款、政策性融资担保以及其他金融服务等方面给予支持。

　　鼓励银行业金融机构适应产业数字化和数字产业化需求，创新金融服务，开发融资产品，提高信用贷款、中长期贷款等产品的比重，提供无还本续贷、循环贷款或者其他创新型续贷产品。

　　支持保险业金融机构为符合国家和省数字经济产业政策的项目、企业贷款提供保证保险和信用保险。县级以上人民政府可以安排资金用于保证保险和信用保险的风险补偿等。

　　鼓励数字经济企业通过股权投资、股票债券发行等方式融资，提高直接融资比例，改善融资结构。

　　第五十三条　县级以上人民政府及其有关部门应当完善政策措施，强化创新服务，在土地供应、电力接引、能耗指标、频谱资源等方面优先保障数字经济发展。

　　第五十四条　县级以上人民政府及其有关部门应当支持举办数字经济领域的国内国际展览、赛事、论坛等活动，搭建数字经济展示、交易、交流、合作平台，帮助建立供需对接渠道，提高企业市场开拓能力。

　　县级以上人民政府及其有关部门应当支持数字经济领域企业参加境内外展览展销等活动。

　　第五十五条　县级以上人民政府及其有关部门应当制定扶持政策，加强数字经济领域关键核心技术人才培养，将数字经济领域引进高层次、高学历、高技能以及紧缺人才纳入政府人才支持政策体系，为其在职称评定、住房、落户、医疗保健，以及配偶就业、子女入学等方面提供支持。

　　教育、人力资源社会保障等部门应当指导和督促高等院校、职业学校开设数字经济专业、课程，培养数字经济研究和应用型人才。

　　高等院校、科研机构、职业学校等应当通过与企业产学研合作、共建实习实训基地等方式，培养符合数字经济发展需求的相关人才。

　　第五十六条　县级以上人民政府及其有关部门应当加强数字经济法律、法规、规章以及技术、知识的宣传、教育、培训，提升全民数字素养和数字技能。

　　教育、人力资源社会保障等部门应当指导和督促学校及其他教育机构将数字经济知识纳入教育教学内容，公务员主管部门应当将数字经济知识纳入公务员教育培训内容。

　　广播、电视、报刊、互联网等新闻媒体应当开展数字经济公益性宣传。鼓励社会团体、企事业单位加强员工数字经济知识培训，提升应用、管理和服务水平。

　　第五十七条　县级以上人民政府人力资源社会保障主管部门应当按照省有关规定，加强对数字经济新业态用工服务的指导，积极探索灵活多样的用工方式，制定和完善数字经济新业态从业人员在工作时间、报酬支付、保险保障等方面规定，保障数字经济新业态从业人员的合法权益。

　　数字经济新业态从业人员通过互联网平台注册并接单，提供网约车、外卖或者快递等劳务的，平台经营者可以通过单险种参加工伤保险的形式为从业人员提供工伤保险待遇。平台经营者单险种参加工伤保险的，社会保险经办机构应当予以办理。法律、行政法规另有规定的，从其规定。

　　第五十八条　促进数字经济发展工作中出现失误，但同时符合下列条件的，对有关单位和个人不作负面评价：

　　（一）符合国家和省确定的改革方向；

　　（二）未违反法律、法规禁止性、义务性规定；

　　（三）决策程序符合法律、法规规定；

　　（四）勤勉尽责、未牟取私利；

　　（五）主动挽回损失、消除不良影响或者有效阻止危害结果发生。

第八章　法律责任

　　第五十九条　违反本条例规定的行为，法律、行政法规已有法律责任规定的，从其规定。

　　第六十条　建设单位违反本条例第十六条第二款规定，新建、扩建建设工程未按国家和省有关标准预留基站站址或者配套建设机房、管道、电力线路、电器装置、防雷、接地等通信基础设施的，由县级以上人民政府住房城乡建设主管部门责令限期改正；逾期不改正的，处五万元以上二十万元以下罚款。

　　第六十一条　县级以上人民政府有关部门、单位及其工作人员在促进数字经济发展中有下列行为之一的，由有权机关按照法定职责责令改正；情节严重的，对直接负责的主管人员和其他直接责任人员依法给予处分：

　　（一）篡改、伪造或者指使篡改、伪造数字经济主要统计指标的；

　　（二）未按规定履行公共数据共享和开放职责的；

　　（三）未按规定核实、更正公共数据的；

　　（四）其他玩忽职守、滥用职权、徇私舞弊的行为。

第九章　附则

第六十二条　本条例自2021年3月1日起施行。《浙江省信息化促进条例》同时废止。

(11)河南省数字经济促进条例

2021年12月28日河南省第十三届人民代表大会常务委员会第二十九次会议通过

第一章　总则

　　第一条　为了促进数字经济发展，全面建设数字经济强省，推动经济社会高质量发展，根据有关法律、行政法规，结合本省实际，制定本条例。

　　第二条　本省行政区域内促进数字经济发展、保障数字经济安全等相关活动适用本条例。

　　本条例所称数字经济，是指以数据资源为关键生产要素，以现代信息网络为重要载体，以数字技术促进效率提升和结构优化的经济形态。

　　第三条　发展数字经济是本省经济社会发展的重要战略。发展数字经济应当遵循统筹规划、市场主导、创新引领、共建共享、包容审慎、数据安全的原则。

　　第四条　县级以上人民政府应当将数字经济发展纳入国民经济和社会发展规划，加大对数字经济发展的投入，促进数字经济高质量发展。

　　县级以上人民政府应当加强对数字经济促进工作的领导，建立统筹协调机制，解决数字经济发展中的重大问题。

　　第五条　县级以上人民政府发展改革部门是数字经济的主管部门，负责组织、协调、推进数字经济发展工作。

　　县级以上人民政府工业和信息化部门负责电子信息产业和软件服务业管理工作，拟定并组织实施相关规划、政策和标准，促进数字经济发展。

　　县级以上人民政府教育、科技、公安、民政、财政、人力资源社会保障、自然资源、生态环境、住房城乡建设、交通运输、水利、农业农村、商务、文化和旅游、卫生健康、应急、市场监管、统计、大数据、能源以及网信、通信管理等有关部门应当按照职责分工，做好促进数字经济发展相关工作。

　　第六条　县级以上人民政府数字经济主管部门应当会同有关部门，根据上级人民政府数字经济发展规划，结合本地实际，编制本行政区域数字经济发展规划，报同级人民政府批准后实施。

　　第七条　省人民政府及有关部门应当推动数字经济开放合作，加强与有关国家和地区在数字基础设施建设、电子商务、数字贸易等领域的合作交流；加强与省外数字经济合作，促进基础设施共建共享，数据资源依法有序流动、合法有效利用，数字产业协同发展。

　　第八条　鼓励和支持各类市场主体在合法、安全的前提下，参与数据资源开发利用、数字产业化发展、产业数字化转型及数字化治理和服务。

　　支持高等院校、科研机构、行业协会等为促进数字经济发展提供人才培养、创业孵化、投资融资、技术支持、产权交易等服务。

　　第九条　县级以上人民政府及有关部门、新闻媒体应当加强数字经济领域相关法律、法规、政策和知识的宣传普及，营造促进数字经济发展的良好氛围。

　　第十条　县级以上人民政府应当对在数字经济发展工作中作出突出贡献的单位和个人，按照有关规定给予表彰和奖励。

第二章　数字基础设施建设

　　第十一条　本条例所称数字基础设施，是指以信息技术为支撑、以信息网络为基础，为经济社会发展提供感知、传输、存储、计算及融合应用等基础性信息服务的公共设施体系，主要包括通信网络基础设施、算力基础设施、新技术基础设施、融合基础设施和信息安全基础设施等。

　　数字基础设施建设应当遵循安全可靠、自主可控的原则。

　　第十二条　县级以上人民政府应当将数字基础设施的建设和布局纳入国土空间规划，在制定市政、交通、电力、公共安全等相关基础设施规划时应当根据数字经济发展的需要，与数字基础设施建设规划统筹衔接。

　　第十三条　县级以上人民政府及有关部门、省通信管理部门应当支持新一代移动通信网络建设、光纤宽带网络优化布局和卫星互联网络、量子通信网络发展，推进互联网骨干网、城域网、接入网等信息通信网络建设。

　　工程建设、设计等单位应当将信息通信网络基础设施作为主体工程的重要组成部分，按照有关建设设计标准和规范同时设计、同时施工、同时验收，并保留完整的管线分布等数字基础设施建设档案。

　　第十四条　省人民政府及有关部门应当统筹推进数据中心、智能计算中心、超级计算中心等基础设施建设；支持数据中心优化建设和升级改造，提升资源利用水平和运行效率；推动云计算、边缘计算等多元计算协同发展，构建高效协同的数据处理体系。

　　设区的市、县级人民政府可以根据需要，按照国家产业政策统筹建设本区域数据中心、边缘计算中心等算力基础设施，在土地、电力保障等方面给予支持。

　　第十五条　省人民政府及有关部门应当统筹推进人工智能、区块链等新技术基础设施建设，支持建设底层技术平台、算法平台、开源社区等基础平台，建立领先的新技术能力支撑体系。

　　鼓励社会力量参与数字经济新技术基础设施建设。政府可以通过购买服务等方式，发挥基础平台作用，提供公共服务。

　　第十六条　县级以上人民政府及有关部门应当推动能源、交通、城建、农业、水利、环保、应急、医疗、健康、教育、文化和旅游等领域的传统基础设施数字化、网络化、智能化改造，建立经济社会智慧化运行的基础设施体系。

　　省人民政府工业和信息化部门、省通信管理部门应当推动工业互联网基础设施建设，完善工业互联网标识解析体系，支持建设多层次工业互联网平台体系，加强工业互联网安全能力建设，推动企业积极开展内外网升级改造，提升生产和管理效能。

　　县级以上人民政府及有关部门应当加快交通、物流、市政等重点领域物联网终端和智能传感器的规模部署，推动感知设备统一接入、集中管理和感知数据共享利用。

　　县级以上人民政府及有关部门应当加强乡村数字基础设施建设，推动乡村信息服务供给和基础设施数字化转型，完善农村电商基础设施，建立健全农产品网络销售的物流设施、供应链设施和支撑保障设施，促进乡村振兴战略实施。

　　第十七条　县级以上人民政府及有关部门应当构建完善云网数端一体化协同安全保障体系，运用可信身份认证、数字签名、接口鉴权、数据溯源等数据保护措施和区块链等技术，强化对数据资源和算力资源的安全防护。

　　县级以上人民政府及有关部门应当推动企业和第三方机构创新云安全服务模式，强化数据安全技术服务能力。

　　数字基础设施以及网络平台的管理者、运营者应当加强对关键信息基础设施和数据资源的安全保护。

第三章　数据资源开发利用

　　第十八条　本条例所称数据资源，是指以电子化形式记录、保存的，可以通过云计算、大数据、人工智能等技术分析处理，并可供社会化再利用的各类信息资源的集合，包括公共数据资源和非公共数据资源。

　　第十九条　公共数据资源，是指国家机关、法律法规授权的具有管理公共事务职能的组织，在依法履行公共管理和服务职责过程中形成的数据资源。

　　非公共数据资源，是指公共数据资源以外的数据资源。

　　第二十条　数据资源开发利用应当遵循依法规范、促进流通、合理使用、保障安全的原则。

　　省人民政府应当按照国家规定制定数据资源开发利用管理办法，提升数据要素质量，培育发展数据要素市场，促进数据资源开发利用和产业化发展。

　　第二十一条　数据资源开发利用应当遵守相关法律、法规，保障信息主体合法权益，保护国家数据资源安全。

　　数据资源开发利用应当履行数据安全保护义务，尊重社会公德和伦理，尊重数据隐私，遵守商业道德和职业道德，诚实守信，承担社会责任。

　　第二十二条　数据资源开发者对其开发的数字技术和数据产品依法享有知识产权，任何单位和个人不得非法侵占、使用。

　　第二十三条　公共数据应当依法共享，法律、行政法规规定不予共享的情形除外。公共数据提供单位应当按照需求导向、分类分级、统一标准、安全可控、便捷高效的原则共享开放公共数据，注明数据共享的条件和方式，并按照规定逐步扩大公共数据开放范围。

　　鼓励使用公共数据从事科学技术研究、咨询服务、产品开发、数据加工等活动。

　　第二十四条　鼓励自然人、法人和非法人组织依法开放非公共数据，促进数据融合创新。

　　数据资源拥有者对其汇集的非公共数据资源依法享有使用权，但是不得侵害信息主体的合法权益。

　　数据信息主体对其个人数据依法享有知情权、同意权、查阅权、复制权、更正权、撤回权和可携带权。

　　第二十五条　省人民政府及其有关部门应当支持数据资源开发市场化发展，创新数据交易模式，拓宽数据交易渠道，促进数据高效流通；鼓励省内高等院校、科研机构及数据运营单位研究建立数据价值评估和定价模式；支持有条件的地区依法设立数据交易中心；鼓励和引导数据供需双方依法进行数据产品交易。

　　县级以上人民政府及其有关部门应当规范数据交易行为，做好流转动态管理，按照包容审慎的原则建立完善数据资源交易监管体制。

第四章　数字产业化发展

　　第二十六条　本条例所称数字产业化，是指通过数字技术的市场化应用，将数字化的知识和信息转化为生产要素，推动数字产业的形成和发展。数字产业包括数字产品制造业、数字产品服务业、数字技术应用业和数字要素驱动业等。

　　第二十七条　省人民政府及发展改革、科技、工业和信息化等有关部门应当根据数字经济技术、产业发展趋势，结合本省产业发展水平和各地区经济禀赋差异，统筹规划全省数字产业化发展。

　　县级以上人民政府及有关部门应当结合本地实际，规划本行政区域的数字产业化发展，做强电子信息制造业、软件和信息技术服务业，重点培育新型显示和智能终端、物联网、软件、网络安全、先进计算、网络通信、集成电路、人工智能、区块链等数字产业集群。

　　第二十八条　省人民政府及发展改革、科技、工业和信息化等有关部门应当统筹新型显示和智能终端产业发展，支持设立研发创新平台，强化基金和科研支撑能力，提升新型显示和智能终端产业发展水平。

　　第二十九条　省人民政府及工业和信息化、发展改革、通信管理等有关部门应当统筹物联网产业发展，完善智能传感器、射频卡、嵌入式芯片、传感网络设备等物联网产业链，构建自主可控的信息感知、网络传输、平台建设、应用示范生态体系。

　　第三十条　省人民政府及工业和信息化、发展改革、科技等有关部门应当统筹软件产业发展，支持高水平软件产业园区建设，培育具有自主知识产权的网络安全、工业基础、地理信息等软件产业，推进软件产品迭代、适配测试和产业化应用，构建自主可控、共建共享的软件产业生态。

　　第三十一条　省人民政府及工业和信息化、发展改革、科技等有关部门应当统筹电子信息制造业发展，做好重大项目推进、产业链上下游对接配套、龙头骨干企业培育，打造电子信息制造优势产业集群。

　　第三十二条　县级以上人民政府数字经济主管部门应当会同有关部门，坚持发展和规范并重，积极培育数字经济新业态和新模式，支持互联网平台企业发展，探索适宜本地的平台经济发展场景和模式，促进平台经济规范健康发展。

　　第三十三条　省人民政府及有关部门应当推动省实验室和省级以上工程研究中心、技术创新中心、重点实验室、制造业创新中心、企业技术中心、产业研究院等创新基地及平台建设。鼓励和支持企业、高等院校、科研机构等主体整合创新要素资源，围绕重点领域关键环节实施科技攻关，推动制造设备、基础器件、高端芯片、关键材料、软件等核心技术突破创新。

　　第三十四条　县级以上人民政府及有关部门应当结合本地实际，引导和支持建设数字经济园区，促进数字经济核心产业集聚发展，打造具有特色竞争优势的产业集群。

　　第三十五条　县级以上人民政府及其有关部门应当引导和支持数字经济核心产业的龙头企业、专精特新中小企业等市场主体的发展，培育多层次的企业梯队。

　　第三十六条　县级以上人民政府及其有关部门应当发挥数据资源和市场优势，鼓励本地企业、高等院校、科研机构加强国内外科技交流，开展数字经济核心产业关键技术和产品的研发合作。

第五章　产业数字化转型

　　第三十七条　本条例所称产业数字化，是指应用数字技术和数据资源为传统产业带来的产出增加和效率提升，是数字技术与实体经济的融合，包括智慧农业、智能制造、智能建造、智慧物流、智慧文旅、数字金融、数字商贸等数字化应用。

　　第三十八条　县级以上人民政府农业农村部门应当推进网络通信、物联网、云计算、大数据、人工智能、区块链等数字技术在农业生产、经营、管理、服务等方面的创新应用，支持智慧农（牧）场、农产品仓储保鲜冷链物流设施信息化、农业生产服务信息网络平台等建设，推广智能农机，推进精准种植养殖，提升农业数字化、智能化、精细化水平。

　　第三十九条　县级以上人民政府工业和信息化部门应当重点支持智能制造、服务型制造，加快工业互联网融合应用，推动制造企业实施制造单元、生产线、车间、工厂的智能化改造和产品智能化升级，推行平台化设计、数字化管理、智能化制造、个性化定制、网络化协同、服务化延伸等基于数字技术的制造业新业态、新模式。

　　第四十条　县级以上人民政府及其有关部门应当推进健康、养老、家政、文化和旅游等生活性服务业数字化，推动线上线下深度融合，丰富服务产品供给方式，提高生活消费便利化水平。

　　县级以上人民政府及有关部门应当推进现代物流、研发设计、检验检测服务、法律服务、评估认证、人力资源服务等生产性服务业数字化，提高生产性服务业智能化、专业化、精细化水平。

　　第四十一条　县级以上人民政府住房城乡建设部门应当大力发展数字设计、智能生产和智能施工，打造建筑产业互联网，形成全产业链融合一体的智能建造产业体系。

　　第四十二条　县级以上人民政府发展改革部门应当支持物流园区、货物管理、运输服务、场站设施等数字化升级，推广数字化技术和智能终端设备应用，提升物流智能化水平。

　　第四十三条　县级以上人民政府商务、工业和信息化等有关部门应当引导和支持电子商务平台、电子商务服务体系发展，促进跨境电商综合试验区、跨境电子商务产业园、公共海外仓等建设，支持工业、农业、物流、商务等领域的垂直电商平台建设，培育社交电商、直播电商等业态和模式。

　　第四十四条　省人民政府地方金融监督管理部门应当推动发展数字金融，优化移动支付应用，推进数字金融与产业链、供应链融合发展。

　　第四十五条　县级以上人民政府及有关部门应当通过财税支持、政府购买服务等方式，鼓励中小微企业平台、产业互联网平台、产业数字化转型服务机构与中小微企业建立对接机制，针对不同行业的中小微企业需求场景提供数字化解决方案，加强对产业数字化转型的技术、资金支撑保障，推动产业数字化转型。

　　第四十六条　县级以上人民政府及其有关部门应当提升各类开发区的数字化管理服务功能，加强数字技术融合应用，支持企业数字化转型发展。

第六章　数字化治理和服务

　　第四十七条　县级以上人民政府应当应用数字技术，推进数字政府、数字社会、新型智慧城市、数字乡村建设，提升公共服务数字化、智能化水平，推进治理体系和治理能力现代化，促进数字经济高质量发展。

　　第四十八条　省人民政府应当遵循集约便捷高效的原则，统筹数字政府建设，提升数字化治理能力。

　　县级以上人民政府及有关部门应当落实数字政府建设的要求，推进数字政府基础设施、公共支撑、数据服务、应用系统等集约化、一体化建设和运行，提升政府决策科学化、社会治理精准化、公共服务高效化、行政管理协同化水平。

　　第四十九条　县级以上人民政府应当落实新型智慧城市建设要求，按照新型智慧城市的总体架构，以需求为导向，根据城市规模和发展特点，推动城市治理、民生服务、生态宜居、产业发展等智能化创新应用，提升城市综合承载力、创造力、竞争力。

　　第五十条　县级以上人民政府应当促进数字技术在乡村产业发展、公共服务、集体资产管理等领域的综合应用，推进城市资源向乡村延伸，提升乡村治理水平。

　　第五十一条　县级以上人民政府应当强化社区服务和管理功能综合集成，推动标准化、规范化智慧小区建设，打造一体化智慧社区。

　　第五十二条　县级以上人民政府及有关部门应当加强信息资源深度整合应用，运用数字技术创新治理和服务模式，加强智慧交通、智慧医疗、智慧康养、智慧教育、智慧城管、智慧安防、智慧生态环境监控等建设，提升公共服务水平。

第七章　数字经济促进措施

　　第五十三条　省人民政府应当统筹各类财政专项资金、政府引导基金，重点用于支持数字经济领域关键核心技术攻关、重大创新平台和产业载体建设、典型示范应用和重大项目建设等。

　　设区的市、县级人民政府应当根据实际情况，统筹财政资金支持数字经济发展，完善投资融资机制，拓宽数字经济企业融资渠道，引导社会资本参与数字经济领域重大项目建设。

　　第五十四条　省人民政府及其有关部门应当加强产业链协同创新的统筹协调，引导和支持企业、高等院校、科研机构加强协同攻关，开展数字经济基础前沿研究和关键共性技术研究。

　　县级以上人民政府应当为数字经济产业科技创新和科技成果转化提供资金、数据资源和政策支持。

　　第五十五条　省人民政府数字经济主管部门应当会同财政、工业和信息化、大数据等部门将物联网、智能终端、网络安全、云计算、大数据、软件、人工智能、区块链等数字技术产品和服务列入全省集中采购目录。

　　经依法批准，政府采购的采购人可以通过非公开招标方式，采购公开招标标准限额的首台（套）装备、首批次产品、首版次软件，支持数字技术产品和服务的推广应用。

　　第五十六条　县级以上人民政府及有关部门应当将引进数字经济领域高层次、高技能以及紧缺人才纳入政府人才支持政策体系，在就业、落户、住房、医疗保健、职称评定以及配偶就业、子女教育等方面提供支持。

　　教育、人力资源社会保障、工业和信息化等部门应当加强数字经济领域人才培养，指导和督促高等院校、职业学校开设数字经济专业、课程，支持学校与企业通过产学研合作、共建产业学院、产业研究院、实习实训基地等方式，培养数字经济研究和应用型人才。

　　第五十七条　县级以上人民政府及有关部门应当支持举办、鼓励参加数字经济领域的国内国际会展、论坛、赛事等活动，搭建数字经济展示、交易、交流、合作平台，畅通供需对接渠道，提高市场开拓能力。

　　县级以上人民政府应当鼓励企业开放数字化应用场景，宣传数字经济文化，推广先进经验、成功模式。鼓励学校开展研学游、工业游，组织学生学习、体验数字经济新业态。

　　第五十八条　县级以上人民政府人力资源社会保障部门应当按照有关规定，加强对数字经济新业态用工服务的指导，制定和完善数字经济新业态的劳动保障政策，维护数字经济新业态劳动者的合法权益。

　　数字经济新业态从业人员通过互联网平台注册并接单，提供网约车、外卖或者快递等劳务的，平台经营者应当根据平台就业特点，为从业人员提供多样化商业保险保障，防范和化解其职业伤害风险。法律、行政法规另有规定的，从其规定。

　　第五十九条　省人民政府市场监管部门应当会同发展改革、工业和信息化等部门制定和完善本省数字经济标准体系并监督实施。

　　县级以上人民政府应当支持行业协会、产业联盟、龙头企业等参与制定数字经济国际规则、国际标准、国家标准、行业标准和地方标准。鼓励和支持有关单位积极参与国际标准化活动。

　　第六十条　县级以上人民政府市场监管等部门应当加强数字经济领域知识产权保护，培育和发展相关知识产权交易市场，探索建立快速维权体系，依法打击知识产权侵权行为。

　　第六十一条　县级以上人民政府及有关部门应当营造促进数字经济发展的公平竞争的市场环境，保障各类市场主体的合法权益。

　　省人民政府市场监管部门应当依法制止平台经济等数字经济领域垄断行为，依法查处滥用市场支配地位、达成或者实施垄断协议、非法的经营者集中、滥用行政权力排除限制竞争等垄断行为。

　　县级以上人民政府市场监管部门应当依法查处各类不正当竞争行为。

　　第六十二条　省网信部门应当会同省人民政府市场监管、公安、工业和信息化等部门加强互联网信息服务算法安全风险监测、算法安全评估、科技伦理审查、算法备案管理和涉算法违法违规行为处置等监管制度和体系建设。

　　算法推荐服务提供者应当遵守法律法规，尊重社会公德和伦理，遵守商业道德，遵循公平公正、公开透明、科学合理和诚实信用的原则。

　　第六十三条　省人民政府统计部门应当会同发展改革、工业和信息化等部门按照有关规定，建立数字经济统计监测机制，开展数字经济统计调查、监测分析工作。

　　第六十四条　各级人民政府及有关部门应当推进信息无障碍建设，坚持智能创新与传统服务相结合，为老年人、残疾人等运用智能技术困难的特殊群体保留传统的服务方式，鼓励针对特殊群体的出行、就医、消费、文娱、办事等，提供适用的智能化产品和服务。

　　第六十五条　各级人民政府及有关部门应当按照鼓励创新的原则，对新技术、新产业、新业态、新模式等实行包容审慎监管，给予数字经济发展相应的创新空间。

　　国家机关、企业事业单位、科研机构及其工作人员在职责范围内对数字经济改革创新进行探索，未能实现预期目标，符合下列条件的，应当免除相关责任：

　　（一）符合国家和本省确定的改革方向；

　　（二）未违反法律、法规禁止性规定；

　　（三）符合程序规范要求；

　　（四）勤勉尽责、未牟取私利。

第八章　数字经济安全保障

　　第六十六条　县级以上人民政府及有关部门应当履行数字经济安全保障职责，健全安全风险评估和安全保障制度，建立监测预警和应急处置机制，采取安全保障措施，保护数据、网络、设施等方面的安全。

　　第六十七条　自然人、法人和非法人组织的数据信息受法律保护。

　　任何单位和个人收集、存储、使用、加工、传输、提供、公开数据资源，应当坚持合法、正当、必要、精准和诚信原则，遵守网络安全、数据安全、密码安全、电子商务、个人信息保护等有关法律、法规以及国家标准的强制性要求，不得损害国家利益、社会公共利益或者他人合法权益。

　　第六十八条　县级以上人民政府网信、公安等部门应当加强对个人信息数据采集和流通各环节的监督管理，依法查处危害个人信息数据安全的违法活动。

　　第六十九条　在数字经济活动中收集和产生的数据涉及出境的，应当遵守数据安全管理的相关法律、法规，依法进行安全评估，不得影响国家安全，不得损害社会公共利益，不得侵害个人信息安全，不得侵害其他市场主体的合法权益。

　　第七十条　数据的采集人、持有人和使用人应当采取技术手段和其他必要措施，确保其收集储存的数据安全，防止数据泄露、篡改、丢失。发生或者可能发生数据泄露、篡改、丢失的，应当及时采取补救措施，按照规定告知用户和相关权利人，并向网信、公安、工业和信息化、发展改革、通信管理等有关主管部门报告。

　　第七十一条　省人民政府及其有关部门应当组织建设网络交易监测平台。有关部门应当通过网络交易监测平台，对互联网平台经营者及网络交易实施在线监测，确保网络交易安全。网络运营者应当按照网络安全等级保护制度的要求，履行安全保护义务，保障网络免受干扰、破坏或者未经授权的访问，防止网络数据泄露或者被窃取、篡改。

　　第七十二条　省人民政府应当按照国家有关规定建立网络安全应急处置机制。发生网络安全事件时，有关主管部门应当依法启动应急预案，采取相应的应急处置措施，防止危害扩大，消除安全隐患，并及时向社会发布与公众有关的警示信息。

　　网络运营者应当制定网络安全事件应急预案，及时处置系统漏洞、计算机病毒、网络攻击、网络侵入等安全风险；在发生危害网络安全的事件时，立即启动应急预案，采取相应的补救措施，并按照规定向网信、公安、工业和信息化、发展改革、通信管理等有关主管部门报告。

第九章　法律责任

　　第七十三条　违反本条例规定，法律、行政法规已有法律责任规定的，从其规定。

　　第七十四条　违反本条例第五十八条第二款规定，平台经营者未对数字经济新业态从业人员提供商业保险保障的，由人力资源社会保障部门责令改正；拒不改正的，处五万元以上十万元以下的罚款。

　　第七十五条　各级人民政府和有关部门及其工作人员，不履行数字经济促进工作职责，有下列行为之一的，由法律、法规规定的相关主管部门责令改正；情节严重的，对直接负责的主管人员和其他直接责任人员依法给予处分；构成犯罪的，依法追究刑事责任：

　　（一）未按照规定履行促进数字经济发展相关支持、保障工作职责的；

　　（二）未按照规定履行数字经济发展规划编制、实施职责的；

　　（三）未按照规定履行数据资源安全保护职责的；

　　（四）其他玩忽职守、滥用职权、徇私舞弊的行为。

第十章　附则

　　第七十六条　本条例自2022年3月1日起施行。

(12)江西省标准化战略领导小组印发《关千开展数字经济标准体系建设的实施意见》的通知

为深入贯彻中共中央、国务院印发的《国家标准化发展纲要》，落实加快发展数字经济的决策部署，按照省委、省政府《关于深入推进数字经济做优做强”一号发展工程”的意见》，以标准支撑和引领我省数字经济高质量发展，制定本实施意见。

一、总体要求

（一）指导思想

以习近平新时代中国特色社会主义思想为指导，认真贯彻党的二十大和二十届一中全会精神，深刻把握数字中国建设的战略要求，立足新发展阶段，贯彻新发展理念，构建新发展格局，聚焦“作示范、勇争先＂目标要求，围绕数字基础设施、数字产业化、产业数字化、数字化治理、数据价值化等领域，加快发展数字经济，促进数字经济和实体经济深度融合，打造具有国际竞争力的数字产业集群，助力推动数字经济成为我省现代化经济体系的主引擎。

（二）主要目标

到2025年，我省数字经济重点领域高水平标准体系进一步健全，标准化工作机制更趋完善，标准创新主体不断涌现，标准化创新应用持续深化，标准化示范推广效益更加凸显。在数字经济领域制修订国际标准、国家标准、行业标准不少于30项，地方标准不少于50项，大力培育先进团体标准；建设不少于3个国家或省级技术标准创新基地，组建不少于5个省级数字经济领域专业标准化技术委员会；面向重点行业领域，打造50个以上具有行业代表性的标准化应用场景，以点带面、延伸拓展，实现数字经济标准的规模化应用，培育一批数字经济标准化组织，有效支撑我省经济社会数字化转型。

二、重点任务

（三）夺实新型数字基础设施标准化建设

加强数字经济术语和定义、数字经济分类和通用符号、数字经济统计规范等数据通用基础标准的实施应用。推动数字基础设施标准应用先行先试，积极参与5G、物联网、工业互联网、数据中心、智能计算中心等标准研制。规划建设云基础标准设施，搭建云计算、边缘计算等多元普惠计算设施。聚焦网络基础设施数字化转型升级，推进融合基础设施标准的研制和应用。构建“数据＋算法＋算力“标准体系，探索开展重大科技基础设施、科教基础设施、产业技术基础设施等标准化前沿研究。

专栏l：数字基建标准化支撑工程

网络基础设施标准化改造工程。推进“感知城市”标准系统建设，全面升级通信网络基础设施。全面推进“双千兆”标准网络的覆盖，加快部署基于1Pv6标准的下一代互联网。建设窄带物联网、4G和5G协同发展的移动物联网综合生态体系。实现5G资源高效利用和智能适配，推进卫星互联网、车联网等基础设施标准的实施应用。（责任单位：省通信管理局、省工业和信息化厅、省科技厅、省发展改革委、省委网信办按职责分工负责）

算力基础设施标准化建设工程。实施数据中心等新型基础设施标准化应用行动，落实构建全国一体化大数据中心协同创新体系要求，以“一核四副两备“为重点，科学布局全省数据中心标准化建设，积极对接“东数西算“工程。（责任单位：省发展改革委、省大数据中心按职责分工负责）

融合应用基础设施标准化建设工程。高效布局人工智能标准化基础设施，提升支撑“智能＋“发展的行业赋能能力。加快工业互联网标识解析节点标准化建设。重点发展协同便捷的智慧交通、智慧电网、智慧管网、智能充电桩、智慧生态、智慧水利等融合基础设施的标准化建设。（责任单位：省工业和信息化厅、省通信管理局、省交通运输厅、省发展改革委、省能源局、省住房城乡建设厅、省生态环境厅、省水利厅按职责分工负责）

（四）建立数字产业化标准体系

聚焦数字产业化发展需求，推进电子信息、电信业、软件和信息技术服务业、互联网行业等基础产业标准制定。抓住VR、元宇宙及数字挛生、区块链、人工智能、量子信息、卫星互联网等新兴产业和平台直播等新业态发展时机，引导开展标准攻关研制，加快新兴产业和新业态标准研制和标准化试点示范，推动数字经济标准在各行业各领域的创新应用。围绕未来网络、碳基芯片、类脑计算等未来产业，做好关键标准超前布局，积极参与或承担国家标准乃至国际标准的制修订工作。

专栏2：数字产业标准化提升工程

LED产业标准化工程。依托硅衬底LED原创技术领先优势，推动建立硅衬底LED领域应用标准体系。（责任单位：省工业和信息化厅）

VR产业标准化工程。围绕打造世界级VR中心，构建以技术产品、配套设施、应用场景、产业融合为主的虚拟现实标准体系，探索VR、AR、MR、XR在制造、教育、交通、医疗、旅游、商贸、文娱影视等领域标准化应用建设，推动建立VR、AR、MR、XR与其他产业融合发展的应用标准体系。（责任单位：省工业和信息化厅、省教育厅、省交通运输厅、省卫生健康委、省文化和旅游厅、省商务厅按职责分工负责）

物联网标准化工程。在标识、感知、信息传送和数据处理等环节开展标准研制，在射频识别、传感、网络通信、嵌入式系统、云计算等核心技木开展标准研制。（责任单位：省科技厅、省工业和信息化厅、省通信管理局按职责分工负责）

信息安全标准化工程。完善信息安全标准体系，重点从基础标准、管理标准、测评标准、密码技术、保密技术、数据融合几方面开展标准研制。（责任单位：省工业和信息化厅）

（五）推动农业数字化转型

聚焦农业七大产业，开展智慧衣业、智慧种养殖、智能农机装备、农产品追溯等标准研制。加快智慧农业产业链、生态农业数字化、乡村信息基础设施建设、乡村管理服务数字建设等标准化试点示范建设，加快现代农业、相橘、油茶等国家农业标准化区域服务与推广平台数字化建设。探索农业绿色化、智能化、标准化发展新模式。深化”互联网＋农产品“出村进城工程，发展农产品直播带货等新模式，开展农村电商、农业物联网、直播带货等标准研制，提升“江西绿色生态”“赣鄱正品”等品牌影响力。深化“赣溯源”运用，加强产品溯源数字标准化建设。

专栏3:农业数字标准化提升工程

数字农业标准化工程。发挥数字经济赋能传统农业的作用，聚焦农业生产数字化、标准化，推动无人农场、农技数字化等标准研制。支持大田种植、园艺作物、畜禽养殖、水产养殖数字标准化项目建设。（责任单位：省农业农村厅）

农村电商标准化工程。深化电子商务进农村标准化示范，探索制定农村直播等新业态标准，多方拓宽农产品销售渠道。（责任单位：省农业衣村厅、省商务厅按职责分工负责）

智慧农业“123+N”平台标准化工程。完善农业指挥调度、12316资讯服务、农业物联网、农产品质量安全监管追溯以及农业技术服务等标准体系建设。（责任单位：省农业农村厅、省商务厅按职责分工负责）

标准化区域服务与推广平台数字化建设工程。加快建设现代农业标准化区域服务与推广平台，强化标准指标服务系统、标准化生产管控服务系统、检验检测服务系统、区块链合格证追溯服务系统、质量评价与选品服务系统应用。依托油茶农业标准化区域服务与推广平台，实现油茶产业全方位、多层次的一站式标准化服务，完成一批油茶标准关键指标的验证服务。（责任单位：省农业农村厅、省林业局、省市场监管局按职责分工负责）

（六）推进制造业数字化升级

聚焦有色金属、电子信息、中医药、装备制造、新能源、新材料等优势产业，开展智能装备、智能工厂、智能供应链、智能服务等标准研制，加快高端装备、工业大数据、绿色能源、绿色制造、新材料等领域数字化标准攻坚，加强智能制造标准体系的推广和实施推进制造业数字化升级。建立工业互联网标准，建立健全大数据与产业融合标准，推进数字产业化和产业数字化。

专栏4：制造业数字标准化提升工程

智能制造标准化升级工程。聚焦制造业与数字化深度融合，研制实施电子信息、汽车、先进材料、高端装备、时尚消费品等重点产业的数字化转型标准，鼓励工业互联网平台提供标准化服务，用标准打通产业链和供应链。（责任单位：省工业和信息化厅）

制造业标准应用试点。鼓励制造业企业运用标准化方式组织生产、经营、管理和服务，围绕智能车间／工厂建设类、新模式应用类、新技术应用类、供应链协同类等重点方向推荐标准化工作基础条件好、标准应用成效强的项目申请智能制造标准应用试点。（贵任单位：省市场监管局、省工业和信息化厅按职责分工负责）

数字赋能重点产业链提质升级。深入实施上云用数赋智工程，推进"5G＋工业互联网“标准示范应用，构建“数字化生产线—数字化车间－智能工厂—未来工厂”梯队。建设有色金属、电子信息、航空产业、家具等重点产业链质量基础设施“一站式”服务平台，加快推进“江西省VR/AR及数字信息产品质量监督检验中心“江西省数字经济（电子信息）产业计量测试中心”建设，完善企业质量数字化管理，提升产业质量水平。（责任单位：省工业和信息化厅、省科技厅、省市场监管局按职责分工负责）

（七）助推服务业数字化提质

聚焦生产性服务业，重点推进数字金融、智慧物流、数字商贸、智慧电商、会展旅游等标准研制。面向生活性服务业，推动数字体育、数字文娱、信息消费、电子商务等标准研制和标准化试点示范建设，促进平台经济、共享经济等有序发展。

专栏5：服务业数字标准化提升工程

智慧物流标准化工程。以提升物流仓储的自动化、智能化水平，促进物流与农业、制造业等产业融合发展为目标，开展智慧物流、智能仓储标准体系构建，加快对传统物流设施的数字化改造升级。（责任单位：省工业和信息化厅、省商务厅、省发展改革委按职责分工负责）

数字金融标准化工程。聚焦金融服务便利化、普惠化建设需求，探索研制普惠金融、开放银行、资产交易、支付清算、登记托管、交易监管等标准，引导各类金融机构加强相关标准的落地应用，发挥标准规范引领作用，延展数字金融服务模式，推动金融业提质增效。（责任单位：省金融监管局、人民银行南昌中心支行按职责分工负责）

数字商贸标准化工程。聚焦商贸流通新技术、新业态发展和消费升级趋势，研制实施智慧零售、智慧供应链、直播电商、线上会展、商务信用、跨境电商、精准营销、虚拟导购等标准，支撑实体商业线上化、生活服务数字化、物流配送即时化和零售终端智能化等重点方向，推动数字技术在商业场景的深度应用。（责任单位：省商务厅）

智慧养老标准化工程。聚焦老年人生活需求，研制实施智慧养老院、智能监护、设施设备等标准，提升养老服务触达性和精准度。（责任单位：省工业和信息化厅、省民政厅）

智慧文旅标准化工程。聚焦文旅资源和服务数字化需求，研制实施数字景区、数字酒店、数字博物馆、数字美术馆等领域标准研制和应用，支撑场景化、沉浸式、互动性文旅体验，推动全省商业、文化、旅游公共资源整合共享和应用。（责任单位：省文化和旅游厅）

智慧文旅标准化工程。聚焦文旅资源和服务数字化需求，研制实施数字景区、数字酒店、数字博物馆、数字美术馆等领域标准研制和应用，支撑场景化、沉浸式、互动性文旅体验，推动全省商业、文化、旅游公共资源整合共享和应用。（责任单位：省文化和旅游厅）

（八）拓展公共服务数字化标准领域

面向公共服务领域，推动文化教育、医疗健康、体育健身、智慧社区等领域数字化标准研制，推动公共服务体系化、数字化、便利化。加快推进政务标准化、规范化，推动政务服务流程优化，建立“数据多跑路“线上政务服务标准体系，实现利企便民高频服务事项“一网通办＂。积极拓展教育、医疗、社保、旅游、健身等服务内容的数字化供给和网络化服务。加强公共服务数字化标准研制，迭代升级“赣服通“服务标准体系。

专栏6:公共服务数字标准化提升工程

智慧医疗标准化工程。鼓励实体医疗机构研制“互联网＋医疗健康”标准，构建线上线下结合医疗健康等新型服务模式。构建省市县乡（村）四级远程医疗服务标准体系，提升基层医疗基本公共服务。加快建设健康信息标准化平台，推动健康医疗大数据开发应用。（责任单位：省卫生健康委）

智慧教育标准化工程。聚焦教育模式变革、教学方式改进和评价方法创新需求，研制实施线上线下融合教学、智慧课堂、虚拟现实教学等标准，支撑大规模智慧教学平台建设，以数字标准赋能推动教育发展。（责任单位：省教育厅）

智慧体育标准化工程。开展健身休闲业和体育相关培训业等体育服务领域的标准研制，引导传统体育制造业向智能化体育产业转型升级，推动“体育＋“新业态创新发展，积极探索体育产业数字转型、智能升级、融合创新。（责任单位：省体育局）

“赣服通”标准化提速工程。深入拓展“赣服通“服务功能，完善“赣服通”标准体系，打造全国“掌上办事“新标杆。（贵任单位：省政务服务办、省发展改革委按职责分工负责）

（九）创新绿色发展数字化实现路径

加强国家技术标准创新基地（江西绿色生态）及分中心建设，强化省碳达峰碳中和管理标准化技术委员会的技术支撑作用，研制能源数字化、零碳智慧园区、数字降碳工具标准，构建完善碳达峰碳中和管理标准体系。积极推进江西省绿色生态品牌价值实现及服务平台建设，制定绿色生态品牌评价标准体系，提升江西绿色生态品牌影响力。建立生态产品质量追溯和“江西绿色生态”产品质量认证标准体系，利用区块链溯源等技术，实现农产品从田间到餐桌的全过程数字化溯源，推动认证结果省际互认、国际互认。建设和完善生态系统生产总值数字化核算标准，为构建“资源—资产—资本－资金”的转化机制提供支撑，推动线上线下资源、渠道深度融合，促进生态产品供给与需求高效对接。

专栏7：绿色发展标准化工程

智慧生态标准化工程。实施生态环境大数据标准化项目，积极发展“互联网＋回收平台＂，完善生活垃圾全过程分类标准信息体系。研制实施城市生态环境保护数据实时获取分析、重点领域碳排放智能监测和动态核算等标准。（责任单位：省发展改革委、省生态环境厅、省住房城乡建设厅按职责分工负责）

自然资源数字标准化工程。研制建设空间数字底座、自然资源执法监控展示、地质灾害监测预警、自然资源栠约节约利用等标准，支撑空间地理资源系统建设。（责任单位：省自然资源厅）

碳达峰碳中和标准化工程。对接国家碳达峰碳中和重大战略目标，建立支撑碳达峰碳中和目标的技术标准体系，积极参与制定碳达峰碳中和相关的国家标准、行业标准、地方标准。加强对国家城市能源计量中心（江西）建设的支持力度，探索开展碳排放核查、计量、碳中和评价等工作，包括重点耗能设备能效测试、节能评估、企业减碳评估测试。加强碳计量量传溯源能力建设，为碳达峰碳中和提供有效的计量技术支撑。（责任单位：省发展改革委、省生态环境厅、省市场监管局等相关行业主管部门按职责分工负贵）

（十）增强数字治理标准化能力

围绕数字政府建设，聚焦数字决策、营商环境、城市管理、乡村建设、市场监管等领域数字化需求，开展智慧城管、智慧消防、智慧司法、电子政务、智慧监管、智慧气象、智慧水利等标准研制，开展农村综合改革、新型城镇化、美丽乡村等标准化试点示范建设，深入实施数字乡村战略，制定实施一批数字乡村标准。建立健全大数据、人工智能、区块链等新技术在统计监测和决策分析方面的应用标准体系，推动提升数字经济治理的精准性、协调性和有效性。鼓励和督促企业诚信经营，强化以信用为基础的数字经济市场监管，研制市场主体信用风险分类管理标准。建设数字政府一体化标准体系，完成企事业单位接入电子政务外网安全技术规范、政务外网终端安全管控等标准制定工作，推进政务外网IPv6相关标准规范制定工作。

专栏8：数字治理标准化提升工程

营商环境标准化工程。研制实施普惠金融、综合纳税、专项资金、用工就业、服务贸易等标准，整合优化各类应用场景，拓展面向企业的全周期服务标准体系，支撑“一站式、全业务“便捷服务落地。（贵任单位：省金融监管局、省税务局、省发展改革委、省人力资源社会保障厅、省商务厅按职责分工负责）

综合监管标准化工程。围绕重点监管领域的数字化转型场景需求，研制实施智能化监管、信用监管、特种设备、食品安全、数字广告等数字标准，支撑数字化重构监管模式，提升监管效能。（贵任单位：省市场监管局）

新型智慧城市标准化工程。加强新型智慧城市总体规划与顶层设计，实施“城市大脑“标准化示范工程，创新智慧城市建设、应用、运营模式，建立完善智慧城市的标准规范体系。重点聚焦城市大脑、城市智能体、数字李生城市领域开展标准攻关精准化水平。（责任单位：省住房城乡建设厅、省发展改革委按职责分工负责）

智慧交通标准化工程。整合公路、铁路、水路等各类交通数据，开展交通大数据标准化应用。推进“智慧信号灯＂、智能停车等标准制定和设备部署，缓解城市“拥堵病＂。（责任单位：省交通运输厅、省住房城乡建设厅、南昌铁路局等省直有关部门按职贵分工负责）

司法领域标准化提升工程。依托国家标准化管理委员会与司法部在标准化领域的合作基础，积极开展司法领域标准研制、标准化试点示范项目建设等工作，探索司法领域数字化转型发展。（责任单位：省司法厅、省市场监管局按职责分工负责）

智慧公共安全标准化工程。深化重大疫情防控信息标准化建设，强化“赣通码”等数字标准在疫情监测分析、防控救治等方面的应用。制定完善民防、技防、安全生产、消防和大客流监测、城市公共安全风险处置、应急感知和通信、危化品全过程监管、自然灾害风险综合监测预警、公共卫生突发事件应急处置等标准，增强城市快速响应效能。（责任单位：省卫生健康委、省发展改革委、省应急厅、省气象局按职责分工负责）

（十一）推动数据要素价值标准化

围绕推进产业数据要素市场化配置，构建数据资源标准体系建设，统一数据标准，推动数据融合。加快数据目录、数据采集、数据汇聚、数据共享、数据治理、数据安全、元数据、数据字典等基础通用标准研制，重点推进数据采集、数据确权、数据定价、数据流通、数据资产价值评估、数字经济效益测算、数据利用、数据安全、数字资产编码等标准研制，提升数据管理和利用水平。推动产业数据要素在制造、能源、物流、金融、医疗、教育等重点领域的创新应用，开展数据应用标准研制。研究制定数字经济发展评价标准，开展数字经济发展水平评价。

专栏9：数据要素价值标准化工程

公共数据共享开放标准化工程。加强公共数据基础设施集约化管理，坚持共建共享，切实防止重复建设和资金浪费。健全公共数据目录编制规范，动态发布数据共享和开放责任清单。依托省统一数据共享交换平台和数据开放平台，制定数据共享和数据开放标准。（责任单位：省发展改革委、省大数据中心、省委网信办、省政务服务办、省商务厅、省文化和旅游厅、省交通运输厅、省市场监管局等相关行业主管部门按职责分工负责）

社会数据共享标准化工程。实施工业数据分类分级标准，组织实施社会数据资源开发利用标准。鼓励企业依法依规运用消费大数据，提升产品和服务质量。（责任单位：省发展改革委、省工业和信息化厅、省大数据中心按职责分工负贵）

数据要素交易标准化工程。加强数据产品和服务定价、交易等相关标准研究，推动数据标准化、资产化、资本化，规范数据入场交易。（责任单位：省发展改革委、省工业和信息化厅、省委网信办、省金融监管局、省市场监管局按职责分工负责）

（十二）培育数宇经济标准创新主体

加大对省信息安全标准化技术委员会、省物联网标准化技术委员会和智慧城市、VR国家技术标准创新基地分中心等数字经济标准技术机构的支持力度，鼓励将数字经济相关前沿新技术、新产品等转化为具有引领性、竞争力的团体标准，支持龙头企业构建覆盖产业链上下游的标准化服务团队，通过市场化运作孵化一批数字经济标准化组织，打造一批数字经济企业标准“领跑者”。鼓励第三方机构基于标准开展数字经济领域标准化应用场景的评价或认证。推动协会、商会等社会团体发挥标准引领、标准自律、标准规范作用，制定创新性强、指标领先、企业应用范围广的团体标准，以弥补行业标准空缺，促进数字经济领域高质量发展。

专栏10:数字经济标准平台打造工程

数字经济科技成果转化。聚焦科技创新过程中的设计、检测、实验、分析等环节的标准化、数字化需求，研制实施集成电路、人工智能、生物医药和智能制造等领域的科研设施、科技资源、公共研发平台的实施标准，促进重点领域数据的规模汇聚和共享。（责任单位：省科技厅、省工业和信息化厅、省药监管局按职责分工负贵）

培育数字经济标准主体。联合政产学研用优势资源，组建数字经济领域相关标准化技术委员会，打造数字经济标准化研究智库。深化大院大所合作，争取数字经济领域国家市场监管重点实验室、国家市场监管技术创新中心、国冢标准验证检验检测试点、国家产品质量检验检测中心（国冢质检中心）等在我省落地。探索筹建江西省数字资产评估与认证中心，推动数字经济标准验证、标准检测、标准统计、标准评估和标准实施。（责任单位：省市场监管局等省直有关部门按职责分工负贵）

（十三）参与数字经济区域标准合作

加强数字经济国际交流合作，鼓励企业参与国际标准化活动，承担国际标准化技术组织工作。加大国际标准实质性参与力度，主动融入共建“一带一路”，深入对接区域全面经济伙伴关系协定(RCEP)，积极参与长江经济带发展，持续深化粤港澳大湾区建设合作，探索在数字贸易等数字经济领域开展标准化合作。加快数字经济重点领域标准互认和比对分析，鼓励企业对标数字经济国际先进标准，探索推进标准外文版工作，推动更多的先进产品、技术、服务“引进来、走出去”。

三、保障措施

（十四）加强统筹协调

充分发挥省标准化战略领导小组作用，加强数字经济标准化工作统筹与协调，与省发展数字经济领导小组加强联动，及时研究解决推动数字经济标准化建设过程中的重大问题，加快形成引领数字经济高质量跨越式发展的标准体系。对数字经济领域急需的具有重要影响的地方标准项目，优先立项并及时宪成。各单位应落实主体责任，组织开展本行业数字经济标准化建设，充分调动各方积极性，着力完善横向协同、纵向贯通的标准化工作协调机制，形成各方协同促进数字经济发展新局面。（责任单位：省标准化战略领导小组成员单位按职责分工负责）

（十五）完善激励机制

采用“揭榜挂帅”等形式支持各类创新主体开展数字经济领域标准研究和应用重点研发。鼓励企业、高校和科研机构将数字经济领域科研成果向标准转化，加快科技成果转化落地，建立健全人才激励机制，支持在数字经济领域标准化工作中做出显著成绩的单位和个人参与标准创新贡献奖评选。（责任单位：省市场监管局、省科技厅按职责分工负责）

（十六）强化实施保障

各单位要加强《国家标准化发展纲要》《江西省标准化条例》《江西省人民政府关于贯彻落实国家标准化发展纲要的实施意见》贯彻实施，加强政策与资金保障，激发企业、社会团体和教育、科研机构等参与数字经济标准化体系建设工作的积极性和于事创业活力。制定数字经济标准化人才支持相关措施，推进数字经济标准化复合型人才培育和交流。鼓励有条件的高等院校、职业院校开设相关专业，培养标准化基础知识扎实、专业水平高、熟悉国际标准化规则的专业型人才。探索推动标准化工作人员职业能力建设，推广国家职业技能标准和相关职业技能等级证书。加大巳出台的数字经济领域相关建设标准的实施力度，鼓励将标准纳入工程项目审批系统落地执行。（贵任单位：省市场监管局、省人力资源社会保障厅等省直有关部门按职责分工负责）

（十七）加强宣传培训

加强数字经济领域标准宣贯培训，开展数字经济领域标准化试点示范项目建设，充分发挥辐射带动作用，推动全社会运用数字化手段、标准化方式组织开展生产、经营、管理和服务。充分利用融媒体等新兴媒体深入宣传标准化，营造全社会重视和促进标准化工作的良好氛围。（贵任单位：省市场监管局等省直有关部门按职责分工负责）

附件

有关单位名单

省委宣传部、省委军民融合办、省委网信办、省发展改革委、省教育厅、省科技厅、省工业和信息化厅、省公安厅、省民政厅、省司法厅、省财政厅、省人力资源社会保障厅、省自然资源厅、省生态环境厅、省住房城乡建设厅、省交通运输厅、省水利厅、省农业农村厅、省商务厅、省文化和旅游厅、省卫生健康委、省应急厅、省国资委、省林业局、省金融监管局、省市场监管局、省广电局、省体育局、省政务服务办、省粮食和储备局、省能源局、省中医药局、省药监局、省工商联、省供销合作社、省税务局、南昌铁路局、人民银行南昌中心支行、南昌海关、省通信管理局、省气象局、省大数据中心

江西省市场监督管理办公室2022年11月4日印发

（13）辽宁省财政厅、辽宁省发展和改革委员会关于印发《数字辽宁智造强省专项资金(数字经济方向)管理暂行办法》的通知

各市财政局、发展改革委，沈抚示范区财政金融局、发展改革局：

　　现将《数字辽宁智造强省专项资金（数字经济方向）管理暂行办法》印发给你们，请遵照执行。

　　附件：数字辽宁智造强省专项资金（数字经济方向）管理暂行办法

辽宁省财政厅

辽宁省发展和改革委员会

2022年8月29日

　　附件

　　数字辽宁智造强省专项资金

　　（数字经济方向）管理暂行办法

　　第一章　总则

　　第一条　为贯彻落实《辽宁省人民政府办公厅关于印发数字辽宁发展规划（2.0版）的通知》（辽政办发〔2021〕25号）、《辽宁省人民政府办公厅关于印发辽宁省加快发展数字经济核心产业的若干措施的通知》（辽政办〔2022〕35号）等相关要求，按照《中华人民共和国预算法》《辽宁省人民政府办公厅关于印发辽宁省本级财政资金管理暂行办法的通知》（辽政办发〔2021〕31号）等有关规定，制定本办法。

　　第二条　本办法所称数字辽宁智造强省专项资金（数字经济方向）（以下简称“专项资金"）是指省财政预算安排，支持推动全省数字经济发展的专项资金。

　　第三条　专项资金安排和使用遵循聚焦重点、精准发力，择优立项、结果导向，科学规范、强化监督的原则，坚持清单化管理、项目化实施、工程化推进。

　　第四条　专项资金安排及项目组织实施由各级财政、发展改革部门按照工作职责分工落实。

　　（一）省财政厅负责筹措专项资金，会同省发展改革委制定资金管理办法，审核省发展改革委提出的专项资金分配方案，下达资金指标，组织开展绩效管理，会同省发展改革委指导各市加强资金监管等工作。

　　（二）省发展改革委负责制定并发布年度项目申报指南，组织各市及相关部门申报并审核下达项目计划，提出专项资金分配方案。编制绩效目标并开展绩效监控评价等。会同省财政厅指导各市及相关部门做好项目储备、开展项目实施等工作。

　　（三）省、市、县（市、区）相关部门结合本部门工作职责，加强项目立项前期准备工作，做好项目规划，建立项目储备库；组织项目实施，推进项目建设，加强资金监管。

　　第二章　资金支持内容和方式

　　第五条　支持内容和方式：

　　（一）支持发展总部经济。对世界500强中数字经济核心产业企业，以及全国电子信息百强、软件百强、互联网百强企业来辽设立总部、区域总部和研发中心、技术中心、采购中心、结算中心等功能性机构，给予最高2000万元资金奖励。

　　（二）支持做强电子元器件及设备制造业。巩固提升集成电路装备及关键零部件产业发展优势，推动电路类、连接类、机电类、传感类、光通信、功能材料等信息技术产业及配套产业提质增效。对新建、增资扩产项目建设，按照不超过投资额30%，给予单个项目最高3000万元资金支持。

　　（三）支持加速培育电子商务平台。对获评国家数字商务企业、国家电子商务示范企业，给予最高300万元资金奖励；对其新建的新技术、新模式应用项目，按照不超过投资额30%，给予最高500万元资金支持。

　　（四）支持推动产业链提升。鼓励数字经济龙头企业通过投资（参股）、并购、重组、外包服务等方式获得先进适用技术，积极引进上下游生产企业或服务性企业，形成产业“上下游"供需链条，增强产业竞争力。对引进超亿元项目的企业，按照不超过投资额5%，给予最高1000万元资金奖励。

　　（五）支持建设产业集群。以园区为载体集聚创新资源和要素，推动集成电路装备、软件、工业互联网产业集群发展。支持创建国家级集成电路装备基地，开展省级数字经济园区等评定。对国家和省新认定的集成电路基地、数字经济园区等主体，给予最高500万元资金奖励。

　　（六）支持融合基础设施建设及创新应用。鼓励应用5G、工业互联网、物联网、大数据、云计算、人工智能、虚拟及增强现实等技术发展新模式、新业态。对交通、能源、民生、环境、乡村、城市、农业、服务业等领域数字技术标杆示范、典型应用项目，按照不超过投资额30%，给予单个项目最高1000万元资金支持。

　　（七）支持创新基础设施建设。对新批准设立或优化整合的数字经济领域国家工程研究中心、国家产业创新中心、国家企业技术中心等创新平台，以及评估优秀的数字经济领域省级数字化转型促进中心、省级工程研究中心等，给予最高500万元资金奖励。

　　（八）支持承担国家支持的场景应用项目。国家要求地方安排配套资金的，按照国家实际拨付资金及规定配套比例安排资金；国家没有配套要求，对在新型基础设施建设、数字技术应用等方面有较大作用的，原则上按照不超过国家拨付项目资金比例1：1安排。对单个项目配套资金超过2000万元（含）的，报省政府审议。

　　（九）支持省级举办发挥引领牵动作用的数字经济创新创业等活动，按照政府购买服务相关管理制度执行。

　　第六条　专项资金的支持主体为具有独立法人资格、诚信经营、依法纳税，切实履行社会责任，具有良好的信用记录和社会信誉，运行管理规范的企事业单位；近三年来未发生过重大安全、质量、环保事故；符合国家和我省产业政策，有良好的经济和社会效益，对新型基础设施建设和数字经济发展有积极推动作用。

　　第七条　推荐项目应符合有关政策要求，对国家产业结构调整指导目录中限制类和淘汰类项目，项目单位被依法列入严重失信主体名单，已获得国家或省级各类财政资金支持的项目，高耗能、高排放、低水平项目不予支持。

　　第三章　资金分配及下达

　　第八条　资金分配采取项目法安排，实行自下而上申报，省级择优确定项目或事项。省发展改革委依据国家及省数字经济重点支持方向，对各地申报项目组织审核。

　　第九条　各市及相关部门应做好项目前期规划、建立和完善项目库，并结合本地区情况实行动态调整。按照省下发的项目申报指南，组织企事业单位申报项目并进行审核推荐，将审核通过的项目及资金申请书等申报材料报省发展改革委。项目申报单位和企业对申报项目的真实性、准确性负责，落实信用承诺，履行项目实施和资金管理主体责任。不得将同一项目、同一内容重复申报或多头申报。

　　第十条　省发展改革委组织相关专家或委托第三方机构对申报项目进行评审，确定拟支持项目。除涉密项目外，原则上通过互联网等媒介向社会公示，公示期为5天。对公示无异议的项目，按程序下达项目计划和绩效目标。省财政厅下达资金指标。

　　第十一条　各市及相关部门收到省下达的项目计划及资金指标后，按照国家及省规定时间分解任务计划，及时下达资金指标。要建立责任分工，明确完成时限，组织做好项目实施，并做好相关材料存档备查。

　　第四章　监督和绩效管理

　　第十二条　各级财政、发展改革部门和专项资金使用单位，要按照预算及国库集中支付等有关规定管理使用专项资金，确保专款专用。要加快预算执行，提高资金使用效益。结转结余资金，按照省财政厅《关于修订省财政收回存量资金管理暂行办法的通知》（辽财预〔2020〕319号）等有关规定执行。

　　第十三条　项目单位应严格执行国家有关法律法规和政策要求，落实项目法人责任制、招标采购制、工程监理制、合同管理制等制度，确保项目安全质量，不得擅自改变主要建设内容、降低建设标准。同时，做好专项资金财务管理，自觉接受财政、发展改革、审计等部门的监督检查。项目完工后，应按国家有关规定组织竣工验收。

　　第十四条　各级发展改革部门应会同有关部门全面加强项目日常监管，每月10日前通过辽宁省重大项目管理平台报送项目进展情况。

　　第十五条　各级财政、发展改革部门和专项资金使用单位，应按照国家和省绩效管理有关规定，实施专项资金全过程预算绩效管理，做好绩效运行监控和绩效评价。各市及相关部门组织开展绩效自评，每年3月底之前将上年度绩效自评报告报省财政厅，并抄送省政府相关部门。

　　第十六条　省财政厅会同省发展改革委适时组织开展专项资金重点绩效评价，可根据需要邀请专家、中介机构等第三方参与评价。对监督和绩效评价中发现的项目实施严重迟缓、资金长期滞留等问题将视情况采取通报、约谈、调减补助资金等措施。

　　第十七条　任何单位及个人不得截留、挤占和挪用专项资金。对于违反国家法律、行政法规和有关规定的单位和个人，有关部门应当及时制止和纠正，并严格按照《中华人民共和国预算法》《中华人民共和国监察法》《财政违法行为处罚处分条例》等予以处理。构成犯罪的，依法追究刑事责任。

　　第五章　附则

　　第十八条　本办法由省财政厅、省发展改革委按职责分工负责解释。

第十九条　本办法自发布之日起施行。如遇政策变化，将适时做出调整。各级各相关部门可根据本办法，结合本地区实际研究具体落实措施。

（14）山西省人民政府办公厅关于数字经济高质量发展的实施意见

晋政办发〔2022〕54号

各市、县人民政府，省人民政府各委、办、厅、局：

　　为深入贯彻落实国家《“十四五”数字经济发展规划》，深化省委、省政府建设“数字山西”的战略布局，大力发展数字经济，培育壮大新动能，全面立体构建全省数字经济发展体系，经省人民政府同意，现结合我省实际提出如下意见。

　　一、指导思想

　　以习近平新时代中国特色社会主义思想为指导，全面贯彻落实习近平总书记考察调研山西重要指示精神，深入贯彻省第十二次党代会和省委经济工作会议关于数字经济决策部署，以国家资源型经济转型发展、能源革命综合改革试点、黄河流域生态保护和高质量发展为契机，将数字经济作为转型发展重要方向，充分发挥数据作为关键生产要素的倍增效应，聚力打造数字基础设施一流，技术创新能力突出，产业生态体系完善，融合应用成效显著，新技术、新业态、新模式蓬勃发展的数字经济发展新格局，为我省全方位推动高质量发展提供有力支撑。

　　二、总体目标

　　到2025年，全省数字经济发展进入“加速期”，数字经济核心产业增加值占地区生产总值比重达到5.4%，数字产业化和产业数字化迈入快速拓展期，数字化治理发展成效显著，数据价值化有序推进，数据作为关键生产要素的价值显现。

　　——数字基础设施体系基本成型。5G网络、千兆光纤网络建设提速，全省5G基站累计达到9.21万个，布局合理、绿色集约的一体化大数据中心协同创新体系基本建成，全省在用在建数据中心机架规模达到50万架，新建大型、超大型数据中心PUE值降到1.2以下。

　　——数字产业化水平大幅提升。数字经济核心产业不断培育壮大，打造一批在全国有影响力的数字技术应用先导区、数字产业发展集聚区。通用计算设备、智能硬件、光电信息等电子信息制造业产业集聚与创新水平显著提升，光电信息产业集群跻身全国先进行列。

　　——产业数字化跨越式发展。依托数字技术和数据资源，推动传统产业产出增加和效率提升。培育500个智能车间和智能工厂，传统制造业重点领域基本实现智能化制造。工业互联网标识解析二级节点达到8个以上，推动龙头企业建成3个以上具有行业影响力水平的工业互联网平台。数字农业高效发展，数字技术在农业生产经营中的应用水平显著提升。

　　——数字化治理水平提档升级。数字技术与社会治理、民生服务等领域深度融合。政府数字化转型加快，网上政务服务能力迈入全国第一方阵。数字生活新服务体系基本建成，教育、医疗等领域公共服务数字化水平显著提升。数据资源化、资产化、资本化价值转化有序推动，数字经济协同治理监管机制基本建成。

　　三、重点任务

　　（一）数字基础设施优化升级工程

　　1.建设先进泛在网络基础设施。加快5G网络建设，推进5G网络向有条件的重点乡镇和农村延伸，加强产业园区、交通枢纽、景点等流量密集区域深度覆盖。强化5G基站建设要素资源供给保障，加快各类社会公共资源向5G基站开放共享，开展铁塔、管道及配套设施共建共享。深化太原国家级互联网骨干直联点作用，持续提升网间带宽能力。（责任单位：省通信管理局、省工信厅）

　　2.打造绿色协同算力基础设施。有序推进新型数据中心、绿色数据中心等智能算力基础设施规模化、集约化、绿色化发展，打造网络安全产业基地、数据服务应用基地。加强数据中心能耗监测管理，提高数据中心能源供给结构中低碳、零碳能源占比，选树一批新型数据中心典型案例。引导新建大型、超大型数据中心PUE值不超过1.2，促进数据中心绿色低碳发展。培育壮大数据中心集群，积极融入国家“东数西算”工程。（责任单位：省发展改革委、省工信厅、省通信管理局、省能源局）

　　3.推动数智赋能融合基础设施。推动农业基础设施和生产装备智能化改造，打造一批智慧农业基地。建设高可靠、广覆盖、大带宽、可定制的工业互联网基础设施，持续推进标识解析二级节点建设，打造特色型工业互联网平台。加快推进能源、交通运输、物流等领域基础设施数字化改造。构建先进普惠、智能协作的生活服务数字化融合设施，打造智慧共享、和睦共治的新型数字生活。（责任单位：省各有关部门）

　　（二）数字技术创新突破工程

　　4.增强核心技术攻关能力。实施前沿引领技术基础研究专项，取得一批数字经济领域引领性原创成果。深入推行“揭榜挂帅”，鼓励企业、高校、科研院所围绕集成电路、核心软件、云计算与大数据等重点领域的“卡脖子”技术，开展联合研发攻关，推动数字技术自主可控安全高效，超前部署量子科技、人工智能、区块链、智能物联网等前沿技术研发。在智能感知、数据治理、计算与建模、数据安全等领域，实施一批关键技术攻关项目。（责任单位：省科技厅）

　　5.打造高能级数字创新平台。支持先进计算山西省实验室及智能信息技术处理、先进控制与智能信息系统、智能感知等省级实验室建设，争创国家级实验室。加快推进国家先进计算太原中心建设及应用，争创高性能计算应用等领域国家技术创新中心。依托骨干企业，建设山西省能源互联网平台、山西省农业大数据平台、山西省文旅大数据平台、山西省医疗大数据平台等一系列行业领域应用平台。（责任单位：省科技厅、省发展改革委、省工信厅、省能源局、省农业农村厅、省文旅厅、省卫健委）

　　6.培育多元化创新主体。建立企业为主体、高校和科研院所协同发力、产学研用融合的创新体系，鼓励发展新型研发机构、企业创新联合体等新型创新主体，打造多元化参与、网络化协同、市场化运作的创新生态体系。设立大数据、人工智能等相关领域科技研发专项，形成支撑数字化转型的学科体系和产业创新体系。（责任单位：省科技厅、省教育厅）

　　（三）数据要素市场培育工程

　　7.加快数据协同高效汇聚。建设完善人口、法人、公共信用、宏观经济、空间地理、电子证照六大基础信息资源库，加快建设工业、能源等重点领域专题数据库，促进数据资源高效汇聚和共享利用。完善省、市两级政务数据资源共享体系，推动政务数据跨层级、跨地域、跨系统、跨部门、跨业务共享和“一源多用”。开展政务数据开放平台建设试点，探索将公共数据开放服务纳入公共服务体系，推动公共数据分类分阶段向社会开放。（责任单位：省审批服务管理局等省各有关部门）

　　8.推动数据有序流通交易。探索开展数据资产价值评估试点，研究产权交易、行业自律、风险防范机制，推动形成数据资产评估、登记结算、交易撮合、争议仲裁等市场运营体系。鼓励发展数据治理、数据代理、数据加工等新兴数据服务，引导更多主体参与数据流通。培育发展数据交易平台，建设山西省大数据交易中心，构建数据资产市场化流通体系，营造数据可信流通环境。（责任单位：省发展改革委、省工信厅、省市场监管局、省地方金融监管局）

　　9.促进数据深度开发利用。推动各级部门深化政务数据应用创新，引导数据应用场景示范建设。探索开展政府数据授权运营试点，鼓励第三方机构对公共开放数据汇聚整合、深度加工和增值利用，提升大数据创新应用解决方案能力。深化数据管理能力成熟度评估（DCMM）试点，推动DCMM标准系列宣贯活动，强化数据管理标准体系建设，打造高素质数据管理人才队伍，鼓励各市在资金补贴、人员培训、贯标试点等方面出台支持政策。（责任单位：省审批服务管理局、省工信厅、省通信管理局等省各有关部门，有关市政府）

　　（四）数字产业集群发展工程

　　10.巩固提升大数据产业优势。围绕数字基础设施、数据基础服务、数据融合应用等大数据产业链条关键环节，培育、引进行业领军企业，壮大数据产业市场主体。深入研究数字产业发展要素，培育大数据产业基地。（责任单位：省工信厅、省通信管理局、省发展改革委）

　　11.聚焦推动信创产业发展。依托信创龙头企业，推动龙头带动、产业协同、支撑完善的信创产业基地（园区）建设。开展信创行业应用试点，推动供给侧与需求侧协同发展，加速信创产业资源高效汇聚。加大核心技术攻关、产业链上下游对接配套、重大项目跟踪服务、龙头骨干企业培育，提升信创产业链现代化水平。（责任单位：省工信厅等省各有关部门，山西综改示范区管委会，有关市政府）

　　12.提升电子信息产业制造能力。围绕光机电、半导体、光伏、计算机等重点领域，培育引进行业头部企业，大力发展智能终端、相机模组、光伏电池、锂离子电池、碳化硅半导体、LED、电子专用设备、安全计算机等主导产品，提升核心关键技术，打造电子材料—专用设备—电子元器件—零部件—整机—应用产业链条，形成国内领先的光伏制造基地和第三代半导体材料产业基地。（责任单位：省工信厅、省科技厅）

　　（五）产业数字化转型提升工程

　　13.创新发展智慧农业。构建农业农村数字资源体系，完善农村综合信息服务平台，提升农业全产业链数字化水平。推进物联网、大数据、人工智能、区块链等数字技术与农业生产经营深度融合，赋能传统产业转型发展，催生新产业、新业态和新模式。加快国家数字乡村试点建设，在全省分级分批开展省级数字乡镇、数字农村试点。（责任单位：省农业农村厅、省委网信办、省审批服务管理局）

　　14.纵深推进工业数字化转型。加快推动研发设计、生产制造、经营管理、市场服务等全生命周期数字化转型，推动工业企业数字化、网络化、智能化水平。强化智能制造支撑体系建设，形成钢铁冶金、轨道交通、煤机装备、汽车制造等智能化产业集群。积极开展智能制造试点示范和智能诊断活动，推进智能制造单元、智能生产线、数字化车间、智能工厂建设。（责任单位：省工信厅）

　　15.全面加快服务业数字化转型。充分发挥数字化对商贸、物流等现代服务业提质增效作用，提高服务业的品质与效益。加快发展电子商务，深化重点企业和专业市场电子商务应用，推动传统商超数字化转型。加快智能物流建设，形成一批设施先进、智能化程度高的物流枢纽、物流园区、配送中心和智能仓储。推进网络货运平台发展，推进多式联运、甩挂运输和共同配送等运输新模式，探索开展无人智慧配送试点推广工作。（责任单位：省发展改革委、省工信厅、省商务厅、省交通厅）

　　16.着力推动重点行业数字化转型。加快推动能源生产、运输、消费等各环节智能低碳转型。加快开展能源互联网试点建设，扎实推进煤矿智能化试点建设，坚持示范引领和全面推广相结合的方式，推进“5G+智能矿山”高水平发展。构建全省智能交通基础设施体系，加快感知、传输、计算等设施与交通基础设施协同高效建设。加快推进综合客运枢纽等基础设施智能化升级，积极稳妥推动“车联网”应用示范，开展车路协同应用试点。（责任单位：省能源局、省发展改革委、省交通厅，有关市政府）

　　（六）公共服务数字普惠工程

　　17.深化开展智慧政务服务。实施数字政府基础能力提升工程，加快构建统一、规范、多级联动的“互联网＋政务服务”体系。推动5G、视频专网、物联网等与电子政务外网融合互联，提升电子政务外网带宽和服务能力。依托一体化在线政务服务平台，提升政务服务事项网上全流程办理能力，构建全省政务服务“一网通办”总门户。不断丰富移动政务客户端“三晋通”服务范围和功能，推动电子社保卡、医保卡电子凭证、电子居住证等关系民生领域服务事项接入“三晋通”。（责任单位：省审批服务管理局等省各有关部门）

　　18.大力推进智慧医疗建设。提质升级省全民医疗健康大数据平台，完善医疗标准体系建设和数据质量治理，开展业务监管和惠民应用。深入推进互联网医院建设，促进优质医疗资源下沉和“互联网+医疗健康”便民服务应用。开展5G技术提升县级医疗集团服务能力试点项目，推广远程会诊、远程超声、远程心电、远程探视等医疗数字化服务应用。（责任单位：省卫健委、省审批服务管理局）

　　19.持续推进智慧教育建设。构建完善由省级主干网、市县教育网和校园网组成的新型教育信息网络，全面覆盖全省教育行政机构和各级各类学校。推动“晋教云”基础服务体系建设，开展教育网络服务、管理服务、教学应用上“晋教云”行动。构建“互联网+教育”公共服务平台，整合各级教育平台数据与资源，搭建省级智慧教育数据资源库。实施数字化校园扩容提速工程，升级校园网络，加快智能终端部署，推进智慧校园建设。（责任单位：省教育厅、省审批服务管理局）

　　20.构筑全域智慧文化旅游圈。以三大世界遗产地和全省5A级旅游景区为示范，大力发展5G+智慧旅游，加快建设智慧景区，提供集游客导览、客流统计、应急预警、咨询投诉和执法监督为一体的智慧指挥平台。升级完善“游山西”App功能，发展“云游览”“云观赏”等新服务。推进智慧文娱建设，研发民间美术、手工技艺、民俗等数字文创产品，实现对文化遗产的数字化保存传承。（责任单位：省文旅厅）

　　21.持续推进新型智慧城市建设。发挥标杆示范引领作用，稳步推进省级新型智慧城市试点建设，打造高水平新型智慧城市样板。支持建设城市大脑、数字孪生模型等应用赋能平台，推动城市数据整合共享和业务协同，提升城市综合管理服务能力。实施城市市政基础设施智能化改造，推动城市信息模型（CIM）和数字孪生技术在城市运行管理中的应用。（责任单位：省发展改革委、省委网信办、省住建厅）

　　（七）数字安全防护保障工程

　　22.强化关键信息基础设施保护。建设完善省关键信息基础设施安全保卫平台，在关键部位部署网络安全威胁分析设备，采集、汇聚、分析全省重要网络系统网络威胁情况。加强行业部门平台、云平台、厂商平台与安全保卫平台对接，提升业务协同联动能力，构建条块结合、纵横互通、联合预警、协同防御的关键信息基础设施安全防护体系。（责任单位：省公安厅）

　　23.提升数据安全保障水平。建立健全数据安全治理体系，研究完善行业数据安全管理政策。建立数据分类分级保护制度和数据目录管理制度，规范数据采集到销毁全生命周期管理，强化数据安全风险评估、监测预警和应急处置。依法依规加强政务数据安全保护，做好政务数据开放和社会化利用的安全管理。强化个人信息保护，加强安全监管，规范身份信息、隐私信息、生物特征信息的采集、传输和使用。（责任单位：省委网信办、省公安厅、省工信厅、省审批服务管理局）

　　24.建立完善数字经济治理体系。健全政策制度，提升数字经济治理体系和治理能力现代化水平。加强征信建设，鼓励和督促企业诚信经营，建立完善信用档案，强化以信用为基础的数字经济监管模式。强化平台经济规范化、健康化发展，明确平台企业主体责任和义务，建立行业自律机制。建立完善政府、平台、企业、行业组织和社会公众多元参与、有效协同的数字经济治理新格局。（责任单位：省发展改革委、省工信厅、省市场监管局）

　　（八）区域合作协同拓展工程

　　25.融入国家重大发展战略。加强统筹谋划，高水平融入京津冀协同发展、黄河流域生态保护和高质量发展等国家重大区域发展战略。紧抓山西能源革命综合改革试点机遇，主动服务雄安新区，承接科技创新、数字产业核心区域业务。深化黄河金三角区域合作，建设区域级云平台、数据交换平台和网络安全中心，共建区域信息网络体系和交流平台，构建跨区域、跨行业、跨业务的“数字黄河金三角”。（责任单位：省发展改革委、省工信厅、省商务厅、省审批服务管理局）

　　四、保障措施

　　（一）加强统筹协调

　　充分发挥省新型基础设施建设领导小组和省数字经济发展领导小组作用，加强对全省数字经济发展的组织领导和统筹协调。聚焦数字经济核心产业重点领域，建立健全工作协调机制，完善配套政策，统筹推动数字经济政策落实及项目建设。借助外力外脑，加强战略研究，为全省数字经济发展提供决策支撑。（责任单位：省发展改革委、省工信厅）

　　（二）强化要素支撑

　　加大省级财政资金对数字经济核心产业和重大项目支持力度，强化政银企合作，常态化开展线上融资对接和重点项目线下专场对接。深化人才支撑，大力推进柔性引才，自主培育专项人才，完善人才激励机制，创优人才集聚环境。拓展招商深度，积极发掘以商招商、产业链招商、园区招商等多种招商形式，打造全链条、多领域、高层次的招商引资格局。（责任单位：省工信厅、省发展改革委、省地方金融监管局、省教育厅、省商务厅）

　　（三）优化营商环境

　　按照“对标先进、争创一流”要求和“三无三可”理念，制定出台以“承诺制+标准地+全代办”改革为牵引的全链条优化我省营商环境的改革政策体系，进一步拓展营商环境相关行业领域数字化应用。（责任单位：省商务厅、省审批服务管理局）

山西省人民政府办公厅

2022年7月4日

（15）云南省人民政府办公厅印发关于大力推动数字经济加快发展若干政策措施的通知

（云政办发〔2022〕32号）

各州、市人民政府，省直各委、办、厅、局：

《关于大力推动数字经济加快发展的若干政策措施》已经省人民政府同意，现印发给你们，请认真贯彻执行。

云南省人民政府办公厅

2022年4月23日

关于大力推动数字经济加快发展的若干政策措施

　　为深入贯彻落实党中央、国务院关于发展数字经济的决策部署，推动我省数字经济加快发展，培育壮大新动能，助力高质量发展，结合我省实际，制定以下政策措施。

　　一、支持数字基础设施建设

　　（一）保障以5G为代表的信息基础设施建设空间。将信息基础设施建设纳入相应层级的国土空间规划及有关专项规划，按照项目建设时序同步保障电力、管道等配套设施建设。制定充分利用建筑物、构筑物、市政地下综合管廊等空间的规定，加快出台信息基础设施设计、建设和验收标准、规范，实现信息基础设施与以上空间同步设计、同步建设和同步验收。（省自然资源厅、省住房城乡建设厅、省交通运输厅、省能源局、省通信管理局、云南电网公司、各基础电信运营企业，各州、市人民政府按照职责分工负责）

　　（二）推进公共资源开放和共建共享。开放公共机构和国有企业所属的民用建筑、公共设施、公共绿地，以及政府投资为主的公共设施（电力设施等法律法规另有规定的除外），用于通信基站及其配套机房、汇聚机房建设。各州、市人民政府推动住宅区、商务办公楼宇向通信基础设施建设单位开放；在保证安全运行的前提下，统筹考虑城乡建筑风貌要求，推动通信杆塔与电力、市政、交通等杆塔资源实现双向开放和一杆多用，鼓励探索多样化合作模式。（各州、市人民政府，省财政厅、省卫生健康委、省国资委、省机关事务局、省通信管理局牵头；省直有关部门按照职责分工负责）

　　（三）降低通信网络建设租赁费。公路、铁路、地铁等交通基础设施行政管理部门、通信行业管理部门和投资运营主体要大力支持信息基础设施建设，与基础电信、广电网络和铁塔企业紧密协作，按照资源共享、互惠互利原则，推进交通基础设施与沿线信息基础设施同步规划、同步设计、同步实施、同步开通。基础电信、广电网络和铁塔企业使用高速公路路网管孔，租赁费按照不高于4000元/管孔/公里/年结算价格签订租赁合同；使用高速公路产权内土地等有关资源的通信基站综合租赁费按照不高于4000元/基站/年结算价格签订租赁合同；既有合同在期满后按此标准执行，租赁企业不得转租。（省交通运输厅牵头；省住房城乡建设厅、省通信管理局，各州、市人民政府配合）

　　（四）加强项目审批和用地支持。依法依规高效办理铁塔、基站建设审批手续，力争缩短各环节审批周期，提高行政服务效率。项目所在地县级自然资源行政主管部门要优先组织保障铁塔企业依法依规取得用地。通信设施项目可按照公共设施用途落实用地，新建基站、铁塔用地可直接取得政府供应土地，也可采取配套建设方式、依法取得地役权的方式使用土地。（省自然资源厅、省通信管理局，各州、市人民政府按照职责分工负责）

　　（五）加强电力保障。开通数字经济企业电力接入绿色通道，优先保障数字经济园区、企业和信息基础设施的电力接入。支持通信基站转供电改直供电，由州、市人民政府会同供电企业和基站所在单位根据电力设施产权归属对满足转改直条件的存量基站进行直供电改造，2022年底前应改尽改；对于暂时只能使用转供电的基站用电，执行国家规定，不得加价。新建基站原则上全部采用直供电方式就近引电，优化报装流程，对于未实行“三零”服务的低压非居民用户、高压单电源用户、高压双电源用户，供电企业用电报装时间分别压减至6个、22个、32个工作日以内。鼓励5G基站、算力中心运营企业参与电力市场化交易，不受电压等级和用电量限制。（省能源局、省通信管理局、云南电网公司、昆明电力交易中心、各基础电信运营企业，各州、市人民政府按照职责分工负责）

　　（六）推动算力基础设施布局。构建布局均衡、协同供给、梯次连续的算力基础设施体系。（省发展改革委牵头；省工业和信息化厅配合）合理布局智能计算中心等算力基础设施，支撑人工智能等新技术应用。引导算力基础设施向集约化、一体化、绿色化、智能化方向发展，降低能耗并提高使用率。（省工业和信息化厅牵头；省通信管理局配合）从全省电源使用效率（PUE值）不高于1.3的大中型算力基础设施中评选一批省级绿色算力中心并授牌。（省发展改革委牵头；省工业和信息化厅、省能源局配合）

　　（七）推动昆明国际通信枢纽建设。由省通信管理局、省发展改革委会同省直有关部门组建工作专班，加快昆明国际通信全业务出入口局、昆明国家级互联网骨干直联点、根域名镜像服务器等项目的前期工作和申报工作。省预算内前期工作经费对上述项目前期工作予以支持，项目申报成功后，省财政和昆明市财政给予适当建设资金支持。（省通信管理局、省发展改革委、省工业和信息化厅牵头；省财政厅等部门，昆明市人民政府，各基础电信运营企业配合）

　　二、支持数字产业化发展

　　（八）培育省级数字经济园区。每年评选5个重点数字经济园区，每个园区省级给予2000万元资金支持，用于支持园区新型基础设施建设、产业培育等工作。（省发展改革委牵头）

　　（九）推动数字经济企业集聚发展。支持数字经济企业向重点数字经济园区集聚，出台政策支持园区特色化、差异化、专业化发展。（省发展改革委牵头；省工业和信息化厅等部门配合）有关州、市人民政府对入驻园区的数字经济企业在用地、网络使用、办公场所、人才公寓等方面给予优惠支持；有关州、市人民政府可在数字经济企业主营业务收入首次突破1亿元（含，下同）、2亿元、5亿元、10亿元时，分别给予100万元奖励，可超额累进。（昆明市等有关州、市人民政府牵头；省工业和信息化厅等部门配合）

　　（十）积极培育市场主体。支持数字经济企业打造制造业单项冠军和专精特新“小巨人”企业，国家级、省级制造业单项冠军和专精特新“小巨人”企业，由省级中小企业发展专项资金择优给予支持。首次上榜中国电子信息百强、软件和信息技术服务竞争力前百家的企业，对进入榜单前10名、11-30名的企业，分别给予300万元、100万元一次性奖励，连续2年进入上述榜单且排名提档的，给予差额奖励。（省工业和信息化厅牵头）

　　（十一）招引企业落地。鼓励数字经济企业在我省设立研发中心或子公司，对实际到位资本金达5000万元（含）以上的，可由企业所在地州、市人民政府给予100万元的一次性落户奖励。（各州、市人民政府牵头）对于重大项目，可采取“一企一策”、“一事一议”的方式，在国家法律法规允许的前提下，从用地、资金、建设、用电及设备投入等方面予以支持。（有关州、市人民政府牵头；省工业和信息化厅、省财政厅按照职责分工负责）

　　（十二）大力发展平台经济。支持各州、市人民政府联合行业协会、互联网公司等各类主体，围绕地方特色产品和群众生产、生活需要，建设网络销售、生活服务、社交娱乐类平台，每年评选不超过10个行业特色鲜明、整合资源明显、示范引领作用强，并且具有良好经济和社会效益的平台，对于项目实际投资3000万元及以上的，给予300万元一次性补助。（省发展改革委牵头；省直有关部门配合）

　　三、支持产业数字化转型

　　（十三）持续推进“数字化转型伙伴”行动。支持由企业或机构自主投资建设数字化转型促进中心，对服务企业数超过200家（含）的，每年择优评选不超过10家，对于数字化转型促进中心年度运营成本300万元及以上的，给予运营企业或机构100万元一次性补助。（省发展改革委牵头；各州、市人民政府配合）

　　（十四）推进传统产业园区数字化转型。遴选10个以上园区作为试点，进行智慧园区建设，每个试点园区从省级新型基础设施建设专项资金中给予2000万元支持，试点任务完成后，总结建设经验，形成可复制模式在全省推广。（省发展改革委牵头；各州、市人民政府配合）

　　（十五）支持平台企业发展

　　引进一批重点平台企业。支持引进重点平台企业，由州、市人民政府按照企业年纳统数据分以下两档给予奖励：限额以上企业年纳统额2亿元（含，下同）以上或规模以上营利性服务业企业年纳统额2000万元以上；限额以上企业年纳统额5亿元以上或规模以上营利性服务业企业年纳统额5000万元以上。（省商务厅，各州、市人民政府按照职责分工负责）

　　示范一批创新平台企业。经商务部首次确认的数字商务企业，由省财政给予一次性50万元奖励。经省商务厅首次确认并向社会公布的省级数字商务企业（每年不超过8家），由省财政给予一次性25万元奖励。（省商务厅牵头）

　　四、支持数字化应用

　　（十六）支持企业数字化改造。每年支持打造不超过10个企业数字化转型标杆，对于固定资产投资额（指厂房和设备，下同）2000万元及以上的，给予每个标杆项目200万元一次性补助；对新认定省级两化融合重点项目，择优评选不超过10个项目，对于近3年信息化有关软硬件投资额2000万元及以上的，单个项目给予100万元一次性补助。对新列入工业和信息化部两化融合管理体系贯标试点的企业，给予10万元-30万元的一次性奖励。对企业投资生产自动化（智能化）成套设备项目年度固定资产投资额1亿元以上的，择优评选不超过5个项目，每个项目给予500万元一次性补助。（省工业和信息化厅牵头）

　　（十七）政府数字化应用示范推广。突出应用导向和问题导向，推动各地各部门数字化应用创新，在经济调节、生态保护、城市管理、公共服务、市场监管、机关运行等领域的政府数字化应用项目中，每年评选20个优秀应用项目，在全省进行重点宣传推广。（省发展改革委牵头；各州、市人民政府，省直有关部门配合）

　　（十八）支持智慧县城建设。遴选10个县城作为试点，进行智慧县城建设，每个试点县从省级新型基础设施建设专项资金中给予2000万元支持，试点任务完成后，总结建设经验，形成可复制模式在全省推广。（省发展改革委牵头；各州、市人民政府，省直有关部门配合）

　　（十九）评选数字经济应用示范项目

　　支持建设一批5G、物联网、大数据、云计算、信创、人工智能、智能制造领域的新技术、新产品、新模式、新应用、新业态应用试点。每年评选5G标杆示范项目及典型应用场景10个、人工智能产业应用示范项目10个、物联网产业应用示范项目30个以上、大数据示范试点项目20个、工业企业数字化转型示范项目10个，在全省进行重点宣传推广。（省发展改革委、省工业和信息化厅按照职责分工负责）

　　重点培育智慧能源、智慧交通、智慧环保、智慧水务、智慧医疗、智慧教育、智慧养老、智慧农业、智慧物流等应用示范项目。省级每年遴选20个应用示范项目，对于实际投资额（含设备、软件）1000万元及以上的项目，给予200万元一次性补助。（省发展改革委牵头；各州、市人民政府，省直有关部门配合）

　　对首个形成规模商用的数字化应用项目，省级每年遴选10个应用试点，对于实际投资额（含设备、软件）2000万元及以上的，给予200万元一次性补助，并对应用试点项目进行重点宣传推广。（省发展改革委牵头；各州、市人民政府，省直有关部门配合）

　　支持企业开展商贸领域基础设施数字化改造，重点培育数字化特色步行街、智慧商圈建设等应用示范项目。省级每年遴选不超过10个应用示范项目，对于实际投资额（含设备、软件）1000万元及以上的，给予200万元一次性补助。（省商务厅牵头；各州、市人民政府，省直有关部门配合）

　　五、支持创新发展

　　（二十）研发创新补助。支持数字经济企业建设重点实验室、工程研究中心、技术创新中心、产业创新中心、制造业创新中心等，对新获批建设的国家重点实验室给予1000万元一次性补助；对新认定的国家级工程研究中心、技术创新中心、产业创新中心、制造业创新中心，给予500万元一次性补助。已获奖的不再重复给予奖补。（省发展改革委、省科技厅牵头）

　　（二十一）制定标准奖励。对牵头制定云计算、大数据、人工智能、物联网、商用密码等产业发展有关标准的单位或个人，省财政给予经过权威机构认定的国际标准50万元/件、国家标准30万元/件、行业标准20万元/件的奖励。（省市场监管局牵头；省直有关部门配合）

　　六、支持数字化人才培育和引进

　　（二十二）培育数字经济高层次人才。对在我省设立的数字经济类院士工作站，符合科技计划项目资助条件的，给予项目资助。（省科技厅牵头）对经批准新设立的数字经济领域博士后科研工作（流动）站给予资助。（省人力资源社会保障厅牵头）鼓励省内高校开设数字经济有关专业，对新增的有关博士点、硕士点，分别给予一次性奖补不超过300万元、200万元。（省教育厅牵头）

　　（二十三）激发数字经济科技人才创新活力。在省内自然科学、技术发明、科技进步奖等科学技术奖励方面，针对数字经济有关领域成效显著的成果，优先提名云南科学技术奖。（省教育厅、省科技厅、省人力资源社会保障厅按照职责分工负责）

　　此前有关政策与上述政策不一致的，按照“就高不重复”的原则执行。以上支持政策奖补资金，主要用于开展项目改造提升和有关新型基础设施建设。区块链技术应用适用《云南省支持区块链产业发展若干措施》规定的奖励和扶持。

以上支持政策由省发展改革委会同有关部门负责具体解释工作，自印发之日起施行，有效期至2024年12月31日。

（16）中共江西省委、江西省人民政府关于深入推进数字经济做优做强“一号发展工程”的意见

（2022年3月14日）

　　近日，中共江西省委、江西省人民政府印发了《关于深入推进数字经济做优做强“一号发展工程”的意见》。现将意见予以公开发布。

　　为深入学习贯彻习近平总书记关于数字经济发展的重要论述，全面落实党中央、国务院决策部署，深化落实省第十五次党代会精神，牢牢抓住数字经济发展时代机遇，以更高站位、更广维度、更大力度深入推进数字经济做优做强“一号发展工程”，特提出如下意见。

　　一、牢牢把握总体要求，把发展数字经济摆在战略位置来抓

　　（一）重要意义

　　新时代的数字经济发展，日益呈现技术迭代加速化、数据要素市场化、产业布局赛道化、实体融合深度化、市场培育场景化、要素支撑生态化、治理体系现代化的发展趋势。数字经济发展速度之快、辐射范围之广、影响程度之深前所未有，已成为重组全球要素资源、重塑全球经济结构、改变全球竞争格局的关键力量。全省各地各部门务必充分认识到深入推进数字经济做优做强“一号发展工程”，是深入贯彻习近平总书记视察江西重要讲话精神、落实“作示范、勇争先”目标要求的具体行动，是加快构建具有江西特色现代化经济体系、培育壮大发展新动能的必由之路，是加快打造全国构建新发展格局重要战略支点、筑牢未来竞争优势的战略抉择，必须坚持以一以贯之的定力、赶超一流的雄心、敢为人先的胆识、开放包容的思维、科学专业的精神，坚定不移深入推进数字经济做优做强“一号发展工程”，推动我省数字经济高质量跨越式发展。

　　（二）指导思想

　　坚持以习近平新时代中国特色社会主义思想为指导，贯彻落实党的十九大和十九届历次全会精神，深刻把握数字中国建设的战略要求，立足新发展阶段，完整准确全面贯彻新发展理念，服务和融入新发展格局，统筹发展和安全，以推动数字经济高质量跨越式发展为主题，以数据为关键要素，以数字技术与实体经济深度融合为主线，着力推动数字技术创新应用，着力培育产业新赛道，着力赋能产业转型升级，着力加强数字基础设施建设，着力完善数字经济治理体系，推动数字经济成为全省培育壮大经济发展新动能、构建具有江西特色的现代化经济体系的主引擎，为携手书写全面建设社会主义现代化江西的精彩华章作出更大贡献。

　　（三）发展目标

　　力争用5年左右时间，推动数字经济发展迈上新台阶。数字经济增加值增速持续快于全省经济增速、快于全国平均增速，努力实现规模倍增、占全省GDP比重达到45%左右，数字经济核心产业增加值占全省GDP比重达到10%以上，加快打造中部地区数字产业发展集聚区、产业数字化转型先行区、场景创新应用先导区、数字营商环境示范区，努力成为全国数字经济发展新高地。

　　--数字产业发展集聚区。基本建立创新引领、特色鲜明、优势突出的数字产业体系，电子信息产业主营业务收入加快突破1万亿元，形成VR（虚拟现实）、物联网等一批超过1千亿元的细分领域产业集群，培育若干市场占有率全国排名前列的产业赛道，部分优势赛道形成全球领先优势。

　　--产业数字化转型先行区。制造业数字化、网络化、智能化更加深入，两化融合发展指数、生产设备数字化率、装备数控化率力争达到并超过全国平均水平。物流、金融、设计等生产性服务业融合发展加快普及，商贸、文旅、康养、家政等生活性服务业多元化拓展显著加快。农业数字化转型加速推进。新智造、新文创、新零售等跨界融合新业态快速健康发展。

　　--场景创新应用先导区。整合全省资源创机会、供场景，构建多元化、多层次场景体系。基本建立政府引导、市场主导、多方参与的场景建设长效机制，打造一批标杆性应用场景，形成一批新技术新业态新模式，场景建设成为江西数字经济发展的新名片。

　　--数字营商环境示范区。数字经济创新创业活力持续迸发，多层次创投体系不断完善，数字人才加快汇聚。新型基础设施体系基本建成，信息基础设施进入国内先进行列，数据资源开发利用达到全国上游水平。“赣服通”“赣政通”服务水平进一步提高，数字化公共服务更加普惠均等。数字经济相关制度标准规范基本建成，适宜数字经济成长的一流生态基本构建。

　　二、重点实施八大工程，做优做强做大数字经济

　　（一）数字技术创新工程

　　1.加强创新平台建设。大力推进鄱阳湖国家自主创新示范区建设。统筹推进南昌VR、鹰潭智慧、上饶大数据等数字经济领域特色科创城建设，提升南昌高新区国家文化和科技融合示范基地、国家数字出版基地发展能级。实施国家级创新平台攻坚行动，聚焦VR、移动物联网、LED（发光二极管）等领域，力争在创建国家技术创新中心、产业创新中心、制造业创新中心上取得突破，支持更多企业创建国家企业技术中心。聚焦数字经济优势领域细分方向，布局建设省实验室、重组省重点实验室，部署建设一批高水平省级科技创新平台。实施高端研发机构共建行动，全面深化与中科院、中国信通院等大院大所、名校名企合作，共建数字经济领域新型研发机构。

　　2.强化关键技术攻关。梳理数字经济领域关键技术目录，精准实施关键技术攻坚行动。建设省产业链创新联合体和科技协同创新体（院），力争在智能传感器、新型半导体、高密度电路板、光电材料等数字产业化技术上取得新突破，在电控系统、高精密数控机床、工业设备数据采集和相关协议合法兼容、农业智能装备等产业数字化技术上实现新进展。密切跟踪“元宇宙”未来趋势，统筹VR、AR（增强现实）、MR（混合现实）发展，支持突破近眼显示、感知交互、渲染处理等核心关键技术，支撑相关软硬件设备取得新突破。深入推进国家“03专项”成果转移转化，加快突破智能感知、新型短距离通信、高精度定位等物联网产业关键共性技术。统筹谋划布局量子通信、类脑智能、6G等重大前沿技术。实施重大科研攻关项目，形成更多具有自主知识产权的核心技术。

　　3.建设数字人才队伍。制定实施数字经济领域人才专项政策，深入实施院士后备人选支持计划、省“双千计划”、省高层次和急需紧缺海外人才引进计划等各级各类人才计划，加快引进海内外战略型人才、科技领军人才、创新团队，力争引进50名左右数字经济领域“高精尖缺”人才。实施企业家数字素养提升工程，培育新时代“数字赣商”，培养500名左右数字经济领域企业家、创投家。实施新时代“赣鄱工匠”工程和技工教育强基工程，支持省属高校、技工学校、龙头企业等合作共建数字经济产教融合联盟和人才培育基地，培养2万名以上“数字工程师”。

　　（二）产业赛道赶超工程

　　1.布局一批产业赛道。深入研究数字产业细分赛道，聚焦专业芯片、电子材料、电子元器件、半导体照明、智能终端、信创、软件和信息技术服务等基础赛道，VR、“元宇宙”及数字孪生、信息安全和数据服务、物联网、智能网联汽车、无人机等新兴赛道，工业互联网、智慧农业、数字健康、数字文创、智慧家居、智慧能源、数字降碳等融合赛道，“一道一策”推动产业能级跃迁。持续开展数字经济风口研究，对赛道布局进行适时调整、动态更新。

　　2.推进一批重大项目。按照抢占新赛道、补强产业链、塑造新优势的思路，深入实施招大引强“三百工程”、“三请三回”、“三企”入赣，开展“5020”项目攻坚行动，招引一批数字经济重大项目。组织实施数字经济领域年度“项目大会战”，推动重大项目早完工、早投产、早见效。研究建立针对数字经济产业项目特点的服务保障机制，在产业基金、场景供给、数据开放等领域给予“一揽子”支持举措；对具有引领性的重大项目，实行“一事一议”、特事特办。

　　3.培育一批重点企业。紧扣赛道发展方向，以平台化思维推动与头部企业深度对接，以定制化方式赋能独角兽企业市场拓展，以营商环境优化支持中小企业健康成长，培育数字经济企业梯队。推动行业领军企业、国有企业实施平台化发展战略，培育具有国际竞争力的大企业和具有产业链控制力的生态主导型企业。支持高新技术企业加大研发投入，强化资本市场对接，力争在数字经济领域孵化更多独角兽企业。综合采取财政奖补、基金引导、股权投资等方式培育优质中小企业，打造一批数字经济领域“专精特新”企业、制造业单项冠军企业、科技型中小企业。

　　（三）上云用数赋智工程

　　1.推动制造业数字化转型。制定制造业数字化转型实施方案，编制转型路线图，明确转型目标、方向和任务，探索不同行业推进数字化转型的模式和路径。开展“产业大脑”建设应用试点，拓展“5G＋工业互联网”应用，挖掘产线级、车间级5G典型应用场景，加快建设国家工业互联网大数据中心江西分中心，打造3个以上具有全国影响力的行业级工业互联网平台，建成5-10个工业互联网标识解析二级节点，工业互联网标识注册量达到6亿次，培育1000个工业APP。推行国有企业数字化转型“一企一战略”和“一把手”负责制，打造企业数字化转型样板。建设数字化转型促进中心，加快中小企业“上云上平台”，推动大中型企业深度上云，企业上云数突破8万家。深入实施智能制造升级工程，培育2000个以上升级版智能工厂、数字车间。

　　2.推动服务业数字化转型。制定服务业数字化转型实施方案，发展数字生活新服务，推进生产性服务业数字化发展。把发展新文创作为提升赣鄱文化影响力的重要抓手，积极引进腾讯、网易等头部企业数字文创平台，挖掘红色经典、景德镇陶瓷、海昏侯国遗址、汤显祖戏剧等文化时代内涵，发展数字出版、数字影视、数字视听、数字艺术、电竞动漫、沉浸式体验、超高清视频等新业态新模式，引育原创设计、研发制作、IP运营、代理发行企业，打造具有世界影响力的文化IP产品。开展传统商贸云化改造提升，培育智慧商圈，发展以供应链管理、品牌建设、线上线下一体等为特征的新零售，推动电子商务、网络直播等规范健康发展。建设智能仓储和智慧物流体系，推进北斗导航、车联网应用，开展“互联网＋货运物流”模式创新，发展云仓、城市末端共同配送等物流新模式。加快推进绿色金融、普惠金融、科技金融、供应链金融、文化金融等试点示范，推动区块链等技术在支付清算、跨境贸易、金融交易等领域应用。

　　3.推动农业数字化转型。深入推进数字乡村建设，着力补齐城乡数字鸿沟。统筹推进国家和省级数字乡村试点，推动农村数字经济发展和乡村数字治理，加快智慧农业“123＋N”平台建设。培育农业物联网示范基地，深化卫星定位、物联网、大数据、无人机等技术应用，建设基于区块链技术的农产品追溯机制，支持新一代信息技术与农业装备制造业融合发展。深化“互联网＋”农产品出村进城工程，发展农产品直播带货等新模式，进一步提升“生态鄱阳湖·绿色农产品”品牌影响力。培育数字新农人、乡创客，发展休闲农业、创意农业、定制农业等新业态。

　　4.推动开发区数字化转型。加快开发区数字基础设施建设，增强信息网络综合承载能力。建设全省统一的开发区数字化管理服务平台，打造一批“数字开发区”标杆，探索发展跨越物理边界的“虚拟”产业园区和产业集群，加快产业资源虚拟化集聚、平台化运营和网络化协同。发展中央工厂、协同制造、共享制造、众包众创、集采集销等新业态，提升区域制造资源和创新资源的共享和协作水平。

　　（四）“全景江西”建设工程

　　1.统筹谋划应用场景。研究制定全省创新营造数字技术应用场景行动计划，全域构建与数字化发展高度契合的多元应用场景，加快打造“全景江西”。推动各部门、各市县资源整合，以政府数字化转型驱动治理方式变革，推动政务服务、数字治理、数据资源的标准化、共享化、集成化。深化推进政务服务、政府运行、生态保护、市场监管等领域应用场景建设，拓展提升“赣服通”“赣政通”功能，助力打造全国政务服务满意度一等省份。围绕精细智能的数字治理场景，有序推进新型智慧城市、公共安全、应急管理、智慧水利、数字乡村等领域应用场景建设。围绕跨界融合的智能生产场景，深入推进生产制造、金融服务、商贸流通、现代农业等领域应用场景建设。围绕智慧共享的数字生活场景，推进社会保障、医疗健康、教育就业、交通出行、文化旅游等领域应用场景建设。重点打造“智慧交通”“VR＋教育”“VR＋培训”“VR＋医疗”等具体应用场景，支持南昌、鹰潭等地开展智慧交通试点。

　　2.建立供需对接机制。探索“政府搭台、各方出卷、企业答题”模式，推动数字化应用场景与经济社会发展需求有机链接。组织各地各部门、企业分别提出诉求，梳理形成“机会清单”“产品清单”向社会公开发布。搭建线上线下发布平台，举办场景沙龙、对接会等主题活动，将应用场景转化为市场机会。启动创新应用实验室和未来场景实验室建设，为应用场景提供技术支撑和市场验证。完善共建共享机制，鼓励数字经济企业、专业服务机构、科研院所、政府部门组建场景建设联合体，协同开展场景建设。

　　3.加快场景示范推广。组织实施应用场景“十百千”计划，为新技术新产品新业态提供集成应用平台。引导政府部门、企事业单位应用企业创新产品（服务），在全省建设50个左右应用场景示范区、推荐推广300个左右应用场景示范项目、培育3000个左右具有爆发潜力的高成长性企业。

　　（五）空间布局优化工程

　　1.建设创新引领区。发挥南昌省会城市优势，强化创新源、动力源和辐射源作用，建设江西省数字经济创新引领区，争创国家数字经济创新发展试验区。依托南昌VR、电子信息等产业基础和优势，在九龙湖区域建设“元宇宙”试验区，构建以九龙湖区域为核心，以南昌高新区、南昌经开区、南昌小蓝经开区为支撑的“一核三基地”数字经济发展格局。

　　2.建设承接转移示范区。积极对接数字大湾区、数字长三角，主动承接沿海发达地区数字产业梯度转移，创建数字经济承接产业转移示范区。重点支持赣州发挥对接粤港澳大湾区桥头堡和省域副中心城市优势，抓住赣深高铁开通机遇，大力发展电子信息制造业，培育壮大信创、区块链、智能家居等产业，建设粤港澳大湾区数字资源延伸承载地。协同吉安等地打造赣深数字经济走廊。支持上饶大力发展数字文娱、智能制造等产业，打造全国知名网络游戏研发、智能网联汽车生产基地。协同鹰潭等地打造对接G60科创走廊、定向承接长三角数字产业转移的示范区。支持九江、萍乡等地建设产业合作平台，发挥区位交通优势，加强与武汉、合肥、长沙等地在数字经济产业领域的对接合作。

　　3.建设特色产业集聚区。制定数字经济集聚区建设行动计划，鼓励各地依据自身基础和发展条件，明确赛道主攻方向，选择一定区域集中布局、集聚发展，全省统筹建设100个数字经济集聚区。支持赣江新区发挥国家级新区优势，加快吸引知名数字经济企业落户。支持鹰潭打造“智联鹰潭2.0”版，建设千亿级的全链条物联网产业集聚区。支持吉安智能终端制造、九江电子元器件、抚州大数据、景德镇数字文创、宜春智慧新能源、新余智能安防、萍乡智慧旅游等数字经济特色产业集聚发展，打造各具特色、协同发展的产业格局。支持各县（市、区）结合实际，培育壮大数字经济与地方优势产业深度融合的特色产业集聚区，提升县域经济发展水平。

　　（六）数据市场培育工程

　　1.推进公共数据共享开放。加强政务信息化管理，坚持集约共建，切实防止重复建设和资金浪费。建立公共数据目录体系，动态发布数据共享和开放责任清单，推动公共事业单位数据资源统一纳入。依托省电子政务一体化平台，推动省市县三级跨层级、跨部门、跨系统数据共享和开放。完善全省政务数据共享协调机制，探索建立省市县“一地创新、全省受益”的数据共享应用模式，推动数据回流，提供多样化、定制化数据服务。健全公共数据授权开放管理制度，推进公共数据开放利用。开展公共数据创新应用试点示范，举办开放数据创新应用大赛。

　　2.激发社会数据资源活力。探索形成政企数据融合的对接机制，促进政企数据对接融合。探索开展社会数据资源开发利用试点。加快工业数据分类分级试点省建设，探索工业数据分类分级管理，开展电子信息、有色金属、有机硅、生物医药、纺织、家居工业大数据应用试点示范。鼓励企业依法依规运用消费大数据，提升产品和服务质量。

　　3.探索数据要素交易流通。加强数据确权、定价、交易等相关基础规则和制度研究，推动数据标准化、资产化、资本化。按照政策要求，建设省数据交易场所及配套机构，规范数据入场交易。积极引进并培育壮大数据服务商。鼓励设立社会性数据经纪机构，规范开展数据要素市场流通中介服务。争取建立有色、中医药等面向全国的数据交易平台，打造全国性数据市场节点。

　　（七）数字基建支撑工程

　　1.全面升级通信网络基础设施。全面推进“双千兆”建设，推动5G网络城市和乡镇全面覆盖、行政村基本覆盖、开发区等重点应用场景深度覆盖，推动千兆光纤网络逐步向行政村延伸覆盖，家庭用户普遍具备千兆接入能力、大型企业机构具备万兆接入能力。加快部署基于IPv6（互联网协议第6版）的下一代互联网，全力推进南昌国家级互联网骨干直联点建设，提升骨干网络和省际出口承载能力。建设窄带物联网（NB-IoT）、4G和5G协同发展的移动物联网综合生态系统。推进卫星互联网、车联网等基础设施建设。

　　2.统筹建设数据智能基础设施。实施数据中心等新型基础设施绿色高质量发展行动计划，落实构建全国一体化大数据中心协同创新体系要求，以“一核四副两备”（南昌为核心，九江、上饶、赣州、宜春为补充，抚州、鹰潭为备份的数据中心空间布局）为重点，科学合理布局全省数据中心建设，积极对接“东数西算”工程。推进数据中心从存储型向计算型升级，推动数据中心与多元算力协同发展，加快构建“云、边、超、智”多元协同数网融合的算力体系。加快对效益差、能耗高的小散数据中心进行整合和绿色节能升级改造，新建大型数据中心电能使用效率值控制在1.3以下，持续提升数据中心可再生能源利用水平。

　　3.稳步发展融合应用基础设施。高效布局人工智能基础设施，提升支撑“智能＋”发展的行业赋能能力。加快工业互联网标识解析节点建设。重点发展协同便捷的智慧交通设施，支持智慧公路、港口、机场等建设。推动智慧电网、智慧管网、智能充电桩等建设，加快建设泛在电力物联网、能源大数据中心，构建多能协同的能源数字管理平台、多元融合高弹性能源互联网。推动智慧生态、水利等基础设施建设，构建生态环境数字化监测机制，优化环境治理与灾害应急设施支撑。

　　（八）开放合作共赢工程

　　1.深化与头部企业合作。瞄准世界500强、大型跨国企业、全国电子信息百强、软件百强、互联网百强等企业和顶级研究机构，聚焦产业链关键环节，开展靶向招商、精准招商和补链招商。“一企一策”实施头部企业落地计划，招引国内外领军企业在赣设立区域总部、研发总部、业务总部、平台总部。纵深推进省政府与阿里巴巴、华为、腾讯、网易、商汤、中兴、中国电子等战略合作协议，将准头部、潜在独角兽企业总部作为招商重点。创新合作共赢模式，引入“事业合伙人”机制，共同建设产业平台、研发项目、数字人才培训基地。深化与中国电信、中国移动、中国联通等战略合作，推动江西成为基础电信企业转型升级的先导区。

　　2.夯实对外开放合作平台。积极参与数字丝绸之路建设，加快跨境电子商务综合试验区等建设，发展数字贸易等服务贸易新业态。高水平举办世界VR产业大会等重大活动，打造数字经济全球重要会展和高端对话平台。深度参与长江中游城市群合作发展，推动新型基础设施共建共享。

　　三、创新制度供给，营造数字经济发展一流生态

　　（一）改革创新政策

　　1.健全制度标准。加快推动数字经济领域地方立法进程，为统筹推动数字经济发展提供法治保障。鼓励企业、科研院所、行业协会等积极参与两化融合、VR、移动物联网、大数据等重点领域的行业标准和国家标准制定。

　　2.构建包容环境。坚持市场主体平等准入，破除“隐性壁垒”。开展“一照多址”“集群注册”等登记改革，深化“宽入严管”的商事制度改革，推行“容缺审批＋承诺制”。探索建立应用场景建设容错纠错机制，实施“包容期”管理，依法审慎开展行政执法。

　　3.创新监管模式。深化信用监管、“互联网＋监管”新模式运用，建立触发式监管机制，探索“沙盒监管”措施，实现事前事中事后全过程全领域监管。对新业态新模式设置一定的观察期，预留发展空间。对存在较大潜在风险的，依法依规严格监管。对触及法律法规红线的，坚决依法予以取缔。加强反垄断、反不正当竞争监管，防止资本无序扩张，推动平台经济健康有序发展。强化数据等知识产权保护。

　　4.强化安全保障。强化数据安全管理，做好数据备份。加强数据处理活动的风险监测评估，强化利用公民个人信息从事商业活动的监督和约束，加强对违法违规收集使用公民个人信息行为的打击治理。推广应用区块链、数据安全沙盒、隐私计算等技术，保障数据隐私和数据安全。健全网络安全保障工作机制，强化网络完全主体责任，落实网络安全等级保护2.0、关键信息基础设施安全保护、涉密网络分级保护、商用密码应用安全评估等要求。

　　（二）人才专项政策

　　1.引进高水平数字人才。制定数字人才需求目录，列入全省急需紧缺人才引进指导目录。制定“高精尖缺”数字经济引进人才政策，在研发、落户、住房、医疗、配偶就业、子女就学等方面给予更具吸引力的政策。保障和落实用人单位自主权，探索给予行业主管部门直接举荐权。鼓励高校、科研院所自主认定数字经济高层次人才，采取年薪制、协议工资等办法自主决定薪酬水平。开展赣籍数字经济人才回归专项行动，建立赣籍数字经济人才联盟。

　　2.培育“新职业”人才。进一步落实高校专业设置自主权，支持高校设置数字经济类学科专业和开设新一代信息技术产业学院，鼓励学科交叉融合，在新增招生指标等方面予以倾斜。提升本土高校数字经济领域毕业生留赣比例。支持企业设立博士后工作站。落实国家产教融合建设试点省有关政策，建设具有全国影响力的数字经济实训基地，发展订单制、现代学徒制等多元化人才培养模式，加大赣鄱“数字工程师”培养培训力度。

　　3.激发数字人才活力。持续推进科技计划项目“包干制”、赋予科研人员职务科技成果所有权或长期使用权等改革试点。构建以创新能力、质量、贡献、绩效为导向的人才评价机制，优化技能型人才多元化评价方式。对基础研究人才、青年人才适当延长评价考核周期。完善职称评聘制度，畅通特殊优秀人才高级职称直接申报渠道。探索竞争性人才使用机制，面向市场遴选重大科技攻关专项首席专家，实行首席专家负责制。

　　4.优化数字人才发展环境。落实党委联系服务专家制度。优先推荐符合条件的数字经济领域高层次人才申报国家级和省重大人才工程。鼓励人才创新创业引导基金优先支持数字经济领域人才创新创业。健全新业态灵活就业者社会保障、权益保障、职称评价等政策制度，完善适应新职业、新工种变化的人才培养、职业技能等级认定、职业行为规范等相关政策措施。

　　（三）融资支持政策

　　1.设立专项基金。依托省发展升级引导基金，专门设立省数字经济发展子基金，重点支持重大项目建设和高成长、初创型数字经济企业发展。鼓励社会投资人和基金管理团队共同参股设立各类基金，支持社会资本来赣发展风险投资、天使投资、创业投资。

　　2.支持企业上市。支持数字经济企业通过股改、并购重组等方式对接资本市场，推动数字经济企业登陆多层次资本市场进行融资。建立省数字经济企业上市挂牌后备资源库，对符合条件的新增首发上市企业按照相关规定给予上市奖励。

　　3.创新金融产品。强化金融产品和服务创新，提供个性化、精准化信贷和保险服务。开发数字经济领域科技融资担保、知识产权质押融资、数据资产质押等产品和服务，创新“云量贷”“人才贷”“数字化转型贷”“文企贷”等产品，用好“财园信贷通”“科贷通”等支持数字经济发展。支持符合条件的数字经济企业通过发行债券进行融资。引导支持保险机构开发科技保险产品。

　　（四）财税支持政策

　　1.加大财政支持力度。完善财政投入机制，统筹省级相关资金，积极争取国家资金，加大对数字经济发展重点场景、重大平台、重大项目及试点示范等支持力度。发挥政府采购政策作用，推动数字经济创新产品和服务率先在政务领域应用，支持装备首台套、材料首批次、软件首版次示范应用。

　　2.落实税收优惠政策。落实高新技术企业、小微企业、软件和集成电路设计等各项税收扶持政策，以及研发费用加计扣除和固定资产加速折旧等政策。全面落实减税降费系列政策，进一步降低数字经济企业成本。落实数字经济领域高端紧缺人才个税相关优惠政策。

　　（五）要素支撑政策

　　1.强化数据支撑。探索建立以数据应用渗透率、数据质量、数据价值转化为重点的指标体系、统计体系和考核体系。编制政务数据开放对接清单，推动促进各地各部门向社会开放数据资源。探索构建数据产品和服务价格形成机制、收益分配机制。探索公共数据授权运营制度，依法允许医疗、交通、金融等特定领域公共数据授权特定机构进行开发利用。

　　2.加强土地保障。对纳入国家重大项目、省级以上单独选址项目清单的数字经济发展领域重大建设项目，使用国家配置用地计划指标。在规划许可的前提下，鼓励各地积极盘活商业用房、工业厂房、企业库房、物流设施和家庭住所、租赁房等资源，为数字经济主体提供低成本办公场所和居住条件。

　　3.降低用能成本。推动能耗、碳排放指标向符合国家产业方向、单位能耗产出效益高的数字经济产业项目倾斜。支持有条件的企业参加电力市场化交易。开通数字技术企业电力接入绿色通道，优先保障数字经济园区、企业电力接入。

　　四、加强组织保障，完善数字经济治理机制

　　（一）强化组织领导。成立江西省发展数字经济领导小组，高位推动全省数字经济发展。成立省数字经济办，设在省发改委。成立数字经济专家委员会，加强对数字经济前沿技术和发展风口研判。组建数字经济研究院、企业家俱乐部、业态共治理事会等支撑性机构，为数字经济发展提供决策咨询。各地各部门要把深入推进数字经济做优做强“一号发展工程”作为“一把手”工程，建立推进机制，形成齐抓共管、协同高效的工作格局。各级党校（行政学院）将数字经济知识作为重要培训内容，切实提高领导干部专业能力、工作人员数字素养。

　　（二）强化工作合力。省数字经济办要切实履行好牵头抓总职责，承担起牵头组织、统筹协调、调度推进、考核评价等具体职责。各有关部门要根据自身职责和任务清单，各司其职、各负其责，共识共为、密切配合，落实好各项重点工程和政策举措。市县要同向同为，凝聚起发展数字经济的强大合力。

　　（三）强化示范引领。积极争取国家各类创新试点在赣布局，在争创国家级数字经济创新发展试验区、人工智能创新发展试验区、数据要素市场化配置改革、数字服务出口基地、智能社会治理实验基地、区块链创新应用试点、IPv6应用等试点示范上取得突破，推动打造国家“03专项”试点示范升级版。统筹推进省内重点领域试点示范，鼓励各地各部门在场景培育、数据开放、产业集聚等方面先行先试，形成一批可复制、可推广的典型案例。

　　（四）强化考核激励。在国家数字经济及其核心产业统计分类的基础上，构建江西数字经济指标统计机制。建立健全数字经济运行监测机制，定期发布数字经济运行监测分析报告，每年发布全省数字经济白皮书。强化各地发展数字经济考核，进一步调动部门、市县、开发区等积极性、主动性、创造性。充分运用各类媒体，加大数字经济领域新技术、新业态、新模式宣传力度，及时宣传先进经验，提升全民数字素养与技能。

（17）江苏省人力资源社会保障厅、江苏省委网信办、江苏省发展改革委、江苏省工信厅、江苏省财政厅、江苏省工商联关于印发《关于实施数字技能提升行动服务数字经济强省战略的指导意见》的通知

苏人社发〔2022〕26号

各设区市人力资源社会保障局、市委网络安全和信息化委员会办公室、发展和改革委员会、工业和信息化局、财政局、工商业联合会：

　　现将《关于实施数字技能提升行动服务数字经济强省战略的指导意见》印发给你们，请结合本地工作实际，认真贯彻执行。

　　附件：关于实施数字技能提升行动服务数字经济强省战略的指导意见.pdf

为贯彻落实党中央、国务院和省委、省政府关于数字经济发展的战略部署，适应数字产业化、产业数字化新要求，打造高质量数字技能人才队伍，根据人社部《提升全民数字技能工作方案》《江苏省“十四五”数字经济发展规划》以及我省提升全民数字素养与技能有关工作安排等，组织实施数字技能提升行动，更好服务数字经济强省战略，现提出如下意见。

一、总体要求

聚焦我省30条优势产业链、10条卓越产业链，在生物医药、大数据、人工智能、区块链、集成电路、物联网、信息技术应用创新、高端装备、智能电网、节能环保、工程机械等重点领域，通过三年的努力，建设集培训、评价、选拔、使用、激励于一体的数字技能人才培育机制，市场主导、共建共享的数字技能资源供给体系，多层次、高效协同的数字技能重点攻关平台，充分释放数字技能要素价值，促进技能链、人才链与产业链、创新链深度融合，赋能智能化改造、数字化转型，着力打造全国数字技能创新发展高地，为谱写“强富美高”新江苏建设现代化新篇章提供强大数字技能支撑和人力资源保障。

二、行动内容

1.大力推进“技能中国行动”在江苏落地见效，聚焦优势产业链、卓越产业链，集中资源、集中力量、集中政策，组织开展“江苏工匠”培育工程和职业技能提升行动、技工院校提升行动、世界技能大赛夺金行动。依托全省人力资源社会保障系统一体化信息平台，加快建设完善全省职业技能培训服务管理信息化系统，定期发布数字技能类职业（工种）就业、职业培训和岗位需求信息，优化数字技能公共服务，推动数字技能创新要素加速集聚、人才队伍加快成长。

2.支持企业将数字技能人才队伍建设上升为企业发展战略。联合工信部门等建立全省“数字工匠”培育库，采取订单式、定向式和项目制等方式，推动数字经济职业技能培训，以智能制造、工业互联网等工业和信息化企业一线从业人员为重点，广泛组织开展数字技能岗前培训、在岗培训和转岗转业培训等，增强产业工人数字技能素质。多措并举组织数字技能交流活动。每年将数字技能相关职业（工种）高级技师纳入省级高级技师岗位技能提升培训范围。加强与发达国家在数字技能领域的交流互鉴，适时举办数字技能海外研修班。

3.采用政、企、校合作的形式，着眼长三角一体化发展，联合龙头企业和院校，推动各地打造一批功能突出、资源共享的区域性数字技能公共实训基地，在加快制造业数字化转型、服务业数字化发展、促进农业数字化提升中，有针对性地提高数字技能人才的供给数量和质量。面向智能制造、工业互联网、大数据和区块链等领域，力争建设10个省级数字高技能人才专项实训基地；争取培育20个数字技能领军人才领办的省级技能大师工作室，加强堵点、难点、重点职业（工种）技能的原创研究和联合攻关，打造一批突破性技能创新成果转化应用示范，开发推广性应用场景。

4.强化与数字企业的深度合作，积极开发适应数字经济发展要求的新职业（工种）培训资源，引导数字技能培训资源向市场急需、企业生产必需和劳动者必备等环节集中。指导数字经济产业集中的地区开发一批当地产业急需的数字职业技能培训项目。实施“互联网+职业技能培训计划”，开展线上职业技能培训，遴选推荐一批优质线上培训平台，推出100个以上数字技能培训课程资源，每年开展线上培训10万人次以上。健全以技能需求和技能评价结果为导向的培训补贴政策，将数字技能类职业（工种）纳入政府补贴性职业技能培训范围，列入高技能人才培训补贴紧缺型职业（工种）目录，按规定给予培训补贴。全省每年新增数字技能人才10万人。

5.充分发挥技工院校职业培训功能，面向社会提供线上线下培训服务，以数字技能普及性培训为突破口，适应智能车间、智能工厂建设的要求，积极承担企业新型学徒制培训、就业技能培训、岗位技能提升培训和创业培训等多样化任务。指导支持职业培训机构针对数字产业从业人员建立完善培训意愿识别和能力短板诊断机制，推行“技能培训+就业服务”全链条模式，提升衍生服务能力，提高培训精准度和实用性，促进数字技能人才就业创业。加速数字技能培训迭代进程，利用数据要素、数字技术加快平台化、定制化、轻量化培训模式创新，推动培训上云、上平台，营造便于劳动者终身学习的技能培训创新生态。

6.鼓励技工院校在专业设置、师资培养、招生规模等方面向数字人才倾斜，将数字技能教学情况及成效作为评估技工院校办学质量的重要指标，建设一批服务数字经济发展、契合当地数字产业特点的技工院校。完善工学交替“双元制”技能人才培养模式，鼓励和支持技工院校与龙头企业、品牌企业共建数字技能实习实训基地，共同开发数字技能课程、教学资源，共同开展企业新型学徒制培训。

7.省重点技师学院普遍建立数字技能类品牌特色专业群，培育5所数字技能教学资源开发应用突出的省级高水平技工院校。鼓励和支持技工院校开设产教融合、校企合作的数字技能专业，适当扩大相关专业办学规模，集聚社会资源，联合开设数字技能“订单班”“冠名班”等。创建10个省级数字技能一体化教学名师工作室。依托技工院校师资研修中心组织开展数字技能师资研修培训，将数字技能作为校长和师资培训的重点内容。在技工院校教师职业能力大赛中设置数字技能类赛项。

8.围绕数字产业衍生的新职业、新技能、新工艺，以技能人才评价技术资源快速响应机制为抓手，每年至少开发10个数字技能类评价标准（规范）或题库。加快培育数字技能类行业组织、用人单位备案成为职业技能等级认定机构，有序开展数字技能人才评价。指导技工院校根据相关专业设置拓展数字职业（工种）评价范围。支持企业结合生产经营特点和实际需要，自主确定数字技能类评价职业（工种）范围，自主设置数字技能岗位等级，自主开发制定数字技能评价标准规范，自主运用评价方法，自主开展数字技能人才评价。

9.加强数字技能类高技能人才与专业技术人才职业发展贯通，探索推进数字产业领域职业资格、职业技能等级与专业技术职称有效衔接，探索开展数字技能类国（境）外职业技能比照认定，加快培育既具备本领域专业素质，又掌握数字技能的复合型“数字工匠”。

10.推动各行各业广泛开展群众性数字技能岗位练兵比武活动。支持地方政府、行业部门（协会）、龙头企业、职业（技工）院校等组织开展人工智能、智能制造类数字技能大赛，对省级数字技能类大赛或省级技能大赛中设置数字技能项目的，优先列入省级一类职业技能竞赛，并给予相应的激励支持措施。支持南京、无锡、苏州、常州等数字产业相对集中的城市围绕软件和信息服务、智能电网装备、物联网等国家先进制造业集群，举办数字职业技能大赛，以大赛引人才、招项目、聚资本。

11.加大对数字技能人才的评比表彰力度，重点引导产业链链主企业设立数字技能首席技师，优先试点评聘数字技能特级技师。支持数字经济龙头企业引进数字技能类高技能人才。优先推荐数字技能类高技能人才参评中华技能大奖、全国技术能手、国务院政府特殊津贴专家和江苏大工匠、江苏工匠、江苏省技术能手、江苏省企业首席技师、江苏省有突出贡献中青年专家、江苏省“双创计划”、江苏省“333高层次人才培养工程”等。

12.试点建设数字技能人才服务产业园，以创新策源和成果转化为重点，统筹开展技能成果展示、技能标准研发、技能人才培训、技能人才评价、技能人才服务等活动，做大数字技能载体量级，做强数字技能服务能级，提高数字技能人才工作的集成度和显示度，吸引更多社会力量和资源要素支持数字技能人才队伍建设。

三、保障措施

一是强化组织协调。深入学习贯彻习近平总书记关于建设网络强国、数字中国、智慧社会的战略部署，认真落实省委、省政府全面提升数字经济发展水平的要求，将实施数字技能提升行动列入各地就业工作领导小组的重要议题和重点任务，建立和完善本地工作推进机制，细化目标，项目化推进，实化举措，清单式管理，采取有效方式和管用措施，加强对任务落实情况的跟踪监测和成效评估，注重示范引领，打造工作品牌，确保各项任务落实落地。

二是加大推进力度。以《江苏省就业促进条例》的颁布实施为契机，聚焦政策设计、载体建设、供需对接、人才生态等关键环节，研究制定适应数字经济发展特点的技能人才政策，鼓励将数字技能人才纳入各类就业、培训、人才计划支持范围，积极探索高效灵活的数字技能人才培养、评价及激励举措，加大数字技能人才工作投入力度，强化全流程绩效评估，为技能劳动者营造更好的成长成才环境。

三是凝聚工作合力。构建由人社、发改、工信、财政、网信、工商联等部门、单位共同参与、联动推进的工作格局，以共识促合力，以任务聚资源，统筹数字技能提升行动中的政策制定、工作协调、监督检查等重大事项，综合采取跨部门、跨领域的政策手段，积极探索促进数字技能发展的改革举措，形成一批可复制推广的经验做法和制度性成果。

四是突出宣传引导。突出“数字技能，智慧工匠”的主题，大力弘扬数字时代劳模精神、劳动精神、工匠精神，选树一批数字技能融合赋能的标杆企业、优秀工匠、典型模式，运用多种形式、多种手段，强化示范引导、政策解读和工作宣传，扩大数字技能提升行动的影响力和知晓度，及时发布工作进展和成果成效，讲好数字技能提升行动中的“好故事、活经验”，营造全社会共同关注、积极参与数字技能提升行动的浓厚氛围。

江苏省人力资源和社会保障厅

中共江苏省委网络安全和信息化委员办公室

江苏省发展和改革委员会

江苏省工业和信息化厅

江苏省财政厅

江苏省工商业联合会

2022年2月25日

（18）黑龙江省工业和信息化厅关于印发《＜推动“数字龙江”建设加快数字经济高质量发展若干政策措施＞》实施细则(试行)的通知

黑工信信软联发[2022]25号

《推动“数字龙江”建设加快数字经济高质量发展若干政策措施》实施细则（试行）

第一章 总则

第一条 为贯彻落实省委省政府关于发展数字经济的决策部署，有效发挥政府资金引导作用，加快推动数字经济高质量发展，依据《黑龙江省人民政府关于印发推动“数字龙江”建设加快数字经济高质量发展若干政策措施的通知》（黑政规〔2021〕14号）（以下简称《若干政策措施》）政策规定，制定本细则。

第二条 本细则所称奖补资金是指由省财政厅统筹安排，用于支持制造业数字化转型、中小企业数字化赋能、其他重点行业和领域数字化发展、引进培育数字产业企业、支持工业互联网平台建设及应用、加快发展壮大数字产品制造业、大力发展软件和信息技术服务业等方面的省级财政资金。

第三条 本细则所称省级主管部门是指《若干政策措施》各项政策条款中位列第一的责任单位，相关责任部门是指位列第一以后的责任单位，不包括各市（地）人民政府（行署），有单独说明的除外。

第四条 本细则所称申报单位是指符合条件的企业、事业单位、融资担保机构、产业园区（基地）以及产业数字化或数字产业化项目牵头单位等。

第二章 支持范围、方式和标准

第五条 落实《若干政策措施》坚持普惠制原则，凡达到政策规定条件的申报单位，一律公平、公开、公正地享受政策支持。

第六条 奖补政策分为免申即享、认定奖励和据实补助三类。

（一）免申即享类

1.获得工信部工业互联网领域专项资金支持项目的牵头单位，省级财政按照中央支持额度的20%给予补助，最高不超过100万元。

2.获得工信部工业互联网、大数据、5G、信息消费等试点示范牵头单位，奖励50万元。

3.对履行统计填报义务、营业收入首次达到10亿元的数字产业企业，省级财政一次性奖励企业核心团队500万元。

4.对通过国家评估、接入20家以上企业的工业互联网标识解析二级节点，省级财政给予建设单位一次性补助500万元。

5.对新纳入规上统计且履行统计填报义务的软件和信息技术服务业、互联网和相关服务业企业，省级财政一次性奖励50万元。

6.对通过软件能力成熟度（CMMI）评估且获得3级及以上等级评定的软件和信息技术服务业、互联网和相关服务业企业，省级财政给予一次性分档奖励，最高不超过150万元。

（二）认定奖励类

1.省级主管部门每年认定不超过50个工业互联网新模式新业态示范应用项目，省级财政给予一次性分档奖励，最高不超过200万元。认定标准详见附件1。

2.经省级主管部门认定的具有标杆示范作用的服务中小企业云平台企业，按照服务企业数量，省级财政给予分档奖励，最高不超过500万元。认定标准详见附件2。

3.省级主管部门每年认定50户中小企业数字化示范标杆企业，省级财政对每户企业一次性奖励50万元，对其中省级“专精特新”中小企业一次性奖励100万元。认定标准详见附件3。

（三）据实补助类

1.对同时满足下列三个条件的项目，省级财政按项目实际完成投资额的10%给予补助，单个项目最高不超过600万元：符合《黑龙江省重点数字产品指导目录》规定范围、投资额（设备和软件）2000万元及以上、在申报年度内已全部建成投产或分期投产。认定标准详见附件4。

2.对首次购买应用黑龙江省行政区域内首版次软件创新产品的企业，省级财政按产品实际销售价格（需实际到账且取得发票）的20%给予补贴，单个产品补贴最高不超过50万。认定标准详见附件5。

3.对经过验收、达到智慧矿山标准的非煤矿山示范项目，省级财政结合年度预算安排，按照不超过项目总投资20%的比例给予一次性补助，单个项目最高不超过1000万元。认定标准详见附件6。

4.建立数字经济核心产业企业贷款担保风险补偿机制，对担保机构发生的贷款担保代偿损失，按照一定额度和比例给予风险补偿。管理办法详见附件7。

第七条 申报单位应满足以下基本条件

（一）在黑龙江省行政区域内依法登记注册

（二）具有独立法人资格

（三）主体、主营业务及纳税均在黑龙江省

（四）不属于失信被执行人且未列入经营异常名录

（五）省级主管部门规定的其他条件

第八条 项目申报需满足不重复原则

（一）申报单位的同一项目在同一年度内只能享受一项奖补资金。

（二）申报单位已获得奖补资金的同一项目，之后的年度不得重复申报。

第三章 兑现流程

第九条 启动兑现。省级主管部门会同相关部门按年度组织开展《若干政策措施》政策兑现。以印发正式通知的形式明确当年启动时间、工作安排和具体要求。

第十条 组织申报

（一）免申即享类

原则上不需要申报单位提交申报材料，省级主管部门另有规定的除外。

（二）认定奖励类、据实补助类

各市（地）主管部门负责组织本辖区内符合条件的企业属地化申报，指导企业按照要求准备申报材料，对企业申报材料进行形式审查并提出推荐意见，汇总本级和所辖县（市，区）相关材料，报送至省级主管部门并抄送同级财政部门。申报材料不作为最终支持依据。

第十一条 开展评审

（一）免申即享类

省级主管部门依据有关文件和行业数据形成评审结果，会同省级财政部门拟定资金分配方案。

（二）认定奖励类

省级主管部门依据相应认定标准组织开展评审，可通过成立专家审核组、委托第三方机构等方式进行，必要时可进行实地评审。专家审核组、第三方机构等应出具审核报告，给出评审结果。省级主管部门会同省级财政部门，根据评审结果拟定资金分配方案。

（三）据实补助类

省级主管部门会同省级财政部门组织开展评审，可通过成立专家审核组、委托第三方机构等方式进行，必要时可进行实地评审。专家审核组、第三方机构等应出具审核报告，给出评审结果。省级主管部门会同省级财政部门，根据评审结果拟定资金分配方案。

第十二条 网上公示

省级主管部门根据评审结果以及相关责任部门意见，确定拟支持对象名单并在其官方网站进行公示，公示期一般不少于5个工作日，确因需要可按规定延长。公示期间有异议的，省级主管部门应会同相关责任部门及时核实处理。

第十三条 请示报批

公示期满无异议后，拟支持对象名单提交省级主管部门党组会议审议。审议通过后，由省级主管部门会同省级财政部门履行报省政府审批程序。

第十四条 拨付资金

经省政府批准同意后，省级财政部门按照规定程序下达奖补资金，同步分解下达经省级主管部门确认的绩效考核指标。各市县财政部门应会同同级主管部门按规定时限和要求拨付奖补资金。

第四章 监督管理与绩效考核

第十五条 建立监督管理机制。申报单位、省级主管部门、财政部门等应分别履行以下相应职责。

（一）申报单位应对其提交申报材料的真实性、准确性负责并承担申报主体责任，自觉接受并积极配合省级主管部门及相关责任部门的监督管理。

（二）省级主管部门牵头负责《若干政策措施》政策兑现的组织实施，对申报单位承担的国家级或省级重大项目依法依规履行监管职责；省级财政部门负责统筹做好政策资金保障和分解下达。

（三）参与评审的专家、第三方机构等应对评审结果负责并承担相应的法律责任。

第十六条 建立绩效目标考核制度。严格落实《中共黑龙江省委 黑龙江省人民政府关于全面实施预算绩效管理的实施意见》有关要求。省级主管部门负责设定绩效目标并细化分解项目绩效目标任务。各市县负责对照绩效目标做好绩效运行监控和绩效评价工作，确保绩效目标如期实现。健全绩效评价结果反馈制度和绩效问题整改责任制，加强绩效评价结果应用。

第十七条 任何单位和个人不得以任何理由截留、挤占或挪用专项资金。申报单位提供的材料应当客观、真实、准确，对以提供虚假材料、虚报指标等行为获取财政扶持资金的，一经发现，取消申报资格，追回专项资金，不再列入今后省级各项政策支持范围，并将依据有关法律、法规追究申报单位及相关人员责任。

第五章 附则

第十八条 本细则由《若干政策措施》各项政策条款中责任单位按职责分工分别负责解释。

第十九条 本细则自印发之日起施行，有效期4年，期间如有变化，将按规定作出调整。

附件：1.黑龙江省工业互联网新模式新业态示范应用认定标准（试行）

2.黑龙江省具有标杆示范作用工业互联网平台企业认定标准（试行）

3.黑龙江省中小企业数字化示范标杆企业认定标准（试行）

4.黑龙江省重点数字产品建设项目认定标准（试行）

5.黑龙江省首版次软件创新产品市场化应用认定标准（试行）

6.黑龙江省（非煤）智慧矿山认定标准（试行）

7.黑龙江省数字经济核心产业企业融资担保贷款风险代偿补偿资金管理暂行办法

附件1黑龙江省工业互联网新模式新业态示范应用认定标准（试行）

为贯彻落实《推动“数字龙江”建设加快数字经济高质量发展若干政策措施》，支撑开展工业互联网新模式新业态示范应用（以下简称示范应用）的认定，制定本标准。

一、认定范围

开展智能化制造、网络化协同、个性化定制、服务化延伸、数字化管理、安全化生产等工业互联网应用的工业企业（以下简称企业），行业范围包含但不限于装备、石化、食品、原材料、医药、电子信息、民爆等。

二、基本条件

企业应满足《<推动“数字龙江”建设加快数字经济高质量发展若干政策措施>实施细则（试行）》（以下简称《实施细则》）第七条所规定的基本条件。

三、评价内容及认定标准

示范应用主要从基础能力、示范内容、产生效益和示范效应四个维度进行认定，共包括十一个方面的评价内容和认定标准。认定标准应符合任意一项是认定的基本（最低）标准，最终认定结论由认定标准中符合高标准项或同时符合多项标准得出。

（一）基础能力

1.网络覆盖

评价内容：企业应建立较为完善的工业互联网网络体系，厂区特别是生产区域已实现较大范围网络覆盖，网络带宽能够满足日常生产经营活动需要，具体如下：

一是主干网络覆盖率达到80%以上。

二是生产区域内工业通信网络覆盖率达到70%以上。

三是网络带宽达到100MB/s以上。

认定标准：以上评价内容中，需至少符合任意1项。

说明：工业互联网网络体系包括网络互联、数据互通和标识解析三部分。主干网络是企业内所有通信、信息化系统连接的承载体，负责本地网络的贯通及本地网络与广域网的连接。工业通信网络是指现场总线、工业以太网、工业无线网络等。

2.生产设备

评价内容：企业应广泛采用数字化生产设备，已开展数字化生产设备联网，能够解析主流的工业互联网协议，具体如下：

一是数字化生产设备数量占全部生产设备比例不低于70%。

二是已联网数字化生产设备占全部数字化生产设备的比例不低于80%。

三是能够解析OPC通讯协议、Modbus通讯协议、ProfiBus通讯协议等主流工业互联网协议中的任意一种。

认定标准：以上评价内容中，需至少符合任意1项。

说明：数字化生产设备是指具备自动信息采集功能的生产设备。

3.安全保障

评价内容：企业应建立较为完善的工业信息安全管理机制，围绕工业互联网建设及应用开展安全防护、风险评估、数据管理等工作，具体如下：

一是已部署生产设备和工业控制系统安全防护措施。

二是在内外网改造过程中落实有关安全标准并进行工业互联网安全评估。

三是对于产品数据、研发设计数据、生产数据、经营管理数据等采取必要的数据规范管理措施并实施管理。

认定标准：以上评价内容中，需至少符合任意1项。

4.企业上云

评价内容：企业应基于工业互联网平台，推动设备上云和业务上云，具体如下：

一是实现上云的工业设备数量占企业工业设备总数量的比例应不低于30%。

二是基于云上部署的系统开展研发设计、经营管理、生产管理、供应链管理、市场营销、用户服务等业务。

认定标准：以上评价内容中，需至少符合任意1项。

说明：实现上云的工业设备是指实现与工业互联网平台边缘端或云端连接并能够进行数据交换的工业设备。

5.创新能力

评价内容：企业应围绕工业互联网建设及应用，不断提升创新能力，具体如下：

一是入选国家或黑龙江省产业数字化试点示范。

二是已接入黑龙江省内工业互联网标识解析二级节点并开展标识解析应用。

三是通过两化融合管理体系贯标评定并获得证书。

认定标准：以上评价内容中，需至少符合任意1项。

说明：产业数字化试点示范包括5G、工业互联网、工业大数据、企业上云、数字化（智能）车间、智能工厂、中小企业数字化标杆等。

6.资金投入

评价内容：企业应围绕工业互联网建设及应用，在认定的上一年度开展工业互联网相关投入，具体如下：

一是将工业互联网相关投入纳入年度财务预算并进行统筹安排。

二是设立企业级工业互联网专项资金并对资金使用进行全流程跟踪管理。

认定标准：以上评价内容中，需至少符合任意1项。

说明：工业互联网相关投入是指企业围绕工业互联网建设及应用，在内外网改造升级、设备联网改造、解决方案购买与实施、工业APP或工业微服务订阅、软硬件采购、培训宣贯等方面的资金投入。

7.数据规范

评价内容：企业应基于工业互联网标识解析体系实现数据联通、标准统一和真实可靠，具体如下：

一是基于标识解析体系开展数据分类汇聚与梳理，形成数据资产。

二是基于标识解析体系统一标准，实现各生产要素数据贯通。

三是基于标识解析体系形成数据挖掘和分析能力，实现数据可视化。

认定标准：以上评价内容中，需至少符合任意1项。

（二）示范内容

示范内容分为智能化制造、网络化协同、个性化定制、服务化延伸、数字化管理、安全化生产六类，单个企业应符合其中的一类。

1.智能化制造

评价内容：企业综合运用互联网、大数据、人工智能等新一代信息技术，实现材料、设备、产品等生产要素与用户之间的在线连接和实时交互，逐步实现机器代替人生产，具体如下：

一是生产排程柔性化。基于工业互联网系统解决方案开展原料采购与市场订单管理、生产计划优化与排产、生产调度优化与排产三个业务环节应用实践，应用多种智能算法提高生产排程效率，实现柔性化生产，满足多品种、小批量的订单需求。

二是生产作业数字化。基于工业互联网系统解决方案开展工单派放管理、工艺参数调优、过程控制执行三个业务环节应用实践，实现生产任务基于生产计划自动生成，关键工序自动控制实现率90%以上的。

三是过程质量可追溯。基于工业互联网系统解决方案开展质量策划、质量控制、质量改进三个环节应用实践，推动质量管理全流程优化，实现质量正向逆向全程可追溯，产品检验覆盖率达到100%。

四是生产设备自管理。基于工业互联网系统解决方案开展设备监控、设备诊断、设备预测性维护、设备自适应控制四个环节应用实践，提升生产设备数字化管理水平，实现对90%以上的生产设备进行监控分析。

五是仓储物流智能化。基于工业互联网系统解决方案开展物流仓储仿真规划、物流调度执行优化、仓储管理、车辆管理四个业务环节应用实践，提升物流及仓储综合管理水平，简单重复性工序90%以上实现自动化。

六是能源管理集约化。基于工业互联网系统解决方案开展能源统计监测、能源在线调度、能源预测优化三个业务环节应用实践，开展主要耗能设备实时监测与控制，实现能源资源的优化调度、平衡预测和有效管理。

七是安环管理规范化。基于工业互联网系统解决方案开展安全管理和环保管理，缩短安全事件响应时间、降低安全事故停机率，实现工业废弃物100%集中管控、达标排放。

认定标准：以上评价内容中，需至少符合任意2项（第7项为必备项）。

2.网络化协同

评价内容：企业通过跨部门、跨层级、跨企业的数据互通和业务互联，推动供应链上的企业和合作伙伴共享客户、订单、设计、生产、经营等各类信息资源，实现网络化的协同设计、协同生产或协同服务，促进资源共享、能力交易以及业务优化配置，具体如下：

一是基于工业互联网系统解决方案整合社会研发资源，开展跨企业、跨区域、跨领域的网络化协同设计，提升产品协同研发效率。

二是基于工业互联网系统解决方案开展客户订单、生产计划、产品配送等信息共享与业务协作，实现网络化协同生产，提升产业链上下游资源对接效率。

三是基于工业互联网系统解决方案整合或利用上下游企业研产供销资源，开展供需精准传递、生产联动联调、产能柔性配置等，实现产业链协同一体化运作，打造产业协作共赢发展生态。

认定标准：以上评价内容中，需至少符合任意1项。

3.数字化管理

评价内容：企业通过打通核心数据链，贯通生产制造全场景、全过程，基于数据的广泛汇聚、集成优化和价值挖掘，优化、创新乃至重塑企业战略决策、产品研发、生产制造、经营管理、市场服务等业务活动，构建数据驱动的高效运营管理新模式，具体如下：

一是基于工业互联网标识解析体系实现企业数据的规范化管理，形成跨部门、跨业务、跨系统的多维度数据汇聚，通过对有效数据资源的价值挖掘与分析，驱动企业高质量发展。

二是基于工业互联网系统解决方案开展经营管理、生产管理、供应链管理、市场营销、用户服务等应用实践，建立适应企业数字化转型的业务流程体系，以数据为驱动提升企业科学管理、精准决策水平。

三是基于工业互联网系统解决方案集成企业研发设计、生产制造、运维服务、市场销售等数据资源，构建覆盖企业业务活动全过程的数字孪生体，在具体工业场景中开展数字孪生应用实践。

认定标准：以上评价内容中，需至少符合任意1项。

4.个性化定制

评价内容：企业面向消费者个性化需求，通过客户需求准确获取和分析、敏捷产品开发设计、柔性智能生产、精准交付服务等实现用户在产品全生命周期中的深度参与，以低成本、高质量和高效率的大批量生产实现产品个性化设计、生产、销售及服务，具体如下：

一是基于工业互联网系统解决方案获取客户需求并开展模块化、个性化设计，实现产品的敏捷研发。

二是基于工业互联网系统解决方案获取客户需求并开展柔性生产、敏捷配送，及时响应制造任务和产品品种变化。

三是基于工业互联网系统解决方案实现客户在设计、生产、配送、服务等环节的全流程参与，提升客户满意度。

认定标准：以上评价内容中，需至少符合任意1项。

5.服务化延伸

评价内容：企业从原有制造业务向价值链两端高附加值环节延伸，从以加工组装为主向“制造+服务”转型，从单纯出售产品向出售“产品+服务”转变，具体如下：

一是基于工业互联网系统解决方案开展智能产品（设备）远程监测、故障诊断、预测性维护等增值服务，提升产品（设备）效能效益。

二是基于工业互联网系统解决方案开展闲置制造能力在线交易、融资租赁、信用贷款等产融服务，提升产业链附加值。

三是基于工业互联网系统解决方案将机理模型、工艺流程、管理经验等工业知识固化封装为面向特定场景的工业APP或工业软件，通过在线交易的方式输出解决方案。

认定标准：以上评价内容中，需至少符合任意1项。

6.安全化生产

评价内容：企业通过工业互联网在安全生产中的融合应用，增强工业安全生产的感知、监测、预警、处置和评估能力，加速安全生产从静态分析向动态感知、事后应急向事前预防、单点防控向全局联防的转变，提升工业生产本质安全水平，具体如下：

一是整合现有安全生产数据、平台和系统，构建企业级或行业级工业互联网安全生产监管平台，具备安全感知、监测、预警、处置、评估等功能，实现安全生产全过程、全要素、全产业链的连接和监管。

二是依托工业互联网平台，开展安全管理经验知识的软件化沉淀和智能化应用，加快工艺优化、预测性维护、智能巡检、风险预警、故障自愈、网格化安全管理等工业APP和解决方案的应用推广，实现安全生产的可预测、可管控。

三是在工业互联网建设中，将数字孪生技术应用于安全生产管理，实现关键设备全生命周期、生产工艺全流程的数字化、可视化、透明化，提升企业安全生产数据管理能力。

认定标准：以上评价内容中，需至少符合任意1项。

（三）产生效益

1.经济效益和社会效益

评价内容：示范应用在企业提质、降本、增效、降耗等方面发挥了重要作用，认定当年，企业相关经济效益或社会效益指标较上一年度应有所改变，具体如下：

一是生产效率提升

二是产品不良率降低

三是生产成本降低

四是单位产值能耗降低

五是产品研发周期缩短

六是社会贡献率提升

认定标准：以上评价内容中，需至少符合任意2项。

说明：社会贡献率=社会贡献总额/平均资产总额×100%。社会贡献总额包括工资（含奖金、津贴等工资性收入）、劳保退休统筹及其他社会福利支出、利息支出净额、应交增值税、产品销售税金及附加、应交所得税及其他税、净利润。

2.两化融合发展水平

评价内容：示范应用在提升企业两化融合发展水平上发挥了重要作用，认定当年，企业两化融合相关指标应实现同比正增长。

认定标准：示范应用在提升企业两化融合发展水平上发挥了重要作用，认定当年，企业两化融合相关指标较上一年度应有所提升，具体如下：

一是企业两化融合发展水平提升2%以上。

二是企业数字研发设计工具普及率、关键工序数控化率、生产设备数字化率、数字化生产设备联网率等关键指标均有不同程度提升或者不低于行业平均水平。

三是企业两化融合发展阶段应处于集成提升或创新突破。

认定标准：以上评价内容中，需至少符合任意1项。

说明：企业应依托两化融合公共服务平台（https：//cspiii.com/）开展两化融合自评估、自诊断、自对标，形成企业两化融合发展水平评估报告，每年至少进行二次。企业两化融合分为四个发展阶段，从低到高依次为起步建设、单项覆盖、集成提升、创新突破。

（四）示范效应

评价内容：示范应用经过企业的不断改进与优化，具备在本行业乃至其他行业示范推广的可行性，具体如下：

一是示范应用已经形成行业典型案例，认定当年已在本行业开展示范推广活动。

二是示范应用已经形成行业解决方案并向本行业其他企业进行了输出，认定当年已经落地并开始实施。

三是示范应用已经形成系统解决方案并向其他行业企业进行了输出，认定当年已经落地并开始实施。

认定标准：以上评价内容中，需至少符合任意1项。

四、认定程序

依据《实施细则》第三章有关规定进行，原则上每年组织一次，企业自愿申报，具体时间和要求以黑龙江省工业和信息化厅通知为准。

五、认定结果

基于评价内容及认定标准确定符合条件的示范应用项目，每年不超过50个，按规定给予分档奖励。

附件2黑龙江省具有标杆示范作用工业互联网平台企业认定标准（试行）

为贯彻落实《推动“数字龙江”建设加快数字经济高质量发展若干政策措施》，支撑开展具有标杆示范作用工业互联网平台企业（以下简称平台企业）的认定，制定本标准。

一、认定范围

承担工业互联网平台（以下简称平台）建设及运营的企业，能够面向黑龙江省内工业企业数字化、网络化、智能化需求，构建基于平台的海量数据采集、汇聚、分析服务体系，支撑制造资源泛在连接、弹性供给、高效配置，助力工业企业实现提质、降本、增效、降耗，服务的行业包含但不限于装备工业、石化工业、能源工业、食品工业、医药工业、冶金工业、建材工业、轻工业、纺织工业等。

二、基本条件

平台企业应满足《<推动“数字龙江”建设加快数字经济高质量发展若干政策措施>实施细则（试行）》（以下简称《实施细则》）第七条所规定的基本条件。

三、评价内容及认定标准

平台企业主要从基础能力和服务能力两个维度进行认定，共包括七个方面的评价内容和认定标准。认定标准应符合任意一项是认定的基本（最低）标准，最终认定结论由认定标准中符合高标准项或同时符合多项标准得出。

（一）基础能力

1.运营能力

评价内容：平台企业应具备可持续的独立运营能力，研发投入合理，经济效益良好，拥有一批稳定的合作伙伴，初步建立多方参与、互利共赢的平台生态。

认定标准：平台企业应符合以下项目中的任意一项。

（1）平台建成运行时间不足1年，认定当年实际投资不低于400万元，与不少于3家云服务商或工业互联网系统解决方案供应商建立稳定的合作关系。

（2）平台建成运行时间满1年但不足2年，认定当年已累计投资不低于800万元，与不少于5家云服务商或工业互联网系统解决方案供应商建立稳定的合作关系。

（3）平台建成运行时间满2年但不足3年，认定当年已累计投资不低于1200万元，与不少于8家云服务商或工业互联网系统解决方案供应商建立稳定的合作关系。

（4）平台建成运行时间达到3年以上，认定当年已累计投资超过2000万元，与10家以上云服务商或工业互联网系统解决方案供应商建立稳定的合作关系。

2.技术支撑能力

评价内容：平台企业应具备相应的资质，拥有一定数量的发明专利（不包括实用新型专利）和软件著作权，技术团队人员数量和架构合理，具有较强技术研发能力，能够及时响应工业企业用户需求。

认定标准：平台企业应符合以下项目中的任意一项。

（1）平台企业已获得国家或我省工业互联网相关资质，拥有不少于5项的发明专利（不包括实用新型专利）和软件著作权，技术团队不少于10人。

（2）平台企业已获得国家或我省工业互联网相关资质，拥有不少于8项的发明专利（不包括实用新型专利）和软件著作权，技术团队不少于20人。

（3）平台企业已获得国家或我省工业互联网相关资质，拥有不少于15项的发明专利（不包括实用新型专利）和软件著作权，技术团队不少于30人。

（4）平台企业已获得国家或我省工业互联网相关资质，拥有不少于20项的发明专利（不包括实用新型专利）和软件著作权，技术团队超过40人。

（二）服务能力

1.数据存储服务能力

评价内容：平台具备稳定可靠的云基础设施运行环境，部署主流数据库系统，能够为工业企业用户提供可灵活调度的计算、存储和网络服务。

认定标准：平台企业应符合以下项目中的任意一项。

（1）平台数据存储环境符合相应的等级保护规定，已存储不少于120户工业企业用户的工业数据，可以基于公有云、私有云或混合云为工业企业用户提供服务。

（2）平台数据存储环境符合相应的等级保护规定，已存储不少于160户工业企业用户的工业数据，可以基于公有云、私有云或混合云为工业企业用户提供服务。

（3）平台数据存储环境符合相应的等级保护规定，已存储240户以上工业企业用户的工业数据，可以基于公有云、私有云或混合云为工业企业用户提供服务。

2.应用服务能力

评价内容：平台面向工业企业用户，可以基于云计算服务架构提供研发、采购、生产、营销、管理和服务等工业软件，或基于平台即服务架构提供面向不同工业场景的机理模型、微服务组件和工业APP，具备各类软件应用及工业APP的搜索、认证、交易、运行、维护等管理能力。

认定标准：平台企业应符合以下项目中的任意一项。

（1）平台已发布的云化工业软件和工业APP（含接入）数量不少于60个，已定阅的工业企业用户数不少于40户。

（2）平台已发布的云化工业软件和工业APP（含接入）数量不少于80个，已定阅的工业企业用户数不少于70户。

（3）平台已发布的云化工业软件和工业APP（含接入）数量不少于100个，已定阅的工业企业用户数超过100户。

3.示范推广能力

评价内容：平台能够面向不同行业工业场景，为工业企业用户提供智能化制造、网络化协同、个性化定制、服务化延伸、数字化管理、安全化生产等系统解决方案，形成一批可复制、易推广的应用案例。

认定标准：平台企业应符合以下项目中的任意一项。

（1）基于平台可提供的系统解决方案，已形成至少2个不同工业场景下的应用案例，具备向相关行业同类场景推广的可行性。

（2）基于平台可提供的系统解决方案，已形成至少4个不同工业场景下的应用案例，具备向相关行业同类场景推广的可行性。

（3）基于平台可提供的系统解决方案，已形成至少8个不同工业场景下的应用案例，具备向相关行业同类场景推广的可行性。

（4）基于平台可提供的系统解决方案，已形成至少10个以上不同工业场景下的应用案例，具备向相关行业同类场景推广的可行性。

4.用户覆盖能力

评价内容：平台服务范围覆盖多个行业，拥有一定数量的工业企业用户，用户活跃度保持相对稳定。

认定标准：平台企业应符合以下项目中的任意一项并计相应分值。

（1）平台服务已覆盖至少3个行业，用户总数不少于100户，月平均活跃用户（单月累计在线10小时以上或登录5次以上）数量不少于30户。

（2）平台服务已覆盖至少4个行业，用户总数不少于150户，月平均活跃用户（单月累计在线10小时以上或登录5次以上）数量不少于45户。

（3）平台服务已覆盖至少6个行业，用户总数不少于200户，月平均活跃用户（单月累计在线10小时以上或登录5次以上）数量不少于60户。

（4）平台服务已覆盖8个及以上行业，用户总数超过300户，月平均活跃用户（单月累计在线10小时以上或登录5次以上）数量不少于90户。

5.用户满意度

评价内容：平台企业应当定期对已服务的工业企业用户开展回访，按照不满意、一般、满意三个等次了解工业企业用户对于平台服务的满意度，收集整理工业企业用户业务需求和意见建议，作为改进和提升平台服务质量的重要依据。

认定标准：平台企业应符合以下项目中的任意一项。

（1）调阅平台企业的工业企业用户回访记录，认为不满意的用户数与平台总用户数之比应不高于25%。

（2）调阅平台企业的工业企业用户回访记录，认为不满意的用户数与平台总用户数之比应不高于20%。

（3）调阅平台企业的工业企业用户回访记录，认为不满意的用户数与平台总用户数之比应不高于15%。

（4）调阅平台企业的工业企业用户回访记录，认为不满意的用户数与平台总用户数之比应不高于10%。

四、认定程序

依据《实施细则》第三章有关规定进行，原则上每年组织一次，企业自愿申报，具体时间和要求以黑龙江省工业和信息化厅通知为准。

五、认定结果

基于评价内容及认定标准确定符合条件的平台企业，按规定给予分档奖励。

附件3黑龙江省中小企业数字化示范标杆企业认定标准（试行）

为发挥数字化示范标杆企业在中小企业数字化改造中的典型带动作用，依据黑龙江省人民政府《推动“数字龙江”建设加快数字经济高质量发展若干政策措施》（黑政规〔2021〕14号）中小企业数字化赋能有关规定，制定本认定标准。

一、认定范围

本认定标准所称黑龙江省中小企业数字化示范标杆企业，是指在推进产品设计、制造设备、生产管理数字化，增强生产管控协同、市场响应、产业协作等能力，提升产品服务、生产制造、企业管理智能化水平等方面具有典型示范作用的中小企业，能够引领带动更多行业和企业推广应用数字化新技术、新模式。

二、基本条件

黑龙江省中小企业数字化示范标杆企业需满足以下基本条件：

（一）以制造业为重点，连续经营3年以上，具有独立法人资格。

（二）符合《中小企业划型标准规定》的企业。

（三）在黑龙江省境内注册成立3年以上，近2年主营业务收入或净利润平均增长率不低于5%。

（四）企业发展前景好，具有较好的经济、社会效益和较强的社会影响力。

三、评价内容及认定标准

黑龙江省中小企业数字化示范标杆企业主要从数字化改造、网络化协同和智能化升级三个维度进行评定，共包括七个方面的评价内容和认定标准。同时针对省级“专精特新”中小企业或国家级专精特新“小巨人”企业设置加分项。

（一）数字化改造

1.产品设计数字化

评价内容：企业在产品研发设计环节具有较好的数字化基础，能够借助多种计算机辅助设计软件、计算机辅助仿真软件缩短研发周期、降低研发成本、优化产品工艺、提升产品性能，并实现产品从研发到生产的全生命周期管理，具体如下：

一是采用计算机辅助设计（CAD）等技术，实现产品数字化设计。

二是采用计算机辅助工程（CAE）、计算机辅助工艺规划（CAPP）、设计和工艺路线仿真、可靠性评价等先进技术，实现工艺数字化设计及仿真优化。

三是建立产品生命周期管理系统（PLM）和物料清单系统（BOM），实现产品多配置管理、研发项目管理，产品设计、工艺数据的集成管理。

认定标准：以上评价内容中，需符合其中一项。

2.制造设备数字化

评价内容：企业广泛采用数字化生产设备，推动设备上云，能够基于信息系统和设备数据实现设备管理，具体如下：

一是在劳动强度大、工作环境差、危险系数高的生产制造环节实现“机器换人”。

二是实现锅炉、制冷等高能耗设备、电机、空压机等通用动力设备、工程机械、数控机床等智能装备、风电、光伏等新能源设备和其他各类生产现场的各型关键生产设备及其辅助设备联网上云。

三是采用计算机辅助制造（CAM）技术，对制造过程和生产设备进行控制与监视。采用分布式数控（DNC）实现CAD/CAM和计算机辅助生产管理系统集成互通。

四是面向企业主要产品生产制造所涉及的关键生产设备和重要辅助设备开展数据采集。基于设备采集数据实现设备台账、点检、保养、维修、产能、能耗等和设备管理数字化；分析稼动率、生产计数、加工节拍、换线时间、物料消耗等生产管理信息，提升设备利用率和生产管理水平。

认定标准：以上评价内容中，需至少符合其中一项。

3.生产管理数字化

评价内容：企业能够基于信息系统和新一代信息技术实现生产资源的优化配置和生产过程的高效管理，具体如下：

一是建立企业资源计划系统（ERP），提高人、财、物、事等企业资源管理功能。

二是建立制造执行系统（MES），实现制造数据、计划排产、生产调度、质量、设备、能效等管理功能。

三是建立仓库管理系统（WMS）和运输管理系统（TMS），实现生产制造现场物流与物料的精准管控。

四是建立质量管理系统（QMS）并实现可视化管理，实现质量检验、关键工序SPC分析、过程质量数据采集、管理、追溯与分析等。

五是制造执行系统（MES）、企业资源计划（ERP）与数字化三维设计仿真软件、产品生命周期管理系统（PLM）、供应链管理（SCM）、客户关系管理（CRM）等系统之间的多元异构数据实现互换，实现企业各个环节的高度柔性与高度集成。

六是基于工业互联网、大数据、云计算、人工智能等技术，实现生产系统自运行、自诊断、自优化，通过算法和模型构建感知、分析、预测、决策能力，促进生产制造智能化。

七是具有工业互联网在安全生产中的融合应用，增强工业安全生产的感知、监测、预警、处置和评估能力。

认定标准：以上评价内容中，需至少符合其中一项。

（二）网络化协同

1.企业内部联网上云

评价内容：企业具有较好的网络基础，能够全面支撑企业办公管理、生产管理、信息管理要求，具体如下：

一是利用现场总线、工业以太网、无线网络、物联网等技术实现设备、系统间的互联与通信。

二是使用5G专网、5G切片、NB—IoT、工业IPv6、无源光网络（PON）等新一代网络技术提升工业企业生产设备和信息系统的数据互通能力，推进生产控制网络与信息管理网络的深度融合。

认定标准：以上评价内容中，需至少符合其中一项。

2.产业链和产业集群协同创新

评价内容：依托工业互联网在产业链数据联通和产业集群资源协同的优势开展协同创新应用，具体如下：

工业互联网、资源、数据、产业链、产业集群、新模式。

一是开展品牌数字展厅、在线交易系统、全员营销系统等网络营销基础设施建设，实现客户画像、营销内容数字化。

二是开展搜索电商、平台电商、社交电商、直播电商、跨境电商等多种网络营销新模式，扩大品牌影响。

三是采用供应链管理系统（SCM），实现物流、信息流、单证流、商流和资金流五流合一。

四是依托互联网开展委托采购、联合采购、即时采购等网络采购新模式。

五是在产业链或产业集群内开展网络化协作，通过平台整合分散的制造资源和制造能力，对接融入大企业、行业或区域产业供应链体系，形成数据驱动的大中小企业融通创新典型模式。

认定标准：以上评价内容中，需至少符合其中一项。

（三）智能化升级

1.产品和服务智能化

评价内容：企业能够基于新一代信息技术提升创新能力，提供智能化的产品和服务，具体如下：

一是推动人工智能、新型传感器、AR/VR等技术的深度应用，研发智能化装备、智能机器人、车载智能终端、金融智能终端等高端装备。

二是发展新一代智能可穿戴设备、智能家居产品等新型消费类产品，创新发展智能安防、智能交通、智慧医疗、智慧教育、智能社区等领域的产品和服务。

三是面向消费者个性化需求，通过客户需求准确获取和分析、敏捷产品开发设计形成个性化定制方案，通过柔性智能生产实现大批量定制化生产、精准交付用户。

四是动态采集产品使用和服务过程数据，提供在线监控、远程诊断、预测性维护等延伸服务，丰富完善服务产品和业务模式，探索平台化、集成化、场景化增值服务。

五是建立全生命周期、全流程的客户关系管理系统（CRM），建设敏捷响应的用户服务体系，实现从订单到交付全流程的按需、精准服务。

认定标准：以上评价内容中，需至少符合其中一项。

2.企业管理智能化

评价内容：企业能够基于系统集成融合和新一代信息技术实现企业综合管理的智能化，具体如下：

一是在企业计划及发展战略制定、人力物力资源调配、风险与舆情监控、企业管理效率最优化等方面，利用数据智能技术，推动管理过程智能化。

二是结合大数据和人工智能技术，基于OA、ERP、MES、PLM系统，构建智能化大脑，推动组织优化、管理优化，提高企业管理效率，实现精细管理和智能决策。

三是推广应用财务机器人、电子发票服务平台等平台化智能管理服务。

认定标准：以上评价内容中，需至少符合其中一项。

（四）加分项

评价内容：对企业所获荣誉或“两化”融合贯标情况进行附加分评定。

一是企业属省级“专精特新”中小企业或国家级专精特新“小巨人”企业。

二是企业完成两化融合管理体系贯标。

认定标准：以上评价内容中，需至少符合其中一项。

四、认定程序

依据《实施细则》第三章有关规定进行，原则上每年组织一次，企业自愿申报，具体时间和要求以黑龙江省工业和信息化厅通知为准。

五、认定结果

基于评价内容及认定标准从高到低依次排序，认定前50户为黑龙江省中小企业数字化示范标杆企业。

附件4黑龙江省重点数字产品建设项目认定标准（试行）

为贯彻落实《推动“数字龙江”建设加快数字经济高质量发展若干政策措施》要求，制定本标准。

一、认定范围

符合《黑龙江省重点数字产品指导目录》的电子信息制造业企业建设项目。

《黑龙江省重点数字产品指导目录》分为重点支持和积极培育两类，重点支持的产品共9个类别，这些产品在我省有产业基础和技术支撑、可有效推动我省产业融合发展；积极培育的产品共3个类别，是符合国家产业政策导向、市场需求旺盛、处于技术前沿。

1.重点支持目录

序号 产品类别 说明

1 半导体材料和装备 碳化硅、砷化镓、蓝宝石等晶体材料及生产装备制造

2 传感器 在航空航天、汽车、气象、工业控制、农业等领域应用的压力、温度、湿度、加速度、声、磁、化学与生物、光、电等传感器

3 光学产品 激光通信系统、多光谱检测设备

4 计算机及外设 基于国产CPU和操作系统的服务器、个人电脑、便携终端、显示器、打印机、存储器以及工控机、嵌入式计算机等产品制造

5 电池 动力电池、储能电池、电动工具、数码及其他

6 汽车电子 汽车仪表、汽车电驱动总成、驱动电机、功率电子控制器、汽车总线产品

7 工业数字控制系统 集散式控制系统（DCS）、可编程逻辑控制器（PLC）、监视控制与数据采集（SCADA）设备、工业网联设备、工业机器人、电工仪表等

8 消费电子 老年服务电子设备、健康电子设备、服务机器人

9 北斗导航 车载北斗导航设备、北斗导航便携终端、基于北斗导航的应急救援设备

2.积极培育目录

序号 产品类别 说明

1 硅光子芯片 硅光子芯片的设计、先进封装和测试

2 虚拟现实（VR）、增强现实（AR）、混合现实（MR） 用于工业、教育、旅游、农业、医疗、产教融合、数字化展示等方面的虚拟现实（VR）、增强现实（AR）产品

3 量子信息 量子计算、量子通信

二、基本条件

企业及项目应满足下列条件：

1.项目企业必须是在黑龙江省行政区域内注册的制造业企业，具有独立法人资格，企业不属于失信被执行人，且未被列为经营异常名录。

2.项目符合国家产业政策，项目备案（核准）、用地、环评等审批要件齐备。

3.项目企业财务管理和会计核算制度健全，管理规范、核算准确，无违法违规记录。

4.项目在申报年度内已全部建成投产或分期投产（分期建成投产项目相关要件须对应分期审批建设内容）。项目申报单位须提供项目建成投产的相关财务、资产等验收证明及申报材料的真实性承诺。

三、评价内容及认定标准

依据以下四项原则：

1.有产业基础和技术支撑的产品；

2.可与我省传统优势产业融合发展的产品；

3.符合国家产业政策导向，市场空间大、增长快的产品；

4.处于技术前沿、前瞻性布局的产品。

附件5黑龙江省首版次软件创新产品市场化应用认定标准（试行）

为贯彻落实《推动“数字龙江”建设加快数字经济高质量发展若干政策措施》，支撑开展首版次软件创新产品市场化应用的认定，制定本标准。

一、认定范围

在黑龙江省行政区域内首次购买应用首版次软件创新产品的企业（以下简称购买方）。本标准所称首版次软件创新产品是指关键技术有重大创新与应用，功能、性能先进或可打破行业封锁、垄断，产品质量可满足行业需求，拥有自主知识产权，在黑龙江省行政区域内首次发布且处于市场推广初期的软件产品，不包括研制企业开发仅限于自用的软件产品。

二、基本条件

购买方应符合《<“数字龙江”建设加快数字经济高质量发展若干政策措施>实施细则》（以下简称《实施细则》）第七条规定的条件。

三、评价内容及认定标准

首版次软件创新产品市场化应用从首版次软件产品和购买方两个维度进行认定，包括5个方面的评价内容和认定标准。

（一）首版次软件产品

1.研制企业

评价内容：研制企业在黑龙江省行政区域内依法登记注册，具有独立法人资格，主体、主营业务及纳税均在黑龙江省，资产及经营状况良好。属于规上的研制企业还应履行黑龙江省软件和信息技术服务业统计填报义务。

认定标准：审核研制企业的营业执照、组织机构代码证、税务登记证（或多证合一证）以及上一年度的纳税证明、财务审计报告等证明文件，应真实有效。属于规上的研制企业应在黑龙江省软件和信息服务业行业统计年报报表之内。

2.知识产权

评价内容：由研制企业主导或自主研制，拥有软件著作权，著作权取得方式需为原始取得，申请认定的时间距产品取得该软件著作权的时间原则上不超过2年。

认定标准：审核研制企业的软件著作权证明文件（如果属于共有，研制企业需为第一权利人，且需提供共有权利人同意申请的证明），应真实有效且文实相符。

3.功能性能

评价内容：在黑龙江省行政区域内首次发布，技术水平达到国内先进，通过专业机构检测，运行安全、性能可靠。

认定标准：审核专业机构出具的查新报告、成果鉴定报告、产品检测报告等，应真实有效且文实相符。

4.市场应用

评价内容：产品实现市场化销售且销售总额不低于100万元。

认定标准：审核研制企业的相关销售合同及发票、货款到账凭证等证明文件，应真实有效。销售总额以研制企业实际入账额为准，不包括产品运维服务费。

（二）购买方

评价内容：购买方应为研制企业认可的首次采购使用其首版次软件产品的用户，且首版次软件创新产品已在购买方完成部署应用。

认定标准：审核购买方与研制企业签订的购销合同以及购买方的支付凭证、研制单位的货款到账凭证等证明文件，相互验证内容一致、真实有效且文实相符。实地查看购买方首版次软件创新产品的应用场景和运行记录，与研制企业销售的首版次软件创新产品实际功能与性能参数相符。

四、认定程序

依据《实施细则》有关规定进行，原则上每年组织一次，以购买方（仅限一家）为主体并与研制企业共同申报，具体时间和要求以黑龙江省工业和信息化厅通知为准。

五、认定结果

基于评价内容及认定标准确定购买方资格，对于符合条件的按规定给予补贴。

附件6

黑龙江省（非煤）智慧矿山

认定标准（试行）

为贯彻落实《推动“数字龙江”建设加快数字经济高质量发展若干政策措施》，支撑开展黑龙江省（非煤）智慧矿山的认定工作，制定本标准。

一、认定范围

（非煤）矿山是指除煤矿以外的其他类型矿山，包括铁、有色金属（铜、金、银、钼、钨、锡等）、石墨等矿山企业。（非煤）智慧矿山至少应包含采矿、选矿、尾矿三个部分。

二、基本条件

（一）企业应在黑龙江省行政区域内依法登记注册，具有独立法人资格，主体、主营业务及纳税均在黑龙江省，资产及经营状况良好等。

（二）企业持有国家有关部门颁发的采矿许可。严格遵守《矿产资源法》等法律法规，合法经营，证照齐全，遵纪守法。

（三）申报项目主要建设条件特别是资金来源基本落实，且应为计划新开工或续建项目。

三、评价内容及认定标准

（非煤）智慧矿山主要从智慧运营、智慧生产两个维度进行认定，共包括八个方面的评价内容和认定标准。认定标准应符合任意一项是认定的基本（最低）标准，最终认定结论由认定标准中符合高标准项或同时符合多项标准得出。

（一）矿山基础条件

1.资源规模

评价内容：按照国家针对矿山规模的划分标准，根据不同种类矿山的储量及开采量进行综合评价。

认定标准：企业应符合大型矿山、中型矿山或小型矿山中的任意一项。

2.投资规模

评价内容：根据矿山企业注册资金、实缴资金、固定资产投资进行综合评价。固定资产投资是指除资源、土地、基建等资产以外的投资，包括设备、系统、生产车辆等。固定资产的认定以取得完税发票并计入固定资产账为凭据。

认定标准：智能化改造项目总投资3000万元以上，企业应至少符合以下项目中的任意一项。

（1）矿山企业注册资金人民币1亿以上且实缴资金人民币2000万以上；

（2）矿山企业在申报年过去5年内固定资产投入总额超过3000万。

3.经营状况

评价内容：矿山企业是否纳入到国家统计的规模以上企业名录，企业营业收入及纳税情况。

认定标准：企业应至少符合以下项目中的任意一项。

（1）矿山企业上一年度纳入到国家统计的规模以上企业名录；

（2）上三年平均营业收入超过1亿元；

（3）纳税500万以上。

4.创新能力

评价内容：矿山企业人才队伍建设及中高级人才占比，企业拥有自主知识产权。

认定标准：企业应至少符合以下项目中的任意一项。

（1）企业在册人员超过500人；

（2）技术人员（本科以上学历或中级以上职称）占比超过10%；

（3）拥有自主知识产权（专利、著作权等）。

（二）信息基础设施

1.信息化架构

评价内容：智慧矿山采用基于工业互联网平台的云、边、端架构，建立面向“矿石流”的全流程智能生产管控系统，将矿山大量基于传统IT架构的信息系统作为工业互联网平台的数据源，继续发挥系统剩余价值，同时逐步推进传统信息化业务云化部署，实现矿山全流程的少人无人化生产。

认定标准：矿山企业应符合以下项目中的任意一项。

（1）矿山信息系统完整清晰采用云、边、端架构且业务运行良好；

（2）矿山信息系统部分采用云、边、端架构相关技术且能够稳定运行。

2.基础网络

评价内容：矿山企业要统筹工业互联网内外网络建设，整体规划部署矿山控制网、生产网、办公网、安防网等网络。

认定标准：矿山信息网络应至少符合以下项目中的任意一项。

（1）信息管理网络采用以太网技术的网络，矿山信息管理网主干网络传输速率不低于1000Mbps，出口带宽不低于100Mbps。

（2）采用矿山物联网平台，提升网络的布局布点与覆盖范围，实现地面与井下的无障碍通讯，满足大批量人员与移动设备精确定位、设备实时控制、大批量实时工业数据的采集与传输等要求。

（3）企业工业互联网内网开展IPv6、5G、NB—IoT（窄带物联网）等新型技术的应用部署，采用无线Mesh网络、Ad—hoc网络等技术实现全部移动装备和作业人员的无缝信息交互。

（4）矿山监控及自动化网络主干网络采用工业以太网或兼容以太网协议的网络（有线或5G），采用工业级设备，支持多种网络拓扑结构和冗余方式，网络故障重构时间不大于50ms，主干网络传输速率不应低于1000Mbps。

（5）配备高系统容量、高传输速率、多容错机制、低延时的高性能网络设备，采用分布式工业控制网络，建设基于软件定义的敏捷网络，实现网络资源优化配置。采用安全性高、抗干扰能力强、传输带宽不少于两倍冗余配置的交换机、路由器、通讯基站、通讯线路等设备，以满足生产控制数据、各类感知设备数据、无线通讯数据、视频信号的准确、可靠、安全、高实时性的传输需要。

（6）矿山安全监控系统、视频监视系统、工控生产系统、应用信息管理系统等网络层面完全隔离，主干传输网络中各独立子系统单独划分VLAN进行通信；支线网络采用工业以太网、现场总线或无线网络等方式。

3.信息安全

评价内容：按照《网络安全法》《工业控制系统信息安全防护指南》《加强工业互联网安全工作的指导意见》等相关文件要求，涵盖物理环境安全、应用系统安全、网络安全、数据安全、应用安全、主机安全、网络通讯安全及备份与恢复等的信息安全体系。

认定标准：矿山企业应至少符合以下项目中的任意一项。

（1）企业信息安全制度完善，信息安全防护体系完整，信息安全建设符合国家相关标准；

（2）网络安全、数据安全、边界安全、物联网安全、软件应用安全等信息安全状况综合评价。

（三）矿山智慧生产

1.智慧采矿

评价内容：智慧矿山包含露天矿、地下矿的采掘过程中采用的智能感知系统、智能装备以及自动化控制系统。

认定标准：矿山企业智慧采矿应至少符合以下项目中的任意一项。

（1）智能感知系统。感知层必须具备以下条件：露天矿山需部署边坡在线监测系统；地下矿山需部署安全检测系统和井下人员定位系统。其他条件根据系统部署数量、传感器点数、系统功能性能等信息进行综合评价。

（2）智能装备与自动化控制。是否采用了智能装备和自动化控制系统，采用智能装备数量、占比和自动化控制系统系统部署数量、控制点数、系统功能性能等信息进行综合评价。

2.智慧选矿

评价内容：智慧矿山在选矿过程中采用智能装备以及自动化控制系统。

认定标准：智能装备和生产过程自动化。是否采用了智能装备和自动化控制系统，采用智能装备的数量、占比和自动化（设备自动化率不低于70%）控制系统系统部署数量、控制点数、系统功能性能等信息进行综合评价。

3.智慧尾矿管理

评价内容：智慧矿山的尾矿处理中采用的监控系统和传感网络。

认定标准：在尾矿库是否部署符合规范的监控系统和传感网络，监控系统功能、性能指标。监测指标是否涵盖库水位监测、干滩监测、浸润线检测、位移监测、视频监测、库区雨量监测等内容。

（四）矿山综合管理平台

1.可视化管理平台

评价内容：企业对矿产资源进行可视化平台管理，在统一时空四维坐标系统下应能管理矿山全部的图视内容、拓扑结构和属性信息。对矿体进行精确圈定和三维建模，对矿产资源及伴生矿体进行品位估值和预测，按照品质和品位级别建立精细矿体分级分块模型，利用科学的技术经济评价方法，为开采设计、采掘计划编制、生产调度管理提供决策支持。

认定标准：矿山企业可视化管理平台应至少符合以下项目中的任意一项。

（1）可视化综合管理平台物理环境建设应包括独立指挥中心、可视化大屏幕、机房等设施；

（2）可视化综合管理平台系统功能、性能综合评价。包含但不限于三维建模与可视化平台、地测地理信息、矿产资源储量评价、地质资源管理、矿山测量管理、数字化孪生矿山等模块。

2.生产管理系统

评价内容：企业对采矿环节采用智能化软件、选矿环节采用MES系统、资源管理环节采用ERP系统。

认定标准：矿山企业生产管理系统应至少符合以下项目中的任意一项。

（1）在采矿生产环节是否部署智能设计模块，能够完整实现采矿数字化设计管理；

（2）在选矿环节是否采用了生产过程决策与执行软件（选矿MES），是否具备生产任务管理、生产过程管理、生产计划和排产管理、设备管理、能耗管理、人员管理、物料管理、质量管理等生产管理模块；

（3）企业是否部署ERP系统，且功能完善、运行稳定；独立部署还是接入集团ERP平台；部署/接入ERP模块的数量以及应用客户端数量（5个模块或20个客户端以上）。

（五）工业互联网数据协同应用

1.工业互联网云平台

评价内容：企业应部署云平台及数据中心，深度挖掘数据分析处理并支持决策能力。实现地质资源数据、生产数据、井巷工程实测模型、采矿设计模型、生产计划模型联动，集中展示和联动处理各类子系统数据，统筹安排各类生产要素和资源分配，动态调节装备作业计划和调度决策。

认定标准：矿山企业工业互联网云平台应至少符合以下项目中的任意一项。

（1）企业部署云平台及数据中心；

（2）能够深度挖掘数据分析处理并支持决策。

2.工业APP应用

评价内容：企业采用生产类APP应用（远程运维、预测性维护、智能分选等），以及移动APP（手机、pad等）应用。

认定标准：矿山企业工业APP应用应至少符合以下项目中的任意一项。

（1）生产过程中有工业APP应用，APP应用数量大于5个及客户端使用数量多于10个；

（2）管理或生产系统采用移动端APP，APP应用数量大于2个及客户端使用数量多于20个。

（六）安全生产与应急管理

评价内容：根据矿山基本情况、时态GIS数据和矿山在线监测数据、隐患排查数据、相似矿井的灾害事故机理分析，利用大数据分析技术建立重大灾害模态化预警方法，对岩体变形、易燃气体突出、突水透水、冲击地压、冒顶、自燃等灾害进行超前预测预报，实现危险性区域快速圈定和实时预警。

认定标准：矿山企业安全生产与应急管理应用应至少符合以下项目中的任意一项。

（1）相关安全信息系统符合国家规定并按要求接入监管部门监管网络；

（2）安全生产监控系统（人员、设备、环境、危险品等）功能完善，安全生产预警及应急指挥系统具备相应功能。部署以下至少3个系统：重大灾害预警系统、重大设备故障系统、重大灾害防治决策支持系统、人员管理（定位）系统、装备碰撞预警系统、安全监测及避险系统、危险品管理系统、应急指挥系统。

（七）绿色矿山

评价内容：实时跟踪监控矿山生产作业过程中的废水废气排放情况及固体废物环境管理情况，实时采集三废产生量及废水废气中的污染物监测等数据，矿山生产过程中产生的废弃物（岩石、矿渣等）进行综合利用，产生新价值。对生产过程中使用的水、电、热等能源进行资源回收利用，降低损耗。

认定标准：矿山企业绿色矿山应用应至少符合以下项目中的任意一项。

（1）资源能源消耗实现智能监控，电、热等能源实现实时监测、控制、分析；

（2）矿山“三废”有效处理，排放达标，有效循环利用，并可实现智能管控。

（八）智慧矿山下一步规划与工作计划

评价内容：矿山企业应聚焦自身信息化需要，按照国家、地方相关标准与规范，统筹规划智慧矿山的近期和远期目标，以及制定实现目标的工作计划。

认定标准：矿山企业下一步规划应至少符合以下项目中的任意一项。

（1）智慧矿山下一步规划合理性；

（2）智慧矿山下一步工作计划可行性。

四、认定结果

基于评价内容及认定标准确定符合条件的（非煤）智慧矿山，按规定给予项目投资补助。

附件7黑龙江省数字经济核心产业企业融资担保贷款风险代偿补偿资金管理暂行办法

第一章 总则

第一条 为深入推动实施数字经济发展战略，鼓励和引导融资担保机构加大对我省初创期、成长期数字经济核心产业企业的融资支持，推动数字经济做大做强，根据《黑龙江省人民政府关于印发“数字龙江”发展规划（2019-2025年）的通知》（黑政规〔2019〕7号）》《黑龙江省人民政府关于印发推动“数字龙江”建设加快数字经济高质量发展若干政策措施的通知》（黑政规〔2021〕14号）规定，制定本办法。

第二条 本办法所称的数字经济核心产业企业，是指为产业数字化发展提供数字技术、产品、服务、基础设施和解决方案，以及完全依赖于数字技术、数据要素的各类经济活动的企业。即：对应国家统计局《数字经济及其核心产业统计分类（2021）》中第01-04大类“数字产业化”基本范围的数字产业制造业、数字产品服务业、数字技术应用业、数字要素驱动业等行业。主要包括计算机通信和其他电子设备制造业、电信广播电视和卫星传输服务、互联网和相关服务、软件和信息技术服务业等。

第三条 数字经济核心产业企业融资担保风险代偿补偿资金（以下简称：“风险补偿资金”）是由省级财政预算安排，主要用于支持融资担保机构为本省数字经济核心产业企业开展软件著作权、专利权、商标专用权等质押抵押担保贷款提供融资担保，对实际发生的担保代偿损失给予适当补偿的专项资金。风险补偿金作为政府支持数字经济核心产业企业发展的融资增信手段，不构成对贷款的担保。

第四条 融资担保代偿损失，是指融资担保机构向被担保企业在银行贷款提供融资担保，在贷款到期时，被担保企业未能按协议或合同约定向银行偿还贷款，融资担保机构依据担保协议或担保合同代其偿还后，发生的实际损失。

第五条 凡是符合本办法第八条规定，专业从事本省中小微企业贷款融资担保、申报期上一年实际发生数字经济核心产业企业融资担保代偿损失业务的融资担保机构，均可申请风险补偿资金。

第六条 省工信厅、省知识产权局负责建立全省数字经济核心产业企业名单，及时提供相关融资担保机构，作为开展融资担保业务、申请担保代偿损失补偿的重要依据。名单根据企业增减变化情况实行动态调整。

第七条 风险补偿资金的管理和使用坚持公平、公开、公正原则，确保资金规范、安全和有效使用，避免资金闲置和浪费。

第二章 申报条件

第八条 申报风险补偿资金的融资担保机构应同时具备下列条件：

（一）在本省依法设立，具有独立法人资格，获得省金融监管部门颁发的融资性担保机构经营许可证。

（二）合规经营融资担保业务1年（含1年）以上，无违规经营行为和不良信用记录。

（三）被担保企业中应有符合《数字经济及其核心产业统计分类（2021）》规定，且经营地、主营业务和纳税均在本省的数字经济核心产业企业。

（四）上年度担保代偿率低于5%。

（五）融资担保机构的财务管理、风控等制度健全，按《融资性担保公司管理暂行办法》（中国银监会等七部委令〔2010〕第3号）规定建立财务会计制度并提取各项准备金。

（六）接受各级金融监管部门、财政部门监管，定期报送担保业务统计表、财务报表及金融监管部门、财政部门要求的其他报表。

第三章 申报材料

第九条 融资担保机构申请风险补偿资金应完整提交如下材料：

（一）数字经济核心产业企业风险补偿资金项目申请表。

（二）工商营业执照副本、融资性担保机构经营许可证（复印件）。

（三）会计师事务所出具的上一年度专项审计报告（需包含实际代偿发生额、代偿率和代偿损失率等）。

（四）经合作银行盖章确认的项目申报期内数字经济核心产业企业贷款担保代偿损失业务明细表。

（五）贷款担保代偿损失业务涉及的担保协议、担保主合同及履行凭证（银行借款合同、借款借据、还贷凭证、担保业务收费发票或收据等）。

（六）反担保协议和风险控制措施相关材料。

（七）银行代偿确认书及担保机构代偿凭证复印件。

（八）担保代偿损失业务情况说明（含基本情况、代偿情况以及是否符合代偿补偿条件进行简要说明）。

（九）代偿债务追偿措施和结果证明资料。

（十）如经过诉讼的，需提供法院裁决书。

（十一）对所有申报材料真实性负责的声明（须加盖申请单位公章）。

（十二）其他需要提供的材料。

第四章 资金规模及补偿标准

第十条 在省级财政每年安排的数字经济专项资金中，统筹一定规模的资金，作为融资担保机构为数字经济核心产业企业提供贷款担保发生代偿损失进行风险补偿的资金。

代偿损失补偿额=代偿实际损失额×补偿比例

代偿实际损失额=担保代偿额-再担保机构、其他政府担保基金及其他担保公司等按照合同约定分担的代偿金额-通过司法诉讼程序和其他途径已经追回的金额-上年度已取得的各级财政各类融资担保专项补贴资金

在年度安排的预算资金总额度内，以专项审计为基础，对融资担保机构开展数字经济核心产业企业软件著作权、专利权、商标专利权、股权等质押融资担保业务的，按照不超过上一年度代偿实际损失额20%的比例标准进行补偿，单户担保机构最高不超过200万元；对融资担保机构开展数字经济核心产业企业软件著作权、专利权、商标专利权、股权、应收账款等质押以及实物资产抵押等组合型融资担保业务的，按照不超过上一年度代偿实际损失额10%的比例标准进行补偿，最高不超过200万元。

融资担保机构为个体工商户提供的贷款担保和以个人名义提供的贷款担保不列入补偿范围。

第十一条 融资担保机构应将风险补偿资金用于充实担保赔偿准备金、未到期责任准备金。

兑现流程、监督管理与绩效考核等相关要求按照《实施细则》第三章、第四章规定执行。

黑龙江省工业和信息化厅办公室 2022年1月28日印发

（19）河北省发展和改革委员会等五部门关于印发《关于破解瓶颈制约助推数字经济健康发展的若干政策》的通知

各市（含定州、辛集市）人民政府、雄安新区管委会、省相关部门：

《关于破解瓶颈制约助推数字经济健康发展的若干政策》已经省委、省政府同意，现印发你们，请结合责任分工，抓好贯彻落实。

河北省发展和改革委员会

河北省工业和信息化厅

河北省市场监督管理局

河北省通信管理局

河北省住房和城乡建设厅

2021年12月16日

附件 关于破解瓶颈制约助推数字经济健康发展的若干政策

为进一步优化我省数据中心布局和建设，破解数字基础设施建设运营中的瓶颈制约，加快产业数字化步伐，推动我省数字经济健康发展，制定如下政策。

一、加强规划布局内数据中心能源等要素保障

（一）支持数据中心集约化规模化发展。根据全国一体化大数据中心协同创新体系算力枢纽实施方案和省委省政府部署，优化数据中心布局，推进建设张家口数据中心集群，主要承接北京数据中心迁移和增量外溢。新建大型、超大型数据中心原则上布局在国家枢纽节点数据中心集群范围内。建设雄安新区数字经济创新发展试验区、石家庄正定数字经济产业园、张家口怀来大数据产业基地、北京大兴国际机场临空经济区（廊坊）等数字经济重大平台，支持发展高性能、边缘数据中心。各地要统筹好在建和拟建数据中心项目，设置合理过渡期，确保平稳有序发展。（责任单位：省发展改革委、省工业和信息化厅、省通信管理局）

（二）加强大数据传输通道和能力建设。积极争取国家支持，优化通信网络结构，加快建设张家口数据中心集群内大型和超大型数据中心间的直联网络，推动建立张家口数据中心集群与北京、天津、雄安新区、石家庄等城市主城区的直联通道，布局建设新型互联网交换中心，提高数据传输质量，实现不同运营商网络间高效互联互通。（责任单位：省通信管理局、省工业和信息化厅、省发展改革委，中国移动河北有限公司、中国联通河北分公司、中国电信河北分公司）

（三）优先保障能源消费。对枢纽节点内数据中心集群、数字经济重大平台的能源消费需求，省市有关部门加强统筹、优先保障；完善能耗双控机制，研究制定具体办法，推动数据中心新增可再生能源消费在一定时间内不纳入区域能源消费总量；积极争取国家政策支持。加强能源发展规划、电网建设规划与张家口数据中心集群及数字经济重大平台建设方案的衔接，优先考虑集群内国家布局的重大算力设施用电需求，推动能源基地、电力设施与数据中心协同布局、联动建设。（责任单位：省发展改革委、省工业和信息化厅、省通信管理局、省能源局，国网河北省电力公司、国网冀北电力公司）

（四）加强供地供水保障。张家口数据中心集群及数字经济重大平台内的建设项目，要优化布局，科学选址，做好与国土空间规划衔接；对国内数据中心优势企业承担的、超大型的、技术先进的数据中心项目，加大建设用地供应力度，优先保障建设用地需求。优化水资源调配，坚持节水优先，研究制定张家口数据中心集群内水资源供给方案和保障措施。（责任单位：省自然资源厅、省发展改革委、省通信管理局、省水利厅）

（五）支持数据中心绿色化、低碳化发展。鼓励数据中心企业通过绿色电力交易、认购可再生能源绿色电力证书等方式，提升可再生能源利用比例，优先支持可再生能源供电达到50%及以上的数据中心新建和扩建。加大数据中心节能改造力度，引入优秀的节能产品和方案，加快推进全闪存、液冷技术、间接蒸发冷却机组等绿色节能设备应用，鼓励数据中心配套建设余热回收供暖设施，有效推进数据中心低碳运行。支持风电、光伏企业与数据中心企业开展深度合作，探索推进数据中心参与“源网荷储”一体化建设新模式。到2025年，电能利用效率（PUE＝数据中心总耗电量/数据中心IT设备耗电量）1.3以上的大型和超大型存量数据中心依法依规全部腾退关停。（责任单位：省发展改革委、省工业和信息化厅、省通信管理局、省能源局）

（六）建立健全数据中心能源消费监管体系。探索建立区域数据中心在线监测平台，开展企业内部能源计量审查，实时监测PUE、水资源利用效率值（WUE＝数据中心总耗水量/数据中心IT设备耗电量）。建立数据中心运行定期与不定期评测机制，加强数据中心单机架功率、经济贡献等指标考核，提升存量数据中心对区域经济的贡献度。加强新建审批、改造优化、能耗监察等各个环节的专业评估，支持有资质的第三方专业机构开展数据中心绿色等级评价、服务能力评价、运维人员培训、PUE测试等服务。（责任单位：省市场监管局、省发展改革委、省通信管理局、省能源局、省工业和信息化厅）

二、降低5G基站、数据中心等数字基础设施运营成本

（七）推进转供电改为直供电。支持具备条件的5G基站转供电改直供电，推动符合公变低压直供条件的5G基站完成直供电改造；不满足公变低压直供条件的，多方统筹推进，具备条件后及时改造。支持符合国标A级或国际T4建设标准的城市数据中心、边缘计算中心、超算中心实施转供电改直供电。电网企业、通信运营商及相关单位密切配合，制定完善全省 5G 基站、数据中心等转供电改造为直供电的具体办法和工作流程，全面清理规范转供电环节加价行为，涉及转供电的各级机关事业单位、学校、医院、国有企业等单位积极支持，共同做好“转改直”工作。（责任单位：省通信管理局、省发展改革委，国网河北省电力公司、国网冀北电力公司、中国移动河北有限公司、中国联通河北分公司、中国电信河北分公司、中国铁塔河北分公司）

（八）提高用电价格灵活性。枢纽节点数据中心集群内数据中心企业、集群外标准机架3000以上且PUE不高于1.35的数据中心企业，可自愿选择执行两部制或单一制电价。数据中心企业名单由相关市政府提供，由发展改革部门会同工信部门协调电网企业落实。具备条件的5G基站按照政策要求执行峰谷电价。（责任单位：省发展改革委、省工业和信息化厅、省通信管理局，国网河北省电力公司、国网冀北电力公司）

（九）鼓励企业参与电力市场化交易。支持数据中心企业通过直接交易方式向绿色电力企业购买绿色电力产品。具备条件的5G基站，不受电压等级和用电量限制，纳入电力市场化交易范围，用电量打包参与市场化交易。基础电信运营企业、数据中心企业加强自身电力消费精细化管理，优化设备用电负荷，研究应用储能设备，降低用电成本。（责任单位：省发展改革委、省能源局，国网河北省电力公司、国网冀北电力公司、中国移动河北有限公司、中国联通河北分公司、中国电信河北分公司、中国铁塔河北分公司）

（十）推进社会公共资源开放。建立管廊、杆塔、楼顶等公共资源合作共享机制，探索多样化合作模式。入廊电信运营商向地下综合管廊建设运营单位缴纳费用，由两者根据市场化原则共同协商确定，降低通讯管道成本。推动通讯杆塔与电力、市政、交通等部门杆塔资源实现双向开放和一杆多用。（责任单位：省住房城乡建设厅、省通信管理局、省交通运输厅，国网河北省电力公司、国网冀北电力公司）

三、强化产业数字化转型的服务保障

（十一）提升数字化转型公共服务能力。鼓励各类平台、第三方机构面向广大中小微企业提供数字化转型所需的开发工具及公共性服务平台，开展低成本、低门槛、快部署服务，促进中小微企业数字化转型。鼓励与基础电信运营商深度合作，开展基于“5G＋”的创新研究与应用开发，在工业、医疗、应急、交通等重点领域实施一批应用示范项目。支持数字化转型服务咨询机构和区域数字化服务载体建设，丰富各类园区、特色小镇的数字化服务功能。（责任单位：省工业和信息化厅、省通信管理局、省发展改革委）

（十二）支持数字化转型工程研究中心建设。在钢铁、化工、汽车及县域特色产业集群等领域，依托龙头企业联合国内外智能制造标杆企业、知名科研院校建设一批数字化转型工程研究中心，面向产业链上下游企业和行业内中小微企业提供需求撮合、转型咨询、系统设计、工程建设等服务，推动大数据、人工智能等新一代信息技术对传统产业进行全方位、全链条改造。（责任单位：省发展改革委、省工业和信息化厅）

（十三）支持构建自主开源生态。支持建设开源社区，引导社会资本建立开源基金会，提高开源基金会在知识产权托管、项目孵化、社区运营等方面的能力。支持引进开源软件人才，培育一批有潜力的开源项目，鼓励建设安全可靠的开源托管平台，吸引和鼓励更多开发者参与社区建设。（责任单位：省科技厅、省工业和信息化厅、省发展改革委、省委网信办）

（十四）加强数字化发展网络安全保障。重点支持5G、云计算、工业互联网、物联网、车联网等新兴领域网络安全产品和服务研发，推动先进适用产品和服务在政务、通信、工业、金融、交通、广播电视、网络视听等领域部署和应用。支持建设安全防护、安全培训、安全运营、安全信息等领域的服务平台建设，建设一批网络安全产业园区。（责任单位：省科技厅、省委网信办、省通信管理局、省公安厅、省工业和信息化厅）

四、培育发展数据要素市场

（十五）建立健全数据要素市场规则。推进政务数据开放共享，促进社会数据资源价值提升。探索建立数据交易机制，激发数据资源活力。建立健全数据分类分级安全保护制度，完善社会化数据审查机制，加强政务数据、企业商业秘密和个人数据的保护。研究制定数据应用违规惩戒机制，依法依规加强对数据滥用、侵犯个人隐私等行为的管理和惩戒力度。（责任单位：省委网信办、省政务服务办、省工业和信息化厅、省发展改革委、省市场监管局、省地方金融监督管理局）

五、加强监管机制与治理体系建设

（十六）完善数字经济监管体系。健全市场准入和公平竞争监管制度，加强公平竞争审查工作，创新监管手段和方式，构建全方位、多层次、立体化监管体系，实现事前事中事后全链条全领域监管。搭建数字经济监测平台，研究建立准确反映我省数字经济发展水平和趋势的指标体系，持续完善以健康发展为导向的考核机制。（责任单位：省市场监管局、省发展改革委、省工业和信息化厅、省委网信办、省统计局）

（十七）构建多方参与的治理机制。坚持创新发展、包容审慎的治理原则，建立政府、行业组织、互联网平台企业和公众等多元主体参与、有效协同的治理新模式新机制。推动各监管部门建立协同联动机制和风险联合预警处置机制。科学界定互联网平台企业主体责任和义务，建立行业自律公约。提高全民数字素养和技能，引导社会积极参与数字治理，完善社会监督举报机制。加强网络空间治理，落实国家网络安全关键信息基础设施和等级保护制度，建立多层级、全天候、全方位的网络安全保障系统。（责任单位：省委网信办、省市场监管局）

（20）黑龙江省人民政府关于印发推动“数字龙江”建设加快数字经济高质量发展若干政策措施的通知 （黑政规〔2021〕14号）

各市（地）人民政府（行署），省政府各有关直属单位：

现将《推动“数字龙江”建设加快数字经济高质量发展若干政策措施》印发给你们，请认真组织实施。

黑龙江省人民政府

2021年10月20日

推动“数字龙江”建设加快数字经济高质量发展若干政策措施

为深入贯彻党和国家发展数字经济战略部署以及《中共黑龙江省委黑龙江省人民政府印发〈关于“数字龙江”建设的指导意见〉的通知》（黑发〔2018〕29号）和《黑龙江省人民政府关于印发“数字龙江”发展规划（2019-2025年）的通知》（黑政规〔2019〕7号），落实2021年政府工作报告“加快发展数字经济”部署，推动制造业数字化转型和中小企业数字化赋能，培育我省经济发展新动能，力争到2025年我省数字经济核心产业增加值占GDP比重达到10%，关键业务环节全面数字化的大型制造企业比例达到60%左右、规模以上企业比例达到40%左右，数字经济发展达到全国平均水平，制定如下政策措施。

一、加快推动产业数字化

大力推进5G、工业互联网建设及应用，加快制造业数字化转型和中小企业数字化赋能，开展智慧农业、智能制造、智能交通、智慧物流、数字社会、数字政府等试点示范，以数字技术促进产业数字化智能化转型和创新发展。

（一）制造业数字化转型。

1.培育工业互联网应用新模式。鼓励企业利用5G实施内网改造，支持装备、石化、食品、原材料、医药、电子信息、民爆等重点行业企业开展智能化制造、网络化协同、个性化定制、服务化延伸、数字化管理、安全化生产等新模式新业态示范应用。经认定省级财政给予分档奖励，最高不超过200万元。（责任单位：省工信厅、财政厅）

2.支持企业争创产业数字化国家级试点示范。对获得工信部工业互联网领域专项资金支持项目的牵头单位，按照中央支持额度的20%省级财政给予补助，最高不超过100万元；对获得工信部工业互联网、大数据、5G、信息消费等试点示范牵头单位，奖励50万元。（责任单位：省工信厅、财政厅）

（二）中小企业数字化赋能。

3.实施中小企业上云用云工程。培育服务中小企业云平台，积极引导我省平台企业提升服务中小企业规模和质量，对于经认定具有标杆示范作用的平台企业，按照服务企业数量，省级财政给予分档奖励，最高不超过500万元。（责任单位：省工信厅、财政厅）

4.开展中小企业数字化改造。鼓励和引导中小企业推进产品设计、制造设备、生产管理数字化，增强生产管控协同、市场响应、产业协作等能力，提升产品服务、生产制造、企业管理智能化水平。每年认定中小企业数字化示范标杆企业50户，省级财政对每户企业一次性奖励50万元，其中省级专精特新中小企业一次性奖励100万元。（责任单位：省工信厅、财政厅）

（三）其他重点行业和领域数字化发展。

5.推动智慧农业建设。提升省级农业大数据平台功能，完善农业大数据共享开放体系。开展数字农业示范试点，推广物联网、大数据、人工智能等技术在测土配方施肥、苗情监测、病虫害防治、质量追溯、农机作业、土地托管等领域应用，推动数字农业高质量发展。支持国家级和省级数字乡村试点地区建设，根据国家政策要求给予配套支持。（责任单位：省农业农村厅、省供销合作社、省委网信办、省财政厅）

6.推动智能交通和智慧物流建设。开展高速公路和普通国省道准全天候管理系统、车路协同系统、智慧养护管理系统、智能建管平台和智能公交建设试点，加强治理超载超限管理系统建设。对纳入国家和省交通运输领域新型基础设施建设项目，按照国家规定给予资金支持。鼓励商贸物流企业在仓储、分拣、包装、配送等环节采用现代化物流装备设施，提高作业标准化、自动化、智能化水平。（责任单位：省交通运输厅、商务厅、财政厅）

7.推动智慧矿山建设。建立健全采矿生产运行监测、管理和调度信息服务网络，采用自动化、数字化、智能化装备，着力推动“机器换人”，提升本质安全。对经过验收、达到智慧矿山标准的煤矿、非煤矿山示范项目，省级财政按照项目总投资的一定比例给予一次性补助。（责任单位：省煤管局、发改委、工信厅、应急管理厅、财政厅）

8.加快数字政府和数字社会建设。全面推进“一网通办”，提升政府服务数字经济发展效能。支持加快建设省级社会治理大数据平台，制定统一数据标准和业务规范，鼓励市（地）按标准规范建设、改造升级市域社会治理大数据平台，实现社会治理数据跨部门、跨区域、跨层级互联互通、信息共享，在精准预测、智慧决策等深度应用中发挥更大作用。加快完善文化和旅游信息化、数字化、智慧化，推动龙江文化旅游产业资源创新发展。〔责任单位：省营商局、省委政法委、省发改委、公安厅、文化和旅游厅、财政厅、工信厅、各市（地）人民政府（行署）〕

二、加快推进数字产业发展

发展培育电子信息制造、软件、互联网、人工智能、大数据、云计算、区块链等数字产业，聚焦引进行业领军企业、壮大本地龙头骨干企业，激发数字产业发展新动能。

（一）引进培育数字产业企业。

9.培育壮大数字产业企业。鼓励市（地）、县（市、区）出台数字产业招商政策，重点引进电子信息、软件、互联网等国内外数字产业百强企业。支持龙头企业通过兼并重组、股权投资等方式开展产业链上下游垂直整合和跨领域产业链横向拓展。对履行统计填报义务、营业收入首次达到10亿元的数字产业企业，省级财政一次性奖励企业核心团队500万元。〔责任单位：省商务厅、工信厅、财政厅、统计局、各市（地）人民政府（行署）〕

10.支持市（地）依托省级以上开发区建设数字经济特色园区（基地）。省级在债券资金方面给予园区基础设施建设优先支持，落实省级分享增量税收返还政策，支持市县政府建设数字经济特色园区（基地）。（责任单位：省商务厅、财政厅）

（二）支持工业互联网平台建设及应用。

11.推动建设全省工业互联网标识解析服务体系。支持企事业单位建设国家工业互联网标识解析二级节点，对通过评估，接入20家以上企业的工业互联网标识解析二级节点，省级财政给予建设单位一次性补助500万元。培育壮大工业互联网平台建设和运营企业，对落地龙江的国家级工业互联网平台企业，按照“一事一议”方式给予政策支持。（责任单位：省工信厅、财政厅）

（三）加快发展壮大数字产品制造业。

12.实施黑龙江省数字产品制造业倍增行动。制定《黑龙江省重点数字产品指导目录》（以下简称《指导目录》），对符合《指导目录》且项目投资额（设备和软件，下同）2000万元以上的建设项目，建成投产后，省级财政按项目实际完成投资额的10%给予补助，单个项目最高不超过600万元。对成功引入符合《指导目录》发展方向总投资在5000万元以上的项目，鼓励市县出台奖励招商引资人员政策。（责任单位：省工信厅、商务厅、财政厅）

（四）大力发展软件和信息技术服务业。

13.支持软件和信息技术服务业企业做优做大。对新纳入规上统计且履行统计填报义务的软件和信息技术服务业、互联网和相关服务业企业，省级财政一次性奖励50万元。鼓励企业开展软件能力成熟度（CMMI）建设，对通过评估且获得3级及以上等级评定的企业，省级财政给予一次性分档奖励，最高不超过150万元。（责任单位：省工信厅、统计局、财政厅）

14.支持首版次软件创新产品应用推广。支持软件企业面向我省制造业数字化转型需求，研发嵌入式软件、应用软件系统和解决方案。对我省首次购买应用省内首版次软件创新产品的企业，省级财政按产品实际销售价格的20%给予补贴，单个产品补贴最高不超过50万元。（责任单位：省工信厅、财政厅）

三、加强要素保障

15.加快新型信息基础设施建设。支持基础电信企业加快5G、千兆光纤网络、哈尔滨国家级互联网骨干直联点建设，推动市、县和重点园区5G全面覆盖，建设规模达到全国平均水平。统筹推进IPv6规模部署和应用。推动哈尔滨成为全国一体化算力网络国家枢纽节点，有序推进超级计算、分布式计算和云计算中心建设，打造全国大数据中心重要基地。建立大数据电力专项交易机制，省、市财政合力，推动对符合有关规定且同时享受大数据电力专项交易价格和大工业电价政策的大数据中心企业降低用电成本。（责任单位：省通信管理局、省委网信办、省发改委、财政厅、工信厅、哈尔滨市人民政府）

16.加强科技创新。支持数字经济核心产业企业、高校和科研院所参与国家重点实验室、国家产业创新中心、国家制造业创新中心、国家技术创新中心、国家工程研究中心、国家企业技术中心等创新平台建设，加大研发投入力度，提升技术创新能力，促进科技成果转化，加快数字产业化步伐。（责任单位：省科技厅、发改委、工信厅、财政厅）

17.加大人才培引。支持省内重点高校加强数字经济新兴学科建设，优化专业结构和师资配备，加强信息技术与传统工业、农业等学科融合的复合型人才培养。深化产教融合、校企合作，建设一批数字经济产教融合联盟和人才培育基地。鼓励市（地）针对数字经济核心产业领域高端人才和骨干人才工资性收入增长、科技成果参与分配，以及住房、医疗保障和子女入托入学等方面制订创新性优惠政策，提升数字经济人才吸引力。〔责任单位：省教育厅、人社厅、省委网信办、各市（地）人民政府（行署）〕

18.加大金融支持。建立数字经济核心产业企业贷款担保风险补偿机制，鼓励担保机构开展软件著作权、专利权、商标专用权等质押担保贷款，引导银行业金融机构加大对初创期和成长期数字经济核心产业企业的贷款支持力度。对担保机构发生的贷款担保代偿损失，按照一定额度和比例给予风险补偿。（责任单位：省工信厅、财政厅、知识产权局）

19.降低数字经济企业办公场地租赁成本。鼓励市（地）政府（行署）整合各部门和事业单位闲置房产，在不改变房产所有权和性质的前提下，探索建立闲置资产盘活利用机制，为数字经济核心产业企业提供廉租办公场地，降低企业运营成本。〔责任单位：各市（地）人民政府（行署）〕

20.支持培育数据要素市场。推进政府数据开放共享，促进社会数据资源价值提升。建立规范的公共数据管理制度，提高数据质量，丰富公共数据产品，支撑数字经济新业态新模式。探索建立数据交易机制，激发数据资源活力。建立健全数据分类分级安全保护制度，加强政务数据、企业商业秘密和个人数据的保护。（责任单位：省委网信办、省营商环境局、发改委、工信厅）

发展数字经济是龙江产业结构转型和高质量发展的关键一招，也是一项开创性、系统性工程，省市两级要在省“数字龙江”建设领导小组统一领导下，建立跨部门工作专班和工作机制，统筹各方资源力量，协调落实政策措施，协商解决重大问题，保障“十四五”期间龙江数字经济实现跨越式发展。各市（地）要按照财政补贴分级原则研究出台相关配套支持政策。

符合本政策措施的项目，同时符合我省其他扶持政策规定的，在同一年度内按从高不重复的原则予以支持，另有规定的除外。本政策措施自印发之日起施行，有效期4年。

（21）山西省人民政府关于印发山西省加快推进数字经济发展的实施意见和若干政策的通知（晋政发〔2021〕25号）

各市、县人民政府，省人民政府各委、办、厅、局：

　　现将《山西省加快推进数字经济发展的实施意见》和《山西省加快推进数字经济发展的若干政策》印发给你们，请认真贯彻执行。2019年8月27日省政府印发的《山西省加快推进数字经济发展实施意见和若干政策》（晋政发〔2019〕20号）同时废止。

**山西省加快推进数字经济发展的实施意见**

为贯彻落实国家数字经济发展战略,加快我省经济社会数字化转型进程,形成具有较强竞争力的数字经济产业体系,现提出以下意见。

一、指导思想

以习近平新时代中国特色社会主义思想为指导,贯彻落实“网络强国”战略部署,紧抓新一代信息技术创新发展契机,围绕“网、智、数、器、芯”五大领域,统筹布局全省数字经济发展体系,大力培育数字化产业,着力推进新技术、新模式、新业态与实体经济深度融合,加快“数字山西”建设,以数字化推动智能化,以智能化培育新动能,以新动能促进新发展。

二、基本原则

包容创新,率先发展。坚持创新引领,鼓励优先发展。推动数字化技术产品、应用模式、商业模式和体制机制的协同创新,提升发展的平衡性、包容性和可持续性。

共建共享,协同共治。扩大共享开放,强化民生服务。鼓励多元主体参与数字经济治理,以数据融通应用,实现协同治理和精准管理。

应用驱动,开放合作。坚持需求导向,以应用为牵引,推动产业集聚发展。激发、调动各类资源要素潜能,加大国内外交流合作力度,建立创新合作、互利共赢的发展模式。

统筹推进,安全发展。建立并持续完善适用于数字经济发展的政策法规和制度体系,加强安全防控、风险监管和权益保障,形成与数字经济发展良性互动的发展体系。

三、发展目标

到2022年,全省数字经济创新发展基础进一步筑实。网络基础设施建设不断完善,公共领域数据资源共享开放机制建立健全,省级智能工厂、数字化车间实现“双百”目标,集聚1—2 个在特色领域具有国际影响力和一批具有区域竞争力的数字经济领域企业,信息产业保持高速增长,数字经济规模突破5000亿元。

到2025年,全省数字经济迈入快速扩展期。先进泛在的数字基础设施基本建成,数字经济与社会各行业领域深度融合,培育2-3个具有国际影响力、若干具备国内牵引性、一批区域竞争力强的数字经济领域企业,打造一批产业集聚度高、规模效益显著的数字产业基地。与数字经济相适应的政策法规和制度体系建立完善,多元协同共治体系基本形成,全民数字素养明显提升,数字经济规模达到8000亿元。

四、重点任务

(一)“网”:提升网络设施能力

夯实基础网络服务能力。认真贯彻执行《山西省通信设施建设与保护条例》,支持基础电信企业持续加大投入,普遍提供固定百兆宽带接入能力,加快固定宽带千兆网络建设,努力实现家庭、企业、园区、写字楼光纤宽带网络覆盖。将通信基础设施纳入城市

规划体系,推动农村光纤和4G 网络的覆盖广度、深度。推进市政公共基础设施全面开放共享,推动通信网络设施IPv6 改造升级。加快建设运营山西综改示范区国际互联网数据专用通道,提升园

区企业对外开放合作能力。加快建设国家级互联网骨干直联点,全面增强网间通信服务能力。(责任单位:省通信管理局、省工信厅,各市人民政府,山西综改示范区管委会)

加快建设新型基础设施。持续完善配套政策体系,降低电力引入和扩容成本,加快推动5G 站址规划和基站建设,实现全省重点区域连续覆盖。加快推动5G 创新应用,组建5G 产业联盟,鼓励设立联合创新中心,协同开展5G 技术研究与行业应用。积极推进物联网、车联网等设施部署,支持阳泉智能物联网应用基地建设,探索资源型中小城市智能化转型路径。(责任单位:省通信管理局、省工信厅,国网山西省电力公司,各市人民政府)

推进工业互联网建设。面向能源、制造业等重点行业,培育一批国内领先的工业互联网平台服务商和研究创新机构,建设一批行业知名度高、影响力强的工业互联网平台,建设和运营工业互联网标识解析二级节点,实现与国家工业互联网系统对接,构建工业互联网产业生态。(责任单位:省工信厅、省通信管理局,各市人民政府)

支持建设试验基础设施。建设完善支撑新型通信设备验证的区域性实验场地,开展规模化试验和集成化应用。支持智能网联重载公路示范基地项目,探索重载货运的智能网联转型路径。支持开展面向车联网、无人机、无人驾驶、无人配送等新技术新装备的专用试验场地建设,完善制度标准和体制机制,推动相关技术产品的试验验证和成果应用。(责任单位:省通信管理局、省发展改革委、省工信厅、省公安厅、省交通厅,各市人民政府)

完善数字经济安全体系建设。按照“谁主管、谁负责”和属地管理的原则,严格落实网络安全工作责任,加强数据安全管理,健全风险预警、情报共享和应急协调机制。建立健全山西省网络安全综合防控体系,形成多部门联合作战工作机制。以大数据、人工智能、机器学习、网络空间地图等新技术新应用为依托建设完善山西省关键信息基础设施安全保卫平台。构建山西省网络安全智慧大脑,绘制山西省网络空间地理信息图谱,实现挂图作战。打造“三化六防”的网络安全防护机制,大力提升我省的网络安全防护能力。(责任单位:省委网信办、省公安厅、省通信管理局、省工信厅、省财政厅、省审批服务管理局,各市人民政府)

专栏1 建设新型智能基础设施

加快5G 网络部署应用。依托山西移动、山西电信、山西联通、山西铁塔等单位开展5G 网络建设,到2022 年,推动建成5G 基站3 万个,实现政务、教育、金融、医疗、工业互联网等垂直行业应用区域、交通枢纽、高新产业园区等重点区域的5G 网络覆盖。鼓励设立5G 联合创新中心,在交通物流、能源制造、公共安全、应急安全等领域开展行业融合和应用创新。

推进工业互联网建设应用。争当能源革命排头兵,提高能源供给体系质量效益,推进能源科技创新,推动建立山西省工业互联网联盟。构建工业互联网标识解析体系,加快建设工业互联网标识解析二级节点,提供工业标识注册、解析、查询、备案、认证等服务。

(二)“智”:推进经济社会智能化转型

推动工业云服务平台建设。依托工业云服务平台,加强工业经济运行监测调度,做好重点企业和重点项目的跟踪服务。积极对接国家工业大数据平台,对工业数据开发利用、分级分类等进行规范管理。研究建立数据管理推进机制,推动数据管理能力成熟度评估模型(DCMM)在企业应用落地,提高企业数据管理能力。(责任单位:省工信厅)

打造“两化”融合升级版。持续开展“两化”融合管理体系贯标。围绕煤炭、焦化、钢铁等传统优势行业,打造无人车间、智慧矿井等“智能+”示范工程。加强总体规划与顶层设计,推动企业自主有序地将基础设施、平台系统、业务应用等逐步上云。推进工业技术软件化,支持面向重点行业研发工业软件、工业 App。鼓励省内软件服务企业向云服务商转型,引导云服务企业加强核心技术和产品研发,主动适应市场需求搭建个性化云平台,重点培育若干有竞争力的云计算平台,不断提升云服务能力。(责任单位:省工信厅、省国资委、省能源局、省应急厅)

全面推动智能制造。把发展智能制造作为主攻方向,推动实现工业企业数字化、网络化、智能化,实施智能制造试点示范创建、智能制造专项支持、智能制造关键核心技术攻关等工程。强化智能制造支撑体系建设,推进形成钢铁冶金、轨道交通、煤机装备、汽车制造等智能化产业集群,着力营造良好发展环境,为建设“智造强省”奠定扎实基础。(责任单位:省工信厅、省科技厅)

打造新型智慧城市。推进城市网络化建设,构建覆盖城乡的智能化治理体系,强化数字技术在城市规划、建设、治理、服务等领域的应用。加强规划引导,支持城市公用领域的物联网应用和智能化改造,加快推进公共安全视频监控建设联网应用,构建覆盖城乡的智能感知体系。加快建设省、市两级城市综合管理服务平台,尽快实现县级数字化城市管理平台全面覆盖。推进平台互联互通、数据同步、业务协同,促进城市运行“一网统管”。(责任单位:省发展改革委、省住建厅,各市人民政府)

推行智慧政务。加强政务信息化顶层设计,创新部门信息系统建设运营模式,建设完善省级政务云平台,推动部门数据资源向省级政务云平台集聚,全面建成山西省大数据中心。推进政务数据资源整合共享,建立数据共享责任清单机制,推动以数据为支撑的政府管理和社会治理模式创新。围绕自然人、法人全生命周期,优化政务服务办理流程,大力推动信息惠民和“互联网+ 政务服务”,提升政务数据利用效果,加快政府服务数字化转型。(责任单位:省审批服务管理局、省发展改革委)

发展数字商务。推进内贸流通数字化建设,实施供应链创新及应用试点。促进对外贸易数字化发展,培育跨境电子商务新业态新模式。激发数字商务新主体活力,培育线上线下、跨界融合新主体及商务代运营等数字服务新主体。(责任单位:省商务厅、省发展改革委、省工信厅、太原海关)

建设数字乡村。推进农业农村大数据和重要农产品全产业链大数据建设,推广大数据、物联网、人工智能在农业生产经营管理中的应用,提高农机信息化水平,逐步建立农产品和投入品电子追溯监管体系,推动电子商务进农村,实施信息进村入户工程,构建“三农”综合服务平台,加强大数据应用,助力巩固拓展脱贫攻坚成果,推进乡村振兴。(责任单位:省委网信办、省发展改革委、省农业农村厅、省工信厅、省商务厅、省乡村振兴局)

建立智能化市场监管体系。提升政府对数字经济的统计监测和决策分析水平,扩大数字经济数据监测和采集范围,提高数字经济态势感知、风险预警和防范能力,提升数字经济市场监测水平。推动构建多元共治的协同监管机制,建立以信用为基础的市场监管体制。(责任单位:省统计局、省市场监管局)

专栏2 推进工业信息化智能化转型

实施智能制造专项。依托中电二所、复晟铝业、大运汽车、太钢不锈、智奇铁路、科达自控等重点企业,打造一批智能制造试点示范、标杆企业。到2022 年,省级智能工厂、数字化车间实现“双百”目标。发挥山西省智能制造产业技术联盟、省物联网和人工智能标准化技术委员会等平台作用,协同开展智能制造关键核心技术攻关、专家咨询诊断等工作。到2025年,传统制造业重点领域基本实现智能化制造。

(三)“数”:培育壮大新兴数字产业

推进数据资源集聚开放。按照集约、绿色、开放、共享原则,统筹布局数据中心建设,避免无序、低水平建设。推动百度云计算(阳泉)中心二期、山西中交高速数据中心、大同云中e谷大数据中心、环首都—太行山能源信息技术产业基地等项目建设。激发应用需求,集聚数据资源,推动政府部门、公共企事业单位的公共数据资源向社会开放,建立公共数据资源负面清单,鼓励和引导社会化开发利用。建立公共数据全流程管理标准和制度规范,探索建立数据服务市场规则,规范市场交易行为,培育发展数据流通市场。(责任单位:省工信厅、省发展改革委、省审批服务管理局、省市场监管局)

加快发展大数据产业。以数据标注等产业为切入口,构建集数据采集、清洗、标注、交易、应用为一体的基础数据服务体系。开展大数据关键技术攻关,鼓励建设行业基础软件平台和重大集成应用平台,面向行业应用需求,形成垂直领域大数据解决方案。积极培育大数据产业基地,加快建设数字类产业园区,引入专业运营服务机构,推进构建智慧园区管理体系,加快培育太原、大同、阳泉、吕梁等大数据产业集群。(责任单位:省工信厅,山西综改示范区管委会)

大力发展网络安全产业。支持安全产品研发和产业化,以大数据安全、工业信息安全、物联网安全、人工智能安全、智慧城市安全等为重点,构建数字安全产业链,培育安全服务新业态,建设信息安全产业基地。加快安全核心技术研发,积极布局新型安全技术攻关。推进工业信息安全态势感知能力建设,培育建设一批网络安全技术、产品协同创新平台和实验室,推动产业共性技术研发和推广应用。(责任单位:省工信厅、省委网信办、省科技厅、省教育厅)

培育发展人工智能产业。积极探索创新人工智能领域数据服务模式、资金支持方式,推动建立完善相关法律制度。在交通物流、健康医疗、文化旅游、工业制造等领域建立专业数据集,形成基础数据能力。加强基础算法、应用算法研究,提升算法分析能力。鼓励开展云计算和边缘计算应用、超算中心建设,提升算力支撑能力。培育建设人工智能基础数据、安全检测等创新平台。鼓励在高精度传感器、智能机器人、智能网联汽车、智能物流、智慧医疗、智能文旅、智能制造等领域开展人工智能融合应用,加快培育发展人工智能产业。(责任单位:省工信厅、相关行业主管部门,各市人民政府,山西综改示范区管委会)

专栏3 发展特色数字产业

加快发展数据标注产业。以百度数据标注项目为依托,积极整合山西知网、迪 奥普等省内重点企业优势,在无人驾驶、空间地理、健康医疗、煤矿电力、知识挖掘等重点领域形成一批国家级专业数据资源集。到2022 年,引进培育百家以上数据标注企业,初步形成集数据采集、数据清洗、数据标注、数据交易、数据应用为一体的基础数据服务体系,推动人工智能产业发展。到2025年,成为全国领先的基础数据产业聚集地。

推动网络安全产业发展。依托山西综改示范区建设国家级网络安全产业基地,培育网络安全服务集群、生物识别产业集群、工业信息安全产业集群,构建研发设计、生产测试、人才培育、公共服务等产业支撑平台。依托云时代、中电科33 所、华北网安、圣点科技、中科天地等骨干企业,聚焦云服务与数据安全、电磁防护、工控安全、特色生物识别等领域,构建网络安全产业生态体系。到2025 年,培育形成一批年产值超过5亿元的网络安全骨干企业,将我省建设成为中部领先、全国重要的网络安全产业基地。

(四)“器”:提升电子信息产品制造能力

支持通用计算设备产业一体化发展。加大对通用计算技术、产品应用的支持力度,加快推进科研攻关与技术应用示范基地建设,围绕整机应用,落实有关专项扶持政策,培育构建中央处理器(CPU)、操作系统、数据库、应用软件、显示器材、办公外设等为一体的产业链条,打造通用计算设备产业集群。(责任单位:省工信厅、省科技厅,太原市人民政府)

推动传感器和智能硬件产业规模化发展。抓住5G、人工智能、物联网、工业互联网等关键发展机遇,加强军民融合、产学研用深度融合,强化整机带动,加强工业机器人、家用清洁机器人、高精度传感器、多模态生物识别、专用无人机等优势产品研发升级,重点打造敏感元器件、传感器、智能硬件、智能机器人产业链条。(责任单位:省工信厅、省科技厅)

推进光电信息产业集聚发展。以光学镜头、相机模组、光通讯连接器、机器人、锂离子电池等光机电融合产业为重点,加大自主创新和人才培养力度,大力提升智能制造水平,打造高端关键材料、智能工具、智能高端装备、光学核心元器件等产业链条,支持建设光机电产业集群。(责任单位:省工信厅、省科技厅、省教育厅)

(五)“芯”:支持半导体高端核心产业快速发展

打造全国领先的半导体产业集群。围绕5G、电力电子、LED等关键应用,重点支持碳化硅、氮化镓第三代半导体、砷化镓第二代半导体、红外探测芯片、深紫外LED 芯片等半导体产业发展,提升装备、材料、衬底、芯片、器件等核心关键技术和工艺水平,打造高纯半导体材料、衬底、外延、芯片、应用等全产业链产品体系,培育形成全国领先的半导体产业集群。(责任单位:省工信厅、省科技厅)

推动培育新型显示产业链。发挥我省在LED 显示、显示面板制造专用装备、激光导光板、激光投影、液晶显示材料等领域的发展基础,以4K/8K 超清显示、LED 显示、专用装备、显示材料、纳米陶瓷等领域为重点,大力培育和引进重大项目,完善产业链条,打造新型显示产业集群。(责任单位:省工信厅、省科技厅)

专栏4 电子信息制造业协同集聚发展

打造太原通用计算设备产业集群。加快建设科研攻关与技术应用示范基地,大力引进培育龙芯中科(太原)、国科晋云、山西百信等重点企业,加大各领域示范应用力度,协同开展技术攻关、集成适配等工作,培育构建软硬件相互配套、紧密协同的产业链条。

打造长治—晋城光电产业集群。依托中科潞安紫外光电、山西高科集团等龙头企业和重大项目,以深紫外 LED、LED 显示等产业为重点,突破深紫外 LED 芯片、MiniLED 等核心关键技术。加快晋城光机电科技园纳米陶瓷材料、高铁钢轨铣刀、高端硬质合金等项目产业化。促成智能制造产业中心、纳米光机电产业示范中心和高端人才培育基地项目落地,搭建金字塔状的研发生产应用模式。到2022 年,光电信息产业集群跻身全国先进行列。

打造太原—忻州半导体产业集群。依托山西烁科、中科晶电等重点企业,以碳 化硅、氮化镓第三代半导体、砷化镓二代半导体材料为重点,延伸上下游产业链条,提升装备、材料、芯片制造、封装、测试、设计等核心技术和工艺水平,打造全产业链产品体系,形成全国领先的半导体产业集聚区。

五、保障措施

(一)加强组织领导

将推动数字经济发展作为各级各部门的“一把手工程”,建立健全工作协调、监督考核等工作机制,明确部门分工,细化推进措施,制定年度行动计划,破除体制机制障碍,推动数字经济加快发展。借助外力外脑,加强战略研究,为全省数字经济发展提供决策支撑。(责任单位:省政府办公厅、省工信厅、省发展改革委,各市人民政府)

(二)优化市场环境

加快数字经济领域法制建设,鼓励支持数字经济领域的政策制度、体制机制创新,逐步建立健全数据资产管理制度,营造良好的营商环境。全面优化数字经济扶持政策,依托省级技术改造专项资金和促进大数据发展应用专项资金,支持数字经济发展,持续加大对数字技术研发、数字产业培育、数字化融合应用、数字类人才培养等的扶持力度。(责任单位:省工信厅、省司法厅、省财政厅、省科技厅,各市人民政府)

(三)打造创新平台

发挥政产学研合力,加快产业创新能力建设,鼓励省内企业、高校、科研机构加强多层次、多领域联合,围绕数字经济发展共性关键技术,建设协同共享的创新中心、工程研究中心、联合实验室,构建国家级、省级共性技术研发平台。设立大数据、人工智能等相关领域科技研发专项,形成支撑数字化转型的学科体系和产业创新体系。(责任单位:省科技厅、省发展改革委、省工信厅、省教育厅,各市人民政府)

(四)强化人才支撑

依托省内高校、培训机构等培育一批数字经济领域专业技术人才,鼓励支持企业引进一批战略科技人才、科技领军人才、紧缺创新人才。积极开展各级、各领域数字经济相关业务培训和职业技能培训,支持校企合作、入企实训、定向培养、工学结合的联合培养模式,鼓励开展创新创业活动,提升全民数字化素养和能力。(责任单位:省教育厅、省人社厅,各市人民政府)

(五)建立统计评价体系

构建全面系统反映本地数字经济运行和发展情况的指标体系、评估方法,加强数字经济发展情况和变化态势运行监测分析,及时发布全省数字经济发展情况,加强对各区域数字经济发展的科学指导。(责任单位:省工信厅、省统计局、省通信管理局)

**山西省加快推进数字经济发展的若干政策**

一、支持数字基础设施建设

1.推进数据中心高质量发展。支持数据中心参加战略性新兴产业用电交易,实现用户终端电价0.3元/千瓦时的目标(用电电压等级为110千伏及以上)。支持绿色数据中心创建、运维和改造,对获得国家绿色数据中心称号的给予一次性100万元奖励。

2.将通信设施建设纳入城乡基础设施建设规划。支持通信基础运营商及铁塔公司开展5G移动通信网络、基于IPv6的下一代互联网、移动物联网等新一代信息基础设施示范项目建设,给予不超过项目投资额的30%、总额不超过500万元的补助。支持在我省设立5G联合创新实验室。

3.支持重点行业、典型企业打造工业互联网企业内网标杆网络,支持中小企业参照标杆网络开展企业生产性网络改造,支持建

设跨行业、跨领域工业互联网重点平台或行业性工业互联网重点平台,建设和运营一批标识解析二级节点,给予不超过项目投资额

的30%、总额不超过500万元的补助。鼓励电信运营商优先保障工业企业网络服务,为工业企业推出更有针对性的优惠资费方案

和企业信息化综合解决方案。

二、推进经济社会智能化转型

4.对大数据产业、人工智能与实体经济深度融合、工业互联网、软件工程化能力、新型信息消费、网络安全技术应用等领域的国家级试点示范企业(项目)、优秀解决方案,分别给予一次性100万元、50万元奖励。

5.推进农业、工业、服务业等领域数字化转型,支持开展数字经济融合应用示范项目,认定后按照投资额的30%给予一次性奖励,最高不超过500万元。

6.推动工业企业数字化、网络化、智能化转型发展,全面推动智能制造,对认定为国家智能制造标杆企业、省级智能制造标杆项目、省级智能制造示范企业的,分别给予一次性300万元、100万元、50万元奖励;工业企业智能制造专家诊断和评估一次性补助不超过20万元。

7.对自主创新能力强、形成核心自主知识产权并快速产业化的数字经济领域相关产品、应用系统、工业App等研发推广应用项目,一次性给予不超过项目投资额的30%、总额不超过500万元的奖励。

三、加强引进和培育市场主体

8.设立省级人工智能基础数据产业发展引导专项资金,引导支持人工智能基础数据产业发展。具有引领性、牵引性的重大项目可“一事一议”重点支持。

9.世界500强、国家规划布局内重点软件(集成电路设计)企业、全国电子信息百强、软件百强、互联网百强在我省落户投资发

展数字化产业,经认定给予一次性200万元至1000万元奖励。

10.支持半导体、通信设备、人工智能、信息安全、传感器、计算机、光电、电子专用装备及关键电子材料、零部件、元器件等电子信息制造企业,年主营业务收入首次达到1亿元、5亿元、10亿元的,分别给予一次性奖补100万元、300万元、500万元;年主营业务收入1亿元以上且同比增幅在10%以上的,按照主营收入增量的3%予以奖励,最高不超过500万元。

11.对我省首次进入全国电子信息百强、软件百强、互联网百强的企业,分别给予一次性奖补200万元。对大数据企业主营业务收入首次达到1亿元、2亿元、3亿元、5亿元的,分别给予一次性奖补100万元、200万元、300万元、500万元。

12.鼓励初创大数据企业入驻政府投资建设的标准厂房和办公用房,省政府按年给予租金补贴。其中,300平方米以内房租全额补贴,300平方米至1000平方米房租减半补贴,补贴期不超过3年。

13.鼓励政策性担保机构加大对大数据产业知识产权质押贷款的担保支持力度,在担保风险分担比例和担保费率等方面给予最大限度支持。同时,对于资信良好、成长性好且经营规范的大数据企业,流动资金贷款200万元及以上的,按照当年新增流动资金贷款的5%给予贴息,每户企业每年最高不超过500万元,补贴期不超过3年。对于获得天使投资的大数据企业,给予所获投资额的10%、最高不超过100万元的一次性奖励。

14.鼓励大数据企业开展市场拓展,企业参与省外招标项目中标,单个中标合同金额达300万元及以上的,按合同完成金额的3%给予奖励。同一个项目、系统或产品最高奖励不超过200万元,同一企业年度最高奖励300万元。鼓励企业参加各类专业展会,对展位费的80%予以补贴,每家每年最高不超过10万元。

15.对首次通过能力成熟度模型集成(CMMI)3级、4级、5级认证的软件企业,分别给予10万元、20万元、30万元奖励;对首次通过信息技术服务标准(ITSS)运行维护标准、云计算服务能力标准符合性评估一级、二级、三级的企业,分别给予30万元、20万元、10万元奖励;对首次通过数据管理能力成熟度评估模型(DCMM)3级、4级、5级的企业,分别给予10万元、20万元、30万元奖励。由低等次向高等次升级的,奖励其差额部分。

四、鼓励数字经济创新发展

16.对牵头制(修)订数字经济领域相关国际标准、国家标准、行业标准和地方标准的企业或单位,在标准公告并执行后分别给予50万元、30万元、20万元和10万元奖励。

17.我省大数据企业、高校或研究机构,新认定为国家(国家地方联合)工程研究中心、技术创新中心、重点实验室的,给予一次性500万元奖励;新认定为省级工程技术研究中心、重点实验室的,给予一次性150万元奖励;新认定为国家级企业技术中心、省级企业技术中心的,分别给予一次性300万元、50万元奖励;新认定为高新技术企业的,给予一次性100万元奖励;新获得“国家知识产权示范企业”“国家知识产权优势企业”的,分别给予一次性50万元、20万元奖励。

18.培育建设大数据产业基地,打造创新产业政策、集约要素资源、构建产业生态的核心载体。支持产业基地引进专业运营服务机构,经认定,按照实际服务费用的50%给予奖励,每个基地每年奖励不超过200万元。

19.支持大数据产业基地建设技术研发、测试测评、标准验证等基础性、支撑性公共服务平台,经认定,按照平台初期建设费用的50%给予一次性奖励,最高不超过500万元;按照平台运营费用的50%给予奖励,每个平台每年不超过100万元。支持省内企业、高校和科研院所组成数字经济共享服务联合体,整合产学研平台资源,为产业数字化转型发展提供研发、合作、推广、培训等服务,每年优选一批联合体给予一次性奖补,每个最高不超过500万元。

20.支持我省行业协会、专业机构或企业在晋举办数字经济领域行业性大赛、产业大会、产业论坛等活动,营造发展环境,培育市场氛围。评估后按照活动费用的50%予以补贴,每场最高不超过50万元。

五、加强数字经济人才培养

21.支持各地对相关机关事业单位和企业人员开展公益性交流培训,组织人员赴先进地区交流学习,对经确认的培训项目,按

照培训费用的50%给予奖励。支持大数据产业基地建设大数据教育实训基地,为本地区大数据产业发展提供人才支撑,经认定,按照实训基地建设费用的30%给予一次性奖励,最高不超过500万元。

22.支持数字经济领域企业实施人才计划项目,对引进、培育数字经济领域技术、管理、市场和财务等优秀骨干人才的企业给予

补贴。支付骨干人才年薪在社会平均工资3倍及以上的,按照每人每年1-3万元给予补贴;承担骨干人才参加国内外高级培训或到国外合作企业工作进修费用的,按照所承担费用的50%给予补贴,每人次最高不超过10万元。科研院所转化职务发明成果收益给予参与研发的科技人员的现金奖励,符合税收政策相关规定的,

可减按50%计入科技人员工资、薪金所得缴纳个人所得税。支持数字经济领域省级研究生教育创新中心建设,对新认定的给予一

次性10万元奖励,对首次考核评价为优秀等次的给予一次性50万元奖励。

山西省人民政府

2021年8月1日

（22）江西省数字经济创新发展领导小组办公室关于印发《关于加快推进数字经济创新发展的若干措施》的通知

各设区市人民政府、赣江新区管委会，省直有关部门：

　　经省政府同意，现将《关于加快推进数字经济创新发展的若干措施》印发给你们，请认真贯彻落实。

江西省数字经济创新发展领导小组办公室

（江西省发展改革委代章）

2020年10月26日

**关于加快推进数字经济创新发展的若干措施**

　　为深入实施我省数字经济“一号工程”，提升数字经济产业链和供应链稳定性和竞争力，培育壮大新产业、新业态、新模式，激活消费新市场，促进形成以国内大循环为主体、国内国际双循环相互促进的新发展格局，培育壮大经济发展新动能。现提出如下政策：

　　一、增强数字技术创新能力

　　1.加快关键技术突破。探索建立开放的科技攻关新机制，梳理数字经济领域核心关键技术目录，组织实施数字经济领域重大科技研发专项和重点研发计划，对获得立项的项目在省级财政科技资金中给予支持。5G、虚拟现实等领域的科研院所、企业作为第一承担单位获批立项的国家科技重大专项、国家重点研发计划项目，按照该类国家项目的配套要求给予相应比例的资金支持。（责任单位：省科技厅）对符合条件的数字经济重大产业项目，在重点创新产业化升级工程中按程序予以优先支持。（责任单位：省工业和信息化厅）对获得国家技术发明奖或国家科学技术进步奖一等奖的虚拟现实等数字技术企业给予100万元、二等奖50万元的一次性奖励；获得省科学技术进步奖、省技术发明奖一等奖和二等奖的数字技术企业，由所在地政府给予配套奖励。（责任单位：各设区市政府）

　　2.强化创新平台建设。充分发挥创新平台在关键核心技术攻关的引领作用，对新认定的数字经济领域国家重点实验室、国家工程研究中心，国家产业创新中心、国家技术创新中心、国家制造业创新中心给予500-1000万元支持，对新认定的国家级科技创新平台预备队予以100-130万元补助，新认定的省级制造业创新中心给予250万元支持。新认定的国家级企业技术中心，省级企业技术中心、工程研究中心、重点实验室和技术创新中心给予30-100万元项目支持。（责任单位：省发展改革委、省科技厅、省工业和信息化厅按职责分工负责）

　　3.支持研发新产品。对经评审符合支持条件的数字经济领域重点新产品项目，鼓励地市采取后补助方式支持。（责任单位：省科技厅）支持数字经济领域产品申报省优秀新产品。（责任单位：省工业和信息化厅）对企业试制省级《首台（套）重大技术装备推广应用指导目录》装备产品，予以资金支持。（责任单位：省工业和信息化厅、省财政厅按职责分工负责）支持本地数字经济领域相关创新产品和解决方案进入公共财政投资类项目。（责任单位：省财政厅）

　　二、加速培育数字产业链

　　4.促进数字产业链集聚发展。落实产业链链长制，持续补链延链强链，培育若干个特色数字产业链。对新认定的省级数字经济创新发展试验区（基地）、数字经济特色小镇给予一定项目资金支持。（责任单位：省发展改革委）对经认定的数字经济领域国家战略性新兴产业集群、新型工业化产业示范基地、省战略性新兴产业集聚区、智能制造基地给予适当奖励。（责任单位：省发展改革委、省工业和信息化厅按职责分工负责）鼓励数字商务创新集聚发展，支持创建国家电商示范城市、示范县及数字商务产业园区，对新认定的国家级、省级电商示范基地给予50-100万元的一次性奖补。（责任单位：省商务厅）对新认定的以数字文化产业为主导的省级文化产业园区给予一次性奖补100万元。（责任单位：省委宣传部）

　　5.支持企业做大做强。实施企业梯次培育行动，遴选培育数字产业链领航企业、龙头骨干企业。对数字经济领域首次入选的江西省独角兽企业、潜在独角兽企业、种子独角兽企业、瞪羚企业、潜在瞪羚企业分别给予400万元、150万元、100万元、20万元、10万元的一次性奖励用于企业开展科技创新活动。企业首次进入我省独角兽名单的，鼓励所在设区市政府采取“一事一议”方式给予支持。（责任单位：省科技厅）支持数字经济领域企业打造国家级制造业单项冠军企业、省级“专精特新”、专业化“小巨人”企业。（责任单位：省工业和信息化厅）

　　6.鼓励各地招大引强。用足用好内陆开放型经济试验区平台，支持产业链企业引进先进技术、资金资本、高端设备等。对国内外数字经济领域制造业龙头企业在江西投资总额超过2亿美元（新设或增资）或30亿元人民币的项目，新设企业投产、增资企业扩产后，省财政按核定的实际引资金额2%的比例一次性奖励当地政府，最高奖励5000万元。鼓励各地对产业升级带动作用强、地方经济发展支撑力大的重大项目，“一事一议”确定扶持政策。（责任单位：省商务厅、省财政厅、各设区市政府按职责分工负责）

　　三、丰富数字技术应用场景

　　7.实施“上云用数赋智”行动。积极参与国家数字化转型伙伴行动。支持在产业集群、园区等建立公共型数字化转型促进中心，提升数字化转型公共服务能力和平台“赋能”水平。遴选数字经济百佳场景，组织研究数字化转型路线图、指引等，指导中小微企业数字化转型。（责任单位：省发展改革委）对国家工信部门认定的工业互联网试点示范项目、“5G＋工业互联网”示范项目，大数据产业发展试点示范项目、大数据优秀产品和应用解决方案等企业，并符合省申报条件的优先给予支持。对企业智能化技术和工业互联网改造项目年度固定资产投资额（指厂房和设备）达2000万元以上的，按程序经审核后予以支持。（责任单位：省工业和信息化厅、省发展改革委、省财政厅按职责分工负责）

　　8.加快虚拟现实推广应用。鼓励和引导虚拟现实在教育、医疗、养老、文化、旅游、工业、警务、军民融合等领域开展虚拟现实示范应用，由所在设区市给予资金支持项目，省级按规定给予配套支持。（责任单位：省工业和信息化厅、省科技厅、各设区市政府按职责分工负责）

　　9.支持移动物联网示范应用。推广智慧消防、智能制造、智慧水务、智慧警务、智慧城市、智慧教育等示范应用，对经认定为省03专项及5G示范应用项目，给予50-100万元支持。（责任单位：省科技厅）

　　10.推动5G融合应用。培育和创新一批5G应用场景，对经认定具有创新和重大推广价值的应用场景每个奖励30万。对经认定可复制性强、社会带动效应明显的5G行业应用项目，按照项目投入的15%给予不超过50万元的奖励。（责任单位：省工业和信息化厅）

　　11.支持新业态新模式发展。实施新业态成长计划，建立新业态成长型企业名录制度，每年择优扶持一批新业态项目，鼓励发展新个体经济、微经济、无人经济。（责任单位：省发展改革委）对获得国家级、省级电子商务示范企业（数字商务企业）分别给予20万元、10万元的一次性奖补。对南昌、赣州、九江跨境电商综试区引进的跨境电商龙头企业按稳定外贸增长政策给予融资贷款贴息补贴。（责任单位：省商务厅）支持行业龙头企业建设的公共服务云平台、共享经济平台、开放型数字化转型中心，经认定后，由有关专项资金予以补助。（责任单位：省发展改革委、省商务厅、省工业和信息化厅按职责分工负责）

　　四、加快新型基础设施建设

　　12.进一步加快项目建设。落实好新型基础设施建设三年行动计划，重点调度、重点推进数字基础设施建设。简化数字基础设施建设的审批流程，缩短各环节审批周期。符合条件的数字基础设施项目优先纳入省重大项目计划协调推进，给予用地用能等要素保障。对符合条件的新型基础设施项目建设给予地方政府专项债券资金支持，支持符合条件的项目开展基础设施领域不动产投资信托基金（REITs）试点。（责任单位：省发展改革委、省财政厅按职责分工负责）

　　13.进一步降低用电成本。鼓励符合条件的5G基站、数据中心用电参与电力市场化交易。用好停征地方水库移民后期扶持资金腾出代征资金，精准降低我省部分新兴产业（数字经济领域）用电成本。（责任单位：省发展改革委）

　　14.推进社会公共资源共建共享。将数字基础设施建设纳入国土空间规划，逐步建立跨行业基础设施“多规合一”体制机制。支持基站、机房建设，免费开放民用建筑和公共设施属于公共机构和政府全额出资的企业所属建筑物以及政府投资为主的公共设施。禁止单位、个人在通信基站、室内分布系统建设、运维过程中收取进场费、协调费等额外费用。（责任单位：各设区市政府、赣江新区管委会，省住房城乡建设厅、省自然资源厅、省交通运输厅、省教育厅、省卫生健康委、省广电局、省通信管理局）

　　五、支持数字经济人才队伍建设

　　15.加大高层次人才引进力度。实施“省双千计划”等重大人才工程，加大对数字经济领域高层次人才和团队引进的支持。支持数字经济领域企事业单位采取兼职、技术咨询、周末工程师、特岗特聘等方式引进急需紧缺高层次人才。鼓励市县落实人才优惠政策，吸引数字经济领域专业人才。（责任单位：省委组织部、省人力资源和社会保障厅按职责分工负责）

　　16.多层次培养人才。鼓励高校、职业院校开设数字经济相关专业，支持申报大数据、人工智能、虚拟现实等相关专业博士点、硕士点。（责任单位：省教育厅）支持数字经济领域专业技术人员、经营管理人才申报“西部之光”访问学者、“远航工程”等公派访学研修。（责任单位：省委组织部、省科协）

　　17.强化人才激励。支持数字经济领域高层次人才申报国家级和省重点人才工程。（责任单位：省委组织部、省人力资源和社会保障厅按职责分工负责）鼓励人才创新创业引导基金、人才银行优先支持数字经济领域人才创新创业。落实党委联系服务专家制度，加强与数字经济领域人才的联系服务。（责任单位：省委组织部、省人力资源和社会保障厅按职责分工负责）落实好虚拟现实产业人才队伍建设的若干措施，积极开展江西省虚拟现实产业创新创业优秀人才团队评选，给予项目资助。（责任单位：省委组织部、省工业和信息化厅按职责分工负责）

　　六、强化金融税收支持

　　18.进一步加大金融支持力度。充分发挥各级财政资金和政府投资平台的引导、带动和放大作用，撬动社会资本参与数字经济领域技术创新、产业化、应用示范、创新载体和产业园区建设。加快设立5G、虚拟现实、新型基础设施等专项基金。对成功上市的数字经济领域企业，按照相关规定享受奖励资金，鼓励企业所在地政府给予适当补助。将符合条件的数字经济领域企业优先纳入“财园信贷通”“科贷通”及全省产融合作主导产业重点企业支持对象。鼓励金融机构针对数字经济领域企业特点，创新金融产品和服务。（责任单位：省金融监管局、江西证监局、省财政厅、省工业和信息化厅、人民银行南昌中心支行按职责分工负责，各设区市政府，赣江新区管委会）

　　19.加大税收支持力度。认真贯彻《新时期促进集成电路产业和软件产业高质量发展的若干政策》，全面落实企业所得税、进口关税、增值税等各项财税政策。进一步落实高新技术企业、小微企业、研发费用加计扣除等各项税收优惠政策，激发数字经济市场主体活力。坚持包容审慎的原则，不得专门针对某一新兴业态、新型商业模式组织开展全面风险应对和税务检查。（责任单位：省税务局、省发展改革委、省工业和信息化厅按职责分工负责）

　　七、营造包容创新的发展环境

　　20.创新管理优化服务。率先在数字经济领域试行“极简审批”“容缺登记”制度。探索实行“包容期”管理，只要不违反法律法规，允许试错、宽容失败。本着鼓励创新的原则，推动省市联合制定分领域监管规则和标准。将数字经济工作纳入及时奖励范围，对各地在数字经济发展中工作推进有力、发展成效显著的按国家和省有关规定给予表彰奖励。对在服务数字经济工作中因先行先试出现的失误错误，按规定实施容错免责。（责任单位：省市场监管局、省人力资源和社会保障厅、省发展改革委按职责分工负责）

（23）河北省人民政府办公厅印发关于支持数字经济加快发展的若干政策的通知

（冀政办字〔2020〕172号）

各市（含定州、辛集市）人民政府，雄安新区管委会，省政府有关部门：

　　《关于支持数字经济加快发展的若干政策》已经省政府同意，现印发给你们，请结合实际认真贯彻落实。

河北省人民政府办公厅

2020年10月1日

**关于支持数字经济加快发展的若干政策**

　　为贯彻落实国家数字经济发展战略，深入实施《河北省数字经济发展规划（2020-2025年）》，紧紧抓住产业数字化、数字产业化赋予的机遇，着力壮大新增长点，形成发展新动能，制定如下政策。

　　一、支持数字基础设施建设

　　（一）保障数字基础设施建设空间。将以5G基站为代表的通信网络基础设施建设纳入县级以上国土空间总体规划及相关专项规划，按照项目建设时序同步保障电力、管道等配套设施建设。制定利用建筑物、构筑物、地下综合管廊等空间建设信息基础设施的设计、建设和验收标准、规范，实现信息基础设施与建筑物、构筑物、地下综合管廊等空间同步设计、建设和验收。建立公共资源合作共享机制，在保证安全运行的条件下，推动通信杆塔与电力、市政、交通等部门杆塔资源实现双向开放和一杆多用，鼓励探索多样化合作模式。（责任单位：省自然资源厅、省住房城乡建设厅、省通信管理局、省电力公司、冀北电力公司、省交通运输厅等部门，各市（含定州、辛集市，下同）政府，雄安新区管委会）

　　（二）降低数字基础设施运营成本。建立通信基站用电报审安装绿色通道，支持具备条件的5G基站转供电改直供电和参与电力直接交易。电网容量扩容时，预留5G基站用电量需求。对省大数据产业发展基地内的数据中心、灾备中心、超算中心等重点大数据企业（项目）和纳入省重点项目计划的大数据中心（项目）用电，可自愿选择执行工商业单一制或两部制电价。优先支持数据中心参加电力直接交易，鼓励可再生能源电力和火电打捆与数据中心开展交易。（责任单位：省发展改革委、省工业和信息化厅、省通信管理局、省电力公司、冀北电力公司等部门，各市政府，雄安新区管委会）

　　（三）支持数字基础设施应用示范项目建设。支持以企业为主体，围绕医疗、教育、交通、环保等领域开展基于5G、下一代互联网、物联网等数字基础设施的典型示范和应用试点，优先纳入省高技术产业化和应用示范项目计划。鼓励各地加强5G、人工智能、大数据、云计算创新应用项目谋划储备工作，完善前期手续，落实建设条件，优先给予资金支持。（责任单位：省发展改革委、省工业和信息化厅、省卫生健康委、省教育厅、省交通运输厅、省生态环境厅等部门，各市政府，雄安新区管委会）

　　二、提升产业数字化支撑和服务能力

　　（四）支持制造业数字化项目建设。围绕产业数字化关键环节，加快智能模块、工业软件、管理系统的集成，推动研发、设计、生产、营销、流通等全链条数字化转型。对传统制造业开展数字化改造的，省级工业转型（技改）专项资金按照“先投后补”方式支持项目建设，补助金额不超过已完成有效固定资产投资（不含土地、建筑物投资）总额的15%，单个项目支持额度不超过1000万元。（责任单位：省工业和信息化厅、省发展改革委、省财政厅等部门，各市政府，雄安新区管委会）

　　（五）支持工业互联网建设。支持工业互联网标识解析二级节点建设，鼓励重点企业打造工业互联网企业内网标杆网络。鼓励电信运营商优先保障工业企业网络服务，推出更有针对性的优惠资费方案和企业信息化综合解决方案。培育遴选一批技术先进、成效突出、可推广复制的工业互联网创新发展试点示范，按照每个试点示范信息化软硬件设备投资20%、总额不超过100万元给予资金补助。（责任单位：省工业和信息化厅、省通信管理局、省财政厅等部门，各市政府，雄安新区管委会）

　　（六）鼓励企业上云上平台。鼓励云服务商对上云企业给予一定幅度的优惠。培育一批专业能力强、运营模式好、带动作用大、年销售收入超亿元的数字化系统解决方案服务商，筛选一批典型案例和解决方案，列入全省推广清单。（责任单位：省工业和信息化厅、省财政厅等部门，各市政府，雄安新区管委会）

　　（七）加快发展软件和信息技术服务业。加强检验认证平台等公共服务能力建设，开发一批具有行业特点、技术优势的嵌入式软件、应用软件系统。鼓励工业技术软件化，支持面向特定行业、场景的工业APP开发应用。鼓励软件企业通过标准认证与评估提升企业核心竞争力，鼓励企业通过软件能力成熟度（CMMI）高等级认证、数据管理能力成熟度（DCMM）评估，纳入支持范围。推动关键软件技术突破、软件产业生态构建。鼓励省属国有大中型企业成立专业化软件和信息技术服务企业，开展社会化的软件业务和系统集成。（责任单位：省工业和信息化厅、省发展改革委、省科技厅、省财政厅等部门，各市政府，雄安新区管委会）

　　三、引进培育市场主体

　　（八）支持信息技术企业做强做优。对于我省首次进入全国电子信息百强、软件百强、互联网百强榜单的企业，给予一次性100万元奖励。对信息技术领域的重大项目在规划、用地、环评、用电等方面予以支持，优先纳入省重点建设项目计划，鼓励各地采取“一事一议”方式确定扶持政策。对信息技术企业中的增值税小规模纳税人按50%的税额幅度减征资源税（不含水资源税）、城市维护建设税、房产税、城镇土地使用税、印花税（不含证券交易印花税）、耕地占用税、教育费附加、地方教育附加（执行期按我省有关规定）。（责任单位：省工业和信息化厅、省自然资源厅、省生态环境厅、省发展改革委、省税务局等部门，各市政府，雄安新区管委会）

　　（九）引进优势企业和战略投资者。发挥省产业投资引导基金、战略性新兴产业创业投资引导基金、工业技改引导股权投资基金、科技创业投资和成果转化引导基金等引导基金作用，下大力引进国内外电子信息、软件、互联网等行业龙头企业，促进一批重大招商项目落地。股权投资基金投资省内数字经济企业期限满两年（含）以上的，除享受省财政金融合力支持企业发展专项资金奖励政策外，省战略性新兴产业发展专项资金对符合条件的项目，按照投资额的15%、最高不超过800万元给予补助支持。（责任单位：省商务厅、省发展改革委、省工业和信息化厅、省财政厅、省科技厅等部门，各市政府，雄安新区管委会）

　　四、加大技术创新投入力度

　　（十）支持关键技术攻关及转化。组织实施一批重大科技攻关项目，加强5G基站用核心材料及大功率GaN器件、北斗导航基带芯片和终端设备、新型显示关键材料和模组终端、钢铁大数据集成化应用、智慧绿色能源大数据、基于环保大数据的监控预警及溯源评估、机器人核心部件和集成应用、智能工厂、智能网联汽车等领域关键共性技术攻关，省级科技专项资金给予单个项目最高不超过1000万元的资金支持。对促成数字技术成果在我省转化的技术转移服务机构，省级科技专项资金按不高于技术交易额的1%、最高不超过50万元给予奖励。支持各地聚焦产业需求，积极争取国家科技重大专项、重点研发计划等专项支持。（责任单位：省科技厅、省工业和信息化厅、省财政厅，各市政府，雄安新区管委会）

　　（十一）完善公共服务平台。支持数字经济领域重点实验室、技术创新中心等创新平台建设，对晋级国家级的重点实验室、技术创新中心，省级科技专项资金给予300万元-500万元一次性奖励。支持建设一批省级数字化转型促进中心，支持其面向产业链上下游企业和行业内中小微企业提供需求撮合、转型咨询、解决方案等服务。定期对各类研发和公共服务平台开展服务能力测试和可信度评估，提升服务水平和服务质量。（责任单位：省科技厅、省发展改革委、省财政厅等部门，各市政府，雄安新区管委会）

　　（十二）加强知识产权创造激励。对数字经济领域新认定的国家知识产权示范企业、优势企业，获得省级专利奖的专利权人、发明人，省级知识产权保护资金给予奖励。对主持制（修）订数字经济领域相关国际标准、国家标准、行业标准的我省各类企业或团体，在完成标准制（修）订项目并发布后分别给予资金资助。（责任单位：省市场监管局、省财政厅，各市政府，雄安新区管委会）

　　（十三）引进高层次创新人才。对具有国内外首创重大技术发明或在关键技术上实现重大突破，且技术经济指标达到国际同类先进水平的高端创新人才或一流创新团队，来冀实施成果产业化项目，且符合省产业投资引导基金等政府引导基金投资方向及要求的，政府引导基金按照市场化方式给予积极支持。对其申报省级以上重大人才工程、重大科技专项、重大研究课题、重大创新平台，同等条件下优先予以支持。（责任单位：省发展改革委、省科技厅、省人力资源社会保障厅、省市场监管局、省教育厅等部门，各市政府，雄安新区管委会）

　　（十四）培养技能型专业人才。聚焦全省数字经济发展方向，支持省内重点高校加强数字经济新兴学科建设，优化专业结构和师资配备，加强数字经济领域相关专业人才培养。深化产教融合、校企合作，建设一批数字经济产教融合联盟和人才培育基地，谋划建设一批大数据学院、实训基地，培养大批量、专业化的职业技能型人才。推动软件龙头企业与高等院校深度合作，培育建设一批特色化示范型软件学院。（责任单位：省教育厅、省人力资源社会保障厅、省工业和信息化厅等部门）

　　五、构建数字经济发展良好生态

　　（十五）打造开放合作的知名品牌和载体。全力支持举办中国国际数字经济博览会，创新举办方式，丰富举办内容，进一步提升行业知名度和品牌影响力。支持省内重点企业、高校院所、行业协会积极承办全国性的行业级、专业级的数字经济展会、论坛、研讨会等活动，强化对外交流合作。定期举办全省性的数字经济创新创业大赛，支持一批优秀创新企业、优秀创新产品。（责任单位：省工业和信息化厅、省商务厅、省发展改革委、省教育厅、省科技厅等部门，各市政府，雄安新区管委会）

　　（十六）推动数字产业化聚集发展。鼓励全省大数据与物联网、电子信息制造业、人工智能与智能装备等数字经济产业向园区集聚。支持现有产业基地、园区创新适应数字经济特点的改革举措，延伸产业链条，吸引配套产业，完善数字化产业体系。对培育数字经济成效显著、聚集效应好、带动性强的省级战略性新兴产业示范基地，省级战略性新兴产业发展专项资金给予奖励。（责任单位：省发展改革委、省科技厅、省商务厅、省财政厅等有关部门，各市政府，雄安新区管委会）

　　（十七）支持典型应用场景建设。支持雄安新区数字经济创新发展试验区建设，探索数字经济生产要素充分流通机制，建立数据资产评估定价、交易规则、标准合约等政策体系。支持新型智慧城市建设试点，推动新一代信息技术与城市规划、建设、管理、服务和产业发展的全面深度融合。组织开展智慧冬奥典型应用、车联网和车路协同创新应用试点。（责任单位：省发展改革委、省委网信办、省住房城乡建设厅、省工业和信息化厅、省交通运输厅、省商务厅等部门，各市政府，雄安新区管委会）

（十八）营造鼓励创新的市场环境。创新数字经济领域新业态、新技术、新产品、新模式的管理方式，探索实行审慎包容的监管模式，推动由处理具体事项的细则式管理向事先设置安全阀及红线的触发式管理转变。（责任单位：省发展改革委、省市场监管局、省工业和信息化厅等部门，各市政府，雄安新区管委会）

（24）贵州省人民政府关于印发贵州省实施“万企融合”大行动打好“数字经济”攻坚战方案的通知 （黔府发〔2018〕2号）

各市、自治州人民政府，贵安新区管委会，各县（市、区、特区）人民政府，省政府各部门、各直属机构：

　　现将《贵州省实施“万企融合”大行动打好“数字经济”攻坚战方案》印发给你们，请认真贯彻执行。

贵州省人民政府

2018年2月7日

**贵州省实施“万企融合”大行动打好“数字经济”攻坚战方案**

　　为深入贯彻落实党的十九大精神和习近平总书记在贵州省代表团重要讲话精神，推进国家大数据综合试验区建设，加快大数据与实体经济深度融合，省人民政府决定在全省实施“万企融合”大行动，打好“数字经济”攻坚战，特制定本方案。

　　一、总体要求

　　（一）总体思路。围绕国家大数据战略和“数字贵州”建设，以应用为核心，深化云计算、量子通信、人工智能等新一代信息技术在实体经济中的创新融合，运用大数据手段推进全产业链、全生命周期以及企业研发、生产、销售、服务各环节优化重组，持续改造提升传统产业，不断培育壮大新业态，促进实体经济向数字化、网络化、智能化转型，由投资驱动、资源驱动向数据驱动、知识驱动转变，推动经济发展质量变革、效率变革、动力变革，为全省实施大数据战略行动、推进国家大数据综合试验区建设、加快转型升级和新旧动能转换提供强大支撑。

　　（二）基本原则。

　　--全面覆盖、分业施策。在全省各级各部门、各行业、各领域全面实施“万企融合”大行动，重点围绕一、二、三次产业中转型升级最急需、融合发展最迫切的各主导产业，分别明确融合方向，找准融合路径，提出融合措施，不断提升大数据融合应用的广度、深度、精度。

　　--研究问题、破解难题。深入研究大数据与实体经济融合发展中的重点难点，利用大数据技术和解决方案攻克关键瓶颈，着力解决企业生产管理粗放低效、产业融合创新能力偏弱、产业链协同发展水平不足等突出问题，增强融合应用的针对性和有效性。

　　--示范引领、典型带动。坚持项目化、实物化推进，分行业、分领域建设打造一批高水平的大数据与实体经济深度融合典型示范项目，形成可复制、可推广的融合技术、融合产品、融合模式和解决方案，带动技术更新、模式创新和产品供给革新。

　　--升级存量、培育增量。准确把握“大数据＋”、“＋大数据”两个方向。加快运用大数据改造传统产业，提升自动化、数字化、绿色化水平，促进核心竞争力升级，加快壮大物联网、人工智能、共享经济、区块链等新业态，优化实体经济结构，提升融合发展质量。

　　--企业主体、政府引导。充分发挥市场和政府“两只手”的作用，坚持以企业为主导推动大数据与实体经济深度融合，各级政府部门针对企业融合需求，优化政策、资金、要素等资源配置，引导和保障工作顺利开展。

　　（三）工作目标。2018-2022年，每年建设100个融合标杆项目，实施1000个融合示范项目。到2020年，带动5000户实体经济企业与大数据深度融合，20000户实体经济企业运用云服务开展融合，数字经济增加值占全省GDP比重达到30%。到2022年，带动10000户以上实体经济企业与大数据深度融合，数字经济增加值占全省GDP比重达到33%，引导推动各领域、各行业实体经济企业融合升级全覆盖。全省大数据与实体经济深度融合水平大幅提升，实体经济企业生产运营效率和产品服务供给质量明显提高，综合竞争力显著增强。

　　二、重点任务

　　（一）加快大数据与工业深度融合，推动工业向智能化生产、网络化协同、个性化定制、服务化延伸融合升级。

　　1.推进智能化生产。加快重点领域智能制造单元、智能生产线、数字车间、数字工厂建设，大力发展智能制造装备和产品，促进大数据、物联网、云计算、人工智能、北斗卫星导航等新技术、新设备在制造业的应用，推动企业全流程和全产业链智能化改造，重点实施以下专项：

　　（1）煤炭安全生产及智能化专项。结合能源工业运行新机制，建设贵州煤矿大数据服务平台，实现煤炭生产、管理等环节不同信息化系统的数据打通和在线监测，提供生产设备运行管理智能化、协同化服务，提升煤炭安全生产管理数字化、机械化、自动化水平。（责任单位：省能源局、省安全监管局、省大数据发展管理局、省经济和信息化委）

　　（2）制造业智能制造专项。加快以传感器、物联网为信息传输基础的智能网络及数字生产线建设，提升制造业企业全过程数字化建模与分析、智能化工艺决策、智能化现场运行管理水平。（责任单位：省经济和信息化委、省发展改革委、省大数据发展管理局）

　　（3）军民融合产业大数据应用专项。促进军工企业和军民融合企业广泛应用大数据，推动移动互联网、物联网等应用于军工安全保密、产品设计、生产制造、信息处理、市场开拓、售后服务等领域。（责任单位：省经济和信息化委、省发展改革委、省大数据发展管理局）

　　（4）电子信息产业生产经营全流程数字化专项。推动手机、电视机、平板电脑、电子元器件、集成电路等电子信息制造企业开展全业务流程数字化改造，实现关键工序、研发设计、生产制造和运营管理的智能化。（责任单位：省经济和信息化委、省发展改革委、省大数据发展管理局）

　　（5）有色产业产线质量数字化管控专项。推动电解铝企业对生产过程数据进行自动采集和分析，进行原材料自动配比，实现铝锭品级率的精准控制和产品质量证明书自动生成。（责任单位：省经济和信息化委、省发展改革委、省大数据发展管理局）

　　（6）民族制药产业生产过程自动化专项。围绕解决多组分中药提取纯化和质量控制难的共性问题，推进关键性工艺参数实时采集检测和分析调控，对提取、浓缩、醇沉、干燥等重点工段实施自动化改造提升，加快中药标准化、现代化进程。（责任单位：省经济和信息化委、省食品药品监管局、省大数据发展管理局）

　　2.推进网络化协同。加快发展协同研发、众包设计、供应链协同、云制造等网络化协同制造模式，推动生产制造、质量控制和运营管理全面互联。重点实施制造业网络化协同专项，在消费电子、机械、服装等行业推动网络协同设计、虚拟仿真、众包研发设计，在电子信息制造、有色、化工等行业开展网络化协同供应链管理。（责任单位：省经济和信息化委、省大数据发展管理局）

　　3.推进个性化定制。鼓励企业运用大数据挖掘和动态体验感知等手段，充分整合市场信息和客户个性化需求，挖掘细分市场，开展个性化定制服务。重点实施特色行业个性化定制专项，推动白酒、特色食品、服装、电子信息制造等行业引进定制解决方案和柔性生产设备，开展小批量、多品种、柔性化的定制生产，提供低成本、高质量、高效率的个性化定制产品和服务。（责任单位：省经济和信息化委、省大数据发展管理局）

　　4.推进传统行业服务化转型升级。鼓励传统企业依托自身优势资源与业务基础，推进研发设计、信息咨询、仓储物流、电商销售、设备租赁及维护等服务功能的商业化延伸应用，从产品制造型企业向制造服务型企业转变。重点实施传统行业服务化转型专项，推动化工、钢铁、装备、白酒、农产品加工等行业运用互联网开展产品全生命周期的管理增值服务，搭建工业电商平台，拓宽发展空间和产品渠道，培育“制造＋服务”跨界融合的新市场主体。（责任单位：省经济和信息化委、省大数据发展管理局、省商务厅）

　　5.发展工业互联网。加快推动低时延、高可靠、广覆盖的工业互联网网络基础设施建设，持续推进网络提速降费。加快推进标识解析体系应用，赋予工业互联网机器和产品“身份证”，为供应链系统和企业生产系统精准对接提供基础标识。以“工业云”为重点强化工业互联网平台支撑能力，鼓励龙头企业积极搭建企业级平台，形成各有侧重、协同集聚发展的工业互联网平台体系。通过工业互联网平台整合资源，构建设计、生产与供应链资源有效组织的协同制造体系，提升大型企业的集成创新应用水平，推动低成本、模块化工业互联网设备和系统在中小企业的普及应用。（责任单位：省经济和信息化委、省大数据发展管理局、省通信管理局）

　　2018-2022年，每年建设60个融合标杆项目，实施600个融合示范项目。2018年，带动900户工业企业与大数据深度融合；重点行业数字化研发设计工具普及率达50%，关键工序数控化率达35%；50%的煤矿实施智能化改造。到2020年，带动3000户工业企业与大数据深度融合；重点行业数字化研发设计工具普及率达74%，关键工序数控化率达58%；所有煤矿完成智能化改造升级；工业互联网平台体系初步形成，在工业领域依托工业互联网平台实现研发设计、生产制造、运营管理等业务规模化应用。到2022年，带动6000户工业企业与大数据深度融合。（责任单位：省经济和信息化委、省大数据发展管理局、省能源局、省安全监管局）

　　（二）加快大数据与农业深度融合，推动农业向生产管理精准化、质量追溯全程化、市场销售网络化融合升级。

　　1.推进农业生产管理精准化。加快构建大数据、云计算、互联网、物联网技术为一体的现代农业发展模式，实现现代农业生产实时监控、精准管理、远程控制和智能决策。重点实施农业脱贫攻坚专项，加快实现贫困农户建档立卡数据与农业生产数据的共享互联。建设农业产业脱贫攻坚大数据库和大数据平台，基于遥感监测、地面调查、网络挖掘等技术，构建“天空地人”四位一体的农业大数据可持续采集更新体系，夯实农业大数据基础，实现农业生产数据的关联整合、时空分析与智能决策，优化农业产业布局，深入推进农业结构调整，促进农业产业脱贫。（责任单位：省农委、省大数据发展管理局）

　　2.推进农产品质量安全可追溯。运用大数据实现农产品质量安全可追溯，形成生产有记录、信息可查询、质量有保障、责任可追究的农产品质量安全追溯体系。重点实施食用农产品质量追溯专项，聚焦茶叶、蔬菜、水果、禽蛋等特色产业，利用贵州省农产品质量安全追溯平台，通过农产品二维码，实现农产品产地、生产单位、产品检测等信息追溯查询，逐步纳入全国农产品质量安全追溯平台。（责任单位：省农委、省大数据发展管理局、贵州科学院）

　　3.推进农业市场销售网络化。积极培育农村电商主体，提升新型农业经营主体电商应用能力，建设信息开放共享、数据互联互通的农业电商公共服务系统，构建农产品冷链物流、信息流、资金流网络化运营体系，破解“小农户与大市场”对接难题，提高农产品流通效率。重点实施农村电商培育专项，加强与阿里巴巴、京东、苏宁、中信国安等电商平台合作，用好贵州电商云、贵农网等电商平台，建立产地仓等直采模式，发展绿色无公害优质农产品线上定制、线下送菜到家的农产品销售服务新模式，加快特色农产品线上推广和线下融合。（责任单位：省商务厅、省大数据发展管理局、省农委、省供销社）

　　2018-2022年，每年建设10个融合标杆项目，实施100个融合示范项目。2018年，带动150户农业企业与大数据深度融合；优化整合10个规模化农业物联网基地，建设50个农业企业产品质量追溯系统，建成10000个农村电商服务站。到2020年，带动500户农业企业与大数据深度融合；优化整合50个规模化农业物联网基地，建设200个农业企业产品质量追溯系统，实现行政村电商服务站全覆盖。到2022年，带动1000户农业企业与大数据深度融合。（责任单位：省农委、省商务厅、省大数据发展管理局、贵州科学院、省供销社）

　　（三）加快大数据与服务业深度融合，推动服务业向平台型、智慧型、共享型融合升级。

　　1.发展平台型服务业。加快旅游、物流、信息咨询、商品交易等领域平台经济发展，融合各领域基础网络、综合管控系统、流量监控预警系统、应急指挥调度系统、公共无线网络、视频监控系统、电商平台、微信平台、手机APP等应用系统为一体，将数据资源整合转化为新型融合服务产品，提升管理、服务、营销水平。重点实施全域旅游融合应用专项，建设智慧旅游“一站式”服务平台，推动数据收集、数据挖掘、融合应用，实现旅游产业监管、旅游产品推广、个性化服务预订等功能，构建全域旅游大数据综合分析管理体系，实现全省5A级景区全覆盖，形成智慧旅游景区规范，在全省推广。（责任单位：省旅游发展委、省发展改革委、省大数据发展管理局）

　　2.发展智慧型服务业。加快培育智慧物流、智慧商贸、智慧科技服务、智慧工业设计等智慧生产性服务业，持续壮大智慧健康、智慧医疗、智慧养老、智慧文化等智慧生活性服务业，推动服务业迈向高端化、智能化、网络化的智慧发展新形态。重点实施智慧物流融合应用专项，建设“贵州物流云”，依托省内大数据物流平台企业，推动物流追踪与物资管理、智能调度与高效储运等新技术应用，进一步降低空驶率，提高仓储周转效率，加快形成智慧高效、通达顺畅、绿色安全的现代物流服务体系。（责任单位：省发展改革委、省大数据发展管理局、省商务厅、省科技厅、省卫生计生委、省文化厅）

　　3.发展共享型服务业。引导推进全省生产、创新、生活领域资源网络整合重组，推动共享经济产品服务体系创新、平台创新和协同式生活方式创新，打造新的重要增长点。重点实施共享经济主体培育专项，大力发展交通出行、房屋住宿、生活服务、生产能力、医疗健康等领域的共享经济，加速建立共享经济发展相配套的社会信用体系、技术支撑体系和风险管控体系，健全相关市场准入机制、消费者权益保障机制和容错试错的审慎监管机制，有效激发共享经济发展活力。（责任单位：省大数据发展管理局、省发展改革委）

　　2018-2022年，每年建设30个融合标杆项目，实施300个融合示范项目。2018年，带动450户服务业企业与大数据深度融合；实现全省智慧旅游“一站式”服务平台5A级景区全覆盖，电子商务网络零售额同比增长30%，物流企业信息化率达80%。到2020年，带动1500户服务业企业与大数据深度融合；实现全省智慧旅游“一站式”服务平台重点景区全覆盖，物流企业信息化率达85%。到2022年，带动3000户服务业企业与大数据深度融合。（责任单位：省发展改革委、省商务厅、省旅游发展委、省大数据发展管理局、省科技厅、省卫生计生委、省文化厅）

　　（四）加快发展以大数据为引领的电子信息产业。

　　1.大力发展电子信息制造业。以智能终端为重点，加快打造贵阳、遵义、贵安新区等智能终端产业集聚区，支持终端芯片及智能终端产品技术研发和产业化，大力发展国产化ARM架构服务器处理器芯片、智能手机、平板电脑、智能穿戴、智能家电等数字终端产品。2018年，全省规上电子信息制造业增加值同比增长15%，到2020年年均增长13%。（责任单位：省经济和信息化委、省大数据发展管理局）

　　2.大力发展软件和信息技术服务业。增强大数据软件服务创新能力，积极推动软件开发、信息系统集成、集成电路设计企业发展，着力增强面向物联网、移动互联网的信息技术服务能力，大力发展数据库、行业应用软件和特色软件服务产品。研制面向贵州特色产业的供应链协同管控解决方案，助推传统产业融合转型。2018年，全省软件和信息技术服务业（全口径）收入同比增长30%，到2020年年均增长20%。（责任单位：省大数据发展管理局）

　　3.大力发展云计算产业。建设贵安新区超算中心等云计算重点项目，引进一批云计算平台企业入驻贵州·中国南方数据中心，面向政府和社会提供云计算服务。探索发放“云使用券”，鼓励助推企业需求上云、企业生产制造能力上云、公共服务能力上云、数据上云、资源上云、业务上云，形成大数据与实体经济深度融合应用服务体系及云生态环境。2018年，全省上云企业突破10000户，到2020年突破20000户。（责任单位：省大数据发展管理局、省投资促进局、省经济和信息化委、省农委、省发展改革委）

　　4.大力发展人工智能产业。依托贵阳市、贵安新区、遵义市等重点区域，规划建设一批人工智能产业园、人工智能创新示范基地、机器人产业园、人工智能研发中心等项目，加快推进计算机视觉、智能语音处理、生物特征识别、新型人机交互、智能决策控制等核心技术研发及应用，打造新一代人工智能产业链。（责任单位：省科技厅、省大数据发展管理局、省经济和信息化委）

　　5.大力发展物联网产业。组织实施国家物联网重大应用示范工程区域试点，在工业制造、生态环保、旅游文化、商贸流通、农业、建筑等重点行业开展一批物联网重大应用示范项目，推进贵阳市、贵安新区、遵义市等重点区域开展物联网应用试点，推动传感器、仪器仪表、多类条码、射频识别、多媒体采集、地理坐标定位等物联网智能感知技术和设备应用。（责任单位：省发展改革委、省大数据发展管理局）

　　（五）开展大数据融合高科技企业招商引资。

　　1.引进落地一批国内外优强高科技企业。结合“千企引进”工程，突出产业招商，依托《高科技产业招商目录指引》，分产业绘制产业链招商图，深度融入“一带一路”、长江经济带建设，用好数博会等重大交流合作平台。面向国内外高科技领先地区，梳理一批优强高科技企业以及运用大数据实施融合升级的传统领域重点企业名单，制定针对性招商政策以及个性化、专业化的招商服务方案，开展专题招商、精准招商、定向招商。2018年，全省引进落地优强高科技企业100户，其中引进国内外知名大数据龙头企业10户以上；到2020年引进落地优强高科技企业300户。（责任单位：省投资促进局、省大数据发展管理局、省科技厅、省发展改革委、省经济和信息化委）

　　2.引进发展一批潜力大成长性好的高科技企业。以产业招商为统领，突出大数据、人工智能、云计算、共享经济等领域，盯紧一批正在起步、成长快速、极具潜力的企业及创新创业团队，按产业链梳理形成招商项目库，分领域、分类别、点对点、人盯人开展招商洽谈，促进项目落地，培育贵州未来的高科技“小巨人”和“独角兽”。2018年，全省引进发展潜力大成长性好的高科技企业300户，其中培育大数据龙头企业20户以上；到2020年引进发展高科技企业700户。（责任单位：省投资促进局、省大数据发展管理局、省科技厅、省发展改革委、省经济和信息化委）

　　（六）完善大数据与实体经济深度融合支撑体系。

　　1.完善评估体系。完善全省大数据与实体经济融合指数评估体系，为全省企业提供融合发展水平、发展重点、价值成效、特征模式、发展趋势的评估诊断、结构性分析和对标性评估，突出问题导向，“一企一策”提出融合方向和融合重点，助推企业精准融合、转型升级，争取成为国家大数据与实体经济深度融合评估标准。（责任单位：省大数据发展管理局）

　　2.加快服务平台建设。依托现有云平台，在工业、农业、旅游、物流、电商等领域建设完善一批开放性、差异化、特色鲜明的行业融合大数据平台，整合“平台提供商＋应用开发者＋海量用户”生态资源，推进模型化、标准化和软件化，优化提升研发设计、生产制造、运营管理等资源配置效率，形成融合发展新生态。（责任单位：省大数据发展管理局、省经济和信息化委、省农委、省发展改革委、省商务厅）

　　3.研究推进一批关键技术和拳头产品。整合相关科研院所、实验室和市场力量，在各行业突破应用一批可复制、可借鉴、市场空间大、带动能力强的融合关键技术和软件产品，通过经验交流会、宣传推介会、专题推广会等形式，在各领域推广应用。重点支持高端工业软件、新型工业APP、工业操作系统及工业大数据管理系统的研发应用，打造新型研发设计模式、生产制造方式和服务管理模式。2018年，全省新建或提升1000个ERP（企业资源计划系统）、PDM（产品数据管理系统）、DNC（分布式数控系统）、MDC（制造数据采集管理系统）、MES（制造执行系统）、CRM（客户关系管理系统）、WMS（仓库管理系统）等应用系统，到2020年新建或提升3000个应用系统。（责任单位：省大数据发展管理局、省经济和信息化委、省科技厅）

　　4.研究推出一批融合解决方案。在各行业征集提炼系统融合解决方案，不定期发布优秀系统解决方案和企业典型方案，在全行业实现产品化推广应用。加强与既懂行业、又懂大数据的国内外解决方案服务商合作，加快集聚、不断拓展大数据与实体经济深度融合支撑服务企业队伍，充分借助丰富经验和先进技术，组建融合服务队和产业联盟，分行业开展精准业务对接和技术引进。（责任单位：省大数据发展管理局）

　　5.健全融合标准规范。依托融合标杆示范项目，编制一批行业通用、引领性强的融合标准规范，争取形成一批省级融合标准，在各领域、各行业推广应用，增强全省大数据与实体经济深度融合标准化、规范化水平，争取成为国家相关领域标准规范。（责任单位：省大数据发展管理局）

　　三、保障措施

　　（一）强化组织推动。省大数据发展领导小组负责统筹、协调、推动“万企融合”大行动实施。省大数据发展领导小组办公室承担日常工作，会同省有关部门制定年度工作计划，确保各项工作目标和重点任务顺畅推进、取得实效。省有关部门对本领域、本行业、本系统的“万企融合”大行动负总责，要明确责任领导、责任专班、责任事项和完成时限，结合自身职能，分别制定实施方案和年度计划，排出时间表、路线图，及时向省大数据发展领导小组办公室报送工作推进情况、存在的问题和工作建议，共同分析问题、破解难题，确保工作项目化、实物化落实。各市（州）政府、贵安新区管委会要主动扎实作为，建立工作机制，保障工作开展，特别是贵阳市、贵安新区、遵义市等重点区域要多挑担子、多做贡献，在产业融合、产业培育、招商引资、技术攻关、产品打造、平台建设等方面充分发挥好龙头引领和示范带动作用。（责任单位：省大数据发展管理局、省经济和信息化委、省农委、省发展改革委）

　　（二）强化职责分工。进一步细化分解“万企融合”大行动目标任务。工业领域的每年60个融合标杆项目和600个融合示范项目，以及到2022年的6000户融合企业，由省经济和信息化委会同省能源局、省安全监管局、省大数据发展管理局等部门牵头落实；农业领域的每年10个融合标杆项目和100个融合示范项目，以及到2022年的1000户融合企业，由省农委会同省商务厅、省大数据发展管理局等部门牵头落实；服务业领域的每年30个融合标杆项目和300个融合示范项目，以及到2022年的3000户融合企业，由省发展改革委会同省旅游发展委、省商务厅、省大数据管理局等部门牵头落实。各牵头单位要分别建立项目库，每季度将项目进度汇总到省大数据发展领导小组办公室。相关目标任务要细化分解到各市（州）、贵安新区、各区（县、市、特区），实现全面覆盖，突出区域重点，其中贵阳市、贵安新区、遵义市承担的任务不应低于全省任务的60%。各市（州）政府、贵安新区管委会也要建立本地区项目库，并督促县级政府指派专人包保，建立台账，精准指导企业开展融合工作，形成省、市、县分级负责、层层推动的工作机制。（责任单位：省大数据发展管理局、省经济和信息化委、省农委、省发展改革委）

　　（三）强化政策引导。省有关部门要充分利用现有资金渠道，创新投入方式，发挥好财政资金的引导、放大效应，通过以奖代补、贴息等方式，大力支持100个融合标杆项目建设。各市（州）政府、贵安新区管委会要切实加大“万企融合”大行动资金投入力度，重点支持1000个融合示范项目建设，并制定配套政策措施，引导带动区域内实体经济企业开展融合升级。要加大对“万企融合”大行动干部人才的培训力度，全年开展两轮专题培训，提高干部开展大数据与实体经济深度融合工作的能力。要面向重点人群开展数字技能培训，举办数字技能竞赛和各种体验活动，宣传引导并普及相关知识，提升公众数字技能水平。（责任单位：省大数据发展管理局、省经济和信息化委、省农委、省发展改革委、省能源局、省商务厅、省旅游发展委、省财政厅、省人力资源社会保障厅）

　　（四）强化督查考核。各地各有关部门要将“万企融合”大行动列入年度目标绩效考核，加强对各项工作任务的分类落实、动态跟踪和监督检查，对工作不实、工作不力的进行通报批评。依托“贵州省大数据与实体经济深度融合服务平台”，对市、县两级大数据与实体经济融合指数、工作绩效进行系统测评。做好“万企融合”大行动宣传报道，重点宣传工作成效、典型案例、先进事迹，营造“大数据大家干大家用”和比学赶超的良好氛围。（责任单位：省大数据发展管理局）

　　附件：1.贵州省实施“万企融合”大行动推动大数据与工业深度融合方案

　　2.贵州省实施“万企融合”大行动推动大数据与农业深度融合方案

　　3.贵州省实施“万企融合”大行动推动大数据与服务业深度融合方案

　　附件1

贵州省实施“万企融合”大行动推动

大数据与工业深度融合方案

　　为深入推进大数据战略行动，推动大数据与工业经济融合发展，培育融合发展新生态，根据《贵州省实施“万企融合”大行动打好“数字经济”攻坚战方案》，制定本方案。

　　一、工作目标

　　加快大数据与工业深度融合，重点围绕制造业、军民融合、电子信息、有色、民族制药、化工等主导产业，实现工业经济发展质量变革、效率变革、动力变革。每年实施60个融合标杆项目和600个融合示范项目，并进行复制、推广，以点带面促进本领域大数据应用水平提升。2018年，带动900户工业企业与大数据深度融合；到2020年，带动3000户工业企业与大数据深度融合；到2022年，带动6000户工业企业与大数据深度融合，推动企业生产经营数据云端聚集、分析应用。支持高端工业软件，新型工业APP、工业操作系统及工业大数据管理系统的研发应用，鼓励企业新建和提升一批ERP、PDM、DNC、MDC、MES、CRM、WMS等应用系统。

　　二、重点任务

　　（一）推进信息化与工业化融合专项。

　　1.煤炭产业安全生产及智能化改造。结合贵州能源工业运行新机制，建设贵州煤矿大数据服务平台，实现煤炭生产、管理等系统的在线监测，提供生产设备运行管理智能化、协同化服务，提升煤矿安全生产管理水平。提高全省煤矿采掘自动化水平，辅助系统智能化水平，推动全省煤炭产业从机械化向智能化转型升级。2018年，50%的煤矿实施智能化改造升级。2020年完成所有煤矿智能化改造升级。

　　2.制造业智能制造。加快以传感器、物联网为信息传输基础的智能网络和数字化生产线建设，提升制造业企业全制造过程的数字化建模分析、智能化工艺决策、智能化现场运行管理水平。

　　3.军民融合产业大数据应用。促进军工企业和军民融合企业广泛应用大数据，推动移动互联网、物联网等应用于军工安全保密、产品设计、生产制造、信息处理、市场开拓、售后服务等领域。

　　4.电子信息产业生产经营全流程数字化。推动手机、电视机、平板电脑、电子元器件、集成电路等电子信息制造企业开展全业务流程数字化改造，实现关键工序、研发设计、生产制造和运营管理的智能化。

　　5.有色产业产线质量数字化管控。推动企业对铝电解生产的过程数据进行自动采集和分析，根据铝水平工艺条件进行原材料自动配比，实现铝锭品级率的精准控制和产品质量证明书自动生产。

　　6.民族制药产业生产过程自动化。围绕解决多组分中药提取纯化和质量控制难的共性问题，推进关键性工艺参数的实时采集检测和分析调控，对提取、浓缩、醇沉、干燥等重点工段实施行动化改造提升，加快中药标准化、现代化进程。

　　7.制造业网络化协同。在消费电子、机械、服装等行业推动网络协同设计、虚拟仿真、众包研发设计，在电子信息制造、有色、化工等行业开展网络化协同供应链管理。

　　8.传统行业服务化转型。推动化工、钢铁、装备、白酒、农产品加工等行业运用互联网开展产品全生命周期的管理增值服务，搭建工业电商平台，拓宽发展空间和产品渠道，培育“制造＋服务”跨界融合的新市场主体。

　　（二）开展传统企业大数据运用能力建设。

　　1.提升企业对云技术的应用水平。推动企业能力云端聚集，支持企业开展产供销、测试认证、技术交易等服务在线化、平台化，促进企业需求、制造资源及能力、检测资源和服务等资源云端协同、开放、共享，通过线上线下相结合方式，提升企业快速响应和柔性高效的供给能力。推动云产品及服务在企业中的应用，支持企业通过购买、租用的方式使用微服务、轻应用类软件和服务，促进企业全业务流程向移动化和互联网化转型，降低企业信息系统建设成本，加快实现企业全流程数据云端汇聚和生产管理过程的数字化、网络化、智能化管控，帮助企业快速形成信息化能力。引导大中型企业信息基础架构和应用系统向云上迁移。支持大中型企业推进基础设施、管理、业务及综合集成应用上云，开展基于云平台的网络化协同、移动化办公和智能化改造，减少企业信息化投入。

　　2.推进企业网络化协同改造。推进企业全产业链改造升级，支持制造企业供应链内及跨供应链网络化协同，支持具备条件的大中型企业实施跨企业、跨区域的网络化协同制造工程，推动生产设备、研发设计、软件与服务等资源协作，加快实现传统的串行工作方式转变为并行工作方式，最大限度缩短产品研发和生产周期。推进企业智能化和数字化改造，加快打通企业内外信息流、数据流、业务流、资金流、知识流的协作链条，促进企业间需求与能力的智能优选和对接，推进企业资源高度共享、能力全面协同。加快推进信息系统综合集成，对处于信息技术单项覆盖阶段的企业，深化企业资源计划（ERP）、产品数据管理系统（PDM）、设备联网控制系统（DNC）、生产数据管理分析系统（MDC）、制造执行系统（MES）、客户关系管理系统（CRM）、仓储物流管理系统（WMS）等管理信息系统应用，逐步提高生产过程自动化控制水平，有序推进信息技术由单项应用向集成提升发展。

　　3.推进“个性化定制”服务。拓展个性化需求，通过工业云公共服务平台汇集需求、产品订单，开展小批量、多品种的协同设计、柔性生产，打造产品供给侧和需求侧沟通平台，加快推进企业柔性制造，开展精准营销、质量追溯、差异性服务，最大程度满足用户个性化定制需求。鼓励企业客户直接或间接参与产品设计和质量、进度监督，推动传统企业从被动生产向交互生产转化，逐步实现个性化、定制化的生产制造。推进个性化服务，鼓励企业运用移动物联网、大数据挖掘和动态体验感知等手段，采集、整合市场信息和客户需求，通过数据建模与分析，挖掘数据核心价值，推动生产管理与技术创新，对原有的业务服务流程及模式进行设计优化，提供低成本、高质量、高效率和用户满意的个性化定制服务。

　　4.培育系统集成商。依托航天云网、力源液压、阿里巴巴等企业在智能制造方面的技术、人才优势，在全省培育一批智能制造系统集成商，针对全省企业不同层次的智能化改造需求，量身打造智能制造解决方案。

　　（三）加快以电子信息制造业为主的新兴产业培育。

　　1.发展电子信息制造业。以智能终端为重点，加快打造贵阳、遵义、贵安新区等智能终端产业集聚区，支持终端芯片及智能终端产品技术研发与产业化，大力发展国产化ARM架构服务器处理器芯片、智能手机、平板电脑、智能穿戴、智能家电、服务器等数字终端产品。2018年，全省规上电子信息制造业增加值同比增长15%，到2020年年均增长13%。

　　2.发展工业互联网。加快推动低时延、高可靠、广覆盖的工业互联网网络基础设施建设，持续推进网络提速降费。加快推进标识解析体系应用，赋予工业互联网机器和产品“身份证”，为供应链系统和企业生产系统精准对接和人、机、物全面互联提供基础标识。以贵州工业云为重点强化工业互联网平台支撑能力，鼓励龙头企业积极搭建企业级平台，形成各有侧重、协同集聚发展的工业互联网平台体系。通过工业互联网平台整合资源，构建设计、生产与供应链资源有效组织的协同制造体系，提升大型企业的集成创新应用水平。通过推动低成本、模块化工业互联网设备和系统在中小企业中的部署应用，推动工业互联网在中小企业的普及应用。到2020年，全省工业互联网平台体系初步形成，在工业领域依托工业互联网平台实现研发设计、生产制造、运营管理等业务规模化应用。

　　3.发展工业品电商。推动化工、钢铁、装备、白酒、农产品加工等行业运用平台大力发展采购和营销两端的电子商务，带动上下游产业链产品及服务发展创新，实现线上线下（O2O）融合发展。依托贵州工业云搭建跨境电商平台，开展深度需求交互和精准大数据营销，依托个性化、网络化定制服务，提供企业发展空间和产品渠道，支持优强企业走出贵州，培育“制造＋服务”跨界融合市场主体。

　　（四）推进工业互联网网络信息安全工作。建立健全安全防护设施，强化技术防范，严格安全管理，切实提高防攻击、防篡改、防病毒、防瘫痪、防窃密能力。加强对工业云平台、工业控制系统，以及物联网应用的安全防护和管理，定期开展安全检查和风险评估，对工业控制重点领域使用的关键产品，定期开展安全测评，实行安全风险和漏洞通报制度。建立工业控制安全管理体系，以数据安全为基础，风险管理为核心建立健全数据安全管理体系、网络式安全保障体系等，切实保护数据安全。落实信息安全等级保护制度，强化网络与信息安全应急处置工作，完善应急预案，加强对网络与信息安全灾备设施建设的指导和协调。

　　三、保障措施

　　（一）强化组织统筹。建立省经济和信息化委、省大数据发展管理局与各级经信部门和大数据主管部门共同参与的协同推进机制，形成有点有面、点面结合的网状工作体系，为专项行动推进提供强有力的组织保障。

　　（二）强化责任落实。各市（州）、贵安新区经济和信息化部门和大数据主管部门要严格明确目标任务和进度要求，建立本地区项目库，积极推动实施，广泛组织企业开展标杆、示范项目申报，建立台账，指导企业开展工作，按月向省经济和信息化委、省大数据发展管理局报告工作进度。

　　（三）强化支撑体系。加大与省内外高校、科研院所、产业联盟、行业协会的合作力度，开展工业大数据理论、方法及关键应用技术等研究探索与推广应用，形成软件、硬件双结合的智力支撑体系、平台支撑体系、资金保障体系。

　　（四）强化保障能力。重点打造贵州工业云平台支撑能力，通过优化升级工业云，为企业提供更加丰富的产品、技术和服务。深入开展专家团队、支撑服务团队、解决方案、典型案例及标准规范等资源上云活动，以线上与线下相结合的方式为企业提供全方位支撑和服务。

　　（五）强化项目支持。发挥两化融合专项资金、信息化专项资金等专项资金的引导作用，打造试点示范，形成带动效应。鼓励企业租赁、购买云端产品服务、企业数字化智能化软硬件及解决方案等，降低企业上云和升级改造成本，帮助企业快速提升信息化能力。

　　（六）强化宣传培训。建立“走出去、引进来”交流学习机制，用好媒体和宣传渠道，宣传推广大数据＋工业发展和应用典型案例及做法并向全省推广。积极向上级机关推送优秀成果，借助两化融合贯标、工业云培训等渠道，形成行业、企业案例库并开展宣传推广。

　　附件2

贵州省实施“万企融合”大行动推动

大数据与农业深度融合方案

　　为深入推进大数据战略行动，推动大数据与农业深度融合，培育贵州现代山地特色高效农业发展新动能，根据《贵州省实施“万企融合”大行动打好“数字经济”攻坚战方案》，制订本方案。

　　一、工作目标

　　重点围绕农业各主导产业，2018-2022年，每年建设10个融合标杆项目，实施100个融合示范项目。2018年，带动全省与大数据深度融合的农业企业达150户；优化整合10个规模化农业物联网基地，建设50个农业企业产品质量追溯系统，建成10000个农村电商服务站。到2020年，带动全省与大数据深度融合的农业企业达500户；优化整合50个规模化农业物联网基地，建设200个农业企业产品质量追溯系统，实现行政村电商服务站全覆盖。到2022年，带动全省与大数据深度融合的农业企业突破1000户。实现现代农业发展质量变革、效率变革、动力变革。

　　二、重点任务

　　（一）推进农业生产管理精准化。加快构建大数据、云计算、互联网、物联网技术为一体的现代农业发展模式，实现现代农业生产管理的实时监控、精准管理、远程控制和智能决策。重点实施农业产业脱贫攻坚专项，加快实现贫困农户建档立卡数据与农业生产数据的共享互联。建设农业产业脱贫攻坚大数据库和大数据平台，基于遥感监测、地面调查、网络挖掘等技术，构建“天空地人”四位一体的农业大数据可持续采集更新体系，夯实农业大数据基础，实现农业生产数据的关联整合、时空分析与智能决策，优化农业产业布局、深入推进农业结构调整、促进农业产业脱贫。

　　（二）推进农产品质量安全可追溯。运用大数据，实现农产品质量安全可追溯，形成生产有记录、信息可查询、质量有保障、责任可追究的农产品质量安全追溯体系。重点实施食用农产品质量追溯专项，重点聚焦茶叶、蔬菜、水果、禽蛋等特色产业，利用贵州省农产品质量安全追溯平台，通过农产品二维码，实现农产品产地、生产单位、产品检测等信息追溯、查询，逐步纳入全国农产品质量安全追溯平台。

　　（三）推进农业市场销售网络化。积极培育农村电商主体，提升新型农业经营主体电商应用能力，建设信息开放共享、数据互联互通的农业电商公共服务系统，构建农产品冷链物流、信息流、资金流网络化运营体系，破解“小农户与大市场”对接难题，提高农产品流通效率。重点实施农村电商培育专项，加强与阿里巴巴、京东、苏宁、中信国安等电商平台合作，用好贵州电商云、贵农网等电商平台，建立产地仓等直采模式，发展绿色无公害优质农产品线上定制、线下送菜到家的农产品销售服务新模式，增强特色农产品线上推广和线下融合。

　　三、保障措施

　　（一）强化组织领导。在省大数据发展领导小组统一领导、协调下，成立由省大数据发展管理局、省农委、省商务厅、省扶贫办等部门共同组成的联席办公室，建立跨系统、跨部门的一体化合作协调机制，确保各项工作目标和重点任务顺畅推进、取得实效。

　　（二）强化职责分工。各市（州）政府、贵安新区管委会要分别建立项目库，每季度将项目进度汇总到省农委。相关目标任务要细化分解到各区（县、市、特区），并督促县级政府指派专人，建立台账，精准指导企业开展融合工作，形成省、市、县分级负责、层层推动的工作机制。

　　（三）强化资金保障。按照投入多渠道、主体多元化、形式多样化的思路，积极引导多种经济成分的资金、技术等要素投入，形成政府引导、社会力量积极参与的投入机制。各级财政资金重点应投向农业大数据平台、农业物联网基地、农产品质量追溯、农村电商等项目的建设与运营发展。

　　（四）强化督查考核。各地各有关部门和单位要增强任意识，把“万企融合”大行动纳入农业发展布局规划和年度工作计划。要压实责任分工，加强工作任务落实、动态跟踪和督促检查，将相关任务纳入对各部门的年度绩效考核，开展动态监测，落实考核奖惩。

　　附件3

贵州省实施“万企融合”大行动推动

大数据与服务业深度融合方案

　　为深入推进大数据战略行动，加快推动大数据与服务业深度融合，根据《贵州省实施“万企融合”大行动打好“数字经济”攻坚战方案》，制订本方案。

　　一、工作目标

　　2018年，带动全省与大数据深度融合的服务业企业达450户；到2020年，带动全省与大数据深度融合的服务业企业达1500户；到2022年，带动全省与大数据深度融合的服务业企业达3000户。围绕服务业各主导产业，实施一批融合标杆项目和示范项目，培育一批融合典型企业，形成一批先进技术、产品、模式，推动大数据在服务业领域应用向纵深拓展。到2022年，以大数据为依托的创新创业蓬勃发展，成为推动服务产业转型升级的重要动力；大数据与服务业融合的深度和广度不断深化，带动服务业领域全要素生产率大幅提高，实现服务业发展质量变革、效率变革、动力变革。

　　二、重点任务

　　（一）全域旅游融合应用专项。以搭建智慧旅游“一站式”服务平台为重点，深化大数据在旅游业领域的能力建设，构建全域旅游大数据综合分析管理体系，加快形成智慧旅游景区指导性规范，逐步扩大覆盖面。2018-2022年，每年实施10个融合标杆项目，实施100个融合示范项目，2018年“一站式平台”实现全省5A级旅游景区全覆盖，2020年实现全省A级旅游景区全覆盖，逐步建设并完善全域旅游大数据综合分析管理体系。

　　1.打造贵州智慧旅游“一站式服务平台”。以全域旅游综合管理、游客服务、产业协同为突破，着力打造完善智慧旅游“一站式”服务平台，优化“一站式平台”架构、运营模式、数据资源建设，形成全省推广的规范标杆。逐步开发添加数据收集传递、挖掘处理、融合应用等模块，加快实现旅游产业监管管理、旅游产品推广、个性化服务预订等功能。

　　2.提升全省旅游信息化基础建设水平。加强旅游景区信息化基础设施建设，加强旅游景区的游客集中区域、环境敏感区域、旅游危险设施和地带监控设施建设。推进旅游目的地数字系统建设，积极利用大数据、互联网、移动互联网等新技术，实现重点旅游景区数字化应用。加强以“云游贵州APP”为龙头的旅游展示、营销网络建设，不断提高全省旅游网络营销水平。

　　3.大力推动大数据与旅游实体经济深度融合。促进大数据技术渗透旅游产品创新、大数据产品支撑旅游产业升级、大数据思维引领旅游企业转型。引导旅游实体经济利用大数据技术改造传统经营管理模式，提高信息化、数据化水平。推动旅游产业实体项目与大数据项目融合建设，建设推出一批旅游实体经济与大数据融合优秀示范项目和优秀企业。

　　（二）智慧物流融合应用专项。发挥信息化技术在资源整合和供应链物流中的积极作用，积极推进公共物流信息平台、物流园区和物流企业信息化建设，推动物流服务模式创新，加快形成智慧高效、通达顺畅、绿色安全的现代物流服务体系。2018-2022年，每年开展10个融合标杆示范项目，100个融合示范项目。到2018年，全省物流企业信息化率达80%，到2020年，物流企业信息化率达85%。

　　1.搭建省级智慧物流信息平台。依托云上贵州政府平台，持续推进“贵州省物流云”等公共信息平台建设，进一步整合铁路、公路、水运、航空及邮政快递、仓储等行业物流信息，加快省域交通运输、物流、仓储、配送等数据集聚，形成集物流信息发布、数据交换、跟踪追溯、智能分析等功能为一体的物流信息服务中心。

　　2.提升物流园区信息化水平。鼓励第三方物流信息服务平台在物流园区的运用，支持物流园区物流信息化建设，进一步提升获取和分析园区企业、货主、司机、货车等相关数据的能力，加快推进各物流园区与省物流云平台实现物流数据互联互通。

　　3.增强物流企业信息化能力。鼓励物流企业加快推进物流信息化建设，支持物流企业大力推进基于大数据、云平台、物联网等信息化技术的供应链管理进程，加快实现物流企业与省物流云、物流园区之间实现互联互通，进一步推动物流业跨越式发展。

　　（三）推动大数据与电子商务深度融合。抓住“新零售”为我省发展生鲜农产品电商带来的机遇，以电商示范县、示范基地建设和电商支撑体系建设为抓手，推动电子商务向乡村、农产品生产基地、社区延伸。2018年网络零售额同比增长30%。2018年-2022年，每年实施10个融合标杆项目，100个融合示范项目。到2022年，电子商务对大数据的应用不断深化，不断提升电子商务的个性化营销、精准营销能力。

　　1.建设一批电商示范县和示范基地。到2020年，全省国家级电子商务进农村综合示范县实现国贫县全覆盖，县级电子商务公共服务中心覆盖100%，示范县建制村电子商务综合服务站点30%覆盖。抓好电子商务进农村示范县评选和绩效评价。切实做好国家级电子商务进农村综合示范县评选。继续建设一批示范基地，以示范带动我省电子商务持续发展。

　　2.推进电子商务助力脱贫攻坚。结合农产品“泉涌”工程，全力推进农村电商建设，以“黔货”品牌塑造、市场主体培育、物流体系建设、人才培养引进为抓手，实现带动建档立卡贫困人口就业增收、脱贫致富目标。加强冷链体系、服务体系、质量追溯体系建设，奠定农村电子商务精准扶贫发展基础。以整合产业链和建立产销对接新模式为重点，突出贫困户在农产品利益链中的位置，增加收益。引导传统制造业利用电子商务中间环节少、地理限制小和销售渠道广的优势，扩大生产规模，为贫困户提供更多的就业岗位。

　　3.推进电商向社区渗透。大力发展电商O2O，推动省内大型农贸市场信息化改造，利用信息化手段优化消费环境，提升终端配送能力，在中心城市的大型社区增设冷柜、自提柜、生鲜车等设施设备，最大限度地为消费者提供便利。联合省外大型企业建立省外销售终端，将优质农产品直销城市社区，缩短中间流通环节，拓宽我省优质农产品销售渠道。

　　（四）实施共享经济主体培育专项。引导推进全省生产、生活和社会管理领域资源网络整合重组，推动共享经济产品服务体系创新、平台创新和协同式生活方式创新，成为新的重要增长点。2018年-2020年，每年引进一批共享型服务业创新企业，培育一批共享型服务平台和“双创”平台。

　　1.拓展共享经济发展空间。重点围绕交通出行、房屋住宿、生活服务、生产能力、医疗健康等服务业领域，支持企业在服务业领域开展融合创新，培育形成一批业绩良好、极具发展潜力和培育价值的初创企业，培育贵州大数据未来的“小巨人”和“独角兽”，有效激发发展活力。

　　2.开展共享经济“寻苗行动”。重点围绕大数据、人工智能、云计算等技术领域，盯紧一批正在起步、成长快速、极具潜力的企业及创新创业团队，发展共享经济新业态，创新共享型服务产品，引进一批正在起步、成长快速、极具潜力的企业及创新创业团队落地贵州。

　　3.构建共享经济公共服务平台。推动企业围绕服务业重点领域建设公共服务平台，支持平台为企业提供公共服务，支持“双创”平台等公共服务平台快速发展，在服务业领域建设完善一批开放性、差异化、特色鲜明的行业融合共享型服务平台，建设形成一批具备一定公共服务能力的共享型平台，为共享经济发展提供服务支撑。

　　4.强化共享经济制度建设。加速建立与共享经济发展相配套的社会信用体系、技术支撑体系和风险管控体系，健全相关市场准入机制、消费者权益保障机制和容错试错的审慎监管机制，营造共享经济发展良好环境。

　　三、保障措施

　　（一）强化组织领导。在省大数据发展领导小组的统一协调下，充分调动各行业主管部门、各地方政府、相关企业的积极性，建立上下联动、协同推进、责任明晰的标杆项目和示范项目推进机制，加强配合、形成合力，适时发现、协调、解决我省在推动大数据与服务业深度融合发展工作中遇到的问题，推进融合工作顺利实施。

　　（二）强化资金保障。积极争取国家服务业相关政策、资金和项目支持，按照“渠道不乱、合力推动”的原则，统筹省大数据发展资金和各有关部门资金对标杆项目、示范项目予以奖补等各种形式支持。采取基金、事中事后贴息、先建后补、绩效激励、保费补贴、贷款风险代偿补偿等方式，引导社会资本和金融资本加大投入。加强政府、企业和金融机构的对接，引导金融机构加大对融合项目的支持力度。

　　（三）强化标准研制及应用。鼓励开展服务业重点行业应用的标准研制；在重点行业、领域、地区开展大数据关键、急需标准的应用验证，强化标准符合性检测，提升标准对市场培育、行业管理的支撑作用。加大对已有标准的宣贯力度。

（四）强化宣传引导。组织开展观摩会、经验交流会、座谈会等活动，对项目建设先进经验进行总结，对具有示范作用的项目和企业等典型案例，以及其他优秀成果进行多渠道宣传推广，充分发挥标杆项目、示范项目的先行、示范引领作用，扩大社会影响。

（25）福建省人民政府办公厅关于加快全省工业数字经济创新发展的意见

（闽政办〔2018〕9号）

各市、县（区）人民政府，平潭综合实验区管委会，省人民政府各部门、各直属机构，各大企业，各高等院校：

　　当前，新一轮科技革命和产业变革蓄势待发，全球数字技术加快与实体经济深度融合。我省正处在经济发展优结构、转方式、提质效、调动能的关键期，发展工业数字经济，推动实体经济和数字经济深度融合发展，是推动工业供给侧结构性改革、增强工业竞争优势的必由之路。为加快推动我省工业数字经济创新发展，提出以下意见。

　　一、总体要求

　　以习近平新时代中国特色社会主义思想为指导，深入贯彻党的十九大精神，按照高质量发展的要求，坚持新发展理念，以促进工业创新发展为主线，以推动工业数字化转型为主攻方向，加快构建以数据为关键要素、以数字技术创新为核心驱动力、以现代信息网络为重要载体的工业数字经济，提升工业供给侧结构性改革质量。

　　坚持创新引领与融合发展，坚持市场主导与政府引导，坚持包容审慎与安全规范，推动数字技术向工业各领域、各环节渗透，激发工业强劲发展动能。到2020年，产业规模持续壮大，电子信息产业规模超过1.2万亿元，年均增长12%以上。创新能力显著增强，在智能制造、物联网、大数据、人工智能等重点领域，突破一批核心技术，以数字技术创新为主要动能的工业新生态初步建立。数字化转型效果明显，互联网、大数据、人工智能和实体经济深度融合，工业企业智能化改造步伐加快，示范应用加快推进，新模式、新业态不断涌现。到2025年，工业数字经济生态更加完善，产业规模与创新能力走在全国前列。数据成为驱动工业创新发展的关键要素，人工智能等新兴数字技术深入应用，工业互联网建设全面推进，形成制造业数字化、网络化、智能化的发展体系。

　　二、夯实工业数字经济产业基础

　　着力电子信息制造业“增芯强屏”和终端产品创新，加快工业软件、物联网、大数据、人工智能等新兴技术产业化，推动信息技术产业高质量、集聚化发展。

　　（一）做强做优电子信息制造业

　　加快集成电路产业集聚。加强与龙头企业合作，积极对接国家集成电路基金，推进重大技术转移项目落地。扩大集成电路芯片28纳米制程量产规模，力争在国际领先的7／10纳米先进工艺上取得突破。推进存储器项目建设，实现F32纳米制程投产。加快6英寸砷化镓芯片项目投产，建设6英寸氮化镓、碳化硅项目。加强与封装测试龙头企业多环节合作，建设国际先进的封装测试基地，完善芯片设计、制造、封装测试的全产业链布局。

　　构筑新型显示产业链。推动福州8.5代线、厦门6代LTPS面板、莆田6代线、南平触摸屏等重大项目量产。策划引进一批产业链配套项目，逐步实现基板玻璃、光学膜、偏光片、驱动IC、整机生产等环节的完备产业链。支持AMOLED、MicroLED、3D显示等新型显示技术的研发和产业化应用。

　　加快计算机与信息通信产业升级。鼓励省内企业加快终端产品研发升级，巩固提升平板电脑、金融智能POS机、移动通信等智能终端产品的优势地位。拓宽与华为公司的合作领域，布局建设5G、IPv6等新一代网络通信项目。深化军民融合，加大北斗卫星导航、位置服务产品开发与应用推广。

　　壮大半导体照明产业。鼓励省内企业加强高光效外延片／芯片制造、高性能封装、中大尺寸背光源及蓝宝石、图形化衬底等关键技术研发。加快新产品、新项目集成创新，发展中高端、个性化、智能化照明产品和系统。重点跟踪石墨烯、量子点、OLED、紫外、深紫外、MicroLED等新兴照明材料创新，发展第三代半导体材料。

　　打造锂电池千亿产业集群。支持省内锂电池企业加快发展，带动上游关键材料、配套设备和下游终端应用产业集聚。鼓励建设锂电池制造公共服务平台与创新中心，加快关键材料研究应用，开展基于石墨烯的电极材料改性研究，发展高端动力锂电池产业。

　　责任单位：省经信委、发改委、科技厅，省通信管理局，省电子信息集团，各设区市人民政府、平潭综合实验区管委会

　　（二）创新提升软件与信息技术服务业

　　积极发展IC设计业。依托福州软件园、厦门软件园等载体，开发设计需求量大、市场面广的多媒体芯片、移动智能终端芯片、数字电视芯片和网络通信芯片。加快发展基于新业态、新应用的信息处理、传感、新型存储等关键芯片。推动成立集成电路设计与应用解决方案联合研发中心。跟踪基于量子计算的芯片研究。

　　培育工业高端软件。针对我省电力、钢铁、石化、装备、纺织、食品等重点行业，鼓励软件企业跨界融合，推动基于网络的协同创新，加快工业软件自主研发。促进省内工业软件企业并购、重组，吸引国内外知名企业来闽设立研发机构。发展和推广省内工业控制通用技术平台以及数字化设计与仿真分析、MES、PLM等工业软件。实施工业APP工程，构建工业数据服务平台。

　　培育壮大动漫游戏产业。支持福州、厦门等地动漫产业集聚发展，打造动漫公共技术研发中心和动漫游戏版权交易等公共服务平台。推动动漫内容创作、形象设计、版权交易的发展，培育拥有自主知识产权、具有较强影响力的动漫形象和品牌。支持原创动漫游戏产品出口，扶持一批掌握核心技术的内容提供商和服务运营商开拓国际市场。

　　提升软件园区发展水平。支持福州、厦门建设“中国软件名城”，鼓励泉州、莆田、南平、平潭综合实验区等地建设软件园。加强对软件园区发展的统筹指导，建设一批重点软件研发机构、创新中心与公共技术平台。支持条件成熟的软件园区拓展发展空间，推动“一区多园”发展模式创新。对软件园区范围内企业税收的地方留成，应安排一定比例设立园区产业发展资金池，由园区管委会统筹用于产业发展。对处于开发建设期、投入大、税源少的园区，各级政府部门应加大财政资金保障力度，同时省级专项资金视具体情况予以一定资金扶持。

　　强化软件园区要素保障。优化软件园区用地保障，软件园区重点研发基地、创新平台、应用示范等纳入省重点项目管理，年度新增建设用地指标由省级统筹优先安排。实行软件企业用电扶持政策，鼓励符合条件的软件企业与发电企业开展电力直接交易；2020年12月31日前，对福州、厦门、泉州、莆田和南平软件园区内的软件企业，或者软件及信息技术服务年收入2000万元以上的软件企业，参照我省大数据产业重点园区企业电价扶持政策执行；上述企业应当向电网企业直接报装接电，由电网企业直接抄表结算电费；南平市可根据当地情况，进一步研究调整峰谷电价的浮动幅度。

　　支持引进国内外知名企业。鼓励地方政府通过市场开放、资源开发、技术采购、服务外包等方式，吸引知名软件企业入驻软件园区，支持省内外软件企业深度合作，培育一批“单项冠军”和掌握核心技术的高成长企业。对新引进实际到位资本金达到5000万元以上、位居国内前100强或全球前500强、具有核心技术的知名软件企业，依据企业水平和贡献程度，由市、县（区）受益财政给予一定奖励。

　　扶持软件企业做大做强。对软件园区内的软件企业主营业务收入首次超过2亿元、4亿元、10亿元的，由市、县（区）受益财政分别给予企业30万元、50万元、100万元奖励。对重大企业兼并重组项目的评估、审计、法律顾问等前期费用及并购利息，由各地统筹省级切块下达的资金予以补助，单个项目最高不超过300万元；对并购境外或省外科技型企业，分别按照并购金额的10%和5%由各地统筹省级切块下达的资金予以补助，单项最高不超过1000万元。鼓励软件园区管委会结合自身实际，对软件企业通过CMM、CMMI、信息系统集成资质等认定的给予一定奖励。

　　支持创新创业平台建设。将软件业纳入制造业创新中心试点培育范围，优先支持软件园区骨干软件企业联合相关企业、科研机构组建软件创新中心。优化软件行业企业技术中心评价方式，培育省级软件企业技术中心。支持在软件园区内建设提升一批软件业众创空间、孵化器和加速器，完善双创服务体系。对通过审核认定的孵化器，由省科技厅给予孵化用房补助；对评为省级、国家级孵化器的，一次性分别奖励50万元和100万元。对经认定的省级小型微型企业创业创新基地，由各地统筹省级切块下达的资金给予一定奖励。

　　完善人才评价和激励方式。鼓励地方政府、软件园区管委会参考《福建省引进高层次人才评价认定办法（试行）》的人才分类，突出软件行业特点和人才业绩与贡献导向，因地制宜制定软件人才评价标准，2020年12月31日前，对符合条件的高层次软件人才按实际缴纳的个人所得税地方留存部分予以适当奖励。地方政府应当为符合条件的高层次人才居住、医疗、子女幼儿园、小学就学提供便利，鼓励地方政府在软件园区内设立优质幼儿园、小学分校（园）。

　　责任单位：省经信委、发改委、教育厅、科技厅、财政厅、国土资源厅、地税局、物价局、新闻出版广电局，省委人才办，省国税局，省电子信息集团、省电力公司，各设区市人民政府、平潭综合实验区管委会

　　（三）加快新兴信息技术产业化

　　壮大传感器与物联网终端产业。围绕信息通信、汽车电子、医疗电子和工业电子应用领域，大力发展高性能、低成本、低功耗传感产品。支持物联网园区基地、产业联盟、技术研发等公共平台建设。支持福州市加快发展物联网感知产业，加强与窄带物联网标准制定及芯片设计龙头企业合作，推进高精度传感器、智能仪器仪表等技术研发与模式创新，建设物联网云创新中心。

　　促进大数据技术与服务创新。着力构建自主可控的大数据产业链、价值链和生态系统，以数据为纽带促进产学研协同创新和开源社区开放创新。加快数据存储、清洗挖掘分析、自然语言理解等大数据技术研发；支持发展分布式文件系统、海量存储数据库、搜索引擎、数据挖掘、数据可视化、数据安全保障等基础软件；发展大数据技术外包和知识流程外包服务，开发行业应用模型，培育数据采集、分析、运营等新业态，探索发展数据流通交易新兴服务。支持数字福建（长乐）产业园、中国国际信息技术（福建）产业园联合省内优势企业打造国家级大数据智库，推动省电子信息集团组建大数据交易中心，争取建成大数据流通与交易技术国家重点工程实验室。积极争取建设国家大数据综合试验区和国家新型工业化产业示范基地（数据中心），发展一批大数据骨干企业和创新型中小微企业。

　　完善虚拟现实产业链。依托数字福建（长乐）产业园已有基础，加快建设“中国？福建VR产业基地”。加强虚拟现实核心芯片、显示器件、光学器件、人机交互等关键技术环节的产学研联合攻关。培育虚拟现实内容生产制作和分发平台，发展内容素材采集、编辑、制作处理等技术设备，推动数字内容向虚拟现实产品移植，丰富娱乐文化等领域的内容供给。

　　探索区块链技术创新。挖掘区块链技术价值，鼓励企业加入开源社区，利用国际开源技术资源进行再创新，推动区块链在社会治理、资产管理、公示公证、社会救助、知识产权、工业检测存证等领域的应用。

　　责任单位：省发改委、经信委、科技厅、质监局，省通信管理局，省电子信息集团，各设区市人民政府、平潭综合实验区管委会

　　（四）推动人工智能研发和产业化

　　加快人工智能芯片与算法研发。支持省内优势芯片设计企业开展类脑芯片基础理论、类脑信息处理等前沿技术研究，推进类脑芯片的自主研发生产。支持开展以深度学习为核心的计算机视觉、语音识别、自然语言处理、生物特征识别、新型人机交互、自主决策控制等算法和新算法研发。推动算法创新与芯片设计联合优化，支持人工智能应用软件创新升级，开展针对垂直应用场景的专用人工智能芯片的研发和产业化，重点开发基于类脑芯片的智能系统以及AI终端解决方案，促进软硬件协同发展。

　　实施“先进制造＋人工智能”示范工程。支持省内优势科研机构、高端装备企业加强与人工智能企业合作，推动智能感知、模式识别、智能控制等智能技术的深入应用，提升数控机床、工业智能机器人等装备对工作任务的认知能力。鼓励机械装备、电子信息、纺织鞋服等行业率先推广应用人机协作、数据分析、智能感知等技术，培育一批人工智能创新应用试点企业。支持先进制造行业工艺流程、生产模型、行家经验等知识库建设，推动深度学习技术在智能装备柔性配置、制造执行系统优化等智能分析方面的应用，提高生产过程控制、远程诊断、供应链跟踪、质量管控等环节智能化应用水平。

　　实施“网联汽车＋人工智能”示范工程。支持省内整车企业与人工智能企业开展合作，建立联合实验室，推动无人驾驶等相关共性关键技术研发，鼓励建设以无人驾驶社会应用测试为目标的智能网联汽车综合示范区，推动基于网联汽车的人工智能数据模型研发。加快环境感知、智能辅助驾驶系统、车端智能操作系统、车载人工智能芯片、汽车网联数据平台、车辆数据分析模型等软硬件产品研发，开展智能客车整车生产销售，试点建设无人驾驶公交线路并逐步推广应用。支持建设人工智能新技术新装备的专用试验场地。

　　实施“泛家居＋人工智能”示范工程。鼓励省内优势家居企业集成应用人工智能技术，加快研发高感知、灵敏控制的传感设备和控制系统，提升家居产品的人机对话、行为交互、设备互联和协同控制等智能化水平。积极发展高端智能消费电子产品，加强面向智能家居的生物特征识别、三维扫码、图像感知等传感技术攻关。推动家庭智能机器人、智能网关等产品研发与应用。

　　实施“安防监控＋人工智能”示范工程。发挥省内安防企业优势，加强与人工智能企业合作，推动智能安防相关技术研发和产业化。支持面向工业安全、社会治安、自然灾害等领域智能感知技术的研发和成果转化，推进智能安防监控解决方案的应用部署。支持开展基于智能视频监控的公共安防区域示范，加快公共区域安防设备的智能化改造升级，加强社区信息的人工智能技术分析应用。

　　责任单位：省科技厅、发改委、经信委，省电子信息集团、汽车集团，各设区市人民政府、平潭综合实验区管委会

　　三、加快工业企业数字化升级步伐

　　深化信息化和工业化深度融合，加快数字技术与制造技术在更大范围、更深程度的渗透和创新应用，推动制造业加速向数字化、网络化、智能化发展。

　　（五）推动企业生产数字化转型

　　深入实施企业智能化改造。加快创建“中国制造2025”试点示范城市，深入开展“数控一代”示范工程，在石油化工、机械装备、建材家居、纺织服装、制鞋等优势行业实施数字化改造示范项目。加快大数据、物联网等新兴技术的深度集成应用，提升制造装备的数控化率和智能化水平，创新发展机床、工程机械、纺织机械等智能装备，推进生产全过程智能化。推动工业企业与大数据科研机构合作，推广应用工业大数据，监控生产过程及设备状态，优化生产工艺流程。分行业加快“机器换工”和智能制造样板工厂（车间）示范项目建设。

　　支持网络协同设计。推动高等学校、科研机构、工业企业、软件企业组建跨企业、跨领域网络协同设计中心，构建产业链协同研发体系。支持机械装备、纺织服装、制鞋、工艺美术等企业采用基于互联网的开放式研发设计模式，创建开放式创新交互平台、在线设计中心，发展客户深度参与的研发设计模式。支持大型工业企业建立全球协同设计平台，推进工业设计资源网上开放共享。依托福州软件园、厦门软件园培育在线设计服务商，打造众包设计平台，实现设计资源的集聚、共享和动态配置。

　　鼓励个性化定制生产。鼓励企业集成先进制造技术与互联网平台，面向市场多样化需求，利用互联网精准对接客户需求，推广让客户深度参与设计的生产模式，以大批量生产的成本和效率提供定制化产品。支持高端装备、船舶、工程机械等行业提升高端产品和装备模块化设计、定制化服务能力。支持发展面向纺织服装、制鞋、建材家居、工艺美术等优势行业的产品在线定制服务，培育个性化定制模式。

　　责任单位：省经信委、发改委、科技厅、教育厅，各设区市人民政府、平潭综合实验区管委会

　　（六）提升企业管理数字化水平

　　持续推动两化融合管理体系贯标，培育一批高水平两化融合咨询服务机构。支持企业导入两化融合管理体系，推动业务流程再造和组织方式变革，构建开放式、扁平化、平台化的组织管理新模式。进一步夯实企业信息化基础，支持企业实施信息化系统建设与整合，合理部署ERP、CRM、MES、PLM、SCM等系统。支持中小企业使用工业云平台，提高信息化应用水平。依托中国两化融合服务平台，引导企业开展周期性两化融合自评估、自诊断与自对标。支持石油化工、机械装备等行业企业开展信息化整体规划，建立企业数据资源目录体系。

　　责任单位：省经信委、发改委、科技厅，各设区市人民政府、平潭综合实验区管委会

　　（七）推动企业数字化服务化融合发展

　　培育一批智能制造咨询、设计与集成服务机构，提高整体解决方案与总集成总承包服务能力。引导有条件的企业设立软件与技术服务机构，输出成熟的信息化技术、产品和服务。支持工程机械、高端装备等企业向提供综合业务的服务商转型，提供产品监测追溯、故障诊断、远程维护、在线分析、大型设备融资租赁等服务。在福州、厦门、泉州、龙岩等地开展服务型制造试点示范工程。构建交通物流大数据中心。实施工业企业与物流企业联动试点示范工程，提升物流与供应链协同能力。

　　责任单位：省经信委、发改委、交通运输厅、商务厅，各设区市人民政府、平潭综合实验区管委会

　　四、打造数据驱动的工业新生态

　　突出数据的基础资源作用和创新引擎作用，实施工业互联网创新发展战略，加快完善数字基础设施，建设工业数字化服务平台，保障数据安全，形成数据驱动型创新体系和发展模式。

　　（八）构建数据驱动能力

　　鼓励厦门大学、中科院海西研究院等高等学校、科研机构，组建工业大数据研究中心，建设大数据应用试验平台，开展工业大数据研究。推进石油化工、汽车制造、工程机械、纺织服装等行业率先开展工业大数据应用创新试点，培育一批试点企业。支持企业提升工业数据采集、集成、分析挖掘能力，构建统一的数据采集平台，推动企业生产运营数据整合。支持和鼓励省内重点企业、科研院校主导或参与工业大数据国家标准、行业标准和地方标准的制修订。

　　（九）培育工业数字化服务平台

　　鼓励各类市场主体建设工业公共云服务平台，为中小企业提供企业管理软件、工业设计软件、工具库、零部件库等资源。鼓励大型企业建设工业数字经济创新中心、企业云服务平台，开放共享研发设计、生产制造、检验检测、工程服务等资源，推动企业资源在线发布与交易，服务中小企业发展。扶持中小企业上“云”，提升信息化能力。推动行业组织、龙头企业、互联网企业建设电子商务平台，促进互联网企业与实体经济深度融合，打造新型商业生态，重点面向工业品提供在线交易、支付结算、加工配送、质押担保等服务。

　　（十）推进工业互联网设施建设

　　加大工业互联网基础设施投资力度，推动省内数据中心和内容分发网络优化布局，完善工业互联网基础设施，进一步推进中小企业专线提速降费，推广工业互联网IPv6应用。鼓励有条件企业开展云平台互联互通改造，推动实现企业内部纵向集成、企业之间横向集成、产业价值链端到端集成，建设支持工厂透明化生产的信息物理系统，提升全产业链的要素资源配置效率。

　　（十一）增强工业数字安全能力

　　围绕新一代高端装备制造、工控系统、智能硬件、数据安全等领域，推动构建工业数字经济安全标准体系。加强安全态势感知、工控安全装备、可信芯片、安全测评等领域关键技术研发和服务创新，加快首台（套）推广应用，推进工业领域自主可控进程，开展工业数字经济安全试点示范。支持建立福建省工业数字安全测评服务机构。

　　责任单位：省发改委、经信委、科技厅、教育厅、质监局，省通信管理局，省电子信息集团，各设区市人民政府、平潭综合实验区管委会

　　五、优化工业数字经济发展环境

　　（十二）强化产业发展指导

　　组建工业数字经济产业发展专家咨询组，为宏观决策、项目策划、招商引资、政策研究等提供智库支撑。定期开展工业数字经济态势分析，引导产业健康持续快速发展。研究建立科学的评估指数和监测体系，开展工业数字经济运行监测，准确掌握数字经济发展动态。

　　（十三）强化治理创新协调

　　建立符合市场规律的工业数字经济治理创新协调机制，营造公平竞争秩序。深化“放管服”改革，优化市场主体审批服务，推行以负面清单为主的产业准入制度，包容鼓励新产业、新业态、新模式有序发展。

　　（十四）强化聚焦重点培育

　　完善“政产学研用金”多方参与的众创、众包、众扶机制。支持申报工业强基、产业振兴、工业转型升级等中央财政专项、新型工业化示范基地等示范项目。统筹用好工业和信息化专项资金、互联网经济新增引导资金、科技项目经费等省级财政专项资金，集中支持工业数字经济基础设施提升、公共平台建设、政府购买服务、人才引进培养、重点孵化项目、技术创新项目等。发挥各类产业基金“倍增器”作用，支持市场前景好的企业和项目加快发展。

　　（十五）强化精准招商引智

　　聚焦引进具备核心技术和领先优势的重点企业项目，加大力度开展“一把手”招商、龙头招商、园区招商、产业链招商和第三方招商。支持各类市场主体和中介组织引进高层次人才团队，推动项目成果转化和投资兴业。加强高等学校、科研院所学科专业建设和实用型人才培养，鼓励校企联合培养工业数字经济复合型人才、紧缺技能人才。

　　（十六）强化全面开放合作

　　支持省内企业与省外优势企业加强技术合作，开展工业数字经济协同创新和集成创新。深化闽台交流合作，推动集成电路、智能制造等高端技术和产业项目落地。响应“一带一路”倡议，主动融入全球产业生态圈，支持参与制定数字经济国际规则和标准，参与国际合作大型开源项目，推动平台型开源软件创新和应用开发。

　　（十七）强化示范宣传力度

　　加强工业数字经济试点示范，培育一批示范园区和示范项目，推广一批优秀成果与典型案例。举办数字经济专题培训班和研讨会，鼓励举办工业数字经济交流及赛事活动，吸引高层次人才来闽对接交流，扩大我省数字经济影响力，树立工业数字经济强省形象。

　　责任单位：省经信委、发改委、教育厅、科技厅、商务厅、人社厅、新闻出版广电局、统计局，省委人才办，省通信管理局，各设区市人民政府、平潭综合实验区管委会

福建省人民政府办公厅

2018年1月25日

（26）中共贵州省委、贵州省人民政府关于推动数字经济加快发展的意见

　　数字经济是指以使用数字化知识和信息作为关键生产要素、以现代信息网络作为重要载体、以信息通信技术的有效使用作为效率提升和经济结构优化重要推动力的一系列经济活动。为贯彻落实党中央、国务院加快发展数字经济的决策部署,培育我省发展新动能、拓展经济发展新空间,促进经济提速转型发展,提出如下意见。

　　一、总体要求

　　(一)基本思路。坚持创新驱动、市场主导、重点突破,突出引领性、应用性、支撑性,以数字化、网络化、智能化为指引,以发展数字经济重点型态为方向,以培育市场主体为抓手,以推动重点领域改革为突破口,以强化发展保障为支撑,共享数字红利,推动数字经济加快发展,深化供给侧结构性改革,充分发挥信息技术在资源合理配置和高效利用中的重要作用,促进全省经济社会发展质量和水平迈上新台阶。

　　(二)发展目标。到2020年,数字经济主体产业增加值年均增长20%以上,数字经济增加值占地区生产总值的比重达到30%以上,数字经济对国民经济发展的先导作用和推动作用进一步得到发挥。

　　——资源型、技术型、融合型、服务型“四型”数字经济发展明显加快。到2020年,智能终端产品制造产值达到1000亿元,集成电路产值达到250亿元,电子材料与元器件产值达到250亿元,电子商务交易额达到3600亿元,软件和信息服务业收入500亿元,通信和广电网络业务总量超过700亿元。

　　——外来企业、领军大企业、中小微企业、中介组织等数字经济市场主体活力明显增强。到2020年,集聚30家以上数字经济龙头骨干企业和200家以上“小巨人”企业。

　　——开放、创新、科技、营商、标准化、立法探索等数字经济重点领域改革取得明显进展。基本建立适应数字经济发展的开放合作机制、创新创业体制机制、科技管理体制、良好营商环境、标准化规范、法规体系,发展环境明显趋好。

　　——基础设施、安全、投融资、人才、财税、土地等数字经济发展保障支撑能力明显提升。信息基础水平和供给能力明显提升,网络安全保障能力明显提高,投融资、人才、财税等配套政策对吸引和促进各类生产要素充分自由流动的作用进一步增强,发展的基础条件和保障水平处于西部前列。

　　二、推动数字经济重点型态加快发展

　　(三)推动资源型数字经济加快发展。

　　1.发展数据采集、集聚与存储等服务。以“云上贵州”系统平台及互联网、移动互联网、社交网络等商业化平台为重点,发展专业化数据采集服务。加快推进贵州射电天文台及FAST等平台建设,大力发展天文数据采集与存储服务。加强与国内外知名企业在超算、云计算、雾计算等方面的技术合作和研发攻关,加快推进全球数据中心、国家智慧城市等项目建设,发展平台即服务、软件即服务、数据即服务等云计算服务,发展数据存储、应用承载、容灾备份等数据服务。2020年,数据存储业务收入达到20亿元以上。

　　2.发展数据加工处理分析服务。加快推进大数据压缩处理服务及设备生产基地等项目建设,加快建设贵阳、贵安等大数据清洗加工基地,发展档案数字化、网络数据清洗、工业自动化数据整理等服务。重点建设贵阳大数据产业园、贵安电子信息产业园及数字经济产业园、遵义大数据软件园等园区,加快发展大数据分析、建模与可视化等服务。2020年,基本建成中国南方重要的数据加工及分析产业集群。

　　3.发展数据交易等服务。加快建设贵阳大数据交易所,发展数据资产评估、大数据征信、大数据融资等相关配套服务。2020年,大数据交易额累计突破200亿元,基本建成国家重要的数据交换交易中心。

　　(四)推动技术型数字经济加快发展。

　　1.发展智能终端、芯片和新型电子材料等数字化研发制造业。打造贵阳、遵义、贵安新区等智能终端产业集聚区,大力发展智能手机、平板电脑、教育多媒体机、医疗健康电子设备、智能可穿戴设备等智能终端产品,以及液晶面板及模组、智能器件、新型电子材料等。加快推进智能终端、服务器生产线等项目建设,推动集成电路芯片设计研发和电子材料产业发展。2020年,建成8个以上智能终端产业园区,本地化配套率达到40%以上。

　　2.发展呼叫、信息外包等数字经济服务业态。推进贵阳、遵义和毕节等地呼叫中心与服务外包产业基地建设,重点建设大数据及信息服务外包产业基地、大数据云服务外包基地等项目,打造“黔中声谷”知名品牌。加强与“一带一路”沿线国家合作,拓展针对国际市场的离岸信息技术服务外包、离岸软件外包、离岸数据外包等服务。

　　3.发展人工智能和智能制造业等数字经济前沿产品和技术。依托贵州·中国南方数据中心示范基地,建设人工智能产业创客空间、人工智能产业园。以贵阳高新区、遵义新蒲新区、贵安新区等为重点,支持工业机器人本体、控制器、伺服电机等关键零部件产品的研发和应用。在贵阳、贵安新区等地率先建设一批省级互联网工业设计中心。在贵州航天高新技术产业园、贵阳高新·白云发动机产业园、安顺民用航空产业国家高技术产业基地发展北斗卫星导航、无人机等。2020年,人工智能业务收入达到5亿元以上,初步形成具有一定竞争力和影响力的智能制造产业集群。

　　(五)推动融合型数字经济加快发展。

　　1.发展数字农业。建好贵州农业云、农经云,加快发展农业农村互联网、农业物联网、农业信息化服务等。2020年,基本建成数字农业产业服务体系。

　　2.推动能源、建材等行业数字化升级。提升发展贵阳高新区新材料新能源产业基地、贵安新区新材料产业基地,推进石墨烯新材料、核电用特种镍基高温合金材料锻件、大尺寸高纯金属靶材用电子级高纯钛等项目数字化水平。推进智能工厂和数字化车间、数字化矿井等建设,加快工业机器人的规模化应用。2020年,传统行业企业生产方式精细化、柔性化、智能化水平显著提升,重点行业数字化研发设计工具普及率达到74%、关键工序数控化率达到58%。

　　(六)推动服务型数字经济加快发展。

　　1.发展智慧旅游、智慧健康等服务业。建设贵州“智慧旅游云”,加快国际旅游数据中心等项目建设,在贵阳、遵义、安顺等地率先建设一批智慧景区、智慧酒店、智慧旅行社。加快数字公园、VR主题公园等智慧公园的建设。大力发展“大数据+医疗”、“大数据+健康管理”等,推进智慧医疗云平台、医药健康科技园、互联网医院等项目建设。2020年,完成30个4A级景区智能化升级,基本建成覆盖城乡居民的智慧医疗健康服务体系。

　　2.发展数字金融、智慧物流等生产性服务业。推进“互联网+”普惠金融发展,加快贵阳互联网金融产业基地建设,争取国家批准贵安新区开展绿色金融试点,推动金融云、信用云建设,发展网络金融、网络股权众筹、互联网信贷等新金融产品。以物流云建设为纽带,加快推进信息化物流园区、智能公路港、智慧物流园等项目建设,整合现有园区、企业信息化平台,实现货源、车辆、仓储的数字化管理和网络化交易。2020年,初步建成西部地区重要的数字金融中心和数字化区域物流枢纽。

　　3.发展电子商务、数字共享、文化创意等新型服务业态。深化与阿里巴巴等知名电商的战略合作,加快建设电商产业园、农村电子商务综合服务体系等项目。重点支持贵农网等综合电商平台建设,积极发展社区电商、行业电商、跨境电商等。大力发展分享经济。加快贵州文化出版产业园、中国文化(出版广电)大数据产业、黔东南原生态民族文化创意产业园等文化创意产业园建设,发展数字媒体、数字出版、VR游戏等。2020年,电子商务交易额突破3600亿元。

　　三、培育数字经济新型市场主体

　　(七)引进一批数字经济市场主体。依托贵州大数据综合试验区、“1+7”国家级开放创新平台、数博会等招商引资引智平台,大力吸引地区总部、外资研发中心、生产性服务业及贸易总部集聚,重点在机器人、芯片制造、人工智能等领域引进一批优强企业。配套引进一批中小型数据应用商、数据生产者,吸引带动一批大数据关联企业到贵州投资发展大数据“加工厂”。

　　(八)打造一批数字经济的领军型市场主体。推动一批品牌影响力大、创新能力强、发展潜力大、带动性强的数字经济平台企业,加快培育发展成具有全国竞争力的大型企业、企业集团和行业龙头企业。鼓励企业或行业协会牵头成立产业战略联盟。

　　(九)培育一批数字经济中小微企业。推动中小微企业向“专精特新”发展,培育一批以数字经济企业为骨干的中小微创新型企业集群。

　　(十)发展一批服务于数字经济企业的中介机构。培育一批知名科技服务中介机构,大力发展技术转移、创业孵化、知识产权等科技服务。加快推进技术评估、知识产权服务、第三方检验检测认证等机构改革。

　　四、深化重点领域改革

　　(十一)加快建立完善数字经济开放合作体制机制。

　　1.深入推进数据资源共享与开放。加快建成数字“一带一路”信息港(贵州)。加快建设“云上贵州”数据共享交换平台。制定实施公共数据开放计划。引导企业、行业协会、科研机构等依法采集并开放数据,鼓励企业、社会组织和个人运用数据进行商业模式创新。

　　2.推进数字经济投资贸易便利化。复制推广上海自由贸易试验区经验,完善外商投资市场准入制度,推进“一口受理、并联审批”改革,不断简化外资企业准入程序。

　　3.推动数字经济区域合作。探索推动数字经济园区共建、共管等模式。深化在远程医疗、远程教育等公共服务领域的合作,加快对口帮扶数字化开放进程。

　　(十二)推动数字经济创新创业。

　　1.建立完善数字经济创业创新政策扶持体系。建立省数字经济创业投资基金,支持大数据、互联网、云计算等领域技术创新。制定《贵州省数字经济创业指导目录》,支持创业指导、融资服务、成果转化等社会机构联合建立数字经济创业服务联盟,鼓励各地以创业服务券等形式支持大众创业。

　　2.打造数字经济创业创新孵化平台。建设一批网络服务、互联网融资中介服务、公共科技创新等平台。支持各地设立数字经济创业中心,推动在黔高校设立数字经济创业苗圃。2020年,建成100个数字经济孵化器和数字经济创新空间。

　　3.打造数字经济创新创业集聚发展平台。创建贵安数字经济创新示范区,打造贵阳数字经济示范城市、遵义数字端产品制造集聚区,建设贵安-贵阳-遵义数字经济核心引领带和一批省级数字经济示范基地(园区),建成10个数字经济示范区(县)、20个数字经济特色小镇(园区)。

　　(十三)深化科技管理体制改革。

　　1.推动政府职能从研发管理向创新服务转变。建立健全决策、执行、评价相对分开和互相监督的运行机制。健全省级科技项目管理平台,探索建立由第三方机构管理科技计划和项目资金的体制,完善科技计划管理体制。

　　2.完善科技创新激励机制。加快科技成果使用、处置和收益管理改革。建立主要由市场决定技术创新项目和经费分配、成果评价和传导扩散的新机制。完善科技人员股权和分红激励办法。

　　(十四)营造良好营商环境。

　　1.减少审批环节。最大限度取消企业资质类、项目类等审批审查事项,消除行政审批中部门互为前置的认可程序和条件,减少政府对数字经济企业创新创业活动的干预。

　　2.深化商事制度等改革。完善数字经济企业行业归类规则和经营范围的管理方式。调整现有行业管理制度中不适应数字经济特点的市场准入要求,改进对与互联网融合的金融、医疗保健、教育培训等企业的监管。

　　(十五)推动数字经济法制化标准化改革。

　　1.加快建立各类数字经济标准。制订政府数据采集、开放、共享、分类、质量、安全管理等关键共性标准,建立政府数据标准体系。开展国家大数据交易等数据资源流通标准研制与试点示范,研制企业间数据共享、数据交易、大数据确权等标准。

　　2.适时开展标准评估验证。推进重点行业和领域开展标准验证和评估试点,在贵阳等地开展标准验证评估工作。

　　(十六)开展数字经济立法探索。研究推动数据权益保护、个人信息和隐私保护、数据安全等地方立法工作。开展《贵州省大数据发展应用促进条例》的评估修订。

　　五、完善数字经济发展支撑保障体系

　　(十七)强化数字经济基础保障。

　　1.强化出省通道建设。加快建设国家骨干直联点,拓宽互联网出省带宽,2017年出省带宽能力超过5500Gbps,2020年超过10000Gbps。

　　2.稳步提升贵州光纤网络发展地位和水平。巩固贵州在中国电信网络的骨干节点地位,加快成为中国移动、中国联通网络骨干节点和广电网络核心节点。推广应用IPv6。

　　3.加快建设城乡高速宽带网络。加快建设“光网贵州”、“满格贵州”,推进“百兆光网城市”、“宽带乡村”建设,推进有线广播电视网络双向化改造。到2020年,家庭固定宽带普及率达70%,城市家庭宽带接入能力达100Mbps,行政村100%通宽带,农村家庭宽带接入能力超过20Mbps。全省信息化发展指数达到85。

　　(十八)强化数字经济安全保障。

　　1.建立网络安全态势动态感知体系。建设省级网络安全信息大数据中心,建立网络安全态势动态感知云平台,建立网络安全信息共享机制。

　　2.建立关键信息基础设施安全保障体系。建立金融、能源、电力、通信、交通等领域关键信息基础设施产品与服务安全审查制度,督促各地各部门及时开展风险消减工作,提升对关键信息基础设施网络安全突发事件的应急处置能力。

　　3.增强网络信息安全防护能力。落实网络安全责任制,制定详尽的网络基础设施防护和应急方案,加强重要网络基础设施的安全防护。建立网络信息安全防线。

　　(十九)强化数字经济投融资保障。

　　1.推动社会资本向数字经济领域加快集中。建立数字经济项目库,及时发布并推进数字经济工程包建设,向在黔商会、大企业大集团加强推广,吸引社会资本向数字经济优质项目加大投入。鼓励天使投资、风险投资、创业投资、私募基金等投资机构支持数字经济企业发展。

　　2.加快建立适应数字经济发展的融资模式。引导金融机构探索开展以知识产权为抵押物的信贷业务。支持外资创业投资、股权投资机构积极探索项目管理新模式。培育有条件的数字经济企业上市融资,鼓励中小数字经济企业在“新三板”等股权交易中心挂牌融资。支持符合条件的数字经济企业通过发行企业债券、公司债券、非金融企业债务融资工具等方式扩大融资。鼓励各县(市、区)政府、产业主管部门、园区管理机构给予数字经济领域创新型企业融资一定额度的贷款贴息、评估补助、风险补助及其他形式的金融服务。

　　(二十)强化数字经济人才保障。

　　1.加大数字经济人才引进力度。将数字经济高层次人才纳入全省急需紧缺高层次人才引进计划,对引进的高层次人才,可享受国家和我省人才引进相关规定的优惠政策。

　　2.加大数字经济人才培育力度。鼓励企业与国内外知名高校院所开展合作,重点培养数字经济领域紧缺技能人才。对高校、科研院所等专业技术人员经同意离岗的,可在3年内保留人事关系。积极支持和指导数字经济高层次人才入选我省百人领军人才、千人创新创业人才,优先向国家推荐“国家百千万人才工程”人选。

　　3.加大数字经济人才培训力度。鼓励有条件的职业院校、社会培训机构和数字经济企业开展网络创业培训。将数字经济相关职业(工种)纳入就业技能培训和高技能人才培训补贴范围。对参加网络创业培训、职业培训和职业技能鉴定的人员,以及组织职工培训的数字经济企业,可按规定享受创业培训、职业培训补贴和职业技能鉴定补贴政策。

　　(二十一)强化数字经济财税保障。

　　1.加大财政支持力度。省大数据产业发展基金重点支持数字经济创新发展,省级工业与信息化专项资金、应用技术研究与开发资金、大数据发展专项资金等项目及资金重点向数字经济倾斜。对在黔投建投资总额1亿元以上、符合本意见发展导向的数字经济产业项目,在合同约定期限内竣工投产的,按项目累计技术设备投资额(以完税发票为依据),由项目税收所在地政府给予补助,最高补助额不超过300万元。

　　2.加大税收扶持力度。对创业投资企业和有限合伙制创业投资企业的法人合伙人从事国家鼓励的创业投资,符合条件的可按投资额的一定比例抵扣应纳税所得额。优先支持符合条件的数字经济企业认定为高新技术企业,对被认定为高新技术企业的,可享受15%的企业所得税优惠税率。对数字经济企业新购进的专门用于研发的仪器、设备,单位价值不超过100万元的,可按规定在税前一次性扣除;单位价值超过100万元的,可缩短折旧年限或采取加速折旧的办法。对处在起步阶段、规模不大但发展前途广阔、有利于大众创业万众创新的数字经济形态,按照国家有关税收激励政策,依法可享受企业所得税、增值税等税收优惠政策。对数字经济企业开发新技术、新产品、新工艺发生的研究开发费用可按规定在计算应纳税所得额时加计扣除。

　　(二十二)强化数字经济土地保障。

　　1.加大用地保障力度。依法保障数字经济新产业、新业态用地供应。对新产业、新业态发展快、用地集约且需求大的地区,可适度增加年度新增建设用地指标。对符合土地利用总体规划和城乡规划的数字经济产业项目,优先保障用地。对数字经济企业用地,在符合产业方向,明确产业用地类型的前提下,可采用挂牌方式出让,提高土地资源开发效能。

　　2.依法依规降低土地成本。对列入省重点项目计划的新建数字经济项目取得国有建设用地使用权,可以分期缴纳土地出让金,签订土地出让合同后1个月内缴纳出让价款的50%,余款在1年内缴清。对纳入省数字经济产业规划且用地集约的数字经济产业重点项目,在确定土地出让底价时可按不低于所在地土地等别相对应工业用地出让最低价标准的70%执行,但不得低于实际土地取得成本、前期开发成本和按规定应收取的相关费用之和。对新落户的数字经济企业,自投产次年起5年内,年度纳税(不含土地使用税)亩均3万元以上的,由地方政府给予一定奖励。对数字经济企业现有工业用地,在符合规划、不改变用途的前提下,提高土地利用率的,不再增收土地价款。鼓励实行长期租赁、先租后让、租让结合的工业用地供应方式,加快办理产业园区用地手续。数字经济产业集聚区和数字经济企业符合住房保障条件的员工可纳入当地住房保障政策范围。

　　六、共享数字经济红利

　　(二十三)推进数字化精准扶贫。加快建成精准扶贫云,实现扶贫精准管理。搭建扶贫信息发布与互动救助平台,实现扶贫济困“点对点”供需见面。结合推进“网络扶贫”,发挥数字化在产业扶贫特别是农发行长期低息专项贷款中的重要作用,为相关机构拓展贷款信息来源提供决策依据。加快建设一批农村电子商务进农村综合示范县、示范镇、示范村和示范企业,到2020年,打造6000个贫困村电商扶贫试点。

　　(二十四)深入推进“互联网+政务服务”。全面梳理编制直接面向公众提供的政务服务事项目录。全程推行标准审批,完善行政处罚服务平台。建设贵州政务服务微信公众号和云上贵州APP平台,推进服务事项网上办理。积极推行“多图联审”“多评合一”“多证合影”“联合验收”服务模式,依托贵州省网上办事大厅数据分析平台,探索智慧审批。加快推进人口信息、法人单位信息、自然资源信息、公共信息信用库等基础信息资源与行政审批服务系统的互联互通,建设全省统一的涉及行政审批的中介服务专家平台。

　　(二十五)推动公共服务数字化升级。大力推动教育管理、教学和科研的数字化开发与应用,推动在线课堂教学、智慧校园、标准化考试系统及教育资源管理与服务系统的融合发展。推动医疗健康数字化应用,完善医疗健康云。以电子政务、数据铁笼、新型智慧城市建设为抓手,实现跨层级、跨地区、跨行业、跨系统、跨部门、跨业务的协同管理和服务。推动社会保障数字化应用,加快建设智慧人社云。推动社区服务数字化应用,建设“社区和云”社区综合服务平台。

　　七、保障措施

　　(二十六)加强组织领导。省大数据发展领导小组要进一步加强对数字经济发展的指导。各部门要从自身职能职责出发,认真贯彻落实省委、省政府决策部署,全力以赴推动数字经济加快发展。各市州要加强对数字经济发展的统筹协调,解决好数字经济发展推进中的重大问题。

　　(二十七)加强监测分析。开展数字经济统计,建立数字经济统计调查和监测分析制度,强化数字经济数据搜集、处理、发布和共享工作,建立数据沟通和分享机制。对数字经济发展进程中出现的新问题新情况进行密切跟踪,加快构建数字经济发展全貌和动态变化的指标体系,准确反映贵州数字经济发展实际情况。

　　(二十八)加强督查评估。建立和完善督查联动机制,对各有关部门和单位落实本意见的情况进行督查,确保意见确定的各项任务有力有序有效推进。适时组织开展专项督查和第三方评估,对未完成任务的地区、部门和单位严肃问责。

(二十九)加强舆论宣传。坚持政府主导、社会参与的原则,综合利用报纸、广播、电视、网站等各种传媒,通过开辟专题专栏等途径,广泛深入宣传发展数字经济的重要意义和取得的成就。加强政策解读和各类示范试点的宣传推广,积极构建有利于数字经济发展的良好社会氛围。

（27）内蒙古自治区人民政府关于加快推进数字经济发展若干政策的通知

（内政发〔2022〕33号）

各盟行政公署、市人民政府，自治区各委、办、厅、局，各大企业、事业单位：

　　为深入贯彻党中央、国务院关于加快数字经济发展的决策部署及自治区党委和政府工作要求，全面落实《内蒙古自治区“十四五”数字经济发展规划》《内蒙古自治区推进数字产业化产业数字化发展行动方案（2021-2023年）》，进一步激发全区数字经济发展活力，促进自治区高质量发展，现就加快推进数字经济发展若干政策通知如下。

　　一、加快5G网络建设

　　（一）自治区通信管理部门以国土空间规划为基础，会同有关部门制定全区5G等通信基础设施空间布局专项规划，除法律、法规另有规定外，公共交通枢纽、校园、医院、政府机关及企事业单位、公园绿地等公共设施和场所无偿向5G基站开放，5G基站用地需求纳入年度用地计划，建筑物规划预留5G微站、室内分布系统等设施空间。（内蒙古通信管理局，自治区自然资源厅、工业和信息化厅、住房城乡建设厅负责）

　　（二）基础电信、铁塔和广电运营企业使用高速公路等路网管孔，租赁费用原则上按不高于4000元/管孔/公里/年结算价格签订租赁合同，同时可考虑市场价格因素，与高速公路经营单位协商租赁费，上下浮动价格，各租赁企业不得转租。（自治区国资委、交通运输厅负责）

　　（三）对5G基站及配套机房、核心枢纽机房等用电价格给予符合国家政策规定的电价扶持。（自治区工业和信息化厅、发展改革委，内蒙古通信管理局负责）

　　二、支持算力网络国家枢纽节点建设

　　（四）优化数据中心建设布局，新建大型、超大型数据中心原则上布局在全国一体化算力网络国家（内蒙古）枢纽节点和林格尔数据中心集群（和林格尔新区、集宁大数据产业园）内，积极争取国家对和林格尔数据中心集群内符合条件的数据中心项目给予支持。（自治区发展改革委、党委网信办、工业和信息化厅负责）

　　（五）对和林格尔数据中心集群内的数据中心建设项目给予符合国家规定的电价扶持。（自治区发展改革委负责）

　　三、支持壮大电子信息制造业和软件信息服务业

　　（六）支持电子信息制造业发展，服务器及数据中心相关设备、半导体、通信设备、智能终端、消费电子产品、传感器、光电、显示、电子专用装备及关键电子材料、零部件、元器件等列入自治区数字经济重点项目名单的项目，由自治区数字经济发展专项资金给予支持。（自治区发展改革委、财政厅、工业和信息化厅负责）

　　（七）对符合财政部等四部委《关于软件和集成电路产业企业所得税优惠政策有关问题的通知》（财税〔2016〕49号）相关条件的企业，按照规定享受企业所得税优惠政策。（内蒙古税务局负责）

　　（八）按照首版次软件取得知识产权或证书规定期限内截至申请时的实际销售总额的20%给予补助，单个企业补助金额最高不超过300万元。对企业新开发的工业软件（工业APP），按照研发该产品实际投入金额的20%给予补助，单个企业补助金额最高不超过300万元。上述支持资金由自治区重点产业发展专项资金安排。（自治区工业和信息化厅、财政厅负责）

　　四、支持制造业数字化、网络化、智能化改造

　　（九）支持智能工厂、数字化车间项目建设。对智能工厂、数字化车间项目，按照实际完成设备投资额的20%、单个项目最高不超过500万元给予补助。支持高危、高强度生产环节“机器换人”，按照工业机器人购置费用的20%、单个项目最高不超过500万元给予补助。上述支持资金由自治区重点产业发展专项资金安排。（自治区工业和信息化厅、财政厅负责）

　　（十）对5G等新一代信息技术在工业领域的典型应用场景项目，按照实际完成投资额的20%、单个项目最高不超过500万元给予奖补。通过国家“两化融合”管理体系标准贯标评定的企业，达到5A、4A、3A、2A、A的分别给予300万元、200万元、100万元、50万元、30万元一次性奖励。对新创建的区域级、行业级、企业级工业互联网平台，按照实际完成投资额的20%、单个项目最高不超过500万元、400万元、300万元给予奖补。对新建工业互联网标识解析二级节点且与国家顶级节点实现互联互通的企业，给予500万元一次性补助。对开展工业互联网标识解析创新应用的企业，依据应用效果给予补助，单个企业最高不超过300万元。上述支持资金由自治区重点产业发展专项资金安排。（自治区工业和信息化厅、财政厅负责）

　　（十一）建设涵盖企业运行、项目建设、能耗管控、污染监测等一体化、“全景式”园区管控平台，由自治区工业园区发展专项资金对单个园区给予最高不超过2000万元一次性奖补。（自治区工业和信息化厅、财政厅负责）

　　五、支持农牧业数字化转型

　　（十二）积极推进数字乡村建设，推动内蒙古农牧大数据平台建设，鼓励开展高分北斗、物联网、人工智能、大数据、区块链等数字技术在农牧业生产、流通等各环节融合应用。鼓励市场主体利用互联网嫁接特色产业，发展农村电商、创意农业、观光农业、认养农业、分享农业等新业态，满足城乡居民消费升级需求。上述领域列入自治区数字经济重点项目名单的项目，由自治区数字经济发展专项资金给予支持。（自治区党委军民融合办、党委网信办、农牧厅、发展改革委、工业和信息化厅、财政厅、商务厅负责）

　　六、支持服务业数字化转型

　　（十三）鼓励企业利用5G、高分北斗、大数据、人工智能、物联网、云计算等新兴技术，在教育、医疗康养、文化旅游、交通设施、电子商务、智慧物流、文化创意、金融消费等服务领域开展数字技术融合应用，促进相关服务业及交通等基础设施数字化、智能化发展。上述领域列入自治区数字经济重点项目名单的项目，由自治区数字经济发展专项资金给予支持。（自治区教育厅、卫生健康委、文化和旅游厅、交通运输厅、商务厅、地方金融监管局、财政厅、发展改革委、工业和信息化厅负责）

　　七、支持政府数字化技术应用

　　（十四）推动各地区各部门在经济调节、生态保护、城市管理、公共服务、市场监管、政务运行等领域开展数字化应用创新，自治区本级政务信息化项目由自治区数字经济发展专项资金给予保障。（自治区发展改革委、财政厅负责）

　　八、支持数字科技创新

　　（十五）支持自治区与国家部委共建数字技术创新平台，对各类创新平台实行动态管理，有进有出，择优支持。国家技术创新中心批准建设后，连续5年由自治区财政科技专项资金每年给予不低于3000万元的资金支持，国家级重点实验室、工程研究中心批准建设后，按照认定渠道，分别由自治区财政科技专项资金、自治区数字经济发展专项资金每年给予不低于1000万元的资金支持。（自治区科技厅、发展改革委、财政厅负责）

　　（十六）引导企业、高校、科研院所等创新主体加大研发投入力度，鼓励对数字科技创新成果进行市场化转化。对新获批的数字经济领域国家级和自治区级科技成果转移转化示范区，由自治区财政科技专项资金分别给予一次性最高500万元、200万元的资金支持。对自治区科技厅组织的绩效评价优秀的科技成果转移转化示范基地、专业化技术研发与中试公共服务平台，由自治区财政科技专项资金给予后补助支持。（自治区科技厅、财政厅负责）

　　（十七）推动规模以上数字经济企业加强研发机构建设，建立研发准备金制度，规范研发费用核算。按照企业上年度研发投入强度及增量增幅，由自治区财政科技专项资金给予补助，单个企业每年最高补助500万元用于技术研发。（自治区科技厅、财政厅负责）

　　（十八）鼓励企业或机构自主投资建设数字化转型促进中心，为中小微企业提供转型咨询、解决方案、技术培训、资源对接等服务，经评估认定为优秀的，由自治区数字发展专项资金给予不超过100万元的后补助支持。（自治区发展改革委、财政厅负责）

　　九、加强人才队伍建设

　　（十九）加大院士后备人选和领军人才培养力度，对有潜力参选院士的高层次数字经济领军人才、行业领军人才，经评审遴选符合条件的，由自治区“草原英才”工程专项资金分别给予一次性500万元、300万元专项资助用于开展科研活动。加快引进数字经济重点领域创新型领军人才和创新团队，鼓励其申报自治区级以上重大人才工程、重大科技专项、重大研究课题、重大创新平台。（自治区党委组织部、科技厅、人力资源社会保障厅、财政厅负责）

　　十、支持数字经济领域小规模纳税人

　　（二十）支持短视频平台、微创新、微应用、微产品、微电影等新个体经济发展，对自治区上述数字经济领域的自然人，按照增值税优惠政策享受税收减免政策。（内蒙古税务局负责）

　　（二十一）对信息技术企业中增值税小规模纳税人按照50%的幅度减征资源税（不含水资源税）、城市建设维护费、房产税、城镇土地使用税、印花税（不含证券交易印花税）、耕地占用税、教育费附加、地方教育附加。（内蒙古税务局负责）

　　十一、拓宽企业融资渠道

　　（二十二）鼓励数字经济企业上市挂牌，对自治区数字经济企业，按照《内蒙古自治区人民政府办公厅关于印发内蒙古自治区鼓励企业上市挂牌奖补办法的通知》（内政办发〔2021〕38号）、《内蒙古自治区人民政府办公厅关于修改内政办发〔2021〕38号文件相关内容的通知》（内政办字〔2021〕92号）相关要求给予企业上市奖补。（自治区地方金融监管局、财政厅负责）

　　（二十三）发挥自治区重点产业发展引导基金的作用，积极引导国内外电子信息、软件、互联网等行业龙头企业在自治区落地项目。（自治区财政厅负责）

　　（二十四）对自治区数字经济领域具有引领性、牵引性的重大项目采取一事一议的方式重点支持。（自治区发展改革委负责）

　　上述相关支持政策依据负责部门的实施细则具体执行，各资金及政策主管部门要做好资金、政策及部门职责分工的衔接，明确具体申请条件、审批程序等实施细则并向社会公布，便于相关主体了解申报流程和具体要求，确保本政策相关部署落地见效。

2022年12月27日

（28）山西省财政厅、山西省工业和信息化厅关于印发《省级数字经济发展专项资金管理办法》的通知 （晋财建〔2022〕65号）

各市财政局、工业和信息化局、大数据应用局：

　　为深入贯彻落实党中央、国务院关于数字经济的战略部署，进一步规范数字经济发展专项资金的管理和使用，提高资金使用效益，省财政厅、省工业和信息化厅对《省级数字经济发展专项资金管理办法》进行了修订。现予印发，请遵照执行。

山西省财政厅

山西省工业和信息化厅

2022年6月16日

**省级数字经济发展专项资金管理办法**

　　第一条　为贯彻落实党中央、国务院数字经济战略部署和《山西省人民政府关于印发山西省加快推进数字经济发展的实施意见和若干政策的通知》（晋政发〔2021〕25号，以下简称《数字经济实施意见和若干政策》）、《山西省人民政府关于印发山西省新时期促进集成电路产业和软件产业高质量发展若干政策的通知》（晋政发〔2020〕20号，以下简称《集成电路和软件产业若干政策》）和《山西省人民政府关于印发山西省加快5G产业发展的实施意见和若干措施的通知》（晋政发〔2019〕21号，以下简称《5G实施意见和若干措施》），规范省级数字经济发展专项资金使用管理，提高资金使用效益，根据《中华人民共和国预算法》和《省级财政专项资金管理办法》（晋财省直预〔2020〕38号）等文件要求及预算管理相关规定，制定本办法。

　　第二条　本办法所称数字经济发展专项资金（以下简称“专项资金”），是指由省级财政预算安排，专项支持数字经济发展的资金。

　　第三条　专项资金由省财政厅、省工信厅按照职能分工，各司其职、各负其责、共同管理。

　　省财政厅主要负责专项资金预算管理，根据省工信厅提出的资金分配建议，确定资金分配方案；审核确定项目预算绩效目标，指导、督促有关部门和单位依据绩效目标开展绩效监控、绩效评价、绩效评价结果应用等相关绩效管理工作。

　　省工信厅主要负责专项资金的项目管理，提前做好项目储备，牵头印发专项资金年度工作指南，组织项目申报和遴选，审核报送的材料和数据，提出资金分配建议并负责项目实施管理；负责预算绩效管理，审核项目绩效目标并提交省财政厅。

　　第四条　专项资金使用管理遵循科学规范、公平合理、注重绩效、强化监督的原则。

　　第五条　专项资金支持对象为独立法人单位或中央驻晋企业在山西省境内实施符合本办法第六条规定的项目。

　　第六条　专项资金支持范围：

　　（一）《数字经济实施意见和若干政策》《5G实施意见和若干措施》《集成电路和软件产业若干政策》确定的相关支持项目；

　　（二）省委、省政府确定支持的重点项目；

　　（三）数字经济领域的政策研究、宣传推介、行业监测、交流培训、安全保障等服务体系项目；

　　（四）“信用中国（山西）”网站确定的失信主体所申报的项目不予支持；

　　（五）已享受省级财政政策支持的项目不得重复支持，重大项目可以根据项目实施进度分年度给予支持。

　　第七条　专项资金主要采取补助、贷款贴息、奖励等支持方式。

　　第八条　申报审核流程：

　　（一）省工信厅根据全省数字经济发展工作重点，制定年度申报指南，向社会公开发布并报驻省工信厅纪检监察组备案。

　　（二）市级工信部门、大数据行业主管部门和山西转型综改示范区管委会按照项目属地原则，组织开展项目申报和真实性、政策符合性、可行性审核，并将通过审核的项目汇总后推荐报送至省工信厅。

　　（三）省工信厅组织专家或委托行业组织、评审机构等第三方，对报送项目进行评审，提出项目评审意见。

　　（四）省工信厅依据第三方评审意见，提出支持项目名单及资金分配建议，公示无异议后报省财政厅。

　　（五）省财政厅根据工信厅提出的资金分配建议，确定资金分配方案，按国库支付要求及时拨付资金。

　　（六）省工信厅要提前做好项目储备，加快报送项目资金分配计划。转移支付项目原则上应于每年11月20日前提前确定下一年度项目资金分配计划（提前下达市县规模不得低于上年预算执行数的70%），资金于11月底前提前下达。未提前下达的转移支付项目，应在省人代会批准预算后45日内报送资金分配计划，60日内全部下达。省本级项目资金在编制下年预算时应明确具体项目，并列已分配，未细化部分的资金分配计划应于每年6月20日前全部报送完毕，资金于6月底前全部下达完毕。

　　第九条　项目申报单位须对申报资料的真实性、资金使用及绩效负责；市级工信部门、大数据行业主管部门和山西转型综改示范区管委会对推荐报送项目的真实性、政策符合性、可行性负责；评审方对出具的评审意见负责。

　　第十条　省财政厅、省工信厅应对专项资金实施预算绩效管理，对专项资金设立客观的绩效目标并组织开展绩效评价，同时加强绩效评价结果运用。

　　第十一条　除涉及保密要求不予公开外，省工信厅应在门户网站或通过其他媒体向社会公开专项资金的申报指南、分配程序和方式、分配结果等相关信息。

　　第十二条　资金使用单位要严格遵守国家有关财务会计制度，自觉接受审计、监察、财政等有关部门的监督检查。

　　第十三条　对截留、挪用、挤占和弄虚作假等骗取专项资金的违纪违法行为，依照相关法律、法规和规定进行处理。

　　第十四条　本办法由省财政厅、省工信厅负责解释。

第十五条　本办法自公布之日起施行，有效期至2025年12月31日。《省级数字经济发展专项资金管理办法》（晋财建一〔2019〕221号）同时废止。

（29）黑龙江省人民政府办公厅关于印发黑龙江省支持数字经济加快发展若干政策措施的通知 黑政办规〔2022〕12号

各市（地）人民政府（行署），省政府各直属单位：

　　《黑龙江省支持数字经济加快发展若干政策措施》已经省政府同意，现印发给你们，请认真贯彻执行。

黑龙江省人民政府办公厅

2022年3月22日

**黑龙江省支持数字经济加快发展若干政策措施**

　　为推进《黑龙江省“十四五"数字经济发展规划》实施，加快数字产业化、产业数字化进程，做强做优做大数字经济，打造数字经济发展新优势，制定如下政策措施。

　　一、培育引进市场主体

　　1.支持引进龙头企业。围绕集成电路、高清晰新型显示、电子产品制造、智能可穿戴、数字通信、机器人、大数据、软件、信息安全、数字创意设计等重点发展产业，加快引进龙头企业。对引进企业总投资在2亿—5亿元和5亿元以上的项目，按照同期市场报价利率，给予新增贷款5年期限贴息，每年单个项目贴息额度分别不超过500万元和1000万元。〔责任单位：省商务厅、省财政厅，各市（地）人民政府（行署）〕

　　2.支持骨干企业做大做优。对营业收入首次超过1亿元、5亿元、10亿元的我省数字制造企业，分别给予一次性100万元、300万元、500万元奖励。对全国电子信息百强、软件百强、互联网百强企业在我省落户且投资超过2亿元的，经认定给予一次性200万元奖励。对我省企业首次进入全国电子信息百强、软件百强、互联网百强，经过认定且在我省入统纳税的，给予一次性200万元奖励。对我省企业成长为独角兽、瞪羚企业的，按贡献率给予特殊奖励。〔责任单位：省发改委、省工信厅、省科技厅、省财政厅，各市（地）人民政府（行署）〕

　　3.支持发展总部经济。对数字经济领域世界和国内500强、龙头企业及境内外上市企业来我省设立企业（集团）总部、区域总部和功能性总部的，五年内分别按其主营业务收入的1%、0.8%、0.5%给予奖励，每年奖励额最高1000万元、500万元、300万元。〔责任单位：省发改委、省商务厅、省财政厅，各市（地）人民政府（行署）〕

　　4.支持平台招商。鼓励建设数字经济领域社会性质的招商平台，对每年招商签约落地项目达10家（单个设计类企业研发设计人员不低于20人、单个制造类和平台类项目实际固定资产投资不低于5000万元）的平台，给予100万元奖励，每超过1家增加10万元，每个平台最高300万元。〔责任单位：省发改委、省商务厅、省财政厅，各市（地）人民政府（行署）〕

　　二、延伸壮大数字产业链

　　5.支持全产业链招商。鼓励数字经济领域企业开展产业链和供应链招商，对于引进固定资产投资超亿元项目的，给予实施招商企业固定资产投资额1%的奖励，最高奖励不超过2000万。存量企业围绕产业链和供应链扩大投资，享受同等招商引资优惠政策。〔责任单位：省发改委、省工信厅、省商务厅、省财政厅，各市（地）人民政府（行署）〕

　　6.支持数字经济供应链创新发展。支持头部企业在我省布局建设智能仓储、智能配送项目，打造未来物流网络，在协调干线运输能力、航空包机补贴等方面给予支持。对数字经济领域企业原材料、零配件和产成品运输实施绿色通道，给予降低物流成本的政策支持。〔责任单位：省发改委、省商务厅、省交通运输厅、省财政厅，各市（地）人民政府（行署）〕

　　7.支持产业链创新示范。围绕发展数字产业开展产品示范评选活动，对技术创新水平较高、市场竞争力突出、在我省生产且年销售收入不低于1000万元的智能硬件和软件产品，每年评选认定不超过20个示范项目，每个给予一次性50万元奖励。〔责任单位：省工信厅、省发改委、省财政厅〕

　　8.支持数字产业集聚建设。整合相关资源，支持建设数字经济产业园区，推动数字经济项目向产业园区集聚，培育壮大优势特色数字产业集群。对进入数字经济园区且主营业务收入超过10亿元的数字经济企业，按其对地方经济贡献新增部分的50%，连续五年给予奖励。〔责任单位：省商务厅、省发改委、省财政厅〕

　　9.支持数字经济园区建设。数字经济企业利用存量土地新建工业厂房，在符合规划、不改变用途的前提下不再增收土地价款。对利用园区建设的标准化厂房进行生产的企业，前三年供企业免费使用，之后可租可购，对先租后购的，租金可抵后期购房款项。对数字经济建设项目，优先安排项目用地。对信息网络产业、信息技术服务、平台经济等经营服务项目，可参照商服用途落实用地。对数字经济发展较快的开发区基础设施建设项目给予政府债券支持。〔责任单位：省自然资源厅、省商务厅、省财政厅，各市（地）人民政府（行署）〕

　　三、推进产业数字化转型升级

　　10.支持传统产业数字化赋能。实施产业数字化行动，推动传统产业数字化改造升级。对关键业务环节全面数字化规模以上企业比例达到50%的市（地）和县（市），每个完成数字化改造的企业给予10万元奖励。按有关规定，对智能工厂、数字化车间建设给予奖励。〔责任单位：省工信厅、省财政厅，各市（地）人民政府（行署）〕

　　11.支持产业园区数字化改造。支持电信运营商等企业在大型厂区、各类开发区和数字产业集聚区，加快建设5G专网、千兆光网、数据中心等数字化基础设施。建立通信基站用电报审安装绿色通道，支持数字经济领域企业不受电压等级和用电量限制参与电力市场化交易。优先安排风电、光伏建设指标，支持数字产业园区新能源源网荷储一体化配套建设。〔责任单位：省通信管理局、省工信厅、省发改委、省电力公司，各市（地）人民政府（行署）〕

　　12.支持数字化转型促进中心建设。推动数字技术与实体经济深度融合，促进产业数字化转型。支持数字经济领域龙头企业和行业协会建设省级产业数字化转型促进中心，面向产业链上下游企业和行业内中小微企业提供需求撮合、转型咨询、解决方案等服务。对认定的省级产业数字化转型促进中心，给予50万元奖励。〔责任单位：省发改委、省财政厅〕

　　13.支持电子商务加快发展。对新认定的国家电子商务示范基地、示范企业和数字商务企业分别给予30万元、20万元、10万元的一次性奖励。对电商企业实现省内生产实物类商品网络零售额达3000万元以上、同比增长10%以上并在省内入统纳税的，在申报年度内对新增网络零售额按不超过5%的比例给予奖励，最高200万元。〔责任单位：省商务厅、省财政厅〕

　　四、推动数字技术创新与成果转化

　　14.支持创新平台建设。鼓励数字经济领域龙头骨干企业牵头、产业链上下游共同参与、产学研深度合作，共同创建科技创新平台。对新认定的国家产业创新中心、国家工程研究中心、国家技术创新中心和国家制造业创新中心，给予一次性1000万元的建设资金补助。上述平台建成运行后，5年内给予一定研发经费支持。对实质引进的国家级科技创新平台的分支机构，一次性奖励500万元。〔责任单位：省科技厅、省工信厅、省发改委、省财政厅〕

　　15.支持关键技术攻关转化。围绕数字经济重点发展产业，每年采取揭榜挂帅等方式组织实施关键共性技术攻关项目，给予每个项目最高1000万元的资金支持。支持数字经济领域重大科技成果转化项目，单个项目支持额度最高1000万元。〔责任单位：省科技厅、省财政厅〕

　　16.支持知识产权创造。支持数字经济企业、高等院校和科研院所开展专利导航，建立专利预申请补助机制，对提出申请国际、国家专利的，经评优认定后，根据拟申请专利类型给予最高20万元补贴，用于专利申请过程中的各项费用。对获得中国专利金奖、银奖和优秀奖的，分别给予每个奖项50万元、20万元和10万元奖励。〔责任单位：省知识产权局、省财政厅〕

　　17.支持创新创业。鼓励各类孵化载体面向大学生团队提供免费的孵化场地，支持在数字经济领域创新创业。打造为数字经济中小微企业服务的专业融资增信平台，支持数字企业与担保公司、保险公司和银行签订合作协议，最高可获得2000万元的科技担保贷款和300万元的科技保证保险贷款。〔责任单位：黑龙江银保监局、人民银行哈尔滨中心支行，各市（地）人民政府（行署）〕

　　五、营造良好发展生态

　　18.支持数字基础设施建设。各级国土空间规划要为数字基础设施建设预留空间，保障全省信息通信产业发展规划实施。制定相关政策，推动信息基础设施与建筑物、构筑物等空间同步设计、建设和验收。依法免费开放公共设施和国有企业所属建筑物，利用路灯、监控杆、办公楼等公共资源，支持建设基站、机房等通信基础设施。〔责任单位：省市场监管局、省委网信办、省发改委、省住建厅、省通信管理局、省电力公司，各市（地）人民政府（行署）〕

　　19.支持数字类学科建设。鼓励我省高校加强数字类学科建设，提升办学实力，提高学科的人才培育水平。对省内高等院校设置数字类相关专业且取得建设成果的，统筹现有专项资金予以支持。对进入数字经济领域世界一流学科建设的高等院校，统筹“双一流"建设资金给予支持。〔责任单位：省教育厅、省科技厅、省财政厅〕

　　20.支持创新金融服务。鼓励金融机构围绕全省数字经济发展的各类融资需求，畅通融资渠道，创新融资产品。鼓励银行业金融机构优化数字经济领域企业信贷审批流程，开展知识产权、股权、应收账款等质押贷款，扩大信用贷款规模。鼓励数字经济龙头企业以市场化方式设立数字经济发展基金，省级现有股权投资基金通过参股、跟投等方式予以支持。支持企业对接多层次资本市场上市挂牌，按规定分阶段予以特殊奖励。对省内上市公司再融资并用于省内投资的，按实际到账金额的2.5%奖励，最高500万元。〔责任单位：黑龙江银保监局、人民银行哈尔滨中心支行、省地方金融监管局、省财政厅、黑龙江证监局〕

符合本政策措施，同时符合我省其他扶持政策规定，按照从高不重复的原则予以支持，另有规定的除外。

（30）广西壮族自治区数字广西建设领导小组关于印发《广西加快数字经济发展指导意见》的通知 桂数广发〔2021〕14号

各市、县人民政府，自治区人民政府各组成部门、各直属机构：

　　经自治区人民政府同意，现将《广西加快数字经济发展指导意见》印发给你们，请认真贯彻执行。

**广西加快数字经济发展指导意见**

为深入贯彻落实国家关于加快发展数字经济的战略部署，抢抓新一轮科技革命和产业变革机遇，加快培育数字经济新动能，推动我区经济社会高质量发展，结合我区实际，提出如下指导意见。

一、总体要求

（一）指导思想

坚持以习近平新时代中国特色社会主义思想为指导，深入贯彻党的十九大和十九届历次全会精神，全面贯彻落实习近平总书记视察广西“4·27”重要讲话精神和对广西工作的系列重要指示要求，按照自治区第十二次党代会工作部署，把握“一带一路”、区域全面经济伙伴关系协定（RCEP）、数字中国等重大战略机遇，共建中国—东盟信息港，助力打造“数字丝绸之路”，推动数字技术创新，推进数字产业化和产业数字化，深挖数据要素价值，打造具有广西特色的数字经济生态，推动广西数字经济高质量发展，加快建成新时代中国特色社会主义壮美广西。

（二）基本原则

——创新驱动，数据赋能。突出创新驱动，加快绿色发展，构建产业创新平台，加强技术、应用和商业模式协同创新，引领数字经济高质量发展。充分发挥数据资源关键生产要素作用，推进政务和社会经济数据开放共享，深化数据融合应用，释放数据资源价值，激发经济新活力，赋能广西产业振兴。

——开放共享，合作共赢。高水平推进中国—东盟信息港建设，推进与长三角、粤港澳和西南中南等地区的产业合作，深度融入国内大循环和国内国际双循环，加快培育发展开放型数字经济。

——市场主导，政府引导。充分发挥市场在资源配置中的决定性作用，以市场需求为导向，加快培育新业态新模式。积极发挥政府引导作用，为数字经济提供全方位制度和政策支撑，营造良好发展环境。

——协调发展，安全可靠。转变发展方式，把新发展理念贯穿数字经济发展全过程和各领域。统筹协调好发展和安全之间的关系，构建安全可靠的政策、技术和标准规范体系，维护网络安全和经济安全。

（三）发展目标

到 2025 年，数字经济成为广西经济高质量发展重要引擎，数字经济核心产业增加值占地区生产总值比重超 6%，数字经济核心产业规模不断壮大，电子信息制造业产值达到 2200 亿元，软件和信息技术服务业收入达到 1000 亿元，电信业务收入达到 430 亿元，数字经济企业总量达到 21000 家。实现数字技术与实体经济深度融合发展，数字经济开放合作全面深化，探索建设面向东盟科技创新合作区，把广西建设成为面向东盟的数字经济创新示范区、数字经济合作发展新高地和“一带一路”数字经济开放合作重要门户。

二、筑牢数字丝绸之路基础，打造双循环数字合作枢纽

加快夯实数字丝绸之路枢纽基础，共建中国—东盟信息港合作新平台，主动对接国家重大发展战略，打造国内国际双循环重要节点枢纽。

（一）夯实数字丝绸之路枢纽基础。加快推进面向东盟的重点数字基础设施建设，建成运营南宁国家级互联网骨干直联点，推动建设柳州国际互联网专用通道，提升中国—东盟信息港工业互联网标识解析二级节点（广西）和工业互联网标识解析二级节点（柳州）运营能力，支持本土企业在老挝、柬埔寨等东盟国家建设云计算中心，通过数字设施互通促进区域联动。加快推进西部陆海新通道、广西北部湾国际门户港等关键基础设施的数字化升级。加快探索中国与东盟各国数据跨境流动规则对接，推进数字贸易中心建设，构建面向东盟的要素资源数字化融通汇聚枢纽。

（二）共建中国—东盟信息港国际数字合作平台。发挥“云上东博会”集聚效应，打通东盟跨境电商大通道，推进中国—东盟跨境金融服务中心、跨境结算服务平台建设，在跨境电商、跨境金融领域成为对 RCEP 国家合作先导区。推动重点数字产业领域国际化合作，建设跨境人文交流与科技合作服务平台，在智慧城市、数字文旅、智慧广电、数字教育、远程医疗等领域打造一批合作亮点和示范标杆。

（三）加强数字经济区域性对接合作。主动对接长江经济带发展、粤港澳大湾区建设等国家重大战略，加强与长江中游城市群、川渝滇黔产业互补和投资合作，精准建链强链补链延链，大力实施“湾企入桂”行动，鼓励社会组织、行业协会、中介机构为产业对接、转移与合作搭建平台。聚焦数字贸易、数字产业、数字金融、数字物流、数字治理领域，推动数字自由贸易区、重点开发开放试验区建设，积极融入共建“一带一路”，高水平共建西部陆海新通道，打造区域数字经济开放合作门户。

三、激活数字化创新能力，打造数字经济发展新动能

着力构建产业创新平台，全面激发企业创新活力，营造良好创新生态，赋能产业、企业发展。

（四）构建区域级产业创新引领平台。聚焦新一代信息技术、新型显示、通信设备、智能终端、工业应用软件、小语种软件等广西数字经济重点领域发展需求，鼓励行业龙头企业、知名高校院所、行业研究机构等在广西建设产业创新中心、工程研究中心、技术创新中心、企业技术中心等产业创新平台，加快推动应用型技术创新取得新突破。发挥中国—东盟信息港鲲鹏生态创新中心、北部湾大数据交易中心等创新平台在行业应用、产业合作方面的耦合带动作用，推动传统优势产业转型升级和战略性新兴产业发展。构建中国—东盟经济合作交流的新型桥梁与纽带，促进卫星遥感数据资源国际共享，争取中国—东盟卫星遥感服务中心落户广西。

（五）激发企业数字化创新活力。强化企业创新主体地位，推动各类创新资源向企业集聚。落实面向数字经济企业的创新平台奖励、研发费用加计扣除等普惠性政策，发挥重大工程牵引示范作用，运用政府采购政策支持创新产品和服务，通过完善标准、质量和竞争机制等措施，引导企业形成持续稳定的研发投入。完善金融支持科技创新体系，加强金融支持力度，推动“桂惠贷—科创贷”等政策向数字经济企业倾斜。

（六）创新科技成果转化机制。加快建设产教融合创新平台，发挥中国—东盟技术转移中心等国家级技术转移平台集聚国内科技创新资源的作用，支持数字经济龙头企业联合广西大学、桂林电子科技大学、浙江大学、中科院等区内外高校科研院所资源，推动科技成果与产业、企业需求有效对接，建立创新联合体，推进企业、高校、科研机构间的技术要素流动，完善技术成果向企业转移扩散的机制，打造科技成果转移转化高地，推动创新成果产业化。

四、大力推动数字产业化进程，打造多元特色增长极

构建现代化数字产业发展体系，大力发展新兴数字产业，持续壮大电子信息制造业，加快推进软件和信息技术服务产业，布局战略新兴产业，打造重点数字产业集群，不断提升我区数字产业竞争力。

（七）构建数字经济集聚发展新格局。统筹推动全区数字经济特色化、差异化、协同化发展，以南宁为核心，以柳州、桂林、北海为重要节点，建设“四点联动”的数字经济“中轴”，带动以柳州、桂林、百色、河池为主的特色产业数字化示范发展区，以梧州、贵港、玉林、贺州、来宾为重点的桂东承接发展区，以钦州、北海、防城港、崇左为核心的沿海沿边开放发展区三大区域，形成数字经济“一核一轴三区多点”的发展格局，促进全区数字经济协调联动发展。加快推进数字经济示范区建设，推进中国—东盟数字经济产业园、柳州大数据产业园、桂林深科技产业园等数字经济园区建设，大力发展楼宇数字经济，发挥示范区和特色园区的载体作用，吸引区内外知名头部企业入驻，大力培育具有核心竞争力本土骨干龙头企业，培育一批“专精特新”中小企业。

（八）着力发展重点新兴数字产业。加快推进 5G、人工智能、大数据、云计算、区块链、信息技术应用创新产业、量子信息等新兴数字产业发展。依托华为、浪潮等龙头企业，重点发展 5G 通信设备及应用产业，大力发展智能产品、解决方案和智能服务等人工智能产业，创建广西（柳州）国家级车联网先导区。支持南宁、梧州等地发展大数据采集、清洗加工等大数据服务业，吸引粤港澳大湾区大数据向我区迁移，打造“东数西算”新模式。推广区块链技术在政府治理、民生服务、新型智慧城市等领域的应用。发展地理信息、遥感、北斗等空间信息产业，打造地理信息小镇、北斗应用创新基地。依托中国—东盟数字经济产业园、中国—东盟信息港北投数字科技园等载体，以领军企业带动，打造自主创新的信创产业体系。培育量子信息产业，支持各市建设量子信息产业园，推动量子信息与各行业的融合发展。

（九）持续壮大优势电子信息制造业。立足产业基础，围绕“强龙头、补链条、聚集群”， 做大做强做优“芯空屏端网”数字产业。培育集成电路产业，发展北斗、光通信、汽车电子芯片，布局本地芯片封测、设计和宽禁带半导体产业。以智能终端、计算机和网络通信设备、新型显示等为重点，着力提升南宁、桂林等地智能终端、计算机和网络通信设备、新型中高端电子产品产能。加快钦州、北海、贺州、河池等地智能终端及新型显示研发生产。积极发展柳州等地汽车电子产品研发生产及车联网、车载智能系统等新业态。

（十）加快发展特色软件和信息技术服务产业。加大通用软件和行业应用软件研发力度，提升高端软件供给能力。推进工业软件发展，发展基于网络协同的办公软件等通用软件，研发面向金融、教育等领域的高质量行业应用软件。支持科大讯飞、广西达译科技等企业研发面向东盟非通用语种相关语音及翻译核心技术和语料库，开发基于网络实时翻译、语言包开发等技术的各类小语种软件和服务。鼓励企业开放搜索、电商、社交等数据，发展第三方大数据服务产业。依托南宁·中关村创新示范基地、中盟科技园、中国电子北部湾信息港等重点产业园区，培育软件和信息技术服务业生态群。

五、深入推进数字化融合发展，打造经济发展新引擎

以供给侧结构性改革为主线，加强数字技术与经济社会深度融合发展，以数字化转型整体驱动生产及生活方式变革，以数字赋能催生新产业新业态新模式。

（十一）重点推进数字技术与工业深度融合发展。推动“工业互联网+安全生产”产业体系建设。实施“上云用数赋智”工程，加快工业设备和企业上云用云步伐，鼓励龙头企业发挥带动作用，深化研发设计、生产制造、经营管理、市场服务等环节的数字化应用。推动冶金、建材、化工、纺织服装等产业实施机器换人、生产换线、设备换芯，打造国家智能制造标杆企业、广西智能工厂示范企业、广西数字化车间。培育众创设计、网络众包、个性化定制、服务型制造等新模式。加快产业园区数字化改造。

（十二）加快推进数字技术与农业深度融合发展。加快发展智慧农业，推进农业生产经营和管理服务数字化改造。立足特色资源，实施“互联网＋现代农业”行动，打造数字农业示范样板。依托广西农业农村大数据管理平台，提升农业智能化管理水平。大力发展农村电商，积极推进“互联网+”农产品出村进城工程，对接全国知名电商平台，促进广西品牌农产品走出去。

（十三）全力推进数字技术与服务业深度融合发展。大力推进生产性服务业高端化延伸，培育壮大电子商务、智慧物流、数字金融。积极推进生活性服务业多样化升级，支持“智慧广电”“智慧市场”等互联网平台向商贸、教育等领域延伸拓展，大力发展全域智慧文旅、远程医疗。加快信息无障碍建设，帮助老年人、残疾人等共享数字生活。依托“智能＋”推动互联网、大数据、人工智能与实体经济深度融合，鼓励数字经济企业扩大服务贸易出口。

（十四）加快培育发展新业态新模式。加快培育在线经济、智能零售、共享经济、平台经济、无人经济等新模式新业态。建立健全各类“互联网+”平台，大力发展“互联网+教育”“互联网+健康医疗”“互联网+旅游”等线上服务新模式。鼓励发展消费新模式，支持线上企业与城市商圈联合开展“广西 33 消费节”等活动，宣传推广广西产品。鼓励区内企业开放自身资源，共享实验验证环境、仿真模拟等技术平台。促进平台经济发展，引导微商电商、网络直播、短视频平台等有序发展。发展“无人经济”，推广不见面交易、零接触服务。

（十五）大力推进智慧城市与数字乡村建设。以数字化助推城乡发展和治理模式创新，全面提高运行效率和宜居度。推动城市运营管理平台集成与信息共享，推进 “智桂通”平台建设，打造面向东盟的新型智慧城市样板。加快数字乡村建设，深入实施国家和自治区数字乡村试点，构建面向农业农村的综合信息服务体系，推进智慧广电乡村建设，建立涉农信息普惠服务机制，推动乡村管理服务数字化。

（十六）着力推动数字海洋强区建设。深化数字技术与传统海洋产业融合，加快建设数字海洋产业园，推进海洋产业数字化升级，加快建设智慧港口、智慧渔业应用平台。建立覆盖中国（广西）近海和东盟国家的立体化海洋信息服务网络，提升海洋监控、监管、生态保护和远洋救助能力。

六、加快推进数据价值化，打造全域数据要素新市场

以数据作为数字经济发展的关键生产要素，推进政务、经济、社会数据流通，打造“大数据+”应用生态，深化数据融合应用，释放数据资源价值。

（十七）促进数据资源全领域流通。拓展数据资源采集渠道，推动人工智能、可穿戴设备、车联网、物联网等领域数据采集标准化。整合优化公共数据资源平台，完善基础数据库及主题数据库，加快推进各类政务数据、经济数据和社会数据归集共享。完善数据开放共享标准规范，扩大数据开放共享范围。推进数据要素市场化配置改革，建立健全数据交易规则、安全保障体系和平台监管机制，培育规范的数据交易平台和市场主体，构建数据交易生态，实现数据价值最大化。开展数据跨境流动试点，建立跨境数据市场化流通和交换机制，推动国际信息流量在广西集聚落地。

（十八）打造“大数据+”应用生态。鼓励社会力量参与“互联网+公共服务”，创新提供服务模式和产品。深入开展公共数据治理，形成全区公共数据资源“一张图”，提升政府数据资源服务能力。扩大数据开放范围，引导具有公共属性企业参与数据开放，探索政企合作开展数据运营，推动高价值社会数据流通利用。实施数据要素融合应用“百千万工程”，推动政务数据与经济社会数据、产业数据创新性融合，推动数据要素与医疗健康、社会保障、城市服务、金融服务、教育文体、交通旅游、应急管理等多领域深度融合，形成一批高质量的数据要素融合示范应用，推动“大数据+”应用生态加速形成。丰富数字生活体验，发展数字家庭。

（十九）筑牢数据安全保障防线。建立数据安全治理协同体系，推进数据分类分级管理，开展数据安全评估。建设数据安全态势感知平台，提升数据安全隐患监测、分析与处置能力。推进“安全大脑”建设，构建一体化数据安全体系。

七、创新数字化治理模式，打造数字安全治理新体系

（二十）创新数字经济治理模式。加强基于信用的监管创新应用。完善广西公共信用信息平台，推动信用信息、监管信息全程可追溯和“一网通享”，建立信用联合奖惩机制，完善社会组织、企业等信用信息公示系统，鼓励企业、研究机构建设第三方信用信息服务平台，提供精准化信用服务。探索技术和算法经济的治理创新，聚焦数字内容、电子商务和人工智能等领域，加强对代码、算法等数字技术应用和规则有效监管，加强区块链技术引导和安全规范，推动区块链安全有序发展。营造知识产权保护优越环境，推进知识产权协同保护机制建设，开展重点企业知识产权保护直通车服务，加强重点领域知识产权执法和专利代理行业监管。

（二十一）强化安全发展和多元共治。加快数字技术在生产安全、自然灾害、城乡公共安全、防灾减灾救灾、食品药品安全、疫情防控等监管体系的应用，实现安全事件“源头可溯、环节可控、风险可防”。建立部门联动、区域协同的危化品信息化监管平台机制，形成危险货物运输全链条安全监管体系。推进数字经济治理手段多样化，建立政府监管、行业自律、公众监督“三位一体”的有效协同、共治共享的数字经济治理机制。加强平台责任主体制度建设，形成技术赋能和多元共治为内核的数字经济开放治理体系。

八、保障措施

（二十二）加强组织实施。发挥数字广西建设领导小组作用，统筹推进全区数字经济发展工作，研究落实数字经济发展战略、总体规划和政策措施。发挥数字广西专家咨询委员会作用，为数字经济发展战略研究、政府科学决策提供技术支撑和智力保障。建立多部门协调机制，通过改革试点、专题会议等多种方式，及时解决数字经济创新发展中存在的制度性、政策性障碍。

（二十三）加大要素保障。统筹全区数字经济相关专项资金支持数字经济重点领域重大项目建设。引导和鼓励社会资本按市场化方式发起设立面向数字经济各细分领域的各类产业、科创投资基金，形成产业基金群。强化对数字经济发展重大项目信贷支持，落实小微企业信贷支持政策。加大数字经济项目用地支持力度，实施差别化的地价政策，保障重大项目建设用地。积极引进数字技术领军人才，建立数字技术及应用人才教育体系，构建企业数字经济人才培训平台。

（二十四）优化发展环境。加快落实促进广西数字经济发展的各项扶持政策，持续深化“放管服”改革，不断优化营商环境。加快研究出台大数据发展条例，制定公共数据资源开放清单，分步开放数据。实施全民数字教育计划，持续提升全民数字素养和能力。加强宣传推广，通过各类媒体加强对我区数字经济发展政策解读、企业典型宣传引导，形成良好社会氛围。

（二十五）强化督查考核。建立健全数字经济发展考核评价机制。建立数字经济发展评估体系，加大督促检查力度，适时开展数字经济发展情况监督检查，综合运用第三方评估、社会监督评价等多种方式，科学评估建设效果。

广西壮族自治区数字广西建设领导小组（代）

2021年12月19日

（31）贵州省大数据局关于印发《贵州省数字经济领域重点人才计划实施办法(试行)》的通知 黔数〔2021〕29号

各市(州)大数据发展主管部门,省直有关单位,各相关单位:

　　现将《贵州省数字经济领域重点人才计划实施办法(试行)》印发给你们,请结合实际认真贯彻落实。

贵州省大数据局

2021年9月30日

**贵州省数字经济领域重点人才计划实施办法(试行)**

　　第一章 　 总  则

　　第一条 　 坚持以习近平新时代中国特色社会主义思想为指导,深入贯彻习近平总书记关于人才工作重要论述和视察贵州时指出“在实施数字经济战略上抢新机"的要求,为加快培养造就一批数字经济重点人才,以选拔培养我省数字经济重点人才为目标,创新培养模式、强化服务管理,为我省发展数字经济提供坚强的人才保障。根据全省人才工作推进会议的有关精神,结合发展实际,特制定本办法。

　　第二条 　 数字经济领域重点人才是数字经济发展的核心驱动要素,是我省贯彻落实大数据战略行动的重要支撑保障。主要面向全省遵纪守法,品德优良,为我省数字经济发展做出积极贡献的人员。

　　第三条 　 聚焦数字经济发展,在“数字产业化、产业数字化、数字化治理、数据价值化"四个领域遴选从事理论、科研、产业发展等方面的优秀人才进行培养。通过3年培养周期,提升培养对象在科学技术研究、技术创新、模式创新、应用创新和产业发展等方面的能力,并形成良好的人才带动效应,进一步促进数字经济领域人才队伍建设。

　　第四条 　 省大数据局负责贵州省数字经济重点人才培养计划的遴选、管理、考核等工作。突出用人单位对人才培养、引进和使用的主体作用,按照“公开、公平、公正、择优"的原则,注重创新引领、注重产业贡献,确保质量、宁缺毋滥。

　　第二章 　 申报条件

　　第五条 　 申报对象。贵州省境内注册的具有独立法人资格的企(事)业单位中任职满6个月的人才,原则上年龄在45岁以下。

　　第六条 　 申报条件。申报对象必须拥护中国共产党的领导,自觉遵守国家法律法规,诚善守信,具有良好的职业操守,社会信用良好,无违法违纪行为。在数字经济领域有重要理论成果或重大技术突破、技术发明、技术创新,解决重要科学问题或技术难题,在应用推广方面做出突出贡献,产生重大的经济效益和社会效益,对行业的发展推动作用较大,为数字经济发展做出积极贡献,得到业内广泛认可。同时,还应具备以下条件之一:

　　1.获得市(州)级及以上人才类称号、奖励等。

　　2.近3年主持过省级及以上大数据相关工程项目、科研项目或市场化、社会化委托的具有一定影响力的项目,取得较好的社会和经济效益。项目成果具有较高应用水平和引领、示范效应,并获得成果登记。

　　3.上市公司主要项目(技术)负责人;或独角兽企业主要项目(技术)负责人;或省级相关部门授予“专精特新"企业主要技术负责人;或省级融合标杆项目主要技术负责人。

　　4.省级及以上重点学科、重点实验室、工程技术(研究)中心、工程实验室相关项目(技术)负责人。

　　第三章 　 申报评选

　　第七条 　 自愿申报。申报人可根据从事的工作领域并结合自身实际申报。

　　第八条 　 逐级推荐。按照隶属关系,逐级向主管部门申报。由主管部门统一出具推荐函及政治表现情况报省大数据局。

　　第九条 　 组织评审。由省大数据局牵头,联合有关部门共同组织专家,对推荐上报的材料进行评审,提出候选人名单。

　　第十条 　 名单公示。经专家组评审推荐、评审组初步审核的候选人名单,在省大数据局门户网站进行公示。

　　第十一条 　 审定公布。公示无异议后,由省大数据局党组审定,并报省人才工作领导小组同意后行文公布。

　　第四章 　 培养措施

　　第十二条 　 入选对象由省大数据局联合有关部门进行重点培养。

　　1.在人才评选、职称评审等方面给予支持。优先推荐入选对象申报国家、省级人才项目。入选对象可不受下一级职称任职资格限制,直接申报相应工程系列大数据专业职称。

　　2.支持参与各类社会活动。积极推荐入选对象进入国家、省各领域咨询委员会、专家库,作为各级政府及部门的技术咨询专家。结合入选对象的需求,协调参加各类调研考察、学术交流、专业论坛、规划论证、项目论证等活动。

　　3.支持制定年度培养计划,推动计划落实落细。与入选对象共同研究制定年度培养计划,在科学研究、项目建设、培训锻炼、专项资金基金申报等方面给予支持。

　　4.帮助入选对象提升能力。结合入选对象需求,优先推荐入选对象参加“西部之光"等人才培训项目,到国内高校、科研院所研修、访学,融入科研主流,掌握前沿动态,参与重大课题(项目)。

　　5.采取省级人才引进专项政策,积极帮助入选对象培育研发团队,引进所需的特殊人才或高级助手。

　　6.协调有关部门落实人才政策,共同支持入选对象的发展。

　　第五章 　 考核评估

　　第十三条 　 考核评估分为年度评估和期终考核,考核评估结果分为优秀、合格、不合格三个等次,是作为享受培养措施、管理期满考核的重要依据。年度评估和期终考核由省大数据局组织实施。第一年、第二年对培养对象进行年度评估,第三年对培养对象进行期终考核。

　　第十四条 　 考核内容

　　参照申报时提出的个人成长目标及培养措施,重点围绕以下方面开展考核评估:

　　1.思想政治表现、职业道德等方面情况。

　　2.理论成果、技术突破、技术发明、技术创新情况。

　　3.解决重要科学问题或技术难题情况。

　　4.大数据应用推广方面情况。

　　5.开展人才培养和团队建设等情况。

　　6.个人或团队取得各类人才称号、奖励等情况。

　　7.其他有关情况。

　　第十五条 　 考核评估结果为不合格等次的,不再继续作为培养对象进行管理,且三年内不得再次申报。考核结果等情况在系统范围内进行通报。培养对象职务变动、辞职、调动、受党纪政纪处分等重大情况,所在单位须按程序及时向省大数据局书面报告。

　　第十六条 　 入选对象应珍惜荣誉、严格自律。管理期内,存在以下情况的人员,不再享受相关支持政策,并依法追回获得的支持资金等。

　　1.违反国家法律法规或犯有严重错误,受到党纪政纪处理的;

　　2.工作中因个人责任给国家、集体或他人造成重大损失的;

　　3.出国出境定居或未经组织同意逾期不归的;

　　4.有学术不端行为经查实的;

　　5.考核考评被评定为不合格等次的;

　　6.有其他不适宜继续作为培养对象情况的。

　　第六章 　 强化服务

　　第十七条 　 省大数据局联合入选对象所在单位及主管部门,有针对性地对入选对象开展培养,并做好服务。主管部门相关负责同志,对本地入选对象要定期联系和看望,详细了解人才的工作和生活情况,及时帮助解决困难和问题。所在单位要在科研团队建设、实验设备、实践平台以及科研经费等方面提供保障。

　　第七章 　 附  则

　　第十八条 　 本办法由省大数据局负责解释。

第十九条 　 本办法自印发之日起施行。

（32）广西壮族自治区数字广西建设领导小组关于印发《广西壮族自治区楼宇数字经济发展规划（2021-2025年）》的通知 （桂数广发〔2021〕1号）

各市、县人民政府，自治区人民政府各组成部门、各直属机构：

　　经自治区人民政府同意，现将《广西壮族自治区楼宇数字经济发展规划（2021-2025年）》印发给你们，请认真贯彻执行。

广西壮族自治区数字广西建设领导小组（代）

2021年1月15日

**广西壮族自治区楼宇数字经济发展规划（2021-2025年）**

二○二一年一月

目　录

　　序　言

　　一、发展基础和面临形势

　　（一）战略意义

　　（二）发展基础

　　（三）面临形势

　　二、总体思路

　　（一）指导思想

　　（二）基本原则

　　（三）发展目标

　　三、空间布局

　　（一）打造区域内楼宇数字经济引领核心

　　（二）提升楼宇数字经济中轴示范效应

　　（三）推动楼宇数字经济多点特色发展

　　四、推进楼宇经济数字化转型

　　（一）建设楼宇数字孪生空间

　　（二）完善楼宇数字服务体系

　　（三）培育楼宇创新创业平台

　　（四）创建楼宇虚拟产业集群

　　五、加快数字经济楼宇化集聚

　　（一）培育数字经济新型楼宇

　　（二）建设数字经济品牌楼宇

　　（三）构建楼宇数字经济生态圈

　　（四）打造楼宇数字经济集聚区

　　六、夯实楼宇数字经济发展基础

　　（一）完善楼宇数字经济发展政策

　　（二）创新机制激发楼宇数字经济活力

　　（三）加强楼宇数据资源汇聚共享

　　（四）构建楼宇数字发展组织支撑

　　七、保障措施

　　（一）加强组织领导

　　（二）强化政策支持

　　（三）加大资金扶持

　　（四）加强人才引育

　　（五）优化服务保障

　　（六）加强监督评估

　　附件1：名词解释

　　附件2：全区各设区市商务楼宇统计表

　　附件3：全区各设区市5A级写字楼发展情况表

序　言

　　楼宇作为宏观经济活动和社会发展的主要载体，是推动广西经济快速、协调发展的重要抓手。2020年7月30日，中共中央政治局会议指出，必须从持久战的角度加以认识，加快形成以国内大循环为主体、国内国际双循环相互促进的新发展格局。广西地理位置独特，处华南经济圈、西南经济圈与东盟经济圈的结合部，为加快构建我区的新发展格局，推进我区楼宇经济与数字经济的融合协同发展，培育数字经济发展新引擎，打造区内集聚、辐射华南、面向东盟的楼宇数字经济新风向，特制定本规划。

　　楼宇数字经济是在数字经济加速崛起的背景下，以楼宇为集中体现的细分经济形态，包含楼宇经济数字化和数字经济楼宇化两方面内容。其中，楼宇经济数字化是依托大数据、云计算、人工智能、5G等新一代信息技术和数字化的管理和运营模式，促进商务楼宇数字化转型升级，探索融合发展的新业态新模式；数字经济楼宇化是以楼宇为载体集聚数字经济产业链上的相关服务业和制造业的商务功能，使各企业依托楼宇空间实现互相配合、协调发展。楼宇经济数字化是实现数字经济楼宇化的基础条件和必要准备，数字经济楼宇化是楼宇经济数字化的终极目标和充分展现。

　　本规划依据《国家信息化发展战略纲要》、《广西壮族自治区人民政府关于印发广西壮族自治区国民经济和社会发展第十三个五年规划纲要的通知》（桂政发〔2016〕9号）、《关于支持新业态新模式健康发展激活消费市场带动扩大就业的意见》（发改高技〔2020〕1157号）、《中共广西壮族自治区委员会广西壮族自治区人民政府关于深入实施大数据战略加快数字广西建设的意见》（桂发〔2018〕16号）、《广西壮族自治区人民政府关于印发广西数字经济发展规划（2018-2025年）的通知》（桂政发〔2018〕39号）、《广西壮族自治区人民政府办公厅关于印发中国-东盟信息港建设实施方案（2019-2021年）的通知》（桂政办发〔2019〕72号）等文件，并与相关专项规划、详细规划等实现相互协同和衔接。规划期为2021年至2025年。

　　一、发展基础和面临形势

　　（一）战略意义

　　1. 发展楼宇数字经济是落实国家和自治区发展战略的重要抓手。中国（广西）自由贸易试验区、中国-东盟信息港、西部陆海新通道、面向东盟的金融开放门户、中国（南宁）跨境电子商务综合试验区等国家战略多重叠加，结合我区加快推进数字广西建设战略，立足国内大循环、畅通国内国际双循环，扩大内需和对外开放，稳定产业链供应链，以扩大内需作为促进经济增长，落实“六稳”“六保”的基础任务，加速推进要素市场化改革，吸引企业落户广西，提升集聚效应和优化发展空间。以楼宇为企业落户的主要空间载体，加快新一代信息技术赋能楼宇转型升级，发挥、优化楼宇的空间承载作用，加快楼宇物理空间和数字空间的协同发展，先行先试探索楼宇数字经济新业态新模式，助推我区数字产业发展、数字经济园区建设和楼宇数字经济集聚效应提升，是落实国家和自治区发展战略的重要抓手。

　　2. 发展楼宇数字经济是培育我区经济新动能的重要途径。我区正处在转变经济发展方式、推动经济高质量发展的关键时期。缓解经济下行压力、挖掘培育经济新增长点成为广西“十四五”时期的一项重要任务。数字经济是新时期楼宇经济发展的内生动力，推动两者融合发展是培育新经济增长点的重要举措。推进楼宇经济数字化，应用新一代信息技术，构建数据驱动型的新型楼宇发展体系，实现系统升级、业务创新、流程优化和管理变革，提升供应链响应速度和协同效率；数字经济楼宇化，不仅是楼宇内部的垂直空间集成，更应是突破空间限制的跨域跨国界的横向产业集成，有利于增强各主体间的信任和协作，降低沟通成本，健全具有广西特色的楼宇数字经济生态体系，实现数字经济高增长点的培育和高质量发展的突破。

　　3. 发展楼宇数字经济是“数字广西”建设的重要内容。我区正依托中国-东盟信息港建设，加快推进“数字广西”建设，尤其是大力发展数字经济，深入推动数字产业化和产业数字化。以“数字广西”建设为契机，发挥楼宇经济引导作用，高质量发展楼宇数字经济，推动楼宇经济数字化转型，加快数字产业向楼宇集聚，着力建设楼宇数字孪生空间、打造楼宇智慧服务体系、培育楼宇创新创业平台、创建楼宇虚拟产业集群，着力培育特色数字经济楼宇、建设数字经济品牌楼宇、构建楼宇数字经济生态圈、打造楼宇数字经济集聚区，提高我区经济运行质量，提升城市建设管理水平，是“数字广西”建设的重要内容。

　　（二）发展基础

　　1. 数字经济发展壮大成为新动能。“十三五”期间，我区数字经济快速发展，总体规模从2015年的3400亿元增长至2019年的6600亿元，占同期全区GDP的比重从20%提高到31%。截至2020年6月底，全区数字经济企业达到11200家，在西部地区排名第4位。数字经济与传统经济融合发展不断增强，2019年全区高技术制造业增加值占规模以上工业增加值比重达7%，软件和信息技术服务业营业收入同比增长32%；智能电视产量同比增长2倍多，手机同比增长72%，电子元件同比增长12%。全区初步形成“一核心两引擎，三热点两带动”，即以南宁为核心，以北海、钦州及柳州、桂林为南北部双引擎，以数字基础设施、数字社会应用、数字产业化为三大热点，以数字政务和产业数字化为带动的数字经济发展总体态势。

　　2. 楼宇经济快速发展进入新阶段。近年来我区产业结构持续优化，新动能加快成长，呈现稳中提质的发展态势。第三产业保持较快增长，2019年第三产业增加值占全区生产总值比重达51%，其中，新兴服务业保持较快增长，规模以上服务业中互联网和相关服务营业收入同比增长1.2倍，规模以上软件和信息技术服务业营业收入同比增长32%，规模以上高技术服务业营业收入同比增长13.7%。全区重点城市商务楼宇和数字产业园建设加快推进，2019年全区商办楼宇成交面积约230万平方米，各市商务楼宇项目超过400个。其中，南宁市有近120个商务楼宇项目，柳州市46个、北海38个、桂林36个分列第二至第四位；全区5A级写字楼项目30余个，其中，南宁市5A级写字楼占比超六成，商务楼宇稳步发展为楼宇数字经济发展提供坚实基础。重点区域楼宇发展成绩显著，南宁市五象新区总部基地金融街成为全区楼宇最为密集的区域，青秀区近年连续获得“中国楼宇经济服务创新城区”、“中国楼宇经济十大潜力城区”、“南宁市楼宇经济示范城区”等称号；柳州市在城市东进的带动下，河东片区正成为商务楼宇的主要集聚地，集聚华润大厦、万达写字楼、地王大厦、阳光100等楼宇群；桂林市高新区按照产业集聚、产城融合的空间发展布局，实施“四城四带”空间发展战略，打造国家创新型特色产业园区和高端智能聚集园区；北海市加速冯家江区域和廉州湾区域两个城市CBD建设，加快建设超高层楼宇以及科技、康养园区。

　　3. 信息基础设施建设取得新成效。以5G、数据中心、工业互联网为代表的新型信息基础设施和以光纤宽带为代表的传统信息基础设施，以及中国-东盟信息港、“数字广西”、“广电云”村村通户户用工程建设取得新成效。截至2019年年底，全区共建成开通5G基站达4400个，实现重点景区、交通枢纽、重点商圈、重点城市干道、重点高校医院等区域5G网络覆盖；区市两级政府机关和数据中心运营企业已建、在建数据中心787个，其中，超大数据中心11个，大型数据中心4个，中型数据中心19个，小型数据中心753个，总承载能力为29万标准机架；建设光缆线路总长度176万公里，互联网省际出口带宽达2500万兆，基础电信企业固定互联网宽带接入用户达1447万户，光纤到户法人用户占比达到94%，物联网终端用户达到477万户，广播电视光缆总长29万公里，联通1.24万个行政村、5.2万个自然村，高清互动电视用户377万户。中国-东盟信息港互联互通通信基础设施逐步完善，形成“一云承载，一网通达，一池共享”的政务基础设施发展格局。

　　（三）面临形势

　　1. 楼宇数字经济发展面临的机遇

　　一是广西多重战略机遇叠加助力楼宇数字经济发展。中国（广西）自由贸易试验区、中国-东盟信息港、西部陆海新通道、面向东盟的金融开放门户、中国（南宁）跨境电子商务综合试验区等重大国家战略多重叠加，将持续催生新模式、新业态，为楼宇数字经济发展带来广阔市场空间。其中，中国-东盟信息港建设扎实推进，加快建设面向东盟的信息枢纽和数字经济产业，中国-东盟信息港南宁核心基地、中国-东盟信息港钦州副中心、中国-东盟网络视听产业基地等区域加速成型，有效推动连接中国与东盟的“数字丝绸之路”建设，为发展楼宇数字经济带来重大发展机遇。

　　二是国家支持数字经济集聚提质发展的力度不断加大。近期党中央、国务院加大对数字经济相关领域的谋划部署。2020年3月中央政治局常务委员会会议提出加快5G网络、数据中心等新型基础设施建设进度；2020年4月，国家发展改革委、中央网信办联合发布《关于推进“上云用数赋智”行动培育新经济发展实施方案》；2020年5月，国家发展改革委联合17个部门以及互联网平台、行业龙头企业、金融机构等145家单位共同启动“数字化转型伙伴行动”；2020年7月，国家发展和改革委、中央网信办等13个部门联合印发《关于支持新业态新模式健康发展 激活消费市场带动扩大就业的意见》，提出支持数字经济15类新业态新模式发展的政策措施。

　　三是广西数字经济发展提速带来楼宇空间承载需求。我区持续推动数字经济发展，加快数字产业化进程，推动产业数字化转型升级。从企业上云到工业互联网“智能＋”带来新变革，从“暖心生活节”、“线上＋线下”新零售激活“内动力”，到数字广西建设打造经济发展“新引擎”，再到中国-东盟信息港重大工程、数字产业项目群和创新平台创建，一大批新业态新模式不断涌现出来。2019年，全区数字经济规模总量达到6600亿元，实现总量、增速、比重全国排名“三提升”。数字经济规模的持续扩大，释放出优化承载空间的巨大需求，为以楼宇为载体推进数字产业化和产业数字化提供了重要基础，为楼宇数字经济发展带来广阔前景。

　　2. 楼宇数字经济发展面临的挑战

　　一是楼宇数字经济规划和顶层设计有所缺失。楼宇经济与数字经济融合发展尚处于起步阶段，缺少政策支持、重点关键技术攻关、重大示范工程、系统解决方案和公共服务平台建设等方面的统筹规划和顶层设计，扶持政策措施亟待出台，管理体制机制有待创新，组织支撑尚需构建。

　　二是楼宇与数字经济融合的深度广度有待提升。楼宇经济与数字经济融合发展的新业态、新模式有待进一步培育，数字经济规模仍需扩大，数字产业结构仍需优化，数字经济特色楼宇和楼宇数字经济生态圈仍需培育，数字楼宇品牌评价和认定体系有待建立。

　　三是楼宇数据要素有序流通、高效利用的新机制尚未形成。全区数字化楼宇或智慧楼宇数量和发展水平仍需提升，楼宇数据要素有序流通、高效利用的新机制尚未形成。楼宇经济数字化基础设施建设亟待加强，楼宇经济信息尚未有效归集和有序汇聚，楼宇经济运营管理和政务服务亟需强化，楼宇数字经济统计评价体系亟待建立。

　　二、总体思路

　　（一）指导思想

　　以习近平新时代中国特色社会主义思想为指导，按照“五位一体”总体布局和“四个全面”战略布局要求，牢固树立创新、协调、绿色、开放、共享的发展理念，坚持创新驱动、开放带动、市场主导、重点突破、特色发展，紧紧围绕中央赋予广西的“三大定位”，顺应信息技术革命、产业结构调整、消费升级趋势，按照“规划先行、创新驱动、数字引领、融合发展、统筹布局、做强特色”的发展思路，推动楼宇经济数字化和数字经济楼宇化，使楼宇数字经济成为我区经济发展重要形态，成为经济转型升级和高质量发展的新引擎、新动力，为扎实推进壮美广西建设提供强有力支撑。

　　（二）基本原则

　　1. 政府引导，市场主导。强化顶层设计、规划引领，遵循发展规划和市场调节功能，推进楼宇数字经济业态能级提升和业态结构优化，强化政府对数字经济发展的政策支持，不断提升楼宇数字经济发展的内生动力。

　　2. 统筹推进，因地制宜。从各设区市的优势和特点出发，鼓励因地制宜聚焦重点领域和关键环节，培育特色鲜明的楼宇数字经济比较优势。依靠特色和优势提高竞争力，发展规模化、品牌化和特色化楼宇。

　　3. 创新驱动，融合发展。实施创新驱动发展，推动数字经济与楼宇业务关联、链条延伸和相互渗透，加快培育楼宇经济与数字经济融合发展的新业态，探索楼宇经济数字化转型发展新路径。

　　4. 集聚发展，打造生态。引导项目和企业向优势区域的楼宇集聚，推进数字产业向楼宇集聚。构建数字生态圈，推动数字经济与楼宇经济融合发展，加快楼宇数字经济产业链、价值链的优化提升。

　　5. 数字引领，智能转型。以国家战略、“数字广西”建设等为契机，加快全区楼宇“新网络”“新平台”和“新服务”等建设，加快构建楼宇数字孪生，强化楼宇全生命周期管理，推动楼宇经济数字化转型升级。

　　（三）发展目标

　　到2025年，全区楼宇数字经济规模占服务业比重大幅提升，形成一批创新活跃、效益显著、带动效应突出的楼宇数字经济新业态新模式，培育一批楼宇数字经济示范区、楼宇数字经济发展生态圈，将广西建设成为国内楼宇数字经济高质量发展先行区，国家面向东盟的楼宇数字经济示范区。

　　1. 楼宇数字经济实力显著增强。到2023年楼宇入驻率和入驻企业质量不断提升，楼宇成为数字产业企业选址首选，形成一批有规模、上档次的特色数字楼宇群，引进和培育一批新一代信息技术龙头企业和创新创业企业。到2025年楼宇产业完成向数字化、网络化和智能化转型，数字经济与楼宇经济融合度不断提升，全区楼宇成为数字经济发展的主阵地。

　　2. 数字楼宇新业态不断涌现并发展壮大。到2023年云计算、大数据、5G网络、人工智能等新一代信息技术不断为产业赋能，依托楼宇的智能制造、工业互联网等为代表的工业融合新业态，以精准农业、农村电商等为代表的农业融合新业态，以移动支付、共享经济等为代表的服务业融合新业态，蓬勃发展。到2025年形成具有较强竞争力的楼宇数字产业体系。

　　3. 楼宇数字经济集聚效应不断加强并形成规模。到2023年建成一批品牌明显、特色突出、税收贡献大的数字产业楼宇，自治区级楼宇数字经济示范区建设取得重大成效，形成若干楼宇数字经济发展样板区。到2025年楼宇数字要素和辐射带动作用得到全面提升，数字经济特色楼宇生态圈基本成型。

　　4. 楼宇数字经济发展基础进一步夯实。到2023年数字经济发展扶持政策进一步完善，楼宇营商环境大幅优化，数字化管理、服务机制不断创新提升。建成楼宇经济数据资源体系，实现楼宇数据资源汇聚共享。到2025年楼宇新型基础设施建设标准、新型楼宇经济数字化解决方案指引全面明确，新一代信息技术基础设施促进楼宇转型升级全面铺开，数字化升级改造传统楼宇取得重大成效，建成若干在全区具有示范效应和带动作用的数字楼宇、智能大厦。

　　表1　广西楼宇数字经济发展主要指标

指标类型具体指标20232025年

发展规模培育面向东盟的楼宇数字经济示范区（个）11～2

培育自治区级楼宇数字经济示范区（个）25

培育市级楼宇数字经济示范区（个）410

发展质量已建成楼宇企业入驻率（%）65以上80

税收超千万元的楼宇数量（幢）50100

空间集聚入驻数字经济品牌楼宇的数字经济企业数量占比（%）3040

培育数字产业特色明显的专业楼宇数量（幢）不低于50不低于100

基础环境全区各市楼宇信息平台覆盖率（%）60100

重点楼宇纳入楼宇信息平台覆盖率（%）60100

　　三、空间布局

　　立足全区数字经济产业发展基础，按照突出优势、凸显特色的原则，加快全区优质资源和要素向重点区域集中，促进空间资源优化整合、分工合作，推进各市数字经济错位发展、融合发展和协同共进，打造“一核引领，一轴示范，多点发展”的全区楼宇数字经济发展总体格局，即以南宁市为全区楼宇数字经济发展核心和龙头，引领全区其他地市共同发展；以桂林-柳州-南宁-钦州-北海为全区楼宇数字经济发展中轴，建设一条具有示范带动作用的全区楼宇数字经济发展带；其他设区市因地制宜推动楼宇数字经济差异化特色发展。形成区内集聚、辐射华南、面向东盟的楼宇数字经济新格局。

　　（一）打造区域内楼宇数字经济引领核心

　　南宁市。充分利用中国-东盟信息港南宁核心基地建设发展机遇，依托五象新区、青秀区、南宁高新区、南宁经开区等重点区域推动大数据、信息服务、数字金融、跨境电商、智慧物流、智慧城市、智慧广电、北斗导航应用、网络文化、智慧医疗、数字中介服务等数字产业向楼宇集聚，打造一批楼宇数字经济集聚区和示范区。重点加快五象新区总部基地金融街、中国-东盟电子商务产业园、中国-东盟网络视听产业基地、南宁高新区中关村双创示范基地、青秀区数字经济产业园区等楼宇数字经济集聚区建设，加速楼宇经济与新一代信息技术及相关产业融合发展。在全区率先探索楼宇全生命周期管理机制、楼宇智慧大脑，全面推进楼宇智慧化建设。

专栏1 南宁市楼宇数字经济发展工程1. 发展目标：有重点有步骤的聚合一批特色产业、培育发展一批特色楼宇、创建一批示范性楼宇经济聚集区，新增培育特色楼宇20个，实现税收过千万的楼宇60座、税收超亿元的楼宇20座，楼宇税收总量较规划初期提升20%，重点楼宇所占城区当年财税收入比上年提升不少于3个百分点。2. 建设重点：推进建设一批辐射华南、面向东盟，具有示范带动作用的楼宇数字经济示范区。

　　（二）提升楼宇数字经济中轴示范效应

　　按照“以点带线、以线带面”的推进方式，加快构建桂林-柳州-南宁-钦州-北海发展轴线，形成广西楼宇数字经济发展的“中轴示范带”。

　　1. 桂林市。依托桂林高新区、经开区、高铁园和雁山区等重点区域，大力发展智慧旅游、智慧物流、智慧康养、电子商务、数字文化创意、智慧医疗等数字产业；依托现有楼宇建设一批线上线下结合、业务跨界融合的信息平台，重点建设智慧旅游管理、服务和营销平台。大力培育发展光通信、北斗导航、物联网等新一代信息技术服务、旅游服务、科技服务等楼宇数字经济平台载体。规划建设数字经济总部基地，积极吸引数字经济龙头企业入驻并建设数字产业园区及总部楼宇。

　　2. 柳州市。依托河东片区、柳东新区、北部生态新区等重点区域，积极推进智慧物流、电子商务、科技创新等领域特色楼宇建设。加快工业化与信息化深度融合，支持采取“总部楼宇＋工业生产厂房”模式，建设一批科技研发、企业孵化、创意设计、金融服务、先进制造业商务服务、工业设计与软件服务等楼宇载体，打造新一代信息技术与制造业融合型楼宇数字经济集聚区。针对不同数字业态定制建设特色楼宇，推进现有楼宇内业态数字化发展。规划建设螺蛳粉产业园，推进融合“互联网＋螺蛳粉”上中下游产业信息数据的总部楼宇建设，依托楼宇建立柳州螺蛳粉产业大数据平台。

　　3. 钦州市。充分利用中国-东盟信息港钦州副中心建设发展机遇，围绕大数据、云计算、人工智能、北斗导航等产业培育，大力引进一批有关联的企业落户建设总部楼宇。大力推进智慧物流、跨境电商、游戏动漫、离岸服务外包、智慧医疗等以及大数据处理、物联网应用领域特色楼宇建设，积极构建楼宇数字经济创新生态产业链。充分利用西部陆海新通道、广西自贸试验区钦州港片区等建设机遇，重点推进物流金融、科技金融、航运金融等数字行业向特定楼宇集中或新建品牌楼宇，促进数字金融楼宇经济加速发展。

　　4. 北海市。支持中国-东盟信息港北海市副中心建设，依托海城区、银海区、北海工业园区、北海高新区等重点区域，推进智慧城市、智慧物流、智慧旅游、跨境电商、智慧医疗健康、数字金融、科技创新、数字文化创意、高端知识性外包服务、北斗导航应用等领域特色楼宇建设，带动临港创意数字化升级。围绕向海经济、高新技术、滨海旅游等，重点培育临海产业商务服务、电子信息制造服务、文化旅游和养老服务型楼宇数字经济集聚区。

　　（三）推动楼宇数字经济多点特色发展

　　1. 防城港市。依托防城港高新区、港口区等重点区域，推动互联网、大数据、人工智能与服务业深度融合，发展壮大数字经济新模式、新业态，重点推进通信服务、信息服务、智慧物流、跨境电商、科技创新、金融贸易、服务外包、智慧医疗等服务型楼宇数字经济发展。

　　2. 梧州市。以承接粤港澳服务业转移为重点，依托粤桂合作特别试验区、苍海新区等重点区域，积极引进物联网、云计算、大数据、北斗导航等产业项目，重点发展大数据清洗加工服务、金融服务、研发设计服务、商务咨询、健康医疗等服务型楼宇数字经济。

　　3. 贵港市。围绕打造以西江流域第一大港为核心的现代服务业集聚发展集中区，重点发展科技创新、电子商务、智慧物流以及“互联网＋航运”、粤港澳产业转移承接服务型楼宇数字经济。

　　4. 玉林市。围绕打造中药材、农产品、工业品商贸物流为核心的现代服务业集聚发展集中区，重点发展智能制造服务、“互联网＋现代农业”服务、中医药健康养生等服务型楼宇数字经济。

　　5. 崇左市。充分利用广西自贸试验区崇左片区、跨境电商综试区等国家战略平台，依托凭祥综合保税区等重点区域，积极推动建设跨境电商、智慧物流、数字金融、智慧文化旅游、科技创新等服务型楼宇数字经济，打造跨境数字经济合作示范区。

　　6. 百色市。依托百东新区等重点区域，规划建设数字经济产业园，积极引进智慧物流、电子商务、智慧文旅、云计算数据中心、数据资源开放平台等类型企业建设楼宇，创建数字产业的集聚集群发展。

　　7. 河池市。依托巴马等重点区域，充分利用大健康特色资源，重点发展5G技术应用、5G＋医疗健康、研学旅游、生命科学、健康科技、生命信息等领域大数据生态型产业楼宇。

　　8. 来宾市。围绕建设广西东部产业转移新高地，依托三江口港产城新区、来宾数字经济产业园，积极推动跨境电商、智慧物流、智慧旅游、数字金融、数字文化、智慧医疗、智慧康养、智慧教育等数字经济产业发展，积极推进软件设计外包、动漫创意外包、专业技术服务等新兴服务业发展，积极培育物联网、云计算、大数据、人工智能等新兴领域企业，积极引进数字经济企业入驻，发展楼宇经济。

　　9. 贺州市。依托广西数字贺州产业园-数字总部基地项目，积极推进科技创新、智慧教育、物联网产业、数据研发、互联网应用、软件产业等楼宇建设。推进新医药研究总部CBD以及智慧健康、智慧康养等楼宇建设。

专栏2 各市楼宇数字经济发展工程1. 桂林市。重点建设桂林华为生态信息产业园、桂林华为科技城、桂林花江智慧谷电子信息创业产业园、腾讯（桂林）众创空间、“桂林电商谷”园区、中辰电商物流园、EGO桂林（象山）数字经济港、桂林智能传媒谷、桂林经开区数字经济小镇等数字经济楼宇工程。2. 柳州市。重点建设大华数字科技园、滨江智谷、上海漕河泾柳东创新创业园、猪八戒网数字经济平台、柳州螺蛳粉产业大数据平台等数字经济楼宇（平台）工程。3. 钦州市。重点建设中国-东盟（钦州）华为云计算及大数据中心、钦州华为数字小镇、中马钦州产业园区北斗及遥感卫星应用创新创业基地、中国-马来西亚（钦州）产业园数据中心、颐高钦州数字经济产业园、钦州跨境电子商务综合试验区、中国-东盟富士康（钦州）医疗影像云中心等数字经济楼宇工程。4. 北海市。重点建设京东云（北海）云计算大数据中心、北部湾大数据及呼叫中心服务外包集群、北海新型智慧城市产业基地、中电北部湾信息港、北部湾东盟线上到线下（O2O）跨境电商产业园、敦煌网·广西跨境数字贸易总部基地、北海红树林现代金融产业城等数字经济楼宇工程。5. 防城港市。重点建设中国-东盟离岸信息服务外包产业园、桂海跨境电子商务快递物流园等数字经济楼宇工程。6. 梧州市。重点建设苍海数字经济产业园、京东（梧州）数字经济产业园、中兴梧州智慧广西云数据中心、梧州大数据产业基地、中国东盟健康医疗大数据应用中心等数字经济楼宇工程。7. 贵港市。重点建设贵港市八戒工场数字经济产业园区、西江科技产业城科技服务业集聚区等数字经济楼宇工程。8. 玉林市。重点建设玉林云计算大数据中心、玉林大数据生态产业园、玉林创客孵化基地等数字经济楼宇工程。9. 崇左市。重点建设凭祥口岸数字小镇、中国-东盟信息港，崇左大数据科创中心、中国-东盟（凭祥）电商产业园等数字经济楼宇工程。10. 百色市。重点建设浪潮桂西南（百色）云计算产业园、百色数字经济产业园区等数字经济楼宇工程。11. 河池市。重点建设中国-东盟（巴马）大数据云计算基地、中国-东盟（巴马）大健康大数据中心、河池巴马数字小镇等数字经济楼宇工程。12. 来宾市。重点建设来宾数字经济产业园等数字经济楼宇工程。13. 贺州市。重点建设广西数字贺州产业园-数字总部基地、广西贺州市东融怡亚通供应链建设、中生·穿越智慧浆云、盛源行大数据云计算中心。

　　四、推进楼宇经济数字化转型

　　坚持创新驱动，拓展融合深度、激发市场活力、开辟创新空间理念，依托商务楼宇、数字产业园，建设楼宇数字孪生空间、打造楼宇智慧服务体系、培育楼宇创新创业平台、创建楼宇虚拟产业集群，推进楼宇产业数字化转型升级，加快数据要素在企业间的流动，培育我区楼宇产业数字化新生态，提高转型效益。

　　（一）推进新型智慧楼宇数字化升级

　　1、加快智慧楼宇新型综合网络体系优化升级。实施部署全光网接入、楼宇CT云和业务云、5G宽带移动网、NB-IoT窄带物联网，实现无线网络泛在安全接入、泛在安全物联。

　　2、推动智慧楼宇管理支撑平台优化。优化楼宇视频监控系统，实现“看得见、看得清，快速处理”，提升入侵报警和门禁系统智能联动；升级无线识别技术提升考勤、自动出入效率；部署精确车位引导和寻车系统实现停车场高效管理；实施设备能耗监测构建敏捷控制，绿色节能型楼宇；提供资产跟踪管理，提高资产管理效率。

　　3、智慧楼宇应用支撑平台提升。探索楼宇前置部署办公云、统一通信平台，协助企业提升沟通和协作效率；全面升级物业管理系统和配套移动端，提供在线巡更、公共广播、融合会议、一卡通、智能指挥服务。

　　（二）建设楼宇数字孪生空间

　　1. 加快楼宇“新网络”建设。高度集成楼宇互联网技术、传感与控制技术、智能信息处理等信息技术，连接楼宇中物理设备设施、信息基础设施、社会基础设施和商业基础设施，构建安全、高速、泛在、智能的新一代楼宇信息网络，实现楼宇内部企业、集聚区楼宇间、楼宇与政务数据中心信息互联互通，打造协同调度能力强的数字楼宇。

　　2. 推动楼宇“新平台”建设。建设楼宇数据中心和信息化公共服务云平台，开发服务基础数据资源库，提升数据存储、数据收集与处理、数据安全等服务水平。建立楼宇数字经济行业信息共享系统，打通物业业主、入驻企业、物业服务公司、行业协会、政府之间的信息管道，促进供应链、人才流、资金流、信息流等多元信息归集共享，实现单个楼宇内、楼宇集聚区和城市楼宇信息共享。

　　3. 探索发展楼宇“数字孪生”。鼓励利用数字孪生技术、楼宇物理模型、传感器更新、运行历史等数据，在网络空间构建与物理世界相匹配的数字孪生楼宇集聚区和楼宇实体。探索建立完善的“感”、“传”、“知”、“决”楼宇大脑架构，鼓励开展楼宇工程数字化建模，实现对楼宇集聚区的辅助治理、运营和决策。

专栏3 重点商务楼宇和数字产业园区数字化提升工程鼓励重点楼宇和数字产业园数字化、智慧化提升，加快5G网络环境和基于人工智能、物联网、云计算、大数据等新一代信息技术的部署，楼宇监控与自治区公共安全视频图像信息共享总平台互联互通，一站式面向入驻企业提供城市公共服务、生活服务、楼宇食堂电子饭卡等。推动集信息服务及行政服务于一体的智能化楼宇集聚区建设。

　　（三）完善楼宇数字服务体系

　　1. 加快楼宇数字服务模式应用。鼓励有条件的楼宇探索创设智能化办公空间应用示范，基于物联网技术，部署智能办公、智能电力和智能办公家具系统，实现物理空间与多种智能软件链接，从楼宇安全性、便捷性及舒适性三方面打造楼宇智能服务平台。

　　2. 建立“监、管、控”一体化运维管理模式。统筹楼宇资产全生命周期环节，加强运维数据深度挖掘，实现高效巡检、快速维修、预防性维护以及全过程监管，提升运维保障能力，保障楼宇内各系统高效运转。

　　3. 拓展楼宇经济数字化运营场景。发展楼宇智能化办公、全天候线上物业服务、在线看房、线上租赁、线上招商、线上结算、线上营销、线上供应链管理等新型数字化服务模式。

　　4.拓宽楼宇数字服务体系的辐射面。统筹中小微企业办公经营场所，推动将零散的中小微企业纳入楼宇数字服务体系，提供与大型写字楼等一致的个性服务，助推中小微企业发展。

专栏4 楼宇智慧服务体系培育工程1. 楼宇智能运营系统。将管理、安防、设备管理等系统融为一体，将物理楼宇抽象为数字楼宇，通过数字楼宇打通互联网人的数据和物联网数据，形成新的管理与运营模式，对楼宇进行集中监管、运维管理等，通过数字化运营提升效能，实现各系统联动控制、协同处置；建设一批安全、舒适、便捷、智慧的楼宇，实现精细化管理，降低运维成本，提升管理效率，通过智能化场景，提升入驻企业及到访顾客体验。2. 打造一批楼宇数字经济共享平台。进一步完善单一窗口国际合作平台、广西“云商标”人工智能平台、南宁市跨境贸易电子商务综合服务平台、西部陆海新通道多式联运综合信息服务平台等，建立跨境金融数字平台、中国-东盟金融信息服务平台、中国-东盟跨境电商平台、中国-东盟大宗商品交易平台、中国-东盟“商贸通”数字平台、中国-东盟金融大数据服务平台、广西智慧养老数字平台、柳州螺蛳粉产业大数据平台等。

　　（四）培育楼宇创新创业平台

　　1. 积极探索“楼宇＋双创”新模式。充分发挥楼宇经济资源集约、创新集成、服务集中的优势，加大力度打造楼宇创新创业集聚示范平台。利用楼宇存量空间，开发联合办公空间，推动办公设施共享，面向中小型创新创业企业提供灵活的办公方式和完善的一站式服务，降低企业运营成本，助推创新创业企业发展。

　　2. 打造特色专业“创新创业孵化楼”。依托全区公共数据开放平台、数字广西创新创业工场等，引进、聚合、甄选、孵化一批数字经济创新创业企业。围绕数字经济发展重点产业和细分行业，打造“数字经济新业态办公＋个性商业服务”创新创业平台。

　　3. 打造多元素创新创业综合体。形成以高新技术研发、创新孵化、人才集聚为主导，集研究交流、教育培训、信息服务、展览展示、品牌营销、行业示范等延伸功能于一体，融合高端人才创新生态服务社区、休闲商业、商务及生活配套设施。

　　4. 打造科技创新综合体。 依托区内高校、科研院所，联合数字产业龙头企业、创投机构、政府高新技术主管部门，引入科技园4.0规划理念，创建“产学研”型楼宇科技创新中心。构建园区创新服务体系，通过产品设计、产业发展服务、智慧生态环境等措施，建设创新型科技园区，集聚创新资源，构建创新网络，强化全球创新资源链接，驱动园区企业集聚发展，推动园区产业转型升级。

专栏5 楼宇创新创业平台培育工程1. 构建全周期创新型楼宇共享办公空间。充分利用闲置的楼宇空间，以共享空间的方式，向自由职业者、初创企业和创业者提供办公空间服务。提供弹性的租赁周期，打造集创新型空间、高品质服务、多元化社群于一体的全新办公体验。引导整合碎片化的办公需求，提供基础设施完善，功能齐全的物理工作空间，打造线上线下功能相结合的资源平台，全方位满足企业办公需求。提供创业投资服务，含项目路演，孵化器合作，链接投资人等，大力支持创新创业企业的成长。2. 创建楼宇创新人才服务社区。立足于标准化的人才义务工作流程和管理制度，建立人才服务信息平台，建立以猎头服务、顾问咨询、培训共同组成的多层面、全方位高层次人才服务体系。建立健全楼宇人才管理制度，细化服务流程，拓宽“双创”人才引进渠道。加强人才网站、高级人才访寻、人才派遣等业务领域的合作，实现便捷的网上跨区域招聘求职服务，打通中高级人才跨区域引进的快速通道，增强人才聚集辐射能力。3. 培育楼宇众创空间和数字经济创新企业。支持有条件地市培育楼宇众创空间和数字经济创新企业，鼓励“创新创业孵化楼”向社会开放数据接口，采用“免费＋开放＋开源”方式，向小微企业、创客团队乃至东盟国家开发者提供低成本的数字产品创新平台。对优质项目给予奖励，对创新企业购房租房予以补贴，强化商务信息支持。4. 打造科技创新综合体。高标准布局业态，建立研发中心、众创空间、科技成果转化中心、科技企业孵化平台、科技智慧示范区、会议交流展示区等，打造具有产城融合、创新驱动、综合开放、智慧园区的全区科技综合体4.0标杆。

　　（五）创建楼宇虚拟产业集群

　　1. 加快创建虚拟产业园平台。依托商务楼宇和数字产业园，融合虚拟产业集群和飞地经济模式，创建与发达地区共建共享的虚拟产业园，引导产业平台化集聚，打造数字化产业集群新生态，实现产业供需调配和精准对接，推进产业高级化和产业链现代化。

　　2. 围绕重点领域培育虚拟产业集群。重点发展电子商务、软件行业、信息服务领域、文化创意行业、农业等领域虚拟产业集群，为入驻企业提供个性化、针对性的“一站式”政务服务和专业服务。

　　3. 加快完善虚拟产业园和虚拟产业集群保障体系。加快对虚拟产业集群的适用范围、中介支撑作用、风险分析、产业集群评估、与传统产业集群关联研究。完善虚拟产业集群政策法规，推动虚拟产业集群试点示范，加快虚拟产业专业人才培育，加快虚拟产业集群基础建设，支持虚拟产业集群发展。

专栏6 全区楼宇虚拟产业集群培育试点1. 电子商务虚拟产业集群培育试点。积极引进国内电商头部企业，打造具有集群集聚、互联共享、一站式服务等特色互联网电商虚拟产业园，吸引头部电商生态链企业入驻，以互联网为渠道销售产品、提供服务，加强产业链上下游之间的联动；打造“你创我帮”的众创平台，构建简单、安全、规范的低成本创业模式，为更多小微企业的创立与成长提供便利；统一托管入驻的创业者，规范和加强行业监管，营造公平有序的交易环境。2. 软件行业虚拟产业集群培育试点。积极引进国内软件行业的优质企业，吸引区内外软件生产商、软件供应商、互补产品生产商及其他企业入驻，创造实现跨区域协作的虚拟创新环境，提高产业生态链中各企业信息共享与创新合作效率；支持具有产业链、供应链带动能力的核心企业打造产业“数据中台”，为各企业资源、服务、技术等交流和对接创造有利环境，以信息流促进上下游、产供销协同联动，实现技术互补与资源共享，为企业发展赋能，保障产业链和供应链稳定发展，加快培育产业服务新生态。3. 农业领域虚拟产业集群培育试点。将地理位置上相近的农产品生产者、加工企业、物流企业及相关服务机构的个人或组织汇聚形成农业产业集群，以互联网为平台开展供应、销售及信息交流活动，实现农业产业链各环节在网络平台中的虚拟化运作，逐渐培育形成“农户-平台-公司”和“企业-平台-经销商”模式，有效弥补地域所造成的局限，打破农业产业链上下游之间各自孤立的现状，进一步提升农业产业共享经济效益。4. 制造业虚拟产业集群培育试点。根据《2020年全区制造业发展攻坚突破年实施方案》中我区重点发展的制造业关键领域，围绕电子信息产业、汽车产业、金属新材料产业、绿色化工新材料产业、精品碳酸钙产业、高端家具家居材料产业、生物医药产业等制造业产业，培育制造业虚拟产业集群。建立产业链培育机制，吸引产业链关联企业入驻，依托虚拟产业园平台精准对接产业链上中下游企业，对产业链链主企业和核心配套企业建立常态化服务机制，发挥链主企业领航作用，对产业链集成能力强、拉动作用大、年产值高的制造业企业予以重点支持，提高产业资金资助比例，鼓励金融机构优先支持，政府投资基金重点投资。5. 信息服务虚拟产业集群培育试点。以互联网创业公司为切入点，构建产学研一体化、培训实训孵化一条龙的信息服务业虚拟众创空间，推行“一址多照、住所托管”的集群注册模式，为区内信息服务产业提供各种代理记账、网站建设、商标专利、法律融资等增值服务，为企业提供创业、管理、营销、发展、上市等企业服务，降低中小企业或新创办企业发展成本；发挥集群集聚效应，形成闭环的服务生态圈，推进全区绿色营商环境和区域竞争优势建设，通过探索新型的区域产业经济生态模式，贯彻“互联网＋”精神及“共创共享”的理念，建立以互联网信息平台为支撑的、具有广西区域特色的集群共享产业园区。6. 文化创意虚拟产业集群培育试点。打破传统文创产业园的地域和容量限制，以通过宽松的政策环境、便利的方式和优质的信用服务，吸引外地优质文化创意企业“离岸注册”，打造文化创意虚拟产业园。为文化创意产业领域的创业者提供一站式服务，为需要虚拟注册模式、办公模式、托管模式的公司提供全方位的商务服务，包括异地企业工商注册、创意设计公共服务平台、文化创意微企孵化、创意设计标准化建设等；通过线上线下的各类培训、创业辅导、专题分享等活动，加快虚拟园区企业的提升和拓展，逐渐培育打造主导产业集群品牌。

　　五、加快数字经济楼宇化集聚

　　积极引导数字经济与楼宇经济融合发展，以加速培育专业和特色品牌数字经济楼宇、数字经济产业园、大数据产业园等为抓手，吸引数字产业企业入驻，加快构建楼宇数字经济生态圈，打造楼宇数字经济聚集区。

　　（一）培育数字经济新型楼宇

　　1. 加快培育数字经济专业楼宇。紧密结合数字产业化和产业数字化关键领域，围绕电子信息制造业、软件与信息技术服务业、信息通信业、制造业商服等产业，重点培育云计算、大数据、人工智能、北斗导航、区块链、跨境电商、智慧城市、智慧广电、网络文化、数字金融、智能制造、物联网等领域。培育数字经济楼宇、数字经济产业园、大数据产业园。围绕芯片产业及软硬件应用等行业融合应用，积极创建数字楼宇特色生态。

　　2. 重点打造新一代信息技术楼宇集群。积极搭建5G生态科技创新平台，建设高水平5G人才培养和孵化中心，积极促进5G与各行业的创新融合与应用推广，着力打造5G产业应用集聚区和融合应用示范区。全力发展“区块链＋”行业深度融合应用，引导发展区块链产业，支持区块链与数据交易、金融、物流、医疗、旅游、能源等行业应用企业，打造区块链国家级创新应用标杆。培育智能工业机器人、智能网联汽车和智能家居等产业，吸引数字文化产品小语种人工智能翻译中心、云端机器人运营中心、农业机器人等企业和产业项目入驻。创新拓展应用场景，依托数字经济特色楼宇，打造国家北斗开发推广应用试验基地和面向东盟的国际化北斗卫星应用产业。

　　（二）建设数字经济品牌楼宇

　　1. 积极推进现有楼宇转型升级。实施低效楼宇“退低高进”，开展楼宇二次招商、精准招商，推动发展一批数字化技术为支撑的总部经济、现代金融、科技创新、文化创意、中介服务、家居设计等领域特色楼宇，加快形成楼宇数字经济新增量。

　　2. 推进楼宇品牌化建设。建立以品牌楼宇为核心的整体性品牌架构体系，根据各楼宇的数字化软硬件基础设施、服务管理配套、发展定位与特色、入驻企业数量、经济贡献、税收贡献等指标，评选一批特色鲜明的自治区级品牌数字楼宇，有效提升数字楼宇品牌知名度。根据楼宇内企业在初创企业孵化、人才培养、平台服务等情况，评选自治区级优秀创新创业平台。推动自治区级品牌数字楼宇、创新平台作为城市名片，全面提升楼宇知名度。

专栏7 品牌数字楼宇建设工程1. 建设信息化创新型品牌楼宇。鼓励软件和信息服务业企业以及云计算、大数据、人工智能、5G、区块链、北斗、信创计算等新兴信息产业企业向楼宇集聚，积极培育智慧教育、智慧健康、智能制造、线上线下消费融合、共享经济、平台经济等新模式新业态，创建信息化创新型品牌楼宇。2. 建设电子商务品牌楼宇。推动行业大宗商品电子商务交易平台集聚发展，引导大型电子商务企业向定点楼宇集中，引进有实力的企业总部、互联网和电商创新创业孵化基地，建立电子商务服务体系，打造一批电子商务特色楼宇。3. 建设智慧物流品牌楼宇。加快建设智慧物流信息平台和仓储式物流平台，积极在城市楼宇推进智能仓储试点，推进智能物流装备、智能调度、智慧付费、无人搬运、智能码垛等新技术新产品应用，促进基于智慧交通路网的电商物流等服务业协同发展。支持建立智慧化共同配送分拨调配平台。4. 建设数字金融品牌楼宇。建设面向东盟金融开放门户核心区金融总部集聚区，鼓励发展金融信息管理、金融信息开发、金融信息中介服务、金融人才服务等业态，打造国际金融大厦、银行区域总部、保险区域总部、投资大厦、证券大厦、基金大厦、资管大厦等特色金融楼宇。5. 建设智慧旅游品牌楼宇。整合各类旅游资源，建立广西智慧旅游在线服务中心，打造全过程、一体化优质智慧旅游在线服务平台，打造“一部手机游广西”智慧旅游综合平台、“广西全域旅游直通车”分享经济平台。以北海为试点，依托北海银基项目、国际旅游服务中心、新营国际海洋文旅康养项目，打造北海智慧旅游品牌楼宇。6. 建设文化创意品牌楼宇。支持全区有条件城市发展特色楼宇文化，建设楼宇创意文化工场，建设都市楼宇文化艺术官、美术馆等线上线下一体化“楼宇智慧文化馆”，打造具有文化特色的楼宇地标。以楼宇为载体，依托广西浓郁壮乡文化、长寿文化和红色文化资源，利用VR、AR、3D等数字技术以及互联网平台，开拓创新性、体验性、互动性的文化服务与共享模式。7. 推进全区品牌楼宇评价和品牌楼宇示范。加强品牌标杆楼宇示范引领作用，出台“楼宇经济数字化标准体系”“楼宇数字经济集聚区评价体系”和“楼宇经济与数字经济融合新业态、新模式评价体系”，创建“城市楼宇城市名片”，培育一批数字化智慧楼宇、园区品牌，创建一批楼宇城市名片和楼宇创新平台，发挥品牌楼宇在数字化建设、产业链集聚、创新创业、服务体系等方面优势，提升全区品牌楼宇的示范效应。

　　（三）构建楼宇数字经济生态圈

　　1. 加快培育数字经济楼宇生态圈。积极推动数字经济主导产业向楼宇集聚，汇聚智能经济、流量经济、平台经济等新经济业态，吸引大数据、云计算、人工智能、数字贸易、软件开发等领军企业和细分行业创新企业入驻楼宇集聚区和示范区，加快商务、法务、财务、金融、教育等辅助行业企业向楼宇聚集，构建各具特色的闭合式数字产业生态链。

　　2. 大力开展楼宇产业链招商。推动各市由资源驱动向数字驱动转型，围绕优势产业、主导产业，推进产业数字化、数字经济楼宇化。着力打造资本、技术、人才等要素有机互融、高度互联的楼宇产业生态圈，提高楼宇产业关联性。加大力度吸引上下游关联产业项目入驻数字经济产业园，突出上下游企业和同类企业的集聚，发挥行业集聚和行业互补优势对楼宇发展的积极作用，推动形成具有广西特色、面向东盟的楼宇数字经济生态体系。以数字经济产业链入手，编制融合商务楼宇、数字产业园区分布、产业发展、产业招商、产业决策、产业展示、发展考核的楼宇数字产业“一张图”，明确产业链各环节招商思路和招商重点，围绕主攻方向开展重点招商和精准招商。

专栏8 数字经济楼宇生态圈培育工程1. 数字经济与楼宇融合产业基地试点。构建集科技研发、设计应用、技术服务、成果展示为一体的产业聚集型楼宇。积极引入国内外数字企业和生态伙伴，推动资源共享、业态融合、产业升级，打造中国-东盟数字经济产业发展新高地。2. 打造智慧商圈新商业模式。建设纳入“智能商务”、“智慧营销”、“智慧环境”、“智慧生活”、“智慧管理”、“智慧服务”、“智慧媒体”、“客流分析”的商圈智慧应用大平台，鼓励商圈对接主流电商平台，促进在线电商和在场实体商贸的融合创新发展。推进商圈信息基础设施升级，打造5G环境商圈、户外智能终端、高清摄像头、智能传感器等设备互联互通的智能物联网，实现商圈人流量、车流量、商品、服务等重要信息自动采集、实时共享，提升智慧商圈内商户精准感知消费需求的能力。推动建设智慧商圈公共服务平台，整合商圈及周边便民、公共设施信息和服务资源，为商户和市民提供免费上网、水电气缴费以及就业、社保、政务服务等各类便捷的生活服务。3. 构建智慧康养楼宇生态圈。建设健康养老大数据服务平台，深化数字技术在养老、保健、康复等领域的应用，培育形成一批新型智慧医疗服务企业，引导企业向特色楼宇和特色园区集中。依托数字技术优化康养建筑、社区、园区功能，鼓励发展医疗养老体验、智慧康养服务、老年金融保险、康养旅游文化、康复医疗护理、临终关怀服务等业态，构建全龄、全域、全时的康养服务体系，打造一批智慧康养楼宇生态圈。4. 构建远程教育楼宇生态圈。建立远程教育试点，推动优质数字教育资源建设和共享，构建形成智慧教育服务业生态圈。继续深入实施“宽带网络校校通”“优质资源班班通”“网络学习空间人人通”等校园信息化工程，支持与专业课程配套的虚拟仿真实训系统开发与应用，推广教学过程与生产过程实时互动的远程教学，促进全区教育服务个性化、公平化发展。5. 构建数字文化产业楼宇生态圈。充分利用5G、大数据、物联网、人工智能、区块链等技术，大力发展数字文创、游戏动漫、网络视听、高清视频等行业。等行业，聚集一批科技含量高、实力雄厚的企业，推动数字创意向制造、服务一体化转型，形成具有影响力的文化符号，推动产品价值与文化价值的良性循环。6. 构建新媒体经济楼宇生态圈。构建新媒体经济楼宇生态圈，充分借助5G网络、人工智能、虚拟现实、大数据等新技术，赋能文化创意、互联网广告、影视、直播、游戏、电竞等相关产业发展；丰富媒体产品、传播内容和用户体验，拓展新媒体产业边界，提供电子商务、电子政务、电子医务、电子教务等多种新型服务，深入经济、社会、文化各个层面，推动形成新媒体经济规模效应。

　　（四）打造楼宇数字经济集聚区

　　1. 推动数字经济领域相关行业向重点发展区域集中。优化全区数字经济产业布局，推进以楼宇为载体集聚数字产业，加强数字产业龙头招商，加快创新创业企业培育，强化重点特色楼宇培育，提升楼宇内优质企业服务和楼宇示范区公共服务，建成若干楼宇数字经济特色功能区。

　　2. 加快创建全区楼宇数字经济集聚区。中国-东盟信息港卫星应用产业示范基地、中国-东盟网络视听产业基地、广西数字贺州产业园、柳州市大数据产业园、北海数字经济创业园、钦州华为数字小镇、玉林大数据生态产业园、百色数字经济产业园区、贵港市数字经济产业园、来宾数字经济产业园、京东（北海）数字经济产业园、京东云（东盟）云计算大数据产业基地等一批楼宇数字经济集聚区，积极打造五象新区总部基地金融街（中国-东盟金融城）、中国-东盟电子商务产业园、南宁高新区中关村双创示范产业基地、青秀区数字经济产业园区、钦州5G智能终端制造产业园、中国-东盟信息港（北海）数字经济产业新城、中国-东盟北斗智能产业园、中国-东盟科技城等一批楼宇数字经济示范区。参照自治区现代服务业集聚区的推进模式，采取申报制方式每年评选自治区级楼宇数字经济集聚区，采取创建制方式每年评选自治区级楼宇数字经济示范区。

专栏9 楼宇数字经济示范区培育工程1. 打造“面向东盟的楼宇数字经济示范区”。大力引进领军型、创新型企业总部，形成一批商务、设计、研发、创新创业型楼宇，形成智慧经济楼宇群。提升楼宇载体品质，引导楼宇定向定制化开发，建设具有高品质、优秀承载能力的智慧型楼宇。重点围绕建设面向东盟的金融开放门户核心区要求，加快中国-东盟金融城建设，大力发展以数字金融为主题的楼宇。2. 打造“具有区域影响力的楼宇数字经济示范区”。以现代服务业、金融会展业楼宇为中心，重点打造现代服务业集聚区。进一步吸引大中型民营企业总部和跨国公司、国内大企业集团的区域性总部入驻，进一步加强各业态之间、楼宇经济与其他经济形态之间的联动机制，实现特色化、规模化和品牌化发展。3. 打造“科技创新楼宇示范区”。重点聚焦信息技术、智能制造、生命健康、科技服务业四大产业，打造配套设施齐全、物业服务专业的楼宇集聚区。发挥产业资源汇聚、科技创新引领作用，积极培育创新创业平台和创意产业园，优化创业创新设施和环境，集聚内外部优质资源，提供专业化、个性化服务。4. 打造“新一代信息技术产业与楼宇数字经济融合发展示范区”。构建以汽车电子产业、工业大数据、应用软件与信息服务为核心，新型显示器件和智能终端制造为延伸的电子信息制造业主要集聚地。加快个性化定制、网络化协同、服务型制造等新模式的发展，推动多种业态不断涌现。5. 打造“向海经济与楼宇数字经济融合发展示范区”。大力发展云计算、大数据、人工智能、互联网、物联网、平台经济、总部经济、流量经济、数字制造、数字金融、软件开发、芯片存储、数字创意等新一代信息技术产业，打造服务广西、辐射东盟、通融粤港澳大湾区的海洋经济楼宇集聚区。

　　六、夯实楼宇数字经济发展基础

　　（一）完善楼宇数字经济发展政策

　　加快出台全区楼宇数字经济发展政策指引，各市因地制宜制定专项楼宇数字经济发展政策。确定楼宇数字经济效益奖励标准，对税收贡献较大、企业自持物业、企业注册资本，重点服务企业和优质项目入驻等明确奖励标准；确定购房租房补贴标准，对企业入驻品质楼宇、楼宇业主改造提升楼宇品种、企业购（租）办公用房、企业员工购买自住房等设定补贴标准；制定楼宇扶持政策，注重引导楼宇业主改善软硬环境，评选示范楼宇、特色楼宇等，给予一定奖励，提高楼宇品位，形成示范带动效应。对企业主动投资改造楼宇公共设施、符合产业发展导向的予以补贴，对楼宇经营主体引进符合产业导向的产业集群，符合特色楼宇、重点商务楼宇认定等，给予奖励；提升政府在楼宇数字经济发展中的服务水平，包括服务角色、服务模式、服务手段，为楼宇企业、物业、业主提供便捷、个性化服务，充分发挥各设区市各职能部门、城区服务楼宇的基础作用，激发主动作为、服务楼宇数字经济发展的积极性。

专栏10 完善楼宇数字经济发展顶层设计1. 开展楼宇专项调查研究。展开重点区域的专项调研。重点把握楼宇数字经济发展基本情况、经济效益、发展规模、发展潜力，开专项研究，形成高质量楼宇数字经济研究报告。2. 加快编制楼宇数字经济发展实施方案。各市加快编制本地区楼宇数字经济发展实施方案，明确规划背景、范围、内容、期限、依据；楼宇载体平台、发展特点、空间资源分析、存在问题等；发展目标、发展路径，提出楼宇经济与数字经济融合发展新业态、新模式、新样本；空间布局部分包括空间布局总体框架、各城区楼宇业态布局等；各市在规划期内，为促进楼宇数字经济发展需重点完成的任务；各市、城区规划期内重点发展的特色楼宇及空间业态特点。

　　（二）创新机制激发楼宇数字经济活力

　　加快培育楼宇经济数字化专业运营企业，支持面向楼宇业主和入驻企业提供信息咨询、中介服务技术支持等专业化、个性化增值服务，推进对楼宇运营企业给予政策指导和扶持。加快推进楼宇开发、建设、招商、管理、运营、资产交易全生命管理机制建设，强化商务楼宇设备设施运营的集约化管理。构建楼宇入驻企业综合服务的全生命周期管理模式，引导有条件的商务楼宇开设楼宇企业全生命周期综合服务终端，提供从商务到政务的全生命周期楼宇企业服务，变“企业找服务”为“政府送服务”，为企业搭建资源交流平台。建立健全以银行、风投为主体的资金提供方，以园区企业和重大项目为代表的资金需求方，以证券公司、担保机构、评估机构为主体的金融服务第三方，以及楼宇发展促进会、楼宇经济行业协会为代表的政策与利益协调方的组织服务体系。

专栏11 创新楼宇数字经济示范区管理机制1. 创新楼宇数字经济示范区管理机制。建立“自治区-地级市-城区”三级楼宇数字经济发展联席会议制度，将楼宇数字经济示范区建设纳入重点改革任务；建立有序的政企合作机制，强化城区楼宇管理机构、物业公司与入驻企业协助配合；搭建服务平台，与各地商会、各产业协会或联盟、房地产及中介机构等专业协会积极开展合作，探索建立楼宇数字经济示范区商会、促进会，引导楼宇企业整合资源、产业聚合、抱团发展；提升楼宇数字经济信息网的大数据功能，为政府发展楼宇数字经济出台扶持政策、分析经济形势，为企业投资经营等提供数据支撑；提升公共服务覆盖面和质量，为楼宇企业提供工商税务、卫生健康、专业培训、党建及社区等一站式服务；形成具有各地特色的楼宇数字经济示范区文化。2. 开展楼宇全生命周期管理试点。鼓励全区有条件地级市创建全生命周期楼宇企业服务培育应用示范，在企业创业期，提供公司注册、银行开户、会计代理、社保代缴、许可证办理等服务；在成长期，提供品牌支撑、商标注册、项目申报、人事外包、融资服务、资质代办、体系认证、线上营销等服务；在发展期，提供专利申请、税务咨询、工商变更、法律咨询、上市咨询、品牌宣传等服务；在转型期，提供公司转让、公司注销、税收筹划、法律咨询等服务。3. 实施全区品牌数字楼宇推介工程。充分利用中国-东盟自贸区、中国-东盟博览会等平台，积极开展数字楼宇推介，创建“楼宇城市名片”、“楼宇创新平台”品牌、“数字楼宇名片”。

　　（三）加强楼宇数据资源汇聚共享

　　推动建立面向新经济形态的监测和统计模式。统筹考虑新经济形态发展特点，联合统计、工信、发展改革、税务、住建等职能部门，加紧研究构建全区楼宇数字经济评价统计指标体系，构建“自治区-地级市-城区”楼宇数字经济统计网络，形成楼宇数字经济政务、企业数据交换机制，形成楼宇数字经济数据共享机制，为其他新经济模式统计提供可复制的经验。依托广西数字政务一体化平台，搭建集信息采集、运营管理和政务服务功能于一体的全区楼宇经济监测和管理信息系统。各市根据权限更新、维护负责区域内楼宇数字经济数据接入全区楼宇经济监测和管理系统，提高楼宇的数字化管理和运营能力。

专栏12 楼宇经济数字化监测分析与管理体系建设1. 建设全区楼宇经济评价统计体系。全面采集全区各设区市楼宇基础数据，含楼宇基础数据指标、入驻企业基础数据指标、楼宇环境基础数据指标；楼宇经济基础数据，含楼宇总量指标、楼宇结构指标；楼宇经济行业划分标准等。从楼宇经济效益、楼宇发展规模、楼宇发展潜力等维度，对全区各设区市、重点城区楼宇数字经济发展水平进行科学评估，定期追踪全区楼宇经济发展变化，发布全区楼宇经济发展分析报告。2. 建设全区楼宇经济监测和管理信息系统。建设全区楼宇经济 “数字驾驶舱”，加强楼宇经济基础数据采集，建立楼宇资源信息库、楼宇资源数据分析；推动入驻企业基础数据采集，入驻企业基本信息、入驻企业经营信息、入驻企业就业信息；完善楼宇经济运行数据采集，楼宇税收情况、楼宇经济效益分析、楼宇经济产业发展评估、楼宇环境评估、楼宇运营管理、楼宇物业管理等；集成楼宇互动地图，实现商务楼宇的新建、改建、租金、待租房源、楼宇主题、业态等信息向公众分享。3. 构建全区楼宇资源及重点培育库。从各市楼宇资源信息库中，依据产业发展规划、产业发展导向和楼宇品质，择优评选自治区级、市级楼宇经济重点培育目标楼宇，并给予政策支持倾斜。4. 建立数字化楼宇标准评价体系。围绕绿色建筑、楼宇自动化系统集成、智慧物业管理、接入智慧城市等方面，构建数字化楼宇标准评价体系，实现数字化楼宇建设有标准可循。

　　（四）构建楼宇数字发展组织支撑

　　加强楼宇促进数字经济发展的产业规划、试点示范、资源对接、平台培育的支持力度。建立完善自治区与各市联动机制，共同推进核心技术研发、重大项目实施、产业示范建设、政策宣贯培训等重点工作。加强政企协同，充分发挥政府在引导行业发展、优化产业环境、保护知识产权、基础设施建设等方面的重要作用，充分发挥市场在资源配置中的决定性作用，加强技术攻关、应用探索、生态建设。持续促进数字经济与楼宇经济的协同，以更开放、更多元、更务实的方式，把各行业领域本身的技术优势、市场优势、资源优势、行业经验、专业知识与楼宇数字经济紧密结合，做优做强楼宇数字经济。

专栏13 楼宇数字发展的组织支撑1. 组建楼宇数字经济发展促进协会。负责推进楼宇经济发展服务、政策规范制定及产业培育工作，搭建政府与企业桥梁纽带，致力于楼宇经济规范、管理、服务，推动改善城市商业地产营商环境，促进楼宇数字经济发展。工作职责：搭建维护楼宇数字经济咨询专家库、提供会员单位专业化服务、搭建业内交流平台、加强楼宇人才建设、推进楼宇定位产业化、推进楼宇绿色化智慧化发展、推动数字楼宇生态圈建设、构筑广西楼宇数字经济生态圈、创建全区楼宇数字经济研究院、组织年度全区楼宇与数字经济融合发展高峰论坛、推动特色专业楼宇打造、推动“互联网＋楼宇＋入驻企业”和“互联网＋产业集团＋楼宇”形成楼宇经济生态圈等。2. 成立楼宇数字经济产业联盟。依托政府机构、科研院所、大专院校、科技企业等社会各界组织成立全区楼宇数字经济产业联盟，搭建产业发展平台，加强楼宇数字经济产业上下游的联系，为打造楼宇数字经济示范区和集聚区贡献力量，为促进经济社会发展与数字经济产业相协调做出贡献。3. 成立楼宇数字经济研究会。吸收和培养相关领域高水平人才，开展楼宇经济、数字经济等领域的创新性、战略性、前瞻性研究和重大关键技术的应用研究，为数字经济发展提供学术支撑；积极研讨，为推动数字经济发展献计献策；加强创新创造，推进研究成果转化应用。4. 召开楼宇与数字经济融合发展高峰论坛。定期开展全区楼宇与数字经济融合发展高峰论坛，邀请行业专家，通过对数字经济热点话题的探讨与研究，共同为全区楼宇数字经济发展寻找新方向、拓展新思路。

　　七、保障措施

　　（一）加强组织领导

　　充分利用数字广西建设领导小组在推进楼宇数字经济发展方面的促进作用，及时将楼宇数字经济发展推进过程中存在的重大问题上报领导小组予以解决。加强规划实施评估，建立规划动态更新机制。设区市建立由发改、大数据、住建、工商、投促、商务、税务等部门组成的联席会议制度，建立健全常态化沟通协调机制，根据本发展规划，制定全市楼宇数字经济发展行动计划、工作方案或实施方案并组织实施。针对不同类型数字产业楼宇，强化部门联动和分类指导，积极引导数字产业集群式发展，避免出现恶性竞争、无序发展等问题。

　　（二）强化政策支持

　　研究出台支持楼宇经济发展的政策措施，强化对楼宇经济数字化、数字经济楼宇化的扶持措施，推动楼宇经济高质量发展。加大对自治区级楼宇数字经济集聚区和示范区的支持力度，优先将其纳入自治区级现代服务业集聚区，享受后者相关支持政策。坚持“一区一策”“一楼一策”，支持相关设区市制定出台专项政策，在项目用地、运营管理等方面对数字经济楼宇项目予以支持。全力破解楼宇数字经济集聚区建设过程中遇到的瓶颈问题，积极支持各地市出台支持楼宇数字经济发展的政策措施，进一步放宽并允许符合数字经济发展方向的企业购买自持物业。

　　（三）加大资金扶持

　　加大资金支持力度，优化现有资金渠道，对获批的自治区级品牌数字楼字、楼宇数字经济集聚区和示范区予以奖励，各设区市可参照执行。对数字产业新行业新业态新模式、楼宇数字经济集聚区建设等的予以资金倾斜。积极通过以奖代补、贴息、股权投资等方式，支持新一代信息技术龙头企业设立分支机构或进行楼宇项目建设。推进对纳税贡献率高的楼宇业主予以一定奖励，引导楼宇业主优先吸引高税收、高附加值、高成长性的数字企业入驻。鼓励和支持政府平台公司与数字企业成立合资公司，主导进行楼宇项目开发。

　　（四）加强人才引育

　　围绕楼宇虚拟产业集群、楼宇智慧服务、楼宇创新创业平台、楼宇科技创新中心、楼宇数字化转型、楼宇产业数字化专业运营、楼宇招商引资等发展需求，大力引进经验丰富的高层次人才和团队。加强传统劳动力数字技能培训，提升数字化素养。引进一流高校协同推进楼宇数字经济发展促进协会、楼宇数字经济产业联盟、楼宇数字经济研究院建设，推动区内外高校、企业开展产学研合作，创建企业对口实践基地，培养数字经济与楼宇经济融合发展所需高层次复合型人才。实施“百千人才引进与培育”，引进一大批具有中高级职称的专业技术人才。

　　（五）优化服务保障

　　完善服务平台，建立全区楼宇数字共享服务平台和楼宇智慧服务平台，为楼宇业主和入驻企业提供政策咨询、工商、税务等综合性服务。加快楼宇的信息化管理，建立楼宇信息化管理平台、展示平台，实时掌握楼宇物业出售出租情况，为有意向进驻的企业提供全方位的楼宇信息。主动介入楼宇招商策划，定期汇总发布楼宇招商公告，积极组织围绕重点楼宇开展数字企业招商，提高楼宇招商的效率和质量。

　　（六）加强监督评估

　　制定特色数字楼宇的分类评定标准，强化楼宇的规范化管理。积极推进楼宇数据资源有效归集和有序汇集，建立楼宇信息平台，加强对全区楼宇建设及其运营情况的信息汇总，实现对全区楼宇整体层面以及重点领域的动态跟踪监测。从经济活力、科技创新、行业发展、空间效益、政策有效性等方面入手，建立楼宇数字经济统计调查和监测分析制度，构建楼宇数字经济发展监测分析体系，重点加强对品牌数字楼宇、楼宇数字经济集聚区和示范区等的数据统计和运营监测，定期发布楼宇数字经济发展评估报告。加大对楼宇数字经济发展的宣传报道，树立若干示范效应明显的数字经济标杆楼宇和楼宇集群典型。加强对规划落实的监督检查，对落实到位、成效突出的设区市给予通报表扬。

　　附件1：

名词解释

　　1. 数字经济：是继农业经济、工业经济之后的更高级经济阶段，是以数字化的知识和信息为关键生产要素，以数字技术为核心驱动力，以现代信息网络为重要载体，通过数字技术深度融合应用，不断提高传统产业数字化、网络化、智能化水平，加速重构经济发展方式与政府治理模式的新型经济形态。数字经济是现代化经济体系的重要组成，包括数字产业化和产业数字化。数字产业化，是数字经济基础部分，即信息产业，具体业态包括电子信息制造业、软件与信息技术服务业、信息通信业等；产业数字化，是数字经济融合部分，即传统产业由于应用数字技术所带来的产出增长和效率提升，具体业态包括以智能制造、工业互联网等为代表的工业融合新业态，以精准农业、农村电商等为代表的农业融合新业态，以移动支付、共享经济等为代表的服务业融合新业态。

　　2. 楼宇：《规划》所提“楼宇”特指商务楼宇和数字经济类产业园，其中商务楼宇参照国家标准《商务楼宇等级要求》（征求意见稿）编制说明（2019年），商务写字楼一般指在统一的物业管理下，以商务为主，由一种或数种单元办公平面组成的租赁办公建筑，基本功能是为商务办公活动提供场地设施及配套服务。数字经济类产业园特指，各类以大数据、云计算、数据中心、人工智能等围绕新一代信息技术相关产业建立的产业园区。

　　3. 楼宇经济：是以商务楼、功能性板块和区域性设施为主要载体，以开发、出租楼宇引进各种企业，从而引进税源，带动区域经济发展为目的，以体现集约型、高密度为特点的一种经济形态。主要表现为现代服务业，如金融业、咨询业、广告策划、影视制作、网络公司、律师事务所、会计事务所、咨询中介公司、高科技企业、娱乐服务企业、房地产开发企业、旅游服务企业、交通通讯企业等国内外各类企业和公司。

　　4. 数字楼宇：也称智能建筑、智能楼宇、智慧楼宇，是将建筑、通信、计算机和控制等各方面的先进科技相互融合，合理集成为最优化的整体，具有工程投资合理、设备高度自动化、信息管理科学、服务高效优质、使用灵活方便和环境安全舒适等特点，是能够适应信息化社会发展需求的现代化新型建筑。

　　5. 数字经济特色楼宇：主要是指以大数据、云计算、5G产业应用、人工智能、“区块链＋”、地理信息、数字文化、金融信息服务、数字金融、金融科技等数字经济领域为主要入驻业态的商务楼宇、数字经济产业园、大数据产业园等。

　　6. 数字经济品牌楼宇：根据各市楼宇的数字化软硬件基础设施、服务管理配套、发展定位与特色、入驻企业业态和数量、经济贡献、税收贡献等指标，评选出自治区级品牌数字楼宇。品牌数字楼宇主要方向包括信息化创新型品牌楼宇、电子商务品牌楼宇、智慧物流品牌楼宇、数字金融品牌楼宇、智慧旅游品牌楼宇、文化创意品牌楼宇等。

　　7. 数字经济示范区：在政府、市场的共同作用下，数字产业向特定区域集中而形成的数字经济集聚区。政府通过一系列改革创新举措，在推动数字经济集聚区发展过程中形成先行先试经验，即成为数字经济示范区。数字经济示范区既可以是工业数字化比较明显的工业园区，也可以是产业数字化、数字产业化比较突出的楼宇经济集聚区，还可以是以大数据产业等新兴信息产业为主要发展方向的数字小镇等。

　　8. 楼宇数字经济示范区：是楼宇经济集聚发展到一定阶段的产物，是数字经济示范区的一种类型，也是最主要的表现形态。其目的是以楼宇为载体，引导数字经济相关要素资源向楼宇集中。

　　9. 5A级写字楼：5A写字楼，是指实现智能化5A的写字楼 ，包括OA：办公自动化系统；CA：通讯自动化系统；FA：消防自动化系统；SA：安保自动化系统；BA：楼宇自动控制系统。

　　附件2：全区各设区市商务楼宇统计表

城市商务楼宇项目数（个）5A级写字楼（个）

南宁11520

柳州465

桂林364

北海383

钦州221

防城港322

贵港--

玉林290

梧州300

贺州150

来宾190

百色120

崇左210

河池120

　　数据来源：根据实地调研以及克而瑞房地产信息系统进行统计得到。

　　注：本表统计为2011-2019年全区各地级市商务楼宇建成项目。

　　附件3：全区各设区市5A级写字楼发展情况表

城市序号楼宇名称区位坐落智能化标准楼宇品质配套设施建筑规模入驻企业业态分布

南宁市1华润大厦南宁市东盟国际商务区核心区国际标准5A甲级写字楼广西“第一高楼”，现代化、高品质装潢；塔楼1-6层为办公大堂及酒店配套，地下室主要为设备机房、车库，裙楼主要用于商业周边为集购物中心、五星级酒店、高尚住宅等诸多功能于一体的大规模、综合性、现代化、高品质的标志性商业建筑群。交通便利，地铁直达。总建筑面积24万㎡（办公17万㎡），楼高403米金融证券、电子信息产业为主

2万达中心南宁市青秀区东葛路与滨湖路交会处200米超高度，国际5A写字楼集群1800平方米超大的整层面积、双回路供电、奢装挑高大堂、国际品牌电梯、中央空调新风系统等自身配套大型购物中心、高级商务酒店、奢华豪宅等；周边具备完善的政务配套总建筑面积约90万平方米，由多栋约200米的超高层建筑群组成主要为软件和信息技术服务业、互联网和相关服务业、保险业等

3九洲国际大厦南宁市青秀东盟国际商务区中新路与合作路交汇处318米超高层5A甲级写字楼南宁首席“地标”项目。项目地上71层，地下6层，为集超五星级酒店、商场、高档办公写字楼为一体的高档次、高规格的综合性超高层建筑。商业、银行、政务、旅游度假等配套齐全。占地面积约11万㎡，总建筑面积约21万㎡主要为金融服务业、软件和信息技术服务业，已有多家世界500强企业入驻

4三祺广场（交投大厦）南宁市青秀民族大道与中新路交汇处民族大道146号国际5A甲级写字楼集顶级写字楼与高端购物中心的综合性大厦、绿色建筑典范，总建筑高度达236米；国际品牌硬件配置中小学、综合商场、银行、医院、休闲度假等配套齐全总建筑面积近19万㎡商务服务业、零售业等

南宁市5总部基地-GIG国际金融资本中心南宁市五象新区宋厢路与云英路交汇处国际5A甲级写字楼建筑规模较大，质量一流；楼宇智能化设备齐全配套商业裙楼，地下五层停车场，以及公建设施、给排水、供电电讯、道路场地和景观绿化等配套设施总建筑面积约20万平方米，建筑高度199.95米银行、证券、基金、信贷、保险、评估、公证等多种金融产品及增值服务

6总部基地-市房产服务大厦南宁市五象新区总部基地，体强路以西，凯旋路以南，歌韵路以东国际5A甲级写字楼一类高层建筑楼内：办证大厅、房产交易大厅、房博馆、办公配套用房、多功能厅、档案馆、停车场总建筑面积6.8万平方米，建筑高度94米房产交易服务

7总部基地-绿地中心南宁市五象新区总部基地凯旋路15号国际5A甲级写字楼建筑规模大，入户大堂豪华宽敞地理位置优越，双地铁交通便捷；周边购物、生活、教育、银行、医疗配套齐全。总建筑面积约16万㎡金融服务业为主，世界500强企业中国平安、中国人寿财险以及中国名企开平住建等先后整栋购置

8总部基地-太平金融大厦南宁市五象新区总部基地“现代、高端、环保、智能化”5A甲级写字楼地下4层、裙楼5层；主楼层高3米净高，高于甲级办公2.8米标准；入户大堂豪华宽敞楼内：配备总部商务行政餐厅＋花园总建筑面积约10万㎡，楼高160米以区域金融合作、货币结算、离岸金融、期货交易、中小企业融资、产权交易为重点。

9南宁宝能环球金融中心南宁市五象大道西段665号（五象大道与飞龙路交汇处）国际5A甲级写字楼采用环幕景观幕墙拉阔全景视野，5.1米层高LOFT个性化空间设计；约6000个智能车位楼内：高速电梯、智慧物管、办公配套用房、多功能厅、档案馆、停车场、美食街等；自身配备15万㎡的国际商业购物中心；

周边自然景观、学区、生活、商业等大配套齐全；与地铁4号线无缝接驳。总建筑面积约86万㎡，高268米金融服务业及相关上下游产业

南宁市10富雅国际商务大厦南宁市五象新区庆歌路20号国际5A甲级写字楼建筑规模较大，质量一流；配有车位约1500个自身为集社区便民服务、医疗卫生、文化体育和社区商业为一体的新型城市综合体，2.5万平方米商业广场环绕，停车位充足总占地面积3万平米，总建筑面积19.5万平米主要计划引进“四新”经济企业

11龙光世纪南宁市东盟商务区中菲路以南超5A甲级写字楼造型极具现代化色彩，塔身玻璃幕墙和铝质装饰条，柔化室内光线，降低室内温度、同时减少建筑能耗；电梯速度最高到6米/秒；约16.4m广西最高大堂层高；智能闸机管理系统；自身为商业、办公、娱乐、文化等集众多功能集于一身的综合体，设置四层地下停车；毗邻商业中心、配套景区休闲用地；周边路网交通完善，出入南宁城区方便快捷。总建筑面积为39万平方米（办公面积约15万㎡），380米高零售业；软件和信息技术服务业；互联网和相关服务；电力、热力生产和供应业等

12和德-科创中心南宁市高新区新际路10号国际5A甲级写字楼集研发办公、创业成长、产业育成等于一体的创新功能区。园区路网、水、电、气等基础设施建设全覆盖。城市快环、外环及地铁1、2、3号线从园区穿过。拥有中高档住宅小区、高端人才公寓、职工宿舍；安吉万达、广西医科大学二附院、高新幼儿园、高新小学、中学等提供休闲娱乐及优质医疗、教育资源。总建筑面积约44万平方米，现可招商载体面积约为39万平方米。重点引进软件开发、软件技术、知名跨国公司、中央企业、行业龙头、平台经济领域企业等研发分支机构等相关行业的企业

13广源国际大厦写字楼南宁市青秀区竹溪大道86号5A甲级写字楼外设单元式玻璃景观幕墙；超五星级豪华入户大堂，近10米挑高；大堂大范围采用高档石材、智能系统、进口吊灯；配备电梯为日本原装进口三菱静音高速电梯，3-6米/秒；内置空中游泳池，分户式中央空调。前有南湖广场和会展中心，后有4A级风景区青秀山和青湖公园，是东盟商务区、五象新区和金湖广场三大CBD商圈交汇中心。车位配比1：2；交通便利，周边教育、医疗、银行、餐饮、酒店等配套齐全。建筑面积3.8万平方米零售业；软件和信息技术服务业；互联网和相关服务等

南宁市14绿地中央广场写字楼南宁市青秀东葛路161号（ 青秀万达对面 ）国际5A甲级写字楼摩天地标写字楼、城市中心综合体地理位置优越，交通便捷，涵盖289邕街沪巷派街区、“海珀·璞晖”高端住区、WE-WORK智慧LOFT集群、摩天地标写字楼打造，集住宅、商铺、公寓、写字楼等多业态国际精品于一体。宁绿地中央广场项目总建筑面积约103万平方米零售业；软件和信息技术服务业；互联网和相关服务等

15南宁万科大厦南宁市良庆区五象总部基地五象大道南侧金龙路2国际5A甲级写字楼绿色三星节能建筑，铝合金LOW-E中空双层玻璃；空调分户独立设计；单梯服务面积约为4500㎡/部；各类大中小型会议功能配置齐全；设置万科里商业、商务会议中心、泊时易智慧停车等功能车位配备达到100㎡/个；周边交通便利，教育、购物、娱乐、生活、餐饮等配套齐全总建筑面积17万平方米重点发展现代金融、科技创新、信息服务、总部经济等

16金投中心（招商中）南宁市民族大道180号330米超高层甲级写字楼采用世界TOP5的知名电梯、全智能机电设施设备，7大楼宇智能化系统：能源、停车、访客、会议、照明、电梯、消防，全面实现智能办公。位于民族大道城市主干道，地铁一号线凤岭站和琅东站分别位于两侧；位于东盟商务区及领事馆区核心区域，周边商务、商业、生活配套成熟而齐全，居住办公休闲娱乐一应俱全；裙楼为8层大型综合商业体；地下五层停车场，约1100个机动车停车位。主体楼高330米，共76层，总建筑面积29万㎡主要为金融业，广西金融投资集团已在项目购置20余层

17五象航洋城（未开放）南宁市五象新区五象大道401号国际5A甲级写字楼自身涵盖商务、商业功能核心功能、大型商务配套交通：直临五象大道，处于在建地铁3/4号线交汇处；总建筑面积41万㎡，高度超210m零售业

南宁市18海尔·青啤（东盟）联合广场（未开放）南宁市五象新区凯旋路9号国际5A写字楼南宁大型纯商务集群，集资本 、企业总部、产业基地、商务客厅、洲际旗下豪华五星级酒店于一体的37万㎡国际5A精端商务平台。北邻凯旋路、东邻飞龙路、西邻飞云路、南邻盘歌路，交通路网密集；靠近广西体育中心，广西规划馆，青少年活动中心，五象湖公园等设施，配套日趋完善齐全。建筑面积37万㎡节能环保、信息技术产业；

海尔和青啤集团面向东盟以及西南地区的资本管理中心、生产管理中心、销售管理中心和品牌推广中心

19中国-东盟信息港南宁五象远洋大数据产业园南宁市五象新区海晖路西侧、金良路北侧5A写字楼节能幕墙，满足建筑幕墙设计中的文化、节能、安全、采光等多种功能性楼内：IDC数据机房；周边在建建筑面积8.4万㎡计划大力引进物联网、5G网络、大数据云计算、人工智能等重点领域配套项目，以龙头企业带动形成“链主＋配套企业”的电子信息产业集群，为数字经济发展做好基础设施服务及数据存储保障

20中国-东盟地理信息与卫星应用产业园写字楼（在建）南宁市良庆区玉洞大道国际5A甲级写字楼建筑规模较大，质量一流涵盖标准写字楼、专家智慧公寓、会展中心、酒店和文化公园等，园区内将设置中国-东盟地理信息与卫星应用行业联络处、院士工作站、东盟总部基地总建筑面积约86万㎡卫星数据接收与应用、测绘遥感数据服务、导航定位服务、地理信息测绘产业

（武汉大学院士工作站、北京航天宏图信息技术股份有限公司等50家单位已签约。预计至2024年，产业园将引进地理信息全产业链260家单位）

柳州市1万达中心柳州市区东环大道与文兴路交汇处5A甲级写字楼配备全智能化办公系统、公共部分六星级精装、12部锋速电梯、国际顶级品牌中央空调及新风系统、双回路供电、五星级物管。本身为集购物、休闲、娱乐、居住、商务等多种功能于一体的大型城市综合体总建筑面积68万㎡科技、电子信息服务产业

2保利国际中心柳州市白沙路2号5A甲级写字楼大堂挑高11米，层高4.2米；智能商务系统、双回路供电、江水源热泵节能中央空调、智能进口电梯、网络连接楼体全信号覆盖、生态节能单元式玻璃幕墙交通、教育、医疗、银行、餐饮等配套齐全总建筑面积21万平方米（办公面积21万㎡）金融服务业、房地产开发经营、零售、餐饮业

3华润大厦柳州市文昌路17号5A甲级写字楼层高47层，标准层面积达1800㎡、层高3.9米，为柳州市金融创新示范楼宇商业配套：万象城、万达广场、五星乐和城等；

市政配套：柳州市政府、柳州市民服务中心、柳州市公安局、紫荆生态公园等；

酒店配套：亚朵酒店、丽笙酒店、优逸酒店、沃顿酒店、万达嘉华酒店等。总高202米，建筑面积约8.5万平方米金融业为主

4地王国际财富中心柳州市广场路北侧、八一路东侧原市人民医院地块5A甲级写字楼高达303米，为柳州市最高建筑。4000个配套停车位。自身为集办公、商务、酒店、居住、购物、文化、娱乐、社交、休闲、游憩于一体的城市综合体。总建筑面积64万㎡，总高303m，73层金融业、电子信息服务产业

5阳光100城市广场-中心写字楼柳州市文昌桥东，位于柳州未来CBD核心区域5A甲级写字楼可视彩显门禁系统，指纹识别密码入户门系统，全套防盗报警系统；商务智能化设施。自身集国际五星级酒店、5A写字楼，国际商业街、国际公寓于一体；周边有广场、超市、银行、餐饮、酒吧、影院、国际五星级酒店等配套。办公空间4.7万㎡零售业

桂林市1金贸中心桂林市 临桂区一院两馆正北面5A甲级写字楼写字楼大堂10.8m挑高；外立面采用LOW-E中空玻璃幕墙，全景采光，具有高透、隔热、隔音等特点；配有16部奥的斯电梯，时速达3.5m/s；智能化安防设置、5A智能化系统。写字楼车位高达2250个，配有国际化会议厅、会客厅、VIP宴会厅等。周边是市政府、国土大厦、建设大厦、发展大厦、环保大厦等行政区，与临桂“一院两馆”（桂林市图书馆、博物馆、大剧院）、1800亩中央公园一路之隔，区域商务气息浓厚。总建面约20万㎡，地上面积约15万㎡，地下面积约5万㎡（含2250个车位）金融业、高新技术产业等；华为、浪潮、中兴、比亚迪等名企已入驻。

2置地金融大厦桂林市秀峰区机场路与红岭路交叉路口5A甲级写字楼LOW-E镀膜双层中空钢化玻璃的全幕墙式写字楼；首层大堂层高8米，面积逾455㎡；打造桂林智能楼宇，为客户创造智慧消防、智慧通行、智慧电梯等智能场景。餐饮、娱乐、购物配套齐全，交通便利总占地223亩，总建筑面积约68万㎡招商中

3汇金1号写字楼桂林市临桂新区人民路与山水大道交汇处5A甲级写字楼大堂10米挑高，15部高端品牌电梯；外立面采用LOW-E双层中空玻璃幕墙；双层机械地下停车场集办公，居住，购物，餐饮，休闲，娱乐，金融，行政功能于一体；与市政府，中央水系公园，兴桂园等建筑遥相辉映，周围大型超市，医院林立。总建筑面积28万平方米，其中写字楼8万平方米主要为金融业

4耀辉·桂林国际商务中心桂林市机场路与西城大道交汇处5A甲级写字楼配合高品质石材和干挂工艺，采用高级玻璃幕墙为外立面；星级精装大堂；知名品牌电梯以及办公自动化系统、楼宇自动化系统等智能配备办公商务综合体，周边交通便利项目总建筑面积约4万平方米，总层数21层，楼体高度92.8米零售业、电子信息服务业等

桂林市5汇才大厦桂林市东二环路与桂磨路交叉口西北角5A甲级写字楼双层隔音LOW-E全玻璃幕墙；8米挑高大堂；3.3米层高；四部高速电梯，智能呼梯系统地处七星区核心区位，交通便利、生活配套齐全；周边有大型商圈，自身一、二层为商铺；品牌咖啡厅、餐厅、宴会厅、国际会议室等配套齐全。建筑面积约17000平方米招商中

北海市1文邦国际大厦北海市北海大道与四川路交汇处5A级国际商务写字楼外观主体结构采用铝合金全玻璃幕墙体系，幕墙玻璃选用高级中空LOW-E玻璃；一楼开放式顶级商务大堂、层层预留休闲大堂空间、商业顶层休闲绿化平台、商务中心、商务泛会所；负三层停车场，并且引入智能。停车优化系统、立体人车分流环线。政府机构、金融机构环伺周围，周边为北部湾商圈、大润华商圈两大商务中心；交通便利。总建筑面积约74378平方米招商中

2正五创新港写字楼北海市新世纪大道与云南路交汇处5A甲级写字楼现代化智能安防设备；充足停车位位于北海国家高新技术产业开发核心区，交通便利，周边生活、教育、医疗配套齐全；自身建设有科技企业孵化中心、科研开发中心、电商信息中心、技术及产品展示中心、文化创意中心、创投金融服务中心和众创空间等“七大中心”。占地2.5万平方米，建筑面积7万平方米以海洋生物医药、海洋环境保护、海洋生物制品、文化创意、电子商务、信息科技和金融服务等小微企业为主导，同时汇集北海各类自治区级以上的工程研究中心。

北海市3银河云谷里产业园写字楼北海市海城区昆明路11号（北海市高新区银河科技园）5A甲级写字楼外立面整体采用高端的LOW-E中空玻璃；写字楼户型方正无梁，8.4米开间，可灵活打造办公空间；充足停车位自身为集科技、商业、生活、文化、教育等为一体的智慧产城综合体，规划有业主食堂、书咖、大型超市、健身运动设施、商业街等；教育配套优势；出行便利。总建筑面积约9万平方米软件与信息服务业、文化创意产业、电力电子产业、海洋生物产业、生物医药产业、新能源新材料、电子商务、教育培训、全域旅游等

钦州市1华元·奥林财富中心河东新区子材东大街8号5A甲级写字楼5A级智能全玻璃幕墙写字楼、9米精装电梯大堂，分割体块方正；6台品牌电梯；地下二层停车周边商业氛围的相对成熟，交通便利；集餐饮、购物、娱乐、休闲等多种消费功能于一体零售业、电子信息服务业等，仍在招商

2光大天骄国际大厦

钦州市钦南区白石湖政务中心旁5A甲级写字楼智慧办公配套；4部三菱电梯；中央空调＋新风系统坐落于白石湖核芯地段，半小时通达全城；自身酒店、会议室、餐厅配套齐全；周边商业配套齐全约168米高、总建筑面积约56万㎡招商中

3北部湾中心（在建）钦州市扬帆大道与坭兴街交汇处5A甲级写字楼建筑外墙材料为蓝色玻璃幕墙＋白色金属构架＋白色穿孔金属板。2层地下车库以及3层临街商业等配套服务设施54层高度238米，总建筑面积18万㎡在建

防城港市1荣顾·翡翠国际防城港市港口江山大道中央大街5A甲级写字楼立面采用中空钢化玻璃幕墙；5A级写字楼办公配套设施，智能化电梯；顶级楼宇自动化；智能化监控、安防系统；地下立体式泊车；管家式物业服务办公、休闲、购物、娱乐、餐饮配套齐全；地处商圈核心位置，交通便利总建筑面积约12万平方米金融服务业

防城港市2三顺·世纪大都会防城港市中心区主干道北部湾大道与灵秀路交汇处5A甲级写字楼立面全玻璃幕墙装饰防城港市委、市政府、市人大、科技图书馆、文化艺术中心、博物馆、青少年活动中心、五星级酒店、大型超市、学校、高铁站、滨海湿地公园、白鹭公园等。总建筑面积约22万平方米招商中

3国门金茂大厦防城港东兴市5A甲级写字楼现代化外观、领先国际的装修理念配套有百业东兴红木社区、五星级商务中心、星级酒店、高端客户服务中心、高尔夫体验会、中国红木文化体验博物馆、红酒咖啡品鉴吧、沉香品鉴会所等，提供高端商业、物业专属服务总建筑面积约8.3万平方米金融服务业为主。其中1至3层为全国首个中国-东盟沿边跨境金融中心，届时将有国有四大银行、产权交易中心、东盟货币兑换中心、国海证券等等。

（33）江苏省政府办公厅关于深入推进数字经济发展的意见

（苏政办发〔2020〕71号）

各市、县（市、区）人民政府，省各委办厅局，省各直属单位：

　　数字经济是引领未来的新经济形态，发展数字经济是构建新发展格局的战略抉择，是推动高质量发展的必由之路。为深入贯彻落实党中央、国务院关于发展数字经济的决策部署，抢抓发展新机遇，培育壮大发展新动能，构筑发展新优势，经省人民政府同意，结合我省实际提出如下意见。

　　一、总体要求

　　以习近平新时代中国特色社会主义思想为指导，践行新发展理念，以供给侧结构性改革为主线，深入实施创新驱动发展战略，坚持创新引领、数据驱动、融合发展、共建共享、安全规范的发展原则，把数字产业化、产业数字化、数字化治理作为主攻方向，充分发挥数据作为关键生产要素的倍增效应，加快推动全要素数字化转型，围绕建设数字经济强省，着力实施数字设施升级、数字创新引领、数字产业融合、数字社会共享、数字监管治理、数字开放合作六大工程，全力打造具有世界影响力的数字技术创新高地、国际竞争力的数字产业发展高地、未来引领力的数字社会建设高地和全球吸引力的数字开放合作高地，为推动“强富美高”新江苏建设和高质量发展走在前列提供有力支撑。

　　二、主要任务

　　（一）数字设施升级工程。

　　1．加快建设信息基础设施。加强新一代通信网络基础设施建设，加快推进千兆光纤网络建设，扩大5G网络覆盖范围，推动南京国家互联网骨干直联点扩容升级和江苏互联网交换中心建设，进一步提升全省互联网IPv6发展水平，加快推进物联网发展，积极布局中低轨道卫星互联网。统筹建设数字算力基础设施，优化数据中心总体布局，强化数据中心的分类引导和集约利用，构建数据中心评价和监测体系，持续推进绿色数据中心建设，面向重点应用场景推进建设边缘计算节点，提升边缘节点的存储和快速响应能力，支持无锡、昆山国家级超算中心建设，探索构建边云超结合的计算服务体系。高标准布局新技术基础设施，聚焦人工智能等领域，完善面向全产业链的公共服务平台，加快发展安全可扩展的区块链基础设施，探索建设跨链平台。（省工业和信息化厅、省通信管理局、省委网信办、省发展改革委、省政务办、省科技厅等按职责分工负责）

　　2．全面升级传统基础设施。推动公路、铁路、水运、民航、水利、邮政等传统基础设施智能化升级，协同建设车联网、船联网等信息网络基础设施，推进平台互联、数据互通和设施共享，完善综合交通协同运营与管控，积极发展多式联运。推进能源与信息领域新技术深度融合，统筹能源与通信、交通等基础设施网络建设，构建“源－网－荷－储”协调发展、集成互补的能源互联网。推动市政基础设施智能化升级，加快新型智慧城市建设向基层延伸，推进县城智慧化改造，推动信息技术与农业农村生产生活基础设施融合发展。加快城乡网络一体化建设。（省交通运输厅、省工业和信息化厅、省发展改革委、省住房城乡建设厅、省农业农村厅、省水利厅、省广电局、省通信管理局，各设区市人民政府等按职责分工负责）

　　（二）数字创新引领工程。

　　1．释放数据资源新动能。推进公共数据资源开发利用国家试点，围绕重点领域，以需求迫切的业务应用为抓手，探索建立长效机制，推动公共数据资源安全有序开放、合理有效利用。引导社会数据资源价值提升，加速数据资源化、资产化、资本化进程，发展大数据产业，构建完备的产业体系，着力提升企业数据管理能力，提高数据资源质量，在重点领域开展数据管理能力成熟度评估，推进工业数据分类分级试点，持续开展大数据应用试点示范。研究数据要素市场运行机制，搭建基于区块链等技术的数据安全共享与开发平台、数据资源交易平台，探索建设数据交易中心，启动数据资本化试点。深化数据要素与传统生产要素的组合迭代、交叉融合，不断激发数据要素创新活力，促进传统产业加快转型和新产业新业态新模式加速涌现，加快构建以数据为关键要素的数字经济。（省工业和信息化厅、省政务办、省委网信办、省发展改革委等按职责分工负责）

　　2．增强关键核心技术创新能力。加快未来网络等重大科技基础设施建设，积极布局一批国家和省级重点实验室、产业创新中心、制造业创新中心、工程研究中心以及企业技术中心，打造多层次高效协同创新平台体系。聚焦核心电子器件、高端通用芯片、基础软件、工业软件以及大数据、云计算、人工智能、5G、区块链、高性能计算、未来网络、量子计算、网络安全等重点领域，汇聚优势创新资源，加快推动前沿基础型、应用型技术创新取得重大突破，推进核心技术自主化，补齐研发链短板，提升关键核心技术对产业发展支撑能力。（省科技厅、省发展改革委、省教育厅、省工业和信息化厅、省委网信办等按职责分工负责）

　　3．提升数字产业新能级。重点推进面向工业企业的软件、互联网和信息服务业创新发展，加大人工智能、AR/VR、工业机器人等前沿产业的高端软件研发和应用。强化电子信息产业优势，着力提高大数据＋、工业互联网、车联网、信息技术应用创新、工业软件、5G等重点领域产业链稳定性和竞争力，提升产业链水平，加快构建自主可控、安全可靠的现代产业体系。积极发挥龙头企业带动作用，加快培育创新型领军企业，重点扶持一批细分领域的瞪羚企业，培育形成一批具有国际影响力的专精特新小巨人和制造业单项冠军企业。支持有综合实力的平台企业跨地区、跨行业、跨所有制整合创新资源，形成一批具有全球影响力的自主创新企业品牌。调整优化大数据等各类产业园区，创建数字技术应用创新试验区，加快建设特色数字产业创新基地，打造具有核心竞争力的数字产业集群。（省工业和信息化厅、省科技厅、省发展改革委、省委网信办等按职责分工负责）

　　（三）数字产业融合工程。

　　1．推进制造业智能化转型。加快工业互联网创新发展，改造升级工业互联网内外网络，构建工业互联网标识解析体系，搭建行业级、企业级工业互联网平台，探索工业互联网大数据中心建设，打造工业互联网标杆工厂，深化工业互联网在先进制造业领域应用，加快制造业生产方式和企业形态根本性变革，提升制造业数字化、网络化、智能化发展水平。挖掘5G在制造业领域的典型应用场景，积极创建国家“5G＋工业互联网”融合应用先导区。以智能制造为主攻方向，加快攻克高端数控系统、伺服系统、精密传感及测量、智能加工等核心技术基础与关键部件，突破一批关键共性环节，提升高端智能装备自主研制水平，鼓励重点企业积极开展智能制造标准建设，聚焦13个先进制造业集群开展智能制造试点示范，围绕人工智能、智能装备、集成电路、工业互联网等领域部署建设省级制造业创新中心。进一步促进服务型制造发展，积极利用工业互联网等新一代信息技术赋能新制造、催生新服务，加快培育发展工业设计、共享制造、供应链管理、节能环保、检验检测认证、定制化服务以及全生命周期管理、总集成总承包、服务外包等新模式，加快供应链金融、智能仓储、快递物流、社交网络等生产性服务业发展。（省工业和信息化厅、省商务厅、省通信管理局等按职责分工负责）

　　2．发展数字化生活和服务业。加快商贸流通企业数字化应用和商业模式创新，支持电子商务平台企业做大做强，鼓励优势产业运用互联网发展垂直电商平台，完善电商生态服务体系，推动数字商务发展，大力发展跨境电商，拓展外贸发展新空间。整合全省文化和旅游资源，完善江苏智慧文旅平台内容功能，构建“一机游江苏、一图览文旅、一键管行业”的智慧文旅体系。推动数字科技与文旅产业深度融合，开发并推广新型优质的数字文旅产品，推动文化场馆和旅游景区打造数字化体验产品，丰富大众体验内容，推广“互联网＋文旅”新业态新模式。加强物流信息资源跨地区、跨行业互联共享，打造一批集交易、结算、跟踪、监管、服务于一体，具有行业和区域影响力的物流信息服务平台，支持数据驱动的车货匹配和运力优化等模式创新。推进交通基础设施全周期数字化管理，加强交通运行监测和交通大数据分析应用。推进自动驾驶在商用车领域率先应用，围绕自动驾驶和智能终端形成若干有影响力的产业。发展金融科技，支持苏州开展央行数字货币试点。加快智慧广电建设，推进超高清视频内容供给和传输覆盖，繁荣发展广播电视和网络视听产业。着力激活消费新市场，推进新零售发展，鼓励共享出行、餐饮外卖、团购、在线购药、共享住宿等领域产品智能化升级，发展在线教育、线上办公、远程医疗、直播电商等线上服务新模式，推动共享经济、平台经济健康有序发展，鼓励发展新个体经济。（省商务厅、省发展改革委、省文化和旅游厅、省交通运输厅、人民银行南京分行、省地方金融监管局、省广电局等按职责分工负责）

　　3．加快农业数字化融合发展。发挥南京国家农创园、南京国家农高区等农业科技创新园区示范带动作用，推动新一代信息技术与农业领域深度融合，建设一批省级数字农业农村应用基地。大力发展农业农村电子商务，建设农业农村电子商务示范基地和电子商务综合示范县，推进“互联网＋”农产品出村进城工程，完善农产品供应链体系、运营服务体系和支撑保障体系，形成乡村产业融合发展新格局。加快推进“苏农云”建设，打造全省农业农村“一张图”，形成“应用全打通、业务全融合、资源全调度”的“一云统揽”新体系，为产业发展、乡村治理、政务服务等提供重要支撑。大力发展休闲农业、创意农业、乡村旅游等新业态，跨界配置数字技术等产业要素，发展线上云游等新模式，做靓“苏韵乡情”品牌。支撑农业生产托管、农业产业联合体、农业创客空间等融合模式创新，探索基于数字化的新型生产经营组织形式。（省农业农村厅、省委网信办、省商务厅、省文化和旅游厅、省通信管理局等按职责分工负责）

　　（四）数字社会共享工程。

　　1．提升政务服务数字化水平。强化统筹协调，深入推进“互联网＋政务服务”，加快建设一体化政务服务平台，完善移动政务服务模式，加快实现“一网通办”，进一步打响“不见面审批（服务）”品牌。强化系统支撑，有序推进部门政务信息系统整合并与一体化政务服务平台对接，推进统一身份认证、电子印章、电子证照、公共支付等基础支撑体系建设、对接、应用。强化数据支撑，优化“1＋N＋13”大数据中心体系，建设完善基础数据库、主题数据库、部门数据仓，构建一体化数据共享交换平台体系，推进政务大数据创新应用。（省政务办负责）

　　2．深化民生领域数字化服务。深入推进智慧健康服务工程，加快省统筹全民健康信息平台、医院信息平台建设和信息互联互通，强化健康医疗大数据开发应用。推进国家健康医疗大数据中心与产业园试点，推广数字化医疗设备和可穿戴健康设备应用，提供定制化健康服务，积极发展互联网医疗。推进江苏“智慧医保”项目实施，建设全省统一入口的医疗保障公共服务云平台。大力推进智慧校园建设，加快完善覆盖全省各级各类学校的教育资源和信息管理公共服务平台，打造网络化、智能化、数字化、个性化、终身化的教育信息化公共服务体系，积极推进线上线下教育常态化融合发展新模式。全面推进城乡公交一体化调度系统建设，升级公路客运联网售票电子客票系统，推广“交通一卡通”NFC支付应用，完善交通出行综合信息服务体系，提供多方式融合衔接、按需响应、随需而行的高质量服务。加快推进省人力资源社会保障一体化信息平台建设，构建纵向省、市、县（市、区）、乡镇（街道）、村（社区）五级业务经办、行政审批和公共服务集中统一，横向各业务板块省集中系统的协同运行，纵横对接一体的人社服务新体系，形成所有经济主体、服务对象全轨迹、全周期、全生命、全画像的动态“大数据”。加强智慧民政建设，完善社会救助、慈善公益、社会组织综合服务平台，积极推进“智慧养老”发展。加快发展智慧体育，提升智慧体育公共服务能力。（省卫生健康委、省医保局、省教育厅、省体育局、省人力资源社会保障厅、省民政厅、省通信管理局等按职责分工负责）

　　（五）数字监管治理工程。

　　1．构建多方共治监管机制。坚持创新发展、包容审慎的治理原则，探索建立党委、政府、行业组织、互联网平台企业和公众等多元主体参与、有效协同的治理新模式新机制，完善社会监督举报机制。加强网络空间治理，全面推行网络实名制，建立社会化数据审查机制，加强个人信息安全保护。研究制定数据应用违规惩戒机制，加强对数据滥用、侵犯个人隐私等行为的管理和惩戒力度，建立相关风险事件的跨部门预警通报和联合处置机制。（省委网信办、省委宣传部、省工业和信息化厅、省发展改革委、省公安厅、省市场监管局、省通信管理局、省税务局、人民银行南京分行、江苏银保监局等按职责分工负责）

　　2．提升政府治理数字化水平。加快构建全省一体化监管平台，动态更新监管事项清单，将监管事项纳入部门监管业务系统运行，创新监管技术方式，将大数据、人工智能、5G、区块链等新技术运用于公共安全、社会治理、市场监管、生态保护、应急管理等领域各环节，推进非现场监管、移动电子执法和风险预警模型等现代化管理方式的应用。深度应用视觉智能、物联感知等先进技术，推进新一代雪亮技防工程建设。推动省市县各层级和各领域信用数据的纵横联通和分析挖掘，构建形成基于大数据的信用约束、协同监管、精准实施、分类扶持的新型监管机制。充分利用“大数据＋网格化＋铁脚板”工作模式，深化基层治理。（省委政法委、省政务办、省委网信办、省工业和信息化厅、省发展改革委、省公安厅、省市场监管局、省药监局、省生态环境厅、省保密局、省税务局、省通信管理局、人民银行南京分行、江苏银保监局等按职责分工负责）

　　3．压实互联网企业主体责任。强化互联网企业内部管理和安全保障。加强互联网行业自律，推动行业协会等社会组织发挥作用，出台行业服务规范和自律公约。强化社会监督，动员各方力量参与网络空间治理。完善互联网平台监管体系，组织开展检查、评议，引导、督促互联网企业落实主体责任。（省委网信办、省市场监管局、省工业和信息化厅、省公安厅、省通信管理局、省商务厅、省司法厅等按职责分工负责）

　　（六）数字开放合作工程。

　　1．推进省内区域协调发展。结合各地发展基础和条件，优化全省数字经济生产力布局，发挥比较优势，加强分类指导，实现错位发展。推动政务、医疗、交通、城市治理等多领域跨区域大数据应用协同，打造虚拟产业集群，深化苏锡常、宁镇扬等一体化先行区数字经济高质量联动发展，以点带面引领构建新动能主导经济社会发展新格局。（省发展改革委、省工业和信息化厅、省委网信办等按职责分工负责）

　　2．深度融入国家区域发展战略。积极融入“一带一路”建设、长江经济带发展、长三角一体化发展等国家战略，加强数字经济重点领域合作，探索区域数字经济发展新模式。发挥数字经济对推进长三角一体化高质量发展的引领支撑作用，促进数据要素跨区域流通共享，加快推进社保、就医、养老、旅游等方面率先实现“同城待遇”，深入开展大数据创新应用，打造长三角一体化数字经济产业生态，建设数字长三角。（省发展改革委、省工业和信息化厅等按职责分工负责）

　　3．提升国际交流合作水平。以举办国际性重大活动为契机，打造新型数字经济全球重要会展和高端对话平台。加强国际合作，积极参与“数字丝绸之路”建设，深化与沿线国家和地区在技术研发、标准制定、行业应用、人才培养等方面合作交流。（省商务厅、省发展改革委等按职责分工负责）

　　三、保障措施

　　（一）加强组织领导。

　　加强数字经济发展顶层设计，建立由省领导担任召集人的江苏省数字经济发展工作联席会议制度，统筹协调全省数字经济发展工作。联席会议办公室设在省发展改革委，主要职责是贯彻落实和督查督办联席会议议定事项，研究制定数字经济发展战略规划和政策举措。省联席会议各成员单位按照职责分工，制定落实推进数字经济发展的具体措施。各设区市要按照省联席会议架构，完善本地区推进数字经济发展的组织机制，结合实际制定本地区促进数字经济发展的实施方案。组建江苏省数字经济发展专家咨询委员会和数字经济研究智库，为数字经济发展战略研究、政府科学决策提供技术支撑和智力保障。（省发展改革委、省工业和信息化厅、省委网信办，各设区市等按职责分工负责）

　　（二）强化政策支撑。

　　落实对高新技术企业、中小微企业减税降费等各项扶持政策。优先保障列入省重大项目计划的数字经济领域重点项目建设用地，研究制定新型基础设施用电相关政策。加大省相关专项资金和基金对数字经济发展的支持力度，科学谋划和系统实施一批数字经济重大示范工程。支持符合条件的数字经济领域重点企业进入多层次资本市场融资。深化科技金融创新，健全完善机制体制，建立管理服务新模式，为科技型中小企业发展提供金融支持。加大对自主创新、安全可靠产品的政府采购力度。开展数字经济创新发展试点工作。创建一批数字经济示范园区、示范平台和示范企业，发挥示范引领效应。（省财政厅、省税务局、人民银行南京分行、省地方金融监管局、江苏银保监局、江苏证监局、省发展改革委、省委网信办、省科技厅、省工业和信息化厅等按职责分工负责）

　　（三）完善法规标准。

　　贯彻落实国家相关法律法规，梳理并修订与数字经济发展不相适应的政策法规，探索研究数据确权、隐私保护、数据安全、电子证照等重点领域地方立法，加强知识产权保护力度。加强基础共性标准和关键技术标准的研制，加快制定新型信息基础设施以及数据采集、开放、交易和安全等重点领域的相关标准规范。（省司法厅、省工业和信息化厅、省发展改革委、省委网信办、省公安厅、省通信管理局、省市场监管局、省政务办、省保密局等按职责分工负责）

　　（四）加大网络安全保障力度。

　　加大关键信息基础设施网络安全防护力度和可靠性保障，强化重点领域工业信息安全防护体系建设。建设网络安全态势实时预警系统，打造一体化网络安全态势感知云平台。建立省级部门网络安全情报协作机制，强化数据供应链、产业链威胁情报共享和应急处置联动。制定网络基础设施安全防护和应急处置预案。探索运用大数据、人工智能、区块链等新技术提高对数字经济风险的预知、预警和预置能力。强化数字基础设施相关供应链、产业链安全管理和可靠运行。加强政务信息化项目网络安全的全过程管理。（省委网信办、省工业和信息化厅、省公安厅、省通信管理局、省市场监管局、省保密局、省密码管理局、人民银行南京分行等按职责分工负责）

　　（五）加强人才队伍建设。

　　强化人才支撑，谋划制定数字人才发展规划。创新人才培养机制，依托产学研协作、高层次平台集聚等方式，培育一批领军人才和高水平创新团队。支持高等院校和职业院校开设适应数字经济发展的相关专业，加强校企联合实践培育，建设一批产教融合创新平台，培养高端技术技能人才。加大国际一流人才和科研团队的引进力度，创新人才引进政策和管理方式，推进江苏人才信息港建设，提升“互联网＋人才服务”水平。研究制定适应数字经济发展特点的就业制度、人才培训制度和社会保险经办服务方式。激发和保护企业家精神，鼓励更多社会主体投身数字经济创新创业。加强宣传引导，进一步提高全社会的数字素养。（省人才办、省教育厅、省人力资源社会保障厅、省发展改革委等按职责分工负责）

　　（六）加强统计监测。

　　积极参与数字经济分类国家标准研究和制定，加快研究构建符合江苏特点、反映数字经济发展变化的统计指标体系，加强数字经济发展运行情况统计监测分析。研究编制江苏数字经济发展报告。（省统计局、省发展改革委、省委网信办、省工业和信息化厅、省通信管理局等按职责分工负责）

江苏省人民政府办公厅

2020年10月8日

（34）内蒙古自治区人民政府关于推进数字经济发展的意见

（内政发〔2019〕23号）　　2019年12月31日

各盟行政公署、市人民政府，自治区各委、办、厅、局，各大企业、事业单位：

　　数字经济是以数据资源为重要生产要素，以现代信息网络为主要载体，以信息通信技术融合应用、全要素数字化转型为重要推动力，促进公平与效率更加统一的新经济形态。为深入贯彻党中央、国务院关于加快数字经济发展的战略部署，全面落实自治区党委和政府关于加快高质量发展的要求，结合自治区实际，现就推进全区数字经济发展提出如下意见。

　　一、总体要求

　　（一）指导思想。以习近平新时代中国特色社会主义思想为指导，深入贯彻党的十九大和十九届二中、三中、四中全会精神，认真贯彻落实习近平总书记对内蒙古工作的重要讲话重要指示批示精神，以“数字内蒙古”建设为核心，以“数字产业化”为推动，以“产业数字化”为主战场，以数字技术应用为重点，坚持创新引领、突出特色、政府推进、市场主导、多元共治、依法安全的发展原则，加快经济社会各领域数字化转型步伐，推动国家大数据综合试验区建设，促进数字经济深入发展，为自治区高质量发展提供有力支撑。

　　（二）主要目标。到2025年，全区数字基础设施进一步完善，产业融合创新取得重大进展，数字化治理能力有较大提高，数字化公共服务能力进一步增强，数字经济对国民经济发展的先导作用和推动作用得到有效发挥。

　　二、重点任务

　　（三）完善数字经济基础设施。

　　1. 完善信息基础设施。启动呼和浩特国家级互联网骨干直联点建设项目，确保2020年建成并投入使用，加快扩大互联网出区带宽。支持基础电信企业和内蒙古广播电视网络集团有限公司持续加大投入，普遍提供固定百兆宽带接入能力，加快固定宽带千兆网络建设，努力实现家庭、企业、园区、写字楼光纤宽带网络覆盖。大幅提升农村牧区网络设施水平，增加4G网络覆盖广度和深度。推动5G站址规划和基站建设，实现全区重点区域连续覆盖，加快通信网络设施IPv6改造升级和推广应用。开展面向物联网、无人机、无人驾驶等新技术新装备的区域性专用试验场地建设，加快物联网规划布局，加强“互联网＋教育”“互联网＋医疗”“互联网＋文旅”等数字应用基础设施建设，将通信基础设施纳入城市规划体系，推进市政公共信息基础设施全面开放共享。（自治区工业和信息化厅、内蒙古通信管理局牵头，自治区大数据发展管理局、内蒙古广播电视网络集团有限公司配合）

　　2. 积极打造算力中心。发挥自治区地质结构稳定、气候适宜、电力充足且价格便宜，以及靠近京津、毗邻八省、外接俄蒙便于信息流交汇的优势，继续支持中国移动、中国联通、中国电信、华为、阿里、腾讯、百度、苹果、亚马逊、曙光、浪潮、旷世科技、中兴能源等大型企业数据中心建设。积极推进和林格尔超算中心建设，鼓励开展云计算、边缘计算应用，打造呼包鄂、乌兰察布大型数据中心基地，加快建设国家政务云北方节点、北斗内蒙古分中心。建设绿色数据中心，降低能耗水平，充分开放数据中心算力空间，面向国内外、政府机关、企事业单位和社会提供应用承载、数据存储、容灾备份等算力和存储服务，着力将自治区建设成为支撑大数据、人工智能发展的国内外知名算力中心。（自治区发展改革委牵头，自治区工业和信息化厅、科技厅、大数据发展管理局配合）

　　3. 建设新型智慧城市。加快传感器技术、地理空间信息技术、卫星定位与导航技术、新一代信息网络技术在智慧城市建设中的推广应用。推进窄带物联网（NB-IOT）应用示范，积极推进公用设施、环卫设施、地下管网、电网等基础设施改造与5G信息网络、传感技术融合建设。统筹传感设备、无线通信设备、控制设备和摄像头等图像采集终端和感知终端在交通、给排水、能源、通信、环保、防灾、安全生产等城镇公共基础设施的布局，加快智慧社区建设及公共安全视频监控建设和联网应用，实现公共服务和管理基础设施的数字化、网络化。（自治区住房城乡建设厅牵头，自治区发展改革委、党委网信办、工业和信息化厅、大数据发展管理局、公安厅、自然资源厅、生态环境厅、应急厅、交通运输厅、能源局，内蒙古通信管理局配合）

　　（四）提升数字产业化水平。

　　1. 发展大数据核心产业。加快推进大数据产业集聚区和产业园建设，依托重点数据存储基地，带动数据清洗、数据集成、数据变换等数据预处理服务发展，壮大数据采集、存储管理、挖掘分析、安全保护及可视化等大数据服务产业。采用网络搜取、文本挖掘、自愿提供、有偿购买、传感采集等方式拓展政府数据采集渠道，推进政府机关、企事业单位的公共数据向社会开放，探索大数据交易，鼓励数据创新应用，深入挖掘大数据价值。（自治区工业和信息化厅牵头，自治区发展改革委、大数据发展管理局配合）

　　2. 做大做强大数据关联产业。以基地和园区为载体，积极开展针对性招商引资，引导大数据上下游产业加速聚集，形成产业链协同发展的格局。依托大数据中心，积极引进龙头企业，大力发展海量存储设备、高性能计算机、网络设备、智能终端、数据采集产品及大数据一体机等大数据硬件产品制造业，加快推进清华同方、浪潮等服务器生产线落地建设。培育软件设计产业，依托数据中心汇聚的海量数据资源，重点发展大型通用海量数据管理软件、数据分析挖掘软件、数据可视化软件、非结构化数据处理软件等软件产品。（自治区工业和信息化厅牵头，自治区发展改革委、科技厅、大数据发展管理局配合）

　　3. 培育前沿信息产业。加快引进区块链技术，推动区块链在证照办理、资质认定、资金流动、监督管理等场景的应用。积极开展语音图像识别、生物特征识别、自然语言理解、机器学习、深度学习等人工智能技术研究，支持工业机器人、服务机器人、智能联网汽车、智能家居等产业发展。（自治区工业和信息化厅牵头，自治区发展改革委、科技厅、大数据发展管理局配合）

　　（五）推进产业数字化转型。

　　1. 加快农牧业农村牧区数字化进程。建设数字乡村，实现数据宽带千兆进村、百兆入户，以乡村数字化支撑精准扶贫，助力打赢脱贫攻坚战。鼓励农牧业生产与大数据、物联网、空间信息、智能装备深度融合，全面采集农牧业气候、土壤等环境信息和农作物、畜禽生产及防疫信息，实现对生产全过程的实时监控、精准管理、远程控制、灾害预警、产品追溯和智能决策。加快发展农村牧区电子商务，加强与阿里巴巴、京东等电商企业的合作，促进农牧业生产与消费需求的高效匹配，重构农牧业农村牧区经济产业链、供应链、价值链。（自治区农牧厅牵头，自治区发展改革委、党委网信办、商务厅、大数据发展管理局，内蒙古气象局、内蒙古广播电视网络集团有限公司配合）

　　2. 发展工业互联网。支持制造企业、信息通信企业、互联网企业、电信运营商协同建立工业互联网平台，整合产品设计、生产工艺、设备运行、运营管理等数据资源，汇聚共享设计能力、生产能力、软件资源、知识模型、科研仪器设备及技术人才等资源，为用户提供设备健康维护、生产管理优化、协同设计制造、制造资源租用等应用。支持企业上云上平台，降低企业信息化一次性投入成本，引导企业运用工业互联网新技术、新模式提升数字化、网络化、智能化水平。（自治区工业和信息化厅牵头，内蒙古通信管理局、内蒙古广播电视网络集团有限公司、自治区大数据发展管理局配合）

　　3. 推动智能制造。以“数字化、网络化、智能化”为引领，支持能源、现代煤化工、钢铁、稀土、石墨新材料、硅材料、矿山机械、乳业、草业、蒙中医药、生物农药兽药、农畜产品加工等优势特色产业进行智能化改造。重点加强智能机床、智能传感器与控制设备、智能检测和智能物流与仓储设备以及工业机器人的研发与生产，建设智能生产单元、智慧车间与智慧工厂，打造集约高效、实时优化、安全可靠、绿色低碳的生产新体系。（自治区工业和信息化厅牵头，自治区发展改革委、大数据发展管理局配合）

　　4. 发展智慧旅游。完善景区通信基础设施，提升景区带宽，拓展文化旅游科技应用场景，强化旅游大数据支撑。建设智慧文旅小镇、智慧博物馆等设施，提升对游客“吃、住、行、游、购、娱”的服务能力。发展在线导游、导览、导购、交通导引、客流管理、质量投诉等网络旅游服务，推广3D景色欣赏、游客交流互动等虚拟旅游产品和服务。（自治区文化和旅游厅牵头，内蒙古广播电视网络集团有限公司、自治区大数据发展管理局配合）

　　5. 培育数字创意产业。依托自治区独具特色的民族文化、历史文化和地域文化，围绕动漫、游戏、影视、音乐、互动娱乐等领域，发展虚拟现实游戏、实景动漫、数字视频、微电影、交互式网络电视（IPTV）、专网手机电视和互联网电视、数字展览等产品和服务。加快融媒体发展，支持数字出版，鼓励主流媒体和龙头企业建立互联网传播平台，培育一批能够开拓创新、推动数字创意产业发展的市场主体。（自治区文化和旅游厅牵头，自治区广电局、新闻出版局、大数据发展管理局配合）

　　6. 积极发展新业态。有序发展共享单车、网约车等新业态，积极开发房屋短租、家政共享、分时租赁、无人零售等新服务，探索众筹、众创平台建设。充分利用互联网平台整合线上线下资源，促进供需高效对接，创新分享方式，提升社会资源配置和使用效率。（自治区交通运输厅、住房城乡建设厅、商务厅、科技厅按职责分工负责）

　　（六）加快智慧政务建设。依托全区电子政务外网推进政务信息资源整合共享，建设和完善人口、法人、空间地理、社会信用和电子证照库等基础数据库。健全数字要素资源管理体制机制，加强政府数据资源管理。强化自治区整体统筹、部门联动协同的“互联网＋政务服务”平台和“互联网＋监管”系统，构建线下办事大厅、线上政务门户、移动政务服务APP、政务服务热线等多元政务服务模式，有效形成自治区、盟市、旗县（市、区）、苏木乡镇（街道）、嘎查村（社区）五级政务服务体系，推动全区政务服务“一网通办”，实现更多政务服务事项“不见面审批”和“最多跑一次”，切实提升群众和企业的办事体验。（自治区政务服务局牵头，自治区发展改革委、党委网信办、工业和信息化厅、公安厅、自然资源厅、市场监管局、大数据发展管理局配合）

　　三、保障措施

　　（七）加强统筹协调。建立数字经济发展统筹协调机制，加强对全区数字经济发展的组织领导，研究促进数字经济发展的重大规划、政策，解决发展过程中出现的重大问题，完善评估评价机制，统筹各地区、各部门力量，形成全区上下协同促进数字经济发展的良好格局。（自治区发展改革委牵头，自治区工业和信息化厅、党委网信办配合）

　　（八）完善监管体系。加快推动清理修订不适应数字经济发展的相关地方性法规、规章及政策，完善市场准入交易规则等相关地方性法规、规章，探索数据立法。构建科学的数字经济统计指标体系和评估方法，制定政府数据采集、开放、共享、分类、质量等关键共性标准。建立与新业态发展相适应、包容创新的审慎监管方式，探索建立政府、互联网平台企业、行业组织和公众有效协同、多元共治的治理机制。提升对数字经济发展态势感知能力，强化对新技术应用的风险防范。落实互联网平台管理责任，引导互联网平台加强内部管理、安全保障和规范经营。（自治区党委网信办牵头，自治区工业和信息化厅、发展改革委、司法厅、市场监管局、统计局、政务服务局配合）

　　（九）加大政策支持。全面梳理已出台的大数据、云计算等相关支持政策，在对现有政策实施效果进行评估的基础上，结合发展新需求，以数据经济为统领，通过整合、修改、新增等方式，形成以大数据、云计算、区块链、人工智能、智慧服务、共享平台等数字产业化和产业数字化为支持方向，包含土地、资金、金融、创新等内容的数据经济扶持政策体系，促进生产力优化布局，引导数字经济健康高效发展。（自治区发展改革委、财政厅牵头，自治区工业和信息化厅、科技厅、大数据发展管理局、自然资源厅、地方金融监管局，人民银行呼和浩特中心支行、内蒙古银保监局、内蒙古税务局配合）

　　（十）强化安全保障。针对数据采集、存储、传输、共享和应用过程中的安全问题，加强云平台安全管理和网络空间实体身份管理。建立金融、能源、电力、通信、交通等领域关键信息基础设施产品与服务安全审查制度，提升对关键信息基础设施网络安全突发事件及数字安全事件的应急处置能力。定期开展信息安全检查、等级评测和风险评估，健全网络安全风险预警、情报共享、研判处置和应急协调机制，完善相关应急预案。（自治区党委网信办牵头，自治区工业和信息化厅、公安厅、应急厅配合）

　　（十一）夯实人才基础。将数字经济高层次人才纳入自治区急需紧缺高层次人才引进计划。鼓励区内企业、高校与国内外知名高校院所和企业开展合作，引进、培养数字经济学科带头人才、技术领军人才和高级管理人才。加快区内高校、培训机构培养数字经济领域专业技术人才步伐，积极开展各级各领域数字经济相关业务和职业技能培训，广泛开展创新创业活动，提升全民数字化素养和能力。（自治区人力资源社会保障厅牵头，自治区科技厅、教育厅、大数据发展管理局配合）

（十二）深化对外合作。加强与北京、上海、广东、浙江、福建、四川、重庆、贵州等数字经济发展先进地区的交流合作，针对自治区需求，精准对接和引进先进地区的产业、技术资源。积极参与“一带一路”数字经济合作，围绕大数据存储、分析、跨境电商、北斗导航、数据交易、支付结算等前沿技术开展形式多样的投资和服务合作。（自治区商务厅牵头，自治区发展改革委、工业和信息化厅、党委网信办、大数据发展管理局配合）

（35）山东省人民政府办公厅关于印发山东省支持数字经济发展的意见的通知

（鲁政办字〔2019〕124号）

各市人民政府，各县（市、区）人民政府，省政府各部门、各直属机构，各大企业，各高等院校：

　　《山东省支持数字经济发展的意见》已经省政府同意，现印发给你们，请认真贯彻实施。

山东省人民政府办公厅

2019年7月12日

**山东省支持数字经济发展的意见**

　　为深入贯彻落实《数字山东发展规划（2018-2022年）》，大力发展数字经济，加快推进新旧动能转换，实现高质量发展，制定本意见。

　　一、目标任务

　　（一）发展目标。以“数字产业化、产业数字化”为主线，以促进新一代信息技术与实体经济深度融合为重点，积极培育新产业新业态新模式，拓宽经济发展新空间。到2022年，数字经济与经济社会各领域融合的广度、深度显著增强，重要领域数字化转型率先完成，数字经济规模占全省地区生产总值比重年均提高2个百分点。

　　（二）重点任务。

　　1.提升数字产业化水平。发挥数据资源基础作用，推动关键技术研发，壮大产业规模，提升创新能力，优化产业结构，将数字产业打造成我省支柱产业。做大做强大数据、云计算、物联网等核心引领产业，超前布局人工智能、虚拟现实、区块链等前沿新兴产业，巩固发展集成电路、基础电子等关键基础产业，全面提升高性能计算机、高端软件、智能家居等特色优势产业。

　　2.发展特色高效数字农业。以打造乡村振兴齐鲁样板为引领，着力发展农业“新六产”，培育数字农业新动能。创新发展智慧农业，推进数字技术在农业生产、经营、管理和服务等环节的集成应用。推动智慧农机、智慧灌溉、智慧渔业、智慧种业、智慧畜牧工程建设，建设一批智慧农业应用基地。培育“互联网＋订单农业”，建立产销衔接服务平台，促进消费需求与农业生产高效匹配。大力发展农村电商，积极推动电子商务进农村综合示范工作。实施信息进村入户工程，加快益农信息社建设。

　　3.推动智能制造升级。大力推进我省现代优势产业集群＋人工智能，促进制造业数字化、网络化、智能化，实现传统制造向高端、绿色、服务转变。构建一批跨行业、跨领域工业互联网平台，推动工业互联网创新应用，加快企业上云。实施智能制造“1＋N”提升工程，大力发展智能制造装备和产品，支持工业机器人、核心工业软件、传感器等发展，加快智能工厂、智能车间建设。围绕新一代信息技术、高端装备、新能源新材料等十强产业，推进智能化、数字化技术及装备深度应用。

　　4.打造智慧服务示范区。推进数字技术与生产性服务业、生活性服务业融合渗透，扩大和升级信息消费。加快发展互联网金融、智慧物流、数字化设计等生产性服务业，促进生产服务体系专业化、高端化。发展智慧精品旅游、数字文化创意产业，提升生活智慧化服务能力和水平。支持发展平台经济、分享经济和体验经济，鼓励科技服务、商品交易、物流运输等领域专业平台建设。加大电商品牌培育，促进电商创新集聚发展。

　　5.加快培育数字化新业态。鼓励探索基于数字化的新型生产关系，推进数据赋能研发、生产、流通、服务、消费全价值链协同和融合应用。大力发展众包众创，培育推广大规模个性化定制、网络协同制造、远程运维服务等新模式。分级分类开展新型智慧城市、数字园区、数字社区建设，加大应用场景开放力度，鼓励更多数字新技术、新产品、新模式在智慧城市建设中应用落地。

　　二、政策措施

　　（一）加大要素供给。

　　1.优化数据资源供给。完善人口、法人单位、公共信用、宏观经济、空间地理和电子证照等基础数据库，分批建设主题数据库和通用业务数据库，健全“6＋N”一体化政务信息资源体系。依托山东公共数据开放网，持续推进政务数据和公共数据共享开放。瞄准社会热点需求，采取购买服务、合作共建等方式，鼓励企业和行业协会、研究机构等各类社会组织开展数据整合和应用。设立数据交易中心，建立健全数据产品评估、定价、交易等机制，不断优化数据流转服务。

　　2.升级基础设施。将信息基础设施建设纳入国土空间规划，组织编制信息基础设施专项规划，统筹考虑光纤网络、通信机房、管道、铁塔、基站等基础设施的布局。加快5G网络建设，按照国家有关规定，使用路灯、监控杆、机关事业单位办公楼等公共资源，在规划、用地、环评、用电等方面予以支持。鼓励各地开展新型智慧城市建设，全面升级信息基础设施，在数字山东专项资金中每年安排5000万元资金给予支持。引导产业园区加快构建工业无线网络和物联网环境。积极推动青岛国际通信业务出入口局申报和建设。

　　3.降低用电成本。对符合条件的各类数据中心、灾备中心、超算中心、通信基站等执行工商业及其他电价中的两部制电价。支持数据中心集约化、节能化建设，对符合规划布局，服务全省乃至全国的区域性、行业性数据中心，用电价格在每千瓦时0.65元的基础上减半，通过各级财政奖补等方式降至0.33元左右。根据实际用电量和产业带动作用，分级分档给予支持，其中数字山东专项资金每年补贴电费不超过3000万元。对于采用蓄能设备的5G基站，进一步降低低谷时段用电价格。对具备转供电条件的基站，纳入直接供电改造计划优先改造。我省大数据、互联网等高新技术电力用户参与电力市场化交易，不受电压等级和用电量限制。开通数字技术企业电力接入绿色通道，优先保障数字经济园区、企业和各类基础设施的电力接入。

　　4.保障建设用地。对纳入省重点建设项目的数字经济发展建设用地优先保障。属于下一代信息网络产业（通信设施除外）、新型信息技术服务、电子商务等经营服务项目，可按商服用途落实用地。数字技术企业所需工业用地的土地出让底价，在国家规定标准范围内可根据土地估价结果和产业政策综合确定。对数字技术企业，鼓励以租赁等多种方式供应土地，积极推行先租后让、租让结合供应土地。

　　（二）强化人才支撑。

　　1.多层次培养人才。对新设立博士后科研工作站的数字技术企业按规定给予招收补贴，对符合条件的入站博士（后）给予每人每年5万元生活补贴，最长补贴3年；出站留鲁企业工作并签订5年以上劳动合同的，按规定给予一次性补贴15万元。优先支持省属高校设置数字类相关专业，对新获批建设的数字类相关专业，由教育发展专项基金给予奖补。通过政府购买服务邀请国内外知名专业机构来我省开展数字经济相关培训。支持数字经济领域专业技术人员、经营管理人才申报非教育系统政府公派留学项目到国外访学研修。

　　2.高质量引进人才。支持数字经济领域企事业单位采取挂职兼职、技术咨询、周末工程师、特岗特聘等方式引进急需紧缺高层次人才。省泰山学者、泰山产业领军人才、“外专双百计划”等省级人才工程加大对数字经济领域人才和团队引进的支持。鼓励市县通过发放生活补贴等方式吸引数字经济领域专业人才。对在推介数字经济领域高层次人才来我省创新创业中起直接、关键作用的中介机构、社会组织和个人，经遴选后给予5-20万元奖励。

　　3.强化激励措施。支持数字经济领域高层次人才申报国务院政府特殊津贴等国家和省重点人才工程，培养选树一批“数字经济领军人才”。事业单位急需紧缺的数字专业人才，可实行年薪制、协议工资制等灵活分配方式，所需经费在绩效工资总量中单列，不作为单位绩效工资调控基数。

　　4.创新引智方式。优先支持数字技术企业在境外建立研发基地、开放实验室、科技孵化器、技术转移中心等离岸创新创业基地，视离岸人才引进使用数量和规模，经评审认定，分别给予500万元、400万元、300万元补贴。鼓励用人单位对作出突出贡献的离岸创新人才采取期权、股权和企业年金等方式进行激励。

　　（三）激发创新活力。

　　1.激励企业创新投入。对数字技术领域具有重大创新引领作用的国际和国家标准给予奖励。对获得中国专利金奖、银奖和优秀奖的数字技术企业，分别给予每个奖项奖补50万元、20万元、10万元；对获得省级及以上自然科学、技术发明、科学技术进步奖的数字技术企业，省财政一次性给予最高500万元奖励。引导企业建立研发准备金制度，对符合条件的数字技术企业按研发投入比例给予最高1000万元后补助。

　　2.支持创新平台建设。优先支持数字技术企业建设国家重点实验室、技术创新中心、产业创新中心等创新平台，新获批的给予最高3000万元贴息、奖补或股权投入。承担国家重点科研项目最高补贴1000万元。对列入省级重点支持的“个十百”产业互联网平台，省财政给予最高1000万元的贴息、奖补或股权投入；列入国家工业互联网平台的，提高到3000万元。

　　3.鼓励科技资源共享。对符合创新券条件的数字技术企业、团队使用共享科学仪器设备开展科技创新相关的检测、实验、分析等活动发生的费用，每年以创新券形式给予最高50万元补助。对提供服务量大、用户评价高、综合效益突出的供给企业，每年给予最高200万元补助。鼓励计量技术机构开放计量实验室。

　　4.促进成果转移转化。符合条件的数字经济相关创新产品纳入山东省政府首购创新产品目录。对促成数字技术成果在我省转化且年度技术合同成交额在1000万元以上的省级技术转移服务机构，省财政按合同成交额的一定比例给予最高50万元经费补助。

　　（四）培育市场主体。

　　1.支持企业做大做强。对首次进入全国电子信息百强、软件百强、互联网百强的企业，分别给予一次性奖补300万元。企业首次进入国家“独角兽企业”名单的，鼓励所在市政府采取“一事一议”方式给予支持。

　　2.鼓励各地招大引强。对总部（含研发总部和区域总部）新落户我省的全国电子信息百强、软件百强、互联网百强等数字技术企业，符合山东省商务厅等部门《关于印发〈山东省总部机构奖励政策实施办法〉的通知》（鲁商发〔2018〕2号）要求的，给予一次性奖补500万元。对数字经济领域年度实际使用外资金额超过5000万美元的新设项目和超过3000万美元的增资项目，符合山东省商务厅山东省财政厅《关于印发〈引进重大外资项目奖励政策实施细则〉的通知》（鲁商字〔2018〕222号）要求的，省财政按其当年实际使用外资金额不低于2%的比例最高奖励1亿元。

　　3.引导产业集聚发展。积极培育优势突出、辐射带动性强的大数据产业集聚区，对新确定的省级产业集聚区，在数字山东专项资金中给予不少于500万元的支持，对新认定的国家级大数据综合实验区或产业集聚区，给予最高3000万元的支持。对入驻数字经济园区的数字技术企业，由所在地政府对其办公用房和厂房给予不低于3年的房租补贴。

　　（五）加强资金扶持。

　　1.支持重大项目建设。大数据、云计算、物联网等重点领域项目，优先列入省新旧动能转换重大项目库和省重大科技创新工程，积极争取国家重大科技专项、科技支撑计划等专项资金支持。鼓励各地对数字经济领域重大项目采取“一事一议”方式确定扶持政策。

　　2.加大税费优惠力度。自2019年1月1日至2021年12月31日，对数字技术企业中的增值税小规模纳税人减半征收地方资源税、城市维护建设税、房产税、城镇土地使用税、印花税（不含证券交易印花税）、耕地占用税、教育费附加、地方教育附加。引导各地加快落实国家和省降费政策，进一步降低数字技术企业制度性成本。

　　3.引导社会资本投入。在省新旧动能转换基金框架下，设立大数据产业发展基金，引入天使投资、风险投资、创业投资、私募基金等优质社会资本。组织筛选适合基金股权投资的数字技术项目，优先纳入新一代信息技术等“十强”产业基金投资项目库。鼓励具备条件的市、县（市、区）结合地方实际设立区域性基金。除政府引导基金参股以外，对投资运作快、投资效益好的私募股权基金管理机构和团队，省金融创新发展引导资金按照相关规定给予单户最高30万元奖励。

　　4.加强金融信贷支持。鼓励各类金融机构针对数字技术企业特点，创新开展知识产权、股权、应收账款等质押贷款和银税互动贷款。对符合省政府有关政策规定的“人才贷”业务条件的高层次数字技术人才或其长期所在企业，在风险可控、商业可持续的前提下，由试点银行最高给予1000万元无抵押、无担保贷款额度，用于科技成果转化和创新创业。支持银行机构在数字经济集聚区，新设或改造分支行作为从事小微企业金融服务的科技支行，支持开展“创投＋银行”的外部投贷联动。鼓励融资担保公司降低数字技术企业担保费收取标准，对符合条件的融资担保公司给予最高100万元的补贴。

　　三、组织实施

　　（一）加强组织协调。数字山东建设专项小组负责统筹推进全省数字经济发展，各市、各有关部门要完善配套政策，形成联动机制。

　　（二）鼓励先行先试。对各地在数字经济发展中工作推进有力、发展成效显著的给予表彰奖励。对在服务数字经济工作中因先行先试出现的失误错误，按规定实施容错免责。

　　（三）推动融合发展。打造“政产学研金服用”共同体，有效聚集省内资源，连接整合国内外资源，优化提升创新创业生态。

　　（四）营造良好氛围。鼓励在我省组织召开国际或全国性的数字经济领域专业性会议或交流活动，根据规模、影响力等给予一次性补助。支持举办全国性、行业性大数据应用创新创业大赛，对获奖项目予以奖励。

（五）强化统计评价。建设山东省数字经济运行监测平台，研究建立数字经济发展水平评估指标体系，开展数字经济发展水平评价。

（36）安徽省经济和信息化厅、安徽省财政厅关于印发支持数字经济发展若干政策实施细则的通知 （皖经信推〔2019〕38号）

各市、直管县经信委（局）、财政局：

　　为贯彻落实《安徽省人民政府关于印发支持数字经济发展若干政策的通知》（皖政〔2018〕95号），省经济和信息化厅、省财政厅联合制定了《支持数字经济发展若干政策实施细则》，现印发给你们，请遵照执行。执行中如遇问题，请及时向省经济和信息化厅、省财政厅反馈。

附件：支持数字经济发展若干政策实施细则

安徽省经济和信息化厅

安徽省财政厅

2019年3月4日

**支持数字经济发展若干政策实施细则**

　　根据《安徽省人民政府关于印发支持数字经济发展若干政策的通知》（皖政〔2018〕95号）要求，按照程序规范、操作简便、权责明确、公正透明的原则，特制定以下实施细则。

　　第一部分　申报条件和材料

　　一、基本要求

　　（一）申报单位须为在安徽省境内登记注册并实际运营，具有独立法人资格的企业或机构。

　　（二）申报单位均需要提供以下基本材料：

　　1. 市（直管县）经信部门上报文件；

　　2. 申报单位的资金申请表；

　　3. 企业法人营业执照、税务登记证、组织机构代码证或事业单位法人证书副本复印件（如与营业执照三证合一，提供一证即可）；

　　4. 企业上一年度经审计的财务报告；

　　5. 申报单位法定代表人对申报材料真实性的承诺书，本人签名并加盖单位公章；

　　6. 文中申报材料要求企业提供有关设备购置、销售等发票的，企业需提供与申报项目有关的全部发票复印件；

　　（三）对荣誉类项目，原则上申报单位仅可对上一年度获得的国家级试点示范、百强企业等申请奖补。

　　（四）资金奖补要求

　　1. 单个企业的同一项目只可享受一项奖补；

　　2. 单个企业可叠加享受多项奖补，最高不超过500万元；

　　3. 企业已获得国家奖补的同一项目，不得重复申报。

　　二、支持数字技术创新

　　（一）奖补“互联网＋制造”国家级试点示范企业（项目）

　　奖补国家级制造业与互联网融合试点示范企业

　　1. 申报条件

　　获批工业和信息化部“制造业与互联网融合试点示范项目”的企业。

　　2. 申报材料

　　本细则要求的基本材料。

　　3. 支持方式

　　给予一次性奖补100万元。

　　奖补国家级工业互联网类试点示范企业

　　1. 申报条件

　　获批工业和信息化部“工业互联网试点示范项目”或“跨行业跨领域工业互联网平台”的企业。

　　2. 申报材料

　　本细则要求的基本材料。

　　3. 支持方式

　　给予一次性奖补100万元。

　　奖补大数据产业发展试点示范项目

　　1. 申报条件

　　获批工业和信息化部“大数据产业发展试点示范项目”的企业。

　　2. 申报材料

　　本细则要求的基本材料。

　　3. 支持方式

　　给予一次性奖补100万元。

　　奖补软件“铸魂”工程（项目）

　　1. 申报条件

　　获批工业和信息化部大型软件、操作系统、应用软件、信息消费等软件“铸魂”工程领域重点项目和示范项目的企业。

　　2. 申报材料

　　本细则要求的基本材料。

　　3. 支持方式

　　给予一次性奖补100万元。

　　奖补国家级智慧健康养老应用示范企业

　　1. 申报条件

　　进入工业和信息化部、民政部、国家卫生健康委员会“智慧健康养老应用试点示范名单”的企业。

　　2. 申报材料

　　本细则要求的基本材料。

　　3. 支持方式

　　给予一次性奖补100万元。

　　（二）奖补国家级人工智能与实体经济深度融合技术创新项目

　　1. 申报条件

　　获批工业和信息化部“人工智能与实体经济深度融合技术创新项目”的企业。

　　2. 申报材料

　　本细则要求的基本材料。

　　3. 支持方式

　　给予一次性奖补100万元。

　　（三）奖补优秀智能硬件产品

　　1. 申报条件

　　（1）申报产品为具有智能化功能的硬件产品，符合数字技术创新发展方向，有利于促进我省数字经济做大做强；

　　（2）申报产品在我省生产且年销售收入不低于 1000万元；

　　（3）申报产品国家有相关管理规定要求的，须符合相关要求；

　　（4）同一单位原则上每年申报产品数不超过3个。

　　2. 申报材料

　　（1）智能硬件产品政策奖补申请报告（包括企业基本情况及上年度生产经营情况，企业研发创新能力、获得专利、研发投入和研发团队情况，获得资质和荣誉情况，申报产品的功能、性能指标、同类产品比较优势、社会效益、市场占有情况等）及产品信息表；

　　（2）申报产品的研发、设计、制造等文档或手册；

　　（3）年度财务审计报告需包含申报产品年销售收入审计情况或者提供申报产品销售收入情况专项审计报告；

　　（4）证明申报产品技术创新水平相关材料（包括已获得的第三方认证或测试报告、自主知识产权证明材料等）；

　　（5）证明申报产品市场竞争力相关材料（包括市场占有情况，用户使用报告等）；

　　（6）申报产品实物照片（需包含产品主要外观、功能特征及铭牌信息等）；

　　（7）申报产品符合国家相关管理规定的证明材料（包括国家或省相关行业管理部门批准颁发的产品生产许可证、强制性产品认证等）

　　3. 支持方式

　　每年优选不超过50个产品，每个给予一次性奖补50万元。

　　（四）奖补优秀智能家居产品、成套智能家居系统

　　1. 申报条件

　　（1）申报智能家居产品、成套智能家居系统奖补的企业应拥有自主知识产权或是知识产权被许可人；

　　（2）智能家居产品是经具备国家规定资质的相关部门评定，奖补申报截止日期前评定有效，且在我省批量生产；

　　（3）成套智能家居系统是经具备国家规定资质的相关部门评定，奖补申报截止日期前评定有效，在我省生产，且该成套系统上一年度销售收入不低于3000万元。

　　2. 申报材料

　　（1）生产证明材料（直接接触食品的家用电器需提供生产许可证），智能家居产品、成套智能家居系统专项审计的财务报告；

　　（2）具备国家规定资质相关部门出具的有效证明文件等。

　　3. 支持方式

　　依据智能化程度、销售收入（产值）等对智能家居产品、成套智能家居系统进行综合评定，择优奖补。对智能家居产品给予一次性奖补，最高100万元；对成套智能家居系统的生产企业给予一次性奖补，最高100万元。

　　奖补资金实行总量控制，单个企业最高可获奖补300万元，单个企业集团最高可获奖补500万元。

　　三、加强市场主体培育

　　（五）奖补总部新落户我省的全国百强企业

　　总部（含研发总部和区域总部）新落户我省的全国百强企业--电子信息行业

　　1. 申报条件

　　（1）全国电子信息百强企业在我省新落户生产或研发等核心业务、或设立区域性总部；

　　（2）新落户总部在我省投资计划达到1亿元以上，且累计完成投资额达到计划投资的50%或累计完成投资额超过1亿元，上一年度对地方财政收入贡献（含政策减免税费）在200万元以上；

　　（3）申报单位为全国电子信息百强企业在我省注册企业，在安徽省内统一纳税入库。

　　2. 申报材料

　　（1）全国电子信息百强企业对在皖新落户总部（含研发总部和区域总部）的相关决策文件材料；

　　（2）全国电子信息百强企业在皖发展的业务发展报告、投资计划或战略规划等证明材料；

　　（3）企业上一年度新增主营业务收入、纳税及社保等证明材料；

　　（4）年度财务审计报告需包含新落户总部项目完成投资情况或提供项目完成投资情况专项审计报告；

　　（5）总部落户我省项目的备案或核准证明材料。

　　3. 支持方式

　　给予一次性奖补200万元。

　　总部（含研发总部和区域总部）新落户我省的全国百强企业--软件行业

　　1. 申报条件

　　（1）全国软件百强企业在我省新落户生产或研发等核心业务、或设立区域性总部；

　　（2）申报单位为全国软件百强企业在我省注册企业，在我省内统一纳税入库，上一年度对地方财政收入贡献（含政策减免税费）在200万元以上，或上一年度主营业务收入在2000万元以上；

　　（3）在省经信厅统计系统按时填报软件统计数据。

　　2. 申报材料

　　（1）全国软件百强企业对在我省新落户总部（含研发总部和区域总部）的相关决策文件材料；

　　（2）在我省新落户总部项目上一年度及已累计完成投资、上一年度新增主营业务收入、纳税及社保等证明材料；

　　（3）奖补资金申请报告，包括在我省设立总部基本情况（业务情况、股东情况、人员情况、办公情况等），及在皖投资计划、发展规划等，并附实有资金证明材料。

　　3. 支持方式

　　给予一次性奖补200万元。

　　总部（含研发总部和区域总部）新落户我省的全国百强企业--互联网行业

　　1. 申报条件

　　（1）全国互联网百强企业在安徽省内新注册成立的公司（含研发总部和区域总部性子公司），注册资本金在1000万元以上，并在安徽省内统一纳税入库；

　　（2）新落户企业上一年度对地方财政收入贡献（含政策减免税费）在200万元以上。

　　2. 申报材料

　　（1）中国互联网百强企业在皖设立总部的相关决策文本材料（董事会会议决议、建设方案及相关投资协议文件等证明材料）。

　　（2）在皖新落户总部项目上一年度及已累计完成投资、上一年度新增主营业务收入、纳税及社保等证明材料。

　　3. 支持方式

　　给予一次性奖补200万元。

　　（六）奖补首次进入全国百强的企业

　　首次进入全国百强的企业--电子信息行业

　　1. 申报条件

　　上一年度进入“中国电子信息百强企业”发布名单。

　　2. 申报材料

　　本细则要求的基本材料。

　　3. 支持方式

　　给予一次性奖补100万元。

　　首次进入全国百强的企业--软件行业

　　1. 申报条件

　　上一年度进入“中国软件业务收入前百家企业”发布名单。

　　2. 申报材料

　　本细则要求的基本材料。

　　3. 支持方式

　　给予一次性奖补100万元。

　　首次进入全国百强的企业--互联网行业

　　1. 申报条件

　　上一年度进入“中国互联网百强企业”发布名单。

　　2. 申报材料

　　本细则要求的基本材料。

　　3. 支持方式

　　给予一次性奖补100万元。

　　（七）奖补首次进入安徽省重点企业名单

　　首次进入安徽省重点企业名单--电子信息行业

　　1. 申报条件

　　依据《安徽省重点电子信息和软件企业管理办法（试行）》经省经信厅发布的安徽省重点电子信息企业。

　　2. 申报材料

　　企业申请奖补报告（包括企业基本情况，上一年度生产经营、经济效益及研发创新情况，产品创新性、市场占有率和经济带动性情况，企业发展战略规划及社会责任贡献情况，经审计的上一年度企业会计报表等）。

　　3. 支持方式

　　对首次进入安徽省重点电子信息企业名单的企业给予一次性奖补50万元。

　　首次进入安徽省重点企业名单--软件行业

　　1. 申报条件

　　依据《安徽省重点电子信息和软件企业管理办法（试行）》经省经信厅发布的安徽省重点软件企业。

　　2. 申报材料

　　企业申请奖补报告（包括企业基本情况、生产经营及技术研发创新情况，公司拥有的软件著作权登记证书、专利证书等，经审计的上一年度财务报告）。

　　3. 支持方式

　　对首次进入安徽省重点软件企业名单的企业给予一次性奖补50万元。

　　（八）奖励主营业务收入首次达到1亿元、10亿元的数字技术企业

　　1. 申报条件

　　（1）企业主要从事互联网、大数据、人工智能等新一代信息技术领域内的技术开发、转让、咨询、服务等；

　　（2）满足以下任一条件：

　　--企业上一年度主营业务收入首次达到1亿元，对地方财政收入贡献（含政策减免税费）在100万元以上；

　　--企业上一年度主营业务收入首次达到10亿元，对地方财政收入贡献（含政策减免税费）在500万元以上。

　　2. 申报材料

　　（1）企业近三年度经审计的财务报告；

　　（2）证明企业上一年度主营业务收入的大数额合同、发票。

　　3. 支持方式

　　对数字技术企业主营业务收入首次达到1亿元、10亿元的，分别给予一次性奖励100万元、500万元。

　　（九）奖补国家级制造业“双创”试点示范的企业

　　1. 申报条件

　　获批工业和信息化部“制造业‘双创’平台试点示范项目”的企业。

　　2. 申报材料

　　本细则要求的基本材料。

　　3. 支持方式

　　给予一次性奖补100万元。

　　（十）奖励“创客中国”“创响中国”大赛获奖项目

　　奖励“创客中国”安徽省创新创业大赛获奖项目

　　1. 申报条件

　　（1）本年度“创客中国”安徽省创新创业大赛一、二、三等奖获奖项目。

　　（2）企业类项目为省内注册企业。

　　（3）创客类项目自获奖正式文件发布之日后60天内，在省内完成企业注册。

　　2. 申报材料

　　本细则要求的基本材料（企业成立时间不满一年的获奖项目，无需提供上一年度经审计的财务报告）

　　3. 支持方式

　　对获得“创客中国”安徽省创新创业大赛一、二、三等奖的项目，每个分别奖补50万元、25万元、10万元。

　　奖励“创响中国”安徽省创新创业大赛获奖项目

　　按照“三重一创”政策关于奖励“创响中国”安徽创新创业大赛获奖项目要求执行。

　　四、大力培育数字经济平台

　　（十一）奖补优秀企业级、行业级工业互联网（云）平台

　　1. 申报条件

　　（1）申报单位应为制造行业重点骨干企业、互联网企业、信息技术企业、自动化企业等，具备较强的技术开发、项目实施、可持续发展能力以及良好的社会信用；

　　（2）申报平台能提供工业设备管理、软件应用管理、用户与开发者管理、数据与平台运营管理、存储和计算服务、应用开发服务、平台间调用服务、安全防护服务、新技术应用服务中至少一项服务。

　　2. 申报材料

　　（1）工业互联网（云）平台政策奖补申请报告（包括企业基本情况、资质与能力，工业互联网平台使用情况和应用效果、工业互联网平台技术架构及方案介绍等）；

　　（2）与申请项目相关的证明材料，包括专利/著作权证书、获奖证书、合作协议及其他申请报告中涉及到的证明材料；

　　（3）企业级工业互联网（云）平台需提供在企业内外网、平台、关键技术、生态体系等建设方面取得突破性业绩的证明材料；

　　（4）行业级工业互联网（云）平台需提供上一年度在皖开展工业互联网（云）业务收入情况的证明材料（经审计的财务报告、大额合同等能证明平台收入的材料）。

　　3.支持方式

　　根据工业互联网（云）平台的创新、效益等情况，每年优选不超过10个企业级工业互联网（云）平台，每个奖补50万元；优选不超过10个工业互联网（云）公共平台，每个奖补100万元。

　　（十二）奖补行业龙头和骨干企业基于互联网的“双创”平台

　　1. 申报条件

　　（1）申报单位具有较好的经济实力、技术研发和融合创新能力，是“双创”平台的运营主体；

　　（2）平台有较好的产业应用基础，在面向企业内部员工的各类创业创新资源分享，或面向全社会的制造资源和能力开放方面成效突出，有较广的发展前景和较强的带动作用；

　　（3）平台内创业项目年收入总计达到1亿元以上，且对平台主体企业所在地方财政收入贡献（含政策减免税费）不低于拟奖补金额。

　　2. 申报材料

　　（1）“双创”平台政策奖补申请报告（包括申报单位基本情况、资质与能力，平台项目实施情况及下一步实施计划，项目负责人与项目团队实力，产学研用联合协作情况，项目实施的创新性）；

　　（2）与申请项目相关的证明材料，包括各类资质证书（重点实验室或技术研发中心、国家新型工业化产业示范基地、国家级经济技术开发区、国家高新技术产业开发区等）、团队人员职称/专业证书、专利/著作权证书、获奖证书、合作协议（产学研用协议、合作单位协议）及其他申请报告中涉及到的证明材料；

　　（3）平台内创业项目收入证明材料（经审计的财务报告、合同等）。

　　3. 支持方式

　　对平台内创业项目年收入总计超过1亿元、5亿元、10亿元的平台主体企业，分别给予20万元、50万元、100万元的一次性奖补。奖补资金实行总量控制。

　　五、打造数字经济产业生态

　　（十三）奖补优秀工业APP

　　1. 申报条件

　　（1）企业在推进工业技术软件化方面有良好基础，相关工业互联网APP及应用解决方案具有成熟性和系统性；

　　（2）通过工业互联网APP的应用，帮助企业在研发设计、生产制造、运营维护和经营管理等环节取得了创新性经验；

　　（3）同一企业原则上每年申报工业APP数不超过3个。

　　2. 申报材料

　　（1）工业APP政策奖补申请报告（包括企业基本情况、资质与能力，工业互联网APP的知识产权归属情况、使用情况应用成效及取得的创新性经验）；

　　（2）与申请项目相关的证明材料，包括专利/著作权证书、获奖证书及其他申请报告中涉及到的证明材料；

　　（3）行业通用工业APP需提供上一年度销售收入情况的证明材料（经审计的财务报告、大额合同、财务单据等）。

　　3. 支持方式

　　根据工业APP的研发成本、效益等设置3类奖补档次，分别给予单个工业APP 30万元、20万元、10万元奖补。奖补资金实行总量控制。优先支持首版次APP。

　　（十四）奖补优秀信息化解决方案

　　奖补国家级工业互联网领域优秀解决方案

　　1. 申报条件

　　获批工业和信息化部工业互联网领域国家级优秀解决方案的企业。

　　2. 申报材料

　　本细则要求的基本材料。

　　3. 支持方式

　　给予一次性奖补100万元。

　　奖补省级工业互联网领域优秀解决方案

　　1. 申报条件

　　（1）申报单位应为从事工业互联网相关领域软、硬件装备和系统的集成设计、生产、安装、调试业务，并具备系统解决方案供应能力的产品供应商、服务提供商和系统集成商；

　　（2）申报方案必须为申报单位自主研发，且不存在任何知识产权纠纷；

　　（3）申报方案已得到实际应用。

　　2. 申报材料

　　（1）省级工业互联网奖补政策奖补申请报告（包括企业基本情况、资质与能力，系统解决方案简介、代表性及推广价值、应用情况及效果、产业合作模式、实际应用案例）；

　　（2）与申请项目相关的证明材料，包括专利/著作权证书、获奖证书及其他申请报告中涉及到的证明材料。

　　3. 支持方式

　　优选不超过20个优秀解决方案，每个给予一次性奖补50万元。

　　（十五）奖补数字经济创新共享服务联合体

　　1. 申报条件

　　（1）联合体成员应是我省内具备独立法人资格的企业、科研院所及高等院校，具有为企业数字化转型发展提供创意设计、研究开发、检验检测、标准信息、成果推广、创业孵化、跨界合作、展览展示、教育培训等服务的能力；

　　（2）每个联合体由不少于三个成员单位自愿组成，并确定一家企业为牵头单位，原则上三年内不得调整；

　　（3）联合体成员签订三年以上的合作协议，明确各自的责任和义务。

　　2. 申报材料

　　（1）联合体合作协议；

　　（2）联合体合作实施方案及各项服务开展情况（服务我省企业数量、服务等）；

　　（3）各联合体有关资质的证明材料；

　　（4）联合体服务业绩相关证明材料（财务报表、服务合同、发票等）。

　　3. 支持方式

　　根据联合体成员单位资质、联合体服务业绩、对地方财政贡献度等，每年优选不超过5个联合体，按其对地方财政收入贡献（含政策减免税费）进行一次性奖补，最高500万元。

　　六、完善信息基础设施

　　（十六）奖补“网效之星”企业

　　1. 申报条件

　　申报单位应是在加强新一代网络建设，推动工业互联的实践中取得明显成效的制造企业。

　　新一代网络建设包括：

　　--采用工业以太网、工业PON、工业无线、TSN、边缘计算等新型技术、设备改造生产现场网络和系统，实现企业内网IP（互联网协议）化、扁平化、柔性化；

　　--采用NB-IoT、eMTC、SDN、ICN等技术，实现多个厂区、工业智能产品、产业链伙伴等互联互通，优化销售、生产、运维、管理数据的采集和流转全过程；

　　--实施企业内、外网络的IPv6改造，实现工厂内基于IPv6网络的生产现场全流程数据采集分析、基于IPv6的工业智能装备/产品运维服务等；

　　明显成效是指实现网络互联、数据共享，在企业研发设计、降本增效、营销管理、节能环保等方面取得较好实际效果。

　　2. 申报材料

　　（1）“网效之星”政策奖补申请报告（包括企业基本情况、资质与能力，企业内外网建设、改造的方案及实施情况，应用成效及创新性经验）；

　　（2）与申请项目相关的证明材料，包括获奖证书及其他申报书中涉及到的证明材料；

　　（3）企业内外网改造资金投入的证明材料（经审计的财务报告、大额合同、财务单据等）。

　　3. 支持方式

　　根据企业内外网改造成本、效益等，每年优选不超过10家“网效之星”企业，每个给予一次性奖补50万元。

　　七、加强人才智力保障

　　（十七）奖补“数字经济领军企业”和“发展数字经济领军人物”

　　按照省政府有关规定开展本年度“数字经济领军企业”和“发展数字经济领军人物”评选工作。每年评选不超过10家“数字经济领军企业”，对获得“数字经济领军企业”称号的，给予一次性奖补100万元；每年评选不超过10名“发展数字经济领军人物”，对获得“发展数字经济领军人物”称号的，给予领导的团队一次性奖补50万元。

　　八、其他

　　其他现有奖补政策按相关政策规定执行。

　　第二部分　审核程序

　　一、下发通知

　　每年由省经济和信息化厅下发申报通知。

　　二、组织申报

　　由市（直管县）经信部门按照要求组织申报，会同财政等有关部门，上报申请文件。

　　三、组织评审

　　省经济和信息化厅组织专家或委托第三方机构对有关项目进行审核，并出具评审意见。

　　四、资金安排

　　省经济和信息化厅研究提出资金安排方案，经厅长办公会审定后，与省有关部门会商会签。

　　五、方案公示

　　资金安排方案确定后，向社会公示，并纳入安徽财政涉企项目资金管理信息系统进行项目比对。

　　六、资金下达

　　公示无异议及项目比对通过后，省经济和信息化厅下达奖补项目安排，行文商省财政厅下达资金。

　　第三部分　管理监督

　　一、项目单位对申报事项的真实性、合规性和资金使用负直接责任。

　　二、市、县、区等各级经信部门承担初审和对项目资金的日常管理监督责任，负责对上报项目进行把关，对弄虚作假者追究相关责任。

　　三、相关事项涉及的会计、审计等中介机构对其出具的报告的真实性、公正性负责。

　　四、省经济和信息化厅负责组织实施支持数字经济发展若干政策，负责开展项目的涉企系统比对核准及项目资金的稽查核查、绩效评价等工作，会同有关部门加强项目审核，避免重复支持。省财政厅负责完善涉企项目资金管理信息系统，协助对涉企项目进行比对。

五、在项目申报过程中，严格执行有关守信联合激励和失信联合惩戒相关规定。对弄虚作假骗取奖补资金，截留、挪用、转移或侵占奖补资金，擅自改变承诺实施事项等行为，停止拨付资金、限期收回已拨付的资金，同时将项目单位及中介机构列入失信信息推送至信用安徽平台，按照国家和省有关信用管理规定进行联合惩戒。对审核把关不严、项目出现问题较多的市（直管县）主管部门，根据情节和实际情况，予以通报批评。对触犯刑法的单位和个人，依法移送司法机关追究其刑事责任。本实施细则自公布之日起执行，由省经济和信息化厅负责解释。

（37）安徽省人民政府关于印发支持数字经济发展若干政策的通知

（皖政〔2018〕95号）

各市、县人民政府，省政府各部门、各直属机构：

　　现将《支持数字经济发展若干政策》印发给你们，请认真贯彻执行。

安徽省人民政府

2018年10月23日

**支持数字经济发展若干政策**

　　为深入贯彻国家数字经济发展战略及省委、省政府加快建设“数字江淮”决策部署，大力发展数字经济，推进数字产业化、产业数字化，构建现代化经济体系，实施以下政策。

　　一、支持数字技术创新

　　支持建设工业互联网创新中心。有效整合高校、科研院所、企业创新资源，围绕重大共性需求和重点行业需要，开展工业互联网产学研协同创新，促进技术创新成果产业化。鼓励各市政府对创新中心建设用地、办公场所、人才引进等方面予以优先保障。（牵头责任单位：省经济和信息化部门，配合单位：省科技、财政等部门，各市人民政府）

　　培育“互联网＋制造”示范。对制造业与互联网融合、工业互联网、大数据产业、软件“铸魂”工程等领域的国家级试点示范企业（项目）给予一次性奖补，最高100万元。（牵头责任单位：省经济和信息化部门，配合单位：省财政等部门）

　　促进人工智能技术创新。对获得国家级人工智能与实体经济深度融合创新项目的，给予一次性奖补100万元。支持智能硬件产品生产，对技术创新水平较高、市场竞争力突出、在我省生产且年销售收入不低于1000万元的智能硬件产品，每年优选不超过50个给予一次性奖补，每个最高50万元。对通过国家智能家用电器标准评定并在我省批量生产的智能家居产品给予一次性奖补，最高100万元。对在我省生产成套智能家居系统且该系统年销售收入不低于3000万元的企业给予一次性奖补，最高100万元。（牵头责任单位：省经济和信息化、发展改革部门，配合单位：省财政等部门）

　　加快数字技术标准研究。对主导制定国际、国家（行业）相关数字技术标准并取得实效的企业，分别给予每个标准一次性奖补100万元、50万元。（牵头责任单位：省经济和信息化部门，配合单位：省财政、质监等部门）

　　二、加强市场主体培育

　　培育和引进骨干企业。对总部（含研发总部和区域总部）新落户我省的全国电子信息百强、软件百强、互联网百强企业，每户给予一次性奖补200万元。对首次进入全国电子信息百强、软件百强、互联网百强的企业，分别给予一次性奖补100万元。对首次进入安徽省重点电子信息、软件企业名单的企业，分别给予一次性奖补50万元。（牵头责任单位：省经济和信息化部门，配合单位：省发展改革、财政、工商等部门，各市、县人民政府）

　　支持企业做大做强。对我省数字技术企业营业收入首次达到1亿元、10亿元的，分别给予一次性奖补100万元、500万元。企业首次进入国家“独角兽企业”名单的，鼓励所在市政府采取“一事一议”方式给予支持。（牵头责任单位：省经济和信息化、发展改革部门，有关市人民政府，配合单位：省财政等部门，安徽证监局）

　　催生“双创”新主体。对获得国家级制造业“双创”试点示范的企业，给予一次性奖补100万元。对获得“创客中国”“创响中国”安徽省创新创业大赛一、二、三等奖的项目，每个分别奖补50万元、25万元、10万元。（牵头责任单位：省经济和信息化、发展改革部门，配合单位：省财政、人力资源社会保障等部门）

　　三、大力培育数字经济平台

　　推动“皖企登云”。鼓励企业突破数据集成、平台管理、开发工具、微服务框架、建模分析等关键技术，建设工业互联网（云）平台。每年优选一批企业级工业互联网（云）平台，每个奖补50万元；优选一批工业互联网（云）公共平台，每个奖补100万元。建立动态管理的工业互联网（云）服务资源库目录，对优秀服务商予以重点宣传推荐。（牵头责任单位：省经济和信息化部门，配合单位：省财政等部门）支持各类企业和创业者以工业互联网（云）平台为基础，利用大数据、物联网、人工智能、区块链等新技术，积极培育平台经济、分享经济等新业态、新模式。（牵头责任单位：省经济和信息化部门，配合单位：省发展改革等部门）

　　促进大中小企业融通发展。鼓励行业龙头和骨干企业基于互联网平台开放各类资源，面向公众提供创新创业服务。对平台内创业项目年收入总计超过1亿元、5亿元、10亿元的平台主体企业，分别给予20万元、50万元、100万元的一次性奖补。（牵头责任单位：省经济和信息化部门，配合单位：省财政、发展改革等部门）

　　完善企业信息化公共服务平台。支持各地建立完善公共服务平台，为企业提供信息系统建设、企业运用云资源、智能化改造等规划咨询、方案设计、监理培训类服务。定期对各公共服务平台开展服务能力测试和可信度评估，推动平台服务水平和质量提升。（牵头责任单位：各市、县人民政府，配合单位：省经济和信息化等部门）

　　四、打造数字经济产业生态

　　建设数字经济特色园区。鼓励高端智能装备、信息网络通信设备等高技术产业，以及大数据服务、互联网金融等应用型产业向园区集聚。支持现有优势产业集聚区探索适应数字经济特点的政策措施，打造数字经济生态体系。鼓励骨干企业以产业链延伸和产业集聚为目标，积极引入产业配套企业，打造优势产业链。相关行业龙头企业在园区设立区域总部或子公司的，当地可根据企业需求提供一定的办公场所和人才公寓。（牵头责任单位：各市、县人民政府，配合单位：省发展改革、经济和信息化等部门）

　　构建数字技术应用生态。支持数字经济领域的平台型企业通过开放平台功能与数据、提供开发环境与工具等方式，广泛汇聚第三方应用开发者，构建开发与应用良性互动生态。每年安排1000万元，奖补一批由我省企业自主研发并取得实效的工业APP。（牵头责任单位：省经济和信息化部门，配合单位：省财政等部门）

　　培育信息化解决方案供应商。培育推广工业研发设计、生产制造、经营管理、营销服务等应用解决方案。对获得工业互联网领域国家级、省级优秀解决方案的，每个分别奖补100万元、50万元。（牵头责任单位：省经济和信息化部门，配合单位：省财政等部门）

　　培育数字经济创新共享服务联合体。支持省内数字经济领域的产学研平台资源整合，提供创意设计、研究开发、检验检测、标准信息、成果推广、创业孵化、跨界合作、展览展示、教育培训等一体化服务。每年优选一批联合体给予一次性奖补，每个最高500万元。（牵头责任单位：省经济和信息化部门，配合单位：省财政、质监、商务、教育等部门）

　　五、大力发展“数字＋”社会服务

　　推广智慧学校、智慧医疗、智慧养老。各级统筹教育项目资金，支持智慧学校（校园）建设，鼓励利用优质数字资源和新一代信息技术，推动教育理念与模式、教学内容与方法的改革创新。加快全省全民健康信息平台和远程医疗服务平台建设，推进基层医疗卫生机构辅助诊疗系统试点，打造省级基层智慧医疗和家庭医生服务示范区。推进智慧养老云平台建设，实施智慧养老机构创建工程，探索推行基于互联网的医养结合服务新模式，发挥各类养老产业基金作用。（牵头责任单位：省教育、卫生计生、民政部门，配合单位：省财政、经济和信息化等部门）对获得国家级智慧健康养老应用示范企业的，按相关规定给予奖补。（牵头责任单位：省经济和信息化部门，配合单位：省财政、卫生计生、民政等部门）

　　发展智慧旅游、智慧交通。指导建设一批智慧旅游试点景区、试点单位，省旅游发展相关专项资金对全省智慧旅游大数据体系研发及服务平台建设给予适当倾斜。建设无车承运人信用管理平台、联网治超综合管理系统、交通安全监测平台、交通一卡通、城市公共交通综合服务平台等。优化创新税收征管模式，支持无车承运人新业态发展。（牵头责任单位：省交通运输、旅游发展部门，配合单位：省财政、经济和信息化、税务等部门）

　　推进电子商务、智慧物流发展。统筹省级电子商务发展专项资金，重点支持农村产品上行、品牌培育、示范创建等，并对贫困地区电子商务发展予以倾斜。培育跨境电商生态链和产业链。推动智慧物流基础设施和信息平台建设，建设一批智慧物流园区。（牵头责任单位：省商务、发展改革部门，配合单位：省财政、交通运输等部门）

　　推进智慧社区建设。融合社区服务资源，推广社区网格化数字管理模式，构建开放的社区服务综合体，推进智慧社区试点。（牵头责任单位：省民政部门，配合单位：省财政、经济和信息化等部门）鼓励其他社会领域开展“数字＋”建设。

　　六、加快发展“互联网＋政务服务”

　　推进“互联网＋政务服务”建设。进一步深化“互联网＋政务服务”，推进“一网、一门、一次”改革，大力提升政务服务水平，为企业和群众提供更便利、更快捷、更高效率的服务。（牵头责任单位：省政务服务部门，配合单位：省发展改革等部门）

　　建立全省统一的政务云平台。原则上各政务部门新建的信息化系统在政务云平台上部署，不再批准政务部门在政务云平台之外新建信息系统。各部门已有的信息平台和信息系统逐步迁入政务云平台。（牵头责任单位：省发展改革部门，配合单位：省政务服务等部门，各市人民政府）

　　深化商事制度改革。统筹推进“证照分离”和“多证合一”改革，进一步扩大证照整合覆盖面。动态调整我省“多证合一”改革证照事项目录。推进企业名称登记管理改革，推进企业登记全程电子化和电子营业执照应用。进一步完善市场主体简易注销登记制度，扩大小微企业名录宣传和应用。（责任单位：省工商等部门）

　　七、完善信息基础设施

　　支持应用基础设施建设。支持有条件的市建设云计算中心、超算中心、数据中心、灾备中心等。全面扩大我省4G网络覆盖面，加快5G网络建设步伐，开放路灯、监控杆、机关事业单位办公楼等公共资源为5G网络提供基础支撑，供电部门提供便捷的5G基站电力报装流程。引导社会资本参与公共场所WLAN建设。加快部署基于IPv6的下一代互联网。统筹推进“一中心两基地多园区”的云计算大数据产业布局，引导各地因地制宜发展物联网。按照国家统一部署，推进北斗系统建设、产业、应用等各层面发展。加强网络安全事件的监测预警、防护处置、态势感知等系统设施建设，全面提升安全保障能力。（责任单位：省经济和信息化部门、省发展改革部门、省通信管理局、省网信办、省电力公司等）

　　推进企业内外网建设。鼓励企业综合运用IPv6、工业无源光网络、时间敏感网络、工业无线技术改造内网，科学运用窄带物联网和宽带网络，每年评选一批“网效之星”企业，每个给予一次性奖补50万元。（牵头责任单位：省经济和信息化部门，配合单位：省财政等部门，省通信管理局）

　　八、加强人才智力保障

　　积极引进高层次人才。对引进人才按有关规定予以支持，对引进的数字经济领域项目团队和高端领军人才按规定发放“江淮优才卡”。开设高层次人才职称评审绿色通道。支持企业在国外建立离岸研发中心、技术基地或孵化器。（牵头责任单位：省人力资源社会保障部门、省人才办，配合单位：各市人民政府，省财政等部门）

　　大力培养数字经济创新人才。支持相关企业设立博士后工作站，培养数字经济青年创新人才。支持企业推广首席信息官制。在数字经济领域组织开展享受政府特殊津贴人员、省学术和技术带头人及后备人选等高层次人才的选拔培养。推进“互联网＋职业培训”，提高数字经济培训领域的便利度和可及性。健全人才工作绩效评估和征信体系，建立数字经济人才创新的容错和奖励机制。（牵头责任单位：省人力资源社会保障部门，配合单位：省经济和信息化、教育等部门）

　　鼓励校企合作培养技能人才。鼓励省内高校和职业院校开设与数字经济相关的专业。鼓励高校和职业院校、科研院所与企业合作培养数字经济应用型人才，支持有条件的企业与高校和职业院校、科研院所合作建立数字经济实训基地。支持各云计算、大数据、网络安全等数字产业基地和高校、职业院校、社会培训机构合作，举办创新创业大赛、技能大赛等活动。（牵头责任单位：省教育、人力资源社会保障部门，配合单位：省经济和信息化等部门）

　　加强重大贡献荣誉激励。每年评选10个“数字经济领军企业”，给予每家企业一次性奖补100万元；评选10名“发展数字经济领军人物”，给予其领导的团队一次性奖补50万元。（牵头责任单位：省经济和信息化部门，配合单位：省教育、人力资源社会保障等部门）

　　九、加强财税支持和要素保障

　　强化资金支持。统筹“三重一创”、科技创新、制造强省等政策资金，对数字经济项目按规定给予支持。充分发挥各类基金的作用，促进数字经济发展壮大。（牵头责任单位：省财政部门，配合单位：省发展改革、科技、经济和信息化等部门）

　　落实税收优惠政策。严格落实固定资产加速折旧、企业研发费用加计扣除、软件和集成电路产业企业所得税优惠、小微企业税收优惠等政策。经认定为高新技术企业的，减按15%的税率征收企业所得税。落实股权激励和技术入股有关所得税政策。（牵头责任单位：省税务部门，配合单位：省财政、金融工作、科技、人力资源社会保障等部门）

　　加大金融支持力度。鼓励银行业金融机构优化数字技术企业信贷审批流程，适度提升风险容忍度，开展知识产权、商标专用权、专利权、股权、应收账款等质押贷款，扩大信用贷款规模。创新“税融通”业务，助力中小企业融资。建立省数字经济企业上市挂牌后备资源库，支持企业对接多层次资本市场上市挂牌，省财政按规定予以奖励。支持符合条件的企业发行债券。（牵头责任单位：省金融工作部门、安徽银监局，配合单位：省经济和信息化、科技、财政、税务、工商等部门，人行合肥中心支行）同等条件下，国有及国有控股担保（再担保）公司对数字经济企业给予优先担保，担保费率不高于1.2%。对数字经济领域高新技术企业投保的科技保险按规定给予补助。（牵头责任单位：省财政部门、安徽银监局，配合单位：省科技部门、省金融工作部门、安徽证监局、省担保集团等）

　　优先安排建设用地。各地可结合实际，确定当地重点发展的数字产业，以“先存量、后增量”的原则，优先安排用地供应。属于下一代信息网络产业（通信设施除外）、新型信息技术服务、电子商务服务等经营服务项目，可按商服用途落实用地。在不改变用地主体、规划条件的前提下，开发互联网信息资源，利用存量房产、土地资源发展新业态、创新商业模式、开展线上线下融合业务的，可实行继续按原用途和土地权利类型使用土地的过渡期政策。过渡期满，可根据企业发展业态和控制性详细规划，确定是否另行办理用地手续事宜。（牵头责任单位：各市人民政府，配合单位：省国土资源等部门）

　　给予用电支持。对符合条件的云计算中心、超算中心、数据中心、灾备中心等执行工商业及其他电价中的两部制电价。支持通信、广电运营企业及相关IT企业参加电力用户与发电企业直接交易。完善信息基础设施用电的临时性扶持政策，进一步降低用电成本。（牵头责任单位：省物价、能源部门，配合单位：省电力公司，有关市人民政府）

　　十、统筹组织实施

　　加强组织领导。设立省数字经济发展领导小组，省政府主要负责同志任组长，有关负责同志任副组长，省有关部门和单位负责同志为成员，统筹协调全省数字经济发展工作。领导小组办公室设在省经济和信息化委，会同省有关单位具体负责数字经济发展、支持政策落实等工作。建立健全数字经济发展统计监测指标体系。

　　保障政策落实。由省经济和信息化委会同有关部门制定落实具体实施细则，规范政策享受申报程序，加强审核评估，强化省、市和部门会商，充分利用信息管理平台，避免多头重复享受政策。加快资金拨付，加强资金监管、绩效评价和审计监督。各地各有关部门要完善配套政策，形成政策联动，推进政策落地。

　　加大宣传力度。充分运用各类媒体及数字经济平台，加强数字经济支持政策及新技术、新产品、新模式和领军企业、领军人物等宣传，营造全社会积极参与数字经济发展的良好氛围。

　　本政策由省经济和信息化委负责解释。
