# Supplementary material for: Research on quantitative evaluation of digital economy policy in China based on the PMC index model
Source: PLoS One. 2024 Feb 15;19(2):e0298312. doi: 10.1371/journal.pone.0298312 (PMC10868804; doi:10.1371/journal.pone.0298312)
Supplement: Attachment — Submitted filename: Response to Reviewers.docx [file pone.0298312.s003.docx]

**Response to Reviewers**

Dear Editor and Reviewers.

Thank you for offering us an opportunity to improve the quality of our submitted manuscript (PONE-D-23-07522R1). We appreciated very much the reviewers’ constructive and insightful comments. In this revision, we have addressed all of these comments. We hope the revised manuscript has now met the publication standard of your journal.

We highlighted all the revisions in yellow colour.

On the next pages, our point-to-point responses to the queries raised by the reviewers are listed.

Journal Requirements:

1. Please ensure that your manuscript meets PLOS ONE's style requirements, including those for file naming. The PLOS ONE style templates can be found at

https://journals.plos.org/plosone/s/file?id=wjVg/PLOSOne_formatting_sample_main_body.pdf and <https://journals.plos.org/plosone/s/file?id=ba62/PLOSOne_formatting_sample_title_authors_affiliations.pdf>

Response: Thanks for your advice. We have performed a full-text revision according to the style requirements of the PLOS ONE journal.

2. We note that the grant information you provided in the ‘Funding Information’ and ‘Financial Disclosure’ sections do not match.

When you resubmit, please ensure that you provide the correct grant numbers for the awards you received for your study in the ‘Funding Information’ section.

Response: Thanks for your suggestions, we have provided the correct funding information: The National Social Science Foundation of China, 21BJL073, A/Prof. Xuebin Tian.

3. Thank you for stating the following financial disclosure:

"33602"

Please state what role the funders took in the study. If the funders had no role, please state: "The funders had no role in study design, data collection and analysis, decision to publish, or preparation of the manuscript."

If this statement is not correct you must amend it as needed.

Please include this amended Role of Funder statement in your cover letter; we will change the online submission form on your behalf.

Response: The funders had no role, and we have stated this in the manuscript: The funder had no role in study design, data collection and analysis, decision to publish, or preparation of the manuscript.

4. In your Data Availability statement, you have not specified where the minimal data set underlying the results described in your manuscript can be found. PLOS defines a study's minimal data set as the underlying data used to reach the conclusions drawn in the manuscript and any additional data required to replicate the reported study findings in their entirety. All PLOS journals require that the minimal data set be made fully available. For more information about our data policy, please see http://journals.plos.org/plosone/s/data-availability.

"Upon re-submitting your revised manuscript, please upload your study’s minimal underlying data set as either Supporting Information files or to a stable, public repository and include the relevant URLs, DOIs, or accession numbers within your revised cover letter. For a list of acceptable repositories, please see http://journals.plos.org/plosone/s/data-availability#loc-recommended-repositories. Any potentially identifying patient information must be fully anonymized.

Important: If there are ethical or legal restrictions to sharing your data publicly, please explain these restrictions in detail. Please see our guidelines for more information on what we consider unacceptable restrictions to publicly sharing data: http://journals.plos.org/plosone/s/data-availability#loc-unacceptable-data-access-restrictions. Note that it is not acceptable for the authors to be the sole named individuals responsible for ensuring data access.

We will update your Data Availability statement to reflect the information you provide in your cover letter.

Response: We have uploaded the relevant data to the manuscript and made it editable in the revised cover letter. Supporting information: S1 Appendix. 37 original policy documents. (DOCX), S2 Appendix. List of policy names (XLSX). Data availability statement: All relevant data are within the paper and its Supporting Information fields.

DOIs:  <https://doi.org/10.5061/dryad.0rxwdbs5s>

5. PLOS requires an ORCID iD for the corresponding author in Editorial Manager on papers submitted after December 6th, 2016. Please ensure that you have an ORCID iD and that it is validated in Editorial Manager. To do this, go to ‘Update my Information’ (in the upper left-hand corner of the main menu), and click on the Fetch/Validate link next to the ORCID field. This will take you to the ORCID site and allow you to create a new iD or authenticate a pre-existing iD in Editorial Manager. Please see the following video for instructions on linking an ORCID iD to your Editorial Manager account: <https://www.youtube.com/watch?v=_xcclfuvtxQ>

Response: Thanks for your suggestion, We have registered an ORCID ID and verified it in the journal's submission system.

Reviewer #1:

1. In order to establish the significance and value of the study, it is necessary to provide a comprehensive rationale for the research, which emphasizes its relevance and unique contributions to the current scholarly discourse. This will strengthen the study's originality and scholarly impact. Following are useful suggested studies to get the benefit to update this part:https://doi.org/10.1007/s11356-022-19718-6;https://doi.org/10.3390/su14031054; https://doi.org/10.1007/s11356-021-17438-x;https://doi.org/10.1108/FS-02-2021-0053; https://doi.org/10.1007/s11356-022-209227;https://doi.org/10.1177/21582440211061554;DOI: 10.26677/TR1010.2019.145; https://doi.org/10.3389/fenvs.2022.967418;https://doi.org/10.1177/0958305X221134113

Response: We have optimized and revised the theoretical foundation based on the references provided by the peer reviewers and aligned it with the direction of our research. The specific changes are as follows:

The theory of public policy evaluation is a theoretical framework for assessing policies, aimed at determining their effectiveness, feasibility, sustainability, and fairness. Its scope of application encompasses various domains, including government policies, social policies, and economic policies. On one hand, this theory enhances the scientific and practical aspects of policy formulation by offering a scientific and systematic approach to evaluating policy effects. On the other hand, evaluation outcomes assist policymakers in comprehending the effects of policy implementation, thereby enabling policy adjustments and improvements based on the evaluation results. Existing methods for public policy evaluation each possess distinctive characteristics, providing crucial references for assessments related to digital economic policies. Due to the distinct scopes of application for each method, evaluation outcomes also exhibit slight variations. The grey relational degree model exhibits subjectivity and substantial error in determining optimal values for various indicators. The slow convergence of the BP neural network hampers the precision of policy evaluation. The fuzzy comprehensive evaluation method lacks objectivity in assessing indicators. It's noteworthy that current policy evaluation research excessively emphasizes ex-post evaluation, paying insufficient attention to the policies themselves. In contrast, the PMC (Policy Modeling Consistency) index model showcases a more prominent comprehensive evaluation effect. This method underscores the internal consistency of policies, offering advantages such as diversity assessment, pliability of indicators, and distinguishable gradations. Additionally, serving as a model for evaluating policy texts, it boasts traits like low cost and ease of operation, which mitigate subjectivity during the evaluation process. While assessing the merits and demerits of individual policies, it better reveals policy disparities across different periods or regions through policy comparisons.

Meanwhile, we have also made certain changes to the contributions of this article:

The contributions of this study are as follows:

Quantitative assessment of national and provincial-level digital economic policy texts in China is conducted through the utilization of content analysis and text mining methods. This endeavor aims to gain deeper insights into the ongoing trends within China's digital economic industry. Moreover, it seeks to furnish optimized recommendations for the formulation and refinement of future policies. Simultaneously, from an empirical standpoint, a quantitative evaluation of China's digital economic policies is performed. The outcomes of policy evaluation serve as a reference for standardizing and enhancing the practicality of the future digital economic industry policy evaluation system, thereby facilitating the high-quality development of China's digital economic sector.

2. English check/editing: I found major typos/ grammatical, this study was presented with depraved English language usage.

Response: Thank you for the reviewer's feedback. We have made corrections to all the spelling and grammar errors in the article.

3. Recheck throughout the paper and ensure that all abbreviations are defined the first time they are used.

Response: Thanks for your suggestion. We have provided definitions for terms such as PMC (Policy Modeling Consistency) index model and "PKULaw" (Peking University Legal Information Retrieval System) legal database in their initial instances of use within the article.

4. The paper clearly lacks the sense of paragraphing; some paragraphs are too shorting and consist of only few lines. Kindly rearrange your paragraphs and provide arguments about one main theme in each paragraph.

Response: Thanks for your advice. We have merged the five paragraphs of the article into four paragraphs, while also summarizing and arranging the themes of each paragraph.

5. The Results and described with reference to the references of the parameters that influence the results of the research will make it more original. The purpose of the study and the reasons clearly set forth.

Response: The research objective of this paper is to employ the PMC index model's research methodology to provide an in-depth interpretation of China's digital economic policies. Building upon text mining and content analysis, this study employs a combined quantitative and qualitative approach to thoroughly explore the strengths and weaknesses of China's digital economic policies. Subsequently, it presents corresponding optimization paths and recommendations, aiming to furnish scientific decision-making foundations for expediting the development of China's digital economic market.

6. Please increase the pixel size of all Figures.

Response: We have increased the pixel dimensions of the eight figures in Figure 1.

7. The conclusion is sufficiently discussed with some useful policy implications. However, limitations and future directions of research should have a reasonably sized paragraph instead of just a few lines written half-heartedly at the end of the conclusion.

Response: Thank you for the suggestions from the reviewer. We have summarized the limitations of this study and outlined future research directions.

**Research Limitations and Future Trends**

It should be noted that this study primarily analyzed national and provincial policies in China and did not select city-level policies within various provinces. Due to variations in the level of digital industry development among different municipalities, a unified calculation standard has not yet been established. Therefore, future research should aim to expand the sample size and diversify the selection of subjects. Additionally, a comprehensive cross-analysis of policy content can be conducted from different dimensions by integrating policy tool theories. This approach can offer decision-makers objective and feasible solutions. Regarding policy quantification assessment, experimenting with various evaluation methods and selecting the optimal option after comprehensive comparisons could be attempted.

Building upon the research findings of this paper and considering the current development of China's digital economic industry, future studies can be directed towards the following aspects:

On one hand, it is essential to accelerate the integration of the digital economy with the real economy. As the digital economy and its systems become increasingly sophisticated, policies need to be appropriately adjusted, encompassing goals, positioning, and tools. Focusing on digital industrialization and industry digitization, policies should provide broader application scenarios for digital economic development, amplifying and multiplying the impact of digital technologies on economic growth.

On the other hand, emphasis should be placed on innovation-driven development. Presently, there are noticeable disparities in the development of the digital economy across the eastern, central, and western regions of China. Innovation-driven approaches can bridge these regional gaps, serving as a crucial lever for regional coordination. This strategy enables the utilization of developed digital economic regions' driving roles and innovation advantages, enhances regional collaborative innovation capabilities, and promotes high-quality development of the digital economic industry.

Lastly, in order to achieve the policy objectives of digital industrialization and industry digitization, internal demand needs to be constantly improved and external demand needs to be fully utilized. The development of digital economy is long-term and systematic. Therefore, it is needed to accelerate the establishment of data element market, promote the equality and benefits of public services, improve the governance system of the digital economy, eliminate the digital divide and lay a solid foundation for building a good modern market system of digital economy.

8. Follow the right style of citation throughout the manuscript by checking the guidelines of (PLOS ONE) journal or any previously published paper in the journal.

Response: Thanks for pointing out the mistake. We have made all the necessary changes to adhere to the reference citation style of the PLOS ONE journal.

**References**

1.	Litvinenko VS. Digital Economy as a Factor in the Technological Development of the Mineral Sector. Natural Resources Research. 2020;29(3):1521-41. doi: 10.1007/s11053-019-09568-4. PubMed PMID: WOS:000492018400001.

2.	Meng FS, Zhao Y. How does digital economy affect green total factor productivity at the industry level in China: from a perspective of global value chain. Environmental Science and Pollution Research. 2022;29(52):79497-515. doi: 10.1007/s11356-022-21434-0. PubMed PMID: WOS:000812488200005.

3.	Urinovich Kobilov A, Khashimova DP, Mannanova SG, Ogli Abdulakhatov MM. Modern Content and Concept of Digital Economy. International Journal of Multicultural and Multireligious Understanding. 2022;9(2). doi: 10.18415/ijmmu.v9i2.3524.

4.	Wang J, Dong K, Dong X, Taghizadeh-Hesary FJEE. Assessing the digital economy and its carbon-mitigation effects: The case of China. 2022;113:106198.

5.	Sturgeon TJJGsj. Upgrading strategies for the digital economy. 2021;11(1):34-57.

6.	Chen YJCER. Improving market performance in the digital economy. 2020;62:101482.

7.	Vasyltsiv T, Lupak R, Kunytska-Iliash M, Shtets TJZNWwP. Trends in state policy with a view to improving structural characteristics of the digital economy. 2020;89(2):41-54.

8.	Golov R, Palamarchuk A, Anisimov K, Andrianov AJRER. Cluster policy in a digital economy. 2021;41:631-3.

9.	Notley TJMIA. The environmental costs of the global digital economy in Asia and the urgent need for better policy. 2019;173(1):125-41.

10.	Van Gorp N, Honnefelder SJC, Strategies. Challenges for competition policy in the digitalised economy. 2015;(99):149.

11.	Ruiz Estrada MAJAaS. The policy modeling research consistency index (PMC-index). 2010.

12.	Jiang XJJoCE, Studies B. Digital economy in the post-pandemic era. 2020;18(4):333-9.

13.	Zhou G. The Impact of COVID-19 Pandemic on the Digital Economy and Policy Responses. COVID-19's Economic Impact and Countermeasures in China2022. p. 175-97.

14.	Liu Y, Li J, Xu Y. Quantitative Evaluation of High-Tech Industry Policies Based on the PMC-Index Model: A Case Study of China’s Beijing-Tianjin-Hebei Region. Sustainability. 2022;14(15). doi: 10.3390/su14159338.

15.	Dai S, Zhang W, Zong J, Wang Y, Wang G. How Effective Is the Green Development Policy of China’s Yangtze River Economic Belt? A Quantitative Evaluation Based on the PMC-Index Model. International Journal of Environmental Research and Public Health. 2021;18(14). doi: 10.3390/ijerph18147676.

16.	Kuang B, Han J, Lu X, Zhang X, Fan X. Quantitative evaluation of China’s cultivated land protection policies based on the PMC-Index model. Land Use Policy. 2020;99. doi: 10.1016/j.landusepol.2020.105062.

17.	Lu C, Wang B, Chen T, Yang J. A Document Analysis of Peak Carbon Emissions and Carbon Neutrality Policies Based on a PMC Index Model in China. Int J Environ Res Public Health. 2022;19(15). Epub 2022/08/13. doi: 10.3390/ijerph19159312. PubMed PMID: 35954666; PubMed Central PMCID: PMCPMC9368600.

18.	Nkoua Nkuika GLF, Yiqun X. Quantitative Evaluation and Optimization Path of Advanced Manufacturing Development Policy Based on the PMC–AE Index Model. International Journal of Global Business and Competitiveness. 2022;17(S1):1-11. doi: 10.1007/s42943-022-00063-x.

19.	Liu L, Chen J, Wang C, Wang Q. Quantitative evaluation of China's basin ecological compensation policies based on the PMC index model. Environ Sci Pollut Res Int. 2023;30(7):17532-45. Epub 2022/10/06. doi: 10.1007/s11356-022-23354-5. PubMed PMID: 36197610.

20.	Zhao Y, Wu L. Research on Emergency Response Policy for Public Health Emergencies in China-Based on Content Analysis of Policy Text and PMC-Index Model. Int J Environ Res Public Health. 2022;19(19). Epub 2022/10/15. doi: 10.3390/ijerph191912909. PubMed PMID: 36232209; PubMed Central PMCID: PMCPMC9566489.

21.	Liu F, Liu Z. Quantitative Evaluation of Waste Separation Management Policies in the Yangtze River Delta Based on the PMC Index Model. Int J Environ Res Public Health. 2022;19(7). Epub 2022/04/13. doi: 10.3390/ijerph19073815. PubMed PMID: 35409497; PubMed Central PMCID: PMCPMC8998125.

22.	Li Z, Guo X. Quantitative evaluation of China's disaster relief policies: A PMC index model approach. International Journal of Disaster Risk Reduction. 2022;74. doi: 10.1016/j.ijdrr.2022.102911.

23.	Li Y, He R, Liu J, Li C, Xiong J. Quantitative Evaluation of China’s Pork Industry Policy: A PMC Index Model Approach. Agriculture. 2021;11(2). doi: 10.3390/agriculture11020086.

24.	Khan A, Yusof Z. Terrorist economic impact evaluation (TEIE) model: the case of Pakistan. Quality & Quantity. 2016;51(3):1381-94. doi: 10.1007/s11135-016-0336-z.

25.	Estrada MARJJoPM. Policy modeling: Definition, classification and evaluation. 2011;33(4):523-36.

26.	Liu J, Li N, Cheng L. Mining and quantitative evaluation of COVID-19 policy tools in China. PLoS One. 2023;18(4):e0284143. Epub 2023/04/08. doi: 10.1371/journal.pone.0284143. PubMed PMID: 37027438; PubMed Central PMCID: PMCPMC10081750.

27.	Yang T, Xing C, Li X. Evaluation and analysis of new-energy vehicle industry policies in the context of technical innovation in China. Journal of Cleaner Production. 2021;281. doi: 10.1016/j.jclepro.2020.125126.

Reviewer #2:

This manuscript studied quantitative evaluation of digital economy policy in China based on the PMC index model. Although the topic is interesting, the innovation and contribution is not high. In addition, there are some shortcomings in this manuscript. Firslty, variable setting of PMC model of digital economy policy is not clearly. Please illustrate in detail. Secondly, how to testify the efficency of the PMC model? Can you compare with other models? Finally, the corresponding countermeasures and suggestions should be more concrete and can be executed.

Response: Thank you for the feedback from the reviewing expert. In this study, all the primary variables of the PMC index model for digital economy policy are based on the research of scholars such as Ruiz, Liu, and Yang. A total of 10 primary variables have been extracted: Policy Type (X_1_), Policy Effectiveness (X_2_), Policy Level (X_3_), Policy Felids (X_4_), Policy Guarantee (X_5_), Policy Audience (X_6_), Policy Objectives (X_7_), Policy Evaluation (X_8_), Policy Perspective (X_9_), and Policy Publicity (X_10_). Due to the nature of policy, policy tools, and other primary variables, the underlying secondary variables cannot be directly obtained from policy texts. Therefore, the use of text mining tool Rostcm.6 is necessary for conducting word frequency analysis. Based on the characteristics of digital economic development, a total of 10 primary indicators and 45 secondary indicators have been constructed.

The PMC index model follows a binary principle, where a value of 1 indicates the presence of a factor, and 0 indicates its absence. The reason for choosing this model is based on the consideration that this approach avoids overlooking any variables, treating each variable's importance consistently. Additionally, the paper highlights the advantages and disadvantages of other policy evaluation models, leading to the final selection of the PMC index evaluation model.

The theory of public policy evaluation is a theoretical framework for assessing policies, aimed at determining their effectiveness, feasibility, sustainability, and fairness. Its scope of application encompasses various domains, including government policies, social policies, and economic policies. On one hand, this theory enhances the scientific and practical aspects of policy formulation by offering a scientific and systematic approach to evaluating policy effects. On the other hand, evaluation outcomes assist policymakers in comprehending the effects of policy implementation, thereby enabling policy adjustments and improvements based on the evaluation results. Existing methods for public policy evaluation each possess distinctive characteristics, providing crucial references for assessments related to digital economic policies. Due to the distinct scopes of application for each method, evaluation outcomes also exhibit slight variations. The grey relational degree model exhibits subjectivity and substantial error in determining optimal values for various indicators. The slow convergence of the BP neural network hampers the precision of policy evaluation. The fuzzy comprehensive evaluation method lacks objectivity in assessing indicators. It's noteworthy that current policy evaluation research excessively emphasizes ex-post evaluation, paying insufficient attention to the policies themselves. In contrast, the PMC (Policy Modeling Consistency) index model showcases a more prominent comprehensive evaluation effect. This method underscores the internal consistency of policies, offering advantages such as diversity assessment, pliability of indicators, and distinguishable gradations. Additionally, serving as a model for evaluating policy texts, it boasts traits like low cost and ease of operation, which mitigate subjectivity during the evaluation process. While assessing the merits and demerits of individual policies, it better reveals policy disparities across different periods or regions through policy comparisons.

Finally, based on the feedback from the peer reviewers, this study has incorporated and enhanced the recommendations and strategies. The specific improvements are as follows:

**Research Limitations and Future Trends**

It should be noted that this study primarily analyzed national and provincial policies in China and did not select city-level policies within various provinces. Due to variations in the level of digital industry development among different municipalities, a unified calculation standard has not yet been established. Therefore, future research should aim to expand the sample size and diversify the selection of subjects. Additionally, a comprehensive cross-analysis of policy content can be conducted from different dimensions by integrating policy tool theories. This approach can offer decision-makers objective and feasible solutions. Regarding policy quantification assessment, experimenting with various evaluation methods and selecting the optimal option after comprehensive comparisons could be attempted.

Building upon the research findings of this paper and considering the current development of China's digital economic industry, future studies can be directed towards the following aspects:

On one hand, it is essential to accelerate the integration of the digital economy with the real economy. As the digital economy and its systems become increasingly sophisticated, policies need to be appropriately adjusted, encompassing goals, positioning, and tools. Focusing on digital industrialization and industry digitization, policies should provide broader application scenarios for digital economic development, amplifying and multiplying the impact of digital technologies on economic growth.

On the other hand, emphasis should be placed on innovation-driven development. Presently, there are noticeable disparities in the development of the digital economy across the eastern, central, and western regions of China. Innovation-driven approaches can bridge these regional gaps, serving as a crucial lever for regional coordination. This strategy enables the utilization of developed digital economic regions' driving roles and innovation advantages, enhances regional collaborative innovation capabilities, and promotes high-quality development of the digital economic industry.

Lastly, in order to achieve the policy objectives of digital industrialization and industry digitization, internal demand needs to be constantly improved and external demand needs to be fully utilized. The development of digital economy is long-term and systematic. Therefore, it is needed to accelerate the establishment of data element market, promote the equality and benefits of public services, improve the governance system of the digital economy, eliminate the digital divide and lay a solid foundation for building a good modern market system of digital economy.

Reviewer #3:

First of all, I congratulates to the authors for making a significant empirical contribution in economic literature. The author implemented significant contribution in order to have their work published in the PLOS One journal.

Response: Thank you for the recognition and trust of the reviewing experts.

Sincerely,

Tianzun Wang
