# Supplementary material for: Research on quantitative evaluation of digital economy policy in China based on the PMC index model
Source: PLoS One. 2024 Feb 15;19(2):e0298312. doi: 10.1371/journal.pone.0298312 (PMC10868804; doi:10.1371/journal.pone.0298312)
Supplement: Attachment — Submitted filename: Response to Reviewers.docx [file pone.0298312.s004.docx]

**Response to Reviewers**

Dear Editor and Reviewers.

Thank you for offering us an opportunity to improve the quality of our submitted manuscript (PONE-D-23-07522R1). We appreciated very much the reviewers’ constructive and insightful comments. In this revision, we have addressed all of these comments. We hope the revised manuscript has now met the publication standard of your journal.

We highlighted all the revisions in yellow colour.

On the next pages, our point-to-point responses to the queries raised by the reviewers are listed.

Reviewer #1:

1. In order to establish the significance and value of the study, it is necessary to provide a comprehensive rationale for the research, which emphasizes its relevance and unique contributions to the current scholarly discourse. This will strengthen the study's originality and scholarly impact. Author don’t provide clearly? revised it

Response: Regarding the comprehensive rationale for the research, we have supplemented and modified it. The specific modifications are as follows:

Compared with other methods, the PMC index model has important advantages. The evaluation dimensions are rich, and by adding evaluation dimensions instead of calculating indicator weights, it effectively avoids indicator weight errors and subjective evaluation biases, making the evaluation results more objective and accurate. Focusing on pre-policy evaluation, analyzing key content and keywords in policy texts, fills the gap in research and analysis of policy content. The PMC index evaluation method can be applied to both single industry policies and regional policy systems.

The digital economy policy is a series of policy tool combinations that promote the development of the digital economy, involving multiple dimensions such as policy nature, policy objectives, policy content, policy effects, etc. How to objectively evaluate is a very important issue. At present, there is relatively little literature on the evaluation of digital economy policies, and policy evaluation is the most important link in the process of public policy formulation and management [25, 26]. It uses relevant research methods to systematically measure and judge the effectiveness of policy intervention and implementation [27]. Through the evaluation of digital economy policies, not only can scientific judgments be made on the value of the policies themselves [28], but also the actual effects of policy formulation and implementation can be tested [29]. Therefore, in view of this, this article combines digital economy policy sample analysis, text mining, and PMC index model to construct a quantitative evaluation index system for digital economy policies. It conducts sample analysis and text mining on China's digital economy policies, and quantitatively evaluates and analyzes typical digital economy policy texts at the central and local levels, in order to provide decision-making basis for the improvement of relevant policies and the formulation of new policies.

25.	Abbass K, Song H, Khan F, Begum H, Asif M. Fresh insight through the VAR approach to investigate the effects of fiscal policy on environmental pollution in Pakistan. Environ Sci Pollut Res Int. 2022;29(16):23001-14. Epub 2021/11/20. doi: 10.1007/s11356-021-17438-x. PubMed PMID: 34797543.

26.	Abbass K, Qasim MZ, Song H, Murshed M, Mahmood H, Younis I. A review of the global climate change impacts, adaptation, and sustainable mitigation measures. Environmental Science and Pollution Research. 2022;29(28):42539-59. doi: 10.1007/s11356-022-19718-6.

27.	Abbass K, Niazi AAK, Basit A, Qazi TF, Song H, Begum H. Uncovering Effects of Hot Potatoes in Banking System: Arresting Die-Hard Issues. SAGE Open. 2021;11(4). doi: 10.1177/21582440211061554.

28.	Fu H, Abbass K, Qazi TF, Niazi AAK, Achim MV. Analyzing the barriers to putting corporate financial expropriations to a halt: A structural modeling of the phenomenon. Frontiers in Environmental Science. 2022;10. doi: 10.3389/fenvs.2022.967418.

29.	Amin N, Shabbir MS, Song H, Abbass K. Renewable energy consumption and its impact on environmental quality: A pathway for achieving sustainable development goals in ASEAN countries. Energy & Environment. 2022. doi: 10.1177/0958305x221134113.

Any single indicator can be misleading, but if multiple composite indicators point to the same result, it can provide a more accurate judgment of the evaluated thing. The PMC index first determines the meaning and level of variables at all levels, and then evaluates and analyzes the policy's advantages and disadvantages through the aggregated consistency level. This method attempts to find highly saturated secondary variables that can characterize policy characteristics and assign consistent weights to these variables to avoid subjective limitations. In addition, all variables are binary balanced, greatly simplifying the complexity of PMC index calculation.

This article uses a composite analysis method that combines policy sample analysis, text mining, and PMC index model. On the basis of sorting out China's digital economy policies, text mining is used to search for important and highly correlated text data, which forms a component of secondary indicators. Unstructured text data is transformed into structured and readable data, and the PMC index model is used to conduct quantitative evaluation research on digital economy policies in central and local areas of China.

Compared with the traditional economy, the digital economy focuses on the application of products and the extension of services, is demand oriented, focuses on discovering potential and intangible user needs, provides personalized services, and creates user value. Due to the existence of digital technology, more consumers are involved in the production and consumption of products, so the dominant position of the digital economy is relatively unclear.

The digital economy is essentially a technology economy paradigm, which is an optimal practice model for economy and society based on technological innovation. It responds to structural crises caused by technological changes through institutional changes, thus forming a relatively stable and sustainable behavior. The digital economy is driven by digital knowledge and information as key production factors, modern information networks as the main carrier, and the efficient utilization of information and communication technology as an important driving force for efficiency improvement and economic optimization. Therefore, introducing data elements and changing social production methods can provide an important experimental environment for expanding current economic and management theories; The development of the digital economy requires new theories from economics and management to explain and promote the construction of new theories.

Since China first included the digital economy in the Two Sessions Report in 2017, the national level has attached increasing importance to promoting the development of the digital economy. To address various practical issues in the development of the digital economy, various Chinese ministries have issued a series of policies and regulations. The introduction of numerous policies has also made the policy and regulatory system in this field increasingly complex, leading to ineffective implementation and poor coordination in the policy integration environment. Policy analysis is the main basis for the abolition, reform, and establishment of digital economy policies. The primary task of policy analysis is to identify and conceptualize the problems that need to be solved, and policy problems stem from unresolved practical problems within the current policy system. The evaluation and analysis based on digital economy policy texts have important practical value and significance. Therefore, when quantitatively evaluating China's digital economy policies, this article will focus on distinguishing the advantages and disadvantages of current policies based on existing practical problems, providing decision-making basis for the improvement of relevant policies and the formulation of new policies.

2. The authors are requested to provide a thorough justification for the choice of the current methodology, including an explanation of the benefits and advantages it offers over alternative approaches. This will lend greater credibility and rigor to the study's research design.

Response: Thanks for your suggestion. Regarding the alternative approaches, we have supplemented and modified it. The specific modifications are as follows:

Compared with other methods, the PMC index model has important advantages. The evaluation dimensions are rich, and by adding evaluation dimensions instead of calculating indicator weights, it effectively avoids indicator weight errors and subjective evaluation biases, making the evaluation results more objective and accurate. Focusing on pre-policy evaluation, analyzing key content and keywords in policy texts, fills the gap in research and analysis of policy content. The PMC index evaluation method can be applied to both single industry policies and regional policy systems.

3. To highlight the novelty and contribution of the current study, it is necessary to outline the ways in which it diverges from the existing body of literature. Therefore, the concluding section of the literature review should include a clear differentiation of the study from prior research.

Response: Thanks for your suggestion. Regarding the differentiation of the study from prior research, we have supplemented and modified it. The specific modifications are as follows:

The digital economy policy is a series of policy tool combinations that promote the development of the digital economy, involving multiple dimensions such as policy nature, policy objectives, policy content, policy effects, etc. How to objectively evaluate is a very important issue. At present, there is relatively little literature on the evaluation of digital economy policies, and policy evaluation is the most important link in the process of public policy formulation and management [25, 26]. It uses relevant research methods to systematically measure and judge the effectiveness of policy intervention and implementation [27]. Through the evaluation of digital economy policies, not only can scientific judgments be made on the value of the policies themselves [28], but also the actual effects of policy formulation and implementation can be tested [29]. Therefore, in view of this, this article combines digital economy policy sample analysis, text mining, and PMC index model to construct a quantitative evaluation index system for digital economy policies. It conducts sample analysis and text mining on China's digital economy policies, and quantitatively evaluates and analyzes typical digital economy policy texts at the central and local levels, in order to provide decision-making basis for the improvement of relevant policies and the formulation of new policies.

25.	Abbass K, Song H, Khan F, Begum H, Asif M. Fresh insight through the VAR approach to investigate the effects of fiscal policy on environmental pollution in Pakistan. Environ Sci Pollut Res Int. 2022;29(16):23001-14. Epub 2021/11/20. doi: 10.1007/s11356-021-17438-x. PubMed PMID: 34797543.

26.	Abbass K, Qasim MZ, Song H, Murshed M, Mahmood H, Younis I. A review of the global climate change impacts, adaptation, and sustainable mitigation measures. Environmental Science and Pollution Research. 2022;29(28):42539-59. doi: 10.1007/s11356-022-19718-6.

27.	Abbass K, Niazi AAK, Basit A, Qazi TF, Song H, Begum H. Uncovering Effects of Hot Potatoes in Banking System: Arresting Die-Hard Issues. SAGE Open. 2021;11(4). doi: 10.1177/21582440211061554.

28.	Fu H, Abbass K, Qazi TF, Niazi AAK, Achim MV. Analyzing the barriers to putting corporate financial expropriations to a halt: A structural modeling of the phenomenon. Frontiers in Environmental Science. 2022;10. doi: 10.3389/fenvs.2022.967418.

29.	Amin N, Shabbir MS, Song H, Abbass K. Renewable energy consumption and its impact on environmental quality: A pathway for achieving sustainable development goals in ASEAN countries. Energy & Environment. 2022. doi: 10.1177/0958305x221134113.

4. I would kindly request that the authors re-examine the policy implications derived from the study's findings, to ensure their accuracy and relevance to the study's objectives.

Response: Thanks for your suggestion. Regarding the policy implications on the role of stakeholders in the digital economy, we have supplemented and modified it. The specific modifications are as follows:

Finally, pay attention to the role of stakeholders in the digital economy. In the era of digital economy, stakeholders have already extended to employees, users, and partners. Stakeholders should not only include shareholders, but also employees in the future. We should not only focus on shareholders, but also on employees. Only with excellent employees can we create greater value for the enterprise and achieve better development. The digital economy is built on the basis of data, and enterprises earn more profits by collecting user data. Enterprises should be regulated while using data to earn more profits. Regulators need to play a more important role in protecting user privacy and data usage. Protecting privacy requires stricter regulations to ensure it. The government is facing a new market composed of an increasing number of platforms, which poses an unprecedented challenge to its market governance. On the basis of a scientific understanding of the new economic form of the digital economy and a rational analysis of the two growth paths of the digital economy, the government should redesign the content of government management functions in the digital economy and further play a positive role.

5. In order to ensure the relevance and timeliness of the study’s cited sources, it is recommended that the authors review and update the references section.

Response: Thanks for your suggestion. We have updated and improved the content of the references section. The modifications are as follows:

14.	Abbass K, Begum H, Alam ASAF, Awang AH, Abdelsalam MK, Egdair IMM, et al. Fresh Insight through a Keynesian Theory Approach to Investigate the Economic Impact of the COVID-19 Pandemic in Pakistan. Sustainability. 2022;14(3). doi: 10.3390/su14031054.

17.	Liu F, Tang J, Rustam A, Liu Z. Evaluation of the central and local power batteries recycling policies in China: A PMC-Index model approach. Journal of Cleaner Production. 2023;427. doi: 10.1016/j.jclepro.2023.139073.

25.	Abbass K, Song H, Khan F, Begum H, Asif M. Fresh insight through the VAR approach to investigate the effects of fiscal policy on environmental pollution in Pakistan. Environ Sci Pollut Res Int. 2022;29(16):23001-14. Epub 2021/11/20. doi: 10.1007/s11356-021-17438-x. PubMed PMID: 34797543.

26.	Abbass K, Qasim MZ, Song H, Murshed M, Mahmood H, Younis I. A review of the global climate change impacts, adaptation, and sustainable mitigation measures. Environmental Science and Pollution Research. 2022;29(28):42539-59. doi: 10.1007/s11356-022-19718-6.

27.	Abbass K, Niazi AAK, Basit A, Qazi TF, Song H, Begum H. Uncovering Effects of Hot Potatoes in Banking System: Arresting Die-Hard Issues. SAGE Open. 2021;11(4). doi: 10.1177/21582440211061554.

28.	Fu H, Abbass K, Qazi TF, Niazi AAK, Achim MV. Analyzing the barriers to putting corporate financial expropriations to a halt: A structural modeling of the phenomenon. Frontiers in Environmental Science. 2022;10. doi: 10.3389/fenvs.2022.967418.

29.	Amin N, Shabbir MS, Song H, Abbass K. Renewable energy consumption and its impact on environmental quality: A pathway for achieving sustainable development goals in ASEAN countries. Energy & Environment. 2022. doi: 10.1177/0958305x221134113.

30.	Xiong Y, Zhang C, Qi H. How effective is the fire safety education policy in China? A quantitative evaluation based on the PMC-index model. Safety Science. 2023;161. doi: 10.1016/j.ssci.2023.106070.

32.	Kuang B, Han J, Lu X, Zhang X, Fan X. Quantitative evaluation of China’s cultivated land protection policies based on the PMC-Index model. Land Use Policy. 2020;99. doi: 10.1016/j.landusepol.2020.105062.

34.	Hu M, Guo C, Wang Y, Ma D. Quantitative evaluation of China's private universities provincial public funding policies based on the PMC-Index model. PLoS One. 2023;18(12):e0295601. Epub 2023/12/12. doi: 10.1371/journal.pone.0295601. PubMed PMID: 38085719; PubMed Central PMCID: PMCPMC10715659.

36.	Meng J, Xu W. Quantitative Evaluation of Carbon Reduction Policy Based on the Background of Global Climate Change. Sustainability. 2023;15(19). doi: 10.3390/su151914581.

37.	Xu J, Zhang Z, Xu Y, Liu L, Pei T. Quantitative evaluation of waste sorting management policies in China’s major cities based on the PMC index model. Frontiers in Environmental Science. 2023;11. doi: 10.3389/fenvs.2023.1065900.

6. The study appears to employ an excessive amount of acronyms, which may impede comprehension for the wider readership of the journal. Thus, it is recommended that the authors avoid excessive usage of acronyms and instead use the full form of terms to ensure ease of understanding for readers.

Response: Thanks for your suggestion. We have provided definitions for terms such as Corona Virus Disease 2019 COVID-19 () and Back Propagation (BP) neural network legal database in their initial instances of use within the article. It should be noted that this study employed the Policy Modeling Consistency (PMC) index model for the quantitative assessment of digital economic policies. As a result, there are multiple instances of the term "PMC" throughout the text, but definitions have been provided at its initial use.

7. The paper examines such selecting the text of digital economy policy issued by China government, the paper constructs a quantitative evaluation model of digital economy policy using the methods of content analysis and text mining etc. What is the unified theoretical framework for the impact of evaluation model of digital economy policy? able to combine all the variable above. What is the theoretical basis for the integration of multiple factors? In addition, why not put the above dependent and independent variables into a unified.

Response: Thanks for your suggestion. Regarding the policy evaluation of digital economy , we have supplemented and modified it. The specific modifications are as follows:

Any single indicator can be misleading, but if multiple composite indicators point to the same result, it can provide a more accurate judgment of the evaluated thing. The PMC index first determines the meaning and level of variables at all levels, and then evaluates and analyzes the policy's advantages and disadvantages through the aggregated consistency level. This method attempts to find highly saturated secondary variables that can characterize policy characteristics and assign consistent weights to these variables to avoid subjective limitations. In addition, all variables are binary balanced, greatly simplifying the complexity of PMC index calculation.

This article uses a composite analysis method that combines policy sample analysis, text mining, and PMC index model. On the basis of sorting out China's digital economy policies, text mining is used to search for important and highly correlated text data, which forms a component of secondary indicators. Unstructured text data is transformed into structured and readable data, and the PMC index model is used to conduct quantitative evaluation research on digital economy policies in central and local areas of China.

Compared with the traditional economy, the digital economy focuses on the application of products and the extension of services, is demand oriented, focuses on discovering potential and intangible user needs, provides personalized services, and creates user value. Due to the existence of digital technology, more consumers are involved in the production and consumption of products, so the dominant position of the digital economy is relatively unclear.

The digital economy is essentially a technology economy paradigm, which is an optimal practice model for economy and society based on technological innovation. It responds to structural crises caused by technological changes through institutional changes, thus forming a relatively stable and sustainable behavior. The digital economy is driven by digital knowledge and information as key production factors, modern information networks as the main carrier, and the efficient utilization of information and communication technology as an important driving force for efficiency improvement and economic optimization. Therefore, introducing data elements and changing social production methods can provide an important experimental environment for expanding current economic and management theories; The development of the digital economy requires new theories from economics and management to explain and promote the construction of new theories.

Since China first included the digital economy in the Two Sessions Report in 2017, the national level has attached increasing importance to promoting the development of the digital economy. To address various practical issues in the development of the digital economy, various Chinese ministries have issued a series of policies and regulations. The introduction of numerous policies has also made the policy and regulatory system in this field increasingly complex, leading to ineffective implementation and poor coordination in the policy integration environment. Policy analysis is the main basis for the abolition, reform, and establishment of digital economy policies. The primary task of policy analysis is to identify and conceptualize the problems that need to be solved, and policy problems stem from unresolved practical problems within the current policy system. The evaluation and analysis based on digital economy policy texts have important practical value and significance. Therefore, when quantitatively evaluating China's digital economy policies, this article will focus on distinguishing the advantages and disadvantages of current policies based on existing practical problems, providing decision-making basis for the improvement of relevant policies and the formulation of new policies.

Reviewer #3:

I congratulates to the authors for making a significant empirical contribution in economic literature. The author implemented significant contribution in order to have their work published in the PLOS One journal.

Response: Thank you for the recognition and trust of the reviewing experts.

Reviewer #4: (No Response)

Reviewer #5:

1. The literature review provides relevant background information on the digital economy; however, it could be strengthened by incorporating more recent and diverse sources. Additionally, a critical analysis of existing literature, pinpointing the gaps the current study aims to fill, would add depth to the review section.

Response: Thanks for your suggestion. Regarding literature review, we have supplemented and modified it. The specific modifications are as follows:

The digital economy policy is a series of policy tool combinations that promote the development of the digital economy, involving multiple dimensions such as policy nature, policy objectives, policy content, policy effects, etc. How to objectively evaluate is a very important issue. At present, there is relatively little literature on the evaluation of digital economy policies, and policy evaluation is the most important link in the process of public policy formulation and management [25, 26]. It uses relevant research methods to systematically measure and judge the effectiveness of policy intervention and implementation [27]. Through the evaluation of digital economy policies, not only can scientific judgments be made on the value of the policies themselves [28], but also the actual effects of policy formulation and implementation can be tested [29]. Therefore, in view of this, this article combines digital economy policy sample analysis, text mining, and PMC index model to construct a quantitative evaluation index system for digital economy policies. It conducts sample analysis and text mining on China's digital economy policies, and quantitatively evaluates and analyzes typical digital economy policy texts at the central and local levels, in order to provide decision-making basis for the improvement of relevant policies and the formulation of new policies.

25.	Abbass K, Song H, Khan F, Begum H, Asif M. Fresh insight through the VAR approach to investigate the effects of fiscal policy on environmental pollution in Pakistan. Environ Sci Pollut Res Int. 2022;29(16):23001-14. Epub 2021/11/20. doi: 10.1007/s11356-021-17438-x. PubMed PMID: 34797543.

26.	Abbass K, Qasim MZ, Song H, Murshed M, Mahmood H, Younis I. A review of the global climate change impacts, adaptation, and sustainable mitigation measures. Environmental Science and Pollution Research. 2022;29(28):42539-59. doi: 10.1007/s11356-022-19718-6.

27.	Abbass K, Niazi AAK, Basit A, Qazi TF, Song H, Begum H. Uncovering Effects of Hot Potatoes in Banking System: Arresting Die-Hard Issues. SAGE Open. 2021;11(4). doi: 10.1177/21582440211061554.

28.	Fu H, Abbass K, Qazi TF, Niazi AAK, Achim MV. Analyzing the barriers to putting corporate financial expropriations to a halt: A structural modeling of the phenomenon. Frontiers in Environmental Science. 2022;10. doi: 10.3389/fenvs.2022.967418.

29.	Amin N, Shabbir MS, Song H, Abbass K. Renewable energy consumption and its impact on environmental quality: A pathway for achieving sustainable development goals in ASEAN countries. Energy & Environment. 2022. doi: 10.1177/0958305x221134113.

2. The paper references the use of 37 policy texts but lacks information on the selection criteria for these texts. Providing clear details on the criteria used for policy selection would enhance the transparency and credibility of the research.

Response: Thank you for the reviewer's feedback. We have revised the selection sources for the 37 policy texts. The specific modifications are as follows:

The selection criteria for digital economic policies are as follows: To ensure the comprehensiveness and authority of the policy sample content, the focus is primarily on national and provincial-level digital economic policy texts. National-level policies, being overarching documents, provide stronger guidance and standardization, serving as crucial foundations for provincial policy formulation, guiding and constraining provincial policies. The selection of provincial policies considers their coordination with the central government and effectiveness. City-level and county-level policies are often extensions and supplements to provincial policies, and therefore, they are not included in the sample selection.

The initial policy search is conducted using the "PKU Law" (Peking University Legal Information Retrieval System) legal professional database with the title "Digital Economy," specifying the policy category as currently effective, and the retrieval date as January 1, 2023. Informal decision documents such as "letters" and "administrative licensing approvals" are excluded, focusing on formal decision documents like laws, regulations, resolutions, orders, opinions, and notifications. To ensure accuracy, verification and supplementation are performed on the official websites of the central government and various provincial governments. Finally, a manual full-text review is employed to eliminate policies with little relevance to the research theme. In total, 37 policy texts were retrieved (detailed information in Supporting Information S1 and S2), including 5 national-level policies and 32 provincial-level policies. The main distribution results are presented in Table 1.

3. The paper includes a comprehensive list of variables used in the evaluation model. However, it is essential to explain the rationale behind selecting these specific variables. Clarifying why these variables were chosen and how they align with the study's objectives would strengthen the research methodology.

Response: Thanks for your advice. Building upon previous research, this study has constructed 10 primary variables: Policy Type (X1), Policy Effectiveness (X2), Policy Level (X3), Policy Field (X4), Policy Guarantee (X5), Policy Audience (X6), Policy Objectives (X7), Policy Evaluation (X8), Policy Perspective (X9) and Policy Publicity (X10). Simultaneously, by combining the results of text mining with relevant scholars' studies, 45 secondary variables were formulated. The construction and interpretation of these variables are detailed and can be referred to in Table 3, "Variable Setting of PMC Model of Digital Economy Policy," along with the notes below Table 3.

**Note: The formulation of Policy Type (X1) is based on reference [27]. The formulation of Policy Effectiveness (X2) is based on reference [28]. The formulation of Policy Level (X3) is based on reference [29]. The formulation of Policy Field (X4) is based on reference [30]. The formulation of Policy Guarantee (X5) is based on references [28], [29], and text mining results. The formulation of Policy Audience (X6) is based on references [28], [29], and text mining results. The formulation of Policy Objectives (X7) is based on references [28], [29], and text mining results. The formulation of Policy Evaluation (X8) is based on reference [30]. The formulation of Policy Perspective (X9) is based on reference [26].**

4. The paper employs the PMC Index Model for policy evaluation, which is a robust approach. However, the technical complexity of the model requires a more detailed and explanation. Providing a clear rationale for the model's construction.

Response: Thanks for your suggestion. Regarding the PMC Index Model, we have supplemented and modified it. The specific modifications are as follows:

Any single indicator can be misleading, but if multiple composite indicators point to the same result, it can provide a more accurate judgment of the evaluated thing. The PMC index first determines the meaning and level of variables at all levels, and then evaluates and analyzes the policy's advantages and disadvantages through the aggregated consistency level. This method attempts to find highly saturated secondary variables that can characterize policy characteristics and assign consistent weights to these variables to avoid subjective limitations. In addition, all variables are binary balanced, greatly simplifying the complexity of PMC index calculation.

This article uses a composite analysis method that combines policy sample analysis, text mining, and PMC index model. On the basis of sorting out China's digital economy policies, text mining is used to search for important and highly correlated text data, which forms a component of secondary indicators. Unstructured text data is transformed into structured and readable data, and the PMC index model is used to conduct quantitative evaluation research on digital economy policies in central and local areas of China.

5. The formulas (1)-(4) used for calculations are mentioned but lack detailed explanation. A thorough breakdown of these formulas and their significance is necessary for readers to grasp the calculations and interpretations fully.

Response: Thank you for the suggestions from the reviewer. The detailed explanation for formulas (1) to (4) has been modified in the "Calculation of PMC index" section. The detailed modifications are as follows:

The calculation of the PMC index model revolves around four aspects [37]. First, inputting the primary and secondary variables from the previous text into a multi-input-output table according to Formula (1). Second, sequentially assigning values to the 45 secondary variables in the digital economic policy multi-input-output table using Formula (2). The values of the secondary variables follow a [0,1] distribution, with a value of 1 assigned if they meet the evaluation criteria and 0 if they do not. Third, calculating the values of the primary variables based on Formula (3). After assigning values to the secondary variables that follow a [0,1] distribution in the digital economic policy PMC index model, the sum of the secondary variable scores is obtained. Then, dividing the sum by the number of secondary variables contained in the respective primary variable yields the arithmetic mean, representing the value of that primary variable. Finally, using Formula (4) to sum the values of each primary variable in the digital economic policy PMC index model, the overall PMC index for each digital economic policy is obtained. The detailed calculation formulas are as follows:

37.	Xu J, Zhang Z, Xu Y, Liu L, Pei T. Quantitative evaluation of waste sorting management policies in China’s major cities based on the PMC index model. Frontiers in Environmental Science. 2023;11. doi: 10.3389/fenvs.2023.1065900.

6. Information regarding the sources of data used for the analysis is missing, making it challenging to assess the reliability of the results. Additionally, it is crucial to discuss limitations related to the data sources and methodology to present a balanced view of the study's scope and potential constraints.

Response: Thanks for your suggestion. Regarding the information on data sources, we have supplemented and modified it. The specific modifications are as follows:

The selection criteria for digital economic policies are as follows: To ensure the comprehensiveness and authority of the policy sample content, the focus is primarily on national and provincial-level digital economic policy texts. National-level policies, being overarching documents, provide stronger guidance and standardization, serving as crucial foundations for provincial policy formulation, guiding and constraining provincial policies. The selection of provincial policies considers their coordination with the central government and effectiveness. City-level and county-level policies are often extensions and supplements to provincial policies, and therefore, they are not included in the sample selection.

The initial policy search is conducted using the "PKU Law" (Peking University Legal Information Retrieval System) legal professional database with the title "Digital Economy," specifying the policy category as currently effective, and the retrieval date as January 1, 2023. Informal decision documents such as "letters" and "administrative licensing approvals" are excluded, focusing on formal decision documents like laws, regulations, resolutions, orders, opinions, and notifications. To ensure accuracy, verification and supplementation are performed on the official websites of the central government and various provincial governments. Finally, a manual full-text review is employed to eliminate policies with little relevance to the research theme. In total, 37 policy texts were retrieved (detailed information in Supporting Information S1 and S2), including 5 national-level policies and 32 provincial-level policies. The main distribution results are presented in Table 1.

Regarding the discussion on research methods and limitations of data sources, we have made modifications in the section titled "Research Limitations and Future Trends." The detailed information is as follows:

It is important to note that this study primarily quantitatively evaluated China's national and provincial-level digital economic policies, excluding policies at the municipal and county levels. Due to variations in the level of digital industry development among different municipalities, a unified calculation standard has not yet been established. Therefore, future research should aim to expand the sample size and diversify the selection of subjects. Additionally, a comprehensive cross-analysis of policy content can be conducted from different dimensions by integrating policy tool theories. This approach can offer decision-makers objective and feasible solutions. Additionally, in terms of policy evaluation methods, since this study exclusively employed the PMC index model, the results are relatively singular. In the future, a more comprehensive approach should be considered, incorporating various evaluation methods rather than relying solely on a single approach.

7. on policy recommendations, a more in-depth discussion of the implications of the findings for policymakers, businesses, and other stakeholders is needed. Exploring practical applications and potential strategies based on the research findings would enhance the paper's relevance and practicality.

Response: Thanks for your suggestion. Regarding the information on the role of stakeholders in the digital economy, we have supplemented and modified it. The specific modifications are as follows:

Finally, pay attention to the role of stakeholders in the digital economy. In the era of digital economy, stakeholders have already extended to employees, users, and partners. Stakeholders should not only include shareholders, but also employees in the future. We should not only focus on shareholders, but also on employees. Only with excellent employees can we create greater value for the enterprise and achieve better development. The digital economy is built on the basis of data, and enterprises earn more profits by collecting user data. Enterprises should be regulated while using data to earn more profits. Regulators need to play a more important role in protecting user privacy and data usage. Protecting privacy requires stricter regulations to ensure it. The government is facing a new market composed of an increasing number of platforms, which poses an unprecedented challenge to its market governance. On the basis of a scientific understanding of the new economic form of the digital economy and a rational analysis of the two growth paths of the digital economy, the government should redesign the content of government management functions in the digital economy and further play a positive role.

Sincerely,

Tianzun Wang
